# Prostaglandin Analogs and Eupatilin as Treatments for Nephronophthisis

**DOI:** 10.1016/j.ekir.2025.04.060

**Published:** 2025-05-02

**Authors:** Alice Tata, Guillaume Rocha, Marguerite Hureaux, Alice S. Serafin, Esther Porée, Lucie Menguy, Nicolas Goudin, Nicolas Cagnard, Lilian Gréau, Marc Fila, Luis Briseño-Roa, Jean-Philippe Annereau, Sophie Saunier, Alexandre Benmerah

**Affiliations:** 1 Laboratory of Hereditary Kidney Disease, Imagine Institute, Université Paris Cité, INSERM UMR 1163, Paris, France; 2 Necker Bio-image Analysis Platform, Structure Fédérative de Recherche Necker, INSERM US24, CNRS UMS3633, Paris, France; 3 Bioinformatic Platform, Institut Imagine-Structure Fédérative de Recherche Necker, INSERM U1163 and INSERM US24/CNRS UAR3633, Paris Cité University, Paris, France; 4 Genomics Core Facility, Institut Imagine-Structure Fédérative de Recherche Necker, INSERM U1163 and INSERM US24/CNRS UAR3633, Paris Cité University, Paris, France; 5 Department of Pediatric Nephrology, CHU de Montpellier, Montpellier, France; 6 Medetia Pharmaceuticals, Imagine Institute, Paris, France

**Keywords:** ciliopathies, Eupatilin, kidney, nephronophthisis, primary cilia, prostaglandins

## Abstract

**Introduction:**

Primary cilia (PCs) are sensory antennae that are present on the majority of quiescent vertebrate cells where they mediate key signaling during development and in response to environmental stimuli. Defects in PCs result in a group of heterogeneous inherited disorders with overlapping phenotypes, called ciliopathies. Nephronophthisis is an autosomal recessive tubulointerstitial kidney ciliopathy with > 25 identified genes called *NPHP*. Presently, no treatment exists beyond supportive care and kidney transplant, underscoring the need for novel therapies.

**Methods:**

Using a phenotypic screening approach in cultured cell lines, we previously identified prostaglandin analogues as candidate therapeutic molecules based on their ability to rescue ciliogenesis defects in kidney tubular cells from patients with *NPHP1* . Here, we investigated the potential beneficial effects of ROCK inhibitor and Eupatilin, similarly identified by other groups in different *NPHP* contexts, in kidney cells from patients with *NPHP1* and those with *IQCB1/NPHP5* as well as in a zebrafish *nphp* mutant line (*traf3ip1/ift54*).

**Results:**

Eupatilin partially rescued *NPHP1*-associated ciliogenesis defects. Transcriptomic analyses pointed out that cell cycle progression was inhibited by Eupatilin, likely explaining its broad effects on cilia assembly. Interestingly, though ciliary defects also observed in *NPHP5* patient cells were rescued by both prostaglandins and Eupatilin, only prostaglandin analogues were able to reduce pronephric cysts size in the used *nphp* zebrafish model.

**Conclusion:**

Our study indicates that these molecules can show beneficial effects across genetic contexts and shed light on their potential as therapeutic interventions for nephronophthisis.

## Graphical abstract

**Figure:**
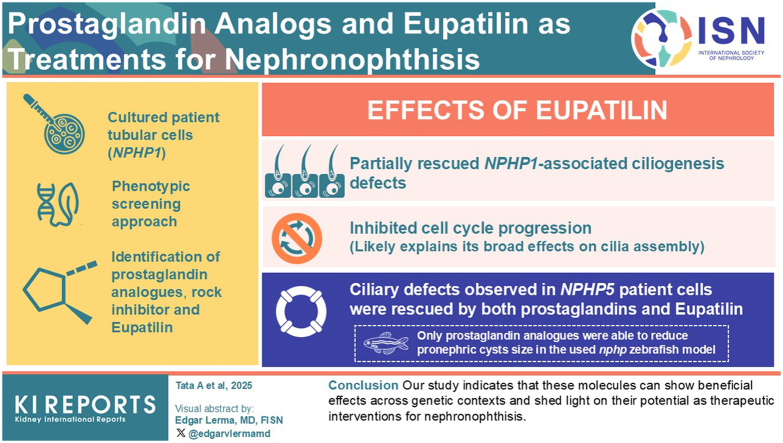

Nephronophthisis (NPH; OMIM: #256100) is a tubulointerstitial hereditary kidney disease caused by variants in genes encoding proteins functioning at different compartments of the PC, a cellular signaling hub found on most vertebrate cells, including kidney epithelial cells. It is therefore classified among ciliopathies, a complex group of disorders caused by PC dysfunction affecting various organs, including kidney, retina, the central nervous system, and the skeleton.^1^, ^2^, ^3^

PCs are assembled in quiescent cells (G_0_) from the centrosome. During this complex process, the mother centriole docks onto the plasma membrane, then called the basal body, and elongates to form the microtubule-based axoneme, which is wrapped into the ciliary membrane. The specific protein and lipid composition of the cilium is maintained by the transition zone (TZ), a structure at the base of PCs which prevents free exchange of proteins between the cytoplasm and the ciliary compartment. Most ciliary components, including ciliary membrane proteins, must therefore be actively transported by the intraflagellar transport, which selects cargos and transports them in and out along the axoneme. This process is required for ciliogenesis as well as for PC-dependent signaling, which is based on the ciliary localization of specific sets of receptors and their downstream effectors.^1^

To date, > 25 genes have been linked to NPH (*NPHPs*), which encode proteins that are playing important roles in PC-mediated signaling functions and/or ciliogenesis.^1^, ^2^, ^3^ The most frequent one is *NPHP1* accounting for nearly 25% of NPH cases, mostly harboring a homozygous deletion of the gene. NPHP1 plays an important role at the TZ together with many other *NPHP* gene products.^3^ Three main clinical subtypes have been described for NPH. Although they all lead to end-stage kidney disease, they differ by the age of onset and by the prevalence of cysts. Juvenile NPH with a mean age of onset of 13 years is by far the most frequent form, accounting for half of NPH cases and for 5% to 10% of end-stage kidney diseases in children and young adults. Biallelic loss of function or missense variants in *NPHP1*, *NPHP4*, and *NPHP5/IQCB1* are among the main causes of juvenile or late onset NPH, which can be isolated (*NPHP1*, *NPHP4*) or associated with retinal dystrophies in Senior-Løken syndrome (*NPHP1*, *NPHP4*, *NPHP5;* OMIM: #266900).^3^^,^^4^

There is no treatment available for NPH, which inevitably leads to end-stage kidney disease requiring dialysis and kidney transplant. Molecules aiming at limiting cyst growth were discovered based on studies made in cystic infantile NPH mouse models. However, those molecules are unlikely to be relevant in juvenile NPH because kidney cysts are mainly observed in the latest stages of the disease.^5^^,^^6^ Interestingly, NPH-causing variants often lead *in vitro*, depending on the cell type, to a decreased proportion of ciliated cells. Because ciliogenesis can be easily quantified by immunofluorescence image analyses, we and other groups used this rationale to identify compounds able to rescue the ciliogenesis defects observed in the context of NPH.^5^^,^^6^ Using this approach, we recently identified prostaglandin E1 (PGE1) and other agonists of PGE2 receptors (EPs) as a class of molecules that restores ciliary defects *in vitro* as well as kidney and retina-associated phenotypes *in vivo* in the context of *NPHP1*.^7^ Other groups have similarly identified additional candidate therapeutic molecules, including ROCK inhibitors and Eupatilin, in the context of *NPHP8* and *NPHP6*, respectively.^5^^,^^6^^,^^8^ The aims of the present study were to further characterize hits that we identified in our initial screen and to evaluate whether compounds identified in a given *NPHP* context could show broader efficiency in different genetic backgrounds. To achieve these aims, the effects of those compounds were tested in kidney tubular cells from patients with *NPHP1* and *NPHP5* as well as in a zebrafish model of NPH.

## Methods

### Urinary Renal Epithelial Cells

Patient and control individuals were recruited either at Necker or Montpellier Hospital, and urine samples were collected after obtaining written informed consent, and anonymized in the frame of the NPH1 protocol approved by the French National Committee for the Protection of Persons under the ID-RCB no.2016-A00541-50. Urinary renal epithelial cells (URECs) were isolated from urine from NPH patients carrying biallelic variants either in *NPHP1* or *NPHP5* and healthy age-matched donors. Control and *NPHP1* URECs were described previously.^7^ All these cell lines belong to the Imagine Biocollection (declared to the French Minister of Research under the number DC-2024-6350). Briefly, *NPHP5* URECS were immortalized by retroviral transduction of thermosensitive SV40 T-antigen (LOX-CW-CRE, Addgene) at a multiplicity of infection of 5 with 8 ug/ml polybrene reagent, and cells were subsequently grown in T75 flasks at the permissive temperature of 33 °C and 5% CO_2_ for maintenance, in UREC culture medium (REBM Basal Medium [CC-3139, Lonza, Colmar, France], 1× REGM Single Quots [CC-4127, Lonza], and 2% certified fetal bovine serum [Invitrogen, Thermo Fisher Scientific, Les Ulis, France]).

### Zebrafish

The zebrafish *traf3ip1* mutant line m649,^9^ a kind gift of Jamira Malicki, was maintained at 28.5 °C under standard conditions and according to current European legislation. Heterozygous m649 zebrafish were crossed with wild-type Tg(wt1b:GFP)^10^ to allow analysis of the proximal pronephros. The resulting line was called m649-GFP. For treatment with compounds, 10 hours postfertilization (hpf) embryos were transferred into 12-well plates (20 embryos/well) and incubated at 24 hpf for 24 hours with either Eupatilin or dmPGE2 diluted in 1% dimethyl sulfoxide (DMSO) Egg-Water in presence of phenylthiourea (to block pigmentation). Phenotypic analysis was done on 48 hpf embryos obtained from in-cross of m649-GFP heterozygous fish.

### Compounds

The compounds selected and used in this study were the following: alprostadil/PGE1 (2μM, 1620 R&D system, Noyal Châtillon sur Seiche, France), Y-27632-dihydrochloride (Sigma Aldrich, Saint-Quentin-Fallavier, France), dexamethasone 21-acetate (D1881 Sigma), isoproterenol hydrochloride (I6504 Sigma), theophylline monohydrate (c5967-84-0 Santa Cruz; Clinisciences, Nanterre, France), cefotetan disodium (A5737 Sigma), cefaclor (C6895 Sigma), simvastatin (S6196 Sigma), fluvastatin sodium hydrate (SML0038 Sigma), Eupatilin (SML1689 Sigma), and dimethyl-PGE2 (dmPGE2; sc-201240B ChemCruz; Clinisciences, Nanterre, France). They were dissolved either in DMSO (Sigma) or in water, based on their solubility.

### Ciliogenesis and Compound Treatment

For ciliogenesis assays, URECs (100,000 cells/well) were seeded in 96-well glass-bottomed plates (Sensoplates, Greiner, Courtaboeuf, France) at 39 °C, restrictive temperature in which cells stop to proliferate and ciliate. To test the selected compounds, cells were incubated at day 3 for 48 hours in the presence of each of them at the indicated concentration or with DMSO, and ciliogenesis was analyzed at day 5. For the other immunofluorescence experiments (ciliary composition), URECs (400,000 cells/well) were seeded on glass-bottomed coverslips in 24-well plates at the same temperature as for ciliogenesis.

### Immunofluorescence

Cells were fixed in either cold methanol for 5 minutes or with 4% PFA for 20 minutes, quenched in 50 mM NH_4_Cl, and permeabilized with 0.1% Triton for 15 minutes when fixed with 4% PFA. Incubation of primary and secondary antibodies was performed in phosphate-buffered saline (PBS), 0.1% Tween-20, 3% bovine serum albumin (Sigma-Aldrich, Saint-Quentin-Fallavier, France), both 1 hour at room temperature. Nuclear staining was performed using 4′,6-diamidino-2-phenylindole (1/2000e, Thermo Fisher Scientific, 62247). For ciliogenesis experiments, cells were left in PBS (96-well plates); for the other immunofluorescence, coverslips were mounted using Mowiol mounting medium on Superfrost slides (Thermo Fisher Scientific). Zebrafish embryos (48 to 50 hpf) were fixed in 4% PFA overnight at 4 °C and placed in 100% methanol gradually. The embryos were then removed from methanol and washed in PBS/triton 0.5%. Postfixation was done in PFA 4% for 20 minutes. Fixed embryos were then incubated in blocking buffer (PBS, triton 0,5%, 10% fetal bovine serum, and 2% bovine serum albumin) for at least 1 hour at room temperature. Incubation with primary and secondary antibodies were performed in blocking buffer at 4 °C overnight and at 37 °C for 2 hours, respectively. The immunostained embryos were washed during 1 day in PBS on a rocker at 4 °C. Fixed and stained embryos were mounted in 1% low gelling temperature agarose (Sigma-Aldrich, A9414) on μ-Dish 35 mm, high Glass Bottom Ibidi (81158).

### Antibodies

The following antibodies were used: acetyl-a tubulin mouse monoclonal (1/300; Sigma, T6793), ADCY3 rabbit polyclonal (1/100e; Invitrogen, PA5-35382), ARL13B rabbit polyclonal (1/800e; ProteinTech, Thermo Fisher Scientific, 17711-1-AP), ARL13B mouse monoclonal (1/100e; ABCAM, Paris, France, ab136648), INPP5E rabbit polyclonal (1/200e; Proteintech, 17797-1-AP), γ-Tubulin mouse monoclonal (1/5000e; Sigma, T6557), GT335 mouse monoclonal (1/5000e; Adipogen, Coger, France, AG-20B-0020-C100), PH3 rabbit polyclonal (1/200; Cell Signaling Technology, Ozyme, Saint-Cyr L'Ecole, 3377S), NPHP4 rabbit polyclonal (1/100e; BiCell scientific, München, Germany, 90004), and NPHP11 rabbit polyclonal (1/100e; BiCell Scientific, 90103). Secondary antibodies (donkey) conjugated to Alexa Fluor 488, 555, or 647 were used (1/1000e; Molecular Probes, Thermo Fisher Scientific).

### Image Acquisition, Quantification, and Analysis

For ciliogenesis assays, the images were acquired using the Opera Phenix microscope (40× water, Perkin-Elmer) and automated acquisition of 41 z-stack/well was performed. The percentage of ciliated cells was measured using a semiautomated workflow with Harmony software (Perkin-Elmer, Villebon-sur-Yvette, France). Images were analyzed as previously described^7^ using the building blocks approach to detect in sequence the following: nuclei (4′,6-diamidino-2-phenylindole staining); with a 20-pixel enlarged region, the basal body (γ-Tubulin staining); and then the PCs (ARL13B staining). The software segmented candidate PCs with filters (intensity, size, distance to the putative basal body, and signal-to-noise ratio). Then, multiple phenotypic parameters were calculated for every candidate cilium, using signal enhancement ratio texture (intensity patterns) and advanced STAR morphology parameters. Using the PhenoLOGIC machine-learning option of Harmony, the parameters best suited to discriminate cilia were defined and used to obtain the final detection and counting. For other assays with cells mounted on microscope slides, images were acquired using either a Spinning Disk microscope (40× or 63×, Zeiss, Rueil-Malmaison, France) or an epi-illumination microscope (Leica, DMR) with a cooled charge-coupled device camera (DFC3000G, Leica, Nanterre, France). All quantifications were performed using Fiji (v2.14.0) opensource software.^11^

For ciliary composition analyses (ADCY3, INPP5E), a semiautomated approach using Fiji (https://imagej.net/software/fiji/) coupled with ilastik (v1.3.3post3, https://www.ilastik.org/) opensource software were used. Briefly, a first macro in Fiji splits and generates Max intensity projections files for the cilium channel (ARL13B) and for the protein of which is required to calculate the abundancy along the cilium (ADCY3, INPP5E). Images of cilium channel were then charged on ilastik, a program that uses machine-learning pixel classification to recognize true versus false signal of interest, generating a simple segmentation file. A second macro uses the ilastik simple segmentation mask to generate the cilium region of interest with the possibility to check and correct it manually. The last macro in Fiji then calculates for each cilium the area and the length, plus the mean gray value and integrated density of protein of interest channel already filtered from the noise signal. Quantification of the intensity of TZ proteins were performed using Fiji.

Cysts in the proximal pronephros were analyzed on live anesthetized 48 hpf embryos using the Opera Phenix (Perkin Elmer) microscope using a 10× dry objective. GFP-positive embryos were individually positioned on the dorsal side in 96-well plates using a previously published method^12^ to perform automated imaging of the proximal pronephros with Opera Phenix HSC system (Perkin Elmer). Percentage of embryos with pronephric cysts was quantified manually (severe: bilateral big cysts; mild: unilateral or small bilateral cysts) or using Fiji to measure area of cysts with manual selection of the region corresponding to the glomerulus and neck regions of the pronephros. Fixed and immunostained embryos were imaged using a Zeiss Axio Observer Z1 inverted microscope equipped with a Yokogawa CSU-X1 spinning disk. Images were acquired with a 20× dry objective through a Hamamatsu Orca Flash 4.0 sCMOS camera (Hamamatsu, Massy, France).

### RNA Extraction and RNA-seq Analysis

URECs were seeded in 12-well plates at 39 °C grown for 3 days and then incubated for 24 hours with Eupatilin (20 μM) or DMS0 (0.20%). Cells were lysed in RLT buffer and mRNA was isolated using an Extraction Mini Kit (Qiagen, Courtaboeuf, France) following the recommendations of the manufacturer. cDNA library was prepared using the Universal Plus mRNA stranded kit (Tecan-Nugen). Then, for each sample, 50 million passing filter reads/cluster (paired-end 100+100 bases) was produced on NovaSeq6000 Illumina. FASTQ files were mapped to the ENSEMBL (Human[GRCh38/hg38]) reference using Hisat2 and counted by featureCounts from the Subread R package (http://www.r-project.org/). Read-count normalizations and group comparisons were performed using the Deseq2 statistical method.^13^ The results were filtered at *P* ≤ 0.05 and fold-change 1.2. Heatmaps were made with the R package ctc: Cluster and TreeConversion and imaged by Java Treeview software (Java Treeview). Functional analyses were carried out using Metascape (http://metascape.org/).

### qRT-PCR Analysis

Total RNA was reverse transcribed using SuperScript II Reverse Transcriptase (LifeTechnologies, Thermo Fisher Scientific) according to the manufacturer’s protocol. Quantitative real-time polymerase chain reaction (PCR) (qRT-PCR) was performed with iTaq Universal SYBR Green Supermix (Bio-Rad) on the CFX-384 Real-Time PCR System (Bio-Rad, Marnes-la-Coquette, France). Each biological replicate was measured in technical duplicates. Primers used for qRT-PCR are listed in Supplementary Table S1. For quantification and normalization, the method of multiple control genes, previously decribed,^14^^,^^15^ step 7 was used.

### Sanger Sequencing

Total DNA was isolated from *NPHP5* URECs using the NucleoSpin Mini Kit (Machery-Nalgene) following the recommendations of the manufacturer. PCR was performed with Mytaq (Neb/Biolabs). For sequencing reactions the following were used: Exosap (Thermo Fisher Scientific), Big-dye (Thermo Fisher Scientific) according to the manufacturer’s protocol. The primers were the following: Fw-exon6-CTGTATCCTCTAACTCCCCAGA, Rev-exon6-TGTGGTTATCTGCACAGTTCA, Fw-exon7-CAGAAAGTTTAGCAGAGATGGTC, and Rev-exon7-TGAAGTTCCATCACCATTGT. Genotyping of zebrafish embryos was performed by placing embryos in 10 mm Tris pH 8.0, 1 mm EDTA and 1.2 mg/ml at 55 °C overnight, and the reaction was stopped by incubating at 95 °C for 5 minutes. DNA from exon 1 was Sanger-sequenced using the following primers: forward 5′GAA-CGA-ATC-GGT-AGC-CAA-AA3′ and reverse 5′AGA-CTA-CGC-GCA-GGC-TAC-AT3′. Sequencing was performed on 3500xL Genetic Analyzer (Thermo Fisher Scientific) and analyzed using Sequencher software (Gene Codes, Ann Arbor, MI).

### Statistics

Statistical calculations were performed with R (https://www.r-project.org/) or GraphPad Prism v6. Figure representation was performed using GraphPad Prism v6 (GraphPad Software, San Diego, CA) and is presented as dot plots, box-and-whisker plots, or bar plots. Differences within the same group with data with normal distribution were analyzed using paired 2-tailed *t* test; if not possible or if data are not normally distributed, they were compared using the Wilcoxon test. Differences between the 2 groups with data with normal distribution were compared using unpaired 2-tailed *t* test. To analyze RT-qPCR data after Eupatilin treatment in cells coming from the same individuals, we used a paired 2-tailed *t* test. To analyze the effects of different compound treatments on ciliogenesis in the same cell line, a mixed linear-regression model with quasi-binomial penalization (R software: qlmmPQL function of MASS package) was used, taking into account the correlation of observations coming from the same individuals using a random effect on the cell line. Statistical tests and exact sample sizes used to calculate statistical significance are stated in the figure legends.

## Results

### Eupatilin and ROCK Inhibitor Increase Cilia Incidence in NPHP1 Patient URECs

A phenotypic screening approach previously led to the identification of small molecules able to modulate the ciliopathy-associated phenotypes. Among them, alprostadil/PGE1, a prostaglandin analogue, robustly rescued ciliogenesis in *NPHP1* patient kidney epithelial cells.^7^ Besides prostaglandins, other hits from this screen that remained to be fully investigated include statins, antibiotics (cephalosporins), glucocorticoids, and other compounds known to modulated cAMP signaling (Table 1). Those molecules were selected based on their published or potential effects on ciliogenesis. In addition, Eupatilin and ROCK inhibitors were included based on their described positive effects on the ciliogenesis defects in the context of NPH^5^^,^^6^^,^^8^ and the observed increased Rho activity in *NPHP1* patient cells.^7^ Those molecules were further tested on immortalized URECs, which were obtained from controls and NPH individuals presenting with homologous deletion of *NPHP1*.^7^

**Table 1:** List of the tested molecules and known or potential effects on cilia

Compounds	Class	Known/*expected* effect on cilia
Alprostadil	EP agonist	Rescue cilionegenesis in NPHP1 URECs^7^
Y-27632	Rock inhibitor	Positive effect on cilia in ciliopathy conditions^5^^,^^16^
Dexmethasone	Glucocorticoid	Positive effect on cilia^17^, ^18^, ^19^
Isoproterenol	β-adrenergic agonist	*Positive effect on cilia through cAMP?*
Theophylline	Phosphodiesterase inhibitor	*Positive effect on cilia through cAMP?*
Fluvastatin	HMG-CoA reductase inhibitor	Positive effect on cilia in high cholesterol conditions^20^^,^^21^
Simvastatin	HMG-CoA reductase inhibitor	Positive effect on cilia in high cholesterol conditions^20^^,^^21^
Cefotetan	Cephalosporin	Positive effect on cilia in cancer cells^17^
Cefaclor	Cephalosporin	Positive effect on cilia in cancer cells^17^
Eupatilin	Flavonoid	Positive effect on cilia in ciliopathy conditions^22^^,^^23^^,^^24^

**Figure 1:**
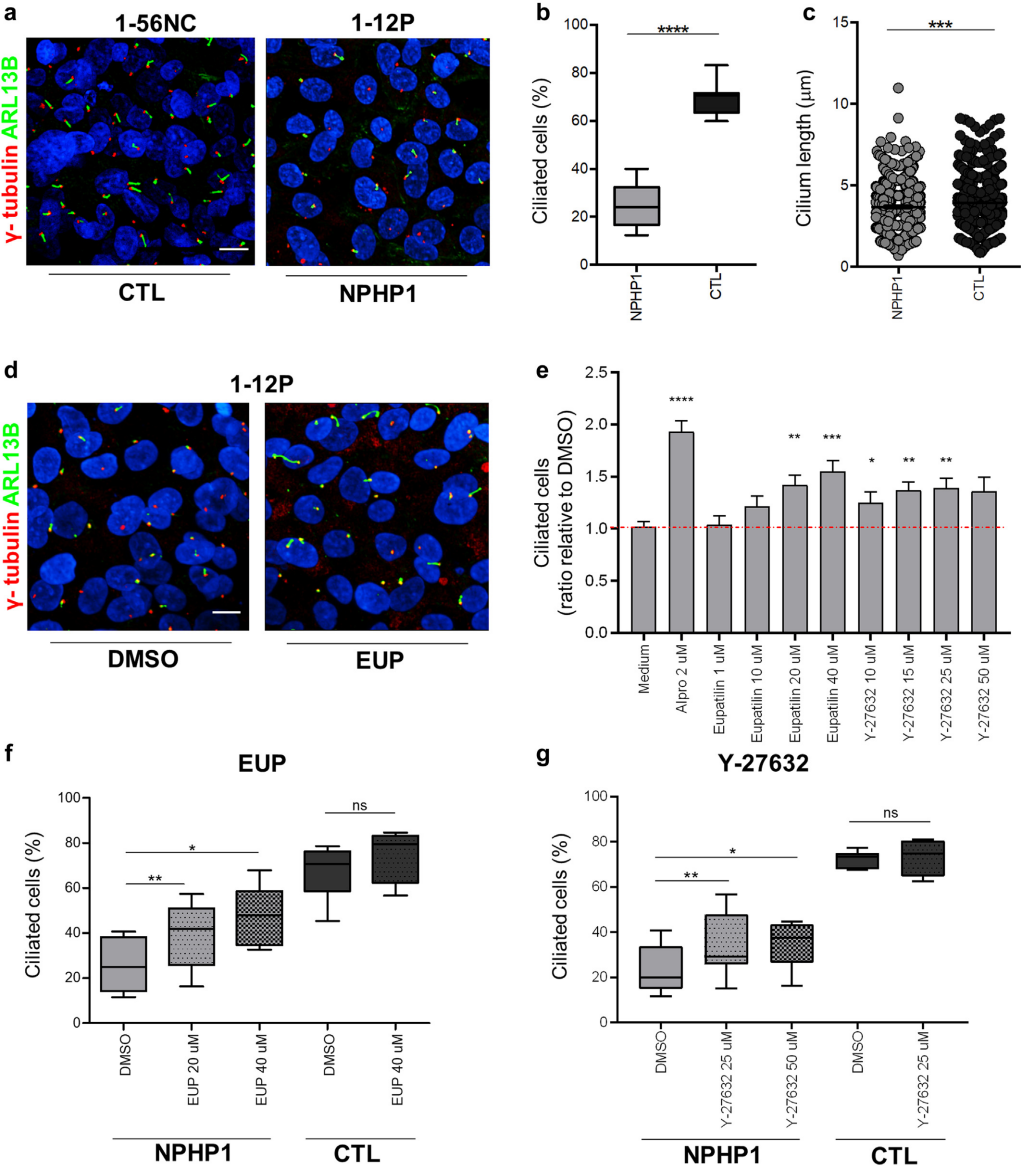
Eupatilin and ROCK inhibitors rescue ciliogenesis defects in *NPHP1* URECs. (a) Control (1-56NC) and *NPHP1* (1-12P) URECs grown in ciliogenesis conditions for 5 days at nonpermissive temperature (39 °C) were fixed and stained for primary cilia (ARL13B, green) and basal bodies (γ- tubulin, red). (b) Ciliogenesis in *NPHP1* (1-12P and 1-03P) and CTL (1-56NC) URECs were quantified using Harmony software and expressed as percentage of ciliated cells shown using a box-and-whisker plot (*n* = 5–11 experiments). Mixed linear regression model with quasibinomial penalization taking into account the correlation of observations coming from the same individuals using a random effect on the cell line: ∗∗∗∗*P* < 0.0001. (c) Cilium length in *NPHP1* (1-12P and 1-03P) and CTL (1-56NC) URECs length was quantified as explained in the Methods and expressed in μm using a dot-plot (*n* = 3 experiments). Unpaired 2-tailed *t* test: ∗∗∗*P* < 0.001. (d) *NPHP1* URECs (1-12P) treated with either DMSO (0.04%) or 20 μM EUP for 48 hours were fixed and stained as in (a) for primary cilia (ARL13B, green) and basal body (γ-tubulin, red). (e) Ciliogenesis in 1.12P and 1.03P *NPHP1* URECs treated for 48 hours with either alprostadil (Alpro), Eupatilin (EUP) or ROCK inhibitor (Y-27632) was expressed as the ratio to control DMSO-treated cells. Mean ± SEM (*n* = 4–7 experiments); paired 2-tailed *t* test: ∗∗∗∗ *P* < 0.0001, ∗∗∗ *P* < 0.001, ∗∗ *P* < 0.01, ∗ *P* < 0.05. (f and g) Ciliogenesis in URECs derived from CTL (1-56NC) and *NPHP1* patients (1-12P and 1-03P) treated for 48 hours with DMSO or EUP (20 and 40 μM; *n* = 4–6 experiments per cell line; (f) or Y-27632 (25 and 50 μM; *n* = 2–5 experiments per cell line) was quantified similarly as in (b). ∗*P* < 0.05,∗∗ *P* < 0.01. Scale bars 10 μm. CTL, control; EUP, DMSO, dimethyl sulfoxide; Eupatilin; EUP, Eupatilin; ns, not significant; UREC, urinary renal epithelial cell.

Ciliation was quantified using the Harmony software (Methods) based on ARL13B (ciliary membrane) and g-Tubulin (basal body) staining (Figure 1a). As previously shown,^7^ *NPHP1* URECs displayed reduced PC incidence compared with controls (Figure 1a and b) with shorter cilia (Figure 1c). The same *NPHP1* cell lines were treated for 48 hours with each of the selected hit compounds using alprostadil (Alpro) as a positive control. Unfortunately, none of the hits coming from the screen showed a positive effect on ciliation (Supplementary Figure S1). Interestingly, treatment of *NPHP1* URECs with Eupatilin increased ciliation (Figure 1d–f), similar to previous findings in *NPHP6* conditions.^22^^,^^23^ In addition, treatment with the ROCK inhibitor Y-27632 was able to increase ciliation in *NPHP1* URECs (Figure 1 e and g). Notably, ciliation was not increased in control cells treated with those 2 compounds (Figure 1f and g).

Altogether, those results did not confirm the beneficial effects on ciliogenesis of the compounds identified in our screen on cell lines, they however show that both Eupatilin and ROCK inhibitor can rescue ciliation defects observed in *NPHP1* patient cells.

### Eupatilin Modulates the Expression of Cell Cycle and Membrane Trafficking Pathways

Although the effects of ROCK inhibitors in the context of PC and renal ciliopathies are well-documented,^5^^,^^16^ the targets and downstream effects of Eupatilin remain poorly understood. Therefore, bulk RNA sequencing was performed on the URECs treated with either DMSO or 20 μM Eupatilin for 24 hours in ciliogenesis conditions.

**Figure 2:**
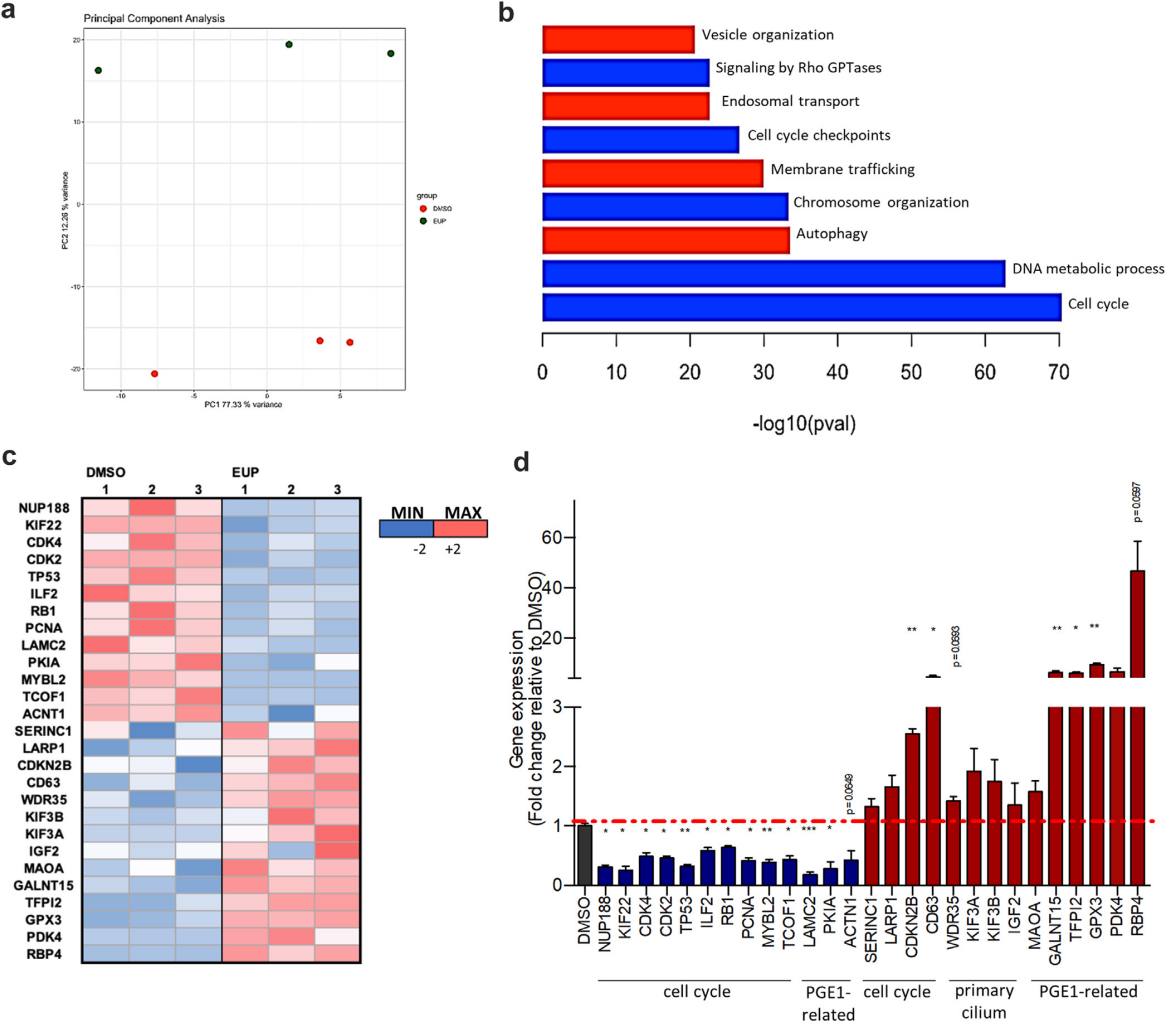
Transcriptomic analyses reveal the cellular pathways modulated in response to Eupatilin treatment. (a) Principal component analysis showing separation between the EUP-treated URECs and the DMSO-treated by PC2 variance. (b) Pertinent down (blue) or up (red) regulated pathways or relevant processes involving ciliary modulators dysregulated in *NPHP1* URECs upon 24 hours of 20 μM EUP treatment were highlighted using Metascape. (c) Heatmap generated from qPCR results of 27 selected genes from RNA-seq dataset of *NPHP1* URECs DMSO versus EUP. (d) RT-qPCR validating the effect of EUP treatment on the expression of 27 selected dysregulated genes in *NPHP1* URECs. Mean ± SEM (*n* 0 = 3 experiments). Paired 2-tailed *t* test: ∗∗∗*P* < 0.001, ∗∗*P* < 0.01, ∗*P* < 0.05. Bars indicate mean ± SEM. DMSO, dimethyl sulfoxide; EUP, Eupatilin; ns, not significant; UREC, urinary renal epithelial cells.

Clustering of the samples by significant differential expression (principal component analysis analysis) showed that the Eupatilin-treated samples had similar transcriptome profiles, distinct from DMSO-treated ones (Figure 2a); 4585 genes were differentially expressed (false discovery rate–adjusted *P*-value cutoff of 0.05), with 2041 being downregulated and 2544 upregulated (Supplementary File S1). Metascape was used to analyze the pathways modulated in response to Eupatilin (Figure 2b). Based on the adjusted *P*-value, the most significantly downregulated gene sets were related to cell-cycle progression (cell cycle, DNA metabolic process, and cell cycle checkpoints). In the G_1_-S subset (Supplementary Figure S2A and File S2), there was a decreased expression of cyclin-dependent kinase (*CDK2*, *CDK4-6*) as well as of promoters of cell-cycle progression to the S phase (*CDC25A, MYBL2* and *RB1*). In the G_2_-M subset (Supplementary Figure S2B and File S2), markers of chromosome alignment (*NUP188*, *KIF22*), cilium resorption (*KIF24*, *NEK2*), and genes related to chromosome organization and DNA repair (*PCNA*, *ILF2*) were downregulated. Interestingly, Eupatilin upregulates the expression of positive regulators of quiescence and/or genes known to be expressed in G_0_-G_1_ cells (*CDKN2B*, *SERINC* and *LARP1*; Supplementary Figure S2C and File S2). RNA sequencing results were mostly confirmed by qRT-PCR analysis (Figure 2c and d), indicating that Eupatilin treatment likely results in antiproliferative effects as previously reported.^25^, ^26^, ^27^

In addition to cell cycle progression, Eupatilin treatment downregulated Rho GTPase signaling and actin-cytoskeletal protein encoding genes (Figure 2b; Supplementary Figure S3A and File S2), in agreement with its positive effect on ciliation. Interestingly, we previously showed that Rho activity was downregulated in *NPHP1* URECs upon alprostadil/PGE1 treatment.^7^ Concerning the upregulated gene families, the most statistically significant enlightened by Metascape was “vesicular trafficking related to autophagy” (Figure 2b; Supplementary Figure S3B and File S3), which was linked to the early steps of ciliogenesis.^28^

In addition to this unbiased pathway analysis, we focused on specific gene families of interest, including ciliary genes and genes that we found modulated upon prostaglandin treatment.^7^ A list of ciliary genes was established based on Ciliacarta.^29^ Among known positive modulators of ciliogenesis, key intraflagellar transport components, including *IFT88*, *WDR19*, *WDR35*, *WDR11*, *KIF3A*, and *KIF3B*, were upregulated (Supplementary Figure S4 and File S4); however, this increased expression could not be confirmed by qRT-PCR (Figure 2c and d). Finally, among the genes that were found to be modulated by alprostadil,^7^ 169 were similarly affected by Eupatilin (Supplementary Figure S5A and B, and File S4), with some downregulated (*LAMC2*, *PKIA*, *CAV1*, and *ACTN1*) and others upregulated (*PDK4*, *ATOH8*, and *CDKN2B*), some of which were confirmed by qRT-PCR (Figure 2c and d), indicating a partially shared signature with alprostadil.

Altogether, our RNA sequencing analysis confirmed previous studies indicating the cytostatic effects of Eupatilin, which may therefore increase ciliogenesis through cell-cycle regulation favoring entry or persistence of cells in G_0_.

### NPHP5 URECs Presents Ciliogenesis Defects Similar to NPHP1 Which are Partially Rescued by Alprostadil/PGE1 and Eupatilin

In order to evaluate the potential broader effects of the molecules identified in the context of *NPHP1,* we next focused on *IQCB1/NPHP5* which was previously associated with Senior-Løken syndrome with juvenile NPH.^30^, ^31^, ^32^, ^33^, ^34^, ^35^ URECs were collected from 2 nontwin brothers (2-05P1 and 2-05P2) harboring compound heterozygous variants in *NPHP5*.^35^ Sanger sequencing confirmed the presence of the 2 loss-of-function variants in URECs, which result in decreased *NPHP5* expression (Supplementary Figure S6).

**Figure 3:**
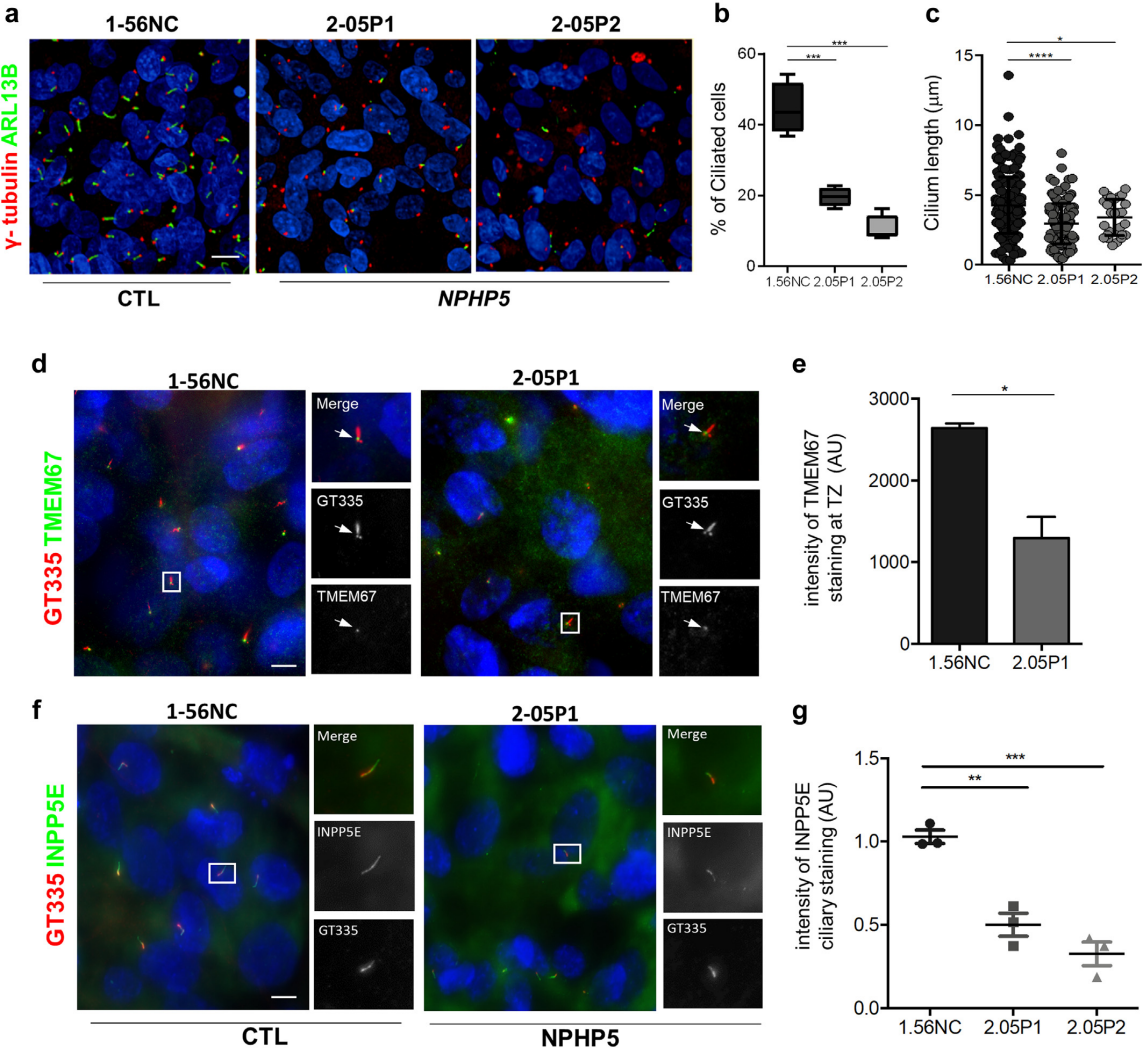
*NPHP5* URECs show ciliogenesis and ciliary composition defects. (a–c) Control (1-56NC) and *NPHP5* URECs (2.05P1, 2.05P2) grown in ciliogenesis conditions fixed and stained for primary cilia (ARL13B, green) and basal bodies (γ- tubulin, red) markers (a). (b) Ciliogenesis and (c) cilium length were quantified as in Figure 1. (b) *n* = 4 experiments; mixed linear-regression model with quasibinomial penalization: ∗∗∗*P* < 0.001. (c) *n* = 3 experiments; unpaired 2-tailed *t* test: ∗∗∗∗*P* < 0.0001; ∗*P* < 0.05. (d) Ciliated control (1.56.NC) and *NPHP5* (2.05P1) URECs were fixed and stained for basal body and axoneme (GT335, red) and for TMEM67 (green), which is a marker of the transition zone (arrows). (e) TMEM67 staining intensity at the transition zone was quantified as detailed in Methods. *n* = 2 experiments; unpaired 2-tailed *t* test: ∗*P* < 0.05. (f) Ciliated control (1.56.NC) and *NPHP5* (2.05P1) URECS were fixed and stained for basal body and axoneme (GT335, red) and for INPP5E (green). (g) Intensity of INPP5E staining in cilia was quantified as explained in Methods. *n* = 3 experiments; unpaired 2-tailed *t* test: ∗∗*P* < 0.01, ∗∗∗*P* < 0.001. Scale bars 10 μm. UREC, urinary renal epithelial cells.

Ciliogenesis was then quantified in the 2 *NPHP5* UREC lines. As expected from previous works,^36^^,^^37^ *NPHP5* URECs from both patients presented severe ciliogenesis defect and shorter cilia (Figure 3a–c). Besides ciliogenesis, *NPHP5* URECs showed partial TZ defects with decreased staining for TMEM67/NPHP11 (Figure 3d and e) whereas NPHP4 remained similar as in controls (Supplementary Figure S7A and B). As a potential consequence of the observed TZ defects, the ciliary fluorescence intensity for both ADCY3 and INPP5E, 2 widely used ciliary membrane markers, was decreased in *NPHP5* URECs compared with control (Figure 3f and g; Supplementary Figure S7C–E).

**Figure 4:**
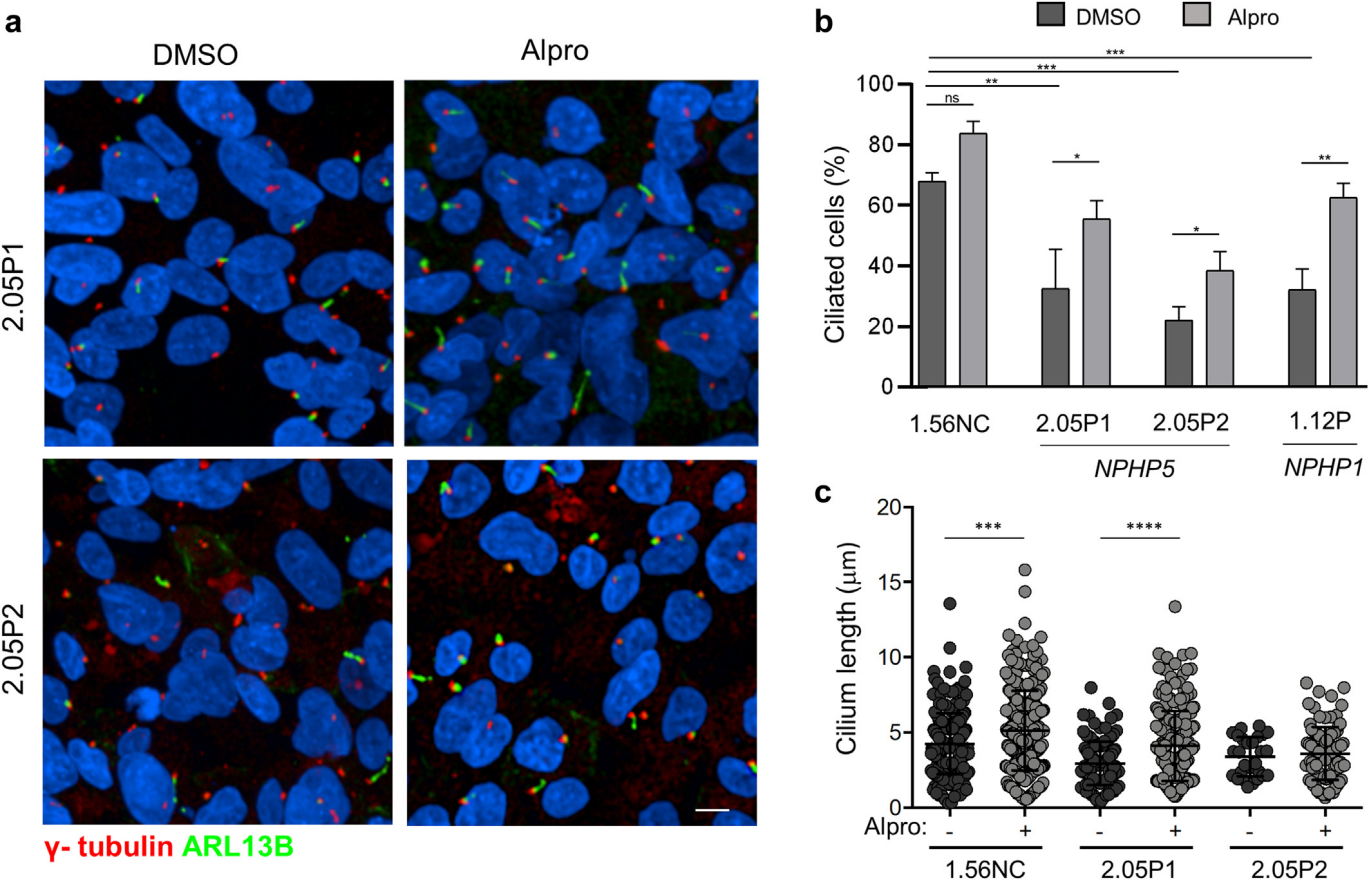
Ciliogenesis defects in *NPHP5* URECs are rescued by Alprostadil/PGE1. (a) Immunofluorescence for primary cilia (ARL13B, green) and basal bodies markers (γ- tubulin, red) of *NPHP5* URECs (2.05P1, 2.05P2) treated with 0.04% DMSO or 2 μM alprostadil (Alpro) for 48 hours. Scale bar 10 μm. (b) Ciliogenesis was quantified in control (1.56NC), *NPHP5* (2.05P1, 2.05P2) and *NPHP1* (1.12P) URECs treated with either DMSO (0.04%) or 2 μM alprostadil (Alpro), were fixed and stained similarly as in (a) (*n* = 4 experiments). Mixed linear regression with quasi-binomial penalization: ∗∗∗*P* < 0.001, ∗∗*P* < 0.01, ∗*P* < 0.05. (c) Cilium length was quantified as in Figure 1 in control (1.56NC) and *NPHP5* (2.05P1, 2.05P2) URECs treated with 0.04% DMSO (-) or 2μM alprostadil (Alpro). *n* = 4 experiments; paired 2-tailed *t*: ∗∗∗∗*P* < 0.0001, ∗∗∗*P* < 0.001, ∗∗*P* < 0.01. DMSO, dimethyl sulfoxide; UREC, urinary renal epithelial cells.

**Figure 5:**
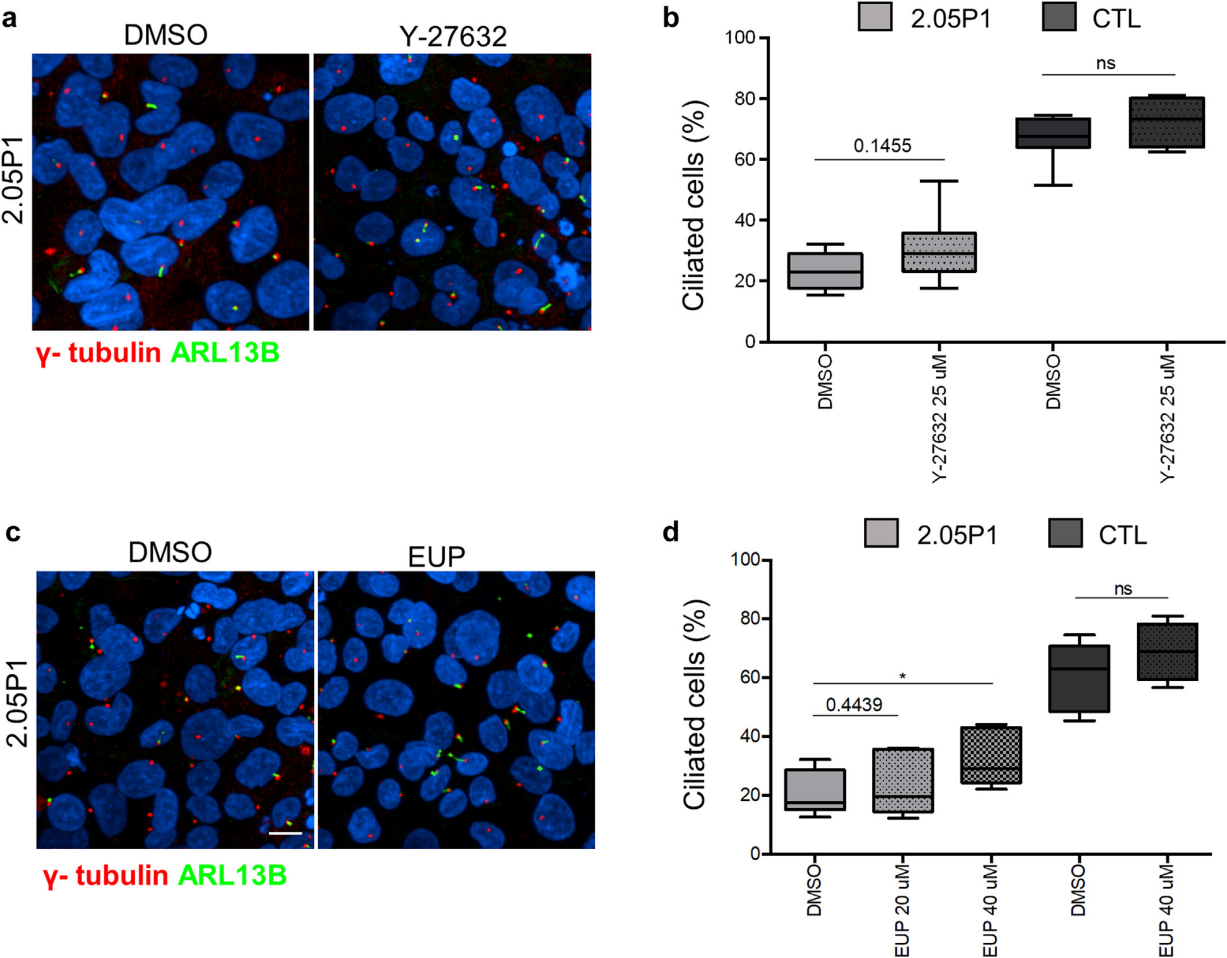
Eupatilin but not Rock inhibitor increases ciliation in *NPHP5* URECs. (a–d) Control (1.56NC) and *NPHP5* (2.05P1) URECs treated with DMSO (0,04%) or either ROCK inhibitor (Y-27632i, 25 μM; a and b) or Eupatilin (EUP, 20 μM and/or 40 μM; c and d) were fixed and stained primary cilia (ARL13B, green) and basal body markers (γ- tubulin, red; a and c). Ciliogenesis was quantified similarly as in Figure 1 (EUP, b; Y-27632, d). (b) *n* = 3 to 5 experiments, ∗∗ *P* < 0.05. (d) *n* =2 to 4 experiments. The statistical analysis were performed using R script for mixed linear-regression model with quasibinomial penalization. DMSO, dimethyl sulfoxide; EUP, Eupatilin; UREC, urinary renal epithelial cells.

In conclusion, URECs from 2 related patients with loss-of-function variants in *NPHP5* presented with ciliogenesis and ciliary composition defects similar to results obtained in *NPHP1* conditions. We therefore tested the compounds which showed positive effect on ciliogenesis in *NPHP1* URECs (Figure 1). In *NPHP5* URECs, alprostadil showed a positive effect on ciliogenesis, with a similar efficiency to the one observed in *NPHP1* cells (Figure 4a and b), as well as on ciliary length (Figure 4c). Finally, we tested Eupatilin and the Y-27632 rock inhibitor. Although the ROCK inhibitor Y-27632 did not show significant effect (Figure 5a and b), Eupatilin significantly increased ciliogenesis in *NPHP5* URECs but only at 40 μM (Figure 5c and d; in Figure 1F, we present a comparison with *NPHP1*).

Altogether, these results show that alprostadil/PGE1 and Eupatilin partially rescue ciliogenesis in the context of *NPHP5* and stress the potential wide therapeutic use of those molecules in the context of juvenile NPH.

### Prostaglandin but not Eupatilin Rescues Pronephric Cysts in a NPH Zebrafish Model

To investigate the effects of Eupatilin and prostaglandin receptor agonists in an *in vivo* model for NPH, we used zebrafish embryos, which allowed rapid testing of compounds. Zebrafish was widely used to validate *NPHP* gene candidates and to investigate the effects of identified variants with characteristic ciliopathy phenotypes, including body axis curvature and cysts in the proximal part of the pronephros.^3^ Interestingly, a *traf3ip1* mutant line (*m649*) with a variant similar to one identified in a NPH patient—Ile17Asn vs Ile17Ser—was reported but not fully phenotypically characterized.^9^^,^^38^

**Figure 6:**
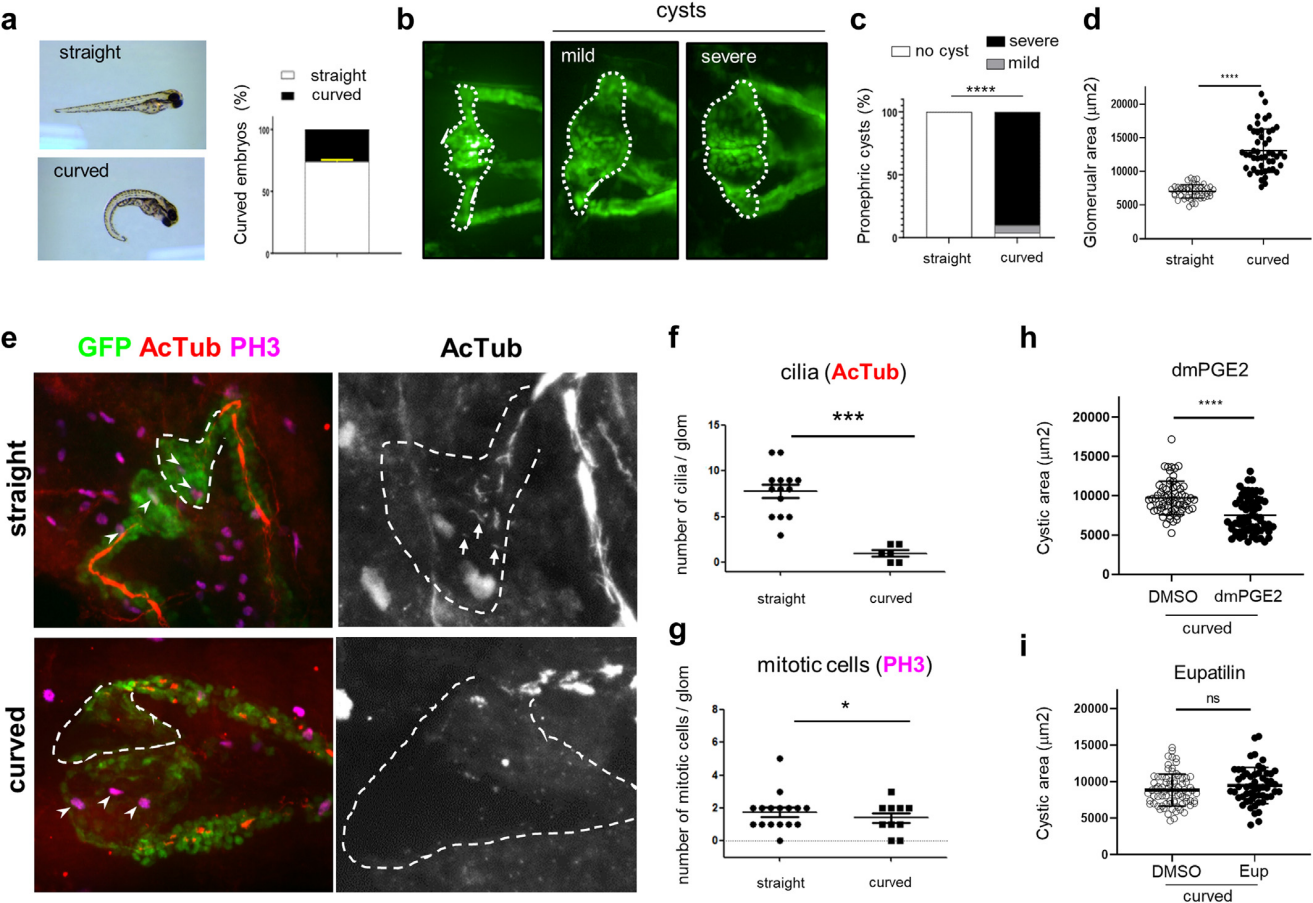
PGE2, but not Eupatilin, shows beneficial effect on cysts size in *traf3ip1* mutant fish. (a) Representative pictures obtained after crossing m649-GFP fish (48 hpf) with an example of straight (up) and curved (down) embryos. The curvature phenotype was quantified (*n* = 8 experiments). (b) Representative pictures of the GFP-expressing pronephros with, from left to right: wild-type, mild (unilateral or small), or severe (bilateral) cystic pronephros. The region of the pronephros used to quantify the cyst area is indicated by a dotted white line. (c) Quantification of cyst severity score in straight and curved embryos (*n* = 4 experiments, chi-square test: ∗∗∗∗*P* < 0.0001). (d) Quantification of cyst area measured as explained in (b) for straight and curved embryos (*n* = 4 experiments; unpaired *t* test with Welch's correction: ∗∗∗∗*P* < 0.0001; error bars representing mean ± SD. (e) Straight (upper panels) and curved (lower panels) m649-GFP embryos were fixed and stained for cilia (acetylated-αtubulin, AcTub; red) and mitotic cells (phospho-histone-H3, PH3; purple). Representative color images are shown on the left. The areas surrounded by dotted lines represent one side of the glomerulus with the initial part of the proximal tubule based on GFP staining. On the right, zoomed (×2.33) views of the same samples are shown for AcTub only. White arrowheads on the left and arrows on the right pictures indicate mitotic cells and cilia, respectively. Scale bars: 20μm. (f) Quantification of cilia number per glomerulus for straight and curved embryos. Unpaired *t* test with Welch’s correction: ∗∗∗∗ *P* < 0.0001. (g) Quantification of mitotic cells number per glomerular region for straight and curved embryos. Mann-Whitney test: ∗*P* < 0.05. (h and i) m649-GFP embryos were treated with DMSO, dimethyl-PGE2 (dmPGE2; 100 μM), or Eupatilin (2.5 μM) starting at 24 hpf. Quantification of the effects of treatments on cyst area (dmPGE2: Mann-Whitney test; ∗∗∗∗*P* < 0.0001; Eupatilin: unpaired *t* test with Welch’s correction: ns *P* > 0.05). DMSO, dimethyl sulfoxide; EUP, Eupatilin.

To facilitate the study of its impact on kidneys, the *m649* mutation was transferred in the *Tg(wt1b:GFP)* transgenic background in which GFP is expressed in the proximal part of the pronephros.^10^ Mutant embryos at 48 hpf presented with a typical ventral curvature of the body axis with the expected proportion (∼ 25%, Figure 6a). Pronephros were analyzed by confocal microscopy in 48 hpf embryos (Methods). As expected, pronephric cysts were only observed in curved fish (Figure 6b and c) with a drastic increase of the glomerular area (Figure 6d), correlated with a loss of PC (AcTub, Figure 6e and f). Interestingly, in contrast to what has been established in cystic mouse models,^39^ pronephric cysts were not associated with increased number of mitotic cells (PH3, Figure 6e and g).

We then tested both Eupatilin and prostaglandins in this validated NPH model. Treatment of mutant embryos with 100 μM dmPGE2 for 24 hours did not rescue body axis curvature but significantly decreased cystic area in curved mutant fish (Figure 6h). Because Eupatilin showed toxic effects on development when used at 40 μM (most efficient concentration in URECs, Figure 1), several concentrations were further tested starting at 1 μM at 24 hpf for 24 hours. Cardiovascular defects (edema, hemorrhages) as well as pronephros migration delay were observed at, or above 5 μM (Supplementary Figure S8). However, when used at 2.5 μM, Eupatilin did not show beneficial effects on both body axis curvature and cyst size (Figure 6i).

## Discussion

Juvenile NPH is the most common form of NPH for which, despite recent advancements, treatments remain limited to palliative care and replacement therapies. Here, we highlight the potential interest of prostaglandin analogues and Eupatilin. Our results show the broad effects of those molecules on ciliogenesis in distinct *NPHP* contexts and provide further evidence for the robust effects of PGE2 using a zebrafish NPH model. However, they failed to demonstrate any positive effect of a list of potential interesting molecules from our initial screen.^7^ Possible explanations for the lack of effect of each of the tested compounds are briefly discussed below. Before addressing these specific points, it is important to mention that the screen was conducted using *Nphp1-*knockdown kidney epithelial cell lines, including MDCK (dog) and IMCD3 (mouse), which present ciliary count and length defects (MDCK and IMCD3) as well as increased migration behavior (IMCD3). Any compound showing a rescue effect on one of these 3 phenotypes was considered as a positive hit. A striking example is alprostadil/PGE1, which shows the strongest positive effect on ciliogenesis in *NPHP1* URECs but was selected based on its ability to rescue the migration defect observed in IMCD3 cells. The differential or lack of effects of the same compounds in the different cells may be because of the variable expression of their targets in the cell lines and URECs. Indeed, whereas IMCD3 cells express mainly EP4 and EP1 PGE2 receptors, URECs predominantly express EP2 and EP4, resulting in different signaling outputs. Those differences could explain the discrepancy observed in the various phenotypic tests.

The first class of selected molecules was modulators of cAMP signaling, a pathway known for its positive effects on ciliogenesis,^40^^,^^41^ and that we demonstrated to be critical for the PGE1-mediated effects.^7^ Isoproterenol is an agonist of β2-adrenergic receptor, which is implicated in cAMP production. The adrenergic system has several crucial roles in kidney physiology; however, the expression of the different receptors is not homogeneous along the tubule.^42^ We then hypothesized that the lack of effect of isoproterenol in URECs could because of the lack or poor expression of the adrenergic receptors in those cells. Theophylline, an inhibitor of phosphodiesterases,^43^ was expected to increase cAMP through inhibition of its degradation. Its lack of effect is likely because of lower levels of cAMP in the absence of a specific stimulation of this pathway.

Another promising class of compounds investigated in our study were HMG-CoA reductases inhibitors, including fluvastatin and simvastatin, which are widely used to decrease cellular cholesterol pool. Interestingly, increased cholesterol was shown to impact cilia.^20^^,^^21^ Because statins did not show positive effects on ciliogenesis under standard UREC culture conditions with serum, a source of exogenous cholesterol, we tested those compounds in URECs grown without serum. In such conditions, URECs showed different shapes and detached from the plates, which might have introduced bias into our analysis.

Finally, we tested 2 types of molecules, which were shown to increase ciliogenesis in cancer cells in a similar phenotypic screening approach as ours.^17^ The glucocorticoid dexamethasone, previously shown to modulate cilium elongation, Hh and cAMP signaling,^18^^,^^19^ did not show positive effect on ciliation in URECs. This is likely because the medium used to grow URECs contains glucocorticoids, which may have masked any effect of similar molecules. In addition, among antibiotics of the cephalosporin family, neither cefotetan nor cefaclor showed significant effects on ciliation in URECs.

In conclusion, even though several studies indicated that the hits that we selected could be of potential interest, none of theme was able to increase ciliation in *NPHP1* URECs. It should be noticed that the screens made by other groups and ours were performed in cancer or nonhuman kidney cell lines grown in different media, which may then respond differently to the identified molecules compared with URECs.

Rho GTPase signaling leads to the activation of ROCK, which controls actin-based cytoskeleton dynamics and contractility^44^ that negatively impacts ciliogenesis leading to decreased ciliation and/or shorter cilia.^16^ Increased Rho activity has been further observed in different ciliopathy conditions where ROCK inhibitors showed positive impact on cilia *in vitro*^45^, ^46^, ^47^, ^48^, ^49^ and on kidney phenotypes in mouse models.^47^, ^48^, ^49^ Interestingly, the ROCK inhibitor Y-27632 was shown to partially rescue ciliogenesis in *NPHP1* URECs, confirming the contribution of dysregulated Rho activity signaling in *NPHP1*-associated phenotypes.^7^ It was not efficient in *NPHP5* URECs in which Rho signaling defects remain to be investigated.

Eupatilin turned out to be the most efficient among the tested molecules, showing a positive impact on ciliation in both *NPHP1* and *NPH5* URECs. This is consistent with its previously observed effects in the context of *NPHP6* and *NPHP8*.^22^^,^^23^^,^^24^ Of note, flavopiridol, a semisynthetic flavonoid, was shown to inhibit CILK1, a negative regulator of cilium length.^50^ Even though Eupatilin treatment may result in the elongation of cilia through CILK1 inhibition, it could not explain its positive impact on cilia biogenesis, because CILK1 is not involved in this process.^51^ Our transcriptomic analysis provides additional evidence for its beneficial effects on ciliogenesis. Indeed, Eupatilin mostly downregulates genes related to cell cycle progression and increases the ones required for entering into quiescence, the first step required to the cells to form PC.^52^ Interestingly, alprostadil/PGE1 treatment also leads to increased expression of p27^kip1^, a positive regulator of quiescence.^7^ Surprisingly, Eupatilin was not able to modulate cyst size in *traf3ip1* mutant zebrafish embryos. This lack of effect is likely because pronephric cysts are not associated with increased proliferation. In addition, and similarly to PGE1, Rho signaling was downregulated upon Eupatilin treatment, which likely positively contributes to its positive effects on ciliogenesis. Finally, among all the genes modulated by Eupatilin, 169 were shared with PGE1 treatment.^7^ Altogether, our results suggest a possible partial convergence in the mechanism of action of these 2 unrelated molecules. In conclusion, the present results extend our previous observations in *NPHP1* condition and demonstrate that prostaglandin also shows positive effects on ciliogenesis in *NPHP5* patient cells. They also extend the potential beneficial impact of Eupatilin, which was initially identified in the context of *NPHP6*, to additional *NPHP* background and broaden the potential use of these 2 different molecules across various NPH contexts.

## Disclosure

JPA and LBR are shareholders at Medetia Pharmaceuticals. SS, LBR, and JPA are authors in the patent application WO2109/075369A1, currently on PCT National examination phase. All the other authors declared no competing interests.

## Supplementary Material

Supplementary File (PDF) **File S1.** List of the genes modulated upon Eupatilin treatment. **File S2.** List of the cell-cycle genes modulated upon Eupatilin treatment. **File S3.** List of the autophagy genes modulated upon Eupatilin treatment. **File S4.** List of the ciliary genes modulated upon Eupatilin treatment. **Figure S1.** Test of the hits from the Prestwick library screening in *NPHP1* URECs. **Figure S2.** Heatmaps of the cell cycle genes modulated upon Eupatilin treatment. **Figure S3.** Heatmaps of the Rho and autophagy gene sets modulated upon Eupatilin treatment. **Figure S4.** Heatmaps of the ciliary gene set modulated upon Eupatilin treatment. **Figure S5.** Heatmaps of the genes commonly modulated by Eupatilin and alprostadil treatments. **Figure S6.** Characterization of URECs from 2 individuals with heterozygous compound variants in *NPHP5.***Figure S7.***NPHP5* URECs show ciliogenesis and ciliary composition defects. **Figure S8.** Determination of nontoxic dose of Eupatilin for treatment of zebrafish embryos. **Table S1.** List and sequences of the used primers.

## Footnotes

Supplementary File (PDF)
**File S1.** List of the genes modulated upon Eupatilin treatment. **File S2.** List of the cell-cycle genes modulated upon Eupatilin treatment. **File S3.** List of the autophagy genes modulated upon Eupatilin treatment. **File S4.** List of the ciliary genes modulated upon Eupatilin treatment. **Figure S1.** Test of the hits from the Prestwick library screening in *NPHP1* URECs. **Figure S2.** Heatmaps of the cell cycle genes modulated upon Eupatilin treatment. **Figure S3.** Heatmaps of the Rho and autophagy gene sets modulated upon Eupatilin treatment. **Figure S4.** Heatmaps of the ciliary gene set modulated upon Eupatilin treatment. **Figure S5.** Heatmaps of the genes commonly modulated by Eupatilin and alprostadil treatments. **Figure S6.** Characterization of URECs from 2 individuals with heterozygous compound variants in *NPHP5.* **Figure S7.***NPHP5* URECs show ciliogenesis and ciliary composition defects. **Figure S8.** Determination of nontoxic dose of Eupatilin for treatment of zebrafish embryos. **Table S1.** List and sequences of the used primers.
