# Supplementary material for: Prostaglandin Analogs and Eupatilin as Treatments for Nephronophthisis
Source: Kidney Int Rep. 2025 May 2;10(8):2821–35. doi: 10.1016/j.ekir.2025.04.060 (PMC12347849; doi:10.1016/j.ekir.2025.04.060)
Supplement: Supplementary File (PDF) — File S1. List of the genes modulated upon Eupatilin treatment. File S2. List of the cell-cycle genes modulated upon Eupatilin treatment. File S3. List of the autophagy genes modulated upon Eupatilin treatment. File S4. List of the ciliary genes modulated upon Eupatilin treatment. Figure S1. Test of the hits from the Prestwick library screening in NPHP1 URECs. Figure S2. Heatmaps of the cell cycle genes modulated upon Eupatilin treatment. Figure S3. Heatmaps of the Rho and autophagy gene sets modulated upon Eupatilin treatment. Figure S4. Heatmaps of the ciliary gene set modulated upon Eupatilin treatment. Figure S5. Heatmaps of the genes commonly modulated by Eupatilin and alprostadil treatments. Figure S6. Characterization of URECs from 2 individuals with heterozygous compound variants in NPHP5.Figure S7.NPHP5 URECs show ciliogenesis and ciliary composition defects. Figure S8. Determination of nontoxic dose of Eupatilin for treatment of zebrafish embryos. Table S1. List and sequences of the used primers. [file mmc1.pdf]

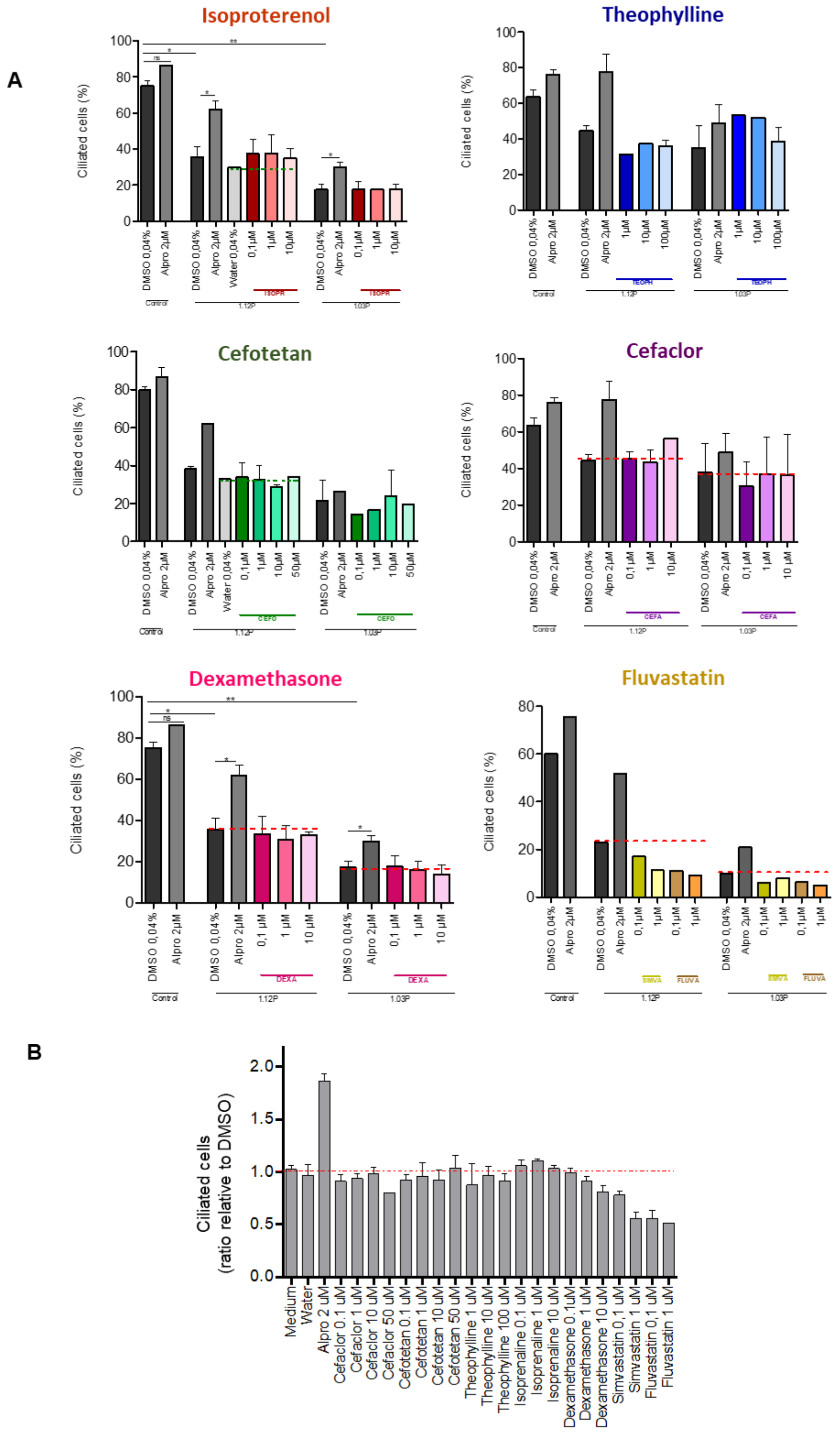

Figure S1: Hits from the Prestwick library screening in *NPHP1* URECs. (A) Control (1-56NC) and *NPHP1* (1-12P, 1.03P) URECs grown in ciliogenesis conditions for 5 days at non permissive temperature (39°C) were fixed and stained for primary cilia (ARL13B, green) and basal bodies (γ-tubulin, red) to score for ciliogenesis after compound treatment at day 3 for 48 hours in complete medium except for Statins. **(B)** Same dataset as in **A**, showing ciliated cells as ratio over DMSO condition after compound treatment. All experiments are n=2. Statistic test explained in Methods.

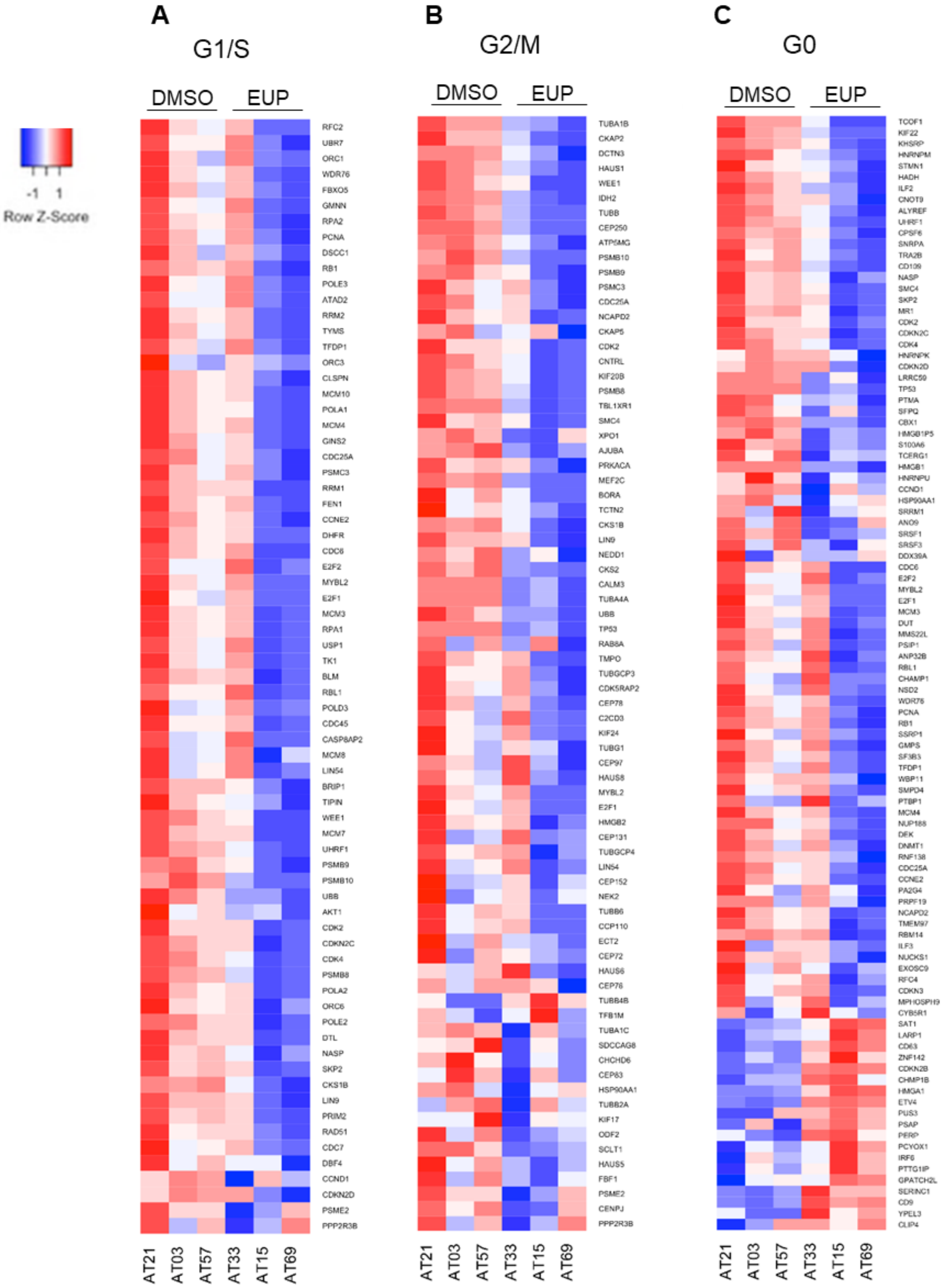

Figure S2: Heatmaps of the cell cycle genes modulated upon EUP treatment. (A) Heatmap showing differential expression of cell-cycle G1/S specific genes from RNA-seq dataset of *NPHP1* URECs DMSO versus EUP. **(B)** Heatmap showing differential expression of cell-cycle G2/M specific genes from RNA-seq dataset of *NPHP1* URECs DMSO versus EUP. **(C)** Heatmap showing differential expression of cell-cycle G0/G1 specific genes from RNA-seq dataset of *NPHP1* URECs DMSO versus EUP.

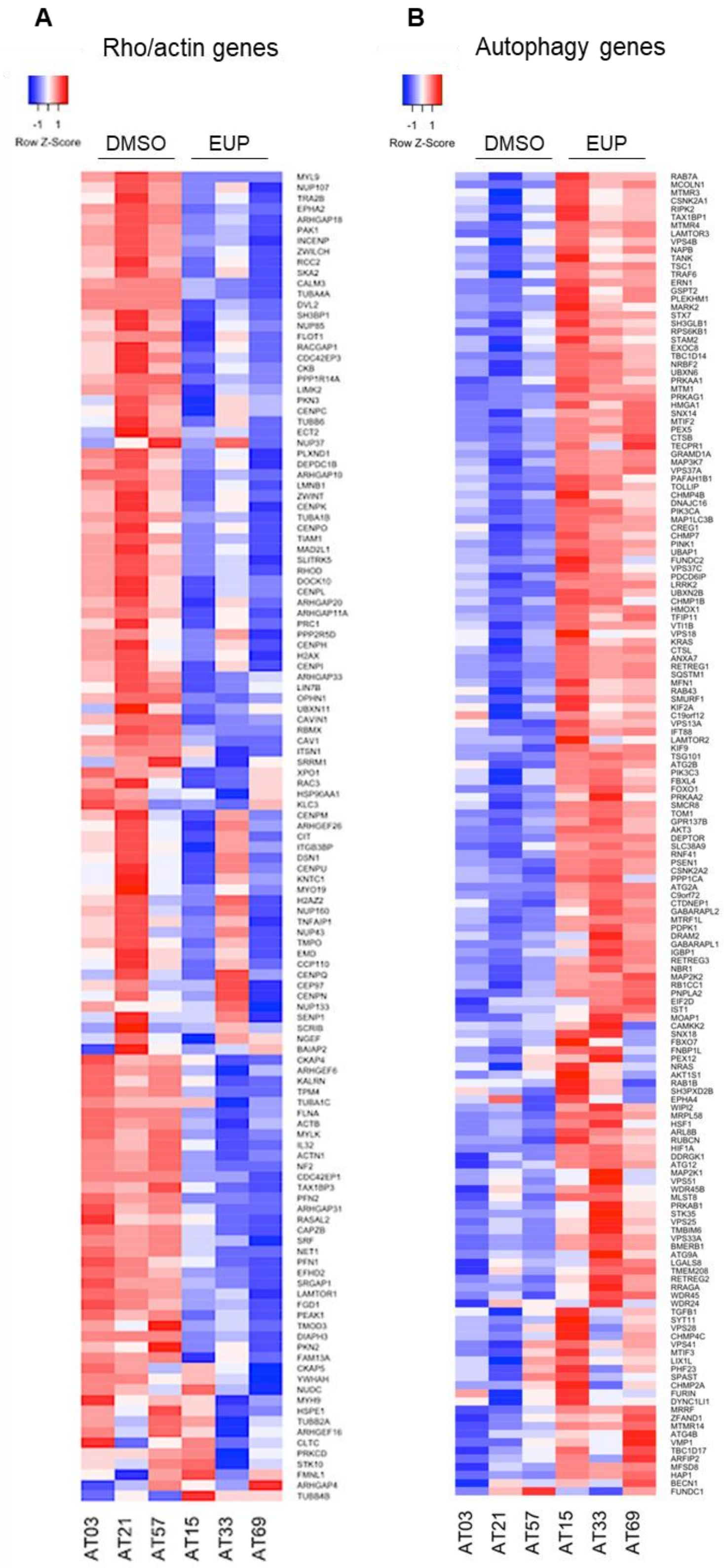

Figure S3: Heatmaps of the RHO and Autophagy gene sets modulated upon EUP treatment. (A) Heatmap showing differential expression of Rho/actin specific genes from RNA-seq dataset of *NPHP1* URECs DMSO versus EUP. **(B)** Heatmap showing differential expression of autophagy specific genes from RNA-seq dataset of *NPHP1* URECs DMSO versus EUP.

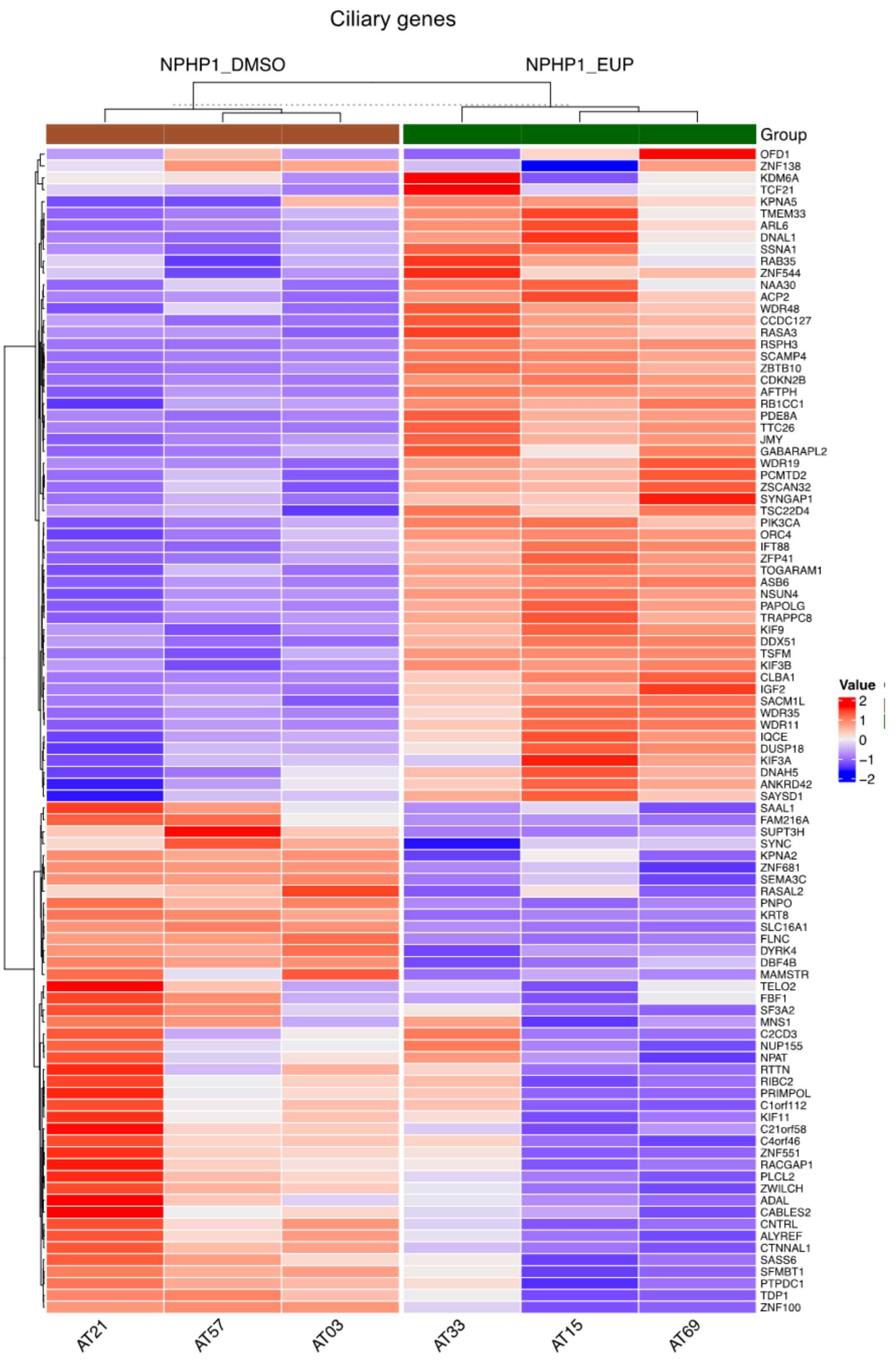

Figure S4: Heatmaps of the ciliary gene set modulated upon EUP treatment. Heatmap showing differential expression of ciliary specific genes from RNA-seq dataset of *NPHP1* URECs DMSO versus EUP (An exhaustive list is in supplementary File S4).

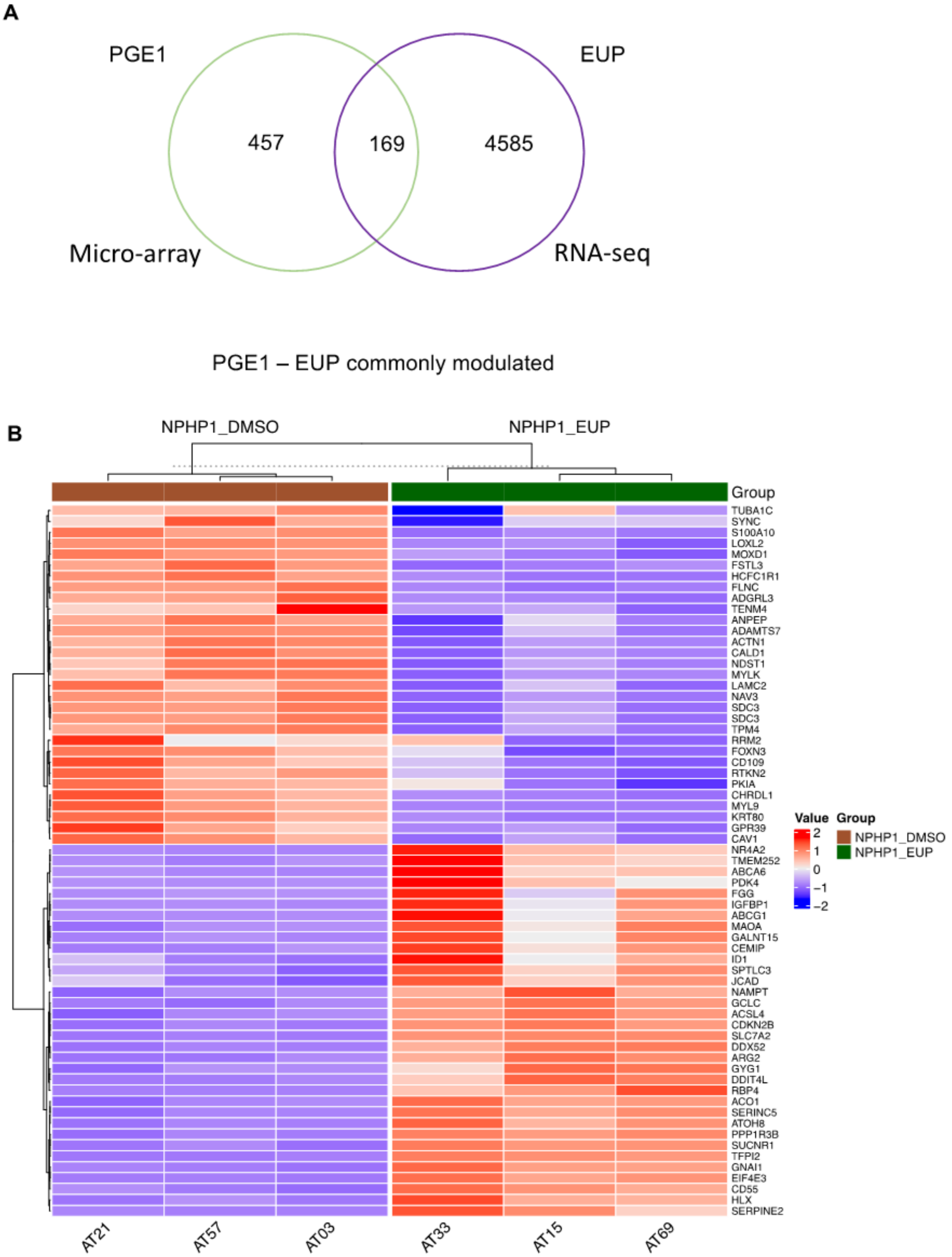

Figure S5: Heatmaps of the genes commonly modulated by EUP and ALP treatments. (A) Venn diagram showing genes modulated by EUP and ALP treatments. **(B)** Heatmap showing differential expression of genes in common with ALP treatment, from RNA-seq dataset of *NPHP1* URECs DMSO versus EUP (An exhaustive list is on File S4).

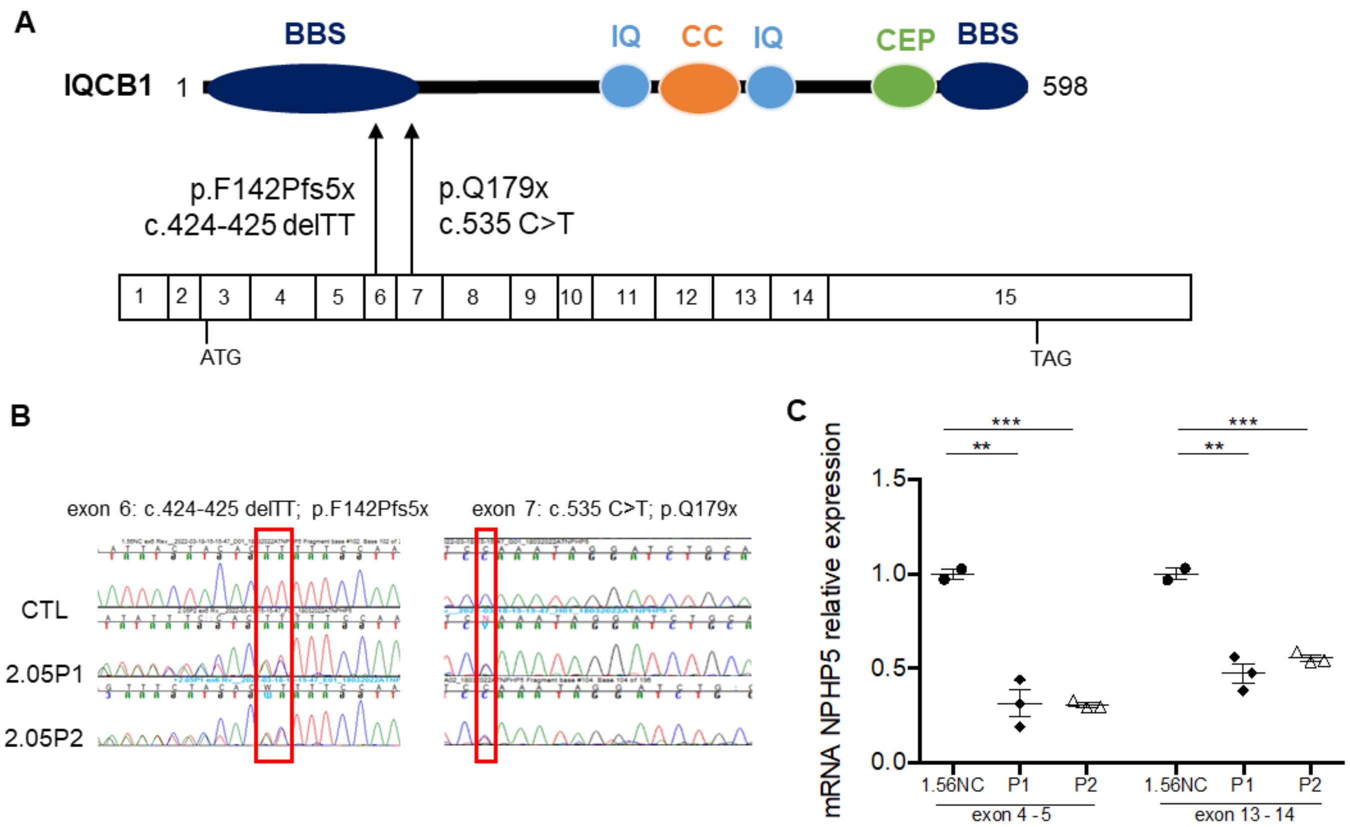

Figure S6: Characterization of URECs from two individuals with heterozygous compound variants in *NPHP5.* (A) Schematic representation of IQCB1/NPHP5 protein domains and exons with the position of the variants identified in 2.05P1 and 2.05P2 affected individuals. **(B)** *NPHP5* variants identified in in 2.05P1 and 2.05P2 patients was validated in URECs by Sanger sequencing (boxed in red). **(C)** Relative expression of *NPHP5* mRNA was quantified by qRT-PCR using primers specific for two different exons 4-5 and exons 13-14 junction regions. Unpaired t-test $^{**}P < 0.01$, $^{***}P < 0.001$.

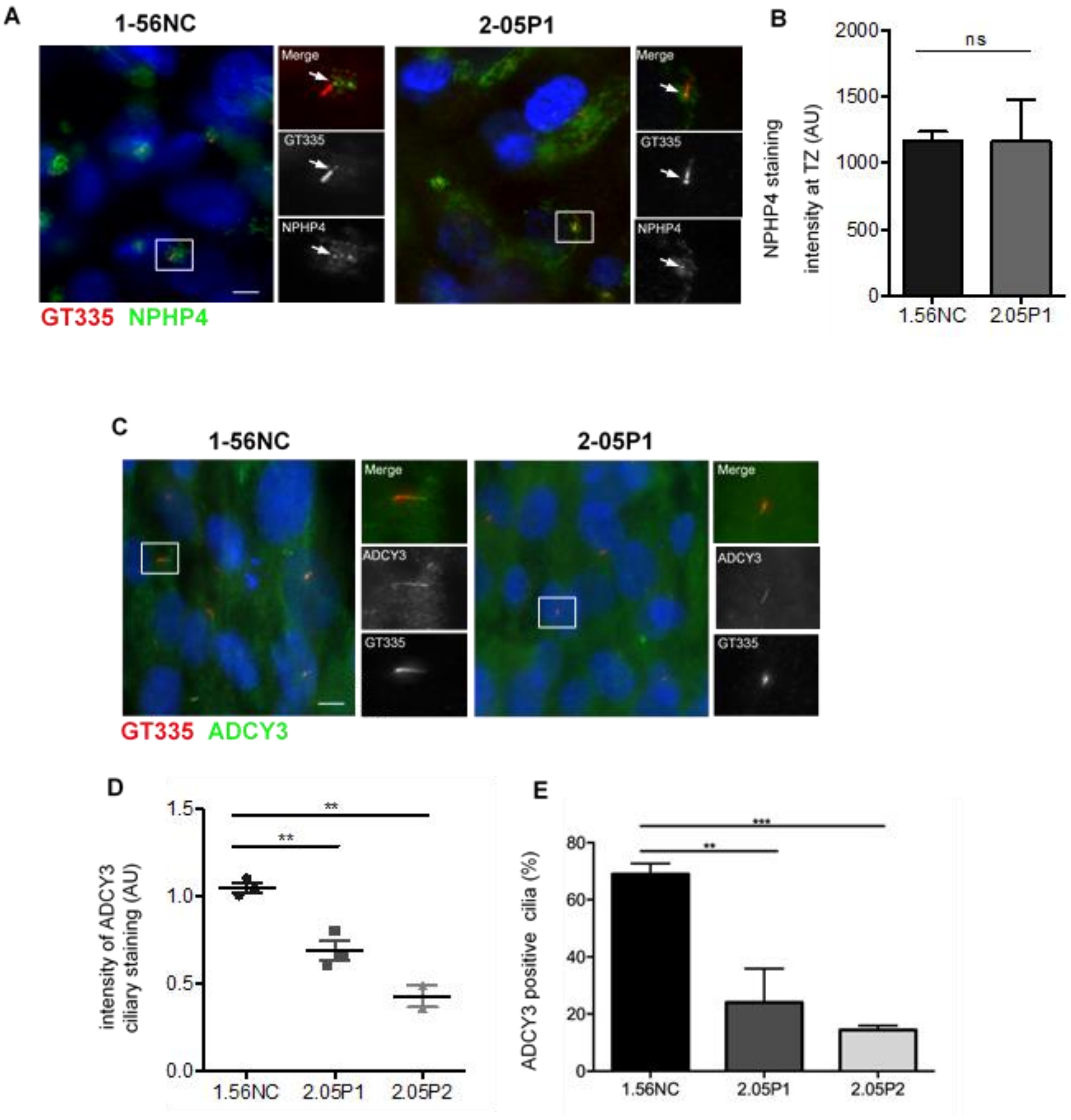

Figure S7: *NPHP5* URECs show ciliogenesis and ciliary composition defects. (A) Ciliated control (1.56.NC) and *NPHP5* (2.05P1) were fixed and stained for basal body and axoneme (GT335, red) and for ADCY3 (green). **(B)** Intensity of ADCY3 staining in cilia was quantified as explained in Methods. n=3; unpaired t test, $**P < 0.01$, Scale bars 10μm. **(C)** Positive cells for ADCY3 staining in cilia was quantified as explained in Methods. n=3; unpaired Student's t test, $**P < 0.01$, $***P < 0.001$. **(D)** Ciliated control (1.56.NC) and *NPHP5* (2.05P1) fixed and stained for basal body and axome (GT335, red) and for NPHP4 (green) which is a marker of the transition zone (arrows). **(E)** NPHP4 staining intensity at TZ was quantified as detailed in the Methods, n=2.

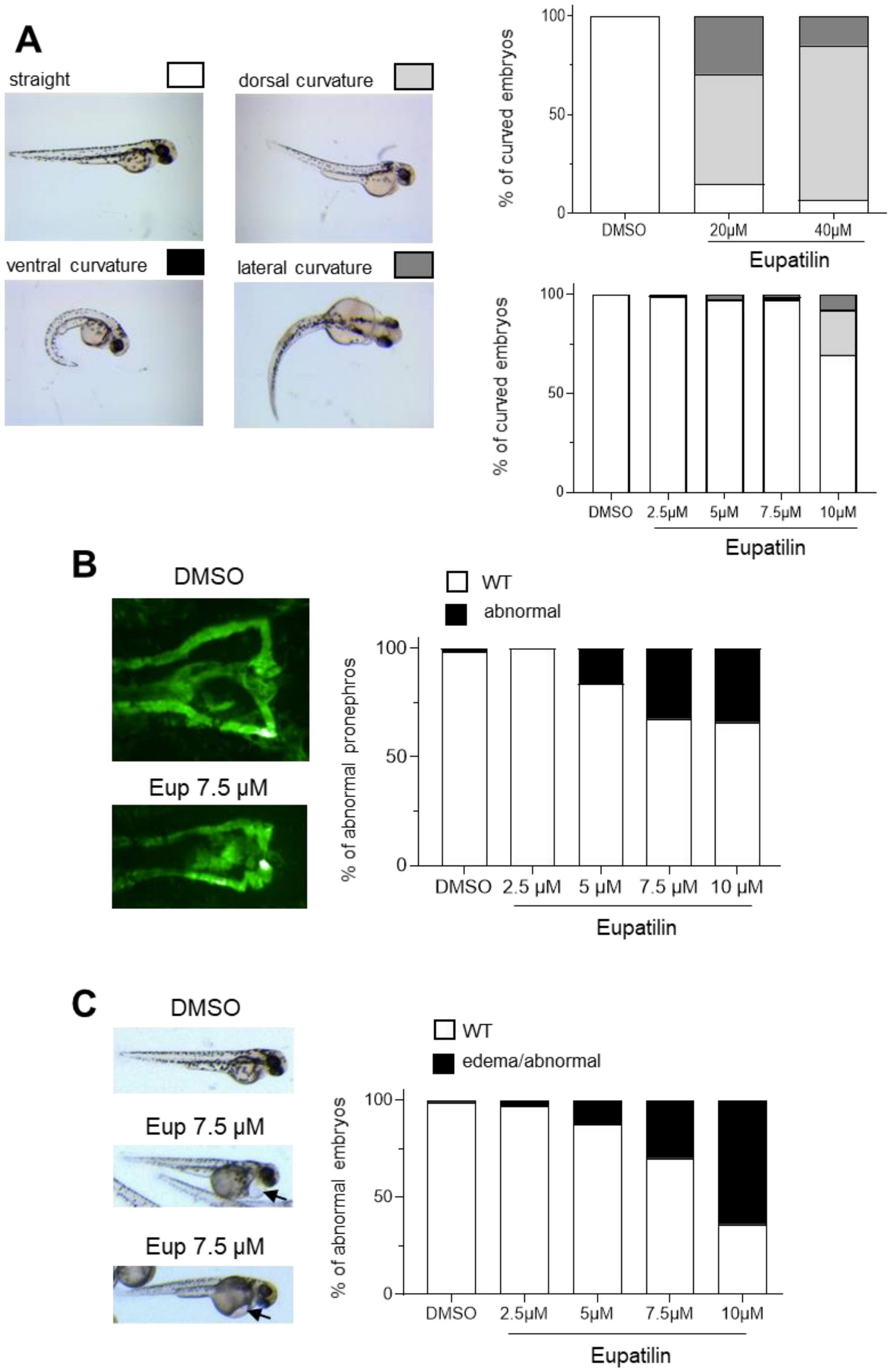

Figure S8: Determination of nontoxic dose of Eupatilin for treatment of zebrafish embryos. (**A-C**) Wild type *Tg(wt1b:GFP)* zebrafish embryos were treated with Eupatilin (Eup) at the indicated concentrations for 24 hrs starting at 24 hpf. (**A**) The impact of Eupatilin on body axis morphology was quantified as percentage of embryos presenting with dorsal, ventral or lateral curvature. (**B**) Proximal pronephros was imaged thanks to GFP expression and the effect of Eupatilin treatment on morphogenesis was quantified and expressed as percentage of abnormal pronephros. (**C**) The presence of edema and/or yolk misorganization (arrows) was quantified and expressed as the percentage of abnormal embryos.

Supplementary Table 1: List and sequences of the used primers.

Gene Name	Sequence
CDKN2B-F	CACCCCCACCCACCTAATTC
CDKN2B-R	TGAGTGTCGAGGGCCAGATA
WDR35-F	CAACTCTAGCCGTCTTGCTATC
WDR35-R	TCTTTGGCCCACTTCATATCC
KIF3A-F	GTGGACCTTTCTCACGTGTATC
KIF3A-R	CGCTTTCTTCTCCCTGTCTTT
KIF3B-F	GGAGAACCAGCAGATGATGAA
KIF3B-R	GTCCAGCTCTAACAGCACAA
IFT88-F	TGGCAGAAGACCTCCAATAAC
IFT88-R	CTGCTGTCATGGGTCTAGTAAC
WDR19-F	ACACATTGTACCCATCCTGAC
WDR19-R	GACCATTCCCTCGATCTTCTTT
SERINC1-F	CAGCATCCGTACTTCAAACAATAG
SERINC1-R	TGACACCATCCCTTTCATTATCT
LARP1-F	CGCCAAAGAAGGCTACAGATA
LARP1-R	CAGAACTTCTCCAGCCCATAC
CD63-F	GTTTGCCATCTTTCTGTCTCTTATC
CD63-R	ATCGAAGCAGTGTGGTTGT
ILF2-F	GCAGGACATGGTCTGCTATAC
ILF2-R	CTTCTTCTCTGGTGGCTTCTC
KIF22-F	AAGAACTGGAGGCCAAGATG
KIF22-R	GCTGCCTGCTCCTGAATTA
NUP188-F	GTGGATGTCATTGCTTCTTGTG
NUP188-R	TTCATCCCTTCCGCACTAATC
TCOF1-F	CCAAGGCAGAGACAGAGAAAG
TCOF1-R	CAGTTTCTGAGACCAACGTAGT
TP53-F	GGAAGAGAATCTCCGCAAGAA
TP53-R	CACGGATCTGAAGGGTGAAATA
MYBL2-F	CACTACCAGGACACAGATTCAG
MYBL2-R	GTCAGTGCGGTTAGGGAAG
RB1-F	CACAACCCAGCAGTTCGATA
RB1-R	CAACATGGGAGGTGAGAGTTT
RBP4-F	GACAGCTACTCCTTCGTGTTT
RBP4-R	GTAACCGTTGTGGACGATCA
MAOA-F	CAGGAACGGAAGTTTGTAGGT
MAOA-R	TTCATGGTTCAGCGTCTCTATG
IGF2-F	CGTGCTTCCGGACAACTT
IGF2-R	CGTTTGGCCTCCCTGAAC
GPX3-F	TCCTTCCTACCCTCAAGTATGT
GPX3-R	AGAAGAGGCGGTCAGATGTA

TFPI2-F	GGCCCTACTTCTCCGTTACTA
TFPI2-R	TTTCTATCCTCCAGCAAGCATC
GALNT15-F	TCTGGAAAGCTCCACAACAC
GALNT15-R	GCCAAAGTGAATCTCCTTCCT
ATOH8-F	TGACTACAGTGCCGACCACA
ATOH8-R	TCACTCCTTGCGCTTCTTGG
PDK4-F	GGTGGTGTTCCCCTGAGAAT
PDK4-R	GGCAAGCCGTAACCAAAACC
LAMC2-F	CGCAGCTCTGCAGAATACAG
LAMC2-R	AGACCCATTTCGTTGGACAG
CAV1-F	CCAAGGAGATCGACCTGGTCAA
CAV1-R	GCCGTCAAAACTGTGTGTCCCT
PKIA-F	TGATATCCTGGTTTCCTCTGCA
PKIA-R	GGCTTCCCCACTTTGTTCTG
TUBB-F	GAAGCCACAGGTGGCAAATA
TUBB-R	CCCAGACTGACCAAATACAAAGT
MYLK-F	CAACCTCACTGTCGTGGATAAG
MYLK-R	TCCCAGATCTCGATGCTGTA
ACTN1-F	TGGGTTACAACATGGGAGAAG
ACTN1-R	CATGACTTGGTCTGCTGTATCT
LAMB1-F	ACTGTTTCCAGGGAGTGTATG
LAMB1-R	CCAAGCACCTTTCACAGTTATG
CDK4-F	GAAGTTCTTCTGCAGTCCACATA
CDK4-R	CAGCCCAATCAGGTCAAAGA
CDK2-F	GAGTTGTGTACAAAGCCAGAAAC
CDK2-R	ACATCCAGCAGCTTGACAATA
PCNA-F	AGGAGGAAGCTGTTACCATAGA
PCNA-R	AGTGTCCCATATCCGCAATTT
NPHP5-exon4-5-F	TGCCTCTTGGTCCTCAGTCA
NPHP5-exon4-5-R	GCCCACACAGCAATGGCTTA
NPHP5-exon13-14-F	TCAGAAGACATTTGGGCTCTCC
NPHP5-exon13-14-R	TCGTTCTTGAGCTTGGGCAT

Supplementary file S1

ID	NPHP1_DMSO_mean	NPHP1_EUP_mean	AveExpr	LogFC	deseq_adj_p	genename	symbol	
ENSG00000132693	0,022294415	4,17803001	0,533405186	-7,349206194	1,83E-11	C-reactive protein	CRP	**RED: UP**
ENSG00000186281	0	0,011144091	1,457732162	-6,493375869	1,74E-08	glycerol-3-phosphate acyltra	GPAT2	**GREEN:DOWN**
ENSG00000138207	1,64172338	134,4774656	8,456628582	-6,22058733	2,35E-05	retinol binding protein 4	RBP4	
ENSG00000004799	2,124720693	91,39814508	5,661725117	-6,081644264	1,24E-06	pyruvate dehydrogenase kir	PDK4	
ENSG00000171557	0,409432916	10,13584863	1,175643492	-5,501665362	2,32E-09	fibrinogen gamma chain	FGG	
ENSG00000198829	0,032987901	1,3766888	-0,458291986	-5,432718741	3,03E-05	succinate receptor 1	SUCNR1	
ENSG00000229807	296,2856779	516,4617323	6,520973128	-5,214412094	0,000633998	X inactive specific transcript	XIST	
ENSG00000116833	0,225331467	5,540212027	0,348704286	-5,05715603	1,60E-08	nuclear receptor subfamily 5	NR5A2	
ENSG00000146678	0,171007613	5,537681282	2,417035236	-4,972973463	3,31E-07	insulin like growth factor bir	IGFBP1	
ENSG00000171564	24,21211193	340,6895706	6,638122234	-4,884669024	0,004048614	fibrinogen beta chain	FGB	
ENSG00000154262	0,209184548	5,459522559	1,069099248	-4,856723323	7,42E-15	ATP binding cassette subfan	ABCA6	
ENSG00000198535	1,77298689	23,93657893	2,700485753	-4,785879998	8,76E-07	C2 calcium dependent doma	C2CD4A	
ENSG00000175426	2,923198804	49,76296522	3,247965948	-4,532041066	1,49E-10	proprotein convertase subti	PCSK1	
ENSG00000153162	0,591020762	10,04352805	1,737937281	-4,529631377	5,17E-20	bone morphogenetic protei	BMP6	
ENSG00000253301	0,186348112	1,957169096	-1,041475391	-4,47934116	0,002553455	long intergenic non-protein	LINC01606	
ENSG00000171724	3,278790963	17,9437834	3,060646961	-4,455856113	2,62E-10	vesicle amine transport 1 lik	VAT1L	
ENSG00000123838	0,103616058	1,254555135	-1,134450917	-4,296958168	0,000872514	complement component 4 b	C4BPA	
ENSG00000171560	17,79980011	182,4152464	6,137795399	-4,235742376	0,008627015	fibrinogen alpha chain	FGA	
ENSG00000258819	0,082468954	1,501062378	0,730754041	-4,187083056	7,93E-08	long intergenic non-protein	LINC02289	
ENSG00000181778	0,15803049	2,38739777	0,146435802	-4,181692215	0,002850836	transmembrane protein 252	TMEM252	
ENSG00000084674	0,333843192	3,61140813	0,964343655	-4,104872275	8,32E-07	apolipoprotein B	APOB	
ENSG00000115353	3,301561926	9,578599815	1,871027879	-3,974803041	1,93E-07	tachykinin receptor 1	TACR1	
ENSG00000050628	0,506456425	6,015340383	1,647524793	-3,951712527	0,000461501	prostaglandin E receptor 3	PTGER3	
ENSG00000086696	2,026711854	22,85846508	3,422713989	-3,912461165	1,15E-05	hydroxysteroid 17-beta deh	HSD17B2	
ENSG00000164794	0,207098879	2,000996207	-0,686451189	-3,892957185	6,06E-07	potassium voltage-gated cha	KCNV1	
ENSG00000166523	2,547482543	31,58569758	3,878828372	-3,882795266	0,011624735	C-type lectin domain family	CLEC4E	
ENSG00000184454	3,82365253	39,69002945	3,876119285	-3,789350433	0,004398977	non-compact myelin associa	NCMAP	
ENSG00000124491	0,812734138	4,784385339	0,285318376	-3,752254915	8,78E-17	coagulation factor XIII A cha	F13A1	
ENSG00000237238	4,432588949	11,23420072	2,08872318	-3,587575533	8,32E-09	BMS1 pseudogene 10	BMS1P10	
ENSG00000123610	0,808259958	9,425963521	2,410343225	-3,580483611	9,10E-07	TNF alpha induced protein 6	TNFAIP6	
ENSG00000012504	0,218092326	2,292821975	1,419056682	-3,569977523	0,014406461	nuclear receptor subfamily 1	NR1H4	
ENSG00000131386	7,024687487	80,60680903	6,801076094	-3,546983396	3,97E-09	polypeptide N-acetylgalacto	GALNT15	
ENSG00000160179	2,951414689	33,19261107	4,5995065	-3,512416382	0,000655445	ATP binding cassette subfan	ABCG1	
ENSG00000186198	0,885757969	4,666018754	0,393370721	-3,507225225	0,000134039	solute carrier family 51 subu	SLC51B	
ENSG00000105825	61,98889868	600,4140919	8,603014457	-3,501382527	8,16E-20	tissue factor pathway inhibi	TFPI2	
ENSG00000154153	3,528821539	35,20358678	4,952933592	-3,444437444	1,15E-14	reticulophagy regulator 1	RETREG1	
ENSG00000073737	0,218676028	1,796380386	-0,271855842	-3,334275346	0,000132785	dehydrogenase/reductase 9	DHRS9	
ENSG00000249853	0,104260912	0,648864917	-1,089978023	-3,315407893	2,97E-06	heparan sulfate-glucosamin	HS3ST5	
ENSG00000283597	0,18072945	1,509301474	0,123390014	-3,286171165	0,010252493	protein FAM169B	FAM169B	
ENSG00000124564	0	0,055744286	-1,205342565	-3,213950439	0,022532898	solute carrier family 17 men	SLC17A3	
ENSG00000154258	0,077190872	0,705797117	-0,657259296	-3,143216605	2,70E-07	ATP binding cassette subfan	ABCA9	
ENSG00000197614	6,677520126	33,04802107	5,030715041	-3,138621859	6,00E-09	microfibril associated protei	MFAP5	
ENSG00000103888	0,257918605	2,27430012	1,147087438	-3,12747227	3,08E-12	cell migration inducing hyalu	CEMIP	
ENSG00000100181	14,89765136	30,98830637	4,437951658	-3,101951563	0,008194886	TPTE pseudogene 1	TPTEP1	
ENSG00000182916	8,390773064	13,78322316	4,370917921	-3,050113509	0,001092626	transcription elongation fact	TCEAL7	
ENSG00000257057	0,065191558	0,391570335	-1,927614776	-3,034958595	0,018151322	chromosome 11 open readi	C11orf97	
ENSG00000153234	4,174990876	34,21247256	4,848300231	-3,001766321	2,94E-09	nuclear receptor subfamily 4	NR4A2	
ENSG00000234695	0,21964442	1,734298449	0,318512632	-2,981605986	1,99E-07	TFPI2 divergent transcript	TFPI2-DT	
ENSG00000175899	0,727194023	3,95687569	1,232286467	-2,95608761	0,009669188	alpha-2-macroglobulin	A2M	
ENSG00000177508	3,564477268	27,77298108	6,277307052	-2,948393907	0,005089419	iroquois homeobox 3	IRX3	
ENSG00000119125	17,89673318	137,9820053	7,20114715	-2,920519867	0,00037035	guanine deaminase	GDA	
ENSG00000112299	0,973812058	7,171641984	1,773674062	-2,869274926	0,002960082	vanin 1	VNN1	
ENSG00000115009	0,63709843	2,934367236	1,587378311	-2,831090368	7,78E-06	C-C motif chemokine ligand	CCL20	
ENSG00000121966	5,950528247	40,72638242	4,558454178	-2,74213059	6,86E-06	C-X-C motif chemokine rece	CXCR4	
ENSG00000137266	4,741036315	16,04279798	3,219705636	-2,738000264	3,26E-14	solute carrier family 22 men	SLC22A23	
ENSG00000104760	0,109632897	0,683057604	-0,88004379	-2,730187549	0,013708635	fibrinogen like 1	FGL1	
ENSG00000176165	1,567313834	2,556122044	3,054442915	-2,72230903	0,000428034	forkhead box G1	FOXG1	
ENSG00000176887	1,761939974	4,354153653	1,163575041	-2,707676286	0,048768546	SRY-box transcription factor	SOX11	
ENSG00000108932	3,440717225	21,15833482	4,723758902	-2,685576529	0,005623789	solute carrier family 16 men	SLC16A6	
ENSG00000131398	1,152740827	4,464141123	2,428460843	-2,661573053	0,003161848	potassium voltage-gated cha	KCNC3	
ENSG00000165646	0,311889149	1,530585322	-0,03765858	-2,647458638	0,011406877	solute carrier family 18 men	SLC18A2	
ENSG00000133048	4,014788058	22,52682376	4,363710594	-2,63885528	6,67E-06	chitinase 3 like 1	CHI3L1	
ENSG00000172000	0,837160049	1,287137992	-0,115968103	-2,55436276	0,035737427	zinc finger protein 556	ZNF556	
ENSG00000129988	0,207402945	1,086407696	-0,218470905	-2,546606667	0,003642344	lipopolysaccharide binding p	LBP	
ENSG00000267327	0,530211636	0,963346781	-0,633857256	-2,534230984	0,001443203	uncharacterized LOC107985	LOC107985164	
ENSG00000211445	112,1497332	532,8280011	9,496540743	-2,533796277	1,02E-06	glutathione peroxidase 3	GPX3	
ENSG00000231701	0,546774339	1,85700186	0,887913922	-2,528940979	1,97E-05	BMS1 pseudogene 13	BMS1P13	
ENSG00000088386	0,711799329	3,031588869	1,963188094	-2,450342084	0,024763005	solute carrier family 15 men	SLC15A1	
ENSG00000136630	13,79360041	57,77459732	5,688571112	-2,440888815	5,67E-07	H2.0 like homeobox	HLX	
ENSG00000144057	0,092978207	0,435789947	-0,434076241	-2,42898909	0,020351219	ST6 beta-galactoside alpha-2	ST6GAL2	
ENSG00000172201	1,501236521	7,242698505	2,524998954	-2,420337835	5,08E-12	inhibitor of DNA binding 4, H	ID4	
ENSG00000197520	0,175759442	0,905523834	0,10167952	-2,413863531	8,89E-08	family with sequence similai	FAM177B	
ENSG00000043355	0,153460172	0,671254015	-1,038112975	-2,367240154	0,000299176	Zic family member 2	ZIC2	
ENSG00000106688	36,55333047	153,2094559	6,983389472	-2,365932479	2,57E-24	solute carrier family 1 meml	SLC1A1	
ENSG00000127324	0,093322363	0,391629919	-1,006749517	-2,358849672	0,023435769	tetraspanin 8	TSPAN8	
ENSG00000128052	3,863145052	14,82919270	2,799417314	-2,354201374	0,006542732	kinase insert domain recept	KDR	
ENSG00000236699	0,110014549	0,492699066	-0,972165122	-2,347321109	0,029785859	Rho guanine nucleotide excl	ARHGEF38	
ENSG00000204876	0,356368844	1,597824114	0,577954036	-2,335763618	0,005790573	uncharacterized LOC389602	LOC389602	
ENSG00000167157	11,3886544	35,05352947	4,792106531	-2,331551931	0,000201431	paired related homeobox 2	PRRX2	
ENSG00000145358	8,628379863	40,95331157	5,268608452	-2,323238459	1,34E-08	DNA damage inducible trans	DDIT4L	
ENSG00000178726	1,50564391	7,398063332	2,713170664	-2,311933237	1,84E-07	thrombomodulin	THBD	
ENSG00000139044	4,035930852	16,88284852	3,554308053	-2,302882386	0,035427225	beta-1,4-N-acetyl-galactosar	B4GALNT3	
ENSG00000003436	117,4772233	490,2896875	9,636537198	-2,300800947	0,006356191	tissue factor pathway inhibi	TFPI	
ENSG00000039139	4,277073258	16,15024614	3,788060505	-2,291015524	0,000168705	dynein axonemal heavy chai	DNAH5	
ENSG00000198795	0,425078718	1,107163129	0,044768737	-2,236367572	0,002941346	zinc finger protein 521	ZNF521	
ENSG00000003989	74,18616986	346,4093932	8,670824109	-2,230373145	9,24E-06	solute carrier family 7 meml	SLC7A2	
ENSG00000125798	2,01204212	9,289686661	2,639275557	-2,21533333	0,000956787	forkhead box A2	FOXA2	
ENSG00000162618	19,53224009	61,25169664	6,179045117	-2,206216485	0,00012598	adhesion G protein-coupled	ADGRL4	
ENSG00000084110	0,088161427	0,425014524	-0,117328142	-2,195504872	0,002905457	histidine ammonia-lyase	HAL	
ENSG00000144668	0,695335287	2,825214664	1,390625252	-2,156806145	3,54E-05	integrin subunit alpha 9	ITGA9	
ENSG00000166342	12,80639225	52,11337748	5,607850038	-2,148382603	2,63E-26	neuropilin and tolloid like 1	NETO1	
ENSG00000183091	0,392111242	1,128839479	0,745151738	-2,142189231	0,003045543	nebulin	NEB	
ENSG00000064989	3,207078758	11,05746781	3,675090237	-2,120003177	0,000179901	calcitonin receptor like rece	CALCRL	
ENSG00000136869	14,34274542	35,00347528	5,791220488	-2,047107412	0,02233594	toll like receptor 4	TLR4	
ENSG00000108551	111,7749132	483,258643	9,328228622	-2,032477904	0,022171389	ras related dexamethasone	RASD1	
ENSG00000172789	0,174362409	0,547771937	-0,260006945	-2,0216418	0,021222843	homeobox C5	HOXC5	
ENSG00000249641	0,389585944	1,097605035	0,12193356	-2,009748131	0,001403697	HOXC13 antisense RNA	HOXC13-AS	
ENSG00000163412	16,68222237	66,53353459	6,163974854	-1,993436622	7,76E-08	eukaryotic translation initiat	EIF4E3	
ENSG00000123364	2,72351927	6,230764305	3,213182175	-1,992755507	0,002453612	homeobox C13	HOXC13	
ENSG00000179071	1,306411679	2,830204655	1,755975648	-1,965452899	0,005338708	coiled-coil domain containin	CCDC89	
ENSG00000261760	0,208334893	0,749619399	-1,169630647	-1,954189332	0,005673492	two pore channel 3 pseudog	LOC100507334	
ENSG00000165591	0,224125589	0,703913954	-0,583887135	-1,937584272	0,012648365	fatty acid amide hydrolase 2	FAAH2	
ENSG00000147408	7,874843922	30,29035196	5,528406887	-1,935458024	0,000383078	chondroitin sulfate N-acetyl	CSGALNACT1	
ENSG00000234840	0,286903459	1,051828843	0,250636259	-1,916540727	0,001131709	long intergenic non-protein	LINC01239	
ENSG00000266992	0,071874982	0,234833984	-1,147193347	-1,906725504	0,017078241	DEAH-box helicase 40 pseuc	DHX40P1	
ENSG00000263958	0,671710124	2,237945355	1,0876044	-1,903579456	2,33E-09	NETO1 divergent transcript	NETO1-DT	
ENSG00000173805	5,967718315	21,30743015	5,009091468	-1,896043561	0,001574121	huntingtin associated protei	HAP1	
ENSG00000118946	0,186159572	0,659622842	-0,402603964	-1,868570693	0,027869524	protocadherin 17	PCDH17	
ENSG00000167972	37,02460219	113,6459936	7,117875955	-1,868065601	2,85E-06	ATP binding cassette subfan	ABCA3	
ENSG00000205795	6,974939415	25,33974176	4,028068685	-1,848306894	0,000563408	cystin 1	CYS1	
ENSG00000165379	0,485415324	0,794225177	0,007154353	-1,831398786	0,011909554	leucine rich repeat and fibrc	LRFN5	
ENSG00000127928	0,273696102	0,604336222	-1,088564267	-1,830049616	0,012783611	G protein subunit gamma tr	GNGT1	
ENSG00000196352	51,25953702	168,3426469	7,35643781	-1,788415445	2,41E-21	CD55 molecule (Cromer bloc	CD55	

ENSG00000118526	3,040468055	8,777476839	4,330736961	-1,786851853	0,000174285	transcription factor 21	TCF21
ENSG00000185565	1,843786841	2,081042169	1,710758374	-1,769782588	0,013847056	limbic system associated me	LSAMP
ENSG00000169429	23,35281046	69,52316509	6,860029004	-1,767147557	5,55E-06	C-X-C motif chemokine ligan	CXCL8
ENSG00000160712	13,9409999	45,04321242	5,921422873	-1,763651961	0,029723073	interleukin 6 receptor	IL6R
ENSG00000134539	0,611711345	1,768188387	2,063888546	-1,763042985	0,040115215	killer cell lectin like receptor	KLRD1
ENSG00000223387	0,10989925	0,34675639	-0,407014991	-1,762542521	0,008625368	long intergenic non-protein	LINC02068
ENSG00000121858	169,8107014	479,3764717	9,114824056	-1,758823041	0,001977335	TNF superfamily member 1C	TNFSF10
ENSG00000167244	81,87670877	257,0826156	10,06311749	-1,753099376	0,000434019	insulin like growth factor 2	IGF2
ENSG00000111913	0,680394303	2,239091936	1,761296184	-1,747174711	0,003292659	RHO family interacting cell p	RIPOR2
ENSG00000188277	3,689527261	11,67894326	3,964246776	-1,742800308	0,00145784	chromosome 15 open readi	C15orf62
ENSG00000110848	0,935648558	2,05010414	0,230138686	-1,735997628	0,010197986	CD69 molecule	CD69
ENSG00000168589	0,115042583	0,36925832	-0,544626534	-1,705416156	0,01137632	dynein light chain roadblock	DYNLRB2
ENSG00000143869	1,892117932	5,570857719	2,57528575	-1,70383182	5,74E-06	growth differentiation facto	GDF7
ENSG00000265386	0,104584313	0,324206793	-0,600152206	-1,700393899	0,032821172	RNA, 7SL, cytoplasmic 219, p	RN7SL219P
ENSG00000162493	54,37403301	80,36002567	7,811540028	-1,689176242	7,21E-06	podoplanin	PDPN
ENSG00000240990	2,007599088	3,546379113	2,975644973	-1,673314569	0,04689235	HOXA11 antisense RNA	HOXA11-AS
ENSG00000167861	6,945098157	16,0929077	4,13405363	-1,664092197	0,000165533	HID1 domain containing	HID1
ENSG00000065361	7,63113308	23,046034	4,758163048	-1,65501712	0,014094904	erb-b2 receptor tyrosine kin	ERBB3
ENSG00000158473	0,341895054	1,072435693	1,279084717	-1,65446452	0,005861049	CD1d molecule	CD1D
ENSG00000134323	0,981715145	2,736553821	2,086055763	-1,649628652	0,049245597	MYCN proto-oncogene, bHL	MYCN
ENSG00000159167	259,5901375	704,0325629	9,234763428	-1,641046318	2,86E-13	stanniocalcin 1	STC1
ENSG00000151715	0,319689119	0,997332054	-0,07643674	-1,637901479	0,003809405	transmembrane protein 45E	TMEM45B
ENSG00000185477	9,151045674	26,75308193	4,899959736	-1,630657088	0,03796193	GPRIN family member 3	GPRIN3
ENSG00000259158	0,115793123	0,357775731	-0,470582664	-1,625379576	0,014263751	ADAM metallopeptidase doı	ADAM20P1
ENSG00000277399	0,165651361	0,491962485	0,614028495	-1,613290363	0,003773385	G protein-coupled receptor	GPR179
ENSG00000150347	99,31526858	277,1908986	8,69094542	-1,612246907	9,35E-07	AT-rich interaction domain 5	ARID5B
ENSG00000112096	572,4316679	1696,093371	11,20350774	-1,609171027	5,17E-16	superoxide dismutase 2	SOD2
ENSG00000168874	6,593790943	20,0163425	5,587824552	-1,600405032	1,28E-08	atonal bHLH transcription fa	ATOH8
ENSG00000135929	41,75091208	126,6751106	7,419908354	-1,595907359	3,88E-09	cytochrome P450 family 27	CYP27A1
ENSG00000189221	26,32200585	79,84481193	7,099789969	-1,580867793	0,002639454	monoamine oxidase A	MAOA
ENSG00000256576	0,192126482	0,558512363	0,271629699	-1,577846504	0,023520032	long intergenic non-protein	LINC02361
ENSG00000059377	19,28851671	50,28897335	5,64590873	-1,575307936	0,035567372	thromboxane A synthase 1	TBXAS1
ENSG00000121743	1,335002956	3,201969134	1,661742754	-1,574273222	0,016132285	gap junction protein alpha 3	GJA3
ENSG00000132429	1,010025975	2,715912003	2,437366775	-1,574173173	8,07E-08	popeye domain containing 3	POPDC3
ENSG00000169302	0,545728383	1,184387514	0,640513122	-1,572796761	0,020126918	serine/threonine kinase 32A	STK32A
ENSG00000198691	3,293079274	7,084853552	2,897642565	-1,567825876	0,001492475	ATP binding cassette subfam	ABCA4
ENSG00000127920	71,34691167	207,2115246	8,205678138	-1,564929181	2,08E-26	G protein subunit gamma 11	GNG11
ENSG00000081181	8,40526261	24,68505663	4,638978429	-1,549714239	0,000505945	arginase 2	ARG2
ENSG00000248323	0,510805717	1,376837383	0,984807382	-1,547276756	0,008641717	lung cancer associated trans	LUCAT1
ENSG00000138670	0,527190268	1,455220266	0,688230017	-1,537989981	0,027766346	RasGEF domain family mem	RASGEF1B
ENSG00000206195	0,932097738	1,367380205	3,605154951	-1,535969442	0,003374369	double homeobox A pseudo	DUXAP8
ENSG00000110090	10,60403712	29,56552308	5,892739748	-1,532583718	0,000442054	carnitine palmitoyltransfera	CPT1A
ENSG00000147883	56,87039324	163,2930559	7,830766939	-1,517796583	5,44E-18	cyclin dependent kinase inhi	CDKN2B
ENSG00000076716	76,72392578	184,7525834	8,448061315	-1,51625937	0,011342738	glypican 4	GPC4
ENSG00000134762	29,38211398	50,53983282	6,6715895	-1,485527299	0,010804101	desmocollin 3	DSC3
ENSG00000068366	141,1018543	391,9055477	9,304930166	-1,479935603	6,78E-09	acyl-CoA synthetase long ch	ACSL4
ENSG00000236404	0,544716406	1,533983734	1,867604469	-1,479070949	0,012755125	VLDLR antisense RNA 1	VLDLR-AS1
ENSG00000236548	0,143176842	0,391172252	-0,312957573	-1,476842554	0,009929805	RNF217 antisense RNA 1 (he	RNF217-AS1
ENSG00000108688	0,213297574	0,480586393	-0,36174428	-1,46453616	0,005849549	C-C motif chemokine ligand	CCL7
ENSG00000184408	2,262559144	4,538958759	3,428115347	-1,434833239	2,61E-10	potassium voltage-gated cha	KCND2
ENSG00000146374	1,105017279	2,302949816	1,932582644	-1,433094925	0,010053632	R-spondin 3	RSPO3
ENSG00000244306	1,327133012	2,038854304	3,668236248	-1,421759148	0,018890518	double homeobox A pseudo	DUXAP10
ENSG00000205502	0,611144107	1,645418304	0,665550008	-1,415602503	0,006520446	C2 calcium dependent doma	C2CD4B
ENSG00000219755	0,247288095	0,603979472	0,399060996	-1,410349526	0,004316492	ribosomal protein S3 pseudo	RPS3P5
ENSG00000141441	17,90082788	45,63437612	6,192594454	-1,409025051	1,44E-08	GRB2 associated regulator o	GAREM1
ENSG00000089199	17,89110139	45,27640286	5,278251495	-1,403177743	0,022330708	chromogranin B	CHGB
ENSG00000204442	21,3277763	53,54012122	6,733506968	-1,400727356	0,007818262	NALCN channel auxiliary fac	NALF1
ENSG00000118473	27,80573293	62,98824837	6,778834892	-1,392160926	0,018890518	SH3GL interacting endocytic	SGIP1
ENSG00000117602	26,32815298	64,98569428	6,376874178	-1,391625556	0,004862498	RCAN family member 3	RCAN3
ENSG00000275450	0,191857419	0,447451288	-0,440707156	-1,377072855	0,011587625	PTGER4P2-CDK2AP2P2 read	PTGER4P2-CDK2AP2P2
ENSG00000139112	89,22282132	224,9916641	8,912215858	-1,374438694	0,000890311	GABA type A receptor assoc	GABARAPL1
ENSG00000160191	9,183096101	23,75283542	5,255697672	-1,368211264	0,006781451	phosphodiesterase 9A	PDE9A
ENSG00000188906	7,619909035	18,83167535	4,725960647	-1,365948865	0,003008608	leucine rich repeat kinase 2	LRRK2
ENSG00000230487	4,246641379	9,670124291	3,974405792	-1,363982458	0,000205591	PSMG3 antisense RNA 1 (he	PSMG3-AS1
ENSG00000118997	0,715786557	1,844669856	1,98870986	-1,35868082	0,000125073	dynein axonemal heavy chai	DNAH7
ENSG00000136842	80,91875275	124,8325574	6,754330857	-1,358092688	0,000584568	tropomodulin 1	TMOD1
ENSG00000139737	7,311243429	15,0858596	4,454253454	-1,356240964	0,000236284	SLAIN motif family member	SLAIN1
ENSG00000278053	69,25744188	176,4598787	8,077717909	-1,34981702	2,42E-20	DExD-box helicase 52	DDX52
ENSG00000163082	16,92016528	41,81719533	6,140580935	-1,349235328	0,004417928	sphingosine-1-phosphate ph	SGPP2
ENSG00000188517	0,581967979	1,342401531	1,022852695	-1,344156364	0,001070519	collagen type XXV alpha 1 ch	COL25A1
ENSG00000171621	63,58182163	155,9019876	8,026418408	-1,343531576	7,99E-23	splA/ryanodine receptor doı	SPSB1
ENSG00000168779	2,461028979	4,133013509	1,725020407	-1,340682704	3,38E-05	short stature homeobox 2	SHOX2
ENSG00000127955	71,96566162	182,912385	8,197915653	-1,338488716	3,31E-06	G protein subunit alpha i1	GNAI1
ENSG00000147119	33,0850209	83,60020711	6,850913733	-1,333622952	4,44E-12	carbohydrate sulfotransfera	CHST7
ENSG00000155792	2,838868415	7,049542193	4,688158979	-1,331575536	0,011949026	DEP domain containing MTC	DEPTOR
ENSG00000178075	16,36280046	41,6172316	5,737477967	-1,331234791	9,52E-06	GRAM domain containing 1C	GRAMD1C
ENSG00000135503	107,0140243	281,5557669	8,956885408	-1,327834787	0,014163069	activin A receptor type 1B	ACVR1B
ENSG00000128311	26,85950179	66,69709956	6,89951442	-1,324082241	1,15E-16	thiosulfate sulfurtransferase	TST
ENSG00000100292	43,8337374	110,6322891	7,871556237	-1,322466256	4,13E-09	heme oxygenase 1	HMOX1
ENSG00000164300	23,16697593	56,96641814	6,967474612	-1,321552432	1,15E-05	serine incorporator 5	SERINC5
ENSG00000101187	40,41390024	99,51682236	7,096849629	-1,320602858	0,003419809	solute carrier organic anion	SLCO4A1
ENSG00000197261	1,828063698	4,438633281	2,787275552	-1,31482478	0,000109564	chromosome 6 open readin	C6orf141
ENSG00000153714	8,725625715	19,41699485	4,517922116	-1,313835855	1,22E-12	leucine rich adaptor protein	LURAP1L
ENSG00000072133	5,979175812	12,27481472	4,303629831	-1,304544382	0,048868149	ribosomal protein S6 kinase	RPS6KA6
ENSG00000144802	84,92259048	193,8904359	8,327430699	-1,289312153	0,044064235	NFKB inhibitor zeta	NFKBIZ
ENSG00000137875	0,199355268	0,469953218	-0,358957166	-1,289256267	0,024626534	BCL2 like 10	BCL2L10
ENSG00000237149	3,940649382	7,778210661	3,784889101	-1,28879122	6,20E-10	ZNF503 antisense RNA 2	ZNF503-AS2
ENSG00000076826	7,88826306	16,33864111	3,913277877	-1,288700575	0,018594792	calmodulin regulated spectr	CAMSAP3
ENSG00000138771	146,5712838	351,5608615	8,676850266	-1,28521912	6,17E-10	shroom family member 3	SHROOM3
ENSG00000003987	2,982329178	7,217672887	3,706789846	-1,278691251	1,82E-06	myotubularin related protei	MTMR7
ENSG00000170579	0,633286808	1,49936913	0,90705619	-1,277750835	0,017168712	DLG associated protein 1	DLGAP1
ENSG00000136158	49,9783494	112,2798561	7,197201227	-1,276302446	1,18E-08	sprouty RTK signaling antag	SPRY2
ENSG00000182636	23,82853718	40,55019928	6,536070447	-1,271785472	0,028527588	necdin, MAGE family memb	NDN
ENSG00000164683	0,635928478	1,443992758	0,60958427	-1,269113821	0,004237717	hes related family bHLH trar	HEY1
ENSG00000173334	26,90290965	63,10083005	7,086978873	-1,266266212	2,72E-05	tribbles pseudokinase 1	TRIB1
ENSG00000010295	19,5056117	36,72860234	6,664318181	-1,260077696	0,021184022	intermediate filament family	IFFO1
ENSG00000178531	19,2311345	41,54523268	5,702113292	-1,256223922	0,001162015	cortexin 1	CTXN1
ENSG00000273045	0,269499562	0,626505236	-0,118679288	-1,253038482	0,027215437	chromosome 2 open readin	C2orf15
ENSG00000155749	0,19308071	0,458518296	-0,175449976	-1,248450992	0,025889867	flagellum associated contair	FLACC1
ENSG00000157578	0,516380637	1,195989272	1,299069036	-1,247328469	0,003370399	lebercilin LCA5 like	LCA5L
ENSG00000144063	0,449969447	1,050819681	4,69917022	-1,245925062	0,000722886	mal, T cell differentiation pr	MALL
ENSG00000205572	1,207209726	2,858419734	2,612196888	-1,241332609	0,032165885	small EDRK-rich factor 1A	SERF1A
ENSG00000146859	11,40234994	26,48673126	5,608358933	-1,239134346	1,50E-08	transmembrane protein 14C	TMEM140
ENSG00000172733	2,131962604	4,255662888	2,750684876	-1,230941748	0,018078283	purine rich element binding	PURG
ENSG00000102287	25,77942226	40,59436377	6,193175372	-1,230831744	0,021671545	gamma-aminobutyric acid ty	GABRE
ENSG00000116106	20,49651911	37,78037143	6,024340508	-1,22830162	0,010547472	EPH receptor A4	EPHA4
ENSG00000113916	97,02362144	225,1798744	8,922693212	-1,219183133	2,20E-13	BCL6 transcription repressor	BCL6
ENSG00000101849	108,9032829	256,9714018	8,876004279	-1,21255816	7,65E-07	transducin beta like 1 X-link	TBL1X
ENSG00000169242	30,87137172	69,34845652	7,061251516	-1,210148735	0,011549822	ephrin A1	EFNA1
ENSG00000221949	0,341478204	0,78335442	0,762656897	-1,203756846	0,005081149	long intergenic non-protein	LINC01465
ENSG00000161835	4,6897649	10,72346283	4,048513716	-1,203391286	0,000126774	trafficking regulator and sca	TAMALIN
ENSG00000261716	3,027364167	5,941613067	3,19132465	-1,199057998	2,93E-05	H2B clustered histone 20, ps	H2BC20P
ENSG00000172296	20,4528458	46,84040311	6,49976016	-1,196665905	0,000196965	serine palmitoyltransferase	SPTLC3
ENSG00000183186	1,21240609	2,684505896	2,053326618	-1,196002334	0,049035655	C2 calcium dependent doma	C2CD4C
ENSG00000162825	6,446623241	14,7180856	4,610245218	-1,195982391	1,38E-06	NBPF member 20	NBPF20

ENSG00000214967	1,619978517	3,71547613	2,853845199	-1,195708437	0,01010902	nuclear pore complex intera	NPIPA7
ENSG00000183578	2,042733298	4,440687355	3,336842278	-1,186270072	0,000366676	TNF alpha induced protein 8	TNFAIP8L3
ENSG00000160200	22,12667508	48,4674011	6,076766718	-1,180964874	0,004692498	cystathionine beta-synthase	CBS
ENSG00000175175	2,512031002	5,626916701	3,865984352	-1,180036056	0,001029528	protein phosphatase, Mg2+/	PPM1E
ENSG00000167772	37,78877498	75,57653187	7,085419549	-1,179405107	0,011051533	angiopoietin like 4	ANGPTL4
ENSG00000169908	70,39375266	159,9863031	8,018582518	-1,17730638	0,014897592	transmembrane 4 L six famil	TM4SF1
ENSG00000173281	81,21985511	182,3364198	8,517861861	-1,174319147	2,76E-06	protein phosphatase 1 regul	PPP1R3B
ENSG00000165215	2,045173792	3,51380259	2,438700934	-1,17264453	0,001748217	claudin 3	CLDN3
ENSG00000214725	0,247486826	0,548758018	1,008489664	-1,168017692	0,005201889	CDIP transferase opposite st	CDIPTOSP
ENSG00000165655	90,12656848	176,9850645	8,351119798	-1,161014781	2,98E-16	zinc finger protein 503	ZNF503
ENSG00000151012	3,082347477	6,777448521	3,386431587	-1,160799892	1,27E-05	solute carrier family 7 meml	SLC7A11
ENSG00000259663	0,164503001	0,290851604	-0,613906379	-1,158681845	0,04700509	ISL1 divergent transcript	ISL1-DT
ENSG00000204173	0,274952879	0,581679374	0,742649791	-1,158313966	0,03684855	leucine rich repeat containir	LRRC37A5P
ENSG00000163536	2,88384857	5,744394045	3,104432909	-1,155722044	0,011881498	serpin family I member 1	SERPINI1
ENSG00000166532	42,04182881	93,25564766	7,115397229	-1,155601147	5,32E-09	ribosomal modification prot	RIMKLB
ENSG00000140104	8,95435374	19,21582812	4,986723981	-1,152031391	3,31E-10	clathrin binding box of aftipl	CLBA1
ENSG00000134627	3,040125002	6,522395359	3,154322424	-1,148489399	4,72E-06	piwi like RNA-mediated gen	PIWIL4
ENSG00000147642	4,011946843	8,60775052	3,811336881	-1,145634807	0,002252027	syntabulin	SYBU
ENSG00000104312	31,14606289	63,69903864	6,579115388	-1,142935777	0,003381748	receptor interacting serine/t	RIPK2
ENSG00000180730	93,3231497	207,0582834	8,573830273	-1,141306221	0,00123697	shisa family member 2	SHISA2
ENSG00000267322	0,910579558	1,922447242	2,147159422	-1,138658081	0,044500254	small Cajal body-specific RN	SCARNA17
ENSG00000281706	0,657255408	1,3412192	1,823650417	-1,137776992	0,013870353	long intergenic non-protein	LINC01012
ENSG00000147027	72,4607786	152,0125453	8,069371581	-1,137346084	0,019476709	transmembrane protein 47	TMEM47
ENSG00000213213	0,600103393	1,264511312	1,411311279	-1,134514914	0,001617645	coiled-coil domain containin	CCDC183
ENSG00000166446	25,5243281	56,18752545	6,310749656	-1,133586821	8,78E-07	chromodomain Y like 2	CDYL2
ENSG00000166046	5,952988196	11,64854149	4,787786944	-1,133068888	0,001370137	t-complex 11 like 2	TCP11L2
ENSG00000180638	0,391823303	0,863157217	0,800048731	-1,13255946	0,036662279	solute carrier family 47 men	SLC47A2
ENSG00000108582	623,0773961	1233,805555	10,79433702	-1,125795588	0,000158128	carboxypeptidase D	CPD
ENSG00000164093	16,51186578	27,52982609	4,555730079	-1,125239902	0,009283728	paired like homeodomain 2	PITX2
ENSG00000007516	9,654241615	21,04970656	4,745353398	-1,12513836	5,07E-05	BAI1 associated protein 3	BAIAP3
ENSG00000187193	15,19413359	32,99034188	5,892284653	-1,123323655	1,50E-06	metallothionein 1X	MT1X
ENSG00000173295	3,104340703	6,805224658	3,560509582	-1,120709033	0,001938698	family with sequence similar	FAM86B3P
ENSG00000205485	8,597594382	15,4008133	4,265121051	-1,119481821	0,013915042	long intergenic non-protein	LINC03009
ENSG00000135063	0,768127199	1,519037566	1,298733771	-1,118542916	0,037407602	family with sequence similar	FAM189A2
ENSG00000147852	66,30569529	141,3560428	8,294549088	-1,117416456	0,003111833	very low density lipoprotein	VLDLR
ENSG00000105835	164,2644829	358,2409574	9,434790285	-1,114196124	0,000706058	nicotinamide phosphoribosy	NAMPT
ENSG00000172594	4,5661074	9,441925155	4,233537538	-1,106360477	1,72E-08	sphingomyelin phosphodies	SMPDL3A
ENSG00000165757	47,0648079	93,56244302	7,404218485	-1,106330099	6,26E-08	junctional cadherin 5 associ	JCAD
ENSG00000113532	11,64673059	22,3888608	5,041959116	-1,106211972	0,035890201	ST8 alpha-N-acetyl-neurami	ST8SIA4
ENSG00000150907	142,8249151	307,7172689	9,239408924	-1,10574863	0,002445324	forkhead box O1	FOXO1
ENSG00000164823	42,02043709	88,90855864	7,18604536	-1,093578978	0,000647119	oxidative stress induced gro	OSGIN2
ENSG00000165030	39,24536284	83,3766162	7,201929751	-1,090086792	3,09E-07	nuclear factor, interleukin 3	NFIL3
ENSG00000022567	22,83732389	48,6651021	6,375969589	-1,089802018	6,60E-21	solute carrier family 45 men	SLC45A4
ENSG00000178607	15,97807656	33,90323005	6,218614592	-1,084029263	1,63E-06	endoplasmic reticulum to nu	ERN1
ENSG00000120738	186,4711316	401,2086817	9,298143737	-1,07642948	0,003606882	early growth response 1	EGR1
ENSG00000140876	1,904226739	3,99395515	2,914497768	-1,075402871	0,012705464	nudix hydrolase 7	NUDT7
ENSG00000260941	0,314263835	0,660311757	0,699249332	-1,073839093	0,02233594	long intergenic non-protein	LINC00622
ENSG00000187231	65,37579969	134,4477835	7,77242265	-1,070044687	5,20E-05	SEC14 and spectrin domain	SESTD1
ENSG00000129946	4,943700269	8,421611929	4,488737886	-1,069657154	8,29E-05	SHC adaptor protein 2	SHC2
ENSG00000149131	129,0373126	277,6831493	9,576378092	-1,068011746	0,025404727	serpin family G member 1	SERPING1
ENSG00000135919	96,58931475	203,2430828	8,984170573	-1,067217931	4,75E-19	serpin family E member 2	SERPINE2
ENSG00000277147	2,826394414	5,009921892	3,490067692	-1,065066785	4,69E-08	long intergenic non-protein	LINC00869
ENSG00000158458	1,102777901	2,089703934	1,792408809	-1,063270408	0,000140841	neuregulin 2	NRG2
ENSG00000141337	4,611782516	9,481898446	4,325498216	-1,058280136	0,011099404	arylsulfatase G	ARSG
ENSG00000185436	6,177086516	11,41620657	4,275502862	-1,054771379	0,014721637	interferon lambda receptor	IFNLR1
ENSG00000283486	1,216767554	2,304203648	2,316577837	-1,054127397	0,038306449	family with sequence similar	FAM95C
ENSG00000166780	8,559498566	16,89926635	5,3144455	-1,049624046	0,007728096	bMERB domain containing 1	BMERB1
ENSG00000140092	4,991124996	9,925552426	4,17072334	-1,041757221	0,002280752	fibulin 5	FBLN5
ENSG00000184060	4,796518984	8,922388085	4,081631895	-1,039181063	0,006599846	ArfGAP with dual PH domair	ADAP2
ENSG00000121039	49,36100525	97,04099312	7,40082428	-1,037121053	0,000169612	retinol dehydrogenase 10	RDH10
ENSG00000168143	3,365439094	6,921824958	3,080214002	-1,027122689	0,022057387	family with sequence similar	FAM83B
ENSG00000152661	167,8692071	312,0238882	9,063695272	-1,01776524	0,020447604	gap junction protein alpha 1	GJA1
ENSG00000006459	10,33150362	19,31803118	5,296694785	-1,01551757	0,001255543	lysine demethylase 7A	KDM7A
ENSG00000110693	1,632588179	2,27905814	3,071926207	-1,015383063	0,032239086	SRY-box transcription factor	SOX6
ENSG00000147324	95,40398564	192,3590409	8,479217908	-1,011828309	1,50E-05	multifunctional ROCO family	MFHAS1
ENSG00000106025	10,26479957	20,16207475	5,08704786	-1,011448386	0,007229645	tetraspanin 12	TSPAN12
ENSG00000074416	321,3038095	669,9774219	10,21292105	-1,010953365	0,016119935	monoglyceride lipase	MGLL
ENSG00000163739	28,4318226	56,13977514	7,403442065	-1,009803356	0,00114203	C-X-C motif chemokine ligan	CXCL1
ENSG00000116991	92,85434676	182,6876452	8,67079644	-1,00917559	0,002500433	signal induced proliferation	SIPA1L2
ENSG00000196177	9,722084537	19,39907514	5,31808301	-1,005882149	4,27E-08	acyl-CoA dehydrogenase shc	ACADSB
ENSG00000130066	230,7366683	456,5843369	9,595207279	-1,004903546	0,007150806	spermidine/spermine N1-ac	SAT1
ENSG00000166741	461,4969921	930,3247166	10,97799759	-1,000057768	0,000500903	nicotinamide N-methyltrans	NNMT
ENSG00000061455	0,270692548	0,547457271	0,963154046	-0,996421319	0,040650873	PR/SET domain 6	PRDM6
ENSG00000198736	28,79809983	56,97794363	6,830698352	-0,993776526	7,21E-06	methionine sulfoxide reduct	MSRB1
ENSG00000159217	46,57293882	71,01142983	6,228507044	-0,992865682	1,69E-06	insulin like growth factor 2 r	IGF2BP1
ENSG00000137309	485,83638	950,3595479	10,65297796	-0,989444441	1,56E-19	high mobility group AT-hook	HMGA1
ENSG00000147174	1,172066364	2,313477246	2,320207993	-0,988038465	0,000704793	germ cell nuclear acidic pep	GCNA
ENSG00000134294	273,8848843	532,8466442	9,825753742	-0,984262057	9,49E-19	solute carrier family 38 men	SLC38A2
ENSG00000122378	172,1665172	335,5118972	9,080868678	-0,984087276	0,002138746	peroxiredoxin like 2A	PRXL2A
ENSG00000120162	6,28106969	12,35165216	4,476981971	-0,980984025	0,040122483	MOB kinase activator 3B	MOB3B
ENSG00000166387	0,999701561	1,959330421	2,331790476	-0,977971653	0,010489097	PPFIA binding protein 2	PPFIBP2
ENSG00000034677	63,48020926	124,9616161	8,002522492	-0,977572757	5,11E-10	ring finger protein 19A, RBR	RNF19A
ENSG00000184557	50,44708772	98,93441792	7,525366885	-0,977137838	0,002199689	suppressor of cytokine signa	SOCS3
ENSG00000231028	0,242309366	0,481447725	0,295408755	-0,973999231	0,04262239	AHI1 divergent transcript	AHI1-DT
ENSG00000113369	136,5095798	252,3457568	8,825456097	-0,971658207	0,010441777	arrestin domain containing 3	ARRDC3
ENSG00000226029	0,485074059	0,928798097	0,8564566	-0,969746402	0,00295009	long intergenic non-protein	LINC01772
ENSG00000137880	20,06204915	38,22887544	5,959797631	-0,96808617	0,012145955	GTP cyclohydrolase I feedba	GCHFR
ENSG00000204052	0,639726293	1,242478213	1,261638764	-0,960545186	0,033373691	leucine rich repeat containir	LRRC73
ENSG00000182919	9,889040824	18,89096587	5,469852062	-0,957086403	0,006538852	chromosome 11 open readi	C11orf54
ENSG00000178201	0,374874717	0,727235884	0,604696103	-0,953540999	0,028031755	vomeronasal 1 receptor 1	VN1R1
ENSG00000134709	41,82555211	78,07683335	7,015240573	-0,952885192	0,017669112	hook microtubule tethering	HOOK1
ENSG00000187678	58,03506168	111,928135	7,578351519	-0,952839741	2,13E-06	sprouty RTK signaling antag	SPRY4
ENSG00000178568	1,290141677	2,475766246	1,456350817	-0,946912399	0,029845912	erb-b2 receptor tyrosine kin	ERBB4
ENSG00000204767	6,522332756	9,977846588	4,005454885	-0,94640403	0,015655434	inhibitory synaptic factor far	INSYN2B
ENSG00000130751	3,034821572	5,849668933	3,309580656	-0,94588181	0,001412324	neuronal PAS domain protei	NPAS1
ENSG00000135407	3,334022537	6,207470004	3,744953211	-0,945430707	0,003619906	advillin	AVIL
ENSG00000163754	47,42801757	91,2249914	7,283380891	-0,944182871	2,93E-08	glycogenin 1	GYG1
ENSG00000077585	16,47126228	30,55334044	5,926190061	-0,937614872	0,000294439	G protein-coupled receptor	GPR137B
ENSG00000176809	4,123172162	7,749553908	4,068565879	-0,936159524	1,09E-05	leucine rich repeat containir	LRRC37A3
ENSG00000176896	1,16601111	2,146600384	2,185588085	-0,935571622	0,001335542	transcription elongation fact	TCEANC
ENSG00000229644	14,99234507	28,73276415	5,955494279	-0,933474809	0,007573996	nicotinamide phosphoribosy	NAMPTP1
ENSG00000162426	6,601022747	12,55349962	4,393733609	-0,933137811	0,00031183	solute carrier family 45 men	SLC45A1
ENSG00000170293	8,439294448	14,87479812	4,851084849	-0,93032576	3,79E-05	CKLF like MARVEL transmem	CMTM8
ENSG00000113361	487,8677226	391,8392381	9,138435391	-0,929623062	0,037468866	cadherin 6	CDH6
ENSG00000166436	8,129906783	14,95839337	4,990165569	-0,929055031	0,00020676	tripartite motif containing 6	TRIM66
ENSG00000185920	8,669905018	15,70238557	5,123759721	-0,927759568	0,000359322	patched 1	PTCH1
ENSG00000144677	100,5372453	190,1248522	8,405614706	-0,925632598	3,18E-05	CTD small phosphatase like	CTDSPL
ENSG00000184995	0,637001473	1,17448534	1,036810602	-0,924361522	0,010305289	interferon epsilon	IFNE
ENSG00000163347	343,6682273	660,3364374	10,14178389	-0,923945444	1,40E-08	claudin 1	CLDN1
ENSG00000264175	0,856997784	1,599131949	2,093654277	-0,922946916	0,049916355	microRNA 3189	MIR3189
ENSG00000175505	20,93244197	37,71774704	6,164312964	-0,91706183	0,001636783	cardiotrophin like cytokine f	CLCF1
ENSG00000145390	36,83881223	69,04093649	7,089113572	-0,913881465	1,83E-05	ubiquitin specific peptidase	USP53
ENSG00000135333	1,354697854	2,382574615	2,458859617	-0,911061576	0,015780855	EPH receptor A7	EPHA7
ENSG00000135083	18,29440175	33,6453307	6,179366347	-0,910910181	0,005992847	cyclin J like	CCNJL
ENSG00000164463	18,1383283	33,92723196	6,301458692	-0,910388397	4,72E-13	CREB3 regulatory factor	CREBRF

ENSG00000181019	89,72981541	170,815227	8,533722948	-0,907778846	8,43E-08	NAD(P)H quinone dehydrog	NQO1
ENSG00000239467	1,78685811	3,312804543	2,657740065	-0,905625158	0,008686213	glutamate rich 2	ERICH2
ENSG00000235257	2,510937703	4,607914633	3,046769593	-0,903079027	0,011097142	ITGA9 antisense RNA 1	ITGA9-AS1
ENSG00000153208	10,34148211	18,14854066	5,173112394	-0,901481536	0,005696942	MER proto-oncogene, tyrosi	MERTK
ENSG00000067082	227,6157953	426,0410668	9,635395611	-0,899564205	5,92E-12	Kruppel like factor 6	KLF6
ENSG00000188596	3,778239381	7,066876888	3,585960504	-0,898180341	6,98E-09	cilia and flagella associated	CFAP54
ENSG00000107679	97,68564293	174,6620997	8,282322675	-0,893894982	0,000268122	pleckstrin homology domair	PLEKHA1
ENSG00000153094	68,88422072	121,8896126	7,571073161	-0,892915631	0,003002426	BCL2 like 11	BCL2L11
ENSG00000141448	8,209241017	13,11177368	5,053126498	-0,890929069	0,001283264	GATA binding protein 6	GATA6
ENSG00000128016	36,08259696	67,08805945	7,025092149	-0,886202787	7,98E-09	ZFP36 ring finger protein	ZFP36
ENSG00000123191	10,10352731	18,142969	5,244316975	-0,885903171	0,000589759	ATPase copper transporting	ATP7B
ENSG00000137819	11,79306017	21,14540058	5,660092301	-0,885087541	0,003970606	progestin and adipoQ recep	PAQR5
ENSG00000125968	17,58857787	32,75023253	5,410667084	-0,88419579	3,97E-06	inhibitor of DNA binding 1, H	ID1
ENSG00000135318	385,3290904	677,2201856	10,11905852	-0,879794183	9,06E-07	5'-nucleotidase ecto	NT5E
ENSG00000198064	0,991032806	1,856432866	1,895510632	-0,878048078	0,015208613	nuclear pore complex intera	NPIPB13
ENSG00000133740	4,440403815	7,949007291	4,07669842	-0,877830278	1,23E-05	E2F transcription factor 5	E2F5
ENSG00000033327	46,99349961	87,22946973	7,524367998	-0,877203982	1,29E-05	GRB2 associated binding prc	GAB2
ENSG00000239521	17,54823848	30,85928202	6,094954378	-0,873392656	0,018659607	CASTOR family member 3	CASTOR3
ENSG00000222112	0,733570841	1,363145048	1,527276258	-0,869634612	0,036903846	RN7SK pseudogene 16	RN7SKP16
ENSG00000157399	9,263733589	16,33787586	4,531099882	-0,869586549	0,022298794	arylsulfatase L	ARSL
ENSG00000119950	52,93864051	90,31998335	7,603428556	-0,867286323	0,001300111	MAX interactor 1, dimerizati	MXI1
ENSG00000272899	1,126415481	2,013471213	2,231427178	-0,86351898	0,001090087	ATP6V1F neighbor	ATP6V1FNB
ENSG00000198873	5,44526076	8,917313894	4,176823286	-0,862844645	0,005480968	G protein-coupled receptor	GRK5
ENSG00000111275	33,16986841	59,50155862	6,650023323	-0,862117108	0,000264544	aldehyde dehydrogenase 2 f	ALDH2
ENSG00000186716	121,6279537	215,9856378	8,694829313	-0,860921376	1,84E-06	BCR activator of RhoGEF and	BCR
ENSG00000167646	1,580931076	2,829544403	2,43822008	-0,859315765	0,005078631	dynein axonemal assembly f	DNAAF3
ENSG00000105514	69,91737308	127,2275994	7,88676841	-0,857481545	0,000441565	RAB3D, member RAS oncog	RAB3D
ENSG00000114948	1,411518943	2,393231623	1,539170064	-0,855572736	0,008889951	ADAM metallopeptidase dor	ADAM23
ENSG00000205639	0,773770838	1,387530142	1,587929268	-0,852420827	0,010217179	major facilitator superfamily	MFSD2B
ENSG00000258727	7,338987674	12,606275	5,040988462	-0,850683732	0,028446005	AP1G2 antisense RNA 1	AP1G2-AS1
ENSG00000177000	47,60830213	85,49436835	7,509767983	-0,848507937	4,75E-12	methylenetetrahydrofolate	MTHFR
ENSG00000100422	65,25590338	112,7302682	7,841166213	-0,847681834	0,000171063	ceramide kinase	CERK
ENSG00000075651	31,64413187	54,77579069	6,661619557	-0,845079984	0,000133059	phospholipase D1	PLD1
ENSG00000128713	14,96321361	22,60439348	5,845520978	-0,844348669	0,032573025	homeobox D11	HOXD11
ENSG00000104447	31,62243046	56,75808855	6,955814593	-0,843402093	2,34E-06	transcriptional repressor GA	TRPS1
ENSG00000151611	11,33930638	19,92258523	5,4077529	-0,842986218	0,000441084	metabolism of cobalamin as	MMAA
ENSG00000236901	2,677527332	4,711555858	3,259988355	-0,842736804	0,001799607	MIR600 host gene	MIR600HG
ENSG00000253276	34,79457886	62,08002413	7,156363206	-0,839157469	6,08E-25	coiled-coil domain containin	CCDC71L
ENSG00000173530	68,70114447	119,3095192	7,955232878	-0,837883685	0,000403938	TNF receptor superfamily m	TNFRSF10D
ENSG00000105855	184,3373511	312,3879927	9,303132595	-0,835445025	1,43E-05	integrin subunit beta 8	ITGB8
ENSG00000105829	29,38186544	52,08313611	6,713646564	-0,835177979	8,48E-13	Bet1 golgi vesicular membra	BET1
ENSG00000165169	41,21692579	72,90632647	7,094961665	-0,83514207	1,56E-10	dynein light chain Tctex-typ	DYNLT3
ENSG00000138678	5,154905123	9,226936852	4,088235919	-0,83485759	0,006852467	glycerol-3-phosphate acyltra	GPAT3
ENSG00000265778	0,699564258	1,241908466	1,651728659	-0,834716243	0,006866526	ZNF516 antisense RNA 1	ZNF516-AS1
ENSG00000152409	38,01546161	67,28799542	7,320586242	-0,834707882	1,34E-13	junction mediating and regu	JMY
ENSG00000158985	46,22813341	82,40089749	7,214988803	-0,834575761	1,55E-05	CDC42 small effector 2	CDC42SE2
ENSG00000114541	14,38068781	24,04771561	5,425102647	-0,830931888	0,034600222	FERM domain containing 4B	FRMD4B
ENSG00000198517	30,23148153	52,69522338	6,71435928	-0,830317996	3,03E-08	MAF bZIP transcription facto	MAFK
ENSG00000072274	61,84686138	106,6889777	8,135733834	-0,829114646	0,001188783	transferrin receptor	TFRC
ENSG00000137414	40,89612461	70,95110115	7,303993013	-0,828175056	2,33E-05	family with sequence simila	FAM8A1
ENSG00000100994	291,4229184	519,0178822	10,06899706	-0,825201639	1,31E-21	glycogen phosphorylase B	PYGB
ENSG00000235508	3,844292494	6,232375671	3,741825017	-0,824212417	0,049819075	ribosomal protein S2 pseudo	RPS2P7
ENSG00000260231	1,450811123	2,483275848	2,5922924	-0,822933427	0,002730086	KDM7A divergent transcript	KDM7A-DT
ENSG00000215769	2,075828351	3,668252866	3,244862032	-0,821887926	0,011840033	ARHGAP27P1-BPTFP1-KPNA	ARHGAP27P1-BPTFP1-KPNA2P3
ENSG00000153551	35,95510641	57,56981375	6,570056245	-0,820728888	8,80E-06	CKLF like MARVEL transmem	CMTM7
ENSG00000214274	1,416873551	2,362632845	2,337092735	-0,820333068	0,010040436	angiogenin	ANG
ENSG00000141934	19,33278654	31,15214787	5,35884479	-0,818892013	0,006355136	phospholipid phosphatase 2	PLPP2
ENSG00000111912	38,07559755	67,42406409	7,009683128	-0,818054848	1,33E-07	nuclear receptor coactivato	NCOA7
ENSG00000113448	17,93196716	31,45691444	6,080752095	-0,816525965	0,010061991	phosphodiesterase 4D	PDE4D
ENSG00000175832	37,52081688	65,68472447	6,829450657	-0,816215382	0,00451996	ETS variant transcription fac	ETV4
ENSG00000205189	37,5224786	65,74751611	7,016963594	-0,815607043	0,001096728	zinc finger and BTB domain	ZBTB10
ENSG00000088727	1,637474879	2,885942606	2,624400527	-0,814513426	5,27E-06	kinesin family member 9	KIF9
ENSG00000118432	29,0185225	51,48585032	6,676827901	-0,812514395	0,000264544	cannabinoid receptor 1	CNR1
ENSG00000168032	1,337901585	2,145554719	2,404899247	-0,812440408	0,00668411	ectonucleoside triphosphate	ENTPD3
ENSG00000163297	72,85947126	126,9147856	7,761835712	-0,811902664	0,00396563	ANTXR cell adhesion molecu	ANTXR2
ENSG00000188766	22,92492548	38,36690056	6,193595249	-0,809855576	7,33E-06	sprouty related EVH1 domai	SPRED3
ENSG00000115274	0,800587198	1,386853144	1,858117548	-0,808248462	0,021952931	INO80 complex subunit B	INO80B
ENSG00000273679	0,473531137	0,828465531	0,778897453	-0,808132129	0,048530149	RING finger protein 145-like	LOC441711
ENSG00000137193	24,83324587	40,99548687	6,340399965	-0,806529914	0,001970574	Pim-1 proto-oncogene, serir	PIM1
ENSG00000183696	35,79642762	62,43759094	6,873993558	-0,804391263	6,55E-07	uridine phosphorylase 1	UPP1
ENSG00000108219	412,2468532	712,3962525	10,34822406	-0,802478905	1,47E-07	tetraspanin 14	TSPAN14
ENSG00000072210	59,52891231	104,2194928	7,796641707	-0,802461923	5,46E-08	aldehyde dehydrogenase 3 f	ALDH3A2
ENSG00000128272	143,0548839	249,174203	9,149655008	-0,801752779	1,59E-12	activating transcription facto	ATF4
ENSG00000119669	181,2118609	312,9847553	9,298481768	-0,801338113	5,50E-05	interferon regulatory factor	IRF2BPL
ENSG00000118985	83,80683957	145,9776069	7,973358754	-0,800088204	1,10E-07	elongation factor for RNA po	ELL2
ENSG00000052795	72,78978775	125,0808783	7,898726475	-0,797699364	0,001546907	folliculin interacting protein	FNIP2
ENSG00000224189	60,86795226	108,3531723	7,436762053	-0,796952232	0,038200757	HOXD antisense growth-ass	HAGLR
ENSG00000187098	30,87040004	53,81539814	6,604419029	-0,795474944	0,008089175	melanocyte inducing transcr	MITF
ENSG00000236992	0,363137889	0,626505236	0,811137375	-0,794503218	0,046072419	ribosomal protein L12 pseud	RPL12P12
ENSG00000106089	12,77645764	22,05515631	5,542615738	-0,792561522	0,001573141	syntaxin 1A	STX1A
ENSG00000077238	98,529249	170,3722263	8,289190523	-0,791719664	1,50E-06	interleukin 4 receptor	IL4R
ENSG00000258441	3,896510995	6,675745967	3,920757499	-0,791705405	0,000578408	long intergenic non-protein	LINC00641
ENSG00000215375	4,86349994	7,92550404	4,316329324	-0,78933114	0,000963614	myosin light chain 5	MYL5
ENSG00000100304	91,81803824	155,4843411	8,171211224	-0,78749526	8,57E-23	tubulin tyrosine ligase like 1	TTLL12
ENSG00000170345	9,295075204	16,21189014	4,883821611	-0,785768058	0,04916982	Fos proto-oncogene, AP-1 tr	FOS
ENSG00000109906	30,34072632	52,39634405	7,028840496	-0,783242002	0,000801742	zinc finger and BTB domain	ZBTB16
ENSG00000179988	2,870741694	4,945350511	3,347307074	-0,783191743	5,18E-08	phosphoseryl-tRNA kinase	PSTK
ENSG00000116285	164,0566126	284,5966353	8,959748402	-0,78179709	0,006443036	ERBB receptor feedback inhi	ERRFI1
ENSG00000120051	0,567513703	0,950712028	0,949952985	-0,780242177	0,046221688	cilia and flagella associated	CFAP58
ENSG00000147676	33,51963094	55,37324589	6,874997742	-0,774255725	0,016823576	mal, T cell differentiation pr	MAL2
ENSG00000139146	57,62974025	97,23163113	7,588735309	-0,772994584	1,22E-10	SIN3-HDAC complex associa	SINHCAF
ENSG00000130363	42,53848609	71,99699749	7,196981294	-0,770080645	4,30E-09	radial spoke head 3	RSPH3
ENSG00000143178	1,527916251	2,528238376	2,550596229	-0,769308296	0,002449761	T-box transcription factor 19	TBX19
ENSG00000131016	331,5258315	523,5626841	10,10274689	-0,766767129	8,32E-06	A-kinase anchoring protein 1	AKAP12
ENSG00000186205	6,423835445	10,92136443	4,659969297	-0,764772669	0,000334264	mitochondrial amidoxime re	MTARC1
ENSG00000169239	9,366587656	14,92163974	4,880531431	-0,764181573	0,000455999	carbonic anhydrase 5B	CA5B
ENSG00000167874	0,660665228	1,096019039	1,832631132	-0,763334443	0,011030516	transmembrane protein 88	TMEM88
ENSG00000137767	18,51379774	31,40889504	5,915482438	-0,762948392	0,001131389	sulfide quinone oxidoreduct	SQOR
ENSG00000152580	0,93702523	1,578529927	1,969315103	-0,762896778	0,039390247	immunoglobulin superfamily	IGSF10
ENSG00000274070	6,623783886	10,97746955	4,680328168	-0,761964288	0,001626255	cytosolic arginine sensor for	CASTOR2
ENSG00000260260	1,507596219	2,516726371	2,612930118	-0,761451708	0,006425873	small nucleolar RNA host ge	SNHG19
ENSG00000146373	27,61833575	45,88882194	6,54662984	-0,760453703	1,87E-07	ring finger protein 217	RNF217
ENSG00000138061	266,9165408	444,7192444	9,774601218	-0,760376905	0,041821274	cytochrome P450 family 1 su	CYP1B1
ENSG00000064225	5,989396138	9,716440062	4,023424921	-0,760120532	0,010197374	ST3 beta-galactoside alpha-2	ST3GAL6
ENSG00000163874	21,33605675	35,91558162	6,498305309	-0,759992037	0,040907876	zinc finger CCCH-type contai	ZC3H12A
ENSG00000214944	97,15027795	164,6250773	8,469698813	-0,759709262	0,000995966	Rho guanine nucleotide excl	ARHGEF28
ENSG00000133134	8,882936582	14,78922968	5,497508447	-0,756398362	4,30E-05	brain expressed X-linked 2	BEX2
ENSG00000127415	42,21671293	71,01588879	7,604876495	-0,754988244	2,29E-05	alpha-L-iduronidase	IDUA
ENSG00000270362	2,14930283	3,569024044	3,204159234	-0,754258379	0,00533892	HMGN3 antisense RNA 1	HMGN3-AS1
ENSG00000198743	161,3611461	266,610297	8,965735263	-0,753171674	0,001208946	solute carrier family 5 meml	SLC5A3
ENSG00000234975	13,83863617	23,40735199	5,981006001	-0,752622169	0,001637756	ferritin heavy chain 1 pseud	FTH1P2
ENSG00000027075	4,58421827	7,080415057	4,02627321	-0,752284253	0,003130467	protein kinase C eta	PRKCH
ENSG00000134686	341,4324243	576,2481625	10,26842762	-0,751939641	2,98E-11	polyhomeotic homolog 2	PHC2
ENSG00000001461	69,94104832	114,1355983	8,003739932	-0,751503609	0,000134853	NIPA like domain containing	NIPAL3
ENSG00000085276	72,11835894	123,414598	8,226443212	-0,75080837	0,01686744	MDS1 and EVI1 complex loc	MECOM

ENSG00000157796	34,2945428	57,90186348	7,031103167	-0,750732301	7,51E-15	WD repeat domain 19	WDR19
ENSG00000187672	2,709441674	4,09481613	3,67383383	-0,750085133	0,013721759	ELKS/RAB6-interacting/CAST	ERC2
ENSG00000142599	200,2402744	329,584673	9,226769942	-0,749719235	2,05E-05	arginine-glutamic acid dipep	RERE
ENSG00000181004	6,867128782	11,54555267	4,865789872	-0,748088397	1,10E-06	Bardet-Biedl syndrome 12	BBS12
ENSG00000160172	1,965240729	3,28720212	2,956078247	-0,747141967	0,00234607	family with sequence similar	FAM86C2P
ENSG00000196975	218,7748616	358,6964394	9,328689071	-0,746767373	0,01686744	annexin A4	ANXA4
ENSG00000259826	0,698984999	1,151810992	1,456953607	-0,743072199	0,037347047	CDK13 divergent transcript	CDK13-DT
ENSG00000077063	39,15322817	57,98827609	6,536417775	-0,74259573	0,017273126	cortactin binding protein 2	CTTNBP2
ENSG00000173801	310,0731145	508,636156	10,17162043	-0,742459986	1,08E-06	junction plakoglobin	JUP
ENSG00000065809	68,90234175	111,0430681	7,557088519	-0,742152956	0,006380931	family with sequence similar	FAM107B
ENSG00000175048	21,49427924	36,04642719	6,258964953	-0,741712876	1,01E-06	zinc finger DHHC-type palmi	ZDHHC14
ENSG00000181690	15,11376565	24,56350536	5,619087746	-0,741463746	0,004522199	PLAG1 zinc finger	PLAG1
ENSG00000226328	5,056361493	8,44761651	4,218518504	-0,740994055	2,90E-05	NUP50 divergent transcript	NUP50-DT
ENSG00000068650	127,0441545	209,7845936	8,641324202	-0,739791004	0,001591103	ATPase phospholipid transpo	ATP11A
ENSG00000167100	31,04656911	39,68947184	5,964597309	-0,739295026	0,021052883	sterile alpha motif domain c	SAMD14
ENSG00000130340	91,21841106	151,6008293	8,152553778	-0,736540581	7,75E-09	sorting nexin 9	SNX9
ENSG00000164654	26,25149746	43,95112883	6,55932496	-0,735795997	1,12E-05	meiosis regulator for oocyte	MIOS
ENSG00000148225	12,53982967	20,81638886	5,279981408	-0,734649689	3,54E-05	WD repeat domain 31	WDR31
ENSG00000186815	173,8509636	283,138503	9,584686147	-0,730920214	0,005711322	two pore segment channel 1	TPCN1
ENSG00000185716	20,01423797	33,2257065	5,951163913	-0,729995531	1,66E-05	modulator of smoothened	MOSMO
ENSG00000107372	216,5120747	357,8471326	9,624225545	-0,729955844	0,00152721	zinc finger AN1-type contain	ZFAND5
ENSG00000274471	3,859840992	6,448772001	3,41829301	-0,728880916	0,035039625	HERC2 pseudogene 7	HERC2P7
ENSG00000148090	16,52390731	27,33583156	5,981221087	-0,727672216	2,57E-05	AU RNA binding methylgluta	AUH
ENSG00000186812	16,74175486	27,27771295	6,110698955	-0,725943044	4,63E-05	zinc finger protein 397	ZNF397
ENSG00000123595	22,30265119	35,93540818	6,137173775	-0,725655888	0,00043578	RAB9A, member RAS oncoge	RAB9A
ENSG00000111058	30,68533087	51,65781859	6,952230995	-0,724909868	0,036206411	acyl-CoA synthetase short cl	ACSS3
ENSG00000171962	1,130774037	1,878779878	2,140718567	-0,722079761	0,001584606	dynein regulatory complex s	DRC3
ENSG00000006756	94,54025525	144,7706391	8,153837372	-0,721371969	0,007962841	arylsulfatase D	ARSD
ENSG00000196782	8,302823923	13,42142602	4,854051663	-0,721141894	0,025430034	mastermind like transcriptio	MAML3
ENSG00000143801	12,45691976	20,47950752	5,522553648	-0,720899149	7,73E-06	presenilin 2	PSEN2
ENSG00000048342	17,73536864	27,74420259	5,839628926	-0,720056355	0,000786313	coiled-coil and C2 domain co	CC2D2A
ENSG00000157429	1,570954347	2,58462391	2,88237231	-0,718537932	0,000320868	zinc finger protein 19	ZNF19
ENSG00000174804	14,73308342	23,97904498	5,536330864	-0,718028136	1,80E-09	frizzled class receptor 4	FZD4
ENSG00000186952	0,945075534	1,498525297	1,742328105	-0,717745135	0,020562036	transmembrane protein 232	TMEM232
ENSG00000164010	10,16114872	16,65706579	5,383316077	-0,717584084	2,19E-06	erythroblast membrane asso	ERMAP
ENSG00000139318	104,8722155	172,1953834	8,429944479	-0,717561768	0,004610561	dual specificity phosphatase	DUSP6
ENSG00000184349	88,6127371	144,0670663	8,104830503	-0,717134174	0,013404194	ephrin A5	EFNA5
ENSG00000256806	2,386439994	3,54850544	3,170192767	-0,71589068	0,029899333	chromosome 17 open readin	C17orf100
ENSG00000174567	5,667963768	9,241025453	3,842182062	-0,71480966	0,012659061	golgi transport 1A	GOLT1A
ENSG00000135069	54,69542552	89,70596186	7,438199549	-0,710760118	0,000438137	phosphoserine aminotransfe	PSAT1
ENSG00000103150	15,77210161	25,41271214	5,836927594	-0,710025196	9,92E-07	malonyl-CoA decarboxylase	MLYCD
ENSG00000112837	48,0590719	73,83269589	7,11841242	-0,709827819	0,001757994	T-box transcription factor 18	TBX18
ENSG00000168890	15,31051866	24,23911577	6,087642509	-0,709670571	6,06E-05	transmembrane protein 150	TMEM150A
ENSG00000151491	141,5482412	231,5951391	8,953286403	-0,709416162	3,51E-28	epidermal growth factor rec	EPS8
ENSG00000165355	14,88102297	24,31655086	5,727962216	-0,70924384	1,47E-15	F-box protein 33	FBXO33
ENSG00000109762	28,16296597	45,75627675	6,573281964	-0,708674097	7,46E-15	sorting nexin 25	SNX25
ENSG00000167470	152,6644436	248,3343553	9,116986834	-0,708667431	1,06E-06	midnolin	MIDN
ENSG00000127328	25,09280764	41,02568433	6,19857397	-0,708629801	7,27E-11	RAB3A interacting protein	RAB3IP
ENSG00000162817	18,3809724	29,5444383	5,935934513	-0,707701746	0,00204966	chromosome 1 open reading	C1orf115
ENSG00000116761	2,581412699	4,226847141	3,450372042	-0,705385193	0,000724196	cystathionine gamma-lyase	CTH
ENSG00000084764	6,068108815	9,087327781	4,691615717	-0,703939497	0,002062061	microtubule associated prot	MAPRE3
ENSG00000129657	764,4565902	1245,362034	11,2106201	-0,699655955	0,002090875	SEC14 like lipid binding 1	SEC14L1
ENSG00000060971	40,9934727	66,34317028	7,417538555	-0,699639166	3,80E-09	acetyl-CoA acyltransferase 1	ACAA1
ENSG00000135750	14,20538019	22,40328263	5,139545865	-0,696990668	0,008449685	potassium two pore domain	KCNK1
ENSG00000171223	158,5468775	253,6981862	9,053457445	-0,695696857	0,010659937	JunB proto-oncogene, AP-1	JUNB
ENSG00000225470	31,25777944	48,99110612	6,645478932	-0,694963917	1,74E-09	JPX transcript, XIST activator	JPX
ENSG00000146409	12,72172882	20,81677132	5,596073271	-0,694948136	0,002650402	solute carrier family 18 mem	SLC18B1
ENSG00000128309	73,54151826	118,6058802	8,068930601	-0,693924522	5,97E-06	mercaptopyruvate sulfurtra	MPST
ENSG00000160999	25,4168561	41,09968652	6,506433034	-0,692598436	1,96E-05	SH2B adaptor protein 2	SH2B2
ENSG00000086712	39,22359536	61,31726872	6,779823553	-0,691443937	3,97E-09	taxilin gamma	TXLNG
ENSG00000123684	96,05092221	154,5792749	8,201105523	-0,690943424	7,88E-14	lysophosphatidylglycerol acy	LPGAT1
ENSG00000129566	98,35470788	158,3463774	8,401668745	-0,688854622	2,20E-10	telomerase associated prote	TEP1
ENSG00000273344	5,793581716	8,938099497	3,926879725	-0,688570147	0,034662508	PAXIP1 divergent transcript	PAXIP1-DT
ENSG00000214940	3,385534513	5,414775315	3,350318092	-0,68601524	0,021603931	nuclear pore complex intera	NPIPA8
ENSG00000001084	23,82611321	38,33022021	6,470443183	-0,684201373	3,52E-10	glutamate-cysteine ligase ca	GCLC
ENSG00000164849	1,74973064	2,732990833	3,23059322	-0,68126309	0,030079097	G protein-coupled receptor	GPR146
ENSG00000113384	111,9727812	179,4941367	8,557312519	-0,678598897	1,92E-08	golgi phosphoprotein 3	GOLPH3
ENSG00000221869	186,2058723	299,8999362	9,540363105	-0,678546348	0,009502255	CCAAT enhancer binding pro	CEBPD
ENSG00000139910	11,56820619	18,2038042	5,341808004	-0,676382334	0,000352653	NOVA alternative splicing re	NOVA1
ENSG00000188747	12,81524234	20,41862435	5,474493774	-0,675560185	0,013074973	NADPH oxidase activator 1	NOXA1
ENSG00000046647	15,04367947	22,91980266	5,651207838	-0,675552619	3,80E-08	gem nuclear organelle assoc	GEMIN8
ENSG00000120129	145,0357044	231,1871505	9,17223037	-0,673459952	3,13E-06	dual specificity phosphatase	DUSP1
ENSG00000114861	65,82937437	103,4490046	7,805637786	-0,672269027	0,000424375	forkhead box P1	FOXP1
ENSG00000124275	37,07710843	59,30768529	6,906857154	-0,671041902	4,38E-06	5-methyltetrahydrofolate-ho	MTRR
ENSG00000146278	99,92781521	157,6789766	8,581531056	-0,671039582	4,86E-10	proline rich nuclear recepto	PNRC1
ENSG00000135953	18,27838271	28,95370949	6,046485032	-0,670361504	1,20E-05	major facilitator superfamily	MFSD9
ENSG00000046651	22,34930892	33,48282691	5,995671826	-0,669705873	0,00038284	OFD1 centriole and centriola	OFD1
ENSG00000170412	123,5088571	194,5058535	8,989394111	-0,66632921	0,006175451	G protein-coupled receptor	GPRC5C
ENSG00000114841	7,717421443	12,21987369	5,057025889	-0,665730307	0,000149419	dynein axonemal heavy chai	DNAH1
ENSG00000072778	431,199712	682,6019418	10,68272287	-0,662808498	0,000713401	acyl-CoA dehydrogenase ver	ACADVL
ENSG00000174080	118,1190251	183,0486116	8,6613684	-0,662090077	0,005657436	cathepsin F	CTSF
ENSG00000169715	118,0462067	185,7482747	8,705541836	-0,661240232	1,83E-09	metallothionein 1E	MT1E
ENSG00000114573	115,8053092	183,7739914	8,51061257	-0,661123112	5,95E-17	ATPase H+ transporting V1 s	ATP6V1A
ENSG00000167315	37,32627046	57,34553759	7,028012617	-0,660643477	0,035256403	acetyl-CoA acyltransferase 2	ACAA2
ENSG00000145439	17,32160275	26,70318414	5,851313224	-0,659860784	0,001211054	carbonyl reductase 4	CBR4
ENSG00000153395	336,0988514	534,5579781	10,15276318	-0,657381754	0,001250281	lysophosphatidylcholine acy	LPCAT1
ENSG00000067167	361,8683333	543,390542	9,983831363	-0,656832847	0,001923579	translocation associated me	TRAM1
ENSG00000166145	154,4076856	228,9741417	8,899774823	-0,656618144	0,004671442	serine peptidase inhibitor, K	SPINT1
ENSG00000213625	136,4947273	217,8361211	9,124160813	-0,65622499	0,007728096	leptin receptor overlapping	LEPROT
ENSG00000145860	95,27691145	146,4594209	8,238578655	-0,655490302	2,53E-07	ring finger protein 145	RNF145
ENSG00000166275	5,335859516	8,138088104	4,225977914	-0,653410656	0,002340014	BLOC-1 related complex sub	BORCS7
ENSG00000100284	36,80083	56,80024697	7,13269668	-0,652959266	3,79E-05	target of myb1 membrane t	TOM1
ENSG00000163900	15,77425105	24,5010588	5,793956377	-0,650921388	5,98E-05	transmembrane protein 41A	TMEM41A
ENSG00000130962	45,85671555	70,1269487	7,349487416	-0,650437817	2,56E-08	proline rich and Gla domain	PRRG1
ENSG00000174306	172,7089811	271,5325633	9,387684783	-0,65024244	8,81E-07	zinc fingers and homeoboxe	ZHX3
ENSG00000196911	6,389999091	9,623443929	4,371304674	-0,649306833	3,77E-06	karyopherin subunit alpha 5	KPNA5
ENSG00000162413	62,03798275	97,4419745	7,794923929	-0,648572564	7,03E-06	kelch like family member 21	KLHL21
ENSG00000139178	95,13757179	145,1809628	8,47975954	-0,647156036	0,000446519	complement C1r subcompo	C1RL
ENSG00000143882	0,927271563	1,453581021	1,668483837	-0,644581398	0,024984641	ATPase H+ transporting V1 s	ATP6V1C2
ENSG00000134070	22,64683048	35,03249812	6,177814753	-0,644400558	0,004951845	interleukin 1 receptor assoc	IRAK2
ENSG00000184304	18,47223479	28,83902026	5,947817824	-0,644400058	0,005476435	protein kinase D1	PRKD1
ENSG00000261087	3,086555413	4,809369594	3,552062791	-0,641954776	0,000690597	ZNF706 neighboring transcri	ZNNT1
ENSG00000196372	16,94727144	26,45898125	6,036476835	-0,639985496	3,00E-07	ankyrin repeat and SOCS bo	ASB13
ENSG00000184481	13,60457553	20,89255717	5,889141405	-0,639785538	3,43E-05	forkhead box O4	FOXO4
ENSG00000108352	7,715880272	11,68738011	4,410653115	-0,63791164	0,002492526	Rap guanine nucleotide exch	RAPGEFL1
ENSG00000104635	170,0872959	264,8329893	9,389915324	-0,637578237	0,004195269	solute carrier family 39 mem	SLC39A14
ENSG00000140961	2,432748938	3,802034449	3,658279125	-0,637406757	0,032044517	oxidative stress induced gro	OSGIN1
ENSG00000223891	2,958941839	4,610154546	3,747166535	-0,637184233	0,000837432	OSER1 divergent transcript	OSER1-DT
ENSG00000149218	113,0928704	170,9058692	8,521780214	-0,637012919	0,00038256	endonuclease domain conta	ENDOD1
ENSG00000114626	39,30413357	61,09904339	7,358272866	-0,636226337	1,36E-07	ankyrin repeat and BTB dom	ABTB1
ENSG00000164056	17,19521084	25,91361603	5,513289404	-0,634975433	0,034486448	sprouty RTK signaling antago	SPRY1
ENSG00000105792	1,573415311	2,426800561	2,934275612	-0,63422698	0,005067234	cilia and flagella associated	CFAP69
ENSG00000172780	7,452124299	11,47704813	4,652586471	-0,634139183	0,00013576	RAB43, member RAS oncoge	RAB43
ENSG00000164647	8,104869544	11,39103726	5,175850032	-0,630856454	0,031799677	STEAP family member 1	STEAP1
ENSG00000177426	74,72728095	115,8667997	7,932844894	-0,630142414	4,23E-11	TGFB induced factor homeo	TGIF1

ENSG00000121350	8,42113349	12,91555748	4,610335296	-0,62899256	0,006226238	pyridine nucleotide-disulphi	PYROXD1
ENSG00000153982	1,98333723	2,93254438	3,129956251	-0,628032898	0,008321651	glycerophosphodiester phos	GDPD1
ENSG00000112137	12,766628	19,73226244	5,188667478	-0,627688659	5,94E-05	phosphatase and actin regul	PHACTR1
ENSG00000008513	103,5524125	154,9926667	7,929622449	-0,624341244	0,000552781	ST3 beta-galactoside alpha-2	ST3GAL1
ENSG00000109814	70,04325743	106,7469169	7,696128356	-0,62316205	7,67E-05	UDP-glucose 6-dehydrogena	UGDH
ENSG00000178093	3,098420594	4,723098031	3,489281049	-0,621977225	2,86E-05	testis specific serine kinase 6	TSSK6
ENSG00000203880	25,96360032	39,74191355	6,842493481	-0,62193892	1,29E-06	protein-L-isoaspartate (D-as	PCMTD2
ENSG00000113732	69,01157253	105,7055732	7,991385033	-0,621733454	3,82E-13	ATPase H+ transporting V0 s	ATP6V0E1
ENSG00000106415	8,324885745	12,66229061	4,974935493	-0,620870126	0,009372977	glucocorticoid induced 1	GLCCI1
ENSG00000204977	19,40800827	28,96991664	6,084571686	-0,620537529	0,000964624	tripartite motif containing 1	TRIM13
ENSG00000228288	3,163977348	4,478741703	3,895165123	-0,620489901	0,003467407	prostate cancer associated t	PCAT6
ENSG00000100441	107,3398102	163,2932702	8,798347685	-0,618926683	1,16E-06	KH and NYN domain contain	KHNYN
ENSG00000122729	220,0780098	339,0022812	9,859834295	-0,618511002	1,27E-09	aconitase 1	ACO1
ENSG00000236778	1,815024148	2,807471477	2,994026215	-0,618097466	0,049143498	INTS6 antisense RNA 1	INTS6-AS1
ENSG00000158528	26,98383746	40,61845586	6,474769608	-0,615789767	0,000134946	protein phosphatase 1 regul	PPP1R9A
ENSG00000184007	204,3864372	313,8166305	9,407308206	-0,615464563	8,89E-18	protein tyrosine phosphatas	PTP4A2
ENSG00000185261	2,505476119	3,802592279	3,3358976	-0,615349367	0,032057444	KIAA0825	KIAA0825
ENSG00000182700	9,483116075	14,17004742	5,219705324	-0,614896773	0,009090825	IgA inducing protein	IGIP
ENSG00000134575	32,10960094	48,66010734	7,046779328	-0,614810113	0,00026607	acid phosphatase 2, lysosom	ACP2
ENSG00000186951	61,18794887	93,6461537	7,818502241	-0,614091689	1,26E-06	peroxisome proliferator acti	PPARA
ENSG00000157224	36,0291556	54,54471341	7,009290675	-0,611862399	2,71E-10	claudin 12	CLDN12
ENSG00000105856	55,99771437	84,17650387	7,845188258	-0,610144499	3,40E-05	HMG-box transcription facto	HBP1
ENSG00000254402	4,501576647	6,922574963	4,620622351	-0,609464825	0,015536857	leucine rich repeat containir	LRRC24
ENSG00000082146	26,89991621	41,04956785	6,480202503	-0,609113677	4,44E-12	STE20 related adaptor beta	STRADB
ENSG00000158805	25,4043271	38,66865278	6,550637255	-0,608994398	0,00347448	zinc finger protein 276	ZNF276
ENSG00000125827	91,82649575	138,5482767	8,372077818	-0,608558163	0,021052883	thioredoxin related transme	TMX4
ENSG00000186409	2,793569531	4,262904739	3,470470311	-0,608294636	0,000108867	coiled-coil domain containin	CCDC30
ENSG00000154309	17,51345405	26,27269251	5,824809522	-0,607047228	0,007554216	dispatched RND transporter	DISP1
ENSG00000175215	199,0409473	299,0936453	9,456955248	-0,605338156	4,94E-06	CTD small phosphatase 2	CTDSP2
ENSG00000166263	26,1679925	40,03288764	6,606378864	-0,605060006	0,014590631	syntaxin binding protein 4	STXBP4
ENSG00000100644	734,8499123	1109,79753	11,31264585	-0,602254661	1,91E-22	hypoxia inducible factor 1 su	HIF1A
ENSG00000115226	18,35731806	27,46348438	6,014194235	-0,601685537	0,008304324	fibronectin type III domain c	FNDC4
ENSG00000113966	6,691996167	10,15986285	4,564745947	-0,598699558	1,98E-05	ADP ribosylation factor like 6	ARL6
ENSG00000231119	3,348504455	5,011538057	3,584093681	-0,598594904	0,000457712	uncharacterized LOC101927	LOC101927377
ENSG00000280351	2,562084476	3,838597382	3,385300715	-0,59796374	0,000481781	uncharacterized LOC100129	LOC100129503
ENSG00000141699	67,53073076	101,9031104	7,959349842	-0,59742629	3,97E-09	reticulophagy regulator fam	RETREG3
ENSG00000188559	71,82282757	107,6261416	7,932094185	-0,597215277	0,012256858	Ral GTPase activating protei	RALGAPA2
ENSG00000168763	43,78241779	66,32822051	7,193509101	-0,59712292	2,56E-08	cyclin and CBS domain divale	CNNM3
ENSG00000112167	12,58582989	18,84126844	5,465048613	-0,596761359	0,001817836	SAYSVFN motif domain cont	SAYSD1
ENSG00000170791	15,59449523	23,59278718	5,765113264	-0,596187671	1,64E-07	coiled-coil-helix-coiled-coil-h	CHCHD7
ENSG00000007944	27,20573932	41,3211222	6,604042503	-0,595541904	0,029918313	myosin regulatory light chain	MYLIP
ENSG00000108840	69,61852325	103,1874189	7,732661916	-0,595264436	0,003865761	histone deacetylase 5	HDAC5
ENSG00000174206	5,494185876	8,309592683	4,36377278	-0,594226807	9,58E-05	KICSTOR subunit 2	KICS2
ENSG00000196998	30,74381968	45,73850895	6,793846957	-0,593768724	2,53E-05	WD repeat domain 45	WDR45
ENSG00000003249	59,41497382	86,80674021	7,472915838	-0,5930242	0,007896018	dysbindin domain containing	DBNDD1
ENSG00000157827	86,37212028	128,6302078	8,240059353	-0,592923321	0,002495098	formin like 2	FMNL2
ENSG00000166886	74,84984289	113,5588995	7,904795134	-0,592706377	1,47E-05	NGFI-A binding protein 2	NAB2
ENSG00000272902	1,212078535	1,834274264	2,136284513	-0,591955465	0,024014756	TBC1D8 antisense RNA 1	TBC1D8-AS1
ENSG00000182324	1,688081912	2,5175337	2,801213979	-0,591862524	0,027828721	potassium inwardly rectifyin	KCNJ14
ENSG00000117151	21,11533418	31,51444881	6,216501147	-0,591723546	0,001108377	chitobiase	CTBS
ENSG00000170854	19,57101644	29,38251803	6,223085862	-0,591568024	0,001410816	ribosomal oxygenase 2	RIOX2
ENSG00000137494	14,97527679	22,50701504	5,903207049	-0,59112578	1,28E-06	ankyrin repeat domain 42	ANKRD42
ENSG00000158467	20,26503449	30,2705403	6,255397684	-0,59099116	0,000107968	adenosylhomocysteinase lik	AHCYL2
ENSG00000169826	28,46324589	42,95728196	6,595921904	-0,590697418	2,76E-08	chondroitin sulfate N-acetyl	CSGALNACT2
ENSG00000102554	88,92234048	130,5568944	7,843021559	-0,589389417	0,009573054	Kruppel like factor 5	KLF5
ENSG00000167536	26,3088462	39,48591664	6,362015781	-0,58733783	0,001232536	dehydrogenase/reductase 1	DHRS13
ENSG00000226950	17,12933233	25,59635954	5,932501125	-0,587320225	0,031830346	differentiation antagonizing	DANCR
ENSG00000173674	92,92748706	133,7421112	8,041454035	-0,587242286	0,000128327	eukaryotic translation initiat	EIF1AX
ENSG00000119711	50,57743236	76,25335283	7,518544717	-0,586560448	4,43E-05	aldehyde dehydrogenase 6 f	ALDH6A1
ENSG00000099326	24,49636287	36,39714482	6,58554046	-0,586303305	0,015065663	myeloid zinc finger 1	MZF1
ENSG00000073417	39,00299608	58,67008056	6,838098238	-0,585472524	6,55E-07	phosphodiesterase 8A	PDE8A
ENSG00000196371	23,25332932	33,8133288	6,00736003	-0,58332843	0,014546322	fucosyltransferase 4	FUT4
ENSG00000178971	45,33272539	68,12326646	7,276798927	-0,582839069	1,12E-07	CST telomere replication cor	CTC1
ENSG00000157214	17,93120158	24,4213529	6,093868122	-0,581793788	0,013365434	STEAP2 metalloreductase	STEAP2
ENSG00000172216	168,4498769	253,4498537	9,317240685	-0,579906265	0,028445904	CCAAT enhancer binding pro	CEBPB
ENSG00000198898	91,09542214	135,9050446	8,262336073	-0,577902707	0,000962799	capping actin protein of mus	CAPZA2
ENSG00000106479	28,63583721	42,3964642	6,842896296	-0,577496102	3,29E-05	zinc finger protein 862	ZNF862
ENSG00000180263	55,28524828	81,67234796	7,366582749	-0,57661422	1,79E-08	FYVE, RhoGEF and PH doma	FGD6
ENSG00000166946	37,44819268	55,74720864	7,104851821	-0,576208574	4,00E-07	cyclin D1 binding protein 1	CCNDBP1
ENSG00000274605	9,208493369	13,76893878	4,712854635	-0,575934263	0,001780456	PCCA divergent transcript	PCCA-DT
ENSG00000244165	8,701306252	12,85323944	5,044641141	-0,574285421	0,000218858	purinergic receptor P2Y11	P2RY11
ENSG00000272686	1,85669836	2,675849713	2,676063762	-0,573931447	0,019043104	WASL divergent transcript	WASL-DT
ENSG00000171234	24,14973444	35,69614071	7,199746164	-0,57316105	0,032684987	UDP glucuronosyltransferas	UGT2B7
ENSG00000114770	63,94143739	95,79602116	7,89002833	-0,573115348	0,002027578	ATP binding cassette subfan	ABCC5
ENSG00000223361	7,627951163	11,35478413	4,989398576	-0,571749807	0,019730633	ferritin heavy chain 1 pseud	FTH1P10
ENSG00000177042	5,729191015	8,524100238	4,324048134	-0,570608818	0,026164504	transmembrane protein 80	TMEM80
ENSG00000006468	28,67289145	42,20945293	6,522874011	-0,569377921	0,026851249	ETS variant transcription fac	ETV1
ENSG00000138166	67,2151579	100,1571228	7,658464026	-0,56731697	0,032292567	dual specificity phosphatase	DUSP5
ENSG00000184838	17,64269595	24,31235525	5,369820556	-0,567194085	0,007663911	proline rich 16	PRR16
ENSG00000138593	81,63003386	120,8816442	8,179410095	-0,566596838	1,05E-10	SECIS binding protein 2 like	SECISBP2L
ENSG00000010818	85,66937075	126,8100475	8,239560319	-0,566511989	7,81E-05	HIVEP zinc finger 2	HIVEP2
ENSG00000130021	25,88607517	36,50890396	6,271352173	-0,566481623	0,007522497	pseudouridine 5'-phosphata	PUDP
ENSG00000186187	43,96519169	65,53397903	7,085604651	-0,566247942	0,003829602	zinc and ring finger 1	ZNRF1
ENSG00000198742	64,07475941	94,2169944	7,662312826	-0,565898439	3,69E-08	SMAD specific E3 ubiquitin p	SMURF1
ENSG00000179715	6,795964509	9,741568102	4,427169481	-0,565028143	0,000521733	PC-esterase domain contain	PCED1B
ENSG00000173599	57,66862944	86,70864099	7,544034228	-0,56460906	0,013870431	pyruvate carboxylase	PC
ENSG00000125384	48,03030569	69,64221171	7,213731181	-0,564495277	0,01103882	prostaglandin E receptor 2	PTGER2
ENSG00000226137	35,12627312	50,55727209	6,983507049	-0,564192701	0,017517018	BAIAP2 divergent transcript	BAIAP2-DT
ENSG00000140564	291,2470162	413,9118793	9,839408381	-0,564086451	0,000212022	furin, paired basic amino aci	FURIN
ENSG00000116661	7,551100509	10,08858666	4,898326054	-0,563512868	0,047143678	F-box protein 2	FBXO2
ENSG00000132361	129,9017968	192,306877	8,870118429	-0,562912616	1,29E-06	clustered mitochondria hom	CLUH
ENSG00000149948	181,1378871	243,8206795	8,852992141	-0,562279776	0,001359655	high mobility group AT-hook	HMGA2
ENSG00000187609	21,57820432	31,60104667	6,531586544	-0,562018986	0,00393704	exonuclease 3'-5' domain co	EXD3
ENSG00000271425	5,275929949	7,801044756	4,09447426	-0,561341678	5,72E-05	NBPF member 10	NBPF10
ENSG00000069493	3,286329276	4,857628865	3,719982888	-0,56097273	0,007846369	C-type lectin domain family	CLEC2D
ENSG00000161011	455,3990549	673,5377698	10,84303762	-0,560688156	1,13E-06	sequestosome 1	SQSTM1
ENSG00000198431	149,9519618	220,776081	8,998589304	-0,560417299	2,61E-10	thioredoxin reductase 1	TXNRD1
ENSG00000109787	60,74386165	89,12241554	7,709061183	-0,55970947	2,57E-05	Kruppel like factor 3	KLF3
ENSG00000169895	110,4418711	155,9126778	8,3154261	-0,559535019	0,000107657	synapse associated protein 1	SYAP1
ENSG00000144136	254,3797502	372,5050927	9,39627299	-0,558920813	0,006036847	solute carrier family 20 men	SLC20A1
ENSG00000163001	17,92297022	26,44512509	5,943910978	-0,558640159	4,82E-05	cilia and flagella associated	CFAP36
ENSG00000167702	91,77016753	136,3800803	8,222865347	-0,557530658	0,048554848	kinesin family member C2	KIFC2
ENSG00000147459	61,03835992	89,91389694	7,586472734	-0,556568517	1,05E-06	dedicator of cytokinesis 5	DOCK5
ENSG00000181523	49,16549816	72,17155383	7,480074493	-0,55632683	0,000797072	N-sulfoglucosamine sulfohy	SGSH
ENSG00000158792	21,72938425	32,03475821	6,177779329	-0,556289706	3,83E-11	spermatogenesis associated	SPATA2L
ENSG00000169249	11,62650874	16,5643985	5,175061464	-0,556111645	3,71E-08	zinc finger CCCH-type, RNA I	ZRSR2
ENSG00000138190	20,50899748	30,02012853	5,859432476	-0,554960481	8,25E-06	exocyst complex componen	EXOC6
ENSG00000130714	36,34079064	53,01508294	7,090232359	-0,554480379	4,37E-21	protein O-mannosyltransfer	POMT1
ENSG00000166900	46,95853975	68,55707565	7,275842786	-0,553564737	0,000582812	syntaxin 3	STX3
ENSG00000160325	30,08858212	43,52867371	6,814126498	-0,553389886	2,00E-06	calcium channel flower dom	CACFD1
ENSG00000224546	7,54093047	10,78876292	4,946875577	-0,552556735	0,033373691	eukaryotic translation initiat	EIF4BP3
ENSG00000172943	42,85550256	62,99834237	7,187103969	-0,551983427	0,000642845	PHD finger protein 8	PHF8
ENSG00000000457	9,925026353	14,53530817	5,182910861	-0,54994275	0,001029215	SCY1 like pseudokinase 3	SCYL3
ENSG00000254206	2,783142701	4,082365711	3,494431952	-0,548601564	0,037241916	nuclear pore complex intera	NPIPB11
ENSG00000130958	18,34334262	26,80389173	5,953550197	-0,548455814	4,59E-06	solute carrier family 35 men	SLC35D2

ENSG00000095303	49,35037821	70,42888466	7,649316385	-0,548197537	0,018750608	prostaglandin-endoperoxide	PTGS1
ENSG00000111254	0,885520749	1,263638064	2,052099955	-0,547617687	0,037494409	A-kinase anchoring protein 3	AKAP3
ENSG00000180185	38,10426289	55,18449461	7,102824634	-0,54745022	3,02E-07	fumarylacetoacetate hydrol	FAHD1
ENSG00000051620	39,89767339	58,19837049	7,170171551	-0,547414718	5,06E-06	heme binding protein 2	HEBP2
ENSG00000198931	77,55752906	111,8642428	7,96940509	-0,5473421	0,006298206	adenine phosphoribosyltran	APRT
ENSG00000127948	73,86062102	108,1319693	8,135615912	-0,546577862	1,87E-07	cytochrome p450 oxidoredu	POR
ENSG00000179941	19,54830601	28,44507968	6,195515786	-0,545738025	2,30E-14	Bardet-Biedl syndrome 10	BBS10
ENSG00000086619	8,208244537	11,94903234	4,93285484	-0,54546565	0,012471979	endoplasmic reticulum oxidi	ERO1B
ENSG00000164327	37,79387384	55,17275136	7,041755359	-0,5439352	2,00E-07	RPTOR independent compa	RICTOR
ENSG00000138036	9,311476978	13,43093798	5,0126612	-0,54389605	0,005473267	dynein cytoplasmic 2 light ir	DYNC2LI1
ENSG00000166272	84,72992855	122,8817155	8,293673294	-0,543492773	1,28E-07	WW domain binding protei	WBP1L
ENSG00000242802	47,62175184	69,18091091	7,265766559	-0,543301878	7,53E-06	adaptor related protein con	AP5Z1
ENSG00000117114	74,5675408	110,2714086	8,127553014	-0,542306834	0,031701082	adhesion G protein-coupled	ADGRL2
ENSG00000066027	65,12519946	95,45019958	7,675790055	-0,54141532	0,017965814	protein phosphatase 2 regul	PPP2R5A
ENSG00000180758	5,379882667	7,81849153	4,320612167	-0,540312785	0,006317113	G protein-coupled receptor	GPR157
ENSG00000247400	2,064983783	2,987730836	2,841941144	-0,5384287	0,034739668	DNAJC3 divergent transcript	DNAJC3-DT
ENSG00000105270	55,17308453	77,77970952	8,005613306	-0,537929523	2,30E-05	CAP-Gly domain containing	CLIP3
ENSG00000230590	17,97307745	25,63098759	6,124082726	-0,537454294	0,010643654	FTX transcript, XIST regulato	FTX
ENSG00000176903	122,8143515	177,385847	8,669660856	-0,536912072	5,51E-05	PNMA family member 1	PNMA1
ENSG00000105854	138,4355122	195,739085	8,41499265	-0,536368169	0,003474173	paraoxonase 2	PON2
ENSG00000250318	0,980487496	1,421526578	1,924689098	-0,535844453	0,030130405	ribosomal protein L13a pseu	RPL13AP26
ENSG00000145214	35,11682809	50,93528575	6,901326204	-0,535691167	0,000747026	diacylglycerol kinase theta	DGKQ
ENSG00000164733	1433,731962	2062,934025	12,05187849	-0,534658664	1,56E-05	cathepsin B	CTSB
ENSG00000188428	20,23793385	29,35215451	6,009589798	-0,534580762	0,004492143	biogenesis of lysosomal orga	BLOC1S5
ENSG00000183793	5,309119462	7,583254634	4,190920264	-0,534319111	0,031310288	nuclear pore complex intera	NPIPA5
ENSG00000197283	72,60201247	105,2382399	8,181444048	-0,533981118	2,45E-05	synaptic Ras GTPase activati	SYNGAP1
ENSG00000165626	23,01241838	32,63375964	6,356317115	-0,53362089	3,13E-05	BEN domain containing 7	BEND7
ENSG00000135749	38,07149621	54,74638435	7,12625455	-0,532248654	2,94E-06	pecanex 2	PCNX2
ENSG00000033867	59,03621396	84,99142044	7,356977945	-0,53192487	0,009050821	solute carrier family 4 meml	SLC4A7
ENSG00000077782	146,9537153	213,2752036	9,12883672	-0,531763722	0,001610711	fibroblast growth factor rec	FGFR1
ENSG00000196693	36,44833988	51,98609226	7,072935092	-0,531576982	0,034731301	zinc finger protein 33B	ZNF33B
ENSG00000132510	69,24022567	98,25482279	8,140154274	-0,531224336	1,80E-05	lysine demethylase 6B	KDM6B
ENSG00000198648	61,71883064	88,07205669	7,396259813	-0,530658404	0,001173842	serine/threonine kinase 39	STK39
ENSG00000157216	36,02600774	49,91397274	7,099319393	-0,529863467	0,016227702	single stranded DNA binding	SSBP3
ENSG00000101577	105,3730323	151,0761653	8,313471291	-0,529072792	0,000334449	lipin 2	LPIN2
ENSG00000161013	310,6481758	446,1919925	9,997417981	-0,52806284	1,18E-13	alpha-1,3-mannosyl-glycopr	MGAT4B
ENSG00000185022	44,01597585	63,64160587	7,182880215	-0,527867259	0,027037036	MAF bZIP transcription facto	MAFF
ENSG00000284308	1,136577312	1,645234724	2,213334894	-0,526910547	0,044396935	chromosome 2 open reading	C2orf81
ENSG00000177663	57,82705158	82,71624821	7,605291429	-0,526896091	3,21E-12	interleukin 17 receptor A	IL17RA
ENSG00000033627	66,56355044	95,89311763	8,022225678	-0,526802369	4,22E-11	ATPase H+ transporting V0 s	ATP6V0A1
ENSG00000062716	133,6391256	190,8573422	8,888601225	-0,526770093	0,000521486	vacuole membrane protein	VMP1
ENSG00000179965	14,22775565	20,53879096	5,687140372	-0,526407397	1,41E-05	zinc finger protein 771	ZNF771
ENSG00000123908	92,15525401	132,5597707	8,169637599	-0,526353892	0,000303776	argonaute RISC catalytic con	AGO2
ENSG00000117481	27,56394605	39,53033256	6,713206748	-0,526220241	0,000716629	NOP2/Sun RNA methyltrans	NSUN4
ENSG00000147894	6,847979258	9,870301587	4,38411025	-0,524761487	6,11E-06	C9orf72-SMCR8 complex sul	C9orf72
ENSG00000152475	3,005289136	4,308674597	3,348882792	-0,523302552	0,001418819	zinc finger protein 837	ZNF837
ENSG00000163328	8,75473058	12,34220692	5,187715089	-0,522559868	5,39E-06	G protein-coupled receptor	GPR155
ENSG00000132640	47,05981526	67,69089503	7,119536159	-0,521951171	0,016456496	BTB domain containing 3	BTBD3
ENSG00000165526	18,97721436	27,30376187	6,154409875	-0,521941005	3,62E-07	RNA pseudouridine synthase	RPUSD4
ENSG00000150712	55,51595533	79,94900435	7,412553886	-0,521564685	7,49E-09	myotubularin related protei	MTMR12
ENSG00000162702	64,4865974	91,8691279	7,603611516	-0,521407538	3,36E-05	zinc finger protein 281	ZNF281
ENSG00000137331	143,1001985	206,4630076	8,949585211	-0,520588045	0,005766093	immediate early response 3	IER3
ENSG00000144182	2,183249262	3,132330681	3,080234769	-0,51846852	0,002769893	lipoyltransferase 1	LIPT1
ENSG00000119979	24,50390165	35,05146128	6,371272896	-0,518400291	3,65E-06	DENN domain containing 10	DENND10
ENSG00000175198	35,60539404	51,37334378	6,992091214	-0,51831579	0,01287055	propionyl-CoA carboxylase s	PCCA
ENSG00000178761	53,86192769	76,86713987	7,599870457	-0,517952048	2,85E-06	family with sequence similar	FAM219B
ENSG00000198894	18,30949547	26,20246051	6,01606555	-0,517726541	5,99E-09	CLOCK interacting pacemak	CIPC
ENSG00000174007	10,08166589	14,43281986	4,92061192	-0,51667068	0,010718878	centrosomal protein 19	CEP19
ENSG00000132970	13,64889346	18,90359508	5,814822642	-0,516543751	0,005485385	WASP family member 3	WASF3
ENSG00000141664	32,29821065	46,24830368	6,765823369	-0,516525896	0,005554219	zinc finger CCHC-type contai	ZCCHC2
ENSG00000185262	82,8885199	118,4715009	7,893853204	-0,51535105	0,003717576	UBA like domain containing	UBALD2
ENSG00000114739	25,17250799	35,57412117	6,461594825	-0,515032007	0,016992482	activin A receptor type 2B	ACVR2B
ENSG00000023287	86,0775661	122,7613636	8,29192889	-0,514984787	4,30E-09	RB1 inducible coiled-coil 1	RB1CC1
ENSG00000113742	75,53412491	107,6135082	8,055570445	-0,514394802	0,000899288	cytoplasmic polyadenylation	CPEB4
ENSG00000080815	111,3082683	158,75798	8,635881715	-0,514351541	8,11E-10	presenilin 1	PSEN1
ENSG00000144283	124,5596153	180,7152767	8,967827138	-0,51391487	0,046968862	plakophilin 4	PKP4
ENSG00000187742	43,30842351	61,82635776	7,2954039	-0,513788389	3,75E-05	SECIS binding protein 2	SECISBP2
ENSG00000158109	43,64576466	62,40188862	7,416654053	-0,513295599	2,00E-11	tumor protein p63 regulatec	TPRG1L
ENSG00000158669	127,9860108	182,4484582	8,786377182	-0,512364635	1,71E-19	glycerol-3-phosphate acyltra	GPAT4
ENSG00000111371	234,3302319	334,8975077	9,536076508	-0,512262034	1,27E-09	solute carrier family 38 men	SLC38A1
ENSG00000152413	16,30357076	22,82617007	5,58122338	-0,511938696	4,82E-08	homer scaffold protein 1	HOMER1
ENSG00000175482	19,35579694	27,43191191	6,16406226	-0,511404498	0,002611237	DNA polymerase delta 4, acc	POLD4
ENSG00000168300	47,22842288	66,84378509	7,544931838	-0,511352736	0,001069783	protein-L-isoaspartate (D-as	PCMTD1
ENSG00000136051	77,44556148	110,5766639	8,094629875	-0,511136698	3,90E-05	WASH complex subunit 4	WASHC4
ENSG00000111725	79,59604157	113,7718698	7,971329651	-0,510718259	0,017410473	protein kinase AMP-activate	PRKAB1
ENSG00000065057	11,70842325	16,62505419	5,501879205	-0,509992076	0,003359305	nth like DNA glycosylase 1	NTHL1
ENSG00000171503	20,02305302	28,28105861	6,255015129	-0,509676503	0,001655024	electron transfer flavoprote	ETFDH
ENSG00000132376	31,24247534	44,52068766	6,735884207	-0,509055734	1,13E-05	inositol polyphosphate-5-ph	INPP5K
ENSG00000147592	27,63015915	38,96603138	6,160144096	-0,508512115	0,014774268	lactamase beta 2	LACTB2
ENSG00000165434	37,40378516	45,40207627	6,693680231	-0,507917841	0,034694869	phosphoglucomutase 2 like	PGM2L1
ENSG00000135070	23,94595911	34,1493924	6,481004476	-0,50765258	1,28E-07	iron-sulfur cluster assembly	ISCA1
ENSG00000125814	7,175727495	10,22277266	4,757212615	-0,507487948	0,000633941	NSF attachment protein bet	NAPB
ENSG00000137962	288,6469047	397,8834277	9,745418546	-0,507455942	0,000253101	Rho GTPase activating prote	ARHGAP29
ENSG00000065615	15,964474	22,74579828	5,729298686	-0,507092107	1,95E-05	cytochrome b5 reductase 4	CYB5R4
ENSG00000196954	56,05142949	79,83242378	7,443917659	-0,506548856	6,79E-07	caspase 4	CASP4
ENSG00000110619	46,61505004	66,33220776	7,426716624	-0,506252893	1,84E-08	cysteinyl-tRNA synthetase 1	CARS1
ENSG00000205808	10,12365267	14,32531139	5,166140648	-0,506114392	5,56E-05	phospholipid phosphatase 6	PLPP6
ENSG00000064932	305,6280115	434,685168	10,05231791	-0,50559941	1,06E-06	strawberry notch homolog 2	SBNO2
ENSG00000163703	69,79002576	97,2275339	8,179427858	-0,504541987	0,001959385	cysteine rich with EGF like d	CRELD1
ENSG00000188542	3,309346123	4,66525125	3,256110476	-0,504539831	0,004953217	dual specificity phosphatase	DUSP28
ENSG00000140941	92,33437825	129,6736889	8,37167567	-0,504114798	1,94E-11	microtubule associated prot	MAP1LC3B
ENSG00000253958	13,08218443	18,50668989	5,136905059	-0,503879523	0,00982797	claudin 23	CLDN23
ENSG00000205593	10,1511498	14,40583478	5,215370263	-0,503481813	0,000161976	DENN domain containing 6B	DENND6B
ENSG00000013364	349,3083398	494,190306	10,32297244	-0,503279852	1,97E-06	major vault protein	MVP
ENSG00000182405	3,988211445	5,570115585	3,748887706	-0,503111612	0,003595246	piggyBac transposable elem	PGBD4
ENSG00000174405	19,81600109	27,85447944	6,166253168	-0,502809263	2,06E-05	DNA ligase 4	LIG4
ENSG00000075234	36,6467564	51,69432379	6,982512157	-0,502735644	0,041659275	tetratricopeptide repeat doi	TTC38
ENSG00000261824	7,113203971	10,01398685	4,636716988	-0,50261031	1,48E-05	long intergenic non-protein	LINC00662
ENSG00000184428	22,3990405	31,77312505	6,291891033	-0,502470811	0,000201431	DNA topoisomerase I mitoch	TOP1MT
ENSG00000166401	24,9213091	35,42253612	6,18547136	-0,502395084	0,002049777	serpin family B member 8	SERPINB8
ENSG00000089351	88,66111613	123,7019504	8,285753034	-0,501232925	3,57E-11	GRAM domain containing 1A	GRAMD1A
ENSG00000110888	39,20634789	56,25695819	6,85733113	-0,500867026	0,026804302	caprin family member 2	CAPRIN2
ENSG00000261373	11,68363795	16,14111855	4,986481363	-0,500456622	0,032951307	VPS9D1 antisense RNA 1	VPS9D1-AS1
ENSG00000176973	4,522914402	6,303469789	3,908742858	-0,50043992	0,004067052	family with sequence similar	FAM89B
ENSG00000179562	39,54508771	55,95836473	7,238946815	-0,499169882	9,76E-25	GRIP and coiled-coil domain	GCC1
ENSG00000167700	24,40416164	34,17306782	6,71236587	-0,499068338	1,18E-06	major facilitator superfamily	MFSD3
ENSG00000088766	33,21344724	46,63393846	6,83787397	-0,498416126	0,001291831	cardiolipin synthase 1	CRLS1
ENSG00000101132	8,928233395	12,63660072	4,929516418	-0,497773775	0,000106017	prefoldin subunit 4	PFDN4
ENSG00000154144	40,4114327	57,16135902	7,227568867	-0,496830755	8,60E-16	transforming growth factor	TBRG1
ENSG00000263465	37,53394416	52,21786963	7,070564757	-0,496247113	8,31E-05	serine and arginine rich splic	SRSF8
ENSG00000079950	56,33336188	79,36783367	7,589643325	-0,495616863	1,33E-08	syntaxin 7	STX7
ENSG00000115993	119,5684235	169,0464998	8,622360768	-0,495580929	5,22E-10	trafficking kinesin protein 2	TRAK2
ENSG00000051523	221,0799535	311,7955752	9,54705777	-0,495406327	1,09E-05	cytochrome b-245 alpha cha	CYBA
ENSG00000248593	4,958220387	6,970208226	4,085011228	-0,495200051	0,017289725	DSTN pseudogene 2	DSTNP2
ENSG00000162735	40,08715217	56,5493988	7,213309439	-0,494969595	9,65E-14	peroxisomal biogenesis facto	PEX19

ENSG00000139636	24,27126111	34,09651607	6,634328234	-0,494539071	0,008883421	limb development membrar	LMBR1L
ENSG00000099282	33,80363	46,2682208	6,770677024	-0,494200008	0,000833383	tetraspanin 15	TSPAN15
ENSG00000173221	33,75798896	47,58668603	6,793527968	-0,493546106	0,001492475	glutaredoxin	GLRX
ENSG00000204634	84,17941093	118,9680727	7,805901743	-0,493450008	0,016891494	TBC1 domain family membe	TBC1D8
ENSG00000011009	95,93307766	132,9589878	8,350660786	-0,493103649	0,000109358	lysophospholipase 2	LYPLA2
ENSG00000198839	19,22361231	26,88072027	6,233777961	-0,49297595	0,001782842	zinc finger protein 277	ZNF277
ENSG00000006118	158,9189913	215,7377712	9,172632122	-0,492660636	0,016827872	transmembrane protein 132	TMEM132A
ENSG00000186350	117,4237121	163,1061042	8,53666309	-0,492327031	0,035062604	retinoid X receptor alpha	RXRA
ENSG00000165943	62,53701164	87,23205664	7,613903251	-0,491863911	0,006049163	modulator of apoptosis 1	MOAP1
ENSG00000138029	89,60370952	126,1321418	8,268217055	-0,491639116	0,000704793	hydroxyacyl-CoA dehydroge	HADHB
ENSG00000197822	13,97287253	19,1434786	5,183629498	-0,491503694	0,00182884	occludin	OCLN
ENSG00000151575	10,33074067	14,44541403	5,162150316	-0,49149683	0,015087786	testis expressed 9	TEX9
ENSG00000018869	1,68959289	2,360636849	2,634058055	-0,491487983	0,036441764	zinc finger protein 582	ZNF582
ENSG00000068796	89,00052172	123,8087204	8,021620193	-0,491214619	0,032091649	kinesin family member 2A	KIF2A
ENSG00000177311	210,8411772	298,2788132	9,693520867	-0,490563041	0,012473068	zinc finger and BTB domain	ZBTB38
ENSG00000113971	5,416155542	7,628805161	4,209436751	-0,49032299	0,002728632	nephrocystin 3	NPHP3
ENSG00000171612	6,543581338	9,197662655	4,222726674	-0,490167139	0,00295009	solute carrier family 25 men	SLC25A33
ENSG00000177674	56,2611052	79,00194504	7,542467947	-0,490002485	1,92E-05	angiotensin II receptor asso	AGTRAP
ENSG00000080608	39,54203484	55,73269302	7,146711438	-0,489655106	0,000160496	pumilio RNA binding family	PUM3
ENSG00000136367	25,36620057	35,1097095	6,418341689	-0,489083754	0,010188802	zinc finger homeobox 2	ZFHX2
ENSG00000160888	77,29936807	108,350763	7,940580777	-0,489052691	2,25E-06	immediate early response 2	IER2
ENSG00000226742	10,96049907	15,40602974	5,380766688	-0,488677564	3,10E-05	heat shock factor binding pr	HSBP1L1
ENSG00000112624	20,14027181	27,93726912	6,211793495	-0,487662044	0,000320868	BICRA like chromatin remod	BICRAL
ENSG00000254860	4,441288651	6,239878938	4,28184725	-0,487360597	0,001748217	TMEM9B antisense RNA 1	TMEM9B-AS1
ENSG00000173442	140,2709387	195,4772086	8,604132235	-0,487240248	1,09E-10	EH domain binding protein 1	EHBP1L1
ENSG00000169193	4,614858318	6,41043162	3,983530323	-0,487017092	0,015087786	coiled-coil domain containin	CCDC126
ENSG00000100744	22,32913173	31,36312922	6,144852245	-0,486576106	0,018367513	GSK3B interacting protein	GSKIP
ENSG00000169087	6,566284873	9,223907831	4,552620759	-0,486545497	0,009682915	HSPB1 associated protein 1	HSPBAP1
ENSG00000235106	37,49231287	52,43297377	7,26961461	-0,486334676	1,44E-05	BRD3 opposite strand	BRD3OS
ENSG00000164022	30,77350493	43,2522779	6,647208708	-0,486204637	2,37E-13	aminoacyl tRNA synthetase	AIMP1
ENSG00000006757	22,09864481	29,85621145	6,016784872	-0,486049053	0,005958837	patatin like phospholipase d	PNPLA4
ENSG00000160972	48,97508322	68,64148727	7,335913318	-0,485467162	4,74E-07	protein phosphatase 1 regul	PPP1R16A
ENSG00000197312	45,52908814	63,88991659	7,276404181	-0,484633555	1,70E-08	DNA damage inducible 1 ho	DDI2
ENSG00000100307	20,76883919	29,07920958	6,230545573	-0,484599003	0,003078863	chromobox 7	CBX7
ENSG00000130810	4,179304243	5,797185639	4,019527539	-0,484083377	0,001478628	peter pan homolog	PPAN
ENSG00000148331	46,69104616	65,29320889	7,37329353	-0,484028986	3,29E-07	ankyrin repeat and SOCS bo	ASB6
ENSG00000110422	110,0909224	153,677309	8,476812387	-0,48301776	0,001298009	homeodomain interacting p	HIPK3
ENSG00000174373	29,28498566	40,88174251	6,616335726	-0,482952661	0,000172534	Ral GTPase activating protei	RALGAPA1
ENSG00000181638	20,84181778	29,15377624	6,35727865	-0,482646416	4,01E-08	ZFP41 zinc finger protein	ZFP41
ENSG00000232040	6,03805043	8,391646558	4,390125951	-0,482387837	0,029451701	zinc finger BED-type contain	ZBED9
ENSG00000268043	17,51512308	24,48903591	6,001388854	-0,482340473	1,27E-06	NBPF member 12	NBPF12
ENSG00000234420	28,84397725	40,19257183	6,637776215	-0,482089486	0,007104131	zinc finger protein 37B, pseu	ZNF37BP
ENSG00000121879	37,11718837	51,76382332	6,956034113	-0,481875481	2,48E-10	phosphatidylinositol-4,5-bis	PIK3CA
ENSG00000165006	112,2414186	155,7309831	8,612803738	-0,48176768	0,004724881	ubiquitin associated protein	UBAP1
ENSG00000103042	39,9540245	55,60906417	7,280205141	-0,481230931	3,44E-07	solute carrier family 38 men	SLC38A7
ENSG00000204947	6,348702761	8,718799678	4,618398389	-0,480685885	0,012273748	zinc finger protein 425	ZNF425
ENSG00000105520	43,36668511	58,89535422	7,371805215	-0,480153688	0,002606905	phospholipid phosphatase r	PLPPR2
ENSG00000112062	86,81641816	121,4061597	8,150223568	-0,479640061	7,71E-25	mitogen-activated protein k	MAPK14
ENSG00000177425	132,434356	186,4019229	8,768624263	-0,479538412	0,013162042	pro-apoptotic WT1 regulato	PAWR
ENSG00000103254	20,32165573	28,31996695	6,263099673	-0,479407226	0,001291532	adenine nucleotide transloc	ANTKMT
ENSG00000213609	1,48229953	2,070581411	2,512132473	-0,47924988	0,03398091	ribosomal protein L7a pseuc	RPL7AP50
ENSG00000166140	54,75165858	75,74774183	7,558164261	-0,479123309	0,000393279	zinc finger FYVE-type contai	ZFYVE19
ENSG00000126012	188,2891478	254,3970819	9,192705567	-0,478977324	1,98E-12	lysine demethylase 5C	KDM5C
ENSG00000119328	11,24551386	15,70806043	5,342729027	-0,47872483	1,46E-07	actin binding transcription n	ABITRAM
ENSG00000185989	65,63355697	91,46663707	7,829556439	-0,478342603	5,52E-05	RAS p21 protein activator 3	RASA3
ENSG00000197063	37,86576466	52,7706059	7,104443558	-0,4782697	0,000169511	MAF bZIP transcription facto	MAFG
ENSG00000138660	19,57718734	27,31128596	6,008049244	-0,478109006	1,17E-07	adaptor related protein com	AP1AR
ENSG00000104714	22,35314365	31,25482431	6,300273704	-0,477797215	0,000227479	glutamate rich 1	ERICH1
ENSG00000064999	44,98530408	62,14893647	7,293868501	-0,477509157	0,000630908	ankyrin repeat and sterile al	ANKS1A
ENSG00000078967	16,01314702	22,32268442	5,91975687	-0,477178989	9,98E-07	ubiquitin conjugating enzym	UBE2D4
ENSG00000130517	55,55634253	77,01636542	7,776548475	-0,476749708	1,68E-05	pyroglutamyl-peptidase I	PGPEP1
ENSG00000165185	27,88575438	38,98494831	6,495110914	-0,474790665	0,010171697	KIAA1958	KIAA1958
ENSG00000136603	57,45698829	77,1708517	7,570717619	-0,474621755	0,00105487	SKI like proto-oncogene	SKIL
ENSG00000124574	29,01603033	40,45686663	6,871755439	-0,474520171	0,00764469	ATP binding cassette subfan	ABCC10
ENSG00000137955	23,14323082	32,12144206	6,35455166	-0,47409851	2,55E-10	Rab geranylgeranyltransfera	RABGGTB
ENSG00000090857	76,79835487	105,7886332	8,336832696	-0,4738288	2,32E-05	pyruvate dehydrogenase ph	PDPR
ENSG00000139160	1,410422498	1,947034167	2,613056002	-0,473002091	0,037806461	electron transfer flavoprote	ETFBKMT
ENSG00000256594	1,677244862	2,339922748	2,467437427	-0,473000727	0,028918322	C-type lectin domain family	LOC374443
ENSG00000114796	57,71457934	80,2751475	7,932353953	-0,472930675	1,33E-05	kelch like family member 24	KLHL24
ENSG00000197860	20,64629145	28,66941308	6,099912802	-0,471237811	0,000151927	small glutamine rich tetratri	SGTB
ENSG00000119900	111,1789653	154,6880513	8,507619884	-0,470873868	0,010480866	opioid growth factor recepto	OGFRL1
ENSG00000148814	6,5124038	9,046481995	4,669104603	-0,469953693	0,001722057	leucine rich repeat containir	LRRC27
ENSG00000076984	59,30961422	82,24097767	7,752628158	-0,469561959	6,41E-09	mitogen-activated protein k	MAP2K7
ENSG00000171100	8,641956838	11,99371037	4,931635213	-0,469306791	6,57E-05	myotubularin 1	MTM1
ENSG00000236753	3,170230672	4,384871568	3,348489862	-0,46909436	0,040716691	MKLN1 antisense RNA	MKLN1-AS
ENSG00000105373	155,0350728	213,3776748	9,137390698	-0,468870208	0,00015265	NOP53 ribosome biogenesis	NOP53
ENSG00000143164	79,58207924	109,3405792	8,067640562	-0,468713936	0,000238792	DDB1 and CUL4 associated f	DCAF6
ENSG00000106701	4,099285923	5,659315977	3,684620794	-0,468063333	0,007082365	fibronectin type III and SPRY	FSD1L
ENSG00000006652	41,04916624	56,87266843	6,940737996	-0,468031677	0,000420202	interferon related developm	IFRD1
ENSG00000213380	12,69578878	17,60971978	5,455574855	-0,467802015	0,00050978	component of oligomeric go	COG8
ENSG00000129003	82,69986694	114,559195	8,065451888	-0,467441357	5,29E-05	vacuolar protein sorting 13	VPS13C
ENSG00000182359	5,091208811	7,049351525	4,311719285	-0,467133619	0,000668346	kelch repeat and BTB domai	KBTBD3
ENSG00000103037	10,76390099	14,91021827	5,237466348	-0,466681418	0,001131389	SET domain containing 6, pr	SETD6
ENSG00000165714	12,55948092	17,29084226	5,30959452	-0,46615438	3,27E-06	BLOC-1 related complex sub	BORCS5
ENSG00000198960	12,14203851	16,6161072	5,515653929	-0,466111954	0,003416907	armadillo repeat containing	ARMCX6
ENSG00000163322	14,15133723	19,58866135	5,727142878	-0,465979595	0,000285904	abraxas 1, BRCA1 A complex	ABRAXAS1
ENSG00000115841	2,573703385	3,571405453	3,256655612	-0,465806963	0,014727222	regulator of microtubule dy	RMDN2
ENSG00000048471	45,91595262	63,8101518	7,305057347	-0,46546075	0,043576905	sorting nexin 29	SNX29
ENSG00000138459	28,62255292	39,58802518	6,615241598	-0,465125605	1,63E-09	solute carrier family 35 men	SLC35A5
ENSG00000119844	35,71581643	49,37096187	6,926299494	-0,465080937	1,09E-05	aftiphilin	AFTPH
ENSG00000183208	3,901226248	5,381259626	3,731622337	-0,464941279	0,021713841	GDP-D-glucose phosphoryla	GDPGP1
ENSG00000145555	266,6986893	367,5951862	9,640252733	-0,464907019	0,000336812	myosin X	MYO10
ENSG00000115289	9,188360859	12,70126741	5,128926989	-0,464679751	0,01024702	polycomb group ring finger	PCGF1
ENSG00000109572	110,6758042	152,3895315	8,585503528	-0,464652728	5,07E-08	chloride voltage-gated chan	CLCN3
ENSG00000141026	22,94568434	31,80094048	6,33489136	-0,464626075	0,007767747	mediator complex subunit 9	MED9
ENSG00000164366	30,70621379	42,04315516	6,647749589	-0,463881339	0,000178878	coiled-coil domain containin	CCDC127
ENSG00000089091	3,618471915	5,010261144	3,780412723	-0,463803488	0,002451829	double zinc ribbon and anky	DZANK1
ENSG00000240230	12,08896826	16,69215073	5,433520482	-0,463639047	1,84E-08	cytochrome c oxidase assen	COX19
ENSG00000112379	57,69372496	77,99795201	7,070072131	-0,463409288	0,010086397	ARFGEF family member 3	ARFGEF3
ENSG00000163683	58,42556846	79,91444143	7,525492524	-0,463192111	0,038913853	small integral membrane pro	SMIM14
ENSG00000114933	30,65543689	42,24640839	6,649261128	-0,46232251	0,001715789	INO80 complex subunit D	INO80D
ENSG00000100577	13,36257752	18,39485316	5,549293742	-0,462196464	1,97E-09	glutathione S-transferase ze	GSTZ1
ENSG00000132589	208,0795081	287,3164902	9,321702071	-0,461504797	7,58E-09	flotillin 2	FLOT2
ENSG00000118496	29,64139229	40,93102518	6,743491414	-0,461440401	2,68E-06	F-box protein 30	FBXO30
ENSG00000164715	69,26766889	95,39944723	7,831639069	-0,461218763	1,50E-06	lemur tyrosine kinase 2	LMTK2
ENSG00000100413	40,34033872	55,45327812	7,14302731	-0,460240774	6,07E-06	RNA polymerase III subunit	POLR3H
ENSG00000217648	1,918859492	2,639738865	2,71568339	-0,459784614	0,032392695	presenilins-associated rhom	LOC100131471
ENSG00000170946	13,95573402	18,95420226	5,38811751	-0,459749778	0,000660835	DnaJ heat shock protein fam	DNAJC24
ENSG00000111897	179,1180735	244,7617594	9,344905734	-0,459668416	1,26E-05	serine incorporator 1	SERINC1
ENSG00000177666	181,1871445	248,6563904	9,383347064	-0,458952431	4,68E-06	patatin like phospholipase d	PNPLA2
ENSG00000197442	48,1138772	64,93004762	7,371328472	-0,458773962	0,002753825	mitogen-activated protein k	MAP3K5
ENSG00000141012	75,20333733	103,2373356	8,169731559	-0,458083859	0,001555219	galactosamine (N-acetyl)-6-s	GALNS
ENSG00000109680	14,55653637	18,77117307	5,503238247	-0,457844511	0,019789945	TBC1 domain family membe	TBC1D19
ENSG00000143384	477,8191857	657,9517977	10,62404712	-0,457780395	1,91E-07	MCL1 apoptosis regulator, B	MCL1
ENSG00000116473	55,5713074	76,11909103	7,527906083	-0,457339223	0,005368788	RAP1A, member of RAS onc	RAP1A

ENSG00000124802	6,214616437	8,547967328	4,126818337	-0,457063158	0,013016242	eukaryotic translation elong	EEF1E1
ENSG00000188997	19,32982921	26,44802783	6,227693899	-0,45680481	1,21E-07	potassium channel tetramer	KCTD21
ENSG00000058600	36,76550934	50,53064784	7,06562772	-0,456222342	1,36E-06	RNA polymerase III subunit I	POLR3E
ENSG00000215114	62,73278482	86,16415518	7,697822443	-0,456136923	0,000332486	UBX domain protein 2B	UBXN2B
ENSG00000168216	31,28466829	42,32740619	6,775946549	-0,456100158	0,04223807	LMBR1 domain containing 1	LMBRD1
ENSG00000263956	7,041485372	9,666613046	4,586529977	-0,45606702	8,25E-06	NBPF member 11	NBPF11
ENSG00000175970	73,98312353	101,3554285	8,01014361	-0,455638694	0,000129325	unc-119 lipid binding chaper	UNC119B
ENSG00000120925	20,22622145	27,47774724	6,187662186	-0,455434391	3,40E-06	ring finger protein 170	RNF170
ENSG00000109270	35,4829755	48,61008633	6,968627765	-0,454473247	1,57E-05	late endosomal/lysosomal a	LAMTOR3
ENSG00000174989	26,61289802	36,38011017	6,700464821	-0,45434279	3,30E-05	F-box and WD repeat domai	FBXW8
ENSG00000271122	16,85948195	22,99967134	5,882776677	-0,45424563	1,64E-06	HERPUD2 antisense RNA 1	HERPUD2-AS1
ENSG00000070159	71,4900508	98,16251699	7,753353409	-0,453933478	0,029059675	protein tyrosine phosphatas	PTPN3
ENSG00000031003	25,54024063	35,01315107	6,432203581	-0,452141763	1,43E-05	family with sequence simila	FAM13B
ENSG00000164114	28,34282883	38,70477209	6,77320319	-0,452036575	0,002075237	microtubule associated prot	MAP9
ENSG00000166562	18,23363171	25,02871178	6,033340886	-0,451095995	0,000166653	SEC11 homolog C, signal pep	SEC11C
ENSG00000234608	6,288751315	8,618859613	4,466929541	-0,450479392	0,000964624	MAPKAPK5 antisense RNA 1	MAPKAPK5-AS1
ENSG00000145623	220,4818763	300,6453523	9,593215979	-0,45003453	0,013488478	oncostatin M receptor	OSMR
ENSG00000173928	2,875689687	3,938430195	3,293148052	-0,449612837	0,008413559	SWIM-type zinc finger 7 asso	SWSAP1
ENSG00000172725	142,6002298	195,0743495	8,969984628	-0,448899178	3,09E-12	coronin 1B	CORO1B
ENSG00000165650	81,21419806	110,4415903	8,075373447	-0,448101447	0,010857469	PDZ domain containing 8	PDZD8
ENSG00000170185	42,41695275	57,95112815	7,245839666	-0,447599224	3,74E-11	ubiquitin specific peptidase	USP38
ENSG00000091317	126,9351482	170,5056091	8,566626042	-0,447436622	1,03E-07	CKLF like MARVEL transmem	CMTM6
ENSG00000215301	316,400632	420,4736439	9,860098579	-0,446774978	2,42E-06	DEAD-box helicase 3 X-linke	DDX3X
ENSG00000142327	111,5624512	151,4392545	8,479415798	-0,446481391	4,35E-09	arginyl aminopeptidase like	RNPEPL1
ENSG00000182552	11,54437738	15,74724643	5,217863859	-0,446481095	1,23E-05	RWD domain containing 4	RWDD4
ENSG00000148334	38,42658942	52,25436263	7,252794836	-0,446461633	5,38E-07	prostaglandin E synthase 2	PTGES2
ENSG00000204070	21,61035068	29,39958745	6,274209075	-0,446375206	0,001438563	SYS1 golgi trafficking protein	SYS1
ENSG00000116044	163,0230779	222,820763	9,148393883	-0,445804374	0,044416159	NFE2 like bZIP transcription	NFE2L2
ENSG00000058799	21,7166304	29,60615794	6,270467476	-0,445733405	0,000682223	Yip1 domain family member	YIPF1
ENSG00000125148	363,1268685	494,8748827	10,25829534	-0,445385752	2,26E-06	metallothionein 2A	MT2A
ENSG00000186666	5,090046762	6,94193278	4,340464848	-0,444351638	0,00378024	BCDIN3D domain containing	BCDIN3D
ENSG00000167996	1307,001077	1786,898098	12,32895718	-0,443870937	0,009702786	ferritin heavy chain 1	FTH1
ENSG00000132906	13,08353441	17,78021517	5,410688402	-0,443626904	5,90E-05	caspase 9	CASP9
ENSG00000185163	29,56218649	40,16439507	6,440898657	-0,443601814	0,000716629	DEAD-box helicase 51	DDX51
ENSG00000123815	25,52074642	34,59682594	6,403479261	-0,443168594	0,000570051	coenzyme Q8B	COQ8B
ENSG00000234741	29,75299559	40,47578682	6,924383536	-0,442961204	0,000227187	growth arrest specific 5	GAS5
ENSG00000166855	40,95952246	55,76142613	7,221628654	-0,442942743	0,001029215	caseinolytic mitochondrial m	CLPX
ENSG00000145545	26,93747243	36,36576389	6,276719202	-0,442374699	0,007689109	steroid 5 alpha-reductase 1	SRD5A1
ENSG00000141570	8,780444745	11,97927738	4,937280355	-0,442009582	0,006100908	chromobox 8	CBX8
ENSG00000136044	64,41584841	86,61799229	7,740431788	-0,441897198	8,82E-05	adaptor protein, phosphotyr	APPL2
ENSG00000119688	47,84889814	64,90941336	7,422300069	-0,441536259	0,001134803	ATP binding cassette subfan	ABCD4
ENSG00000174705	123,7391626	163,5496157	8,478207936	-0,441033734	0,000201431	SH3 and PX domains 2B	SH3PXD2B
ENSG00000271383	22,31436078	30,31978565	6,245637568	-0,440845686	0,000859983	NBPF member 19	NBPF19
ENSG00000148187	20,12842218	27,35450983	6,216584071	-0,440795893	1,21E-06	mitochondrial ribosome rec	MRRF
ENSG00000137942	101,763989	136,1691549	8,289865017	-0,439939619	0,032057444	formin binding protein 1 like	FNBP1L
ENSG00000164695	18,446912	24,95268164	5,831084839	-0,439518691	0,028722613	charged multivesicular body	CHMP4C
ENSG00000109854	47,0667963	63,91640095	7,483751616	-0,439393194	0,00284005	HIV-1 Tat interactive protein	HTATIP2
ENSG00000089195	23,15991138	31,44782821	6,169724064	-0,439223682	3,34E-06	tRNA methyltransferase 6 n	TRMT6
ENSG00000153485	6,281681934	8,539234062	4,362513538	-0,438829584	0,001940753	transmembrane protein 251	TMEM251
ENSG00000090238	55,02532056	74,7377864	7,709297642	-0,437988649	0,020374142	yippee like 3	YPEL3
ENSG00000215190	4,590319093	6,232167504	4,283484694	-0,437539757	0,030758994	long intergenic non-protein	LINC00680
ENSG00000181852	48,87375601	65,75969141	7,457725887	-0,437329899	8,56E-05	ring finger protein 41	RNF41
ENSG00000105948	10,73950673	14,56070393	5,082380946	-0,43708191	2,13E-05	tetratricopeptide repeat dor	TTC26
ENSG00000197006	94,09189403	127,3973745	8,209019191	-0,437043464	5,07E-06	methyltransferase like 9	METTL9
ENSG00000167740	14,59433399	19,29311538	5,78548341	-0,435657601	0,035256403	cytochrome b5 domain cont	CYB5D2
ENSG00000260549	90,25160853	122,7060254	8,271498067	-0,435266338	0,013547505	metallothionein 1L, pseudog	MT1L
ENSG00000157741	27,89658455	37,73537689	6,7085414	-0,434999368	0,002510106	ubinuclein 2	UBN2
ENSG00000088888	256,7240803	347,1805971	9,793551343	-0,434956207	8,51E-08	mitochondrial antiviral signa	MAVS
ENSG00000146433	60,75479661	81,74810797	7,648567281	-0,434129806	5,50E-07	transmembrane protein 181	TMEM181
ENSG00000158470	158,3397156	201,8199555	8,697914846	-0,433884524	0,004237964	beta-1,4-galactosyltransfera	B4GALT5
ENSG00000100592	27,80166988	37,42417508	6,227083039	-0,433480461	0,000253451	dishevelled associated activ	DAAM1
ENSG00000143353	12,05870646	16,34487151	5,430247982	-0,433293876	0,006426446	lysophospholipase like 1	LYPLAL1
ENSG00000130939	83,45866661	112,2091523	8,124023144	-0,433204325	0,000133059	ubiquitination factor E4B	UBE4B
ENSG00000112182	13,83259989	18,07148794	5,075069811	-0,433020791	0,047219175	BTB domain and CNC homol	BACH2
ENSG00000188419	44,60622209	59,91963301	7,098242143	-0,432799037	0,000655445	CHM Rab escort protein	CHM
ENSG00000267169	5,948452835	8,020654411	4,363515389	-0,432205221	0,007862151	ADGRL1 antisense RNA 1	ADGRL1-AS1
ENSG00000182287	49,38246892	64,56472174	6,854419679	-0,431925426	0,005729772	adaptor related protein com	AP1S2
ENSG00000091844	18,37785659	24,82038922	5,82684047	-0,431649375	0,000626978	regulator of G protein signal	RGS17
ENSG00000167114	60,88038745	82,2492924	7,685264105	-0,43153081	0,000181681	solute carrier family 27 men	SLC27A4
ENSG00000273820	6,165776274	8,332243197	4,514020956	-0,43152511	0,004248354	ubiquitin specific peptidase	USP27X
ENSG00000172932	48,96315302	66,23835531	7,383570938	-0,430947743	0,0009262	ankyrin repeat domain 13D	ANKRD13D
ENSG00000169814	14,30212199	19,32084084	5,846522242	-0,430832883	0,001636463	biotinidase	BTD
ENSG00000167065	10,3745934	14,00384351	5,214792794	-0,430695825	1,42E-05	dual specificity phosphatase	DUSP18
ENSG00000131871	30,93062852	41,71053052	6,68862439	-0,430614197	9,38E-06	selenoprotein S	SELENOS
ENSG00000163013	26,28841243	35,34767609	6,578534908	-0,430255357	0,000178878	F-box protein 41	FBXO41
ENSG00000226688	7,848642091	10,61586866	4,639420562	-0,430007328	0,002993265	ENTPD1 antisense RNA 1	ENTPD1-AS1
ENSG00000132824	138,5519966	185,5441731	8,946472774	-0,429147819	7,54E-07	serine incorporator 3	SERINC3
ENSG00000135404	337,1468522	450,4023621	10,21529841	-0,428649543	3,56E-05	CD63 molecule	CD63
ENSG00000173575	156,6113367	210,7315068	9,091435188	-0,42810094	0,000959321	chromodomain helicase DN/	CHD2
ENSG00000103707	13,87244713	18,71462701	5,460513893	-0,428023334	1,36E-07	mitochondrial methionyl-tRI	MTFMT
ENSG00000119661	11,46864854	15,47396002	5,330586916	-0,427573049	0,000205424	dynein axonemal light chain	DNAL1
ENSG00000104823	24,85056063	33,41335175	6,502867801	-0,427265146	0,002560747	enoyl-CoA hydratase 1	ECH1
ENSG00000118816	384,2654687	516,379483	10,42940147	-0,427204856	4,73E-05	cyclin I	CCNI
ENSG00000185825	135,701557	182,4340615	8,843758645	-0,426516023	1,92E-13	B cell receptor associated pr	BCAP31
ENSG00000184307	10,13245636	13,55783522	5,008967628	-0,426308492	0,010555436	zinc finger DHHC-type palmi	ZDHHC23
ENSG00000129625	97,84564287	130,9448571	8,357883069	-0,425397615	0,005954336	receptor accessory protein 5	REEP5
ENSG00000062725	55,42703551	74,56488257	7,565368535	-0,425229374	1,93E-12	amyloid beta precursor prot	APPBP2
ENSG00000185305	15,44609113	20,78913449	5,634700007	-0,425117431	0,010237262	ADP ribosylation factor like 1	ARL15
ENSG00000159917	3,677006772	4,889986056	3,651125292	-0,424948069	0,04625345	zinc finger protein 235	ZNF235
ENSG00000083457	7,090626853	9,532248294	4,53525859	-0,424834019	0,001777492	integrin subunit alpha E	ITGAE
ENSG00000083223	47,98117646	64,44055231	7,437692945	-0,424747083	0,000206495	terminal uridylyl transferase	TUT7
ENSG00000169756	185,9505848	229,6004545	9,074323621	-0,424592225	0,013968505	LIM zinc finger domain cont	LIMS1
ENSG00000158828	98,78434742	132,5596673	8,662667352	-0,424229886	0,000411424	PTEN induced kinase 1	PINK1
ENSG00000102908	89,73794681	119,6704554	8,238854807	-0,423572491	0,007064137	nuclear factor of activated T	NFAT5
ENSG00000258301	3,153047294	4,229080719	3,616683101	-0,423512563	0,008622162	VASH1 antisense RNA 1	VASH1-AS1
ENSG00000073712	100,437748	134,8861152	8,355670633	-0,423148764	7,26E-06	FERM domain containing kir	FERMT2
ENSG00000162923	160,9622492	215,3335211	9,116134743	-0,422630331	8,19E-10	WD repeat domain 26	WDR26
ENSG00000238227	96,45196812	128,6900953	8,348103887	-0,421287415	0,000129906	transmembrane protein 250	TMEM250
ENSG00000260917	7,986297379	10,70950121	4,839838884	-0,420804408	0,000387345	uncharacterized LOC103344	LOC103344931
ENSG00000138434	163,6622689	214,5140777	8,880195435	-0,420677962	1,62E-06	ITPR interacting domain con	ITPRID2
ENSG00000126882	3,084065019	4,137604664	3,713302498	-0,419790483	0,026102039	family with sequence simila	FAM78A
ENSG00000169902	35,38102304	47,35789918	6,9157415	-0,419719213	0,0056251	tyrosylprotein sulfotransfera	TPST1
ENSG00000032742	14,62063387	19,60043477	5,74007497	-0,419156819	5,78E-05	intraflagellar transport 88	IFT88
ENSG00000147050	42,17009132	54,75389721	6,852324712	-0,418570573	0,000281399	lysine demethylase 6A	KDM6A
ENSG00000078902	93,38692079	125,0369484	8,413742423	-0,418481467	2,40E-05	toll interacting protein	TOLLIP
ENSG00000128274	141,5365269	187,8169609	8,649997954	-0,418455341	0,021331471	alpha 1,4-galactosyltransfer	A4GALT
ENSG00000143315	18,13968137	24,18579905	5,989524897	-0,418442407	0,000158831	phosphatidylinositol glycan	PIGM
ENSG00000173812	214,6269089	287,018713	9,556970219	-0,417995941	1,36E-14	eukaryotic translation initiat	EIF1
ENSG00000147853	142,6169346	192,7935411	8,844822747	-0,417684125	0,021324805	adenylate kinase 3	AK3
ENSG00000187535	47,47192979	62,94191641	7,460265224	-0,417263289	0,004175389	intraflagellar transport 140	IFT140
ENSG00000159792	49,77396191	65,69137751	7,409395423	-0,417065019	4,74E-07	protein serine kinase H1	PSKH1
ENSG00000027001	14,04556174	18,7746184	5,525008748	-0,417035815	6,97E-05	mitochondrial intermediate	MIPEP
ENSG00000140396	66,80590259	89,20949036	7,651991754	-0,416847281	0,004314961	nuclear receptor coactivator	NCOA2
ENSG00000196792	54,23009216	71,19444503	7,359554662	-0,416490113	0,001089957	striatin 3	STRN3
ENSG00000054793	128,3256301	164,9651466	8,623040507	-0,416489892	0,022093275	ATPase phospholipid transp	ATP9A

ENSG00000260852	3,904836915	5,167591075	3,490295268	-0,41623695	0,023554979	FBXL19 antisense RNA 1	FBXL19-AS1
ENSG00000182199	109,3974279	146,2519189	8,534009541	-0,416082804	0,000154864	serine hydroxymethyltransfe	SHMT2
ENSG00000100364	150,7598852	201,244783	8,859564357	-0,415648117	4,21E-06	KIAA0930	KIAA0930
ENSG00000128294	42,035728	55,31599414	7,253682502	-0,414779705	0,000290422	tyrosylprotein sulfotransfera	TPST2
ENSG00000160226	7,901844909	10,44914188	4,988171827	-0,414721871	0,005586489	cilia and flagella associated	CFAP410
ENSG00000109458	34,41666206	46,07869597	6,703005379	-0,414455087	0,001442016	GRB2 associated binding pro	GAB1
ENSG00000215817	4,34604052	5,784965715	3,923154087	-0,414424678	0,013960458	zinc finger CCCH-type contai	ZC3H11B
ENSG00000188522	53,37433872	71,45048656	7,493218946	-0,413882362	0,001837146	family with sequence similar	FAM83G
ENSG00000005889	53,34415675	68,65408758	7,253433537	-0,413658553	0,000116708	zinc finger protein X-linked	ZFX
ENSG00000086544	30,84167872	40,90894812	6,6209414	-0,413460902	0,002334842	inositol-trisphosphate 3-kina	ITPKC
ENSG00000120306	46,36983553	61,98464132	7,224215232	-0,413381713	0,029974206	cysteine rich transmembran	CYSTM1
ENSG00000141013	30,01729429	39,99082123	6,525114068	-0,412979197	0,001080224	growth arrest specific 8	GAS8
ENSG00000182208	22,73886486	30,30698482	6,365533475	-0,412616514	3,09E-06	MOB kinase activator 2	MOB2
ENSG00000104763	83,93066252	111,7019885	8,324271661	-0,412579571	0,000162726	N-acylsphingosine amidohyd	ASAH1
ENSG00000170469	1,769453806	2,359479104	2,737645468	-0,41207872	0,034667784	spermatogenesis associated	SPATA24
ENSG00000113719	364,0089195	482,9039499	10,21564997	-0,411860104	1,58E-08	endoplasmic reticulum-golgi	ERGIC1
ENSG00000118965	25,23972296	33,65758791	6,457498517	-0,411247989	4,82E-05	WD repeat domain 35	WDR35
ENSG00000141425	48,83500936	65,07179494	7,29900294	-0,411022429	4,09E-08	regulation of nuclear pre-ml	RPRD1A
ENSG00000267100	4,995976434	6,634977264	4,167399733	-0,410989259	0,003891498	ILF3 divergent transcript	ILF3-DT
ENSG00000150401	13,55645449	17,95943037	5,465034906	-0,410636397	0,000792993	defective in cullin neddylatio	DCUN1D2
ENSG00000169093	34,50822311	45,95139099	7,022214658	-0,410349999	6,20E-08	acetylserotonin O-methyltra	ASMTL
ENSG00000181284	11,75998447	15,42113267	5,380368614	-0,41001989	0,010248341	transmembrane protein 102	TMEM102
ENSG00000179454	12,63155366	16,7450671	5,534154663	-0,409943786	0,000644493	kelch like family member 28	KLHL28
ENSG00000166575	14,32153975	19,07362565	5,654275039	-0,40993621	9,45E-05	transmembrane protein 135	TMEM135
ENSG00000226752	11,39388591	14,89821131	4,87577613	-0,40934418	0,02241728	cutA divalent cation toleranc	CUTALP
ENSG00000167671	98,33657503	129,043449	8,511671612	-0,409270326	9,14E-10	UBX domain protein 6	UBXN6
ENSG00000130396	167,172753	221,1253803	9,063715867	-0,40890694	0,001903763	afadin, adherens junction fo	AFDN
ENSG00000100568	73,23705769	97,03670477	8,03622523	-0,408756915	2,77E-09	vesicle transport through int	VTI1B
ENSG00000105656	30,04356698	39,94846904	6,652348284	-0,408440206	0,000175921	elongation factor for RNA po	ELL
ENSG00000185278	18,4562192	24,5076356	6,208702078	-0,40843132	0,001232536	zinc finger and BTB domain	ZBTB37
ENSG00000106771	192,3243737	256,0808883	9,424577299	-0,408348858	0,017707462	transmembrane protein 245	TMEM245
ENSG00000233016	19,76512243	26,24735784	6,326617868	-0,408270065	0,010423364	small nucleolar RNA host ge	SNHG7
ENSG00000049239	139,8686927	186,0112825	8,934787591	-0,407954102	0,011914023	hexose-6-phosphate dehydr	H6PD
ENSG00000164050	316,1757536	419,901233	10,12049344	-0,407645533	0,001659852	plexin B1	PLXNB1
ENSG00000185418	10,4814685	13,92752737	5,285610996	-0,406724176	0,000297463	threonyl-tRNA synthetase 3	TARS3
ENSG00000169016	9,467431705	12,55569823	5,034453345	-0,406548905	1,21E-05	E2F transcription factor 6	E2F6
ENSG00000184867	76,66967136	101,6129368	8,217657925	-0,406096591	0,000499488	armadillo repeat containing	ARMCX2
ENSG00000156239	11,7920956	15,65948016	5,356576864	-0,405901224	0,000251891	N-6 adenine-specific DNA m	N6AMT1
ENSG00000138439	22,60862535	29,52844493	6,191561303	-0,405492964	0,003354336	family with sequence similar	FAM117B
ENSG00000102409	51,75260574	68,05697019	7,604689833	-0,405419419	0,004441706	brain expressed X-linked 4	BEX4
ENSG00000120159	26,49088273	34,98530723	6,461328981	-0,405087256	0,000899288	caspase activity and apopto	CAAP1
ENSG00000196459	19,85683597	25,20661908	5,665502936	-0,404796306	0,000752141	trafficking protein particle c	TRAPPC2
ENSG00000100294	17,94646817	23,82725578	5,962475809	-0,404489364	2,46E-05	malonyl-CoA-acyl carrier pro	MCAT
ENSG00000172663	19,08948964	24,95042345	6,148685622	-0,404256366	3,07E-06	transmembrane protein 134	TMEM134
ENSG00000211456	31,08047937	41,16763247	6,646216544	-0,404155101	4,14E-05	SAC1 like phosphatidylinosit	SACM1L
ENSG00000123094	47,52377941	63,02401265	7,408628584	-0,403427754	1,73E-05	Ras association domain fami	RASSF8
ENSG00000132718	88,03863806	114,6240046	8,34402587	-0,403024774	4,72E-08	synaptotagmin 11	SYT11
ENSG00000072954	14,26025726	18,74209359	5,578174425	-0,4030177	0,04934157	transmembrane protein 38A	TMEM38A
ENSG00000186106	9,637174672	12,7441371	5,113370639	-0,402447523	0,000163347	ankyrin repeat domain 46	ANKRD46
ENSG00000175782	18,18619614	23,9992403	6,066300681	-0,402380459	0,004010807	solute carrier family 35 mem	SLC35E3
ENSG00000177192	20,2322848	26,91237744	6,09271218	-0,402317929	0,011688416	pseudouridine synthase 1	PUS1
ENSG00000106460	58,17566391	76,19206866	7,795506293	-0,402071089	0,025923396	transmembrane protein 106	TMEM106B
ENSG00000130313	66,07159418	86,96730022	7,869134939	-0,401961324	2,18E-05	6-phosphogluconolactonase	PGLS
ENSG00000174456	8,836118197	11,66841175	5,072092362	-0,401945433	0,002031801	chromosome 12 open readir	C12orf76
ENSG00000174276	10,10280948	13,36252724	5,168257112	-0,401833974	0,00172634	zinc finger HIT-type containi	ZNHIT2
ENSG00000110002	30,42234161	40,40369295	6,770591682	-0,401775221	0,032106025	von Willebrand factor A don	VWA5A
ENSG00000198952	174,9241265	231,3216259	9,158446449	-0,401664618	9,14E-10	SMG5 nonsense mediated n	SMG5
ENSG00000136560	31,76653445	41,77015402	6,686401877	-0,400955621	0,007365234	TRAF family member associa	TANK
ENSG00000135775	22,51844376	29,78665638	6,271315254	-0,400606038	0,003057795	component of oligomeric go	COG2
ENSG00000113758	304,3666034	391,9598559	9,982948834	-0,400543047	0,022200617	drebrin 1	DBN1
ENSG00000171425	31,08154665	41,13818734	6,95594193	-0,40054097	0,004375938	zinc finger protein 581	ZNF581
ENSG00000111412	81,77063302	107,784928	8,07513188	-0,400478998	3,26E-05	SREBF pathway regulator in	SPRING1
ENSG00000129158	7,854212272	10,2626085	4,761265961	-0,400425105	0,043209997	secretion regulating guanine	SERGEF
ENSG00000185238	13,80210336	18,17068659	5,471995862	-0,400243115	0,00136334	protein arginine methyltrans	PRMT3
ENSG00000181045	23,39240221	30,85999775	6,585395724	-0,40019084	0,005418239	solute carrier family 26 mem	SLC26A11
ENSG00000101350	81,30711563	107,5075483	8,116507408	-0,400136304	1,50E-05	kinesin family member 3B	KIF3B
ENSG00000174529	3,120078815	4,119896473	3,41199195	-0,399716712	0,017497106	transmembrane protein 81	TMEM81
ENSG00000155970	4,925688787	6,501800425	4,135909035	-0,399496371	0,017452789	mitochondrial calcium uptak	MICU3
ENSG00000197622	127,5456205	165,9452387	8,691208777	-0,399427421	0,000837432	CDC42 small effector 1	CDC42SE1
ENSG00000108187	6,006762219	7,685588024	4,277007637	-0,399381651	0,043104347	phenazine biosynthesis like	PBLD
ENSG00000170145	105,1987013	138,5454591	8,542939725	-0,399315924	0,000172292	salt inducible kinase 2	SIK2
ENSG00000277972	24,48586567	32,30204475	6,34487121	-0,399214419	0,006400635	CDGSH iron sulfur domain 3	CISD3
ENSG00000170502	41,092704	53,9919332	7,199051656	-0,398563457	0,012648365	nudix hydrolase 9	NUDT9
ENSG00000084731	74,50856661	97,03811314	7,903914087	-0,397819932	0,001905789	kinesin family member 3C	KIF3C
ENSG00000176101	39,20370029	51,69261966	7,122378445	-0,397071555	3,64E-06	SS nuclear autoantigen 1	SSNA1
ENSG00000085063	351,5538405	455,3101984	10,14715606	-0,397055821	0,01601298	CD59 molecule (CD59 blood	CD59
ENSG00000008394	37,75620493	48,11104808	6,855470847	-0,396988052	0,02070272	microsomal glutathione S-tr	MGST1
ENSG00000164323	39,48253854	51,91984767	6,987499863	-0,396826754	0,000306516	cilia and flagella associated	CFAP97
ENSG00000185621	19,74222617	26,04236708	6,114456419	-0,396389214	0,000256011	leishmanolysin like peptidas	LMLN
ENSG00000132405	97,44312864	128,0969635	8,308532569	-0,395790802	1,16E-09	TBC1 domain family membe	TBC1D14
ENSG00000177058	15,92272354	20,94876395	5,764444329	-0,395085243	0,000522893	solute carrier family 38 mem	SLC38A9
ENSG00000146833	34,36629736	45,16443376	7,031408403	-0,394847618	0,000586289	tripartite motif containing 4	TRIM4
ENSG00000197162	15,94149911	20,63291059	5,831801846	-0,394360177	0,01557049	zinc finger protein 785	ZNF785
ENSG00000113163	63,56239897	83,4442065	7,732496549	-0,394355402	0,000663106	ceramide transporter 1	CERT1
ENSG00000173065	73,90037734	97,0574062	8,110671898	-0,394297804	0,000313909	family with sequence similar	FAM222B
ENSG00000109466	42,88973534	56,46132545	7,09692442	-0,39420043	0,015352575	kelch like family member 2	KLHL2
ENSG00000166987	85,03342764	111,4932033	8,218622154	-0,394051571	2,14E-05	methyl-CpG binding domain	MBD6
ENSG00000132535	48,73215929	63,33670685	7,429468998	-0,393962453	0,004029627	discs large MAGUK scaffold	DLG4
ENSG00000123358	10,77079885	14,18151885	5,04502511	-0,393804467	0,04032275	nuclear receptor subfamily 4	NR4A1
ENSG00000125124	57,95546878	76,21110115	7,448810434	-0,393751055	5,79E-05	Bardet-Biedl syndrome 2	BBS2
ENSG00000057252	65,23961658	85,81513577	7,557229009	-0,392737088	0,011551305	sterol O-acyltransferase 1	SOAT1
ENSG00000118922	54,37472294	71,26838932	7,463526045	-0,392424483	0,032382945	Kruppel like factor 12	KLF12
ENSG00000204619	50,06704341	65,82715107	7,522248528	-0,392361361	2,65E-09	protein phosphatase 1 regul	PPP1R11
ENSG00000108406	60,79189564	79,92049462	7,737428698	-0,392198327	0,001187196	DEAH-box helicase 40	DHX40
ENSG00000198185	14,63116045	18,98135315	5,452932974	-0,392145519	0,030679438	zinc finger protein 334	ZNF334
ENSG00000186001	61,51642881	80,85731963	7,625190162	-0,391702397	1,82E-08	leucine rich repeats and calp	LRCH3
ENSG00000198369	65,88870699	86,14883549	7,758269083	-0,39163726	1,40E-06	sprouty related EVH1 domai	SPRED2
ENSG00000117305	50,24816238	66,05851809	7,456866513	-0,391626975	2,67E-07	3-hydroxy-3-methylglutaryl-	HMGCL
ENSG00000167468	192,1969331	252,7266983	9,297931659	-0,39088441	1,26E-06	glutathione peroxidase 4	GPX4
ENSG00000147586	4,72301144	6,199187317	4,114452674	-0,390836572	0,012241012	mitochondrial ribosomal pro	MRPS28
ENSG00000173917	62,19316118	81,41220973	7,71146812	-0,390297779	0,01167137	homeobox B2	HOXB2
ENSG00000082258	35,32228266	46,25138664	6,89171757	-0,390249474	0,00027349	cyclin T2	CCNT2
ENSG00000162688	33,8112184	44,43769236	6,829545876	-0,390012484	4,53E-05	amylo-alpha-1, 6-glucosidas	AGL
ENSG00000102805	24,32844405	31,58831762	6,474660154	-0,389890804	0,045159107	CLN5 intracellular trafficking	CLN5
ENSG00000073711	58,84933946	77,11783271	7,776492795	-0,389432012	0,010647997	protein phosphatase 2 regul	PPP2R3A
ENSG00000116918	14,94563237	19,59101817	5,702446402	-0,389423519	0,000896029	translin associated factor X	TSNAX
ENSG00000172086	33,7181168	44,18237524	6,72033311	-0,38909041	0,004835313	lysine rich coiled-coil 1	KRCC1
ENSG00000012232	133,1187425	170,5794195	8,726601306	-0,38893874	0,007867533	exostosin like glycosyltransf	EXTL3
ENSG00000227500	147,6075841	193,5514801	8,882391629	-0,388921059	2,18E-12	secretory carrier membrane	SCAMP4
ENSG00000158006	19,56172993	25,63675474	6,187390001	-0,388702452	1,33E-05	platelet activating factor ace	PAFAH2
ENSG00000249348	2,683497483	3,502308084	3,623925865	-0,388198395	0,020169635	UGDH antisense RNA 1	UGDH-AS1
ENSG00000100330	80,45941998	104,1257689	8,048988273	-0,388197519	0,003572693	myotubularin related protei	MTMR3
ENSG00000081791	46,89951379	61,28909237	7,338109638	-0,387429893	0,005673492	DAP3 binding cell death enh	DELE1
ENSG00000115421	23,58118815	30,90108369	6,293331617	-0,387174591	2,76E-08	poly(A) polymerase gamma	PAPOLG
ENSG00000089289	44,00140252	57,35892207	7,400498232	-0,386611739	0,007068426	immunoglobulin binding pro	IGBP1

ENSG00000171490	95,16941989	124,5740196	8,355303816	-0,386276849	7,10E-07	ribosomal L1 domain contai	RSL1D1
ENSG00000168291	51,98431093	68,16524387	7,502605979	-0,385990348	0,000729946	pyruvate dehydrogenase E1	PDHB
ENSG00000183718	11,53792097	15,0946674	5,489766171	-0,385786054	0,012433099	tripartite motif containing 5	TRIM52
ENSG00000108799	34,70853588	45,51856691	7,063975803	-0,385769564	0,002644321	enhancer of zeste 1 polycon	EZH1
ENSG00000278619	11,00645922	14,40767586	5,328007072	-0,385678318	0,03031737	mitochondrial rRNA methylt	MRM1
ENSG00000171448	9,190157326	11,81124127	5,06217987	-0,385607816	0,007523753	zinc finger and BTB domain	ZBTB26
ENSG00000188643	283,4321376	369,1303485	9,816880436	-0,385467235	0,000974102	S100 calcium binding protei	S100A16
ENSG00000139197	34,81539074	45,56073753	7,036645964	-0,385377693	5,23E-13	peroxisomal biogenesis fact	PEX5
ENSG00000167930	83,61410162	109,0946911	8,287449296	-0,385266902	0,005657436	family with sequence simila	FAM234A
ENSG00000272142	3,381180883	4,427370023	3,736439126	-0,385013971	0,042248013	LYRM4 antisense RNA 1	LYRM4-AS1
ENSG00000198858	52,95360022	69,09870873	7,43494035	-0,384788482	8,94E-09	R3H domain containing 4	R3HDM4
ENSG00000134874	113,8619641	147,9980767	8,457133593	-0,384042623	0,030658854	DAZ interacting zinc finger p	DZIP1
ENSG00000132004	11,16895334	14,59508578	5,334936858	-0,383676312	0,000411452	F-box and WD repeat domai	FBXW9
ENSG00000052749	34,2456015	44,84883965	6,866350896	-0,383223835	0,040532587	ribosomal RNA processing 1	RRP12
ENSG00000165322	32,71187272	42,74524556	6,931996796	-0,383049875	9,43E-05	Rho GTPase activating prote	ARHGAP12
ENSG00000181754	11,18103625	14,59432536	4,915102016	-0,382587684	0,000622442	adhesion molecule with Ig li	AMIGO1
ENSG00000010278	168,5356492	221,1072204	9,106026171	-0,382440141	0,014226128	CD9 molecule	CD9
ENSG00000010810	60,01041473	78,95873543	7,778135054	-0,382030003	0,018560376	FYN proto-oncogene, Src far	FYN
ENSG00000197586	100,4138907	131,1629129	8,416318003	-0,381834301	1,74E-06	ectonucleoside triphosphate	ENTPD6
ENSG00000131437	19,259151	25,17270613	6,103168001	-0,381543894	0,000393279	kinesin family member 3A	KIF3A
ENSG00000149260	43,53862589	56,80671163	7,049235615	-0,380844209	0,000599889	calpain 5	CAPN5
ENSG00000244405	40,85480253	53,11140808	6,904852048	-0,380270026	0,010591182	ETS variant transcription fac	ETV5
ENSG00000168758	43,68615878	56,68564825	7,255849452	-0,379939878	9,79E-06	semaphorin 4C	SEMA4C
ENSG00000158711	88,48786281	114,8348069	8,149056949	-0,37989291	0,00063033	ETS transcription factor ELK	ELK4
ENSG00000036549	61,26180489	79,77390592	7,741492324	-0,379597712	7,84E-06	zinc finger ZZ-type containin	ZZZ3
ENSG00000119782	7,263763866	9,409289049	4,486142106	-0,379518377	0,001716076	FKBP prolyl isomerase 1B	FKBP1B
ENSG00000132334	52,04927245	67,54032319	7,125561309	-0,379424594	0,01641311	protein tyrosine phosphatas	PTPRE
ENSG00000228794	12,07777775	15,51912664	5,093199138	-0,378657782	0,001410816	long intergenic non-protein	LINC01128
ENSG00000151553	28,40079177	36,83438948	6,693636515	-0,3785624	0,00087133	FHF complex subunit HOOK	FHIP2A
ENSG00000219545	13,67221547	17,82027034	5,412580959	-0,378079068	0,020027944	UBAP1-MVB12-associated (I	UMAD1
ENSG00000119655	122,5862617	159,5727088	8,689991442	-0,377937597	3,16E-05	NPC intracellular cholestero	NPC2
ENSG00000143409	48,6925684	61,54992059	7,354837929	-0,376518918	0,049587633	MINDY lysine 48 deubiquitir	MINDY1
ENSG00000141101	49,80561585	64,83515381	7,584958363	-0,376500069	0,000116937	NIN1 (RPN12) binding prote	NOB1
ENSG00000168826	4,270440485	5,548694318	3,877385772	-0,376323814	0,005933717	zinc finger and BTB domain	ZBTB49
ENSG00000178996	40,42748132	52,02653388	7,178690365	-0,376243619	5,00E-05	sorting nexin 18	SNX18
ENSG00000072134	68,77293139	88,73166897	7,87687859	-0,376217464	4,95E-10	epsin 2	EPN2
ENSG00000169217	98,16122326	126,9544616	8,42431723	-0,376154053	0,000123291	CD2 cytoplasmic tail binding	CD2BP2
ENSG00000114125	30,9212486	39,84851637	6,748324385	-0,376106948	1,91E-07	ring finger protein 7	RNF7
ENSG00000198113	145,2106428	188,5370094	8,867575662	-0,375619762	2,89E-08	torsin family 4 member A	TOR4A
ENSG00000106665	163,0871839	211,7330683	9,159570199	-0,374980423	0,001170333	CAP-Gly domain containing	CLIP2
ENSG00000119912	49,46347138	63,5677449	7,133337553	-0,374687906	0,000112953	insulin degrading enzyme	IDE
ENSG00000100379	45,75662909	59,32111141	7,368994369	-0,374649651	0,000481781	potassium channel tetramer	KCTD17
ENSG00000181873	16,21620873	20,99384489	5,772731866	-0,374093111	2,91E-05	iron-sulfur cluster assembly	IBA57
ENSG00000127947	118,6193184	154,3512215	8,592884087	-0,373495222	0,000825041	protein tyrosine phosphatas	PTPN12
ENSG00000103126	69,89806988	90,61289623	7,813615259	-0,373262416	0,000414456	axin 1	AXIN1
ENSG00000125375	7,948268365	10,31307197	4,83055038	-0,373259773	0,012017374	distal membrane arm assem	DMAC2L
ENSG00000135241	46,18614522	59,93320291	7,283217378	-0,373209916	6,62E-08	patatin like phospholipase d	PNPLA8
ENSG00000189339	83,50580972	106,8611233	8,262837422	-0,373200555	0,000244923	solute carrier family 35 men	SLC35E2B
ENSG00000121671	22,19089827	28,6962407	6,353661029	-0,373017635	0,005579696	cryptochrome circadian regu	CRY2
ENSG00000174132	13,05715927	16,84065281	5,625448032	-0,372957126	2,50E-05	family with sequence simila	FAM174A
ENSG00000133112	931,7907737	1200,002452	11,6478927	-0,372940462	0,014051947	tumor protein, translational	TPT1
ENSG00000137145	70,5833615	91,6038001	7,863193202	-0,372476471	0,000268829	DENN domain containing 4C	DENND4C
ENSG00000156345	6,706374564	8,700599606	4,805676472	-0,372034022	0,030679438	cyclin dependent kinase 20	CDK20
ENSG00000136877	62,24012183	80,16640837	7,568939096	-0,371857888	4,34E-06	folylpolyglutamate synthase	FPGS
ENSG00000162851	8,717729115	11,30170651	4,87206071	-0,371815238	0,018840397	transcription factor B2, mitc	TFB2M
ENSG00000075399	27,35586038	35,50915704	6,809079487	-0,371687956	1,94E-05	VPS9 domain containing 1	VPS9D1
ENSG00000028203	64,29922857	83,18332111	7,810202898	-0,371602679	1,56E-07	vezatin, adherens junctions	VEZT
ENSG00000107669	27,87503275	36,15704171	6,461303741	-0,37147623	0,001206637	arginyltransferase 1	ATE1
ENSG00000174238	67,32649243	87,18158016	7,750550768	-0,371282202	0,000108343	phosphatidylinositol transfe	PITPNA
ENSG00000148943	34,87880031	45,14815846	6,743027638	-0,370976578	0,000619537	lin-7 homolog C, crumbs cell	LIN7C
ENSG00000078487	3,833407164	4,976888456	3,872237636	-0,370860632	0,007416257	zinc finger CW-type and PW	ZCWPW1
ENSG00000106246	7,172244812	9,287968296	4,506714771	-0,37083371	0,000236739	pentatricopeptide repeat dc	PTCD1
ENSG00000023734	112,7747529	145,2793427	8,479110463	-0,370180789	3,73E-11	serine/threonine kinase rec	STRAP
ENSG00000163444	48,11988595	62,29616415	7,404559523	-0,370159555	6,57E-07	transmembrane protein 183	TMEM183A
ENSG00000187097	20,19470634	26,19722582	6,196693651	-0,370145892	0,003236222	ectonucleoside triphosphate	ENTPD5
ENSG00000119138	133,271281	173,0336588	8,891581248	-0,369798856	0,027176393	Kruppel like factor 9	KLF9
ENSG00000170903	33,69635949	43,55411717	6,858434838	-0,369329693	2,34E-07	Myb/SANT DNA binding dor	MSANTD4
ENSG00000174749	15,04010408	19,48514892	5,258738812	-0,369233387	0,013304947	family with sequence simila	FAM241A
ENSG00000128590	20,77077848	26,6940092	6,370474079	-0,369168793	0,013342659	DnaJ heat shock protein fam	DNAJB9
ENSG00000111144	102,3571686	132,4325202	8,488597947	-0,369046126	1,01E-11	leukotriene A4 hydrolase	LTA4H
ENSG00000145868	45,24225638	58,31812046	7,27685674	-0,369043884	4,20E-08	F-box protein 38	FBXO38
ENSG00000178904	30,99674868	40,10566795	6,759592172	-0,368742998	0,012001105	dpy-19 like C-mannosyltrans	DPY19L3
ENSG00000188786	36,83700974	47,66519473	7,069033732	-0,368490364	0,001638769	metal regulatory transcripti	MTF1
ENSG00000117500	93,09435962	120,1631996	8,278568144	-0,368244088	0,00500026	transmembrane p24 traffick	TMED5
ENSG00000196151	10,04178933	12,96754959	5,003043668	-0,367967866	0,008517521	WD repeat, sterile alpha mo	WDSUB1
ENSG00000059758	61,33722131	79,61215358	7,645266907	-0,367915325	0,001306258	cyclin dependent kinase 17	CDK17
ENSG00000124486	206,0719594	265,2596593	9,428094293	-0,36789601	0,000128182	ubiquitin specific peptidase	USP9X
ENSG00000070444	57,05924206	73,65302759	7,568210966	-0,367809907	4,68E-05	MAX network transcriptiona	MNT
ENSG00000204186	20,05643152	25,66207264	6,108980023	-0,367060052	0,00240505	zinc finger DBF-type contain	ZDBF2
ENSG00000170088	44,78649255	57,88238937	7,324290457	-0,366559767	1,59E-05	transmembrane protein 192	TMEM192
ENSG00000071243	7,951203351	10,24846484	4,672505465	-0,366073895	0,000240247	inhibitor of growth family m	ING3
ENSG00000165689	46,46880602	60,01633901	7,26541345	-0,365976882	7,25E-06	endosome associated traffic	ENTR1
ENSG00000138463	18,99909287	24,45881768	6,028156606	-0,365767599	0,023182219	solute carrier family 49 men	SLC49A4
ENSG00000176783	58,5298189	75,62642703	7,729024275	-0,364675733	8,33E-05	RUN and FYVE domain conta	RUFY1
ENSG00000137876	41,33489545	53,21556398	7,180303867	-0,363667986	0,000232174	ribosomal L24 domain conta	RSL24D1
ENSG00000174165	21,27752264	27,35223342	6,255327398	-0,3632782	0,001864154	zinc finger DHHC-type conta	ZDHHC24
ENSG00000010404	75,2022202	96,15514891	7,966666102	-0,363276775	0,00057185	iduronate 2-sulfatase	IDS
ENSG00000006715	94,95791965	120,9109039	8,277438608	-0,362951101	5,65E-05	VPS41 subunit of HOPS com	VPS41
ENSG00000255112	66,07986496	84,81105227	7,885753593	-0,362908806	0,000799471	charged multivesicular body	CHMP1B
ENSG00000144455	49,73338124	63,91617629	7,447136426	-0,36271875	0,017214829	sulfatase modifying factor 1	SUMF1
ENSG00000155097	64,93394808	83,65601798	7,751304675	-0,362249682	9,47E-08	ATPase H+ transporting V1 s	ATP6V1C1
ENSG00000196914	418,5386884	538,9613292	10,39493255	-0,362102345	0,000378907	Rho guanine nucleotide excl	ARHGEF12
ENSG00000154813	28,43417329	36,5825806	6,669189281	-0,361781626	1,28E-05	diphthamide biosynthesis 3	DPH3
ENSG00000165775	35,0015788	44,95809003	7,019040326	-0,36176557	0,017462737	FUN14 domain containing 2	FUNDC2
ENSG00000177125	30,12605005	38,66287832	6,546233237	-0,361430017	0,000146559	zinc finger and BTB domain	ZBTB34
ENSG00000177082	23,44241173	30,20958337	6,441573983	-0,361054845	0,017494379	WD repeat domain 73	WDR73
ENSG00000091136	668,4028205	859,3516942	11,17433022	-0,360885973	0,000386811	laminin subunit beta 1	LAMB1
ENSG00000163686	14,30670354	18,37900128	5,575684748	-0,360476101	0,032348721	abhydrolase domain contair	ABHD6
ENSG00000157600	58,29356016	74,84702211	7,556056423	-0,36036504	4,78E-05	transmembrane protein 164	TMEM164
ENSG00000166454	75,96508166	97,4594515	7,991202405	-0,360000523	5,50E-11	ATM interactor	ATMIN
ENSG00000109065	27,03997147	34,73046603	6,773202891	-0,35994212	0,003228221	N-acetyltransferase 9 (putat	NAT9
ENSG00000248508	3,471045876	4,453863192	3,949023618	-0,3597893	0,024044213	SRP14 divergent transcript	SRP14-DT
ENSG00000164068	58,02958807	74,59063028	7,733574587	-0,359674319	0,000240247	ring finger protein 123	RNF123
ENSG00000111321	190,6668596	245,1747275	9,464768678	-0,359490758	1,44E-06	lymphotoxin beta receptor	LTBR
ENSG00000103502	98,78160724	126,9277828	8,514373176	-0,359297628	1,23E-07	CDP-diacylglycerol--inositol	CDIPT
ENSG00000048162	13,2315972	17,03462988	5,600127985	-0,359261796	0,028432292	NOP16 nucleolar protein	NOP16
ENSG00000122203	142,4792251	181,7999875	8,912423801	-0,358957172	4,08E-12	KIAA1191	KIAA1191
ENSG00000134352	406,9561474	520,2233013	10,31810351	-0,358652932	0,007029049	interleukin 6 cytokine family	IL6ST
ENSG00000130821	76,96582964	98,11932024	8,277996985	-0,358471918	0,003809405	solute carrier family 6 meml	SLC6A8
ENSG00000112378	141,7296559	179,6685586	8,715068453	-0,358215022	0,000324206	p53 apoptosis effector relat	PERP
ENSG00000163956	191,2611475	243,5753543	9,313402765	-0,358200151	0,000267214	LDL receptor related proteir	LRPAP1
ENSG00000174227	54,22063972	69,58653591	7,532275537	-0,357627628	4,29E-05	phosphatidylinositol glycan	PIGG
ENSG00000100271	13,23539933	16,89276669	5,621482584	-0,357515867	0,002629329	TTL family tubulin polyglutai	TTLL1
ENSG00000122335	14,86655329	19,01792498	5,873806675	-0,357364782	0,000271702	serine active site containing	SERAC1
ENSG00000137207	115,4583175	147,3527466	8,686304192	-0,35716823	1,70E-05	Yip1 domain family member	YIPF3

ENSG00000147457	54,56349033	70,02700557	7,545037594	-0,356883483	3,09E-07	charged multivesicular body	CHMP7
ENSG00000123739	35,29326952	45,35556639	6,930388406	-0,356875789	0,003979019	phospholipase A2 group XIIA	PLA2G12A
ENSG00000106105	106,030194	135,955275	8,582046286	-0,356741474	3,90E-06	glycyl-tRNA synthetase 1	GARS1
ENSG00000114503	75,81059921	96,37911596	7,982948647	-0,356615357	6,15E-05	nuclear cap binding protein	NCBP2
ENSG00000255529	16,90588939	21,69529248	5,890322762	-0,35659433	0,005849639	RNA polymerase II subunit M	POLR2M
ENSG00000214756	3,085315578	3,96203104	3,616990795	-0,356452303	0,021487591	citrate synthase lysine meth	CSKMT
ENSG00000034713	46,26417786	59,29283998	7,366263884	-0,356401313	6,98E-05	GABA type A receptor assoc	GABARAPL2
ENSG00000170242	74,1272859	94,47466699	7,95306049	-0,356106915	8,22E-05	ubiquitin specific peptidase	USP47
ENSG00000158156	14,07221402	18,0408192	5,643072331	-0,355859088	0,000975006	XK related 8	XKR8
ENSG00000186312	18,42588894	23,22135182	5,711447489	-0,355611321	0,000209054	carbonic anhydrase 5B pseu	CA5BP1
ENSG00000110046	85,35384974	109,2570305	8,236845381	-0,355542102	0,000282321	autophagy related 2A	ATG2A
ENSG00000168310	39,85241924	51,09236672	7,031418715	-0,355524309	0,007054064	interferon regulatory factor	IRF2
ENSG00000131507	108,181159	138,5149894	8,52723794	-0,355371274	0,000746399	Nedd4 family interacting pro	NDFIP1
ENSG00000113851	22,57448813	28,91131424	6,398469657	-0,355227983	0,000204699	cereblon	CRBN
ENSG00000108509	90,65407572	116,0361389	8,269344799	-0,354777201	4,73E-07	calmodulin binding transcrip	CAMTA2
ENSG00000224597	35,39640373	44,88377293	6,634946734	-0,354654495	0,000676327	SVIL antisense RNA 1	SVIL-AS1
ENSG00000132434	13,66969917	17,43998788	5,521843936	-0,354405989	0,017162174	LanC like 2	LANCL2
ENSG00000006451	84,54297983	108,1372322	8,041949564	-0,35437943	0,000169612	RAS like proto-oncogene A	RALA
ENSG00000229809	8,426690938	10,73736258	4,905690354	-0,354291264	0,010735631	zinc finger protein 688	ZNF688
ENSG00000135317	58,84760732	75,3313805	7,548309551	-0,354156933	9,99E-07	sorting nexin 14	SNX14
ENSG00000095209	14,94425231	19,1292746	5,578011461	-0,354058004	0,001564605	transmembrane protein 38B	TMEM38B
ENSG00000113407	124,1065329	159,0595212	8,684981064	-0,35367495	0,000182074	threonyl-tRNA synthetase 1	TARS1
ENSG00000196639	77,50177867	94,26483057	7,888499586	-0,35344635	0,038916036	histamine receptor H1	HRH1
ENSG00000157823	19,21240154	24,55756833	6,179725328	-0,353324854	0,000443984	adaptor related protein com	AP3S2
ENSG00000101493	44,1889293	55,47042776	7,386832388	-0,353242092	0,019183019	zinc finger protein 516	ZNF516
ENSG00000183255	358,8972238	456,6283488	10,3260453	-0,353052294	0,015087786	PTTG1 interacting protein	PTTG1IP
ENSG00000170175	19,78541718	24,86865554	6,065459405	-0,352544034	0,008586745	cholinergic receptor nicotini	CHRNB1
ENSG00000117139	126,2009328	156,0673898	8,886159812	-0,352386821	0,004726544	lysine demethylase 5B	KDM5B
ENSG00000107815	20,811587	26,6355663	6,23438939	-0,352069469	0,003815356	twinkle mtDNA helicase	TWNK
ENSG00000138709	19,30259502	24,73026308	5,852446913	-0,351910885	0,020360489	La ribonucleoprotein 1B	LARP1B
ENSG00000186448	28,82621785	36,68306401	6,723872164	-0,351712005	0,00030241	zinc finger protein 197	ZNF197
ENSG00000104320	59,36331347	75,84725473	7,499409316	-0,351574405	0,032087804	nibrin	NBN
ENSG00000175414	42,98828033	53,91401987	7,332146068	-0,351511254	0,005383909	ADP ribosylation factor like (	ARL10
ENSG00000130147	288,3699518	362,2909809	10,11677177	-0,351469047	0,000999253	SH3 domain binding protein	SH3BP4
ENSG00000179632	118,8959207	152,0030319	8,683321083	-0,351312604	4,94E-07	MAF1 homolog, negative re	MAF1
ENSG00000136718	40,73392762	52,07827959	7,176124916	-0,351303625	0,001835661	IMP U3 small nucleolar ribo	IMP4
ENSG00000183726	97,95255312	124,9024198	8,263886461	-0,350810354	7,65E-05	transmembrane protein 50A	TMEM50A
ENSG00000274020	3,502610385	4,418916425	3,698070726	-0,35069919	0,031293747	long intergenic non-protein	LINC01138
ENSG00000116977	91,44595183	116,9597234	8,337595693	-0,350580373	0,005914771	galectin 8	LGALS8
ENSG00000059769	8,410414251	10,72894398	4,723641523	-0,350379524	0,00109819	DnaJ heat shock protein fam	DNAJC25
ENSG00000189369	17,90923761	22,84285794	5,95795752	-0,350042825	0,00050994	G1 to S phase transition 2	GSPT2
ENSG00000224870	22,85385009	29,06798768	6,338803195	-0,349901838	0,01143273	MRPL20 antisense RNA 1	MRPL20-AS1
ENSG00000100418	57,4228778	72,71102335	7,55678946	-0,34982654	3,41E-05	desumoylating isopeptidase	DESI1
ENSG00000104613	38,01415895	48,57344553	6,996265425	-0,349724223	3,10E-05	integrator complex subunit	INTS10
ENSG00000133318	181,5984972	231,5427993	9,110459079	-0,349344235	0,00079811	reticulon 3	RTN3
ENSG00000186501	32,00927544	40,62078625	6,585942205	-0,348457475	0,000739656	transmembrane protein 222	TMEM222
ENSG00000204271	9,273020985	11,81406435	5,030956875	-0,348277197	0,024334865	spindlin family member 3	SPIN3
ENSG00000160948	41,11413616	51,86549545	7,244090525	-0,34814001	5,38E-05	VPS28 subunit of ESCRT-I	VPS28
ENSG00000164414	9,141374034	11,66186515	4,884847091	-0,348108319	0,007701679	solute carrier family 35 mem	SLC35A1
ENSG00000149231	43,15519208	55,0522354	7,046814999	-0,348104093	1,00E-05	coiled-coil domain containin	CCDC82
ENSG00000197324	515,4493305	655,2972333	10,81450324	-0,347953756	4,74E-07	LDL receptor related protein	LRP10
ENSG00000154743	7,745324745	9,875998717	4,811537475	-0,347802939	0,006997299	tRNA splicing endonuclease	TSEN2
ENSG00000124151	62,0606639	79,13261427	7,725533834	-0,347770068	0,00058459	nuclear receptor coactivator	NCOA3
ENSG00000189180	53,11493852	67,36061755	7,569044618	-0,347334122	0,014752738	zinc finger protein 33A	ZNF33A
ENSG00000163513	529,9656356	652,3374135	10,61159848	-0,347126587	0,001054265	transforming growth factor	TGFBR2
ENSG00000129925	143,946628	182,3648585	8,860474573	-0,347082554	0,001018977	post-GPI attachment to prot	PGAP6
ENSG00000151093	7,415490899	9,414593704	4,703882556	-0,346537471	0,01263669	3-oxoacyl-ACP synthase, mit	OXSM
ENSG00000136932	7,335167503	9,354085094	4,780579589	-0,346517805	0,034731301	tRNA methyltransferase O	TRMO
ENSG00000150990	42,48185917	54,12461737	7,186141877	-0,346220138	0,001087469	DEAH-box helicase 37	DHX37
ENSG00000151176	170,4340667	215,6882611	9,293821865	-0,345938886	4,43E-05	phospholipase B domain con	PLBD2
ENSG00000089060	43,84520112	55,96783781	7,241103482	-0,345625355	2,31E-05	solute carrier family 8 meml	SLC8B1
ENSG00000033100	94,89225003	117,8428492	8,308067565	-0,345599706	0,000167081	chondroitin polymerizing fac	CHPF2
ENSG00000143162	35,4573921	45,16837585	7,149938142	-0,345584256	0,001179119	cellular repressor of E1A stir	CREG1
ENSG00000171365	9,276831104	11,81988249	4,982527074	-0,345584231	0,008733241	chloride voltage-gated chan	CLCN5
ENSG00000168397	78,15471866	99,60788573	8,066642794	-0,345583103	0,005673492	autophagy related 4B cystei	ATG4B
ENSG00000148175	153,8740843	195,8301983	8,62544157	-0,345346976	0,016122315	stomatin	STOM
ENSG00000187531	30,57219675	39,01738465	6,762125553	-0,345121717	0,017776195	sirtuin 7	SIRT7
ENSG00000103264	45,76072393	58,23860846	7,340109778	-0,345085018	2,30E-08	F-box protein 31	FBXO31
ENSG00000066827	16,68616723	21,20551187	5,901472945	-0,344444764	0,000565813	zinc finger and AT-hook dom	ZFAT
ENSG00000092531	53,45864009	67,64808115	7,426029817	-0,343969468	3,85E-05	synaptosome associated pro	SNAP23
ENSG00000085719	85,16992202	108,2378761	8,200299718	-0,343715351	9,90E-05	copine 3	CPNE3
ENSG00000157954	103,9402758	132,1477253	8,475499431	-0,343676351	3,60E-05	WD repeat domain, phospho	WIPI2
ENSG00000198034	441,6038872	549,1194442	10,44406327	-0,343526062	0,014425153	ribosomal protein S4 X-linke	RPS4X
ENSG00000112357	6,54613203	8,323944517	4,522820498	-0,343499385	0,014727222	peroxisomal biogenesis facto	PEX7
ENSG00000026508	590,4849312	751,1830234	10,76038107	-0,34348382	0,038549774	CD44 molecule (Indian blood	CD44
ENSG00000165527	216,8399799	276,4184125	9,458092864	-0,343200759	0,001629674	ADP ribosylation factor 6	ARF6
ENSG00000148572	22,67229898	28,73434529	6,274982115	-0,342957534	0,000386794	nuclear receptor binding fac	NRBF2
ENSG00000135365	53,9188102	67,81946873	7,546765415	-0,341974028	1,22E-06	PHD finger protein 21A	PHF21A
ENSG00000225190	66,99612898	85,15674044	7,978602412	-0,341960414	0,000595739	pleckstrin homology and RU	PLEKHM1
ENSG00000123505	73,49669267	93,40925335	7,735833428	-0,341480885	0,000945752	adenosylmethionine decarb	AMD1
ENSG00000050820	237,930731	302,3429564	9,69538184	-0,34133971	0,00134607	BCAR1 scaffold protein, Cas	BCAR1
ENSG00000121749	41,47191688	52,50277527	7,062664717	-0,340867695	0,00493244	TBC1 domain family membe	TBC1D15
ENSG00000066739	44,77327861	56,74230561	7,307747423	-0,340525686	0,00033705	autophagy related 2B	ATG2B
ENSG00000234009	5,032688911	6,357098912	4,140402264	-0,340442839	0,021864746	ribosomal protein L5 pseudo	RPL5P34
ENSG00000175893	20,58204718	26,12732673	6,076775213	-0,340250242	0,030658854	zinc finger DHHC-type palmi	ZDHHC21
ENSG00000118197	15,1488883	19,19930709	5,497409562	-0,339588519	0,000315602	DEAD-box helicase 59	DDX59
ENSG00000167733	10,22760216	12,91974723	5,437150567	-0,339555465	0,044626063	hydroxysteroid 11-beta deh	HSD11B1L
ENSG00000168917	16,02387075	20,19049545	5,43868446	-0,339338028	0,024313538	solute carrier family 35 mem	SLC35G2
ENSG00000110442	33,26066246	41,85704372	7,016237092	-0,339191602	0,015281691	COMM domain containing 9	COMMD9
ENSG00000099949	103,9571139	131,9445999	8,605101458	-0,339035507	0,005451231	leucine zipper like transcript	LZTR1
ENSG00000260032	334,1872521	420,3483927	10,18803784	-0,338558682	0,00036815	non-coding RNA activated b	NORAD
ENSG00000134759	65,48693505	82,99049763	7,924605884	-0,338315825	6,16E-06	elongator acetyltransferase	ELP2
ENSG00000100522	49,69324452	62,90160083	7,168535616	-0,337559061	0,004001791	glucosamine-phosphate N-a	GNPNAT1
ENSG00000144959	81,33051194	103,0140781	7,98112457	-0,337434443	9,46E-05	neutral cholesterol ester hyd	NCEH1
ENSG00000181396	52,22848743	65,87651076	7,378499511	-0,337203667	3,59E-05	2-oxoglutarate and iron dep	OGFOD3
ENSG00000117597	25,74217845	32,5591487	6,471982834	-0,337164288	0,001134205	UTP25 small subunit proces	UTP25
ENSG00000114302	92,06929891	116,2057988	8,270644689	-0,337100746	0,006240601	protein kinase cAMP-depen	PRKAR2A
ENSG00000177189	63,104306	79,59304436	7,598469708	-0,336901341	0,032292339	ribosomal protein S6 kinase	RPS6KA3
ENSG00000166548	39,38165363	49,08942601	7,041194952	-0,33664935	0,022768082	thymidine kinase 2	TK2
ENSG00000214160	31,05121037	39,25899855	6,830880034	-0,335863623	0,000165788	ALG3 alpha-1,3- mannosyltr	ALG3
ENSG00000151729	51,51047624	65,17267715	7,378868325	-0,335758268	3,35E-06	solute carrier family 25 mem	SLC25A4
ENSG00000165209	24,19091463	30,58693399	6,255576439	-0,335604218	0,001632978	spermatid perinuclear RNA b	STRBP
ENSG00000117614	39,49629651	49,7652544	7,143325511	-0,335389165	0,005418239	SYF2 pre-mRNA splicing fact	SYF2
ENSG00000253719	96,31763484	120,3217341	8,374627507	-0,335374898	0,000475908	ataxin 7 like 3B	ATXN7L3B
ENSG00000088854	40,15185071	50,59698246	7,063606131	-0,335276929	0,000113906	dynein axonemal assembly f	DNAAF9
ENSG00000213015	40,05970313	49,7132496	7,098517923	-0,335086604	0,000743119	zinc finger protein 580	ZNF580
ENSG00000075240	63,16818752	79,41805538	7,709184139	-0,33490315	0,002024857	GRAM domain containing 4	GRAMD4
ENSG00000179361	9,335963391	11,78948225	4,937095528	-0,334833754	0,013486533	AT-rich interaction domain 3	ARID3B
ENSG00000155760	58,09124483	70,2987625	7,31823819	-0,334605434	0,032715415	frizzled class receptor 7	FZD7
ENSG00000205356	50,52240679	63,87279772	7,608058452	-0,33448262	0,001108677	tectonin beta-propeller repe	TECPR1
ENSG00000173153	40,43221996	50,96751465	7,130425349	-0,334332882	0,000899371	estrogen related receptor al	ESRRA
ENSG00000151292	47,87878182	60,34164243	7,330322105	-0,334275804	0,00015265	casein kinase 1 gamma 3	CSNK1G3
ENSG00000101966	74,63817008	93,94867338	7,959121398	-0,33405108	0,002087029	X-linked inhibitor of apoptos	XIAP
ENSG00000006607	50,86907485	63,88002637	7,294530108	-0,333996502	0,028782774	FERM, ARH/RhoGEF and ple	FARP2
ENSG00000204256	276,5173594	349,2048851	9,818458581	-0,333935316	1,81E-11	bromodomain containing 2	BRD2

ENSG00000261609	22,23873895	28,09365835	6,301808374	-0,333908831	0,028782774	gigaxonin	GAN
ENSG00000170113	32,16304141	40,59784898	6,604863608	-0,333594405	0,005731296	NIPA magnesium transporte	NIPA1
ENSG00000157191	83,28147525	104,7506915	8,14957272	-0,33334612	3,71E-06	NECAP endocytosis associat	NECAP2
ENSG00000080189	51,21687731	64,45201882	7,534657308	-0,33311889	7,07E-09	solute carrier family 35 men	SLC35C2
ENSG00000169905	92,15284174	116,2379206	8,292986259	-0,332868208	6,19E-05	torsin 1A interacting protein	TOR1AIP2
ENSG00000110367	215,766415	271,4236426	9,532440655	-0,332456411	0,004267759	DEAD-box helicase 6	DDX6
ENSG00000114520	29,20658311	36,86464011	6,561502394	-0,332420712	0,005647626	sorting nexin 4	SNX4
ENSG00000240857	16,49920126	20,81808844	5,775177377	-0,332284518	0,000296647	retinol dehydrogenase 14	RDH14
ENSG00000164889	202,6824622	255,0481175	9,515523854	-0,331897316	4,45E-09	solute carrier family 4 meml	SLC4A2
ENSG00000146587	26,96858554	33,95575917	6,627203045	-0,331897044	0,003259041	RB associated KRAB zinc fing	RBAK
ENSG00000103335	322,6464556	406,6255262	9,851148579	-0,331869507	0,037495794	piezo type mechanosensitiv	PIEZO1
ENSG00000197498	36,64607806	46,21062913	6,911531406	-0,331847981	0,000851061	ribosome production factor	RPF2
ENSG00000073331	23,36861102	29,48741936	6,367415002	-0,331820969	0,009231213	alpha kinase 1	ALPK1
ENSG00000267858	5,132352936	6,458317395	4,087501121	-0,331568226	0,032537946	MZF1 antisense RNA 1	MZF1-AS1
ENSG00000097033	110,2962018	138,3943703	8,535120631	-0,331442616	2,37E-13	SH3 domain containing GRB	SH3GLB1
ENSG00000105976	305,1870034	384,7190331	9,762549076	-0,331372868	0,014779249	MET proto-oncogene, recep	MET
ENSG00000142784	107,9746749	134,9433295	8,479300214	-0,331032251	1,82E-06	WD and tetratricopeptide re	WDTC1
ENSG00000087157	37,85664181	47,63169774	7,072950929	-0,330969847	0,008753538	phosphatidylglycerophosph	PGS1
ENSG00000119820	58,22095278	73,14343157	7,627290221	-0,330916274	0,00014126	Yip1 domain family member	YIPF4
ENSG00000061676	229,7485258	289,3384091	9,50421487	-0,330701151	2,31E-08	NCK associated protein 1	NCKAP1
ENSG00000171055	53,60432812	67,49578254	7,479410689	-0,330515305	1,90E-11	fasciculation and elongation	FEZ2
ENSG00000145391	86,73867716	108,6511892	8,135748419	-0,330075709	0,002717809	SET domain containing 7, his	SETD7
ENSG00000161277	13,65256559	17,18911437	5,722538404	-0,32997706	0,003422917	THAP domain containing 8	THAP8
ENSG00000112367	31,73350301	39,93444382	6,741614189	-0,329720522	1,94E-12	FIG4 phosphoinositide 5-pho	FIG4
ENSG00000105993	104,9642949	131,7891486	8,468059872	-0,329045039	1,29E-09	DnaJ heat shock protein fam	DNAJB6
ENSG00000147799	10,6379807	13,37176029	5,239480995	-0,328727868	0,017430704	Rho GTPase activating prote	ARHGAP39
ENSG00000174915	51,28469313	64,51946098	7,535501255	-0,328695233	2,97E-07	phosphatidylserine synthase	PTDSS2
ENSG00000185917	12,04915305	15,1892067	5,350660979	-0,328416126	0,031877487	SET domain containing 4	SETD4
ENSG00000139719	13,98299873	17,58087462	5,676761501	-0,328294471	0,000257817	VPS33A core subunit of COR	VPS33A
ENSG00000115661	23,01816162	28,95918252	6,441506944	-0,328274434	0,000300122	serine/threonine kinase 16	STK16
ENSG00000119048	47,00676387	59,09794535	7,249282657	-0,327755097	1,18E-05	ubiquitin conjugating enzym	UBE2B
ENSG00000166783	91,52432442	115,1457699	8,380237968	-0,327649269	0,000737568	meiosis regulator and mRNA	MARF1
ENSG00000061794	41,5358951	52,3663263	7,117084357	-0,327366395	0,017668901	mitochondrial ribosomal pro	MRPS35
ENSG00000140948	77,17352545	96,19302382	8,027387068	-0,327105076	0,006698774	zinc finger CCHC-type conta	ZCCHC14
ENSG00000100814	21,88523585	27,61341422	6,305903955	-0,327048361	0,00765772	cyclin B1 interacting protein	CCNB1IP1
ENSG00000178385	21,2087141	26,66558667	6,226802598	-0,326329798	0,002928456	pleckstrin homology domair	PLEKHM3
ENSG00000130703	46,89544522	58,65841113	7,292398132	-0,326207817	5,69E-05	oxysterol binding protein lik	OSBPL2
ENSG00000157637	207,1161748	257,8167553	9,450498035	-0,325881611	0,001349739	solute carrier family 38 men	SLC38A10
ENSG00000168612	19,57491479	24,50462407	6,176782935	-0,325582983	0,00123697	zinc finger SWIM-type conta	ZSWIM1
ENSG00000050327	31,19236385	39,11707876	6,553808099	-0,325582544	0,002186003	Rho guanine nucleotide excl	ARHGEF5
ENSG00000169359	53,33350663	66,942961	7,537216653	-0,325518217	9,93E-06	solute carrier family 33 men	SLC33A1
ENSG00000166821	8,370025317	10,49916515	5,069476279	-0,32543029	0,003253291	peroxisomal biogenesis fact	PEX11A
ENSG00000165506	14,80213903	18,55015059	5,601185494	-0,32539207	0,006589376	dynein axonemal assembly f	DNAAF2
ENSG00000153214	34,00593546	42,26146002	6,705805369	-0,325253712	0,001162001	transmembrane protein 87E	TMEM87B
ENSG00000159423	18,71197008	23,40095472	6,000372379	-0,325000439	0,008670419	aldehyde dehydrogenase 4 f	ALDH4A1
ENSG00000141084	47,83570277	59,94758642	7,307464928	-0,323924008	0,000800627	RAN binding protein 10	RANBP10
ENSG00000149476	38,75957214	48,23867782	7,069387149	-0,32391199	0,000814998	triokinase and FMN cyclase	TKFC
ENSG00000100528	65,07789167	81,39270424	7,663781652	-0,323834013	0,034821189	cornichon family AMPA rece	CNIH1
ENSG00000087095	36,81743563	46,16602966	6,888741345	-0,323673757	1,22E-10	nemo like kinase	NLK
ENSG00000198818	21,30895054	26,69603357	6,07081733	-0,323425693	0,007352329	SFT2 domain containing 1	SFT2D1
ENSG00000090674	30,20772257	37,85711959	6,925546691	-0,323393856	1,20E-06	mucolipin TRP cation chann	MCOLN1
ENSG00000147475	84,62220825	105,9470957	8,358530421	-0,323390171	0,003045543	ER lipid raft associated 2	ERLIN2
ENSG00000153339	51,82406983	64,96999074	7,398484049	-0,323103153	2,89E-09	trafficking protein particle c	TRAPPC8
ENSG00000101442	8,038451815	10,06859723	4,918481499	-0,322903595	0,008801429	actin related protein 5	ACTR5
ENSG00000112679	34,29895822	42,7407393	6,850599816	-0,322687652	0,006475635	dual specificity phosphatase	DUSP22
ENSG00000197798	17,37522169	21,7320692	5,82805357	-0,3218791	0,001193793	family with sequence similar	FAM118B
ENSG00000185019	8,561046146	10,70887113	5,140626513	-0,321697825	0,003445886	U-box domain containing 5	UBOX5
ENSG00000130827	173,6836103	217,5843101	9,157352608	-0,321609766	0,017517018	plexin A3	PLXNA3
ENSG00000179168	5,633463386	7,001384591	4,086805538	-0,321481176	0,02279142	gametogenetin	GGN
ENSG00000110697	202,9938393	255,149825	9,359882239	-0,321479997	0,029418574	phosphatidylinositol transfe	PITPNM1
ENSG00000134440	149,5843434	187,4835495	9,028798813	-0,320700582	2,51E-05	asparaginyl-tRNA synthetase	NARS1
ENSG00000131725	27,22784723	34,05675229	6,424710133	-0,32045093	1,81E-05	WD repeat domain 44	WDR44
ENSG00000160211	130,8940591	163,8734327	8,97292815	-0,320376074	0,000168141	glucose-6-phosphate dehydr	G6PD
ENSG00000204954	6,131456502	7,632130566	4,36362092	-0,320196168	0,040338043	chromosome 12 open readi	C12orf73
ENSG00000126062	60,56191396	75,22633081	7,764319863	-0,320098018	0,000124316	transmembrane protein 115	TMEM115
ENSG00000175066	24,02740587	30,06196112	6,098390986	-0,319691063	0,030164351	glycerol kinase 5	GK5
ENSG00000017797	111,322342	139,1714156	8,557569296	-0,319558859	3,24E-06	ralA binding protein 1	RALBP1
ENSG00000125449	22,56297376	28,1306574	6,393690904	-0,31952411	0,02668802	armadillo repeat containing	ARMC7
ENSG00000104946	53,75195223	67,18893325	7,698480762	-0,319234101	0,000589849	TBC1 domain family membe	TBC1D17
ENSG00000105329	313,2576969	389,1526932	9,886645099	-0,31909054	0,014568965	transforming growth factor	TGFB1
ENSG00000174151	18,84109057	23,4253279	6,109858103	-0,319082961	0,002244251	cytochrome b561 family me	CYB561D1
ENSG00000246582	3,950728089	4,91423373	3,472962783	-0,318881179	0,04559528	TNFRSF10A divergent transc	TNFRSF10A-DT
ENSG00000148296	56,61627618	70,53388226	7,631623455	-0,318852155	8,09E-06	surfeit 6	SURF6
ENSG00000166888	238,8852565	298,9059979	9,69919166	-0,318748824	0,005006243	signal transducer and activa	STAT6
ENSG00000139826	16,95038811	21,15965708	5,81968015	-0,318626146	0,000319917	abhydrolase domain contair	ABHD13
ENSG00000124831	171,5784991	214,1266138	8,996515574	-0,318440017	7,52E-07	LRR binding FLII interacting	LRRFIP1
ENSG00000115947	25,8002588	32,25812231	6,445792577	-0,318342621	3,05E-05	origin recognition complex s	ORC4
ENSG00000110318	12,11110139	15,10207076	5,484532658	-0,318236209	0,015301681	centrosomal protein 126	CEP126
ENSG00000182093	27,76248112	34,59025729	6,348034926	-0,31795809	0,003025862	guided entry of tail-anchore	GET1
ENSG00000170852	48,98151204	61,13943135	7,242388264	-0,317922756	1,23E-11	kelch repeat and BTB domai	KBTBD2
ENSG00000215717	20,67391898	25,53427606	6,376692496	-0,317909598	0,003078863	transmembrane protein 167	TMEM167B
ENSG00000188554	150,8269417	188,1953824	9,093175304	-0,31750743	0,000304932	NBR1 autophagy cargo rece	NBR1
ENSG00000274211	39,17549501	48,71368544	7,099907084	-0,317336224	8,39E-06	suppressor of cytokine signa	SOCS7
ENSG00000182871	1571,22946	1935,734452	12,15073127	-0,317207793	0,007548524	collagen type XVIII alpha 1 c	COL18A1
ENSG00000116406	65,24128223	81,28724106	7,839426629	-0,317023694	0,000277198	ER degradation enhancing a	EDEM3
ENSG00000152484	52,05320043	65,05877145	7,341820368	-0,316706741	0,000597373	ubiquitin specific peptidase	USP12
ENSG00000155903	25,57595229	31,85632171	6,205892091	-0,316239762	0,000329603	RAS p21 protein activator 2	RASA2
ENSG00000180901	60,04745727	74,89695117	7,693870141	-0,316142537	3,50E-05	potassium channel tetramer	KCTD2
ENSG00000176095	102,4217124	127,6237748	8,413572944	-0,315694109	5,51E-05	inositol hexakisphosphate ki	IP6K1
ENSG00000137513	17,15333092	21,39491374	5,865027358	-0,315455016	0,012510831	asparaginyl-tRNA synthetase	NARS2
ENSG00000108733	6,990080395	8,663179645	4,703929497	-0,315182248	0,0110867	peroxisomal biogenesis fact	PEX12
ENSG00000198718	29,30318602	36,51960713	6,657480563	-0,315147742	2,19E-07	TOG array regulator of axon	TOGARAM1
ENSG00000186104	8,156994057	10,16776013	4,881550095	-0,314738922	0,006846119	cytochrome P450 family 2 su	CYP2R1
ENSG00000006007	85,8832874	107,0080329	8,180801074	-0,314638254	2,57E-05	glycerophosphodiester phos	GDE1
ENSG00000231312	11,3011039	14,07120705	5,194125578	-0,314332608	0,033510589	MAP4K3 divergent transcrip	MAP4K3-DT
ENSG00000008710	167,7034078	208,0990059	9,186718726	-0,314257971	0,006735141	polycystin 1, transient recep	PKD1
ENSG00000100335	60,84047543	75,7776371	7,744132829	-0,313856541	6,25E-06	mitochondrial elongation fa	MIEF1
ENSG00000136485	182,470261	227,0166674	9,124379887	-0,313631538	0,000666985	DDB1 and CUL4 associated f	DCAF7
ENSG00000162889	150,1304331	186,8683498	9,026908371	-0,313622811	0,000924628	MAPK activated protein kina	MAPKAPK2
ENSG00000117595	25,90710563	32,2581875	6,397399835	-0,313258791	0,005485391	interferon regulatory factor	IRF6
ENSG00000179820	159,5427552	197,5232721	9,028301477	-0,313244655	0,016460791	myeloid associated different	MYADM
ENSG00000007202	378,5331666	469,9205645	10,27540208	-0,312845777	7,83E-05	KIAA0100	KIAA0100
ENSG00000141551	262,2071288	326,4029678	9,768508669	-0,3128318	7,22E-06	casein kinase 1 delta	CSNK1D
ENSG00000101421	144,6344794	179,5319986	8,884213302	-0,312412777	0,000236872	charged multivesicular body	CHMP4B
ENSG00000132128	98,48468168	117,0571202	8,423391909	-0,312164787	0,037252957	leucine rich repeat containir	LRRC41
ENSG00000100664	152,2778583	189,3544531	8,902941445	-0,31215356	1,61E-05	eukaryotic translation initiat	EIF5
ENSG00000095574	13,92793413	17,30980911	5,50326677	-0,311980532	0,002639264	IKAROS family zinc finger 5	IKZF5
ENSG00000129245	66,44358683	82,44656389	7,771326456	-0,311956665	6,69E-08	FMR1 autosomal homolog 2	FXR2
ENSG00000133731	20,48681766	25,46100312	5,917162872	-0,311764277	0,005883024	inositol monophosphatase 1	IMPA1
ENSG00000155256	36,21723381	44,92450253	6,888207766	-0,311532781	7,48E-06	zinc finger FYVE-type contai	ZFYVE27
ENSG00000075151	219,9145146	273,1708666	9,511981889	-0,31140872	2,75E-10	eukaryotic translation initiat	EIF4G3
ENSG00000135482	13,31827997	16,54656963	5,63721073	-0,311407257	0,000516967	zinc finger CCCH-type conta	ZC3H10
ENSG00000059145	27,52000708	34,22063739	6,69882505	-0,31103795	0,000243722	unk like zinc finger	UNKL
ENSG00000104812	124,5314841	154,4228384	8,810331196	-0,310804711	8,50E-05	glycogen synthase 1	GYS1
ENSG00000032444	180,1495948	222,8215862	9,312322821	-0,310171983	7,07E-08	patatin like phospholipase d	PNPLA6

ENSG00000119537	93,65930601	116,3819727	8,122117644	-0,309669526	0,000102283	3-ketodihydrosphingosine re	KDSR
ENSG00000278845	34,21260684	42,50357411	6,887903818	-0,309459308	5,93E-05	mitochondrial ribosomal pro	MRPL45
ENSG00000109519	29,39366005	36,47700368	6,611261346	-0,309161313	0,003095276	GrpE like 1, mitochondrial	GRPEL1
ENSG00000007392	44,59576591	55,34984252	7,255073245	-0,309074397	0,016847857	LUC7 like	LUC7L
ENSG00000176994	92,99783545	115,3919797	8,307658673	-0,309066605	0,000136259	SMCR8-C9orf72 complex sul	SMCR8
ENSG00000123297	17,97995026	22,32176426	5,823430329	-0,308997404	0,000145352	Ts translation elongation fac	TSFM
ENSG00000167778	45,12904523	55,76140715	7,294781335	-0,308661368	0,021973382	SPRY domain containing 3	SPRYD3
ENSG00000038274	53,95677843	67,01620299	7,500393373	-0,308019645	0,002122852	methionine adenosyltransfe	MAT2B
ENSG00000257218	48,06087983	59,57165856	7,321097666	-0,308010249	2,85E-06	glutamyl-tRNA amidotransfe	GATC
ENSG00000164074	15,52145014	19,27146995	5,614125569	-0,307927679	0,01043918	abhydrolase domain contair	ABHD18
ENSG00000168734	16,62853387	20,46603781	5,806754168	-0,307910258	0,01557049	cAMP-dependent protein kii	PKIG
ENSG00000104660	40,15716635	49,44791141	7,015762463	-0,307799777	0,000596833	leptin receptor overlapping	LEPROTL1
ENSG00000106012	40,49491938	50,26079182	7,065322607	-0,307715625	0,00107776	IQ motif containing E	IQCE
ENSG00000115548	63,5337636	78,15950264	7,823547786	-0,307328493	0,003281307	lysine demethylase 3A	KDM3A
ENSG00000175104	19,52960673	24,18743905	6,070228417	-0,306675975	0,000135394	TNF receptor associated fact	TRAF6
ENSG00000198642	75,73955148	93,30994384	8,010492415	-0,30659663	0,009128503	kelch like family member 9	KLHL9
ENSG00000140987	14,17025257	17,56636937	5,65319028	-0,306459761	8,25E-06	zinc finger and SCAN domair	ZSCAN32
ENSG00000185753	23,71106533	29,21067827	6,286700216	-0,306410832	1,41E-06	chromosome X open reading	CXorf38
ENSG00000166557	50,7893085	62,47662613	7,509456843	-0,306182661	0,000401951	transmembrane p24 traffick	TMED3
ENSG00000134243	356,1555501	437,7188452	10,08814089	-0,305966346	0,000588787	sortilin 1	SORT1
ENSG00000185515	40,07978609	49,62267436	6,942335817	-0,305954695	0,026619649	BRCA1/BRCA2-containing cc	BRCC3
ENSG00000133606	93,76068748	115,8243727	8,358547986	-0,305949061	6,07E-05	makorin ring finger protein :	MKRN1
ENSG00000105778	57,70097455	71,35715033	7,597651131	-0,305756177	5,48E-05	AVL9 cell migration associat	AVL9
ENSG00000120314	62,87454306	77,86299907	7,823425053	-0,305510346	3,06E-06	WD repeat domain 55	WDR55
ENSG00000160445	102,8954652	126,9373726	8,54080739	-0,30537902	4,14E-06	zyg-11 related cell cycle regu	ZER1
ENSG00000164168	77,27974296	95,68254428	8,041753017	-0,305252659	2,34E-10	transmembrane protein 184	TMEM184C
ENSG00000267575	8,214173797	10,1716672	4,839263321	-0,305243156	0,00764469	long intergenic non-protein	LINC02987
ENSG00000122085	25,33450016	31,37440337	6,534706359	-0,305165645	0,013612266	mitochondrial transcription	MTERF4
ENSG00000198752	426,8608593	528,0134099	10,37538253	-0,305069974	0,000299176	CDC42 binding protein kinas	CDC42BPB
ENSG00000156697	26,82950491	33,23880453	6,622272858	-0,304922615	0,00018151	UTP14A small subunit proce	UTP14A
ENSG00000172915	22,73893037	28,09381405	6,400231971	-0,304798985	0,046911355	neurobeachin	NBEA
ENSG00000144567	116,695899	144,527806	8,790057977	-0,304780688	4,29E-05	reticulophagy regulator fam	RETREG2
ENSG00000213563	23,88827458	29,5537246	6,6158404	-0,304736658	0,002164145	chromosome 8 open reading	C8orf82
ENSG00000145901	428,26919	528,6463641	10,5499348	-0,304663141	0,030839088	TNFAIP3 interacting protein	TNIP1
ENSG00000139718	66,54795402	81,92911736	7,736574869	-0,304538472	0,003204328	SET domain containing 1B, h	SETD1B
ENSG00000182362	6,727398488	8,29134899	4,666918209	-0,304404178	0,006997299	ybeY metalloendoribonucle	YBEY
ENSG00000167535	75,85213061	92,65178598	7,916270149	-0,304400459	0,029423161	calcium voltage-gated chanr	CACNB3
ENSG00000125648	104,0656446	125,5194762	8,606101129	-0,304386213	0,010388629	solute carrier family 25 men	SLC25A23
ENSG00000146416	55,71083207	69,13738423	7,450851955	-0,3040593	0,003365598	androgen induced 1	AIG1
ENSG00000102309	20,5565309	24,91125341	5,812903693	-0,303687946	0,010940455	peptidylprolyl cis/trans isom	PIN4
ENSG00000178719	197,7557087	243,9566183	9,523645452	-0,303665558	6,67E-06	glutamate ionotropic recept	GRINA
ENSG00000040633	56,45472344	69,21220192	7,602286498	-0,303619071	0,024648903	PHD finger protein 23	PHF23
ENSG00000144233	51,28798519	63,23188464	7,432523613	-0,303563198	9,25E-06	AMMECR1 like	AMMECR1L
ENSG00000205133	30,92710219	38,22012697	6,670947217	-0,303400398	0,02328472	triple QxxK/R motif containi	TRIQK
ENSG00000122696	14,09480859	17,43269761	5,579585278	-0,303318436	2,62E-05	solute carrier family 25 men	SLC25A51
ENSG00000256537	30,66815559	37,74643123	6,642899832	-0,302844491	0,001939296	small integral membrane pr	SMIM10L1
ENSG00000088930	123,1522564	151,9882233	8,646854937	-0,302358627	0,000298954	5'-3' exoribonuclease 2	XRN2
ENSG00000087152	92,44866695	113,6501483	8,3187288	-0,302291633	5,27E-08	ataxin 7 like 3	ATXN7L3
ENSG00000145916	42,32774676	52,28536905	7,002273879	-0,302189663	0,002678842	required for meiotic nuclear	RMND5B
ENSG00000104450	18,72133165	23,1725895	5,777601921	-0,302152063	0,027909049	sperm associated antigen 1	SPAG1
ENSG00000138600	68,6851616	84,92421251	7,810195702	-0,302026544	0,016748227	signal peptide peptidase like	SPPL2A
ENSG00000213753	39,33552742	48,40344434	7,052337927	-0,301986907	0,001853042	CENPB DNA-binding domain	CENPBD2P
ENSG00000107404	125,06815	154,44437	8,752763738	-0,301321358	8,86E-06	dishevelled segment polarity	DVL1
ENSG00000198131	23,40475862	28,90947313	6,232164596	-0,301226641	6,97E-05	zinc finger protein 544	ZNF544
ENSG00000023228	133,0259836	164,42009	8,80633228	-0,301158062	2,03E-05	NADH:ubiquinone oxidored	NDUFS1
ENSG00000170653	55,14301978	67,84346945	7,475039264	-0,300791216	0,001374194	activating transcription facto	ATF7
ENSG00000171443	20,553837	25,34253408	6,159698251	-0,300695468	0,039797975	zinc finger protein 524	ZNF524
ENSG00000168538	45,82961294	56,57813267	7,266238809	-0,300656884	9,84E-11	trafficking protein particle c	TRAPPC11
ENSG00000110429	28,75407781	35,34957223	6,646333183	-0,300400745	0,021633617	F-box protein 3	FBXO3
ENSG00000143093	37,63414396	46,45297906	7,063270635	-0,300314529	8,27E-08	striatin interacting protein 1	STRIP1
ENSG00000125734	75,11342773	92,85573114	7,951899064	-0,300289698	0,046198955	G protein-coupled receptor	GPR108
ENSG00000182108	29,42931289	36,13753048	6,687417251	-0,300256845	0,000251187	Dexi homolog	DEXI
ENSG00000186352	7,507703206	9,294027785	4,72949976	-0,300063884	0,025869404	ankyrin repeat domain 37	ANKRD37
ENSG00000125037	47,39804717	58,45309476	7,392322509	-0,299804587	0,000835298	ER membrane protein comp	EMC3
ENSG00000104412	25,87809787	31,91838361	6,464829378	-0,299703804	0,001571003	ER membrane protein comp	EMC2
ENSG00000096063	107,4157556	132,5095397	8,358767384	-0,299662618	2,52E-09	SRSF protein kinase 1	SRPK1
ENSG00000172466	83,18395035	102,5334681	8,166648911	-0,299464423	2,50E-05	zinc finger protein 24	ZNF24
ENSG00000128923	65,00119364	79,53477337	7,69720743	-0,299259793	0,020881237	MINDY lysine 48 deubiquitir	MINDY2
ENSG00000166822	28,18745105	34,86679281	6,356923695	-0,299164737	0,024288772	transmembrane protein 170	TMEM170A
ENSG00000117862	72,96712857	90,06026464	7,950614941	-0,298955044	0,001211386	thioredoxin domain containi	TXNDC12
ENSG00000197892	118,6353187	146,0610145	8,425746578	-0,298856089	0,001401472	kinesin family member 13B	KIF13B
ENSG00000150776	41,31340685	50,93551167	7,221113836	-0,298850553	1,56E-06	NKAP domain containing 1	NKAPD1
ENSG00000129219	35,18897641	43,21285689	6,845858719	-0,298171464	6,97E-05	phospholipase D2	PLD2
ENSG00000110911	35,60114855	43,56203482	7,046282247	-0,298008146	0,027766346	solute carrier family 11 men	SLC11A2
ENSG00000023330	48,2320792	59,3566908	7,324543122	-0,297910405	6,24E-05	5'-aminolevulinate synthase	ALAS1
ENSG00000171763	14,64523916	18,02953132	5,578827858	-0,297852566	0,007784158	spermatogenesis associated	SPATA5L1
ENSG00000171566	56,80520382	69,97846554	7,591241213	-0,297817479	1,57E-07	pleiotropic regulator 1	PLRG1
ENSG00000085733	370,3077826	455,9471792	10,27269853	-0,297722867	0,003710191	cortactin	CTTN
ENSG00000119977	53,88388319	66,42735478	7,547945244	-0,297555433	0,006294423	tectonic family member 3	TCTN3
ENSG00000109133	48,39719222	59,56121307	7,272882073	-0,29735184	2,98E-06	transmembrane protein 33	TMEM33
ENSG00000270170	10,00050384	12,24232011	5,099151501	-0,297271918	0,002302159	NCBP2 antisense 2 (head to	NCBP2AS2
ENSG00000079785	116,8687149	143,0946451	8,373761999	-0,297154363	0,015512406	DEAD-box helicase 1	DDX1
ENSG00000170100	9,265513087	11,40326832	5,047164805	-0,29701281	0,001406759	zinc finger protein 778	ZNF778
ENSG00000005486	48,99017979	59,96329331	7,332599248	-0,296731816	6,95E-05	rhomboid domain containin	RHBDD2
ENSG00000175137	67,43040669	82,62792288	7,944744262	-0,296599195	0,014227508	SH3 binding domain protein	SH3BP5L
ENSG00000118960	62,87801059	77,34335089	7,772545154	-0,295618929	0,001748217	HCLS1 binding protein 3	HS1BP3
ENSG00000121579	172,7706845	212,6487628	8,960294574	-0,295551023	1,97E-09	N-alpha-acetyltransferase 5(	NAA50
ENSG00000239305	37,91368077	46,08621827	7,000401568	-0,295434205	0,025793518	ring finger protein 103	RNF103
ENSG00000067182	256,3612502	313,5688639	9,799831114	-0,295270072	0,000152113	TNF receptor superfamily m	TNFRSF1A
ENSG00000183283	139,515328	171,1280093	8,918489921	-0,29522139	2,87E-06	DAZ associated protein 2	DAZAP2
ENSG00000106346	23,47828343	28,75190264	6,278158127	-0,295204903	0,011842057	ubiquitin specific peptidase	USP42
ENSG00000116138	37,24206559	45,81998934	7,002448247	-0,295198104	1,79E-05	DnaJ heat shock protein fam	DNAJC16
ENSG00000090565	114,2361614	140,0924748	8,496118291	-0,294875321	3,30E-05	RAB11 family interacting pro	RAB11FIP3
ENSG00000107872	13,80015066	16,93044346	5,68911449	-0,294736066	0,001905789	F-box and leucine rich repea	FBXL15
ENSG00000109736	121,4400151	149,1588742	8,823891235	-0,294724448	0,01043918	major facilitator superfamily	MFSD10
ENSG00000167965	46,10114485	56,54321788	7,259037182	-0,294682644	5,29E-05	MTOR associated protein, LS	MLST8
ENSG00000164609	48,77975085	59,91879071	7,433920746	-0,294675663	0,002099994	SLU7 homolog, splicing facto	SLU7
ENSG00000112234	33,13807858	40,75529808	6,838680136	-0,294089181	0,000995966	F-box and leucine rich repea	FBXL4
ENSG00000140859	232,0743388	283,5701491	9,667518362	-0,293977033	0,01838133	kinesin family member C3	KIFC3
ENSG00000173548	105,8682828	129,7705646	8,542298885	-0,293692471	3,71E-06	sorting nexin 33	SNX33
ENSG00000135912	42,02706147	51,23210512	7,114286845	-0,293510349	0,037726208	tubulin tyrosine ligase like 4	TTLL4
ENSG00000035687	52,37148801	64,53726632	7,388981886	-0,293338946	0,032001283	adenylosuccinate synthase 2	ADSS2
ENSG00000131149	132,4935776	162,5450847	8,577168869	-0,293216548	0,024939159	Gse1 coiled-coil protein	GSE1
ENSG00000116668	6,166089932	7,571771291	4,547243148	-0,293194968	0,018860866	SWT1 RNA endoribonucleas	SWT1
ENSG00000185043	57,90859393	70,98311094	7,443497261	-0,293095326	0,024810041	calcium and integrin binding	CIB1
ENSG00000127914	100,6378457	123,2471957	8,371586381	-0,293070665	0,01240676	A-kinase anchoring protein 9	AKAP9
ENSG00000155636	10,04904098	12,3395641	5,050890381	-0,292724128	0,017154398	RNA binding motif protein 4	RBM45
ENSG00000136830	859,6671835	1054,954051	11,46147082	-0,292651269	0,000238792	niban apoptosis regulator 2	NIBAN2
ENSG00000162396	10,69588585	13,13487069	5,351268543	-0,292443282	0,03532597	prolyl-tRNA synthetase 2, m	PARS2
ENSG00000120910	13,55462869	16,61142068	5,489582042	-0,292369199	0,000346585	protein phosphatase 3 catal	PPP3CC
ENSG00000164542	15,03110209	18,39750639	5,401787572	-0,292351709	0,04927027	KIAA0895	KIAA0895
ENSG00000108306	32,91981871	40,14552051	6,882427568	-0,292313346	0,000286033	F-box and leucine rich repea	FBXL20
ENSG00000197818	23,32060885	28,51188542	6,419012244	-0,292012965	0,013131665	solute carrier family 9 meml	SLC9A8
ENSG00000130159	18,53154072	22,75572984	6,323183737	-0,2919182	0,030856092	ECSIT signaling integrator	ECSIT
ENSG00000158092	27,05806639	33,19129706	6,478438817	-0,291777353	0,001026371	NCK adaptor protein 1	NCK1

ENSG00000143499	47,46856495	58,18176128	7,111285758	-0,29177262	0,001298407	SET and MYND domain cont	SMYD2
ENSG00000137364	25,60122922	31,4400412	6,419636398	-0,291627213	0,030646971	thiopurine S-methyltransfer	TPMT
ENSG00000107521	62,68983032	76,73778922	7,74296228	-0,291467869	0,000633998	HPS1 biogenesis of lysosom	HPS1
ENSG00000135164	50,95854275	62,50484623	7,384717499	-0,291391754	0,017154398	cyclin D binding myb like tra	DMTF1
ENSG00000089234	27,0634743	33,17572865	6,500743056	-0,291185186	2,76E-08	BRCA1 associated protein	BRAP
ENSG00000105072	15,4341968	18,9348716	5,821034806	-0,291099203	0,000886469	chromosome 19 open readi	C19orf44
ENSG00000152104	320,3199468	391,8074743	9,854241878	-0,291014883	0,043895442	protein tyrosine phosphatas	PTPN14
ENSG00000102531	118,478775	145,2361509	8,674080838	-0,290730036	0,001591103	fibronectin type III domain c	FNDC3A
ENSG00000051128	37,85139447	46,07154851	7,092261477	-0,290596238	0,015628892	homer scaffold protein 3	HOMER3
ENSG00000185658	62,17753003	76,19545255	7,779126333	-0,290449933	0,005883024	bromodomain and WD repe	BRWD1
ENSG00000087269	61,64289722	75,54438654	7,656800632	-0,290428633	1,45E-09	NOP14 nucleolar protein	NOP14
ENSG00000160991	88,36482236	107,968597	8,007930286	-0,290379007	0,011812428	ORAI calcium release-activa	ORAI2
ENSG00000074356	82,35972288	100,815684	8,086053774	-0,29031975	0,017053177	nuclear cap binding subunit	NCBP3
ENSG00000164040	131,5461545	161,1576382	8,760517113	-0,290300043	0,007321584	progesterone receptor mem	PGRMC2
ENSG00000100564	9,639937813	11,82266907	5,163752805	-0,290210484	0,019186564	phosphatidylinositol glycan	PIGH
ENSG00000144895	46,90208462	57,37917702	7,431723412	-0,290181123	0,006308972	eukaryotic translation initiat	EIF2A
ENSG00000163597	46,46996402	56,83993574	7,473112685	-0,289970372	0,000230013	small nucleolar RNA host ge	SNHG16
ENSG00000186376	10,12424575	12,37461812	5,171369309	-0,289741772	0,006325195	zinc finger protein 75D	ZNF75D
ENSG00000006534	55,15458531	66,92782533	7,350601092	-0,289474631	0,000343512	aldehyde dehydrogenase 3 f	ALDH3B1
ENSG00000109103	31,36238527	38,16531523	6,89589905	-0,289253222	0,001129898	unc-119 lipid binding chape	UNC119
ENSG00000134333	940,8335681	1147,005349	11,53405437	-0,289076144	0,000859749	lactate dehydrogenase A	LDHA
ENSG00000198863	32,42475161	39,60477667	6,853441685	-0,289065458	0,000134039	RUN domain containing 1	RUNDC1
ENSG00000115364	33,65621248	41,22292543	6,858186539	-0,288985906	5,38E-05	mitochondrial ribosomal pro	MRPL19
ENSG00000175354	31,23843404	38,28804806	6,671278451	-0,288876591	0,000162497	protein tyrosine phosphatas	PTPN2
ENSG00000281649	53,10344294	64,96164741	7,636526783	-0,288826459	0,001080224	endogenous Bornavirus like	EBLN3P
ENSG00000136986	73,43927952	89,25910996	8,064457817	-0,288200772	0,014721637	derlin 1	DERL1
ENSG00000115464	141,3067458	171,9404105	8,849840726	-0,287989878	0,00968521	ubiquitin specific peptidase	USP34
ENSG00000178149	27,10886882	33,18843568	6,661721263	-0,287957836	0,005480792	DALR anticodon binding dor	DALRD3
ENSG00000124532	29,18103739	35,6895556	6,656536751	-0,287904787	3,30E-05	magnesium transporter MRS	MRS2
ENSG00000172661	63,54862194	77,72750523	7,975386477	-0,287585095	0,000453223	WASH complex subunit 2C	WASHC2C
ENSG00000138760	334,8831709	408,7497637	10,05371158	-0,287331845	0,001303345	scavenger receptor class B n	SCARB2
ENSG00000135956	126,7095631	154,7954391	8,784972137	-0,2872443	8,97E-05	transmembrane protein 127	TMEM127
ENSG00000186222	12,88526422	15,57296317	5,476044412	-0,287169706	0,025149157	biogenesis of lysosomal orga	BLOC1S4
ENSG00000124571	61,92444459	75,63941521	7,702685029	-0,286890995	1,23E-05	exportin 5	XPO5
ENSG00000075790	47,08407796	57,55075486	7,148889693	-0,28663783	0,018704526	B cell receptor associated pr	BCAP29
ENSG00000152520	26,21119954	32,0564186	6,47015814	-0,286632087	5,56E-05	poly(A) specific ribonuclease	PAN3
ENSG00000108443	34,45215395	42,10395463	6,836136414	-0,286507338	6,24E-05	ribosomal protein S6 kinase	RPS6KB1
ENSG00000136816	37,64635458	46,03248519	6,866152425	-0,286472761	0,001610711	torsin family 1 member B	TOR1B
ENSG00000164615	41,37365163	50,5494392	7,138679304	-0,286300652	0,005792845	calcium modulating ligand	CAMLG
ENSG00000198721	50,21589747	61,25563811	7,348979516	-0,286046243	0,00628744	enoyl-CoA delta isomerase 2	ECI2
ENSG00000048140	57,39236045	69,98242234	7,618036965	-0,285782021	0,001704546	tetraspanin 17	TSPAN17
ENSG00000143418	185,2288783	225,3607178	9,271998504	-0,2855718	0,003238071	ceramide synthase 2	CERS2
ENSG00000162600	11,47626098	13,96349672	5,327624076	-0,285245901	0,013584045	OMA1 zinc metallopeptidas	OMA1
ENSG00000146083	62,08071884	75,30968264	7,766366343	-0,285044267	0,038875484	ring finger protein 44	RNF44
ENSG00000130177	58,36637372	71,23430624	7,676826458	-0,284909715	1,18E-05	cell division cycle 16	CDC16
ENSG00000084092	34,82875928	42,53975571	6,96615815	-0,284722673	0,000849778	nitric oxide associated 1	NOA1
ENSG00000181929	44,7387508	54,50199076	7,286296123	-0,284686765	0,003949726	protein kinase AMP-activate	PRKAG1
ENSG00000155506	283,8939971	346,6907577	9,889748839	-0,28467484	0,000213373	La ribonucleoprotein 1, tran	LARP1
ENSG00000132825	10,81378459	13,16925329	5,207062637	-0,284624332	0,001475586	protein phosphatase 1 regul	PPP1R3D
ENSG00000019995	45,74949643	55,84114168	7,266485083	-0,284404828	7,73E-06	zinc finger RANBP2-type cor	ZRANB1
ENSG00000118402	13,74791117	16,7664615	5,335633426	-0,284258238	0,008760865	ELOVL fatty acid elongase 4	ELOVL4
ENSG00000101290	97,77555917	119,2564597	8,288295328	-0,28389443	0,000636936	CDP-diacylglycerol synthase	CDS2
ENSG00000225663	75,963855	92,58977814	8,141914181	-0,28378039	0,00464409	MAPK regulated corepresso	MCRIP1
ENSG00000125991	159,4684001	193,4866685	9,106251039	-0,283733175	0,029876673	ERGIC and golgi 3	ERGIC3
ENSG00000160213	104,4837015	127,4576765	8,376816054	-0,283299401	3,10E-05	cystatin B	CSTB
ENSG00000174796	13,98952847	17,06165701	5,511561208	-0,28318258	0,017938848	THAP domain containing 6	THAP6
ENSG00000171222	40,64925021	48,98080841	7,147889666	-0,283131356	0,028721939	SCAN domain containing 1	SCAND1
ENSG00000152942	31,0553906	37,8332525	6,825917602	-0,283071897	0,001111512	RAD17 checkpoint clamp loa	RAD17
ENSG00000165233	26,97621002	32,79040367	6,531186751	-0,282925076	0,024707535	caspase recruitment domair	CARD19
ENSG00000144228	24,992569	30,45180171	6,2362117	-0,282753642	0,001927972	speckle type BTB/POZ prote	SPOPL
ENSG00000078142	47,95112723	58,29863002	7,341951948	-0,282701168	0,000157892	phosphatidylinositol 3-kinas	PIK3C3
ENSG00000100852	113,0100162	136,3312842	8,479840361	-0,282487871	0,007728096	Rho GTPase activating prote	ARHGAP5
ENSG00000173545	32,8005964	39,9355841	6,864924036	-0,282365179	8,64E-05	zinc finger protein 622	ZNF622
ENSG00000176542	37,47209595	45,60239397	6,93990962	-0,282142178	0,009333947	upstream transcription facto	USF3
ENSG00000165678	122,3401385	149,1390843	8,755186595	-0,281955316	0,000453234	growth hormone inducible t	GHITM
ENSG00000172171	5,989128393	7,295050685	4,357813317	-0,281415258	0,049854097	transcription elongation fact	TEFM
ENSG00000188315	11,05914577	13,46015257	5,150260162	-0,280977597	0,015351198	chromosome 3 open reading	C3orf62
ENSG00000166949	183,9183911	223,9060873	9,003028168	-0,280877802	0,009704268	SMAD family member 3	SMAD3
ENSG00000134287	137,8172775	167,2650752	8,746942389	-0,280673388	0,000411452	ADP ribosylation factor 3	ARF3
ENSG00000169609	12,42842468	15,12806722	5,619084115	-0,280490913	0,008957196	chromosome 15 open readi	C15orf40
ENSG00000153989	38,46855444	46,73823768	7,00088235	-0,280385056	0,000186868	NUS1 dehydrodolichyl diphc	NUS1
ENSG00000145782	34,05826709	41,3339373	6,80491615	-0,280196186	3,07E-05	autophagy related 12	ATG12
ENSG00000166171	14,34218917	17,45129094	5,727747889	-0,27999216	0,014436421	deleted in primary ciliary dy	DPCD
ENSG00000074319	54,96347924	66,8681672	7,470280275	-0,279891814	2,53E-06	tumor susceptibility 101	TSG101
ENSG00000107819	55,13672572	66,12585604	7,587643266	-0,279764233	0,011319944	sideroflexin 3	SFXN3
ENSG00000070770	84,6927038	102,9451764	8,147727414	-0,279480029	0,002642877	casein kinase 2 alpha 2	CSNK2A2
ENSG00000063046	521,3329659	632,8528012	10,84695433	-0,279404825	0,033385434	eukaryotic translation initiat	EIF4B
ENSG00000122140	47,71922315	58,03921469	7,367378904	-0,279323112	0,000161391	mitochondrial ribosomal pro	MRPS2
ENSG00000119242	67,34351229	82,14978469	7,846733042	-0,279284651	0,009708421	coiled-coil domain containin	CCDC92
ENSG00000151893	79,86716298	97,07599166	8,024638725	-0,279215976	8,89E-06	CDK2 associated cullin doma	CACUL1
ENSG00000148481	25,26069784	30,71091507	6,437760026	-0,27901599	1,30E-05	MINDY lysine 48 deubiquitir	MINDY3
ENSG00000180817	79,00173566	95,14732418	7,914345146	-0,279014507	0,025653368	inorganic pyrophosphatase	PPA1
ENSG00000156171	40,07376583	48,68582633	7,060970623	-0,278958568	0,040716691	DNA damage regulated auto	DRAM2
ENSG00000185730	11,99404242	14,59708964	5,50718156	-0,278936495	0,000458664	zinc finger protein 696	ZNF696
ENSG00000178802	59,46408207	72,04592711	7,796188322	-0,278699715	0,001211386	mannose phosphate isomer	MPI
ENSG00000135047	100,5034501	121,4310718	8,558395642	-0,277902338	0,048147098	cathepsin L	CTSL
ENSG00000008869	31,28510023	38,03899587	6,748284498	-0,277793019	0,008444663	HEAT repeat containing 5B	HEATR5B
ENSG00000197969	78,71577395	95,62456236	8,012531109	-0,277736712	0,006523003	vacuolar protein sorting 13	VPS13A
ENSG00000172766	18,44029852	22,36685712	5,685251476	-0,277522267	0,019054642	N-alpha-acetyltransferase 16	NAA16
ENSG00000125304	188,7300079	227,9740809	9,329807405	-0,277454085	4,41E-05	transmembrane 9 superfam	TM9SF2
ENSG00000167977	41,89437004	50,72474993	7,076561866	-0,277130545	0,008473306	potassium channel tetramer	KCTD5
ENSG00000064601	278,0508317	336,9972592	10,01181938	-0,276990253	0,024549936	cathepsin A	CTSA
ENSG00000136802	191,6821095	230,1014022	9,073940965	-0,276960971	0,001707928	leucine rich repeat containir	LRRC8A
ENSG00000116251	158,8840346	192,3304244	9,12472543	-0,276956464	0,009199756	ribosomal protein L22	RPL22
ENSG00000107341	107,9620044	130,4285239	8,485157218	-0,27655154	0,000648403	ubiquitin conjugating enzym	UBE2R2
ENSG00000197302	14,07549157	17,03940537	5,461642806	-0,276304777	0,013750815	KRAB box domain containing	KRBOX5
ENSG00000106052	103,9175043	125,7713171	8,53325122	-0,276083661	1,34E-05	Tax1 binding protein 1	TAX1BP1
ENSG00000174839	25,358416	30,7994273	6,290037836	-0,27603088	0,034547452	DENN domain containing 6A	DENND6A
ENSG00000183340	24,38723227	29,47977162	6,324577427	-0,275853869	0,000489631	JRK like	JRKL
ENSG00000100897	65,58443302	79,63149525	7,879438884	-0,275836341	0,000127733	DDB1 and CUL4 associated f	DCAF11
ENSG00000132467	49,20793235	59,63941059	7,280723896	-0,275826501	0,000471793	UTP3 small subunit processo	UTP3
ENSG00000067533	18,39215837	22,31768251	5,882764794	-0,275498019	0,049807042	ribosomal RNA processing 1	RRP15
ENSG00000164543	73,83170655	89,1810545	7,801776225	-0,275485991	0,01240676	serine/threonine kinase 17a	STK17A
ENSG00000162980	24,40565563	29,6096684	6,254858636	-0,27521107	0,005201889	ADP ribosylation factor like	ARL5A
ENSG00000198171	60,95203269	73,76101438	7,647664405	-0,275035897	0,000632998	DDRGK domain containing 1	DDRGK1
ENSG00000125630	28,3229612	34,35548217	6,593789047	-0,274948588	0,002110069	RNA polymerase I subunit B	POLR1B
ENSG00000135622	44,55093654	53,89677091	7,075153362	-0,274946691	0,00164352	ssemaphorin 4F	SEMA4F
ENSG00000108946	180,9833736	219,4446542	9,200533449	-0,274945512	0,011177001	protein kinase cAMP-depen	PRKAR1A
ENSG00000110060	10,04070021	12,16181648	5,150274764	-0,274941941	0,025256081	pseudouridine synthase 3	PUS3
ENSG00000070269	26,1543886	31,71927971	6,476056605	-0,274774022	0,01263669	transmembrane protein 260	TMEM260
ENSG00000162604	22,10750463	26,74903744	6,21845573	-0,274714651	0,003170841	TM2 domain containing 1	TM2D1
ENSG00000197363	6,481504716	7,85252447	4,543388822	-0,274697053	0,031333004	zinc finger protein 517	ZNF517
ENSG00000247626	8,561024043	10,35956724	4,889254987	-0,274529513	0,037252957	methionyl-tRNA synthetase	mars-02
ENSG00000089220	151,3966248	184,123952	9,083414194	-0,274527256	0,036245832	phosphatidylethanolamine b	PEBP1
ENSG00000174032	31,1521641	37,80235734	6,815371148	-0,274486154	0,005349696	solute carrier family 25 men	SLC25A30

ENSG00000138756	68,82659932	83,28500798	7,675040851	-0,274469463	0,002342027	BMP2 inducible kinase	BMP2K
ENSG00000067704	115,9023128	140,4218657	8,576450494	-0,274149774	5,32E-06	isoleucyl-tRNA synthetase 2,	IARS2
ENSG00000056586	106,1626043	128,4708592	8,362584118	-0,27407831	2,62E-07	ring finger and CCCH-type d	RC3H2
ENSG00000122557	49,64421492	59,83036343	7,333936165	-0,273886195	4,32E-05	HERPUD family member 2	HERPUD2
ENSG00000101452	10,65663552	12,91087805	5,242600891	-0,273741816	0,00276934	DEAH-box helicase 35	DHX35
ENSG00000141965	48,89409947	59,2339613	7,430189285	-0,273655073	1,76E-05	fem-1 homolog A	FEM1A
ENSG00000126391	76,39246279	92,43907867	7,691361508	-0,273617814	0,007176863	FERM domain containing 8	FRMD8
ENSG00000104231	17,72610301	21,46958022	5,927373166	-0,273511405	0,005287297	zinc finger AN1-type contain	ZFAND1
ENSG00000114742	52,26125215	63,10271243	7,521738484	-0,273483179	5,74E-06	WD repeat domain 48	WDR48
ENSG00000160714	66,60768178	80,5119522	7,821327037	-0,273296284	2,54E-05	ubiquitin conjugating enzym	UBE2Q1
ENSG00000119878	12,81817923	15,53793854	5,479498189	-0,273248567	0,0192037	CXXC repeat containing inte	CRIPT
ENSG00000047578	47,54716719	57,6196334	7,428951851	-0,27319264	0,0056251	katanin interacting protein	KATNIP
ENSG00000076944	45,91262513	55,53656099	7,117592848	-0,272833553	0,003838105	syntaxin binding protein 2	STXBP2
ENSG00000171135	25,21331098	30,50374464	6,350212649	-0,272766433	0,01565531	jagunal homolog 1	JAGN1
ENSG00000119729	59,48494977	71,91532679	7,741778237	-0,272042198	0,015843926	ras homolog family member	RHOQ
ENSG00000102401	88,46275981	106,1416246	8,343205459	-0,271681885	0,000203041	armadillo repeat containing	ARMCX3
ENSG00000061273	223,739429	268,6857304	9,494214946	-0,271204284	0,003994995	histone deacetylase 7	HDAC7
ENSG00000168395	49,24474417	59,6230807	7,278677633	-0,271159507	0,009325593	inhibitor of growth family m	ING5
ENSG00000063176	33,57484436	40,66359159	6,864026179	-0,271152611	0,001253612	sphingosine kinase 2	SPHK2
ENSG00000215030	7,562260762	9,14118254	5,969294126	-0,270750298	0,031496891	ribosomal protein L13 pseuc	RPL13P12
ENSG00000108389	74,82657944	90,44634671	7,926334746	-0,270537519	4,21E-12	myotubularin related protei	MTMR4
ENSG00000198945	12,87742568	15,46924004	5,496860416	-0,270406356	0,008972898	L3MBTL histone methyl-lysir	L3MBTL3
ENSG00000090581	78,474333	94,85827712	8,200899468	-0,270327329	0,000586406	N-acetylglucosamine-1-phos	GNPTG
ENSG00000148300	47,59593115	57,37957435	7,401424767	-0,270155523	0,000113447	REX4 homolog, 3'-5' exonucl	REXO4
ENSG00000107789	54,42884213	65,68612459	7,474027588	-0,27012952	0,00262797	multiple inositol-polyphospl	MINPP1
ENSG00000111641	37,18273357	45,00172064	6,952289941	-0,270093411	0,026022205	NOP2 nucleolar protein	NOP2
ENSG00000021355	25,07947261	30,30962686	6,325151423	-0,269813992	0,028267702	serpin family B member 1	SERPINB1
ENSG00000125650	8,776923838	10,61346389	4,914657171	-0,269353069	0,019736847	persephin	PSPN
ENSG00000132356	65,60782187	79,17605368	7,652341975	-0,269119864	0,00710643	protein kinase AMP-activate	PRKAA1
ENSG00000179195	150,3481068	181,2249057	8,943090844	-0,268843023	3,83E-06	zinc finger protein 664	ZNF664
ENSG00000185127	49,17848064	59,28349902	7,473072808	-0,26850079	0,006900652	chromosome 6 open readin	C6orf120
ENSG00000198042	33,00210752	39,90916011	6,62225879	-0,268365319	0,026322854	MAK16 homolog	MAK16
ENSG00000076067	101,7903649	122,1100327	8,405043186	-0,268281937	0,007811045	RNA binding motif single str	RBMS2
ENSG00000139697	82,67201782	99,78768707	8,133420353	-0,268222394	0,001012913	strawberry notch homolog 1	SBNO1
ENSG00000269556	14,62576264	17,57606896	5,699586136	-0,267860911	0,018005004	transmembrane protein 185	TMEM185A
ENSG00000152102	205,7531702	247,746034	9,387408857	-0,267822004	0,018666418	family with sequence similai	FAM168B
ENSG00000151498	25,68032655	31,0458128	6,493701358	-0,267759129	0,026592803	acyl-CoA dehydrogenase far	ACAD8
ENSG00000156931	36,74268886	44,3607578	7,049792287	-0,267497656	0,001687602	VPS8 subunit of CORVET cor	VPS8
ENSG00000142166	96,60867983	116,5572218	8,297969204	-0,267464531	0,008706426	interferon alpha and beta re	IFNAR1
ENSG00000112146	46,64848808	56,13585015	7,372603245	-0,267439772	0,012510957	F-box protein 9	FBXO9
ENSG00000017260	179,2499561	215,9262436	9,079812282	-0,267394534	0,021376698	ATPase secretory pathway C	ATP2C1
ENSG00000139132	43,20502014	51,61803299	7,203272636	-0,267149841	0,009035271	FYVE, RhoGEF and PH doma	FGD4
ENSG00000147400	35,76263421	43,17093636	7,021855368	-0,267003652	0,032234189	centrin 2	CETN2
ENSG00000175387	98,9424703	119,3111451	8,437432951	-0,266996228	0,000154828	SMAD family member 2	SMAD2
ENSG00000092148	198,3736799	238,9794507	9,374350568	-0,266979729	0,000404904	HECT domain E3 ubiquitin p	HECTD1
ENSG00000168495	47,27170757	56,76153617	7,270715752	-0,266901084	0,000689391	RNA polymerase III subunit I	POLR3D
ENSG00000162402	82,24042641	99,1577079	8,109119207	-0,266814965	0,010800723	ubiquitin specific peptidase	USP24
ENSG00000137992	51,56671216	62,24973449	7,490220446	-0,26670117	0,000632347	dihydrolipoamide branched	DBT
ENSG00000157800	27,81029873	33,30867904	6,5122643	-0,266549941	0,020627132	solute carrier family 37 men	SLC37A3
ENSG00000171206	209,3256588	251,1992178	9,310232588	-0,26629174	0,006611401	tripartite motif containing 8	TRIM8
ENSG00000152291	379,3274965	457,1461994	10,30841765	-0,265992185	0,000286835	trans-golgi network protein	TGOLN2
ENSG00000144566	70,73292383	84,83923207	7,791771504	-0,265627361	3,59E-05	RAB5A, member RAS oncoge	RAB5A
ENSG00000131043	39,36833996	47,36821434	7,068612992	-0,265083295	0,000425073	AAR2 splicing factor	AAR2
ENSG00000116584	127,2119627	153,0016567	8,624582065	-0,265049403	0,00380809	Rho/Rac guanine nucleotide	ARHGEF2
ENSG00000168288	45,77435825	55,08215969	7,214549614	-0,265039644	2,58E-06	metabolism of cobalamin as	MMADHC
ENSG00000124702	87,60722736	105,3755986	8,153485997	-0,26456824	0,006522566	kelch domain containing 3	KLHDC3
ENSG00000180228	44,14510927	53,13643263	7,148152494	-0,26436399	7,51E-06	protein activator of interfer	PRKRA
ENSG00000082458	42,59607523	50,6959557	6,911993988	-0,264115382	0,045398437	discs large MAGUK scaffold	DLG3
ENSG00000196922	29,51481295	35,51363943	6,930687802	-0,264073236	0,001528805	zinc finger protein 252, pseu	ZNF252P
ENSG00000104731	37,4614963	45,09438305	7,022495082	-0,263564751	0,004090778	kelch domain containing 4	KLHDC4
ENSG00000096717	34,08267764	41,00008674	6,631170753	-0,263552478	0,004486773	sirtuin 1	SIRT1
ENSG00000204138	115,6845463	139,0286375	8,351560196	-0,263414474	0,001971873	phosphatase and actin regul	PHACTR4
ENSG00000138279	81,63094307	98,19068342	8,049815162	-0,263302108	0,000256191	annexin A7	ANXA7
ENSG00000114784	39,91657313	48,01741399	7,057618726	-0,263266776	0,007308284	eukaryotic translation initiat	EIF1B
ENSG00000147471	51,91624844	62,44389061	7,373624265	-0,263164265	0,004072496	pyridoxal phosphate binding	PLPBP
ENSG00000163945	16,83208584	20,22410726	5,932798391	-0,262590272	0,047030446	UV stimulated scaffold prote	UVSSA
ENSG00000166295	62,87831098	75,45109243	7,736226738	-0,262275576	6,05E-05	anaphase promoting compl	ANAPC16
ENSG00000173486	74,54452097	89,63972442	8,013681323	-0,262272407	0,019789945	FKBP prolyl isomerase 2	FKBP2
ENSG00000124299	73,23668829	87,97893086	8,08153404	-0,262247213	0,001064558	peptidase D	PEPD
ENSG00000196850	27,16257544	32,64756964	6,487294592	-0,26218715	0,033982026	protein phosphatase targeti	PPTC7
ENSG00000233927	96,16390785	114,9191323	8,463266358	-0,262024976	0,004579444	ribosomal protein S28	RPS28
ENSG00000186908	27,23216686	32,67828652	6,599258433	-0,261970231	0,021354763	zinc finger DHHC-type palmi	ZDHHC17
ENSG00000136854	91,94032207	110,5207236	8,096014528	-0,261704753	0,015418764	syntaxin binding protein 1	STXBP1
ENSG00000183354	45,09670052	54,13318939	7,199397314	-0,261686642	0,000725763	KIAA2026	KIAA2026
ENSG00000154222	85,68250748	102,9750938	8,21846487	-0,261510116	1,76E-05	coiled-coil and C2 domain cc	CC2D1B
ENSG00000180488	37,69130633	45,26566118	6,93238794	-0,261447791	0,000321177	mitoguardin 1	MIGA1
ENSG00000173757	61,31370835	73,62591929	7,512382227	-0,261219704	0,018226868	signal transducer and activa	STAT5B
ENSG00000069509	16,4062243	19,41045123	5,565648445	-0,26115396	0,0265534	FUN14 domain containing 1	FUNDC1
ENSG00000068097	34,46312632	41,43039375	6,872124752	-0,261093817	0,005589962	HEAT repeat containing 6	HEATR6
ENSG00000169599	25,99038811	31,23698532	6,353802911	-0,261083465	0,00126037	NFU1 iron-sulfur cluster scal	NFU1
ENSG00000101246	44,01151284	52,62778426	7,347446349	-0,260991675	0,001247333	ADP ribosylation factor relat	ARFRP1
ENSG00000138802	66,83030152	80,21158612	7,790457068	-0,260980364	0,00055661	SEC24 homolog B, COPII coa	SEC24B
ENSG00000137269	23,54950722	28,26072666	6,006778922	-0,260979861	0,007758997	leucine rich repeat containir	LRRC1
ENSG00000187239	59,39630726	71,02776433	7,440762683	-0,260925984	0,006965781	formin binding protein 1	FNBP1
ENSG00000112983	50,73516211	60,90108734	7,444071916	-0,260825505	0,001584606	bromodomain containing 8	BRD8
ENSG00000133703	51,54030687	61,83553524	7,271944636	-0,260522874	0,002241716	KRAS proto-oncogene, GTPa	KRAS
ENSG00000130312	44,49405307	53,41789072	7,189455423	-0,260126207	0,004659477	mitochondrial ribosomal prc	MRPL34
ENSG00000108061	44,10401498	52,890983	7,208114257	-0,260017676	7,22E-05	SHOC2 leucine rich repeat sc	SHOC2
ENSG00000105726	97,10635464	116,4341277	8,358038895	-0,260013668	2,35E-06	ATPase 13A1	ATP13A1
ENSG00000178988	45,20077783	53,79090649	7,286200534	-0,25936713	0,006947762	Morf4 family associated pro	MRFAP1L1
ENSG00000165733	76,99851028	92,36419342	7,926971286	-0,259169896	0,002365501	BMS1 ribosome biogenesis f	BMS1
ENSG00000164346	68,58726193	81,84434735	7,908995163	-0,259083717	0,008889951	NSA2 ribosome biogenesis f	NSA2
ENSG00000132768	22,78841487	27,32902583	6,338884461	-0,259045519	0,044225701	diphthamide biosynthesis 2	DPH2
ENSG00000148218	56,1295205	66,93853032	7,52568361	-0,258529942	0,015534069	aminolevulinate dehydratas	ALAD
ENSG00000007168	169,5990066	202,6829601	9,073859228	-0,258516731	1,03E-08	platelet activating factor ace	PAFAH1B1
ENSG00000107185	115,5659265	138,3668668	8,612593721	-0,25845338	4,40E-05	RGP1 homolog, RAB6A GEF	RGP1
ENSG00000161920	13,30425804	15,94679515	5,536405058	-0,258184376	0,009199756	mediator complex subunit 1	MED11
ENSG00000076108	206,9672292	247,6770745	9,45761274	-0,257993184	0,003388833	bromodomain adjacent to zi	BAZ2A
ENSG00000142188	35,05068969	42,01755985	6,86313976	-0,257801184	0,003572693	transmembrane protein 50E	TMEM50B
ENSG00000143776	140,1551711	167,0889881	8,756447419	-0,257440206	0,04902536	CDC42 binding protein kinas	CDC42BPA
ENSG00000072401	34,98381186	41,85989475	6,723595024	-0,257328896	0,040775722	ubiquitin conjugating enzym	UBE2D1
ENSG00000133316	26,3453413	31,55914588	6,430741051	-0,257196966	0,009325593	WD repeat domain 74	WDR74
ENSG00000138385	99,53160648	119,2697753	8,333291478	-0,257073358	0,004265413	small RNA binding exonucle:	SSB
ENSG00000069966	54,27420578	64,78297351	7,331236722	-0,256989561	0,02335808	G protein subunit beta 5	GNB5
ENSG00000075785	294,802015	352,7322324	9,883958648	-0,256975293	4,42E-05	RAB7A, member RAS oncoge	RAB7A
ENSG00000115211	38,10229397	45,63777203	7,063450286	-0,256932248	0,000152879	eukaryotic translation initiat	EIF2B4
ENSG00000080371	87,84556821	105,1916836	8,207023678	-0,256877626	3,43E-05	RAB21, member RAS oncoge	RAB21
ENSG00000119402	94,95813185	113,8175005	8,311644728	-0,256783473	0,001165078	F-box and WD repeat domai	FBXW2
ENSG00000085760	35,08145696	42,01022417	6,915089822	-0,256757845	7,28E-06	mitochondrial translational i	MTIF2
ENSG00000143612	118,2765921	141,4528931	8,672305368	-0,256476995	0,000102773	chromosome 1 open readin	C1orf43
ENSG00000101152	131,7767983	157,3777515	8,681257903	-0,256392559	0,00067427	DnaJ heat shock protein fam	DNAJC5
ENSG00000197746	960,5106545	1141,984359	11,7833966	-0,256321056	0,014162505	prosaposin	PSAP
ENSG00000177707	64,25851126	76,98936212	7,571819835	-0,256083882	0,009055749	nectin cell adhesion molecul	NECTIN3
ENSG00000180879	71,2906546	84,61040586	8,028174233	-0,255716354	0,035915187	signal sequence receptor su	SSR4
ENSG00000105341	64,55978378	77,27657466	7,74305142	-0,255666336	0,00236072	distal membrane arm assem	DMAC2

ENSG00000100129	118,658772	141,7165299	8,635456142	-0,255336035	0,03274155	eukaryotic translation initiat	EIF3L
ENSG00000088367	78,88759937	92,80341091	8,06835221	-0,255303323	0,00710643	erythrocyte membrane prot	EPB41L1
ENSG00000100580	58,51656762	70,07837314	7,510340848	-0,255221445	0,026910007	transmembrane p24 traffick	TMED8
ENSG00000155100	15,5845271	18,65008915	5,499755072	-0,255214234	0,023686609	OTU deubiquitinase 6B	OTUD6B
ENSG00000188636	75,68176691	90,07757732	8,078893846	-0,254977113	0,02430925	retrotransposon Gag like 6	RTL6
ENSG00000107581	422,1856239	504,824125	10,42114382	-0,254694173	1,75E-05	eukaryotic translation initiat	EIF3A
ENSG00000112893	138,2389815	165,4435404	8,899370915	-0,254594726	0,040821375	mannosidase alpha class 2A	MAN2A1
ENSG00000160953	69,72388148	83,50763557	7,882195162	-0,254548655	0,028446005	PWWP domain containing 3	PWWP3A
ENSG00000136807	71,12329665	84,90201079	7,798245368	-0,254415222	1,64E-10	cyclin dependent kinase 9	CDK9
ENSG00000005812	42,53755927	50,84543421	7,083961055	-0,25433819	0,00397365	F-box and leucine rich repea	FBXL3
ENSG00000111450	46,41715738	55,49557703	7,108735893	-0,254165196	0,007232246	syntaxin 2	STX2
ENSG00000066583	24,57118487	29,40879365	6,160582837	-0,254032505	0,019761328	isochorismatase domain cor	ISOC1
ENSG00000089876	42,14240699	50,07844844	7,041510685	-0,253805211	0,040914126	DEAH-box helicase 32 (putat	DHX32
ENSG00000095794	11,28572596	13,47532923	5,293071078	-0,253605828	0,018811196	cAMP responsive element m	CREM
ENSG00000149823	58,79832956	70,17818285	7,637723712	-0,253502155	0,048451523	VPS51 subunit of GARP com	VPS51
ENSG00000165406	38,12649669	45,48322711	7,172998559	-0,253423801	0,027074454	membrane associated ring-C	MARCHF8
ENSG00000173875	28,55820802	34,11340301	6,692456893	-0,253260298	0,003419809	zinc finger protein 791	ZNF791
ENSG00000126653	29,38993593	35,12510518	6,51761436	-0,253151396	0,022321616	nuclear speckle splicing regu	NSRP1
ENSG00000139644	625,0761913	747,8935474	10,97315382	-0,253091905	0,008889951	transmembrane BAX inhibito	TMBIM6
ENSG00000120008	77,81702418	92,94467042	8,01322903	-0,253007702	6,02E-07	WD repeat domain 11	WDR11
ENSG00000168092	107,31119	128,0136163	8,416486454	-0,252929733	0,000113759	platelet activating factor ace	PAFAH1B2
ENSG00000184863	70,93333768	84,72498194	7,885287223	-0,25283478	0,039696407	RNA binding motif protein 3	RBM33
ENSG00000081692	20,71958171	24,66097501	6,178536032	-0,252745486	0,002915149	jumonji domain containing 4	JMJD4
ENSG00000103769	140,2324941	167,5412138	8,797280791	-0,252741258	0,013162042	RAB11A, member RAS oncog	RAB11A
ENSG00000116604	71,89788293	85,80615755	7,985557754	-0,252529689	0,000424375	myocyte enhancer factor 2D	MEF2D
ENSG00000005302	36,93523032	43,88931163	7,008594102	-0,252515964	0,006765885	MSL complex subunit 3	MSL3
ENSG00000153006	28,82941691	34,39327993	6,566915057	-0,252506866	0,000240781	SREK1 interacting protein 1	SREK1IP1
ENSG00000182544	28,48813957	34,00249384	6,635611469	-0,252229451	0,009829853	major facilitator superfamily	MFSD5
ENSG00000167658	2246,766426	2677,3272	12,99434378	-0,252155966	0,008242848	eukaryotic translation elong	EEF2
ENSG00000163719	29,72973233	35,44362037	6,709070249	-0,251970567	0,000124316	myotubularin related protei	MTMR14
ENSG00000170871	76,95931563	91,44363291	7,964222655	-0,251862399	0,006419018	KIAA0232	KIAA0232
ENSG00000119616	37,41593404	44,49788301	6,995620895	-0,25172822	0,000434019	FCF1 rRNA-processing prote	FCF1
ENSG00000096080	30,72817903	36,65942512	6,675373289	-0,251495996	0,004659477	mitochondrial ribosomal pro	MRPS18A
ENSG00000122033	19,2183374	22,90856398	6,094193002	-0,251356144	0,000169612	mitochondrial translational i	MTIF3
ENSG00000130559	78,92954881	94,0062293	7,90728621	-0,251247061	0,001823225	calmodulin regulated spectr	CAMSAP1
ENSG00000083168	118,6070494	141,3839056	8,513435063	-0,251193651	0,001470439	lysine acetyltransferase 6A	KAT6A
ENSG00000167447	25,25371144	30,01074919	6,370479291	-0,250873257	0,000181298	SMG8 nonsense mediated m	SMG8
ENSG00000167515	23,09651449	27,51242143	6,457729961	-0,250823865	0,01398657	trafficking protein particle co	TRAPPC2L
ENSG00000157259	53,98535228	64,39592666	7,485526292	-0,250822415	0,000465969	GATA zinc finger domain cor	GATAD1
ENSG00000163947	31,64925449	37,1674193	6,895479187	-0,250775241	0,009835011	Rho guanine nucleotide excl	ARHGEF3
ENSG00000187051	39,80954344	47,45613073	7,154266699	-0,250570225	0,002580927	ribosomal protein S19 bindi	RPS19BP1
ENSG00000108788	40,84889554	48,70079636	7,077795872	-0,250394506	0,000216167	MAX dimerization protein M	MLX
ENSG00000117020	110,3587064	131,5813012	8,416688856	-0,250316807	0,004137002	AKT serine/threonine kinase	AKT3
ENSG00000163848	71,13558402	84,49938204	7,924609826	-0,250241259	1,79E-05	zinc finger protein 148	ZNF148
ENSG00000235173	26,87115784	32,02803775	6,495170925	-0,250062581	0,039118606	HGH1 homolog	HGH1
ENSG00000133961	71,77256469	85,26475418	7,794694769	-0,249731391	0,006542732	NUMB endocytic adaptor pr	NUMB
ENSG00000142751	39,42207029	46,91155386	7,045206807	-0,249261092	1,41E-06	GPN-loop GTPase 2	GPN2
ENSG00000177156	139,4710336	166,2524979	8,831589161	-0,249228248	0,013547505	transaldolase 1	TALDO1
ENSG00000154114	35,11436872	41,83626518	6,889275553	-0,24909979	2,89E-05	tubulin folding cofactor E lik	TBCEL
ENSG00000147419	41,69754049	49,7152311	7,071650049	-0,248949614	0,003602476	coiled-coil domain containin	CCDC25
ENSG00000164219	27,54454722	32,81827874	6,462877443	-0,248849295	0,017035533	protein geranylgeranyltrans	PGGT1B
ENSG00000112031	21,12440284	25,13194373	5,978261984	-0,248839431	0,017398103	mitochondrial translation re	MTRF1L
ENSG00000104408	163,4393432	194,0924569	9,157026389	-0,248756085	0,025213517	eukaryotic translation initiat	EIF3E
ENSG00000092439	86,67360701	103,3337988	8,123545655	-0,248366745	0,000237072	transient receptor potential	TRPM7
ENSG00000160075	92,46868374	110,0540999	8,284294606	-0,248328917	0,000333071	SSU72 homolog, RNA polym	SSU72
ENSG00000173402	226,0058746	268,2932802	9,349772717	-0,248248284	0,049628218	dystroglycan 1	DAG1
ENSG00000113312	40,45405453	48,09665642	7,174934005	-0,248233273	0,000914691	tetratricopeptide repeat dor	TTC1
ENSG00000127666	43,10483566	51,11763776	6,994628172	-0,24817089	0,006108651	toll like receptor adaptor mo	TICAM1
ENSG00000049245	102,9902424	122,4518088	8,46585077	-0,247920473	0,000581421	vesicle associated membran	VAMP3
ENSG00000083520	64,6408484	76,72789924	7,673208138	-0,247641565	0,000431607	DIS3 homolog, exosome enc	DIS3
ENSG00000122068	75,10919275	89,14395757	7,973097537	-0,247500617	0,000108104	forty-two-three domain con	FYTTD1
ENSG00000089916	65,82640751	78,21558781	7,84838852	-0,247363897	0,004136706	G-patch domain containing 2	GPATCH2L
ENSG00000008952	127,8310843	152,0129562	8,817817725	-0,247227893	0,000597797	SEC62 homolog, preprotein	SEC62
ENSG00000139645	243,9644883	289,3305431	9,699724207	-0,247211243	0,004422946	ankyrin repeat domain 52	ANKRD52
ENSG00000164117	17,06220523	20,27878051	5,852979148	-0,247053139	0,017318215	F-box protein 8	FBXO8
ENSG00000115998	14,06023262	16,71411233	5,553909745	-0,246760349	0,000447222	chromosome 2 open reading	C2orf42
ENSG00000126070	38,55276906	45,83175082	6,969478665	-0,246217127	0,003376469	argonaute RISC catalytic con	AGO3
ENSG00000135452	29,58393031	35,06729212	6,707088362	-0,24620346	0,001569681	tetraspanin 31	TSPAN31
ENSG00000018510	123,6959309	146,717955	8,572456319	-0,246016554	0,004028949	alkylglycerone phosphate sy	AGPS
ENSG00000130304	27,54917108	32,62449464	6,818683273	-0,245820407	0,004887213	solute carrier family 27 mem	SLC27A1
ENSG00000163605	65,69435981	77,97605234	7,720073722	-0,245818874	7,22E-05	protein phosphatase 4 regul	PPP4R2
ENSG00000075292	129,1783433	153,4944969	8,708950592	-0,245628591	0,00584646	zinc finger protein 638	ZNF638
ENSG00000157326	19,48123557	23,15845214	6,224640745	-0,245384262	0,040339353	dehydrogenase/reductase 4	DHRS4
ENSG00000148429	29,74316839	35,02302869	6,514801908	-0,2452101	0,001070271	USP6 N-terminal like	USP6NL
ENSG00000204387	50,25453073	59,64045469	7,470848371	-0,245195039	0,029517165	small nucleolar RNA host ge	SNHG32
ENSG00000177830	109,9929001	130,2378461	8,507476763	-0,245163802	0,009521811	chitinase domain containing	CHID1
ENSG00000170248	181,2799324	215,2157542	9,240238333	-0,245067247	1,05E-08	programmed cell death 6 int	PDCD6IP
ENSG00000059691	29,3484643	34,87613791	6,71092271	-0,244979369	0,013158743	glutamyl-tRNA amidotransfe	GATB
ENSG00000179151	39,55368061	46,92474541	7,089187187	-0,244927505	0,000335128	enhancer of mRNA decappir	EDC3
ENSG00000152464	11,93023912	14,16376935	5,331229617	-0,244806291	0,034695276	ribonuclease P/MRP subunit	RPP38
ENSG00000168434	18,49609447	21,8832354	6,049386511	-0,244683583	0,026573226	component of oligomeric go	COG7
ENSG00000183605	8,000061029	9,488491178	4,967930091	-0,244659608	0,04197983	sideroflexin 4	SFXN4
ENSG00000144445	18,21831486	21,66351294	5,901156427	-0,244627504	0,014245305	KAT8 regulatory NSL comple	KANSL1L
ENSG00000139977	37,99735899	45,03973542	7,002593173	-0,244386579	2,39E-05	N-alpha-acetyltransferase 30	NAA30
ENSG00000132406	11,12589733	13,20911855	5,372526646	-0,243919221	0,019479039	transmembrane protein 128	TMEM128
ENSG00000181315	10,59498199	12,56655632	5,300225911	-0,24374704	0,012589313	zinc finger protein 322	ZNF322
ENSG00000167113	44,47194237	52,73884869	7,286055991	-0,243135756	0,007962841	coenzyme Q4	COQ4
ENSG00000134970	57,04177692	67,56852889	7,540950148	-0,243047501	0,040715965	transmembrane p24 traffick	TMED7
ENSG00000106086	32,845238	38,85931133	6,767314981	-0,242960568	0,000257524	pleckstrin homology domair	PLEKHA8
ENSG00000254598	10,65861505	12,63173953	5,165216443	-0,242911118	0,005006243	casein kinase 2 alpha 3	CSNK2A3
ENSG00000158864	66,47045495	78,85411518	7,879322144	-0,242778146	0,005914771	NADH:ubiquinone oxidored	NDUFS2
ENSG00000052841	80,54703582	95,27340217	8,10682145	-0,242670074	0,002500433	tetratricopeptide repeat dor	TTC17
ENSG00000138095	179,6622716	213,4705746	9,168897937	-0,242617304	0,009743623	leucine rich pentatricopepti	LRPPRC
ENSG00000197982	56,35453746	66,57628411	7,552776117	-0,242453261	0,033171701	chromosome 1 open reading	C1orf122
ENSG00000107021	53,02123691	62,62502062	7,553981833	-0,242237047	0,001359886	TBC1 domain family membe	TBC1D13
ENSG00000170142	56,99649398	67,27025157	7,543746695	-0,242161788	0,007548524	ubiquitin conjugating enzym	UBE2E1
ENSG00000131849	6,472860668	7,676630598	4,333261125	-0,242035642	0,033510228	zinc finger protein 132	ZNF132
ENSG00000084733	180,4534326	213,7655274	9,187622579	-0,241812971	0,000173892	RAB10, member RAS oncoge	RAB10
ENSG00000132254	34,5571327	40,87378838	6,964156877	-0,241714735	0,00946808	ADP ribosylation factor inter	ARFIP2
ENSG00000164576	45,91312879	54,40837568	7,223576715	-0,241714394	0,004835497	SAP30 like	SAP30L
ENSG00000135090	54,31804775	64,38437081	7,385501549	-0,241672945	3,67E-05	TAO kinase 3	TAOK3
ENSG00000205060	34,54440935	40,90407503	6,771921208	-0,241482653	0,013314453	solute carrier family 35 mem	SLC35B4
ENSG00000163820	103,3072607	122,155336	8,60489846	-0,241423768	0,003914841	FYVE and coiled-coil domain	FYCO1
ENSG00000168884	55,03765364	65,20651873	7,538335319	-0,241342539	8,65E-07	TNFAIP3 interacting protein	TNIP2
ENSG00000100029	75,20059807	88,98044485	7,984967363	-0,240649543	0,000913658	pescadillo ribosomal biogen	PES1
ENSG00000186416	19,87351587	23,53288253	6,035313303	-0,240648177	0,002781319	NFKB repressing factor	NKRF
ENSG00000156990	25,57375135	30,26147337	6,480836138	-0,24029047	0,021547631	RNA pseudouridine synthase	RPUSD3
ENSG00000131626	81,34652054	96,22129061	8,165590733	-0,240152591	0,023421261	PTPRF interacting protein al	PPFIA1
ENSG00000099330	26,9410408	31,87751022	6,670490877	-0,239783778	0,018832703	occludin/ELL domain contai	OCEL1
ENSG00000214113	16,39408728	19,37792012	5,854800109	-0,239642518	0,032115729	LYR motif containing 4	LYRM4
ENSG00000115020	61,49014745	72,7769751	7,627550225	-0,239600864	0,003961884	phosphoinositide kinase, FY	PIKFYVE
ENSG00000175348	38,01166022	44,83581467	6,938711363	-0,239309598	0,004237964	TMEM9 domain family mem	TMEM9B
ENSG00000163684	12,74229753	15,0612965	5,48344684	-0,239303777	0,032222534	ribonuclease P/MRP subunit	RPP14
ENSG00000166260	28,48160532	33,68275239	6,668759033	-0,239273321	0,045995847	cytochrome c oxidase coppe	COX11
ENSG00000165699	61,20044463	72,38850902	7,811751013	-0,239213202	6,65E-06	TSC complex subunit 1	TSC1

ENSG00000137996	32,15946832	38,02888703	6,731437087	-0,239185665	6,64E-05	RNA 3'-terminal phosphate	RTCA
ENSG00000198707	41,90534373	49,60689934	7,115408229	-0,239093859	0,017494379	centrosomal protein 290	CEP290
ENSG00000236104	26,56570346	31,26231033	6,627194899	-0,238971258	0,00018895	zinc finger and BTB domain	ZBTB22
ENSG00000249915	60,58155824	71,65880197	7,658904456	-0,238901028	9,32E-05	programmed cell death 6	PDCD6
ENSG00000107263	137,2997346	162,3071211	8,709541312	-0,238829742	0,009407083	Rap guanine nucleotide exch	RAPGEF1
ENSG00000164031	43,19883164	51,03931443	7,109774122	-0,238737437	0,030132165	DnaJ heat shock protein fam	DNAJB14
ENSG00000117543	14,81117821	17,53234427	5,819658892	-0,238725145	0,013996785	diphthamide biosynthesis 5	DPH5
ENSG00000181704	52,6095696	62,15382022	7,415240734	-0,238633312	0,002607706	Yip1 domain family member	YIPF6
ENSG00000107959	79,95858117	94,48662955	8,033383345	-0,238295088	0,011397779	pitrilysin metallopeptidase 1	PITRM1
ENSG00000085415	37,09558215	43,8432479	6,781738051	-0,238275196	0,000676777	SEH1 like nucleoporin	SEH1L
ENSG00000133243	203,9723013	240,7254889	9,386491576	-0,238221799	0,000743539	BTB domain containing 2	BTBD2
ENSG00000115970	41,42086401	48,91818843	7,110985782	-0,238088982	0,003109814	THADA armadillo repeat cor	THADA
ENSG00000131408	75,56847764	89,22648019	8,045046858	-0,237900525	3,90E-06	nuclear receptor subfamily 1	NR1H2
ENSG00000117298	590,2411964	696,6111373	10,88421858	-0,237832792	0,035666514	endothelin converting enzym	ECE1
ENSG00000115762	112,4108492	132,863764	8,57376727	-0,237757551	0,003067458	pleckstrin homology domair	PLEKHB2
ENSG00000103066	61,58201143	72,63922608	7,751037072	-0,237702527	0,010764215	phospholipase A2 group XV	PLA2G15
ENSG00000168283	17,24320618	20,34898467	5,968967318	-0,237624152	0,012668233	BMI1 proto-oncogene, polyc	BMI1
ENSG00000158850	39,90117349	47,18753008	7,074118009	-0,237555049	0,025137698	beta-1,4-galactosyltransfera	B4GALT3
ENSG00000047056	33,08640081	39,04858359	6,752929368	-0,23745799	0,012331853	WD repeat domain 37	WDR37
ENSG00000007047	82,48756298	97,29101019	8,088478397	-0,23737511	0,007198523	microtubule affinity regulati	MARK4
ENSG00000146223	155,6547968	183,7124485	9,045464288	-0,23722719	5,76E-05	ribosomal protein L7 like 1	RPL7L1
ENSG00000106299	86,02181112	100,6644488	8,028792094	-0,236801605	2,32E-05	WASP like actin nucleation p	WASL
ENSG00000204568	36,07713527	42,52310664	6,973074776	-0,236787366	0,009385884	mitochondrial ribosomal pro	MRPS18B
ENSG00000087086	811,7348288	956,1306113	11,55696909	-0,236306475	0,001840642	ferritin light chain	FTL
ENSG00000198833	75,9198208	89,28576441	7,932522041	-0,23622246	0,001591024	ubiquitin conjugating enzym	UBE2J1
ENSG00000145348	44,27953745	52,20222944	7,183011944	-0,23621981	0,006279527	TBC1 domain containing kin	TBCK
ENSG00000198198	71,6710033	84,51010679	7,998980221	-0,236195924	0,038672856	SZT2 subunit of KICSTOR cor	SZT2
ENSG00000170632	36,63509423	43,18745175	6,931699563	-0,235961044	0,043528118	armadillo repeat containing	ARMC10
ENSG00000250317	20,04685773	23,621572	6,193880268	-0,235889877	0,009914343	small integral membrane pro	SMIM20
ENSG00000083838	15,52906251	18,30855701	5,799713894	-0,235785622	0,014161716	zinc finger protein 446	ZNF446
ENSG00000111737	72,80038618	85,90831394	7,783710741	-0,235756097	0,000264544	RAB35, member RAS oncoge	RAB35
ENSG00000165684	36,94897064	43,62981517	6,937965828	-0,235717968	0,032292567	small nuclear RNA activating	SNAPC4
ENSG00000123415	25,45402661	29,92189514	6,451171024	-0,235532052	0,049916355	single-strand-selective mono	SMUG1
ENSG00000130787	84,36193037	98,66931007	7,808383449	-0,23500755	0,030679438	huntingtin interacting prote	HIP1R
ENSG00000131943	23,46213883	27,53500678	6,390919845	-0,234871459	0,031452612	chromosome 19 open readi	C19orf12
ENSG00000140497	93,88134049	110,6685264	8,282796919	-0,234696299	0,00019551	secretory carrier membrane	SCAMP2
ENSG00000134698	23,27363113	27,32028623	6,150775751	-0,234594931	0,043797278	argonaute RISC component	AGO4
ENSG00000197362	16,55569991	19,54191099	5,856624249	-0,234177588	0,039799594	zinc finger protein 786	ZNF786
ENSG00000065183	38,68722046	45,60534333	7,021006005	-0,234112633	0,015084448	WD repeat domain 3	WDR3
ENSG00000185122	113,1499798	133,0463205	8,470015102	-0,233983343	1,64E-05	heat shock transcription fac	HSF1
ENSG00000068120	81,48660119	96,01914159	8,099403885	-0,233972441	0,000382951	Coenzyme A synthase	COASY
ENSG00000198925	119,0539816	140,3262331	8,769854681	-0,233965034	0,005945569	autophagy related 9A	ATG9A
ENSG00000175073	42,77852306	50,42023466	7,190404688	-0,233899313	0,006771	valosin containing protein ir	VCPIP1
ENSG00000162409	34,81694828	41,12245871	6,750248303	-0,233898128	0,028944799	protein kinase AMP-activate	PRKAA2
ENSG00000129933	70,55697176	82,96236703	7,94616655	-0,23368936	3,59E-05	MAU2 sister chromatid cohe	MAU2
ENSG00000171109	38,9924166	45,94435737	6,915763512	-0,23364669	0,018811196	mitofusin 1	MFN1
ENSG00000128654	35,73455247	42,1031123	6,797679565	-0,233483948	0,006278614	metaxin 2	MTX2
ENSG00000182195	45,65180215	52,90324912	7,539209886	-0,233355017	0,025048754	LDOC1 regulator of NFKB sig	LDOC1
ENSG00000165097	37,17203386	43,7969839	6,940325238	-0,233300986	0,003228221	lysine demethylase 1B	KDM1B
ENSG00000085382	24,00416712	28,32086099	6,187205764	-0,233299154	0,017776195	HECT domain and ankyrin re	HACE1
ENSG00000082641	431,4938357	504,7732654	10,49853906	-0,233189909	0,000823433	NFE2 like bZIP transcription	NFE2L1
ENSG00000005436	12,021684	14,16814193	4,935744588	-0,233176249	0,022057387	GC-rich sequence DNA-bindi	GCFC2
ENSG00000171475	89,50604503	105,3727303	8,237858719	-0,233174698	0,010910872	WAS/WASL interacting prot	WIPF2
ENSG00000182768	44,93804649	52,73404811	7,23700433	-0,232841224	0,005721281	neugrin, neurite outgrowth	NGRN
ENSG00000166166	19,24112299	22,66742807	6,142630612	-0,232723343	0,025213517	tRNA methyltransferase 61A	TRMT61A
ENSG00000167862	16,37226373	19,24633561	5,742012042	-0,232713226	0,013387879	mitochondrial ribosomal pro	MRPL58
ENSG00000127580	28,28793977	33,11419817	6,608625259	-0,232711963	0,020202447	WD repeat domain 24	WDR24
ENSG00000074603	47,70950534	56,16107277	7,347709871	-0,232594888	0,000772707	dipeptidyl peptidase 8	DPP8
ENSG00000131475	60,88019248	71,60350745	7,640705972	-0,232424215	0,005064177	vacuolar protein sorting 25 l	VPS25
ENSG00000196715	74,65771519	87,61911403	7,801096586	-0,231900029	0,015087786	vitamin K epoxide reductase	VKORC1L1
ENSG00000272333	107,2730453	126,1471808	8,498801754	-0,231838391	0,018666418	lysine methyltransferase 2B	KMT2B
ENSG00000197045	61,67289789	72,48181273	7,614731048	-0,231747631	0,019366072	glia maturation factor beta	GMFB
ENSG00000084623	125,3020442	147,1335835	8,722402133	-0,231703185	0,002529924	eukaryotic translation initiat	EIF3I
ENSG00000079332	141,3504394	166,2670948	8,746422729	-0,231096083	0,012510957	secretion associated Ras rel	SAR1A
ENSG00000111269	43,12624885	50,76205346	7,200100013	-0,231079647	0,013243439	cAMP responsive element b	CREBL2
ENSG00000178951	126,5581895	148,8120956	8,684687304	-0,231037516	0,025150525	zinc finger and BTB domain	ZBTB7A
ENSG00000197885	8,724495144	10,24849501	4,976159616	-0,230864942	0,03730836	NFKB inhibitor interacting Ra	NKIRAS1
ENSG00000116005	201,4600732	236,9821646	9,339240407	-0,230840721	0,041201936	prenylcysteine oxidase 1	PCYOX1
ENSG00000138433	23,8618673	28,04275851	6,427537705	-0,230632632	0,013518704	corepressor interacting with	CIR1
ENSG00000164080	56,04761207	65,81940235	7,337426388	-0,230359809	0,002416812	RAD54 like 2	RAD54L2
ENSG00000132964	25,71329679	30,22591552	6,482619267	-0,229981633	0,008654297	cyclin dependent kinase 8	CDK8
ENSG00000122417	28,14670836	33,10044221	6,583063961	-0,229909813	0,007083724	outer dense fiber of sperm t	ODF2L
ENSG00000234851	13,48267165	15,86126726	5,532492856	-0,229811848	0,023381137	ribosomal protein L23a pseu	RPL23AP42
ENSG00000167987	30,4275625	35,73551462	6,745185539	-0,229783667	0,002429654	VPS37C subunit of ESCRT-I	VPS37C
ENSG00000071051	80,25043358	93,96412558	7,900634549	-0,229652497	0,017154398	NCK adaptor protein 2	NCK2
ENSG00000122741	50,44660092	59,19730451	7,432523199	-0,229402594	0,001592468	DDB1 and CUL4 associated f	DCAF10
ENSG00000164073	9,056402116	10,6288315	4,840656131	-0,229169418	0,017422742	major facilitator superfamily	MFSD8
ENSG00000101150	160,7778255	188,8907931	9,039199255	-0,229123235	0,000830154	TPD52 like 2	TPD52L2
ENSG00000072518	76,81552619	90,15413026	7,917713374	-0,228859672	0,000425907	microtubule affinity regulati	MARK2
ENSG00000179387	29,89396581	35,08880542	6,645286614	-0,228769047	0,042235959	ELMO domain containing 2	ELMOD2
ENSG00000089248	194,6187773	228,6450393	9,305906372	-0,228733581	0,001725854	endoplasmic reticulum prote	ERP29
ENSG00000065613	100,879088	118,5182384	8,180852004	-0,228724139	0,041400102	STE20 like kinase	SLK
ENSG00000182149	118,3024701	138,6983218	8,635647335	-0,228717738	0,000175013	IST1 factor associated with E	IST1
ENSG00000160256	28,34178393	33,2580503	6,541458381	-0,228640837	0,01470248	SLX9 ribosome biogenesis fa	SLX9
ENSG00000169871	226,8264364	266,3523016	9,537123293	-0,228628425	0,008902956	tripartite motif containing 5	TRIM56
ENSG00000122406	305,0125219	356,8523146	10,03477341	-0,228619584	0,008957196	ribosomal protein L5	RPL5
ENSG00000277258	84,070619	97,9242139	8,047968342	-0,228598818	0,011928955	polycomb group ring finger 2	PCGF2
ENSG00000114209	29,11226031	34,1688542	6,486050284	-0,228565101	0,018727997	programmed cell death 10	PDCD10
ENSG00000121680	15,57228759	18,27318199	5,820465425	-0,228490704	0,005389543	peroxisomal biogenesis fact	PEX16
ENSG00000131381	37,7154049	44,12131993	6,9965939	-0,228477988	0,032771107	rabenosyn, RAB effector	RBSN
ENSG00000272886	35,43691881	41,57381302	6,840396575	-0,228371977	0,027160034	decapping mRNA 1A	DCP1A
ENSG00000116198	51,44128096	60,40818751	7,400200553	-0,228268992	0,002595366	centrosomal protein 104	CEP104
ENSG00000253797	38,61391787	45,28604083	7,02538969	-0,227995327	0,001080224	UTP14C small subunit proce	UTP14C
ENSG00000144043	67,21692867	78,66881914	7,858597605	-0,227916361	0,020940497	testis expressed 261	TEX261
ENSG00000106400	50,91117507	59,74503257	7,498495553	-0,227772348	0,001157152	zinc finger HIT-type containi	ZNHIT1
ENSG00000169410	78,09521771	91,01905856	7,814958145	-0,227585776	0,021713841	protein tyrosine phosphatas	PTPN9
ENSG00000204272	27,5612061	32,12983231	6,419320866	-0,227564828	0,001293366	negative regulator of P-body	NBDY
ENSG00000166925	88,22373042	103,0912516	8,186232435	-0,22756275	2,05E-05	TSC22 domain family memb	TSC22D4
ENSG00000183513	20,31074595	23,82399147	6,115305773	-0,227082331	0,035522448	cytochrome c oxidase assem	COA5
ENSG00000132950	10,65645681	12,47482035	5,110586776	-0,227040153	0,031223729	zinc finger MYM-type contai	ZMYM5
ENSG00000271601	57,77419936	67,46428669	7,504831765	-0,226631119	0,000261779	limb and CNS expressed 1 lik	LIX1L
ENSG00000168701	17,71340631	20,67844287	5,972575866	-0,226164069	0,027165299	transmembrane protein 208	TMEM208
ENSG00000124217	14,40842776	16,87179826	5,58203573	-0,226108113	0,001926519	molybdenum cofactor synth	MOCS3
ENSG00000172273	24,90225105	29,15929841	6,44791782	-0,225945395	0,049143498	histone H4 transcription fact	HINFP
ENSG00000157837	57,67486647	67,48788041	7,580035645	-0,225916747	0,005561171	signal peptide peptidase like	SPPL3
ENSG00000163788	32,16493904	37,69355543	6,567516333	-0,22565902	0,003062517	SNF related kinase	SNRK
ENSG00000159592	77,18915075	90,22382494	8,017737915	-0,225532026	0,000393454	GC-rich promoter binding pr	GPBP1L1
ENSG00000089063	76,19396067	89,25396173	8,232788378	-0,22538484	0,014410389	transmembrane protein 230	TMEM230
ENSG00000177542	35,22412852	41,25507853	6,826947607	-0,225324401	0,043432599	solute carrier family 25 mem	SLC25A22
ENSG00000136628	152,888215	179,1186015	9,005358934	-0,225152342	0,022529794	glutamyl-prolyl-tRNA synthe	EPRS1
ENSG00000140299	75,12798046	88,00106035	7,901223124	-0,224960474	0,000106914	BCL2 interacting protein 2	BNIP2
ENSG00000133872	177,4949142	207,5100617	9,21578146	-0,224954	0,0218944	store-operated calcium entr	SARAF
ENSG00000147679	16,52626392	19,34463643	5,813089743	-0,224825785	0,013547505	UTP23 small subunit proces	UTP23
ENSG00000162819	58,50302546	68,47611129	7,561376155	-0,224687018	0,001416987	BRO1 domain and CAAX mo	BROX
ENSG00000100109	45,5033166	53,30842019	7,330879126	-0,224681055	0,008736346	tuftelin interacting protein 1	TFIP11

ENSG00000151502	57,53394982	67,29714508	7,641494019	-0,22457404	0,026692324	VPS26, retromer complex cc	VPS26B
ENSG00000085185	64,69926019	75,42922513	7,704714804	-0,224572105	0,037252957	BCL6 corepressor like 1	BCORL1
ENSG00000143543	62,8880491	73,51548911	7,686636299	-0,224368173	0,002638792	jumping translocation break	JTB
ENSG00000132740	29,16522325	34,17098357	6,701034032	-0,224269056	0,036411691	immunoglobulin mu DNA bi	IGHMBP2
ENSG00000130158	98,23110646	115,13475	8,184712021	-0,224245088	0,032222534	dedicator of cytokinesis 6	DOCK6
ENSG00000176105	107,9901667	126,3669748	8,440120236	-0,224218838	0,000108867	YES proto-oncogene 1, Src fa	YES1
ENSG00000139131	21,2687532	24,89341009	6,135370725	-0,22413232	0,035332467	tyrosyl-tRNA synthetase 2	YARS2
ENSG00000110395	99,81235694	116,8534933	8,202503996	-0,223907131	0,012947438	Cbl proto-oncogene	CBL
ENSG00000109220	17,72540835	20,59435571	5,829322982	-0,22375162	0,028158824	cysteine rich hydrophobic do	CHIC2
ENSG00000065135	112,2432577	131,3343949	8,500327884	-0,223425301	0,032951307	G protein subunit alpha i3	GNAI3
ENSG00000118454	30,80578402	35,98705316	6,69400741	-0,22323129	0,024745123	ankyrin repeat domain 13C	ANKRD13C
ENSG00000123091	102,5864877	118,9412266	8,465512465	-0,223175975	0,00347448	ring finger protein 11	RNF11
ENSG00000185236	168,1597309	195,9770163	9,045027169	-0,2230988	0,000219115	RAB11B, member RAS oncog	RAB11B
ENSG00000196422	54,28482524	63,43543952	7,438844969	-0,222960359	0,003975968	protein phosphatase 1 regul	PPP1R26
ENSG00000179918	59,18529057	69,23454991	7,727255402	-0,222487725	0,006478967	selenophosphate synthetase	SEPHS2
ENSG00000101473	20,7894816	24,3089616	6,244084075	-0,222157028	0,020049348	acyl-CoA thioesterase 8	ACOT8
ENSG00000112996	46,75857838	54,69620237	7,234727203	-0,222019423	0,017263357	mitochondrial ribosomal pro	MRPS30
ENSG00000115306	711,3285854	822,9190131	11,06350519	-0,221972094	0,000954096	spectrin beta, non-erythrocy	SPTBN1
ENSG00000214063	121,1087752	141,645226	8,508086218	-0,221489669	0,001329436	tetraspanin 4	TSPAN4
ENSG00000114982	78,78313458	92,04359167	8,058888778	-0,221234113	0,000491675	KAT8 regulatory NSL comple	KANSL3
ENSG00000145191	41,59550621	48,58898274	7,142420112	-0,221192667	0,005877599	eukaryotic translation initiat	EIF2B5
ENSG00000060069	45,95285314	53,70323251	7,392816872	-0,221157096	0,032344203	CTD phosphatase subunit 1	CTDP1
ENSG00000139233	17,02940611	19,88107511	5,812489077	-0,221077364	0,012823037	LLP homolog, long-term syn	LLPH
ENSG00000011405	110,5066085	128,6469363	8,46255818	-0,220729291	0,031157959	phosphatidylinositol-4-phos	PIK3C2A
ENSG00000204628	525,2190469	612,7101888	10,83899268	-0,220692395	0,018238722	receptor for activated C kina	RACK1
ENSG00000156709	37,22492195	43,49173988	6,964793861	-0,220682156	0,035627268	apoptosis inducing factor mi	AIFM1
ENSG00000183475	22,42772311	26,1837949	6,244918878	-0,220519974	0,004376181	ankyrin repeat and SOCS bo	ASB7
ENSG00000159079	11,37560371	13,28991304	5,17750609	-0,220421358	0,014690633	cilia and flagella associated	CFAP298
ENSG00000170677	33,96891825	39,63719981	6,720947451	-0,220292189	0,001934881	suppressor of cytokine signa	SOCS6
ENSG00000101266	134,7937421	157,1749264	8,799940769	-0,219918904	0,000626978	casein kinase 2 alpha 1	CSNK2A1
ENSG00000154945	58,18707506	67,88269684	7,489675562	-0,219776567	0,001209105	ankyrin repeat domain 40	ANKRD40
ENSG00000215712	21,21070815	24,75031056	6,094257038	-0,219743159	0,008230663	transmembrane protein 242	TMEM242
ENSG00000099800	44,24731215	51,57471529	7,301043455	-0,219671581	0,029587186	translocase of inner mitocho	TIMM13
ENSG00000188938	40,57475837	47,34651189	7,01257877	-0,219631937	0,032348721	family with sequence simila	FAM120AOS
ENSG00000204438	30,12591787	35,08122582	6,64109805	-0,219449909	0,022061748	G-patch domain and ankyrin	GPANK1
ENSG00000166908	51,00210605	59,53177737	7,44688594	-0,219347794	0,005302089	phosphatidylinositol-5-phos	PIP4K2C
ENSG00000126581	60,71902518	70,59679913	7,696604074	-0,219317467	0,005713857	beclin 1	BECN1
ENSG00000125977	117,1495717	136,7011608	8,542665567	-0,219111053	0,000440533	eukaryotic translation initiat	EIF2S2
ENSG00000134014	35,32664637	41,23022232	6,977235088	-0,218878783	0,010682982	elongator acetyltransferase	ELP3
ENSG00000160058	82,92048495	96,37002231	8,178319091	-0,218845091	0,001147713	BSD domain containing 1	BSDC1
ENSG00000137824	47,14244273	54,94362341	7,181492374	-0,218783247	0,01546699	regulator of microtubule dy	RMDN3
ENSG00000154059	30,64523342	35,58827195	6,688764038	-0,2184797	0,018012829	impact RWD domain protein	IMPACT
ENSG00000143811	47,79284616	55,72276171	7,229520273	-0,218448376	0,002138914	pyrroline-5-carboxylate redu	PYCR2
ENSG00000162852	28,77587219	33,54376481	6,506055179	-0,218288339	0,022467625	consortin, connexin sorting	CNST
ENSG00000139637	28,88477119	33,66768555	6,681818563	-0,218213573	0,002132979	MYG1 exonuclease	MYG1
ENSG00000197965	162,7250002	189,7972038	8,942951823	-0,218212994	0,01288489	myelin protein zero like 1	MPZL1
ENSG00000144744	53,18417602	61,88833652	7,363352862	-0,218197669	0,005788091	ubiquitin like modifier activa	UBA3
ENSG00000181830	43,09612068	50,011587	7,276779781	-0,218089863	0,016461437	solute carrier family 35 mem	SLC35C1
ENSG00000104177	71,61314522	83,57055205	7,860068954	-0,218008425	0,015087786	myelin expression factor 2	MYEF2
ENSG00000117410	77,09603224	89,92692313	7,894440319	-0,217711345	0,006259451	ATPase H+ transporting V0 s	ATP6V0B
ENSG00000183751	49,77404456	58,0080812	7,463538288	-0,217445574	0,028923794	transducin beta like 3	TBL3
ENSG00000166128	63,80322806	74,16873707	7,694130971	-0,217431933	0,013378634	RAB8B, member RAS oncoge	RAB8B
ENSG00000110514	61,97231533	72,13576197	7,661070965	-0,217422696	0,005708803	MAP kinase activating death	MADD
ENSG00000126243	32,50886314	37,6593587	6,882749905	-0,217197308	0,005021638	leucine rich repeat and fibro	LRFN3
ENSG00000164144	40,10440624	46,72592873	7,007185359	-0,217046994	0,043528118	ADP ribosylation factor inter	ARFIP1
ENSG00000165487	55,96572067	65,23297135	7,495679604	-0,216988765	0,004485838	mitochondrial calcium uptak	MICU2
ENSG00000124177	83,64475641	97,28994775	8,154476883	-0,216699248	0,017074154	chromodomain helicase DNA	CHD6
ENSG00000166313	88,94583418	103,4733075	8,265135867	-0,216375801	0,002903769	amyloid beta precursor prot	APBB1
ENSG00000112306	153,5363613	178,3381514	9,056595732	-0,216367402	0,019765017	ribosomal protein S12	RPS12
ENSG00000159267	48,07295687	55,89871033	7,219358462	-0,216307541	0,029325123	holocarboxylase synthetase	HLCS
ENSG00000140992	74,25015959	86,45502802	7,885748356	-0,216173959	4,51E-07	3-phosphoinositide depende	PDPK1
ENSG00000254986	57,62749206	67,08210024	7,505575397	-0,216056421	0,022321616	dipeptidyl peptidase 3	DPP3
ENSG00000264522	54,95790569	63,8419157	7,492326454	-0,215924997	0,008409545	OTU deubiquitinase 7B	OTUD7B
ENSG00000148843	82,45510186	95,99044203	8,156742582	-0,215744303	0,00594291	programmed cell death 11	PDCD11
ENSG00000213463	51,67682695	60,13733977	7,42738589	-0,215521919	0,020709077	synaptojanin 2 binding prote	SYNJ2BP
ENSG00000166135	90,27705101	105,0584954	8,314077207	-0,21549119	6,24E-06	hypoxia inducible factor 1 su	HIF1AN
ENSG00000100425	58,77272754	68,24065584	7,571874983	-0,215289181	0,017631019	bromodomain containing 1	BRD1
ENSG00000083844	33,49929053	38,9637576	6,838399345	-0,215246689	0,031508638	zinc finger protein 264	ZNF264
ENSG00000148634	69,15121202	80,48315777	7,861202737	-0,215088873	0,005863565	HECT and RLD domain conta	HERC4
ENSG00000135930	77,94442674	90,53049674	8,028692205	-0,215052216	0,00164269	eukaryotic translation initiat	EIF4E2
ENSG00000125676	76,19970852	88,66762274	7,85698357	-0,215033979	0,008131715	THO complex 2	THOC2
ENSG00000168259	75,76876763	88,18498788	7,96523644	-0,214978583	0,000279761	DnaJ heat shock protein fam	DNAJC7
ENSG00000166848	58,97440749	68,39900124	7,702009959	-0,214618883	0,007293036	TERF2 interacting protein	TERF2IP
ENSG00000078140	86,38460192	100,523358	8,027243276	-0,21448246	0,023847367	ubiquitin conjugating enzym	UBE2K
ENSG00000159111	51,88266016	60,38269965	7,491708228	-0,214431876	0,033317992	mitochondrial ribosomal pro	MRPL10
ENSG00000118482	137,6580036	160,0258725	8,85607679	-0,214291604	0,043678397	PHD finger protein 3	PHF3
ENSG00000198663	136,0887434	158,2019466	8,892824595	-0,214202279	0,000598772	chromosome 6 open reading	C6orf89
ENSG00000093144	36,08366064	41,902595	6,891869807	-0,214006433	0,037945866	ethylmalonyl-CoA decarboxy	ECHDC1
ENSG00000182307	35,20241347	40,91964272	7,01182531	-0,213843837	0,044598113	chromosome 8 open reading	C8orf33
ENSG00000160908	15,72516979	18,27350857	5,780837563	-0,213839615	0,022730887	zinc finger protein 394	ZNF394
ENSG00000105204	49,6350913	57,36240577	7,490687983	-0,213424648	0,023299458	dual specificity tyrosine pho	DYRK1B
ENSG00000130731	52,99075739	61,39613533	7,380113964	-0,213337572	0,007728096	methyltransferase like 26	METTL26
ENSG00000156650	39,42858914	45,68939016	7,104988071	-0,213304313	0,001211386	lysine acetyltransferase 6B	KAT6B
ENSG00000119682	94,61079124	109,8870433	8,28748515	-0,213238022	0,00111894	apoptosis resistant E3 ubiqu	AREL1
ENSG00000143252	90,61236651	105,3189102	8,256302071	-0,212898341	0,001836571	succinate dehydrogenase co	SDHC
ENSG00000196365	192,7529279	223,4413159	9,260369326	-0,212888969	0,000724081	lon peptidase 1, mitochondr	LONP1
ENSG00000102471	57,42310899	66,50288109	7,543997498	-0,212702146	0,040821375	Nedd4 family interacting pro	NDFIP2
ENSG00000107758	38,41348023	44,57165573	7,051840715	-0,2125892	0,000696961	protein phosphatase 3 catal	PPP3CB
ENSG00000162227	16,13693949	18,72998803	5,75348059	-0,212260777	0,014002483	TATA-box binding protein as	TAF6L
ENSG00000172046	95,75427536	111,1133084	8,401817175	-0,212222263	0,005009665	ubiquitin specific peptidase	USP19
ENSG00000183597	27,03320005	31,34398513	6,34264332	-0,212161431	0,042842741	transport and golgi organiza	TANGO2
ENSG00000087995	24,5878625	28,50871265	6,350640772	-0,212069962	0,028148044	methyltransferase 2A, meth	METTL2A
ENSG00000081177	33,06247892	38,25115701	6,86969492	-0,21195078	0,009125263	exonuclease 3'-5' domain co	EXD2
ENSG00000006194	32,78352348	38,05770911	6,787365395	-0,211859433	0,006063727	zinc finger protein 263	ZNF263
ENSG00000169032	107,1899275	124,1389865	8,256030781	-0,211816743	0,00695946	mitogen-activated protein k	MAP2K1
ENSG00000084754	159,8851951	185,6656887	9,120951745	-0,211676632	0,001807176	hydroxyacyl-CoA dehydroge	HADHA
ENSG00000177951	67,51536148	77,96173473	7,898938057	-0,211628719	0,043632528	Bet1 golgi vesicular membra	BET1L
ENSG00000186591	291,566493	334,3850863	9,881072036	-0,211540826	0,015793254	ubiquitin conjugating enzym	UBE2H
ENSG00000102738	14,5227265	16,85744718	5,59505884	-0,211491137	0,043632528	mitochondrial ribosomal pro	MRPS31
ENSG00000273749	197,8544971	229,0691626	9,255605356	-0,211395359	0,001170333	cytoplasmic FMR1 interactin	CYFIP1
ENSG00000117118	49,87878011	57,82258156	7,426430395	-0,211364958	0,026382396	succinate dehydrogenase co	SDHB
ENSG00000119285	72,40665572	84,00230184	7,891364328	-0,211164096	0,015236431	HEAT repeat containing 1	HEATR1
ENSG00000136888	78,49680595	90,96943977	8,059439914	-0,210637483	0,030037171	ATPase H+ transporting V1 s	ATP6V1G1
ENSG00000143379	39,85305292	46,16725815	7,128151588	-0,210615076	0,030124229	SET domain bifurcated histo	SETDB1
ENSG00000181038	17,85478378	20,6801957	5,88626431	-0,210590419	0,043031513	methyltransferase like 23	METTL23
ENSG00000255302	173,6246877	199,4266721	9,146963243	-0,210345293	6,96E-05	EP300 interacting inhibitor c	EID1
ENSG00000188242	11,18912022	12,79964456	5,064158395	-0,210231915	0,036516433	uncharacterized LOC25845	PP7080
ENSG00000148730	211,0386153	244,674219	9,370046789	-0,210205237	0,001979857	eukaryotic translation initiat	EIF4EBP2
ENSG00000140553	139,6801173	161,9527897	8,911723384	-0,210148971	5,22E-08	unc-45 myosin chaperone A	UNC45A
ENSG00000162971	11,92254428	13,80849471	5,299556077	-0,2098303	0,032449993	tRNA-yW synthesizing prote	TYW5
ENSG00000224470	80,49935286	93,31846812	8,089833723	-0,209826785	0,009480878	ataxin 1 like	ATXN1L
ENSG00000048649	102,0949205	118,1891685	8,349544512	-0,209810379	0,002905246	remodeling and spacing fact	RSF1
ENSG00000089818	21,61246837	25,04298499	6,194468064	-0,209680926	0,017325599	NECAP endocytosis associat	NECAP1
ENSG00000198815	55,40932228	64,19643351	7,507315838	-0,20964643	0,000146053	forkhead box J3	FOXJ3
ENSG00000076043	63,94880588	74,11788007	7,749764101	-0,209613934	0,018604828	RNA exonuclease 2	REXO2

ENSG00000127054	100,0735857	115,9668504	8,387186083	-0,209145474	0,004054055	integrator complex subunit	INTS11
ENSG00000070413	169,1964156	195,0256106	9,100597597	-0,209087622	0,03002067	DiGeorge syndrome critical	DGCR2
ENSG00000115540	16,68596375	19,31923509	5,61995203	-0,209076123	0,043144415	MOB family member 4, pho	MOB4
ENSG00000072803	79,69703421	92,22879564	7,974213548	-0,208672545	0,000375131	F-box and WD repeat domai	FBXW11
ENSG00000144579	128,440661	148,3038712	8,750862562	-0,208510301	0,012891506	CTD small phosphatase 1	CTDSP1
ENSG00000163312	13,38339474	15,45601017	5,553258973	-0,208398818	0,015141548	helicase, POLQ like	HELQ
ENSG00000180035	29,41113996	34,00055815	6,549952066	-0,20826606	0,025444677	zinc finger protein 48	ZNF48
ENSG00000105701	305,4978724	352,8374905	10,02824427	-0,208219844	0,000359322	FKBP prolyl isomerase 8	FKBP8
ENSG00000124160	50,99570384	59,07244871	7,303420566	-0,20813946	0,000635203	nuclear receptor coactivator	NCOA5
ENSG00000125971	95,33286664	110,3779284	8,290610687	-0,207925357	0,026292754	dynein light chain roadblock	DYNLRB1
ENSG00000047365	25,41735665	29,42362846	6,165148429	-0,207653684	0,029524276	ArfGAP with RhoGAP domai	ARAP2
ENSG00000105849	34,96217644	40,47252357	6,775875647	-0,207650838	0,013463237	RNA polymerase I subunit F	POLR1F
ENSG00000149182	70,04884651	81,02549391	7,93378585	-0,207326824	0,000262206	ADP ribosylation factor GTP	ARFGAP2
ENSG00000129187	80,82453815	93,13575921	8,034740544	-0,207312563	0,010266814	dCMP deaminase	DCTD
ENSG00000161036	41,12702087	47,57199899	6,952394754	-0,207296378	0,000543054	leucine rich repeats and WD	LRWD1
ENSG00000176407	52,07566527	60,25554027	7,358427473	-0,207247415	0,004738421	potassium channel modulat	KCMF1
ENSG00000204463	293,501501	339,1465406	10,00598547	-0,206923671	0,001134803	BAG cochaperone 6	BAG6
ENSG00000124214	176,8573415	204,0783375	9,092867731	-0,206828886	0,012379594	staufen double-stranded RN	STAU1
ENSG00000198315	47,21940642	54,49952261	7,354104137	-0,206726692	0,018677402	zinc finger with KRAB and SC	ZKSCAN8
ENSG00000157538	58,38090512	67,50327384	7,570546825	-0,20635296	7,04E-06	VPS26 endosomal protein sc	VPS26C
ENSG00000177105	40,93351083	47,33157764	7,023704267	-0,206138716	0,04505123	ras homolog family member	RHOG
ENSG00000004766	28,94420233	33,463746	6,562907645	-0,205978725	0,001907827	VPS50 subunit of EARP/GAR	VPS50
ENSG00000187954	72,03399533	83,11770448	8,014519216	-0,205758808	0,027766346	cysteine and histidine rich 1	CYHR1
ENSG00000140943	220,9326491	255,23356	9,619286941	-0,205577236	0,00076167	membrane bound transcript	MBTPS1
ENSG00000101608	224,5478694	258,6685675	9,441058676	-0,205564197	0,00512802	myosin light chain 12A	MYL12A
ENSG00000109118	98,89975426	114,2981598	8,340830703	-0,205171484	0,007584976	PHD finger protein 12	PHF12
ENSG00000121775	15,72484564	18,15451598	5,579455143	-0,204828018	0,020765479	transmembrane protein 39E	TMEM39B
ENSG00000185129	66,23333914	76,30356504	7,800320828	-0,204780225	0,021075564	purine rich element binding	PURA
ENSG00000171634	123,7984777	142,6317079	8,670271385	-0,204760827	0,045606906	bromodomain PHD finger tr	BPTF
ENSG00000005483	116,2403641	133,4822938	8,604322605	-0,204710104	0,026998743	lysine methyltransferase 2E	KMT2E
ENSG00000144659	33,42271961	38,59902752	6,865535142	-0,204337829	0,010697328	solute carrier family 25 men	SLC25A38
ENSG00000111802	36,93849242	42,65431131	6,856514846	-0,204318375	2,79E-05	tyrosyl-DNA phosphodiester	TDP2
ENSG00000166169	38,79935146	44,90582623	7,126723438	-0,204214039	0,03551491	DNA polymerase lambda	POLL
ENSG00000124228	64,55426907	74,49991693	7,820100173	-0,204210322	0,019636812	DEAD-box helicase 27	DDX27
ENSG00000123728	27,75479796	31,9470683	6,578486476	-0,204031317	0,037636348	RAP2C, member of RAS onc	RAP2C
ENSG00000142528	19,17584067	22,14773455	5,951622947	-0,203540821	0,041560864	zinc finger protein 473	ZNF473
ENSG00000145041	46,85587248	54,05041486	7,155306221	-0,203429424	0,006045139	DDB1 and CUL4 associated f	DCAF1
ENSG00000165669	16,97844698	19,57953633	5,793312865	-0,203253623	0,005945569	family with sequence similar	FAM204A
ENSG00000215193	105,3150798	120,737235	8,407258029	-0,203151692	0,017938848	peroxisomal biogenesis fact	PEX26
ENSG00000146457	97,5884955	112,5967966	8,23495502	-0,20301362	0,037941542	WT1 associated protein	WTAP
ENSG00000165629	75,28581775	86,85241323	7,97218363	-0,202782149	0,008678882	ATP synthase F1 subunit gar	ATP5F1C
ENSG00000177352	36,63953166	42,19193136	6,920042577	-0,202522742	0,028537157	coiled-coil domain containin	CCDC71
ENSG00000188976	113,9657885	131,4046467	8,568568168	-0,20242695	0,001655371	NOC2 like nucleolar associat	NOC2L
ENSG00000128908	51,46189714	59,3458774	7,448289487	-0,202230519	0,018413588	INO80 complex ATPase subu	INO80
ENSG00000152904	30,10686079	34,70841496	6,655828068	-0,202214344	0,016332963	geranylgeranyl diphosphate	GGPS1
ENSG00000113812	32,81005089	37,85912117	6,829062215	-0,202206457	0,022174273	actin related protein 8	ACTR8
ENSG00000158019	26,23149282	30,25816063	6,585013827	-0,202185152	0,049803646	BRISC and BRCA1 A complex	BABAM2
ENSG00000119523	31,20877098	35,942383	6,770777942	-0,201792665	0,001353639	ALG2 alpha-1,3/1,6-mannos	ALG2
ENSG00000104388	107,6796901	123,7906514	8,523074704	-0,201786179	0,041428572	RAB2A, member RAS oncoge	RAB2A
ENSG00000018189	29,52545214	33,91269175	6,359594857	-0,201690571	0,02150251	RUN and FYVE domain conta	RUFY3
ENSG00000143013	54,04482386	62,3527444	7,393320423	-0,201553486	0,03038062	LIM domain only 4	LMO4
ENSG00000128928	82,61812274	95,26055665	8,130160949	-0,201437581	0,025834965	isovaleryl-CoA dehydrogena	IVD
ENSG00000119650	16,62333504	19,16209099	5,924862207	-0,201395993	0,004398977	intraflagellar transport 43	IFT43
ENSG00000109790	65,94598849	75,25425029	7,614142183	-0,201202536	0,005731296	kelch like family member 5	KLHL5
ENSG00000127125	38,93988431	44,78402987	7,042298592	-0,201161622	0,021272615	phosphopantothenoylcystei	PPCS
ENSG00000140632	169,6331643	195,4459157	9,086398046	-0,200819501	2,75E-05	glyoxylate reductase 1 hom	GLYR1
ENSG00000143771	43,85220546	50,47820766	7,04359472	-0,200656586	0,021354763	cornichon family AMPA rece	CNIH4
ENSG00000137575	150,5934788	173,3744473	8,921882832	-0,200563757	0,000929678	syndecan binding protein	SDCBP
ENSG00000168040	48,38854144	55,62450708	7,385530782	-0,200469475	0,032690993	Fas associated via death dor	FADD
ENSG00000204673	97,95767123	112,4865779	8,375413052	-0,20038742	0,00588664	AKT1 substrate 1	AKT1S1
ENSG00000078304	87,49100417	100,6357064	8,186537436	-0,200235231	0,012648365	protein phosphatase 2 regul	PPP2R5C
ENSG00000180104	59,08563784	68,06452335	7,73066053	-0,200148352	0,004884762	exocyst complex componen	EXOC3
ENSG00000188706	76,16628613	87,32300052	8,017336797	-0,200025003	0,034166489	zinc finger DHHC-type palmi	ZDHHC9
ENSG00000163162	39,72523108	45,73199662	7,066188864	-0,199197772	0,046122744	ring finger protein 149	RNF149
ENSG00000056097	140,9186126	162,0551625	8,861400907	-0,199174471	0,000426089	zinc finger RNA binding prot	ZFR
ENSG00000155463	84,11269267	96,76729492	8,175744099	-0,198911264	0,007142063	OXA1L mitochondrial inner	OXA1L
ENSG00000095059	44,23769201	50,88863578	7,229754426	-0,19890684	0,009259175	deoxyhypusine synthase	DHPS
ENSG00000165525	56,06326507	64,58429384	7,512062885	-0,198764662	0,047189697	nuclear export mediator fac	NEMF
ENSG00000187713	38,24473285	43,94848779	7,044270877	-0,198723557	0,002900362	transmembrane protein 203	TMEM203
ENSG00000125875	80,07034657	92,06206256	8,059437115	-0,198615985	0,000755681	TBC1 domain family membe	TBC1D20
ENSG00000103249	170,0247644	195,4505967	9,298590671	-0,198615012	0,040109596	chloride voltage-gated chan	CLCN7
ENSG00000126945	86,15209767	98,97957378	8,18493915	-0,198568046	0,001656763	heterogeneous nuclear ribo	HNRNPH2
ENSG00000175029	148,6138988	170,5600857	8,906025434	-0,198152304	0,037211155	C-terminal binding protein 2	CTBP2
ENSG00000124209	57,20770183	65,63448212	7,55033361	-0,198011499	0,007840228	RAB22A, member RAS oncog	RAB22A
ENSG00000160746	47,13052386	54,26426301	7,305860343	-0,197995732	0,045145043	anoctamin 10	ANO10
ENSG00000109111	187,8832861	215,6698976	9,275201857	-0,197953314	0,004376181	SPT6 homolog, histone chap	SUPT6H
ENSG00000076685	95,25549386	109,553722	8,182967857	-0,197902391	0,002837312	5'-nucleotidase, cytosolic II	NT5C2
ENSG00000055917	132,0561343	151,7337821	8,672878073	-0,19778821	0,001519807	pumilio RNA binding family	PUM2
ENSG00000178605	48,77195988	56,07187617	7,49712264	-0,197787266	0,040290075	GTP binding protein 6 (putat	GTPBP6
ENSG00000088035	14,8058156	17,02138176	5,476423861	-0,197778963	0,045159107	ALG6 alpha-1,3-glucosyltran	ALG6
ENSG00000228300	26,84545715	30,84515024	6,590167295	-0,197608255	0,028924125	family with sequence similar	FAM174C
ENSG00000143457	35,34556501	40,62231599	6,931215222	-0,197349682	0,002275384	golgi phosphoprotein 3 like	GOLPH3L
ENSG00000198917	62,87111963	72,14253474	7,725819589	-0,197141694	0,037825739	SPOUT domain containing m	SPOUT1
ENSG00000065883	94,93057566	109,0042846	8,267057439	-0,197135917	0,000115394	cyclin dependent kinase 13	CDK13
ENSG00000196547	151,6802354	174,2498033	8,837241805	-0,197004018	0,032669882	mannosidase alpha class 2A	MAN2A2
ENSG00000137413	40,52935385	46,49079425	7,090299133	-0,196534869	0,015349286	TATA-box binding protein as	TAF8
ENSG00000117133	31,80213418	36,49167504	6,694819781	-0,196365741	0,027855591	ribosome production factor	RPF1
ENSG00000140995	74,19710187	85,34088878	7,978610739	-0,196333272	0,029986351	differentially expressed in FI	DEF8
ENSG00000164961	69,65671281	80,01566452	7,839928283	-0,196302301	0,012145955	WASH complex subunit 5	WASHC5
ENSG00000168036	510,0541533	584,1618208	10,67547217	-0,19615837	0,000449565	catenin beta 1	CTNNB1
ENSG00000159202	184,2449419	211,4135847	9,178884321	-0,195990581	5,88E-06	ubiquitin conjugating enzym	UBE2Z
ENSG00000168818	26,46376822	30,37668769	6,461657536	-0,195797184	0,000396021	syntaxin 18	STX18
ENSG00000174485	33,98405286	38,96608576	6,807586527	-0,195654411	0,022201866	DENN domain containing 4A	DENND4A
ENSG00000162298	76,42024201	87,60724826	8,040861009	-0,195548324	0,005978314	synoviolin 1	SYVN1
ENSG00000115295	39,12322409	44,88181492	7,037762562	-0,195460955	0,043505878	CAP-Gly domain containing	CLIP4
ENSG00000128989	163,3687506	187,3925685	8,908732249	-0,195240062	0,014405823	cAMP regulated phosphopro	ARPP19
ENSG00000021574	20,70890137	23,64980687	6,143199249	-0,19510903	0,014854	spastin	SPAST
ENSG00000140157	65,75962194	75,45784193	7,636316254	-0,195102364	0,018459254	NIPA magnesium transporte	NIPA2
ENSG00000036054	42,63002977	48,82277918	7,191297504	-0,195018005	0,007157016	TBC1 domain family membe	TBC1D23
ENSG00000139651	54,3204091	62,3648266	7,535327691	-0,194918111	0,002486862	zinc finger protein 740	ZNF740
ENSG00000187778	44,24765489	50,74191143	7,245979764	-0,194562428	0,00177835	microspherule protein 1	MCRS1
ENSG00000156671	52,26373695	59,88323847	7,349618786	-0,19454624	0,006171368	sterile alpha motif domain c	SAMD8
ENSG00000130724	75,08245264	85,82322377	8,043918809	-0,194386541	0,017053177	charged multivesicular body	CHMP2A
ENSG00000157077	44,07284339	50,4464836	7,253937849	-0,194367157	0,007704004	zinc finger FYVE-type contai	ZFYVE9
ENSG00000176386	9,772156161	11,20928163	5,092866275	-0,194019147	0,034821189	cell division cycle 26	CDC26
ENSG00000178913	87,05630217	99,58891037	8,265908758	-0,194010706	0,011740979	TATA-box binding protein as	TAF7
ENSG00000103510	29,69812133	34,03182835	6,619427836	-0,193979701	0,015449339	lysine acetyltransferase 8	KAT8
ENSG00000168610	380,718756	436,6846921	10,26008117	-0,193949261	0,021751051	signal transducer and activa	STAT3
ENSG00000123200	122,5952796	140,1888105	8,617570945	-0,193610307	0,002685586	zinc finger CCCH-type contai	ZC3H13
ENSG00000141741	41,52320529	47,59626897	7,122860349	-0,193263215	0,012069461	migration and invasion enha	MIEN1
ENSG00000127314	96,02239944	109,9743425	8,177954811	-0,192968929	0,007586876	RAP1B, member of RAS onc	RAP1B
ENSG00000065548	67,1366685	76,91135828	7,80790143	-0,192729696	0,015634294	zinc finger CCCH-type contai	ZC3H15
ENSG00000141446	27,49583408	31,39433576	6,487983057	-0,192338308	0,025616707	establishment of sister chro	ESCO1
ENSG00000077147	227,8950584	260,1486891	9,533872987	-0,192328996	0,008736967	transmembrane 9 superfam	TM9SF3
ENSG00000162378	60,36482658	69,10555168	7,684688352	-0,192195739	0,019914684	zyg-11 family member B, cel	ZYG11B

ENSG00000178764	43,97774866	49,98137213	7,454250425	-0,191933341	0,037296238	zinc fingers and homeoboxe	ZHX2
ENSG00000100554	68,26649308	77,89079327	7,697178116	-0,191224398	0,017626368	ATPase H+ transporting V1 s	ATP6V1D
ENSG00000029725	108,5185155	124,1700007	8,434613312	-0,191174178	0,000477645	rabaptin, RAB GTPase bindir	RABEP1
ENSG00000115568	59,78979514	68,41249105	7,575207015	-0,190918864	0,009637435	zinc finger protein 142	ZNF142
ENSG00000163517	36,77397036	42,03194182	6,937885229	-0,190911202	0,008221286	histone deacetylase 11	HDAC11
ENSG00000165102	119,9424362	137,1608044	8,554397737	-0,190726178	0,032789814	heparan-alpha-glucosaminic	HGSNAT
ENSG00000169692	68,80342257	78,41622039	7,678770555	-0,190409954	0,024939159	1-acylglycerol-3-phosphate	AGPAT2
ENSG00000081019	23,85996852	27,26454116	6,315870604	-0,189673985	0,028156355	round spermatid basic prote	RSBN1
ENSG00000076554	31,89955954	36,44308913	6,539876097	-0,189452199	0,045047954	tumor protein D52	TPD52
ENSG00000068400	79,98160366	91,48627079	8,125085459	-0,189360364	0,036423096	GRIP1 associated protein 1	GRIPAP1
ENSG00000033011	28,41505105	32,46449425	6,680847369	-0,189287567	0,029785859	ALG1 chitobiosyldiphosphoc	ALG1
ENSG00000171703	44,86131308	51,178371	7,360752389	-0,189259378	0,007442995	transcription elongation fact	TCEA2
ENSG00000104142	88,64171331	101,1582859	8,246881331	-0,189203095	0,012073524	VPS18 core subunit of CORV	VPS18
ENSG00000177034	23,34419398	26,68132091	6,289156676	-0,189056807	0,029810167	metaxin 3	MTX3
ENSG00000164941	46,14541704	52,71328196	7,222906629	-0,188529955	0,013304045	integrator complex subunit	INTS8
ENSG00000100865	24,34552871	27,80822094	6,375256114	-0,188399055	0,034440153	cyclin dependent kinase 2 in	CINP
ENSG00000139842	85,65327889	97,8289649	8,143781938	-0,18822388	0,001025248	cullin 4A	CUL4A
ENSG00000160218	43,63450554	49,79357598	7,071640443	-0,187331244	0,00069465	trafficking protein particle c	TRAPPC10
ENSG00000133142	75,30303953	85,69442961	8,059846056	-0,187053118	0,01611148	transcription elongation fact	TCEAL4
ENSG00000174748	377,3136325	429,9011234	10,33122762	-0,186662995	0,042073419	ribosomal protein L15	RPL15
ENSG00000198546	13,56645239	15,46421427	5,431061441	-0,18655331	0,029400231	zinc finger protein 511	ZNF511
ENSG00000141252	77,75284917	88,5998406	7,993203251	-0,185462427	0,000634874	VPS53 subunit of GARP com	VPS53
ENSG00000218739	38,41330676	43,59794199	6,918945267	-0,185195847	0,01335244	CEBPZ opposite strand	CEBPZOS
ENSG00000155827	72,67960651	82,82846148	7,941140195	-0,185067737	2,65E-05	ring finger protein 20	RNF20
ENSG00000078061	80,5935837	91,76118024	8,095448456	-0,184681333	0,000608365	A-Raf proto-oncogene, serin	ARAF
ENSG00000140521	141,213528	160,8228797	8,864499688	-0,184173501	0,012353714	DNA polymerase gamma, ca	POLG
ENSG00000186866	55,87885737	63,5996241	7,764466571	-0,18407384	0,012699891	protein O-fucosyltransferase	POFUT2
ENSG00000064545	34,04923861	38,78998899	6,892528095	-0,184025023	0,003274783	transmembrane protein 161	TMEM161A
ENSG00000155876	106,3068341	120,7548683	8,516562705	-0,183958054	0,022509054	Ras related GTP binding A	RRAGA
ENSG00000104853	172,0598823	195,8334912	9,235732451	-0,183755418	0,000176146	CLPTM1 regulator of GABA t	CLPTM1
ENSG00000198677	116,9903673	133,0568371	8,599622625	-0,183356786	6,41E-05	tetratricopeptide repeat dor	TTC37
ENSG00000101940	71,50726272	81,35295282	7,934307767	-0,182522447	0,015146992	WD repeat domain 13	WDR13
ENSG00000171497	52,84347332	60,0940497	7,360167451	-0,182243863	0,001829881	peptidylprolyl isomerase D	PPID
ENSG00000099810	52,52522467	59,74325769	7,262998611	-0,181843163	0,011851441	methylthioadenosine phosp	MTAP
ENSG00000088808	42,58571953	48,23080105	6,981589554	-0,181302797	0,032894265	protein phosphatase 1 regul	PPP1R13B
ENSG00000142039	41,37743635	46,98994716	7,102555016	-0,181251074	0,013205852	coiled-coil domain containin	CCDC97
ENSG00000143756	39,09647115	44,42087011	7,015052905	-0,181079717	0,023608166	F-box protein 28	FBXO28
ENSG00000162032	56,97451377	64,79557299	7,52300141	-0,180957813	0,01724109	splA/ryanodine receptor dor	SPSB3
ENSG00000187866	21,34969852	24,24873674	6,215403366	-0,180572955	0,010344965	PP2A Aalpha (PPP2R1A) and	PABIR1
ENSG00000024048	65,30516128	74,17864555	7,754246361	-0,180315956	0,033158959	ubiquitin protein ligase E3 c	UBR2
ENSG00000112290	27,0790715	30,72528998	6,3163211	-0,180191302	0,02528712	WASP family member 1	WASF1
ENSG00000109606	161,9748119	184,0073441	8,918870717	-0,180102093	0,04734779	DEAH-box helicase 15	DHX15
ENSG00000257267	32,19374719	36,41877557	6,78542492	-0,179680886	0,043096205	zinc finger protein 271, pseu	ZNF271P
ENSG00000185085	43,6936991	49,61658799	7,104834288	-0,179577089	0,035618076	integrator complex subunit	INTS5
ENSG00000105819	51,82645696	58,80838079	7,388733422	-0,179380378	0,002300424	peptidase, mitochondrial pr	PMPCB
ENSG00000155729	19,13605053	21,70845085	6,027039214	-0,179369596	0,027331914	potassium channel tetramer	KCTD18
ENSG00000022840	201,1802023	228,174606	9,404786555	-0,179330776	0,000359322	ring finger protein 10	RNF10
ENSG00000165282	42,38858108	48,09872874	7,216757148	-0,1792881	0,023282777	phosphatidylinositol glycan	PIGO
ENSG00000090612	24,34850255	27,61644807	6,470851022	-0,17926593	0,022666264	zinc finger protein 268	ZNF268
ENSG00000183741	330,3033566	374,63064	10,02368517	-0,179220783	0,017544376	chromobox 6	CBX6
ENSG00000171940	112,9580081	128,1453245	8,319612729	-0,179185175	0,013011117	zinc finger protein 217	ZNF217
ENSG00000127980	33,79297059	38,33146771	6,733735362	-0,178930985	0,004838736	peroxisomal biogenesis fact	PEX1
ENSG00000148719	51,96790378	58,89807416	7,621575331	-0,178785558	0,018252623	DnaJ heat shock protein fan	DNAJB12
ENSG00000086666	77,65105333	88,10236859	7,934746071	-0,178637562	0,026276367	zinc finger AN1-type contair	ZFAND6
ENSG00000100084	15,82385038	17,94491653	5,625627963	-0,178436875	0,03073547	histone cell cycle regulator	HIRA
ENSG00000135341	83,88461209	95,13571605	8,139006801	-0,178414805	0,005360016	mitogen-activated protein k	MAP3K7
ENSG00000137409	240,1297077	271,6026021	9,51468091	-0,178271887	0,024676066	mitochondrial carrier 1	MTCH1
ENSG00000100815	80,47523325	91,21226812	8,155211946	-0,178176905	0,030953539	thyroid hormone receptor ir	TRIP11
ENSG00000198792	187,6358071	212,6401758	9,224385268	-0,178176653	0,044380369	transmembrane protein 184	TMEM184B
ENSG00000155313	76,96568888	87,27630093	7,962124076	-0,178089784	0,002323598	ubiquitin specific peptidase	USP25
ENSG00000155975	48,45513433	54,93597055	7,253561526	-0,178072236	0,041174291	VPS37A subunit of ESCRT-I	VPS37A
ENSG00000151806	33,95364627	38,46882012	6,736272757	-0,177922857	0,002634046	GTP binding elongation fact	GUF1
ENSG00000105698	97,95214488	110,9415142	8,337528591	-0,177725065	0,003163197	upstream transcription fact	USF2
ENSG00000120727	64,61340931	73,2966097	7,700285235	-0,177631446	0,009720689	poly(A) binding protein inter	PAIP2
ENSG00000197265	43,93605496	49,74238938	7,085175934	-0,176971244	0,014282526	general transcription factor	GTF2E2
ENSG00000163875	39,45731672	44,67069313	6,949332207	-0,176928762	0,016962808	MYST/Esa1 associated facto	MEAF6
ENSG00000115145	38,93864184	44,091578	7,026785391	-0,176837927	0,020973795	signal transducing adaptor n	STAM2
ENSG00000171988	68,77880103	77,64248628	7,713661507	-0,176695768	0,031896746	jumonji domain containing 1	JMJD1C
ENSG00000125166	127,5189081	144,4136185	8,649687861	-0,176516321	0,014590631	glutamic-oxaloacetic transar	GOT2
ENSG00000121741	88,6367441	100,2661382	8,214180808	-0,176460113	0,000872824	zinc finger MYM-type contai	ZMYM2
ENSG00000036257	103,8549951	117,6022488	8,393300527	-0,175900685	0,001777529	cullin 3	CUL3
ENSG00000197217	97,34455769	110,1043431	8,369677503	-0,175695324	0,029712249	ectonucleoside triphosphate	ENTPD4
ENSG00000104529	294,5550568	333,6158976	9,968910124	-0,175683176	0,020579062	eukaryotic translation elong	EEF1D
ENSG00000214078	78,03426697	87,93649318	8,11475265	-0,17524182	0,015516164	copine 1	CPNE1
ENSG00000180357	134,9507472	152,4213841	8,666686056	-0,175099638	0,037118272	zinc finger protein 609	ZNF609
ENSG00000136448	177,2852427	200,6083704	9,177043165	-0,174969014	1,14E-05	N-myristoyltransferase 1	NMT1
ENSG00000174953	51,92317195	58,75609782	7,374082879	-0,174891939	0,000586645	DEAH-box helicase 36	DHX36
ENSG00000085721	45,53640144	51,51410426	7,206553284	-0,174679171	0,013488292	RRN3 homolog, RNA polyme	RRN3
ENSG00000144635	40,38831589	45,63235225	6,939678358	-0,174544141	0,017920959	dynein cytoplasmic 1 light ir	DYNC1LI1
ENSG00000163960	97,4003101	110,2073794	8,250132471	-0,17445974	0,03724123	UBX domain protein 7	UBXN7
ENSG00000166167	36,09966442	40,67658231	6,860320639	-0,174051954	0,016978281	beta-transducin repeat cont	BTRC
ENSG00000197912	96,25608301	108,8561539	8,308059945	-0,174017633	0,00116887	SPG7 matrix AAA peptidase	SPG7
ENSG00000136643	25,63263922	28,96158929	6,518721285	-0,173385391	0,022940758	ribosomal protein S6 kinase	RPS6KC1
ENSG00000113552	72,53300439	81,9243553	7,972461135	-0,173260451	0,024455051	glucosamine-6-phosphate d	GNPDA1
ENSG00000055950	38,0326648	42,93012142	7,127028961	-0,173126606	0,006953475	mitochondrial ribosomal pro	MRPL43
ENSG00000168256	77,83233587	87,72562193	8,013526549	-0,173061775	0,01103882	NFKB inhibitor interacting R	NKIRAS2
ENSG00000011007	61,78197589	69,75410833	7,61753251	-0,172756022	0,00764469	elongin A	ELOA
ENSG00000131023	41,45112431	46,79214346	7,037205849	-0,171845774	0,029095657	large tumor suppressor kina	LATS1
ENSG00000104219	64,27755682	72,5129455	7,627199652	-0,171752712	0,042984855	zinc finger DHHC-type palmi	ZDHHC2
ENSG00000275066	78,30167376	88,33910474	8,005090786	-0,171618942	0,000642548	synergin gamma	SYNRG
ENSG00000102572	159,4141265	179,920341	8,855288464	-0,171616227	0,020126918	serine/threonine kinase 24	STK24
ENSG00000025800	146,20357	165,0101377	8,872615044	-0,171605924	0,001720129	karyopherin subunit alpha 6	KPNA6
ENSG00000143437	71,38084361	80,43152158	7,960627853	-0,171201666	0,027837633	aryl hydrocarbon receptor n	ARNT
ENSG00000114650	136,7938309	154,3479051	8,853917167	-0,171123183	0,004060846	SREBF chaperone	SCAP
ENSG00000055130	66,27478094	74,71353731	7,624794221	-0,171088221	0,041713257	cullin 1	CUL1
ENSG00000124659	21,43177143	24,16858352	6,131066167	-0,170659813	0,04956274	tubulin folding cofactor C	TBCC
ENSG00000133835	147,4755879	166,3087692	8,907610531	-0,169868123	0,008915026	hydroxysteroid 17-beta deh	HSD17B4
ENSG00000134077	40,38265606	45,4436646	7,07264192	-0,169837863	0,042975723	THUMP domain containing 3	THUMPD3
ENSG00000025796	97,99961702	110,229354	8,340874307	-0,169606589	0,005685477	SEC63 homolog, protein trai	SEC63
ENSG00000198961	141,8305943	159,5709662	8,893361078	-0,168979514	0,000892554	praja ring finger ubiquitin lig	PJA2
ENSG00000175826	106,6553423	120,1135361	8,494652091	-0,168722925	0,03155677	CTD nuclear envelope phosp	CTDNEP1
ENSG00000110931	70,24301379	78,87980989	7,730686829	-0,168198061	0,007785692	calcium/calmodulin depend	CAMKK2
ENSG00000134644	157,5354326	177,1524176	9,018273437	-0,16783045	0,038278232	pumilio RNA binding family	PUM1
ENSG00000143486	51,20343078	57,61531477	7,387872199	-0,167820565	0,008577945	eukaryotic translation initiat	EIF2D
ENSG00000112763	30,38270479	34,12075619	6,671790586	-0,167683778	0,042444022	butyrophilin subfamily 2 me	BTN2A1
ENSG00000160007	300,8065935	338,5239879	9,928609977	-0,167325045	0,028404732	Rho GTPase activating prote	ARHGAP35
ENSG00000185963	84,38839284	94,99140506	8,069868444	-0,167250334	0,0172734	BICD cargo adaptor 2	BICD2
ENSG00000107290	152,8405705	172,0084198	8,896000939	-0,167249074	0,013496151	senataxin	SETX
ENSG00000125863	23,9942051	26,98327782	6,415313822	-0,167137723	0,011842057	MKKS centrosomal shuttling	MKKS
ENSG00000130779	122,9846467	137,6728744	8,449569224	-0,166842203	0,04559528	CAP-Gly domain containing	CLIP1
ENSG00000166200	60,45171538	67,99786952	7,710703267	-0,166820088	0,011022769	COP9 signalosome subunit 2	COPS2
ENSG00000165055	40,96529909	46,06368285	7,005057953	-0,166486198	0,027907495	methyltransferase 2B, meth	METTL2B
ENSG00000009307	441,155154	493,534609	10,57086289	-0,166423162	0,007514898	cold shock domain containir	CSDE1
ENSG00000105677	58,08284146	65,30485379	7,658752748	-0,166356543	0,043694075	transmembrane protein 147	TMEM147
ENSG00000145741	194,4027961	218,1228999	9,312115457	-0,165177153	0,011860451	basic transcription factor 3	BTF3

ENSG00000116030	92,17443226	103,372534	8,161103976	-0,165073318	0,018338084	small ubiquitin like modifier	SUMO1
ENSG00000125834	79,8906072	89,77626079	7,884482132	-0,165056876	0,004941436	serine/threonine kinase 35	STK35
ENSG00000103035	65,30998967	73,40500828	7,689400083	-0,165005353	0,023918492	proteasome 26S subunit, no	PSMD7
ENSG00000065559	40,08968545	44,92492777	6,978325288	-0,164738499	0,026931418	mitogen-activated protein k	MAP2K4
ENSG00000177885	139,6872708	156,858215	8,755520443	-0,164613073	0,000886174	growth factor receptor bour	GRB2
ENSG00000167193	144,7839212	162,6091731	8,832383773	-0,164473311	0,009393122	CRK proto-oncogene, adapt	CRK
ENSG00000090989	45,21039598	50,77990773	7,244737833	-0,164272418	0,025714328	exocyst complex componen	EXOC1
ENSG00000126777	334,1658276	374,3154433	10,07429639	-0,163582116	0,031805499	kinectin 1	KTN1
ENSG00000006744	96,85853144	108,6866386	8,288334752	-0,163310978	0,034694869	elaC ribonuclease Z 2	ELAC2
ENSG00000101343	33,96226377	38,09327963	6,781918603	-0,163295408	0,032892572	crooked neck pre-mRNA spli	CRNKL1
ENSG00000007080	70,31276396	78,82866316	7,941090009	-0,162315488	0,020492175	coiled-coil domain containin	CCDC124
ENSG00000065000	345,7706481	388,0546149	10,06349715	-0,162058286	0,049385775	adaptor related protein com	AP3D1
ENSG00000145016	54,86900248	61,5239617	7,384473288	-0,16197075	0,013505769	rubicon autophagy regulato	RUBCN
ENSG00000099968	73,16804035	82,00100762	7,973014588	-0,161752048	0,012938703	BCL2 like 13	BCL2L13
ENSG00000105197	48,85857704	54,77900296	7,31517631	-0,161744723	0,024803426	translocase of inner mitoch	TIMM50
ENSG00000147649	152,6382809	171,1356677	8,942794515	-0,161711855	0,024754261	metadherin	MTDH
ENSG00000136754	62,60267656	70,18708304	7,588628882	-0,161362284	0,016388135	abl interactor 1	ABI1
ENSG00000188895	155,4800956	174,2124594	8,879206944	-0,16108892	0,000479822	MSL complex subunit 1	MSL1
ENSG00000166913	321,6237564	360,5936436	9,998398204	-0,161088626	0,01163461	tyrosine 3-monooxygenase/	YWHAB
ENSG00000147439	21,21346211	23,76351786	6,151775108	-0,160911613	0,036569933	bridging integrator 3	BIN3
ENSG00000135926	181,3205579	202,9219381	9,136131538	-0,160712734	0,041869565	transmembrane BAX inhibit	TMBIM1
ENSG00000130382	149,2813721	167,026023	8,936040164	-0,160492409	0,047921413	MLLT1 super elongation con	MLLT1
ENSG00000116903	26,1075765	29,20217218	6,448534537	-0,160398248	0,025923396	exocyst complex componen	EXOC8
ENSG00000081386	19,91592688	22,3127115	6,146203169	-0,160299556	0,011678952	zinc finger protein 510	ZNF510
ENSG00000114062	117,1715655	131,130221	8,561163928	-0,159952143	0,011717284	ubiquitin protein ligase E3A	UBE3A
ENSG00000160767	68,74866424	76,8845157	7,856276059	-0,159567407	0,01639481	family with sequence simila	FAM189B
ENSG00000160551	163,1640197	182,3788381	8,979780082	-0,159078624	0,046160473	TAO kinase 1	TAOK1
ENSG00000116586	41,89736008	46,88339056	7,064639896	-0,158838833	0,030281135	late endosomal/lysosomal a	LAMTOR2
ENSG00000132670	108,2047148	120,8539139	8,345134788	-0,158597376	0,031112334	protein tyrosine phosphatas	PTPRA
ENSG00000112701	97,15256772	108,6688987	8,263500461	-0,158496969	0,016545952	SUMO specific peptidase 6	SENP6
ENSG00000072364	221,4781978	247,1691783	9,449235146	-0,158203069	0,036404525	AF4/FMR2 family member 4	AFF4
ENSG00000091164	56,66807001	63,39379127	7,638484809	-0,158188426	0,032161801	thioredoxin like 1	TXNL1
ENSG00000196531	184,3879291	205,9497913	9,300753583	-0,157951445	0,027034462	nascent polypeptide associa	NACA
ENSG00000198015	45,37488541	50,74012134	7,227824188	-0,157684642	0,018602162	mitochondrial ribosomal pro	MRPL42
ENSG00000168958	54,99744031	61,51959539	7,495873028	-0,15761057	0,02878063	mitochondrial fission factor	MFF
ENSG00000179933	49,28058872	55,0733192	7,317032034	-0,157447856	0,034884519	chromosome 14 open readi	C14orf119
ENSG00000136813	152,3565915	170,2488405	8,86724604	-0,157434366	0,001015802	Ecm29 proteasome adaptor	ECPAS
ENSG00000124164	124,7958156	139,2791459	8,5679732	-0,156743508	0,045354276	VAMP associated protein B a	VAPB
ENSG00000187555	102,4482455	114,395598	8,346846659	-0,156666245	0,002821641	ubiquitin specific peptidase	USP7
ENSG00000066427	33,59571006	37,54065995	6,78760187	-0,156588774	0,046932188	ataxin 3	ATXN3
ENSG00000213799	15,19357756	16,95182114	5,579623943	-0,156371146	0,048245185	zinc finger protein 845	ZNF845
ENSG00000132912	64,53601242	71,98940829	7,882496032	-0,15631657	0,015534069	dynactin subunit 4	DCTN4
ENSG00000145592	193,6370409	215,8680073	9,426592359	-0,156059251	0,044585171	ribosomal protein L37	RPL37
ENSG00000129250	426,0496075	475,6295479	10,33887126	-0,155806344	0,006049163	kinesin family member 1C	KIF1C
ENSG00000050130	30,88372159	34,41120971	6,619089406	-0,155801018	0,026776627	JNK1/MAPK8 associated me	JKAMP
ENSG00000198799	27,77466386	30,99226183	6,555765262	-0,155242706	0,040052245	leucine rich repeats and imn	LRIG2
ENSG00000134313	131,3558036	145,3806839	8,757011615	-0,154633664	0,035494979	kinase D interacting substra	KIDINS220
ENSG00000119509	27,14956586	30,2287911	6,413915711	-0,154489271	0,040661743	inversin	INVS
ENSG00000156515	245,2380966	273,5331936	9,710790648	-0,154426099	0,001134803	hexokinase 1	HK1
ENSG00000215271	41,76370989	46,56210776	7,003765004	-0,153424212	0,021631439	homeobox and leucine zippe	HOMEZ
ENSG00000101558	129,872161	144,7682682	8,684252706	-0,153220327	0,004441706	VAMP associated protein A	VAPA
ENSG00000109756	50,94466275	56,6122166	7,403904056	-0,151600729	0,030074718	Rap guanine nucleotide exch	RAPGEF2
ENSG00000204713	80,61437912	89,6272248	8,030750724	-0,151004206	0,021713841	tripartite motif containing 2	TRIM27
ENSG00000150459	97,11188603	107,9852412	8,266661311	-0,150110871	0,025090056	Sin3A associated protein 18	SAP18
ENSG00000137275	72,74950195	80,86839626	7,7354008	-0,14947594	0,019260861	receptor interacting serine/t	RIPK1
ENSG00000100225	86,52476239	96,10872291	8,075170266	-0,149265515	0,022008372	F-box protein 7	FBXO7
ENSG00000172531	194,1200248	215,8169459	9,237555743	-0,149179201	0,032535258	protein phosphatase 1 catal	PPP1CA
ENSG00000181027	44,05617447	48,92143346	7,151838887	-0,149052091	0,044380369	fukutin related protein	FKRP
ENSG00000125818	100,5910331	111,7820462	8,513631192	-0,148871805	0,000593396	proteasome inhibitor subun	PSMF1
ENSG00000153827	322,3138219	357,9868337	10,01099891	-0,148696324	0,000512849	thyroid hormone receptor in	TRIP12
ENSG00000100804	104,0932462	115,1224697	8,372556784	-0,148497087	0,044887899	proteasome 20S subunit bet	PSMB5
ENSG00000213995	44,93440602	49,82711143	7,334161673	-0,148247639	0,004441706	NAD(P)HX dehydratase	NAXD
ENSG00000197226	279,5251571	310,1228468	9,833493357	-0,148239584	0,000502999	TBC1 domain family membe	TBC1D9B
ENSG00000185728	104,1781535	115,5164732	8,448943652	-0,147817245	0,005995234	YTH N6-methyladenosine RN	YTHDF3
ENSG00000068323	137,223058	152,1172618	8,840700003	-0,147681681	0,019000098	transcription factor binding	TFE3
ENSG00000143515	96,74656306	106,8675812	8,191298402	-0,1476268	0,032287945	ATPase phospholipid transp	ATP8B2
ENSG00000134108	84,29898137	93,56306943	8,059052233	-0,147304565	0,005440914	ADP ribosylation factor like	ARL8B
ENSG00000142453	98,57373638	109,0556348	8,318325013	-0,146970515	0,041174291	coactivator associated argin	CARM1
ENSG00000141580	93,97748459	104,1338102	8,224682176	-0,146962859	0,016382856	WD repeat domain 45B	WDR45B
ENSG00000099817	133,1662485	147,7569405	8,736356313	-0,146766515	0,043192637	RNA polymerase II, I and III s	POLR2E
ENSG00000126934	143,634988	159,3966238	8,940114255	-0,146661101	0,006726207	mitogen-activated protein k	MAP2K2
ENSG00000117859	112,3511233	124,4995684	8,466495606	-0,146611226	0,007144177	oxysterol binding protein lik	OSBPL9
ENSG00000069329	176,1245944	195,1442124	9,156902938	-0,146495524	0,021259033	VPS35 retromer complex co	VPS35
ENSG00000130985	413,89106	457,1851199	10,38338915	-0,146046137	0,031870307	ubiquitin like modifier activa	UBA1
ENSG00000171853	55,2436202	61,24593219	7,561972044	-0,145930555	0,037983472	trafficking protein particle c	TRAPPC12
ENSG00000204227	81,47997683	90,3334714	8,11885127	-0,145724031	0,026831476	ring finger protein 1	RING1
ENSG00000213281	109,9835233	121,8185305	8,436521659	-0,145530341	0,042752241	NRAS proto-oncogene, GTPa	NRAS
ENSG00000088832	182,6470625	201,8425928	9,145256812	-0,144560616	0,026998743	FKBP prolyl isomerase 1A	FKBP1A
ENSG00000084093	89,19117706	98,83262344	8,208871857	-0,144546888	0,023670723	RE1 silencing transcription fa	REST
ENSG00000134452	110,1913817	121,9250876	8,456023799	-0,144423735	0,04324319	F-box DNA helicase 1	FBH1
ENSG00000182473	193,0722703	213,3391609	9,384495389	-0,144400175	0,013968505	exocyst complex componen	EXOC7
ENSG00000135392	43,75021613	48,43002802	7,20219433	-0,144399942	0,010329789	DnaJ heat shock protein fam	DNAJC14
ENSG00000166326	158,3096434	175,0850097	9,004151017	-0,144285806	0,002406971	tripartite motif containing 4	TRIM44
ENSG00000119396	105,2427301	116,2338049	8,365547106	-0,142647724	0,046332487	RAB14, member RAS oncoge	RAB14
ENSG00000118579	66,6203183	73,7649863	7,703659255	-0,142182811	0,045733898	mediator complex subunit 2	MED28
ENSG00000110925	58,27632192	64,34258993	7,496398077	-0,140421156	0,0192037	cysteine and serine rich nucl	CSRNP2
ENSG00000172939	93,00424305	102,6668992	8,206420659	-0,1401453	0,002439125	oxidative stress responsive k	OXSR1
ENSG00000161204	55,890125	61,66922405	7,631374889	-0,138801864	0,028209981	ATP binding cassette subfam	ABCF3
ENSG00000131653	148,5893414	163,5935464	8,966873225	-0,138542236	0,030126238	TNF receptor associated fact	TRAF7
ENSG00000119041	45,39283884	50,0528744	7,13999385	-0,138276857	0,017328711	general transcription factor	GTF3C3
ENSG00000116731	51,25692612	56,28384135	7,32921325	-0,137828945	0,041723511	PR/SET domain 2	PRDM2
ENSG00000159348	41,66615903	45,84807635	7,251521635	-0,137743791	0,042673364	cytochrome b5 reductase 1	CYB5R1
ENSG00000115207	60,02366338	66,13469229	7,600383356	-0,137727413	0,040705099	general transcription factor	GTF3C2
ENSG00000115234	78,05948394	86,05932417	8,078910431	-0,137643982	0,005666418	sorting nexin 17	SNX17
ENSG00000100296	46,86530897	51,61988526	7,241230505	-0,137434026	0,048245185	THO complex 5	THOC5
ENSG00000105063	194,1987244	213,569469	9,211842185	-0,135599347	0,030814939	protein phosphatase 6 regul	PPP6R1
ENSG00000129292	70,33314304	77,43794503	7,823182297	-0,135530619	0,020626276	PHD finger protein 20 like 1	PHF20L1
ENSG00000141002	84,05361865	92,52636479	8,136676633	-0,135403517	0,002819748	transcription factor 25	TCF25
ENSG00000015676	113,3407881	124,3827215	8,511622613	-0,132100617	0,026916433	NudC domain containing 3	NUDCD3
ENSG00000110075	131,1472304	144,0161222	8,646466769	-0,131698052	0,005427202	protein phosphatase 6 regul	PPP6R3
ENSG00000275052	89,52641978	98,30310552	8,193659023	-0,13147638	0,026011668	protein phosphatase 4 regul	PPP4R3B
ENSG00000108107	355,8830223	390,4393409	10,2369507	-0,130458135	0,047806984	ribosomal protein L28	RPL28
ENSG00000109332	212,6315176	233,0976204	9,403728463	-0,129068154	0,029841199	ubiquitin conjugating enzym	UBE2D3
ENSG00000092108	44,78567435	49,0672722	7,184482294	-0,128411891	0,042792022	sec1 family domain containi	SCFD1
ENSG00000164164	74,39740305	81,39710451	7,877498708	-0,127835733	0,034791089	OTU deubiquitinase 4	OTUD4
ENSG00000174903	253,2127008	276,7644949	9,649143017	-0,127245722	0,039657975	RAB1B, member RAS oncoge	RAB1B
ENSG00000021776	66,62967745	72,96361087	7,66281628	-0,127125059	0,007150911	aquarius intron-binding splic	AQR
ENSG00000156599	181,2983661	198,1742529	9,124126781	-0,126243051	0,045011958	zinc finger DHHC-type palmi	ZDHHC5
ENSG00000055070	213,6725097	233,3328454	9,376282292	-0,125855613	0,041729039	SUZ RNA binding domain co	SZRD1
ENSG00000028528	112,7910409	123,3012304	8,502565001	-0,125379265	0,020734771	sorting nexin 1	SNX1
ENSG00000049656	117,5508974	128,4443723	8,588557685	-0,124865629	0,020958336	CLPTM1 like	CLPTM1L
ENSG00000183576	74,00637387	80,87153097	7,80296302	-0,12412177	0,008337928	SET domain containing 3, ac	SETD3
ENSG00000103275	140,2324947	153,1532681	8,761182579	-0,123869752	0,002431754	ubiquitin conjugating enzym	UBE2I
ENSG00000144021	86,05343893	93,91829843	8,139735171	-0,122863776	0,021285779	cytosolic iron-sulfur assemb	CIAO1
ENSG00000166266	48,50443577	52,8886429	7,28821414	-0,122642808	0,032408578	cullin 5	CUL5

ENSG00000158161	53,21399052	58,04407103	7,350552312	-0,121646804	0,012526917	EYA transcriptional coactiva	EYA3
ENSG00000113845	66,73158411	72,71017795	7,806763857	-0,12127192	0,046347195	translocase of inner mitochc	TIMMDC1
ENSG00000114354	129,4814418	141,1135894	8,637999598	-0,121238942	0,025941806	trafficking from ER to golgi r	TFG
ENSG00000180957	74,65627409	81,34512175	7,853567326	-0,120348691	0,04142346	phosphatidylinositol transfe	PITPNB
ENSG00000013374	80,55269265	87,64192323	8,064738057	-0,118734262	0,021566888	negative regulator of ubiqui	NUB1
ENSG00000119541	76,41404237	83,12536476	7,921321393	-0,118686908	0,037978268	vacuolar protein sorting 4 h	VPS4B
ENSG00000118873	74,45467592	81,02037764	7,893499758	-0,118138981	0,014224487	RAB3 GTPase activating non	RAB3GAP2
ENSG00000008294	133,6181358	145,0404357	8,683743976	-0,115719068	0,00735813	sperm associated antigen 9	SPAG9
ENSG00000182952	45,32841639	49,21921221	7,114937858	-0,115367174	0,026562729	high mobility group nucleos	HMGN4
ENSG00000185651	108,0505016	116,8680827	8,339649425	-0,111810139	0,033510589	ubiquitin conjugating enzym	UBE2L3
ENSG00000257103	136,5231696	147,6477127	8,833159926	-0,110117222	0,006542732	LSM14A mRNA processing b	LSM14A
ENSG00000165637	95,30422809	102,937809	8,32986507	-0,107677573	0,048991887	voltage dependent anion ch	VDAC2
ENSG00000170027	320,3570736	344,7511021	9,963520289	-0,105409729	0,048147098	tyrosine 3-monooxygenase/	YWHAG
ENSG00000067900	134,0614915	144,5486219	8,704736594	-0,105224821	0,044537764	Rho associated coiled-coil cc	ROCK1
ENSG00000179010	290,8705725	312,9442567	9,885011245	-0,103839595	0,009914343	Morf4 family associated pro	MRFAP1
ENSG00000107816	171,2923878	183,612965	9,05267486	-0,103256301	0,019634833	leucine zipper tumor suppre	LZTS2
ENSG00000135387	404,9554695	385,7927618	10,25980055	0,073252835	0,033756303	cell cycle associated protein	CAPRIN1
ENSG00000142864	410,490292	390,2850888	10,27398927	0,076008824	0,029980251	SERPINE1 mRNA binding prc	SERBP1
ENSG00000165119	538,0700947	511,246576	10,62745845	0,076199118	0,019305698	heterogeneous nuclear ribo	HNRNPK
ENSG00000038358	100,9192487	94,88711748	8,219700015	0,090734461	0,039459974	enhancer of mRNA decappir	EDC4
ENSG00000266412	198,5595724	185,5728847	9,279049503	0,100832199	0,033658629	nuclear receptor coactivator	NCOA4
ENSG00000065243	87,52289101	81,45628446	7,992866777	0,107316185	0,048288259	protein kinase N2	PKN2
ENSG00000165934	97,07442752	90,09539674	8,223049929	0,107718256	0,04252378	cleavage and polyadenylatic	CPSF2
ENSG00000134910	204,0637683	189,5850743	9,271009932	0,109269344	0,020046907	STT3 oligosaccharyltransfera	STT3A
ENSG00000149930	166,9514764	154,2994293	9,103001972	0,115592175	0,031799677	TAO kinase 2	TAOK2
ENSG00000136504	61,03065114	56,2997784	7,516755582	0,116692293	0,020993537	lysine acetyltransferase 7	KAT7
ENSG00000142208	263,8570878	243,1793981	9,53471064	0,120392708	0,011707383	AKT serine/threonine kinase	AKT1
ENSG00000011304	417,5175731	383,6961993	10,1725071	0,121228947	0,04625345	polypyrimidine tract binding	PTBP1
ENSG00000153187	736,0944822	677,4828044	11,03395605	0,12177213	1,05E-05	heterogeneous nuclear ribo	HNRNPU
ENSG00000115073	93,79874639	86,39692462	8,189302222	0,122028951	0,013006217	actin related protein 1B	ACTR1B
ENSG00000213676	147,818835	136,1781263	8,854461575	0,122091175	0,013158743	activating transcription facto	ATF6B
ENSG00000112531	185,3939022	170,7955334	9,129186785	0,122094051	0,017026867	QKI, KH domain containing F	QKI
ENSG00000100266	157,4298154	144,7545952	8,772317828	0,123984464	0,020575048	protein kinase C and casein	PACSIN2
ENSG00000162244	221,1047317	203,176195	9,648244836	0,124786836	0,045606906	ribosomal protein L29	RPL29
ENSG00000132256	59,75739052	54,73297196	7,428125338	0,126820681	0,039118606	tripartite motif containing 5	TRIM5
ENSG00000205937	215,5407561	197,2371205	9,264073964	0,129041085	0,018951705	RNA binding protein with se	RNPS1
ENSG00000137845	291,5960335	266,3372462	9,887822347	0,130313318	0,048245185	ADAM metallopeptidase doi	ADAM10
ENSG00000102786	61,05231367	55,77742382	7,434230375	0,130474476	0,049819075	integrator complex subunit	INTS6
ENSG00000123562	187,0079934	171,0349703	9,187356533	0,132073671	0,016726908	mortality factor 4 like 2	MORF4L2
ENSG00000138594	173,7500201	158,8762241	9,085060131	0,132229147	0,024204621	tropomodulin 3	TMOD3
ENSG00000089597	670,9774914	612,9093696	10,96178324	0,134423871	0,026902503	glucosidase II alpha subunit	GANAB
ENSG00000106682	371,0480576	338,0511533	10,12595264	0,138188481	0,014821687	eukaryotic translation initiat	EIF4H
ENSG00000129351	420,6425849	382,3750308	10,22235822	0,138473953	0,006652344	interleukin enhancer binding	ILF3
ENSG00000102316	158,1284904	143,9690499	9,165468551	0,139582552	0,049245597	MAGE family member D2	MAGED2
ENSG00000265354	51,22910277	46,54566865	7,220178945	0,140402818	0,03646392	translocase of inner mitochc	TIMM23
ENSG00000228474	87,95390086	79,90073542	8,13071072	0,140494002	0,046801276	oligosaccharyltransferase cc	OST4
ENSG00000077721	100,5699014	91,28308887	8,146303615	0,142014592	0,035492592	ubiquitin conjugating enzym	UBE2A
ENSG00000105576	147,5060983	133,8741498	8,859921536	0,143240654	0,029553578	transportin 2	TNPO2
ENSG00000163939	119,101912	108,0758641	8,423040753	0,144157295	0,04252378	polybromo 1	PBRM1
ENSG00000170144	372,8858381	335,9549745	9,983652871	0,147938518	0,005280949	heterogeneous nuclear ribo	HNRNPA3
ENSG00000119638	152,1898443	137,7216041	8,941990345	0,148692407	0,031344743	NIMA related kinase 9	NEK9
ENSG00000086061	257,4121747	232,3980748	9,406059246	0,14987657	0,03924155	DnaJ heat shock protein fan	DNAJA1
ENSG00000136938	218,3395252	196,6225216	9,260450677	0,150549628	0,010061991	acidic nuclear phosphoprote	ANP32B
ENSG00000172340	77,76455009	69,96507187	7,909216128	0,151211828	0,022174273	succinate-CoA ligase GDP-fo	SUCLG2
ENSG00000177613	68,33027143	61,58585519	7,658705037	0,151730816	0,023362152	cleavage stimulation factor	CSTF2T
ENSG00000178691	105,1930608	94,21576277	8,192949254	0,151768302	0,016005922	SUZ12 polycomb repressive	SUZ12
ENSG00000135486	577,0426226	521,3847756	10,72802458	0,151834077	0,040596397	heterogeneous nuclear ribo	HNRNPA1
ENSG00000103507	130,3264341	117,1557019	8,722684881	0,152524523	0,025862909	branched chain keto acid de	BCKDK
ENSG00000115677	893,0274126	805,1124522	11,49311254	0,153278692	0,041428572	high density lipoprotein binc	HDLBP
ENSG00000143033	36,34372126	32,73842978	6,771174937	0,153713475	0,02559698	metal response element bin	MTF2
ENSG00000136925	31,78690362	28,61978714	6,760391326	0,154270107	0,01226463	thiosulfate sulfurtransferase	TSTD2
ENSG00000144401	16,85190932	15,16135649	5,540225071	0,15500399	0,03742913	methyltransferase 21A, HSP	METTL21A
ENSG00000136527	128,9405662	115,9303309	8,473576149	0,155394407	0,010647127	transformer 2 beta homolog	TRA2B
ENSG00000163029	94,37358414	84,68053064	8,057262669	0,157349084	0,001637965	structural maintenance of cl	SMC6
ENSG00000196504	169,4839873	151,80192	8,931814813	0,157478935	0,005528042	pre-mRNA processing factor	PRPF40A
ENSG00000182749	60,50032184	54,30717213	7,578592669	0,158867084	0,010037742	progestin and adipoQ recep	PAQR7
ENSG00000100281	40,64480748	36,47404949	6,915633584	0,159023485	0,040326672	HMG-box containing 4	HMGXB4
ENSG00000152242	44,47974835	39,62453411	7,060498615	0,16246678	0,026804302	chromosome 18 open readi	C18orf25
ENSG00000177045	61,75185648	55,16687059	7,633000214	0,162800582	0,00781742	SIX homeobox 5	SIX5
ENSG00000188612	156,9713059	140,2626394	8,885020687	0,165121276	0,008703847	small ubiquitin like modifier	SUMO2
ENSG00000099917	158,0504706	141,1385741	8,87417114	0,165238497	0,003084583	mediator complex subunit 1	MED15
ENSG00000077549	211,0653746	188,4378981	9,415380075	0,165964955	0,025125327	capping actin protein of mu:	CAPZB
ENSG00000181090	123,9586896	110,7306929	8,492328794	0,166107849	0,007700789	euchromatic histone lysine r	EHMT1
ENSG00000140262	123,225588	110,0723849	8,54489159	0,166147126	0,002508242	transcription factor 12	TCF12
ENSG00000126254	91,93448109	81,89671477	8,139618512	0,166350116	0,02603062	RNA binding motif protein 4	RBM42
ENSG00000197043	355,5918477	317,5119448	9,975844573	0,16646822	0,03454312	annexin A6	ANXA6
ENSG00000116497	37,65443117	33,59800347	6,689010951	0,167344707	0,021713841	S100P binding protein	S100PBP
ENSG00000143398	120,3289943	107,3445636	8,446373998	0,16748882	0,004083387	phosphatidylinositol-4-phos	PIP5K1A
ENSG00000139746	72,056888	64,27896484	7,725987922	0,167761028	0,025290585	RNA binding motif protein 2	RBM26
ENSG00000068438	56,88294305	50,7427355	7,397086839	0,168170405	0,018470819	FtsJ RNA 2'-O-methyltransfe	FTSJ1
ENSG00000182872	158,4474108	141,0736924	8,835088904	0,168237148	0,009095639	RNA binding motif protein 1	RBM10
ENSG00000130766	125,9160633	112,2434051	8,591393416	0,168753388	0,039204617	sestrin 2	SESN2
ENSG00000138780	37,03791425	32,84635947	6,860184789	0,168868841	0,017668901	glutathione S-transferase C-	GSTCD
ENSG00000105135	56,19342481	50,08164957	7,528551453	0,169475509	0,013780922	ilvB acetolactate synthase lil	ILVBL
ENSG00000188529	109,3307485	97,3110652	8,238314343	0,169636733	0,010778873	serine and arginine rich splic	SRSF10
ENSG00000204435	21,35378944	19,04034829	6,014305872	0,169775995	0,036671203	casein kinase 2 beta	CSNK2B
ENSG00000101361	125,4521337	111,5738489	8,459555618	0,170458321	0,032018988	NOP56 ribonucleoprotein	NOP56
ENSG00000175115	170,8810893	152,0771193	8,945930368	0,170946494	0,013247375	phosphofurin acidic cluster :	PACS1
ENSG00000101811	33,48478278	29,70940108	6,593935431	0,172076254	0,026652845	cleavage stimulation factor :	CSTF2
ENSG00000204371	139,5803423	124,1145465	8,834582595	0,172113148	0,003838989	euchromatic histone lysine r	EHMT2
ENSG00000136699	121,3118018	107,7934865	8,420324693	0,172159059	0,007769838	sphingomyelin phosphodies	SMPD4
ENSG00000185024	59,60346422	52,8945903	7,476745228	0,172167917	0,005803008	BRF1 RNA polymerase III tra	BRF1
ENSG00000108578	60,90007008	54,16676234	7,405299763	0,172277335	0,008703847	bleomycin hydrolase	BLMH
ENSG00000162368	112,3227999	99,86409522	8,366691693	0,172494133	0,009199756	cytidine/uridine monophosp	CMPK1
ENSG00000167461	109,3255268	96,81570311	8,316072661	0,173199705	0,010291029	RAB8A, member RAS oncog	RAB8A
ENSG00000113658	123,0833406	109,3319716	8,554809893	0,173639005	0,002717654	SMAD family member 5	SMAD5
ENSG00000087502	65,74976816	58,41203374	7,618832029	0,173695495	0,017418326	ERGIC and golgi 2	ERGIC2
ENSG00000130770	122,7441535	109,0309872	8,557562916	0,173737842	0,009702233	ATP synthase inhibitory fact	ATP5IF1
ENSG00000167674	196,2456431	174,2627168	9,100637102	0,174450378	0,004350089	HDGF like 2	HDGFL2
ENSG00000110583	47,85680137	42,5025117	7,159308632	0,175116805	0,013832998	N-alpha-acetyltransferase 4(	NAA40
ENSG00000125844	457,6098118	406,2356704	10,53191234	0,175449544	0,020636212	ribosome binding protein 1	RRBP1
ENSG00000088038	93,34655917	82,73035873	8,131268452	0,175818185	0,001720129	CCR4-NOT transcription con	CNOT3
ENSG00000023041	63,61378333	56,3407839	7,460794012	0,175939548	0,00146565	zinc finger DHHC-type palmi	ZDHHC6
ENSG00000138381	39,73311256	35,05417558	6,945715172	0,176424229	0,039554272	asparagine synthetase doma	ASNSD1
ENSG00000101457	29,20244402	25,87103494	6,430469737	0,177449003	0,017228063	deoxynucleotidyltransferase	DNTTIP1
ENSG00000083642	79,62264741	70,53504506	7,857483382	0,178067121	0,0054023	PDS5 cohesin associated fac	PDS5B
ENSG00000172845	125,2269285	110,4719821	8,394605241	0,178644297	0,005650696	Sp3 transcription factor	SP3
ENSG00000100372	22,67042625	20,02124277	5,976267774	0,178817161	0,013915042	solute carrier family 25 men	SLC25A17
ENSG00000132952	20,31491042	17,95780862	5,965665353	0,179812393	0,03441521	ubiquitin specific peptidase	USPL1
ENSG00000186318	191,0895097	168,8859816	9,09534395	0,179815388	0,010991456	beta-secretase 1	BACE1
ENSG00000167283	76,90107591	68,02873783	7,924360855	0,180162146	0,042843331	ATP synthase membrane sul	ATP5MG
ENSG00000084463	139,004581	122,6406043	8,741640006	0,180176662	0,02085622	WW domain binding proteir	WBP11
ENSG00000124767	144,1473428	127,2096032	8,720205586	0,180802414	0,038677861	glyoxalase I	GLO1
ENSG00000010803	37,81398028	33,34464766	6,738794835	0,181081019	0,024126881	Scm polycomb group protei	SCMH1
ENSG00000159082	28,02321752	24,6844846	6,418141294	0,181087744	0,013342659	synaptojanin 1	SYNJ1

ENSG00000096401	96,7077605	85,18270947	8,152266592	0,181776173	0,007044209	cell division cycle 5 like	CDC5L
ENSG00000112473	167,2636605	147,6911453	9,140378584	0,181796997	0,003446607	solute carrier family 39 men	SLC39A7
ENSG00000157978	41,83933036	36,88882817	6,806532785	0,181798234	0,043371582	low density lipoprotein rece	LDLRAP1
ENSG00000135597	35,1394722	31,00393509	6,663232323	0,182083226	0,041428277	RALBP1 associated Eps dom	REPS1
ENSG00000159788	55,68098059	49,17407576	7,249240864	0,182327064	0,018252623	regulator of G protein signal	RGS12
ENSG00000089693	233,4739988	205,5259951	9,478882877	0,183011849	0,024650889	myeloid leukemia factor 2	MLF2
ENSG00000072415	59,991713	52,78005778	7,470367075	0,183045566	0,00598108	protein associated with LIN7	PALS1
ENSG00000137274	20,14143522	17,69525705	6,07666708	0,183290781	0,02644417	biphenyl hydrolase like	BPHL
ENSG00000128534	32,17606405	28,39520092	6,463337165	0,183537998	0,001708632	LSM8 homolog, U6 small nu	LSM8
ENSG00000128513	35,01923066	30,84558887	6,634960391	0,183844603	0,013131665	protection of telomeres 1	POT1
ENSG00000145725	43,75134734	38,56792902	7,099576176	0,183850253	0,04101387	diphosphoinositol pentakisp	PPIP5K2
ENSG00000100320	207,6980612	183,2315599	9,282191897	0,18424043	0,003190797	RNA binding fox-1 homolog	RBFOX2
ENSG00000101160	408,3398896	360,358329	10,23449206	0,18479302	0,024565526	cathepsin Z	CTSZ
ENSG00000179889	122,444972	107,9764808	8,549042494	0,184822735	0,000229871	pyridoxal dependent decarb	PDXDC1
ENSG00000135945	51,22196478	44,93613625	7,155811236	0,185747971	0,015938622	REV1 DNA directed polymer	REV1
ENSG00000107625	75,40766123	66,3413	7,75600753	0,186366305	0,000426011	DExD-box helicase 50	DDX50
ENSG00000143458	21,84169502	19,13136038	5,975858813	0,186450822	0,036902013	GA binding protein transcrip	GABPB2
ENSG00000100150	27,11917005	23,76976651	6,331878679	0,186999905	0,003290137	DEP domain containing 5, G.	DEPDC5
ENSG00000176953	107,1040776	93,45203035	8,221893087	0,187864824	0,001923579	nuclear factor of activated T	NFATC2IP
ENSG00000132792	38,95061292	34,16934129	6,890637573	0,188636174	0,045213676	catenin beta like 1	CTNNBL1
ENSG00000134453	103,1044685	90,29855098	8,133439946	0,189940515	0,002265858	RNA binding motif protein 1	RBM17
ENSG00000198824	74,1750533	64,79704239	7,659305701	0,190380658	0,002769893	chromosome alignment mai	CHAMP1
ENSG00000144580	60,00241157	52,68050238	7,365379705	0,190993126	0,013256022	CCR4-NOT transcription con	CNOT9
ENSG00000163781	102,634436	89,96647172	8,127585144	0,191218446	0,028397311	DNA topoisomerase II bindir	TOPBP1
ENSG00000138814	104,2721945	91,32062111	8,408923644	0,191568877	0,020372071	protein phosphatase 3 catal	PPP3CA
ENSG00000094975	91,89648139	80,60749033	7,998252486	0,191612789	0,008236017	SUN domain containing ossi	SUCO
ENSG00000138231	26,39838817	23,16886219	6,178913836	0,192271923	0,036704761	debranching RNA lariats 1	DBR1
ENSG00000205531	196,3632516	171,9495357	9,106457119	0,192462635	0,001702956	nucleosome assembly prote	NAP1L4
ENSG00000205352	34,71125072	30,36246694	6,703520225	0,19277474	0,001584606	proline rich 13	PRR13
ENSG00000088247	453,9598372	397,3290661	10,31173103	0,193476784	0,001649399	KH-type splicing regulatory p	KHSRP
ENSG00000239306	90,41217367	78,96563718	7,982515428	0,194092186	0,000150098	RNA binding motif protein 1	RBM14
ENSG00000111605	94,9362039	83,21933528	8,064485002	0,19414952	0,000956275	cleavage and polyadenylatic	CPSF6
ENSG00000112419	64,36979189	56,35824968	7,474163059	0,194563348	0,016216365	phosphatase and actin regul	PHACTR2
ENSG00000137200	69,10131748	60,33211194	7,74995836	0,194926104	0,027741677	cap methyltransferase 1	CMTR1
ENSG00000067225	2216,64661	1940,904174	12,72004948	0,194935222	0,008595903	pyruvate kinase M1/2	PKM
ENSG00000168090	125,0003038	109,1837269	8,545078833	0,194972719	0,006586212	COP9 signalosome subunit 6	COPS6
ENSG00000129636	93,26473386	81,65597467	8,11662394	0,195014839	0,007675821	integrin alpha FG-GAP repea	ITFG1
ENSG00000175662	103,0709623	90,16558234	8,359099878	0,195346529	0,024729918	target of myb1 like 2 membr	TOM1L2
ENSG00000244462	103,7212837	90,66914915	8,164926125	0,195413772	0,002243141	RNA binding motif protein 1	RBM12
ENSG00000125944	198,2810108	173,1563961	9,162591372	0,195490215	0,000178952	heterogeneous nuclear ribo	HNRNPR
ENSG00000126698	126,4832189	110,5135082	8,507432602	0,196266515	0,001789326	DnaJ heat shock protein fam	DNAJC8
ENSG00000104824	197,0927897	172,3092947	9,094084512	0,196892245	0,016889601	heterogeneous nuclear ribo	HNRNPL
ENSG00000162613	171,5942253	149,6370344	8,996385926	0,197269717	0,003395425	far upstream element bindir	FUBP1
ENSG00000060749	104,4654197	91,29291243	8,180171567	0,198463473	0,000831835	glutamine and serine rich 1	QSER1
ENSG00000095485	30,20929675	26,12091193	6,431105363	0,198649738	0,011740979	CWF19 like cell cycle contro	CWF19L1
ENSG00000183648	23,63839695	20,5771823	6,232539204	0,198781559	0,023554979	NADH:ubiquinone oxidored	NDUFB1
ENSG00000101413	56,41325548	49,17309144	7,331230875	0,199074724	0,010858161	regulation of nuclear pre-ml	RPRD1B
ENSG00000174547	37,742533	32,87668969	6,734020591	0,199315617	0,036915316	mitochondrial ribosomal prc	MRPL11
ENSG00000187514	663,7507286	578,8897076	10,87807027	0,199360085	0,007584976	prothymosin alpha	PTMA
ENSG00000148688	26,8185233	23,27740519	6,241916398	0,199946969	0,04323001	ribonuclease P/MRP subunit	RPP30
ENSG00000115825	72,28621008	63,1209873	7,650008205	0,20000684	0,00764469	protein kinase D3	PRKD3
ENSG00000134001	104,1719333	90,34795717	8,228980147	0,20006546	0,042495501	eukaryotic translation initiat	EIF2S1
ENSG00000244038	246,6884614	215,0616245	9,624006987	0,200702115	0,027855591	dolichyl-diphosphooligosacc	DDOST
ENSG00000204576	20,28002969	17,6822711	5,971068127	0,201348022	0,007024471	proline rich 3	PRR3
ENSG00000184517	18,87347379	16,40635278	5,799586058	0,201807477	0,018748078	ZFP1 zinc finger protein	ZFP1
ENSG00000169992	174,1492829	151,7193509	9,122523897	0,202099763	0,035085404	neuroligin 2	NLGN2
ENSG00000230124	46,94518795	40,89988752	7,101455583	0,202231246	0,024176727	acyl-CoA binding domain coı	ACBD6
ENSG00000179218	1135,690318	988,3978783	11,77443683	0,202313266	0,000992005	calreticulin	CALR
ENSG00000167693	234,182495	203,7910402	9,46541523	0,202728883	0,00690557	nucleoredoxin	NXN
ENSG00000129691	50,03519542	43,42245586	7,223735407	0,203015522	0,026509588	ASH2 like, histone lysine me	ASH2L
ENSG00000100105	53,25368456	46,29129019	7,360062046	0,203874653	0,007554216	POZ/BTB and AT hook conta	PATZ1
ENSG00000137312	213,0466733	185,03964	9,416084268	0,204036945	0,007182322	flotillin 1	FLOT1
ENSG00000001497	57,81000542	50,12820674	7,43427065	0,204268361	0,014907214	LAS1 like ribosome biogenes	LAS1L
ENSG00000120963	43,73806914	38,03229435	6,924512603	0,204449475	0,009995743	zinc finger protein 706	ZNF706
ENSG00000112941	46,9296268	40,81091762	6,990921963	0,204580929	0,007252973	terminal nucleotidyltransfer	TENT4A
ENSG00000145022	46,11412996	40,10692736	7,160571617	0,205145235	0,010730498	T cell leukemia translocatior	TCTA
ENSG00000170315	440,9504856	383,1957351	10,31543116	0,205243285	0,010506744	ubiquitin B	UBB
ENSG00000058262	481,5949607	418,734805	10,50382913	0,205633228	0,007706027	SEC61 translocon subunit al	SEC61A1
ENSG00000160305	94,65847926	81,99413901	8,044700501	0,205809956	0,00347448	disco interacting protein 2 h	DIP2A
ENSG00000079691	64,24727737	55,62551292	7,436307764	0,205903887	0,014098892	capping protein regulator ar	CARMIL1
ENSG00000132323	29,12783539	25,26895613	6,329803317	0,206243509	0,018704526	ILK associated serine/threor	ILKAP
ENSG00000137822	60,6066683	52,66694411	7,321138082	0,206306575	0,025213517	tubulin gamma complex ass	TUBGCP4
ENSG00000105323	553,0744155	479,7793185	10,58424668	0,206949588	0,006753815	heterogeneous nuclear ribo	HNRNPUL1
ENSG00000158882	25,9497247	22,49951037	6,428427305	0,2070525	0,017622692	translocase of outer mitoch	TOMM40L
ENSG00000082898	272,0873494	235,1530624	9,605526886	0,207939147	0,001300665	exportin 1	XPO1
ENSG00000108518	707,1990939	613,0303985	10,95495602	0,207974213	0,014683904	profilin 1	PFN1
ENSG00000179456	25,00598504	21,67381543	6,310548501	0,20891769	0,015793254	zinc finger and BTB domain	ZBTB18
ENSG00000012660	156,3906239	135,5282543	8,739971285	0,209114069	0,048295631	ELOVL fatty acid elongase 5	ELOVL5
ENSG00000104983	25,26575374	21,77317308	6,233822378	0,209239709	0,031317304	coiled-coil domain containin	CCDC61
ENSG00000132024	88,24412239	76,39818041	8,089328904	0,209610136	0,000125073	coiled-coil and C2 domain cc	CC2D1A
ENSG00000131759	76,65849474	66,34191664	7,781637156	0,209741465	0,01369652	retinoic acid receptor alpha	RARA
ENSG00000087088	233,9751792	202,4854234	9,48975774	0,209746038	0,002013564	BCL2 associated X, apoptosi	BAX
ENSG00000198324	59,45125359	51,53200569	7,468375117	0,210485012	0,048530149	PH domain containing endo	PHETA1
ENSG00000100092	47,44987605	41,02024448	6,929753278	0,210518277	0,031955395	SH3 domain binding protein	SH3BP1
ENSG00000167543	80,62361649	69,5802887	7,756517009	0,210594366	0,020891609	tumor protein p53 inducible	TP53I13
ENSG00000138614	47,2471166	40,89524007	7,077782277	0,21084242	0,00365817	integrator complex subunit	INTS14
ENSG00000110107	136,7611635	117,8113622	8,664944889	0,211555033	0,012917694	pre-mRNA processing factor	PRPF19
ENSG00000090273	184,6796832	158,9253816	8,983355008	0,211679829	0,013613674	nuclear distribution C, dynei	NUDC
ENSG00000167182	31,05080111	26,7882782	6,46801974	0,212488208	0,020177339	Sp2 transcription factor	SP2
ENSG00000106290	159,0878608	136,2965076	8,910240111	0,213769033	0,000370002	TATA-box binding protein as	TAF6
ENSG00000149136	288,1726747	248,0048416	9,591191545	0,21414989	0,003714396	structure specific recognitio	SSRP1
ENSG00000181192	65,31365898	55,6935263	7,533338035	0,214365103	0,009031165	dehydrogenase E1 and trans	DHTKD1
ENSG00000069248	66,24627881	56,86175766	7,647316609	0,215369454	0,005416784	nucleoporin 133	NUP133
ENSG00000137161	120,8921196	104,3979537	8,428763136	0,215601597	0,007151911	canopy FGF signaling regulat	CNPY3
ENSG00000116560	335,0120449	287,8841164	9,764175096	0,216045742	0,005623149	splicing factor proline and gl	SFPQ
ENSG00000022976	15,3251331	13,20813476	5,538680933	0,216251987	0,03259842	zinc finger protein 839	ZNF839
ENSG00000127463	163,0082566	140,5656253	8,999409506	0,216267203	0,002432714	ER membrane protein comp	EMC1
ENSG00000096746	220,7685888	189,8680979	9,256079511	0,216509194	0,001810747	heterogeneous nuclear ribo	HNRNPH3
ENSG00000113460	34,63278015	29,70041982	6,489568257	0,218216726	0,019734534	biogenesis of ribosomes BR)	BRIX1
ENSG00000112640	117,9120663	101,3332152	8,394999403	0,218513976	0,004556058	protein phosphatase 2 regul	PPP2R5D
ENSG00000205581	224,8739007	193,479244	9,367786403	0,218861911	0,000275875	high mobility group nucleos	HMGN1
ENSG00000106524	30,97618763	26,63171426	6,560018228	0,219755537	0,028849403	ankyrin repeat and MYND d	ANKMY2
ENSG00000069275	421,0019794	361,5681793	10,28263992	0,220380527	0,001196119	nuclear casein kinase and cy	NUCKS1
ENSG00000144848	55,10191631	47,17544679	7,368899421	0,220676582	0,005788091	autophagy related 3	ATG3
ENSG00000066557	28,91554145	24,76393293	6,361101538	0,221106456	0,01557049	leucine rich repeat containir	LRRC40
ENSG00000112081	184,9845386	158,5118345	8,92936942	0,221341737	0,005889068	serine and arginine rich splic	SRSF3
ENSG00000133114	30,51012735	26,20406883	6,548553962	0,22142744	0,003150468	GPALPP motifs containing 1	GPALPP1
ENSG00000006712	104,126001	89,15570635	8,305260427	0,221819103	0,001904176	PAF1 homolog, Paf1/RNA pc	PAF1
ENSG00000158691	16,64368814	14,28077633	5,621447269	0,221844901	0,003790749	zinc finger and SCAN domair	ZSCAN12
ENSG00000185624	1205,422555	1035,528973	11,81283595	0,22213383	0,016947292	prolyl 4-hydroxylase subunit	P4HB
ENSG00000009954	261,5230075	224,3983533	9,458331046	0,222135884	0,000213956	bromodomain adjacent to zi	BAZ1B
ENSG00000060688	52,68821615	45,24148977	7,159185399	0,223136789	0,01182468	small nuclear ribonucleopro	SNRNP40
ENSG00000182512	41,78375615	35,7600721	6,918289684	0,223180738	0,003047277	glutaredoxin 5	GLRX5
ENSG00000168488	277,3502301	237,2908177	9,634293678	0,223306367	0,015535376	ataxin 2 like	ATXN2L
ENSG00000139496	99,77516327	85,39715079	8,070310962	0,223312612	0,006416797	nucleoporin 58	NUP58

ENSG00000168944	40,99952548	34,93749368	6,941972962	0,223358989	0,049056846	centrosomal protein 120	CEP120
ENSG00000176142	28,54963928	24,48402281	6,484642313	0,223974375	0,028358917	transmembrane protein 39A	TMEM39A
ENSG00000124789	127,5034099	108,9734918	8,475324541	0,224065516	0,00146565	nucleoporin 153	NUP153
ENSG00000150787	17,23660762	14,73496414	5,643056426	0,224722287	0,038954669	6-pyruvoyltetrahydropterin	PTS
ENSG00000156531	66,72612349	57,17010521	7,581397085	0,225280877	6,61E-06	PHD finger protein 6	PHF6
ENSG00000196428	94,839036	81,19964266	8,100150729	0,225594347	0,04002838	TSC22 domain family memb	TSC22D2
ENSG00000182165	17,86182854	15,31342231	5,796306019	0,225643844	0,031698491	TP53 target 1	TP53TG1
ENSG00000132313	25,67223503	21,9120154	6,404316446	0,226065187	0,021272615	mitochondrial ribosomal pro	MRPL35
ENSG00000047621	32,68088793	27,8465479	6,645332488	0,226118921	0,033405646	chromosome 12 open readi	C12orf4
ENSG00000197056	23,41640602	19,86693703	6,329036298	0,226167763	0,018069282	zinc finger MYM-type contai	ZMYM1
ENSG00000125703	19,67747475	16,69635014	5,71564523	0,22632226	0,010349436	autophagy related 4C cystei	ATG4C
ENSG00000126883	116,5835395	99,66435985	8,465799478	0,226523674	0,009687026	nucleoporin 214	NUP214
ENSG00000136240	189,7785525	162,6567806	9,111524717	0,227238555	0,040153975	KDEL endoplasmic reticulum	KDELR2
ENSG00000092094	25,06939187	21,43154337	6,430061518	0,227725422	0,041963908	O-sialoglycoprotein endope	OSGEP
ENSG00000137100	33,85367537	28,92744199	6,742145394	0,227858138	0,010804101	dynactin subunit 3	DCTN3
ENSG00000106628	130,4858266	111,6934795	8,531327823	0,227863896	0,026619649	DNA polymerase delta 2, ac	POLD2
ENSG00000115875	91,47336284	77,87974906	7,905264353	0,228184892	0,010931789	serine and arginine rich splic	SRSF7
ENSG00000066044	131,8060373	112,5130472	8,511266046	0,228618717	0,014224098	ELAV like RNA binding prote	ELAVL1
ENSG00000099308	49,54475552	42,0144418	7,280958822	0,228670923	0,043038812	microtubule associated serin	MAST3
ENSG00000139687	196,1454799	167,2008172	9,119143352	0,22907136	1,49E-05	RB transcriptional corepress	RB1
ENSG00000105321	46,8770422	40,00513126	7,026261026	0,229499118	0,026992499	coiled-coil domain containin	CCDC9
ENSG00000166503	88,54570694	75,54891354	7,892984102	0,229710651	0,022253614	HDGF like 3	HDGFL3
ENSG00000083290	52,03169866	44,43801966	7,046845365	0,229812195	0,024165426	unc-51 like autophagy activa	ULK2
ENSG00000135185	25,14446823	21,47171618	6,071396651	0,230298542	0,007407596	transmembrane protein 243	TMEM243
ENSG00000009844	65,11405855	55,4510955	7,635987966	0,230441304	0,024539061	vesicle trafficking 1	VTA1
ENSG00000049759	96,41659659	82,29685564	8,145286516	0,230670055	0,01300699	NEDD4 like E3 ubiquitin prot	NEDD4L
ENSG00000112658	94,12117326	80,42379778	8,124588642	0,23071449	0,006087776	serum response factor	SRF
ENSG00000166452	38,20788597	32,62722493	6,583559612	0,230953739	0,032690993	A-kinase interacting protein	AKIP1
ENSG00000100997	107,7655774	91,94871234	8,117700266	0,231218278	0,002104607	abhydrolase domain contair	ABHD12
ENSG00000112514	105,6070569	90,12698383	8,39134904	0,231604181	0,005376694	cutA divalent cation toleran	CUTA
ENSG00000086200	33,58105979	28,30103842	6,614368746	0,232650032	0,032690993	importin 11	IPO11
ENSG00000124104	32,25578441	27,50081967	6,748192933	0,232847272	0,000865057	sorting nexin family membe	SNX21
ENSG00000178096	12,31298627	10,49910632	5,14396403	0,233312225	0,042603684	bolA family member 1	BOLA1
ENSG00000118412	81,35723033	69,14036022	7,647321689	0,233319202	0,032210114	caspase 8 associated protein	CASP8AP2
ENSG00000141543	113,502451	96,43333886	8,381654321	0,233340426	0,005082167	eukaryotic translation initiat	EIF4A3
ENSG00000115365	112,5326928	95,08430781	8,378222484	0,233357141	0,014721637	LanC like 1	LANCL1
ENSG00000242732	22,53770467	18,87535463	6,070148279	0,233479643	0,024918513	retrotransposon Gag like 5	RTL5
ENSG00000066697	41,08354623	35,02253678	6,900005595	0,233611659	0,013700852	Myb/SANT DNA binding dom	MSANTD3
ENSG00000100258	247,5819334	209,3660484	9,378915883	0,233849563	0,011279006	lipase maturation factor 2	LMF2
ENSG00000008405	30,30426046	25,67146164	6,501444182	0,234457677	0,025657124	cryptochrome circadian regu	CRY1
ENSG00000143390	90,76188077	77,25496118	8,001732189	0,234487891	0,030327958	regulatory factor X5	RFX5
ENSG00000115561	59,5275628	50,5964718	7,533742538	0,234662188	0,001306045	charged multivesicular body	CHMP3
ENSG00000132664	13,77557615	11,65798599	5,318130566	0,235255522	0,042864586	RNA polymerase III subunit I	POLR3F
ENSG00000149357	100,9749575	85,72593688	8,328557084	0,235362188	0,016360561	late endosomal/lysosomal a	LAMTOR1
ENSG00000109501	90,22044934	76,67948358	8,200231096	0,236117851	0,009686155	wolframin ER transmembran	WFS1
ENSG00000205726	85,30880755	72,24884927	7,990348522	0,23658615	0,000870334	intersectin 1	ITSN1
ENSG00000030066	87,14763425	74,09917589	7,82002347	0,236999695	0,000865521	nucleoporin 160	NUP160
ENSG00000131778	43,02443581	36,48023454	6,801037841	0,237589929	0,017282737	chromodomain helicase DNA	CHD1L
ENSG00000139624	65,15149865	55,23979387	7,472735681	0,238013493	0,000876666	ceramide synthase 5	CERS5
ENSG00000196961	284,467177	241,4064786	9,719922445	0,23886192	0,006565723	adaptor related protein com	AP2A1
ENSG00000053371	105,7525781	89,51612889	8,248084478	0,239299666	0,043139291	aldo-keto reductase family 7	AKR7A2
ENSG00000125107	374,2044079	316,6557649	10,01684245	0,239869527	0,005244987	CCR4-NOT transcription com	CNOT1
ENSG00000262879	16,24445468	13,77547193	5,570190727	0,239987963	0,026752114	uncharacterized LOC101927	LOC101927060
ENSG00000196507	50,56262186	42,71869127	7,191975828	0,240086296	0,025430034	transcription elongation fact	TCEAL3
ENSG00000115827	30,8069647	25,97841386	6,466467828	0,240765157	0,028412939	DDB1 and CUL4 associated f	DCAF17
ENSG00000130347	7,711447816	6,480052575	4,492962269	0,240942064	0,035039625	reticulon 4 interacting prote	RTN4IP1
ENSG00000121064	46,93966692	39,79063936	7,216263522	0,240962887	0,014752738	serine carboxypeptidase 1	SCPEP1
ENSG00000183386	45,12779499	38,28709089	7,068051036	0,241750056	0,015837595	four and a half LIM domains	FHL3
ENSG00000163655	137,2191874	116,2314115	8,397314389	0,242058374	0,004835234	guanine monophosphate syn	GMPS
ENSG00000155368	96,14089371	81,29277189	8,067498501	0,242164198	0,043797278	diazepam binding inhibitor,	DBI
ENSG00000111785	31,31279856	26,49420286	6,547514559	0,242964896	0,000285045	RIC8 guanine nucleotide exc	RIC8B
ENSG00000111843	95,13019049	80,46182874	8,195238537	0,24321021	0,004589323	transmembrane protein 14C	TMEM14C
ENSG00000100263	28,01396525	23,68963788	6,396978598	0,243623228	0,008948632	rhomboid domain containin	RHBDD3
ENSG00000135336	41,49152677	34,83453654	6,976309101	0,244108151	0,0028908	origin recognition complex s	ORC3
ENSG00000148308	79,50445168	67,25006278	7,78822371	0,244146571	0,000630908	general transcription factor	GTF3C5
ENSG00000172037	623,5148632	527,4019881	11,07289033	0,244937027	0,048428331	laminin subunit beta 2	LAMB2
ENSG00000119777	96,62579028	81,65894559	8,228843499	0,245649041	0,032392695	transmembrane protein 214	TMEM214
ENSG00000175573	33,91085976	28,66582246	6,758039258	0,246571919	0,041560864	chromosome 11 open readi	C11orf68
ENSG00000162139	28,37541623	23,46654715	6,374543386	0,247569013	0,046485083	neuraminidase 3	NEU3
ENSG00000196141	144,9170666	122,4028241	8,598105082	0,247573873	0,014228004	spermatogenesis associated	SPATS2L
ENSG00000151233	82,89674473	69,25838761	7,833935545	0,247681327	0,022024638	glucoside xylosyltransferase	GXYLT1
ENSG00000106144	89,62310902	75,67354696	7,89905635	0,247765077	0,031310288	caspase 2	CASP2
ENSG00000175105	44,21821865	37,34573585	6,87954749	0,247904445	0,02229271	zinc finger protein 654	ZNF654
ENSG00000109670	53,78844868	45,21741376	7,29302664	0,248055326	0,017631019	F-box and WD repeat domai	FBXW7
ENSG00000019144	225,4799279	190,2339089	9,340007938	0,248191468	0,046808962	pleckstrin homology like dor	PHLDB1
ENSG00000143179	98,13007875	82,66930928	7,975742819	0,248336535	0,033612341	uridine-cytidine kinase 2	UCK2
ENSG00000134262	17,86279564	15,0629857	5,767265081	0,248381532	0,017860638	adaptor related protein com	AP4B1
ENSG00000147044	110,7306848	93,19635559	8,64909192	0,24842217	0,04952883	calcium/calmodulin depend	CASK
ENSG00000162458	196,8750279	166,2941665	9,198137771	0,248462202	0,025834965	filamin binding LIM protein 1	FBLIM1
ENSG00000162909	493,2691191	416,926622	10,5941674	0,249107278	0,011524233	calpain 2	CAPN2
ENSG00000089159	273,7473145	230,5933098	9,653068097	0,25021103	0,000404904	paxillin	PXN
ENSG00000171469	41,51728387	34,91966785	7,033226944	0,250756337	0,041560864	zinc finger protein 561	ZNF561
ENSG00000120253	64,50266341	53,91261034	7,546608274	0,251005558	0,005480968	nucleoporin 43	NUP43
ENSG00000113734	7,692096615	6,446400219	4,568880268	0,251252638	0,040015488	BCL2 interacting protein 1	BNIP1
ENSG00000167384	12,02806083	10,11704641	5,130281751	0,251310294	0,039540933	zinc finger protein 180	ZNF180
ENSG00000173905	145,0296227	122,0396587	8,701449847	0,251630393	0,004767324	golgi integral membrane pro	GOLIM4
ENSG00000135093	17,62385072	14,81500452	5,896909121	0,251770467	0,003976462	ubiquitin specific peptidase	USP30
ENSG00000142279	48,66203568	41,03164441	7,050418544	0,25248926	0,032573025	WT1 interacting protein	WTIP
ENSG00000132002	188,2556142	158,3222497	9,000135711	0,252645221	0,005983829	DnaJ heat shock protein fam	DNAJB1
ENSG00000270647	269,9586643	226,938846	9,539396651	0,252866144	0,000533183	TATA-box binding protein as	TAF15
ENSG00000128951	109,9837541	92,26468481	8,315408027	0,252888725	0,017593537	deoxyuridine triphosphatase	DUT
ENSG00000157036	14,02701824	11,78063133	5,285535709	0,252893588	0,023324274	exo/endonuclease G	EXOG
ENSG00000124074	26,64264915	22,39749391	6,241954738	0,253506922	0,011824485	enkurin domain containing 1	ENKD1
ENSG00000100578	25,23756146	21,10138914	6,052370752	0,253615699	0,04877785	KIAA0586	KIAA0586
ENSG00000070010	55,66458508	46,67070398	7,326024363	0,25370933	0,016347497	ubiquitin recognition factor	UFD1
ENSG00000180900	399,2493823	332,668942	10,14172895	0,253997677	0,048295631	scribble planar cell polarity p	SCRIB
ENSG00000141577	73,61074523	61,22440769	7,573407617	0,254072802	0,007515897	centrosomal protein 131	CEP131
ENSG00000072062	226,2379082	190,1867308	9,26744772	0,254865406	0,000793334	protein kinase cAMP-activat	PRKACA
ENSG00000175984	5,702111372	4,780030231	4,069483067	0,254872775	0,041419883	DENN domain containing 2C	DENND2C
ENSG00000108829	189,2414639	159,2650067	9,059734135	0,255079979	0,037702221	leucine rich repeat containin	LRRC59
ENSG00000214223	19,54845609	16,43618782	5,777301664	0,255160756	0,015187938	heterogeneous nuclear ribo	HNRNPA1P10
ENSG00000178229	8,094594103	6,791065279	4,556169156	0,255327852	0,020886678	zinc finger protein 543	ZNF543
ENSG00000151929	163,727048	137,4448892	8,887289247	0,255706426	0,001447363	BAG cochaperone 3	BAG3
ENSG00000124370	4,653759208	3,893996834	3,801209607	0,256338398	0,043632528	methylmalonyl-CoA epimera	MCEE
ENSG00000187792	16,80537767	14,08475156	5,832133597	0,25641047	0,020345228	zinc finger protein 70	ZNF70
ENSG00000130305	29,91220208	24,650887	6,407578923	0,257152218	0,040173695	NOP2/Sun RNA methyltrans	NSUN5
ENSG00000180921	156,0776526	129,7562433	8,686359033	0,257514685	0,002084412	family with sequence similar	FAM83H
ENSG00000272602	10,38055951	8,673296065	4,984936903	0,257581969	0,004835497	zinc finger protein 595	ZNF595
ENSG00000133226	149,7270853	124,9774149	8,724180172	0,257926064	0,00993402	serine and arginine repetitiv	SRRM1
ENSG00000176340	50,70314194	42,16949864	7,215207471	0,258149974	0,006019887	cytochrome c oxidase subur	COX8A
ENSG00000109586	97,87047963	81,20001509	8,332682265	0,258250398	0,011688416	polypeptide N-acetylgalacto	GALNT7
ENSG00000074266	32,83960931	27,41373441	6,461908751	0,258537399	0,010388629	embryonic ectoderm develo	EED
ENSG00000112304	21,96212719	18,35171753	6,125288881	0,258905773	0,005903263	acyl-CoA thioesterase 13	ACOT13
ENSG00000130244	24,29275218	20,22075166	6,135543507	0,259533871	0,039913518	family with sequence similar	FAM98C
ENSG00000189114	15,22016732	12,72634549	5,649223998	0,259563532	0,033317992	biogenesis of lysosomal orga	BLOC1S3

ENSG00000165410	40,95646499	34,22478765	7,068226103	0,260177476	0,019528216	cofilin 2	CFL2
ENSG00000134779	152,8007784	127,584472	8,807730127	0,260187588	0,017123608	tubulin polyglutamylase con	TPGS2
ENSG00000141985	233,5487515	195,5718582	9,30132249	0,260193852	0,007082365	SH3 domain containing GRB	SH3GL1
ENSG00000111727	22,6777952	18,96101207	6,09904086	0,260413476	0,008972898	host cell factor C2	HCFC2
ENSG00000131374	143,042084	119,8215389	8,680317406	0,260430864	0,0149713	TBC1 domain family membe	TBC1D5
ENSG00000164985	231,4577369	192,8322004	9,213973718	0,260652223	0,026687049	PC4 and SFRS1 interacting p	PSIP1
ENSG00000197451	240,6696186	201,4733117	9,25327015	0,260744825	0,006233663	heterogeneous nuclear ribo	HNRNPAB
ENSG00000116809	51,40667491	42,98123804	7,265599457	0,260828086	0,046294665	zinc finger and BTB domain	ZBTB17
ENSG00000135899	35,75992103	29,82740279	6,806292213	0,261161487	0,024046607	SP110 nuclear body protein	SP110
ENSG00000137478	59,7169738	49,91222631	7,482847898	0,261317403	0,01380523	FCH and double SH3 domair	FCHSD2
ENSG00000100916	22,60637431	18,83877266	5,943819422	0,261850755	0,000614296	BRMS1 like transcriptional r	BRMS1L
ENSG00000074621	24,86668023	20,72923291	6,373154145	0,262685554	0,018077049	solute carrier family 24 men	SLC24A1
ENSG00000134758	30,00159772	25,04994564	6,234802095	0,262823697	0,008228378	ring finger protein 138	RNF138
ENSG00000171970	5,869162833	4,823507679	4,021533038	0,263072013	0,0347883	zinc finger protein 57	ZNF57
ENSG00000184220	25,16266614	20,48604549	6,220381153	0,263255428	0,045220168	cms1 ribosomal small subun	CMSS1
ENSG00000121851	11,98651941	10,00246926	5,227953595	0,263396637	0,029451701	RNA polymerase III subunit	POLR3GL
ENSG00000127527	50,95322865	42,63435205	7,354517921	0,263760344	0,049513879	epidermal growth factor rec	EPS15L1
ENSG00000079432	394,055635	328,527659	10,1530503	0,265029485	0,001108386	capicua transcriptional repr	CIC
ENSG00000198604	85,80488377	71,54467798	7,704115794	0,265793574	0,000302706	bromodomain adjacent to zi	BAZ1A
ENSG00000167088	47,57035608	39,55937956	6,951520769	0,266028217	0,003852301	small nuclear ribonucleopro	SNRPD1
ENSG00000113621	44,59831621	37,14629598	7,052997731	0,266711284	0,037726215	thioredoxin domain contain	TXNDC15
ENSG00000196150	18,34613478	15,28372291	5,878942357	0,26678155	0,007054064	zinc finger protein 250	ZNF250
ENSG00000087263	66,39662062	54,82882367	7,494412719	0,267100081	0,008500773	2-oxoglutarate and iron dep	OGFOD1
ENSG00000114850	165,980148	138,2742295	9,018312671	0,267176353	0,027010117	signal sequence receptor su	SSR3
ENSG00000184983	52,16584613	43,36929074	7,309137751	0,267233412	0,00043152	NADH:ubiquinone oxidored	NDUFA6
ENSG00000138674	275,4168163	229,3191963	9,73866493	0,26730885	1,56E-07	SEC31 homolog A, COPII coa	SEC31A
ENSG00000140406	75,13559281	62,52183104	7,62502058	0,267406118	0,037888806	talin rod domain containing	TLNRD1
ENSG00000083093	41,97128315	34,5182463	6,856061551	0,267739072	0,001574236	partner and localizer of BRC	PALB2
ENSG00000092201	323,052315	268,0249571	9,755051905	0,268148701	0,000113958	SPT16 homolog, facilitates c	SUPT16H
ENSG00000144040	62,66083834	51,90630872	7,495836852	0,268544242	0,005458684	sideroflexin 5	SFXN5
ENSG00000144034	17,01999091	14,08465064	5,734193692	0,268561012	0,01809203	TP53RK binding protein	TPRKB
ENSG00000132763	12,60294528	10,43911421	5,44523875	0,268610127	0,030080899	metabolism of cobalamin as	MMACHC
ENSG00000064933	36,4217624	30,15567637	6,552294267	0,27099267	0,003409264	PMS1 homolog 1, mismatch	PMS1
ENSG00000116871	429,7489591	357,03933	10,23522152	0,271005724	0,003027181	MAP7 domain containing 1	MAP7D1
ENSG00000128228	32,44020577	26,79405726	6,49412767	0,271103782	0,048245185	stromal cell derived factor 2	SDF2L1
ENSG00000182504	28,05396008	23,19786227	6,192088831	0,271106655	0,035849796	centrosomal protein 97	CEP97
ENSG00000168116	18,77880426	15,59096775	5,719506934	0,271174306	0,004652574	KIAA1586	KIAA1586
ENSG00000136717	250,9439901	207,3594974	9,826516963	0,271237302	0,010552963	bridging integrator 1	BIN1
ENSG00000137054	35,00301246	28,98785677	6,787369498	0,271239834	0,00760079	RNA polymerase I subunit E	POLR1E
ENSG00000079482	36,2811935	30,1072756	6,648408401	0,271815562	0,005280949	oligophrenin 1	OPHN1
ENSG00000108826	29,69779276	24,58544795	6,51135126	0,272186981	0,00273018	mitochondrial ribosomal pro	MRPL27
ENSG00000116120	44,81348674	36,9502671	6,97555484	0,273533839	0,013162042	phenylalanyl-tRNA syntheta	FARSB
ENSG00000189067	202,609508	166,047444	9,206304449	0,274237535	0,001546907	lipopolysaccharide induced	LITAF
ENSG00000104129	16,68603493	13,81669347	5,626238293	0,274554955	0,034267845	DnaJ heat shock protein fam	DNAJC17
ENSG00000175334	101,6291466	84,0442021	8,125620592	0,275032602	0,00578139	BAF nuclear assembly factor	BANF1
ENSG00000184939	67,3050609	55,48850045	7,614185763	0,275179958	0,000292219	ZFP90 zinc finger protein	ZFP90
ENSG00000108468	154,2122812	127,6849111	8,785731077	0,275192081	0,001401444	chromobox 1	CBX1
ENSG00000160789	632,5600784	521,869922	10,77955569	0,275624182	0,008125307	lamin A/C	LMNA
ENSG00000144792	7,526027599	6,222865244	4,588354042	0,276469152	0,034933127	zinc finger protein 660	ZNF660
ENSG00000123352	79,10412354	65,42050674	7,77066729	0,276861861	0,026202103	spermatogenesis associated	SPATS2
ENSG00000188827	41,82210348	34,25265865	6,881185573	0,277076862	0,015087786	SLX4 structure-specific endo	SLX4
ENSG00000189091	277,0814261	228,1819883	9,586818608	0,2771544	0,001677713	splicing factor 3b subunit 3	SF3B3
ENSG00000105968	155,100163	127,7605212	8,592965938	0,277697778	0,041685481	H2A.Z variant histone 2	H2AZ2
ENSG00000159445	14,01269169	11,532906	5,373644491	0,278244681	0,045213676	thioesterase superfamily me	THEM4
ENSG00000181896	13,28596233	10,91029742	5,154363091	0,278272025	0,010677887	zinc finger protein 101	ZNF101
ENSG00000164347	48,55506877	39,99123936	7,182543085	0,27836425	0,030750231	GTP dependent ribosome re	GFM2
ENSG00000102119	68,97550715	56,79781894	7,668062209	0,278493889	0,003807127	emerin	EMD
ENSG00000123609	30,8600843	25,36176389	6,441618802	0,278786807	6,68E-06	N-myc and STAT interactor	NMI
ENSG00000183431	124,093989	101,9772458	8,46189389	0,278841547	6,59E-06	splicing factor 3a subunit 3	SF3A3
ENSG00000198951	73,70449459	60,26741759	7,784848715	0,279081736	0,003295773	alpha-N-acetylgalactosamini	NAGA
ENSG00000166997	34,756174	28,69406445	6,816424044	0,279202968	0,007010271	canopy FGF signaling regula	CNPY4
ENSG00000128245	181,8450998	149,5038103	8,874529544	0,279324994	0,048868149	tyrosine 3-monooxygenase/	YWHAH
ENSG00000136450	186,5532941	153,5367619	8,973696173	0,279527067	0,006865813	serine and arginine rich splic	SRSF1
ENSG00000122299	75,50282286	62,15292919	7,649514598	0,279976041	0,005548328	zinc finger CCCH-type conta	ZC3H7A
ENSG00000181220	49,555536	40,74904868	7,185903279	0,279977098	0,00696149	zinc finger protein 746	ZNF746
ENSG00000156374	11,10850012	9,193361549	4,930811231	0,280053442	0,027139191	polycomb group ring finger	PCGF6
ENSG00000119685	66,0156449	54,06293525	7,481957558	0,281411471	0,003594488	tubulin tyrosine ligase like 5	TTLL5
ENSG00000075624	2479,922784	2047,435889	12,80782554	0,2815063	0,000548477	actin beta	ACTB
ENSG00000156831	14,37705888	11,81232598	5,418296123	0,282044619	0,043138842	NSE2 (MMS21) homolog, SM	NSMCE2
ENSG00000127191	56,66206621	46,61497644	7,249764817	0,282398238	0,000647244	TNF receptor associated fact	TRAF2
ENSG00000121542	19,06091483	15,65906304	5,806414889	0,282616313	0,003426976	SEC22 homolog A, vesicle tra	SEC22A
ENSG00000075188	19,11212527	15,59033284	5,769100393	0,28315434	0,019484295	nucleoporin 37	NUP37
ENSG00000005022	293,243731	237,3299397	9,702320928	0,283491694	0,000326704	solute carrier family 25 men	SLC25A5
ENSG00000169972	18,23908106	15,01130745	5,665761637	0,284118098	0,029785859	pseudouridine synthase like	PUSL1
ENSG00000147874	65,40137646	53,3662814	7,516293744	0,28422868	0,029250373	HAUS augmin like complex s	HAUS6
ENSG00000142230	184,8321726	151,1513996	8,93637699	0,284401383	0,036806339	SUMO1 activating enzyme s	SAE1
ENSG00000164237	115,7574869	94,84660223	8,392224271	0,284622231	0,026841504	carboxymethylenebutenolid	CMBL
ENSG00000129680	32,89751604	26,8925533	6,730508431	0,284669103	0,017072398	MAP7 domain containing 3	MAP7D3
ENSG00000174842	8,142350031	6,682160701	4,454307725	0,284790075	0,01943712	glomulin, FKBP associated p	GLMN
ENSG00000162769	17,53523181	14,35976471	5,684576648	0,2849306	0,007251945	FLVCR heme transporter 1	FLVCR1
ENSG00000172992	76,01073807	62,14749113	7,652010938	0,285391624	0,010639708	dephospho-CoA kinase dom	DCAKD
ENSG00000099783	261,1039759	214,4052425	9,431324692	0,28546004	2,32E-06	heterogeneous nuclear ribo	HNRNPM
ENSG00000135469	13,60578401	11,10011638	5,178519641	0,285544844	0,009122816	coenzyme Q10A	COQ10A
ENSG00000138617	15,35482038	12,56050313	5,401008438	0,286057778	0,041350246	poly(ADP-ribose) polymeras	PARP16
ENSG00000158186	50,51587756	41,61263976	7,425680946	0,286440159	0,011104984	muscle RAS oncogene homo	MRAS
ENSG00000149269	71,18960468	58,42479068	7,648903663	0,286697403	0,000977943	p21 (RAC1) activated kinase	PAK1
ENSG00000165996	18,70435236	15,24173461	5,799079813	0,287886004	4,04E-05	3-hydroxyacyl-CoA dehydrat	HACD1
ENSG00000175416	71,14493442	58,39598383	7,78805487	0,288031853	0,011444849	clathrin light chain B	CLTB
ENSG00000117632	261,9884165	214,7128396	9,346955173	0,288040261	0,037762215	stathmin 1	STMN1
ENSG00000124608	58,2866083	47,58172947	7,672211201	0,288081755	0,007412337	alanyl-tRNA synthetase 2, m	AARS2
ENSG00000167397	32,96224402	26,98545181	6,714733141	0,290010009	0,002075434	vitamin K epoxide reductase	VKORC1
ENSG00000147274	185,0582452	151,3164017	9,025475546	0,290329606	1,39E-06	RNA binding motif protein X	RBMX
ENSG00000143748	33,80439476	27,56832365	6,618748504	0,290374917	0,000320171	nuclear VCP like	NVL
ENSG00000160014	515,2027501	422,0813716	10,44426308	0,290708075	0,000118306	calmodulin 3	CALM3
ENSG00000185420	19,49165312	15,90457938	5,837939059	0,291482609	0,004092143	SET and MYND domain cont	SMYD3
ENSG00000203993	18,45819336	15,11470297	5,877826413	0,292489101	0,046938459	ARRDC1 antisense RNA 1	ARRDC1-AS1
ENSG00000166974	49,74388437	40,56464492	7,187558431	0,29321729	0,011528677	microtubule associated prot	MAPRE2
ENSG00000164403	45,38804389	37,02440846	6,86567879	0,293477647	0,017626368	shroom family member 1	SHROOM1
ENSG00000184990	54,07602708	44,0785823	7,069710779	0,293615445	0,02913527	SIVA1 apoptosis inducing fa	SIVA1
ENSG00000112561	29,67395952	24,16308967	6,281085422	0,293742715	0,019448309	transcription factor EB	TFEB
ENSG00000197724	88,58766923	72,47446767	7,919481066	0,293806134	0,003476859	PHD finger protein 2	PHF2
ENSG00000091436	133,4597248	108,6551177	8,595528737	0,293915891	0,018716242	mitogen-activated protein k	MAP3K20
ENSG00000105202	86,77617509	70,76390671	7,866661446	0,294101223	0,000769944	fibrillarin	FBL
ENSG00000163191	241,0275911	197,4285574	9,453167585	0,294487112	0,00234607	S100 calcium binding protei	S100A11
ENSG00000119812	73,08661761	59,56503876	7,916943028	0,295312439	0,037006556	family with sequence similar	FAM98A
ENSG00000136159	35,30435722	28,49401112	6,759567394	0,295476156	0,011734595	nudix hydrolase 15	NUDT15
ENSG00000080824	1438,299992	1161,741261	11,89282487	0,295734619	0,003308393	heat shock protein 90 alpha	HSP90AA1
ENSG00000172336	41,44551213	33,63372031	6,850898169	0,296130092	0,031585895	POP7 homolog, ribonucleas	POP7
ENSG00000171606	31,26694468	25,24463364	6,605196153	0,296161709	0,002492758	zinc finger protein 274	ZNF274
ENSG00000068903	65,23033834	53,26771587	7,540350854	0,296285037	0,006652344	sirtuin 2	SIRT2
ENSG00000054148	145,3973751	118,4559462	8,756895226	0,296369771	0,010036004	phosphohistidine phosphata	PHPT1
ENSG00000158042	79,31113381	64,68178194	7,918537518	0,296568535	0,017228063	mitochondrial ribosomal pro	MRPL17
ENSG00000166619	60,21270529	48,90762525	7,551455639	0,296708041	0,00997998	BLCAP apoptosis inducing fa	BLCAP
ENSG00000177932	16,78670044	13,7390933	5,472161389	0,296848897	0,033373691	zinc finger protein 354C	ZNF354C
ENSG00000086015	159,6117069	129,8062817	8,65804487	0,296945755	0,00334651	microtubule associated serin	MAST2

ENSG00000167645	66,95671474	54,5294756	7,611955177	0,296963151	0,023300388	Yip1 interacting factor homo	YIF1B
ENSG00000083814	7,613473432	6,212452153	4,673397704	0,297673186	0,045398437	zinc finger protein 671	ZNF671
ENSG00000256229	19,23731962	15,61803295	5,959579119	0,298006865	0,02430925	zinc finger protein 486	ZNF486
ENSG00000205413	51,59326705	41,97583367	7,313857993	0,298659106	0,01943712	sterile alpha motif domain c	SAMD9
ENSG00000163697	121,1977793	98,5823597	8,583940813	0,298876172	0,032222534	amyloid beta precursor prot	APBB2
ENSG00000196535	146,5931262	119,1512285	8,866316747	0,300084893	0,010555436	myosin XVIIIA	MYO18A
ENSG00000181826	33,9595393	27,60934213	6,364828113	0,300630655	0,000559809	RELT like 1	RELL1
ENSG00000158636	39,59871526	32,08185748	6,847435557	0,30091586	0,005388095	EMSY transcriptional repres	EMSY
ENSG00000188785	10,51219503	8,547461994	5,118665798	0,300972752	0,022798198	zinc finger protein 548	ZNF548
ENSG00000133065	95,43037654	77,48672701	8,060551854	0,301170326	0,040513231	solute carrier family 41 mem	SLC41A1
ENSG00000164663	27,07861784	22,00416212	6,363663896	0,302022992	0,049618721	ubiquitin specific peptidase	USP49
ENSG00000111602	142,2057821	114,9952504	8,610760484	0,302070406	0,028638907	timeless circadian regulator	TIMELESS
ENSG00000175550	131,1612449	105,4637434	8,523876249	0,302751856	0,048245185	DR1 associated protein 1	DRAP1
ENSG00000161547	161,664015	131,096311	8,738231362	0,30292828	0,001833617	serine and arginine rich splic	SRSF2
ENSG00000136631	49,43318148	40,08755682	7,194693919	0,302992389	3,01E-08	vacuolar protein sorting 45 I	VPS45
ENSG00000105738	226,4793912	182,7437824	9,387999789	0,303012561	0,0056297	signal induced proliferation	SIPA1L3
ENSG00000102302	50,29960202	40,85884322	7,094676662	0,303080058	0,003223665	FYVE, RhoGEF and PH doma	FGD1
ENSG00000117448	87,11814334	70,68108586	8,020155978	0,303446872	0,000284065	aldo-keto reductase family 1	AKR1A1
ENSG00000087299	20,52761105	16,39176258	5,827133133	0,303480907	0,032373764	L-2-hydroxyglutarate dehydi	L2HGDH
ENSG00000236565	5,355586119	4,341775457	3,807044071	0,303992336	0,025465274	heterogeneous nuclear ribo	HNRNPA3P5
ENSG00000154642	16,74129166	13,522306	5,667584315	0,30409853	0,026227043	chromosome 21 open readi	C21orf91
ENSG00000065457	51,63369805	41,32982056	7,168133823	0,304729578	0,010659937	adenosine deaminase tRNA	ADAT1
ENSG00000213977	6,156214854	4,977980283	4,249670542	0,305380665	0,018000577	Tax1 binding protein 3	TAX1BP3
ENSG00000117394	387,5660177	314,9328811	10,07650857	0,305420871	0,001890733	solute carrier family 2 meml	SLC2A1
ENSG00000138031	168,3721333	135,4138702	8,719438486	0,305793788	0,027914226	adenylate cyclase 3	ADCY3
ENSG00000120647	17,60544502	13,91351325	5,871947314	0,306122153	0,00012942	coiled-coil domain containin	CCDC77
ENSG00000130204	101,7073104	82,01801347	8,128443099	0,306328174	0,012164659	translocase of outer mitoch	TOMM40
ENSG00000170515	157,3249726	126,098206	8,735713943	0,306519547	0,009149701	proliferation-associated 2G4	PA2G4
ENSG00000172175	43,69509158	35,42992577	7,130008523	0,306824528	0,007176863	MALT1 paracaspase	MALT1
ENSG00000156858	79,85114288	63,57695154	7,882988748	0,30712559	0,020897752	proline rich 14	PRR14
ENSG00000198176	192,7905247	156,3321129	8,879248026	0,307204616	0,035917062	transcription factor Dp-1	TFDP1
ENSG00000197956	334,6054133	270,8201381	9,892569495	0,308025083	0,000884858	S100 calcium binding protei	S100A6
ENSG00000154237	89,09130894	72,08510304	7,978882146	0,308341756	0,003078863	leucine rich repeat kinase 1	LRRK1
ENSG00000173085	22,79832826	17,88471064	6,003821925	0,3092675	0,008736346	coenzyme Q2, polyprenyltra	COQ2
ENSG00000148229	68,43768589	55,31012858	7,640553724	0,309294693	0,022171232	DNA polymerase epsilon 3, a	POLE3
ENSG00000117868	227,9272663	182,5334312	9,417478713	0,309457917	0,000856945	extended synaptotagmin 2	ESYT2
ENSG00000124641	32,51800136	26,28345046	6,703889848	0,310188861	0,001871027	mediator complex subunit 2	MED20
ENSG00000162929	33,22715229	26,75744742	6,232285119	0,31030941	0,046446462	SANT and BTB domain regul	SANBR
ENSG00000073969	45,25317414	36,5762533	7,16668987	0,310320112	1,38E-05	N-ethylmaleimide sensitive	NSF
ENSG00000183808	52,38850409	42,27417891	7,222473417	0,310321372	7,91E-05	RNA binding motif protein 1	RBM12B
ENSG00000152193	31,18528806	25,14591905	6,436317272	0,310615635	6,66E-05	ORC ubiquitin ligase 1	OBI1
ENSG00000198860	25,25247063	20,34373731	6,134311074	0,310684066	0,001340887	tRNA splicing endonuclease	TSEN15
ENSG00000125351	21,30476864	17,07554789	5,891347342	0,312083775	0,011924221	UPF3B regulator of nonsens	UPF3B
ENSG00000155858	19,61937128	15,81603773	5,975516928	0,312492534	0,008733241	LSM11, U7 small nuclear RN	LSM11
ENSG00000204536	95,30617595	76,86731398	8,326588646	0,312515716	0,010244879	coiled-coil alpha-helical rod	CCHCR1
ENSG00000095319	169,3598747	136,2006834	8,789209146	0,312794467	0,000212903	nucleoporin 188	NUP188
ENSG00000117308	103,1045943	82,00426987	8,079788039	0,313971471	0,021709226	UDP-galactose-4-epimerase	GALE
ENSG00000132017	58,85851815	46,85113729	7,451940372	0,314643468	0,000293273	DDB1 and CUL4 associated f	DCAF15
ENSG00000224578	44,72666361	36,08494853	6,948003098	0,31470298	8,26E-06	heterogeneous nuclear ribo	HNRNPA1L3
ENSG00000086475	57,59360423	46,36154534	7,23838169	0,315167884	0,000668619	selenophosphate synthetase	SEPHS1
ENSG00000104881	144,5371345	116,4499288	8,728494227	0,31561286	0,005325789	protein phosphatase 1 regul	PPP1R13L
ENSG00000119326	111,949369	90,12777216	8,187994148	0,316315653	0,000337138	catenin alpha like 1	CTNNAL1
ENSG00000128708	73,08682777	58,71704419	7,602978746	0,316513292	0,002182977	histone acetyltransferase 1	HAT1
ENSG00000159200	35,08470397	28,28788642	6,949186299	0,316989521	0,011789614	regulator of calcineurin 1	RCAN1
ENSG00000109079	184,0941535	147,5168889	9,117198617	0,31719627	0,002503312	TNF alpha induced protein 1	TNFAIP1
ENSG00000189159	119,2906214	95,8707423	8,321775651	0,317329977	0,008412727	Jupiter microtubule associat	JPT1
ENSG00000100147	10,09239613	8,100605039	4,918309162	0,31775816	0,010804101	coiled-coil domain containin	CCDC134
ENSG00000143578	19,66538189	15,84354081	5,856147162	0,318421026	0,041721932	cAMP responsive element b	CREB3L4
ENSG00000102189	110,1692769	88,42958841	8,263701124	0,318603324	5,36E-05	early endosome antigen 1	EEA1
ENSG00000075568	156,4546111	125,0054532	8,865023384	0,318908525	0,000909101	transmembrane protein 131	TMEM131
ENSG00000141367	527,8256057	419,5755645	10,76599015	0,319145593	0,012224936	clathrin heavy chain	CLTC
ENSG00000110987	51,18321475	40,95425802	7,113503716	0,319240011	0,01011861	BAF chromatin remodeling c	BCL7A
ENSG00000090061	88,6241328	70,84962276	7,919590562	0,319487478	0,006991033	cyclin K	CCNK
ENSG00000181035	18,53210269	14,90210721	5,936665614	0,319992054	0,009657669	solute carrier family 25 mem	SLC25A42
ENSG00000089094	58,49312312	46,908107	7,217691088	0,320462353	0,002396612	lysine demethylase 2B	KDM2B
ENSG00000153406	54,01076814	43,09950441	7,409947982	0,32082131	9,06E-05	NmrA like redox sensor 1	NMRAL1
ENSG00000131591	17,90574564	14,30247018	5,824695509	0,32170773	0,005903127	chromosome 1 open reading	C1orf159
ENSG00000168461	48,55852885	38,93524934	7,153362553	0,322242009	0,017734869	RAB31, member RAS oncoge	RAB31
ENSG00000143951	4,915897149	3,938965697	3,864424647	0,322484862	0,024360774	WD repeat containing plana	WDPCP
ENSG00000171793	68,24422287	54,55014764	7,377340866	0,32274309	0,011924221	CTP synthase 1	CTPS1
ENSG00000100504	178,5378695	143,2194588	9,116288471	0,32279407	5,20E-05	glycogen phosphorylase L	PYGL
ENSG00000278259	153,8767347	122,5154383	8,481712755	0,323298707	0,000963524	myosin XIX	MYO19
ENSG00000132394	35,53142583	28,25321703	6,698507891	0,323887277	0,023686609	eukaryotic elongation factor	EEFSEC
ENSG00000142634	133,4944123	107,2486211	8,496904758	0,325131626	0,028904136	EF-hand domain family mem	EFHD2
ENSG00000140395	27,20614622	21,72639964	6,356189445	0,325248775	0,000178952	WD repeat domain 61	WDR61
ENSG00000011478	20,68963398	16,33178618	6,140977482	0,325602351	0,032449993	glutaminyl-peptide cyclotrar	QPCTL
ENSG00000112699	32,73145865	26,2791296	6,544502267	0,325631095	0,033793552	GDP-mannose 4,6-dehydrat	GMDS
ENSG00000171444	104,7862322	82,24425546	7,980940877	0,325714387	0,010349436	MCC regulator of WNT signa	MCC
ENSG00000121774	249,7912222	199,4525732	9,416064243	0,325772726	3,38E-07	KH RNA binding domain con	KHDRBS1
ENSG00000213024	146,4929564	116,6842495	8,619465865	0,325800068	0,001083586	nucleoporin 62	NUP62
ENSG00000132773	18,64093235	14,77481114	5,521904286	0,32634723	0,002753338	target of EGR1, exonuclease	TOE1
ENSG00000162775	28,50088762	22,61554952	6,217947664	0,327717439	0,000135947	RNA binding motif protein 1	RBM15
ENSG00000267534	13,62507387	10,78861434	5,035538489	0,328482445	0,014192671	sphingosine-1-phosphate re	S1PR2
ENSG00000137449	29,96525251	23,70487265	6,752373309	0,328580882	0,009856756	cytoplasmic polyadenylatior	CPEB2
ENSG00000120694	228,5679287	182,3316083	9,140933	0,329274737	0,044787536	heat shock protein family H	HSPH1
ENSG00000173588	11,97024876	9,486109016	5,094738807	0,33006562	0,004376181	centrosomal protein 83	CEP83
ENSG00000185215	179,0747183	142,5617411	9,268812494	0,330081714	0,0126665	TNF alpha induced protein 2	TNFAIP2
ENSG00000196924	2817,011115	2247,399776	13,0485148	0,330308218	0,001602767	filamin A	FLNA
ENSG00000196562	414,7142728	329,6778817	10,47580596	0,330541965	0,027048857	sulfatase 2	SULF2
ENSG00000139343	28,7859674	22,88184995	6,311379289	0,330787302	0,000706637	small nuclear ribonucleopro	SNRPF
ENSG00000266338	26,85689347	21,25722916	6,509850887	0,330815575	0,00019551	NBPF member 15	NBPF15
ENSG00000168000	7,016532433	5,583065003	4,498763694	0,331964731	0,001898038	BSCL2 lipid droplet biogenes	BSCL2
ENSG00000006432	53,82407049	42,69717693	7,397806407	0,332547036	0,00066162	mitogen-activated protein k	MAP3K9
ENSG00000038210	17,48424294	13,84406472	5,549465968	0,332650534	0,006126946	phosphatidylinositol 4-kinas	PI4K2B
ENSG00000175216	208,4406336	163,9387473	9,079008676	0,332693297	0,013162042	cytoskeleton associated pro	CKAP5
ENSG00000128050	263,8371242	210,3381518	9,441203734	0,333636311	0,003723069	phosphoribosylaminoimidaz	PAICS
ENSG00000136026	303,965571	242,8116551	9,848717217	0,334177172	0,020465588	cytoskeleton associated pro	CKAP4
ENSG00000142949	565,9057507	450,0738374	10,75111333	0,334270675	0,030916458	protein tyrosine phosphatas	PTPRF
ENSG00000188811	24,56182351	19,46942076	6,140791481	0,334447897	0,000499866	NHL repeat containing 3	NHLRC3
ENSG00000131652	28,28064045	22,22015704	6,42900122	0,334710442	0,013070314	THO complex 6	THOC6
ENSG00000258920	4,268718292	3,389197765	3,736504602	0,334790753	0,033581189	FOXN3 antisense RNA 1	FOXN3-AS1
ENSG00000142733	100,7576357	80,08728393	7,848950121	0,334930254	0,000375899	mitogen-activated protein k	MAP3K6
ENSG00000100345	2807,272268	2235,558145	13,12274972	0,335084554	0,039927934	myosin heavy chain 9	MYH9
ENSG00000219607	13,03137349	10,36667243	5,066582983	0,335106958	0,045877837	protein phosphatase 1 regul	PPP1R3G
ENSG00000097046	31,82903727	25,25971192	6,132734948	0,336243852	0,005788091	cell division cycle 7	CDC7
ENSG00000186532	52,36970088	41,28051961	7,12820227	0,336450604	0,005741347	SET and MYND domain cont	SMYD4
ENSG00000156136	44,01971501	34,51352177	6,796436665	0,336557189	0,022692623	deoxycytidine kinase	DCK
ENSG00000166889	97,83737606	76,57667496	8,02819444	0,336888246	0,005951397	PAT1 homolog 1, processing	PATL1
ENSG00000115541	17,31624209	13,68083021	5,571652642	0,33711375	0,01032395	heat shock protein family E	HSPE1
ENSG00000106992	21,55137796	16,99517384	5,844332981	0,337193864	0,001589058	adenylate kinase 1	AK1
ENSG00000124795	264,110438	208,792921	9,361671103	0,337272839	0,000509385	DEK proto-oncogene	DEK
ENSG00000136715	74,29409251	58,58196189	7,606312971	0,337333525	0,000125073	Sin3A associated protein 13	SAP130
ENSG00000160392	27,4109667	21,73079708	6,207159675	0,337340212	0,005349696	chromosome 19 open readi	C19orf47
ENSG00000178685	126,2247509	98,33273553	8,661706557	0,337591557	0,011342549	poly(ADP-ribose) polymeras	PARP10
ENSG00000182628	50,06243223	39,68261135	7,071728135	0,337758977	0,003572693	spindle and kinetochore ass	SKA2

ENSG00000136861	112,2565439	88,25747529	8,367138994	0,338352816	0,010171383	CDK5 regulatory subunit ass	CDK5RAP2
ENSG00000138668	225,3540585	178,4446537	9,280580144	0,339088212	0,000756666	heterogeneous nuclear ribo	HNRNPD
ENSG00000213551	80,99037268	63,67319139	7,719419697	0,339252959	0,02102653	DnaJ heat shock protein fam	DNAJC9
ENSG00000130695	42,9291486	33,68056902	6,832903835	0,33947292	0,002846897	centrosomal protein 85	CEP85
ENSG00000167394	17,1919567	13,61620966	5,697090036	0,33959682	0,002642877	zinc finger protein 668	ZNF668
ENSG00000162104	86,00381812	67,33813635	8,068978377	0,339913343	0,012610827	adenylate cyclase 9	ADCY9
ENSG00000110811	149,071015	118,2140663	8,630507018	0,340717986	0,012804591	prolyl 3-hydroxylase 3	P3H3
ENSG00000164070	157,6383779	123,4710204	8,613721	0,340841009	0,030856092	heat shock protein family A	HSPA4L
ENSG00000126453	30,1626748	23,4329488	6,544725399	0,341927953	0,003062517	BCL2 like 12	BCL2L12
ENSG00000203778	9,389770878	7,422311006	5,014894707	0,342219468	0,025268496	family with sequence similar	FAM229B
ENSG00000137288	37,03043287	28,97339361	6,828063032	0,342312416	0,00127287	ubiquinol-cytochrome c redu	UQCC2
ENSG00000137103	55,55934673	43,92566561	7,423578121	0,342403332	0,00440735	transmembrane protein 8B	TMEM8B
ENSG00000110047	132,7890737	104,9357804	8,597246872	0,343064761	2,83E-05	EH domain containing 1	EHD1
ENSG00000183527	31,83051596	24,99122712	6,270225681	0,343425736	0,040195631	proteasome assembly chape	PSMG1
ENSG00000103942	47,20204555	37,26654162	7,07584186	0,343757	0,002205646	homer scaffold protein 2	HOMER2
ENSG00000167005	184,1976282	144,7780687	8,938351705	0,343855387	1,05E-07	nudix hydrolase 21	NUDT21
ENSG00000156110	40,95851921	32,13455048	6,781231622	0,344057777	5,57E-06	adenosine kinase	ADK
ENSG00000138468	19,75921318	15,57199052	5,975860706	0,344125907	0,010549797	SUMO specific peptidase 7	SENP7
ENSG00000147224	62,74898624	49,45458123	7,412147715	0,344144466	0,000505596	phosphoribosyl pyrophosph	PRPS1
ENSG00000234444	21,83455765	17,14535876	6,143133976	0,344881019	0,001631634	zinc finger protein 736	ZNF736
ENSG00000175548	18,14519829	14,18027618	5,651744306	0,344885231	0,017517018	ALG10 alpha-1,2-glucosyltra	ALG10B
ENSG00000059573	151,0299178	118,9258607	8,88530447	0,345189939	6,58E-07	aldehyde dehydrogenase 18	ALDH18A1
ENSG00000054282	29,21495524	22,91152223	6,207568615	0,345202413	0,00544089	SHH signaling and ciliogenes	SDCCAG8
ENSG00000148426	71,77905443	56,76071147	7,64452075	0,345774706	0,034833115	proline and serine rich 2	PROSER2
ENSG00000070371	27,52551771	21,63855174	6,442931274	0,345824659	0,029059675	clathrin heavy chain like 1	CLTCL1
ENSG00000197785	50,70108226	39,70393252	7,174998582	0,345831242	0,017626368	ATPase family AAA domain c	ATAD3A
ENSG00000131899	147,6280565	115,8806175	8,750273825	0,34636481	0,000102996	LLGL scribble cell polarity co	LLGL1
ENSG00000136425	23,26253179	18,31824317	6,112805735	0,346543704	0,021066465	calcium and integrin binding	CIB2
ENSG00000105619	29,05472231	22,65549973	6,427864178	0,346551561	0,000593725	TCF3 fusion partner	TFPT
ENSG00000104897	134,0628841	105,2874838	8,522794962	0,347140699	0,000479822	splicing factor 3a subunit 2	SF3A2
ENSG00000197467	10,72883257	8,340982797	5,35352982	0,347319497	0,022054197	collagen type XIII alpha 1 ch	COL13A1
ENSG00000179409	51,73242307	40,36343463	7,224622806	0,347775182	9,83E-06	gem nuclear organelle assoc	GEMIN4
ENSG00000275004	12,32138956	9,680726545	5,197539537	0,347806976	0,009116465	zinc finger protein 280B	ZNF280B
ENSG00000148832	12,1867974	9,494990112	5,357920212	0,347950569	0,012672176	polyamine oxidase	PAOX
ENSG00000164331	19,02548556	14,88329531	5,810705413	0,348161285	0,018982637	ankyrin repeat family A men	ANKRA2
ENSG00000124788	72,39303034	56,45349845	7,695307854	0,348284952	0,015730715	ataxin 1	ATXN1
ENSG00000107020	16,91299883	13,16047161	5,841845866	0,348668538	0,016443041	plasminogen receptor with a	PLGRKT
ENSG00000174307	219,8168498	173,1334948	9,357182502	0,348812198	0,000832137	pleckstrin homology like dor	PHLDA3
ENSG00000186073	11,40303052	8,963410334	5,053064511	0,349330185	0,017154398	CDAN1 interacting nuclease	CDIN1
ENSG00000106392	49,63609919	39,00585085	7,161126067	0,349636312	0,000409803	core 1 synthase, glycoprotei	C1GALT1
ENSG00000110104	50,07225694	38,29732906	7,472792055	0,349859872	0,001866744	coiled-coil domain containin	CCDC86
ENSG00000178935	6,211257595	4,878669549	4,246003686	0,34996804	0,031496891	zinc finger protein 552	ZNF552
ENSG00000113649	78,60287797	61,47258111	7,745256349	0,350512597	0,000776182	transcription elongation reg	TCERG1
ENSG00000261115	119,3397214	93,41276936	8,537742566	0,350684552	0,012492662	transmembrane protein 178	TMEM178B
ENSG00000079387	34,5903512	26,88553332	6,403140057	0,351056039	0,012331853	SUMO specific peptidase 1	SENP1
ENSG00000171992	173,3403934	136,5520052	9,159062349	0,351132269	0,004887213	synaptopodin	SYNPO
ENSG00000168214	134,4039957	105,3423001	8,668293282	0,351607871	0,002432836	recombination signal binding	RBPJ
ENSG00000213523	52,28377725	41,12328658	7,309850479	0,351788388	0,021902174	steroid receptor RNA activat	SRA1
ENSG00000160703	55,9344669	43,44445698	7,282788683	0,351857714	0,005996632	NLR family member X1	NLRX1
ENSG00000025770	92,24034205	71,99537261	7,861799984	0,351897491	0,008925025	non-SMC condensin II comp	NCAPH2
ENSG00000175866	60,89866256	47,30659082	7,439318746	0,351935304	0,041623145	BAR/IMD domain containing	BAIAP2
ENSG00000130332	33,80778565	26,48771255	6,67816312	0,352110506	0,008233528	LSM7 homolog, U6 small nu	LSM7
ENSG00000166004	53,4042139	41,34188071	7,117411798	0,352236364	0,000801742	centrosomal protein 295	CEP295
ENSG00000103121	20,39237071	15,88192585	5,869067589	0,352652875	0,002296435	C-X9-C motif containing 2	CMC2
ENSG00000182004	36,66315739	28,65838182	6,733938346	0,352852625	7,35E-05	small nuclear ribonucleopro	SNRPE
ENSG00000164086	59,16314689	46,53899171	7,348280623	0,352975486	0,002775747	dual specificity phosphatase	DUSP7
ENSG00000176102	52,03599218	40,74434391	7,131010762	0,353084796	1,24E-09	cleavage stimulation factor s	CSTF3
ENSG00000126216	46,4467717	36,17671569	6,865710902	0,353273381	0,000510259	tubulin gamma complex ass	TUBGCP3
ENSG00000160062	11,80019422	9,257874099	4,865071182	0,353599989	0,005958459	zinc finger and BTB domain c	ZBTB8A
ENSG00000143621	168,1009969	131,6841425	8,826076682	0,354116947	3,45E-06	interleukin enhancer binding	ILF2
ENSG00000076321	21,75342987	17,04998454	6,092494326	0,354176403	4,76E-05	kelch like family member 20	KLHL20
ENSG00000054392	46,79225144	36,48317858	7,313902687	0,354335839	0,002438732	hedgehog acyltransferase	HHAT
ENSG00000089505	15,39325812	12,05231682	5,39213268	0,354480303	0,003203133	CKLF like MARVEL transmem	CMTM1
ENSG00000122642	199,3093481	156,2009508	9,025133221	0,35493589	0,006481226	FKBP prolyl isomerase 9	FKBP9
ENSG00000075336	34,11191432	26,63602726	6,660684341	0,354941061	1,96E-05	translocase of inner mitocho	TIMM21
ENSG00000117411	78,13129425	61,25331177	7,819610871	0,355028121	0,004384877	beta-1,4-galactosyltransfera	B4GALT2
ENSG00000165916	131,01483	102,1076028	8,443831473	0,355184174	0,001617645	proteasome 26S subunit, AT	PSMC3
ENSG00000159899	66,5633481	52,12037432	7,692705715	0,35559095	0,032238371	natriuretic peptide receptor	NPR2
ENSG00000125945	38,57611254	30,33189046	6,849680844	0,356087474	0,0101059	zinc finger protein 436	ZNF436
ENSG00000049449	320,3291559	249,7921465	9,876022956	0,35647913	0,002494372	reticulocalbin 1	RCN1
ENSG00000168297	38,0004534	29,52538652	6,733852943	0,357050151	0,038910085	PX domain containing serine	PXK
ENSG00000128283	131,0919824	102,4331938	8,386417487	0,357067613	0,000108043	CDC42 effector protein 1	CDC42EP1
ENSG00000068028	25,22214365	19,67660637	6,37065999	0,357236458	0,006375267	Ras association domain fam	RASSF1
ENSG00000067057	537,5374223	419,4231734	10,63692658	0,357643827	0,000314281	phosphofructokinase, platel	PFKP
ENSG00000083544	14,30835621	11,1842482	5,464470004	0,35829535	0,001973877	tudor domain containing 3	TDRD3
ENSG00000140365	82,073042	63,5343275	7,797764552	0,358303802	0,002439351	COMM domain containing 4	COMMD4
ENSG00000078237	56,42544569	43,86224793	7,325006296	0,358611701	0,014844499	TP53 induced glycolysis regu	TIGAR
ENSG00000177302	87,72482542	67,96343536	7,75601918	0,358755503	0,001976504	DNA topoisomerase III alpha	TOP3A
ENSG00000112992	115,5049585	90,327614	8,404100865	0,35886202	2,92E-05	nicotinamide nucleotide tra	NNT
ENSG00000182263	19,3695734	15,1337994	6,187385553	0,359245015	3,56E-05	fidgetin, microtubule severi	FIGN
ENSG00000170860	51,93329731	40,07980737	7,293028879	0,360842399	0,000288806	LSM3 homolog, U6 small nu	LSM3
ENSG00000049541	87,93875031	68,65726538	7,760036971	0,360999107	0,018529162	replication factor C subunit 2	RFC2
ENSG00000111581	61,83206348	47,84779969	7,417197252	0,362955336	4,92E-08	nucleoporin 107	NUP107
ENSG00000188229	572,6385346	427,8211519	10,71570839	0,363073967	0,007318651	tubulin beta 4B class IVb	TUBB4B
ENSG00000068079	29,78976926	22,62472217	6,238707894	0,363735019	0,031849368	interferon induced protein 3	IFI35
ENSG00000054983	19,47787637	15,11348263	5,89830185	0,36385487	0,0028908	galactosylceramidase	GALC
ENSG00000165389	42,0822714	32,57965786	6,879440909	0,363895751	0,000864104	serine palmitoyltransferase	SPTSSA
ENSG00000240682	13,69487376	10,66677709	5,315439637	0,364501528	4,54E-06	ISY1 splicing factor homolog	ISY1
ENSG00000064115	238,1547303	185,0723202	9,308544548	0,364512481	0,000336483	transmembrane 7 superfam	TM7SF3
ENSG00000149782	148,0371965	113,9558485	8,550186453	0,364557746	0,000108752	phospholipase C beta 3	PLCB3
ENSG00000243156	107,8526432	83,61783979	8,188058021	0,365187954	0,000191655	microtubule associated mor	MICAL3
ENSG00000196456	10,92048795	8,483241029	5,011750635	0,365644156	0,010617796	zinc finger protein 775	ZNF775
ENSG00000108666	18,61887059	14,46178014	5,803156684	0,365656544	0,000358765	chromosome 17 open readi	C17orf75
ENSG00000058804	53,1913873	40,26741913	7,22878592	0,365683157	0,007326572	NDC1 transmembrane nucle	NDC1
ENSG00000254858	20,31636741	15,47659877	5,824759833	0,366118409	0,043873569	MPV17 mitochondrial inner	MPV17L2
ENSG00000124508	27,59349802	21,49597806	6,434556054	0,366500238	0,018480097	butyrophilin subfamily 2 me	BTN2A2
ENSG00000103540	63,97582485	49,35139281	7,221396034	0,367319678	0,001277811	centriolar coiled-coil proteir	CCP110
ENSG00000113648	325,0856756	251,9867516	9,741675595	0,367703435	2,94E-17	macroH2A.1 histone	MACROH2A1
ENSG00000117593	74,84467532	57,34459965	7,725866302	0,367730666	9,48E-07	aspartyl-tRNA synthetase 2,	DARS2
ENSG00000114790	17,1144025	13,30443726	5,39463184	0,367940007	0,023780749	Rho guanine nucleotide excl	ARHGEF26
ENSG00000179051	286,334429	222,4723823	9,641390636	0,368163274	0,000539989	regulator of chromosome co	RCC2
ENSG00000081870	30,69507894	23,74750291	6,254448124	0,368835347	0,000301755	heat shock protein family B	HSPB11
ENSG00000120868	99,37333516	77,03997988	7,896439184	0,368851175	6,31E-06	apoptotic peptidase activati	APAF1
ENSG00000124067	265,6206089	205,8506394	9,645903164	0,368918916	0,001885261	solute carrier family 12 men	SLC12A4
ENSG00000117748	67,95808164	52,79742286	7,579181572	0,368948745	0,003914841	replication protein A2	RPA2
ENSG00000185404	27,55980336	21,31088921	6,489173711	0,369091207	3,40E-05	SP140 nuclear body protein	SP140L
ENSG00000155380	138,3827962	107,4929734	8,510280585	0,369469073	0,000102436	solute carrier family 16 men	SLC16A1
ENSG00000177469	995,5495513	770,7636312	11,51412497	0,369696471	0,000704793	caveolae associated protein	CAVIN1
ENSG00000172009	118,941487	91,04643351	8,258925524	0,369769807	0,002500433	thimet oligopeptidase 1	THOP1
ENSG00000188807	70,0876128	53,80271184	7,461896581	0,370079762	0,001275578	transmembrane protein 201	TMEM201
ENSG00000118508	60,23417089	46,69809423	7,226280361	0,370428882	0,021201318	RAB32, member RAS oncoge	RAB32
ENSG00000112144	21,50209553	16,4950605	5,882685374	0,372378532	0,020421247	ciliogenesis associated kinas	CILK1
ENSG00000170558	199,0682686	153,5987531	9,263612298	0,37243591	0,006149185	cadherin 2	CDH2
ENSG00000101220	25,32943615	19,40328496	6,365879344	0,372580366	0,017626368	chromosome 20 open readi	C20orf27
ENSG00000106355	16,37954088	12,59818401	5,585866763	0,372744909	0,006409312	LSM5 homolog, U6 small nu	LSM5

ENSG00000242265	310,2113954	234,08782	9,736360801	0,373046186	0,037252957	paternally expressed 10	PEG10
ENSG00000150593	99,77081581	77,13580856	8,063675624	0,373132568	0,001987968	programmed cell death 4	PDCD4
ENSG00000035403	621,9455487	481,2983887	10,61217106	0,373935498	0,001617527	vinculin	VCL
ENSG00000179611	14,02180385	10,76416333	5,242610657	0,374654409	0,024111702	diacylglycerol kinase zeta ps	DGKZP1
ENSG00000198088	11,23039714	8,643529204	4,262099722	0,374892479	0,032649166	nucleoporin 62 C-terminal li	NUP62CL
ENSG00000153976	48,08499773	36,50996819	7,219123847	0,375047031	0,018476047	heparan sulfate-glucosamin	HS3ST3A1
ENSG00000132274	197,132573	152,1023029	9,065136784	0,375827782	1,84E-05	tripartite motif containing 2	TRIM22
ENSG00000133739	24,07130136	18,43362358	5,938239896	0,376623544	0,035546128	leucine rich repeat and coile	LRRCC1
ENSG00000127589	13,44538162	10,28229519	5,449465522	0,376668354	0,00129084	tubulin beta pseudogene 1	TUBBP1
ENSG00000172819	26,79051877	20,65213732	6,298523695	0,376817682	0,000526351	retinoic acid receptor gamm	RARG
ENSG00000109685	325,5680931	247,1833534	9,682323613	0,377058702	0,009277138	nuclear receptor binding SE	NSD2
ENSG00000168765	16,67051457	12,78874439	5,899244585	0,37711603	0,02994471	glutathione S-transferase m	GSTM4
ENSG00000164976	67,72303357	52,13385554	7,4336713	0,377685853	0,021052883	myogenesis regulating glyco	MYORG
ENSG00000120837	28,30031668	21,73468336	6,266371525	0,3779185	0,007003179	nuclear transcription factor	NFYB
ENSG00000113504	173,5034758	132,9628512	8,861220311	0,377940706	0,019484295	solute carrier family 12 men	SLC12A7
ENSG00000162777	32,96899319	24,97655504	6,673079174	0,378206395	0,012021379	DENN domain containing 2D	DENND2D
ENSG00000160917	72,64107977	55,55955879	7,607063355	0,378612972	0,041132248	cleavage and polyadenylatic	CPSF4
ENSG00000149177	333,0550064	256,7015316	10,17391442	0,37869326	0,001157642	protein tyrosine phosphatas	PTPRJ
ENSG00000067066	76,53383065	58,54547247	7,884085964	0,378938675	0,018596854	SP100 nuclear antigen	SP100
ENSG00000205476	65,73255368	50,28631152	7,347008958	0,379006166	0,00355085	coiled-coil domain containin	CCDC85C
ENSG00000101544	68,47297832	52,10329245	7,599180627	0,379068603	8,42E-05	ADNP homeobox 2	ADNP2
ENSG00000121988	13,12606928	10,04633212	5,365802143	0,379615412	9,81E-05	zinc finger RANBP2-type cor	ZRANB3
ENSG00000107672	27,31903612	20,83219005	6,225182179	0,379765469	0,000605587	NSE4 homolog A, SMC5-SM(	NSMCE4A
ENSG00000184162	36,23516567	27,58426326	6,529442496	0,380165699	0,001074588	nuclear receptor 2C2 associ	NR2C2AP
ENSG00000149091	94,38698569	72,34135983	7,932074031	0,381001232	0,004316853	diacylglycerol kinase zeta	DGKZ
ENSG00000163932	76,972529	58,01604922	7,672998019	0,381290873	0,001353639	protein kinase C delta	PRKCD
ENSG00000109062	119,5519871	91,19575593	8,215835685	0,383230325	0,002709468	SLC9A3 regulator 1	SLC9A3R1
ENSG00000148339	27,24974372	20,72590039	6,011459254	0,383426571	0,048254404	solute carrier family 25 men	SLC25A25
ENSG00000268061	2,776292246	2,138664037	3,162045631	0,383716207	0,035625276	NAPA antisense RNA 1	NAPA-AS1
ENSG00000129028	11,96609432	9,076515825	5,177814582	0,383723057	0,000207169	THAP domain containing 10	THAP10
ENSG00000158079	17,26662389	13,19727363	5,652398746	0,384792351	2,47E-05	protein tyrosine phosphatas	PTPDC1
ENSG00000012963	63,64712445	49,07312776	7,244173439	0,38491693	0,012682119	ubiquitin protein ligase E3 c	UBR7
ENSG00000083444	523,3027302	401,7020056	10,59352013	0,385673332	0,002903769	procollagen-lysine,2-oxoglut	PLOD1
ENSG00000139350	56,43706779	42,98719994	7,247075104	0,386778126	0,000491127	NEDD1 gamma-tubulin ring	NEDD1
ENSG00000163006	8,112895377	6,134346679	4,61318254	0,386815054	0,038916076	coiled-coil domain containin	CCDC138
ENSG00000143367	34,29189376	26,29370456	6,714070601	0,387056811	0,011226728	tuftelin 1	TUFT1
ENSG00000136104	29,03037646	22,13808595	6,235288527	0,387293538	0,002533386	ribonuclease H2 subunit B	RNASEH2B
ENSG00000149639	183,7431095	140,4293632	9,033042803	0,387359249	0,004688158	suppressor of glucose, autop	SOGA1
ENSG00000138640	40,64904831	31,13220454	7,06079633	0,3874297	0,017776195	family with sequence similar	FAM13A
ENSG00000184178	14,32954454	10,97375164	5,431141867	0,387662451	0,006089924	sec1 family domain containi	SCFD2
ENSG00000144935	19,38292691	14,83741745	5,661668475	0,388482867	0,001973524	transient receptor potential	TRPC1
ENSG00000138496	64,92310392	49,45790714	7,444797748	0,388588603	0,00512802	poly(ADP-ribose) polymeras	PARP9
ENSG00000124201	177,5145235	134,4443789	9,030818339	0,388796379	0,014204217	zinc finger NFX1-type contai	ZNFX1
ENSG00000158301	18,58654237	14,19982846	5,919085903	0,38882247	2,49E-06	G protein-coupled receptor	GPRASP2
ENSG00000171067	76,29646103	58,35626641	7,906229935	0,388997606	0,000682541	chromosome 11 open readi	C11orf24
ENSG00000274180	150,5633727	115,4281578	8,92809433	0,389088236	0,032744945	N-acetyltransferase domain	NATD1
ENSG00000071205	46,42608837	35,62431761	6,910652233	0,38928509	0,002449869	Rho GTPase activating prote	ARHGAP10
ENSG00000051825	45,47203109	34,19300802	6,809706044	0,389518425	0,009902161	M-phase phosphoprotein 9	MPHOSPH9
ENSG00000100321	45,78368861	35,04123554	7,211098323	0,389744022	0,032711963	synaptogyrin 1	SYNGR1
ENSG00000004975	125,576776	95,12924575	8,502852739	0,390908257	2,71E-10	dishevelled segment polarity	DVL2
ENSG00000130816	363,4154177	276,4675112	9,720379842	0,391041539	0,00235404	DNA methyltransferase 1	DNMT1
ENSG00000002549	83,60706905	62,77974434	7,883511961	0,391062128	0,041821274	leucine aminopeptidase 3	LAP3
ENSG00000145365	29,80983944	22,76620656	6,213321764	0,391480741	0,003253291	TRAF interacting protein wit	TIFA
ENSG00000189164	5,622211387	4,288418162	4,196039376	0,391526063	0,016891494	zinc finger protein 527	ZNF527
ENSG00000176894	15,96169338	12,19812204	5,33722701	0,391707417	0,040697752	peroxisomal membrane pro	PXMP2
ENSG00000155561	143,6858698	108,7499124	8,593850575	0,391713085	1,01E-05	nucleoporin 205	NUP205
ENSG00000125450	79,01842881	59,80921547	7,657544133	0,391854909	3,52E-08	nucleoporin 85	NUP85
ENSG00000118620	16,96630107	12,86797035	5,614269409	0,392230214	3,74E-06	zinc finger protein 430	ZNF430
ENSG00000136811	92,10803503	69,42264595	7,997724436	0,392805733	0,00462862	outer dense fiber of sperm t	ODF2
ENSG00000213186	19,94678919	15,09717127	5,779650779	0,393493363	0,004790234	tripartite motif containing 5	TRIM59
ENSG00000105397	221,0247457	167,1785928	9,295709655	0,393793488	0,000116515	tyrosine kinase 2	TYK2
ENSG00000005801	23,75303054	17,99927182	6,038468892	0,394036166	0,001228613	zinc finger protein 195	ZNF195
ENSG00000166710	534,9010683	403,7478442	10,61153556	0,394041385	0,008176901	beta-2-microglobulin	B2M
ENSG00000205220	12,31491607	9,378315269	5,172319984	0,394338291	7,68E-06	proteasome 20S subunit bet	PSMB10
ENSG00000168067	54,33027348	41,09702315	7,197302116	0,396909944	0,005666418	mitogen-activated protein k	MAP4K2
ENSG00000123427	5,618895844	4,274025749	4,519226563	0,397913681	0,008678882	EEF1A lysine methyltransfer	EEF1AKMT3
ENSG00000151287	16,79203817	12,47858517	5,411403759	0,398497425	0,000404904	testis expressed 30	TEX30
ENSG00000187325	20,53190962	15,60651869	5,665994834	0,398976741	0,014880976	TATA-box binding protein as	TAF9B
ENSG00000102900	77,10191588	58,26049226	7,797395738	0,399293092	2,38E-06	nucleoporin 93	NUP93
ENSG00000080839	51,30600883	38,83069758	6,947871142	0,399514431	0,015093441	RB transcriptional corepress	RBL1
ENSG00000134297	5,806002521	4,408740917	3,946881262	0,39954251	0,007423706	pleckstrin homology domair	PLEKHA8P1
ENSG00000130684	52,62721691	39,84475048	7,402307857	0,399638755	0,00997998	zinc finger protein 337	ZNF337
ENSG00000283154	14,00700745	10,66839959	5,248185925	0,400343712	0,041764698	schwannomin interacting pr	SCHIP1
ENSG00000075391	52,63128898	39,81471951	7,182307483	0,400421774	0,000241198	RAS protein activator like 2	RASAL2
ENSG00000159388	212,8799239	161,3122626	9,19228752	0,400500097	0,002766128	BTG anti-proliferation factor	BTG2
ENSG00000152223	117,7215132	86,58464215	8,505298284	0,401125801	0,002560747	ectopic P-granules 5 autoph	EPG5
ENSG00000182704	82,48629335	62,54259761	8,176106476	0,401646029	0,001478149	tsukushi, small leucine rich p	TSKU
ENSG00000102897	24,2582832	18,33657028	6,280212431	0,401981155	0,001770827	LYR motif containing 1	LYRM1
ENSG00000241058	12,20170521	9,106551191	5,246741722	0,402866216	0,029423161	NOP2/Sun RNA methyltrans	NSUN6
ENSG00000245060	8,620825485	6,525009522	4,81565247	0,403135119	0,00145233	long intergenic non-protein	LINC00847
ENSG00000026103	67,89251963	51,19011046	7,565769791	0,403499243	0,004659771	Fas cell surface death recept	FAS
ENSG00000135446	153,773785	116,5469197	8,736026908	0,403623944	0,000256191	cyclin dependent kinase 4	CDK4
ENSG00000165806	51,51848455	38,91505559	7,126751772	0,403649631	0,002472581	caspase 7	CASP7
ENSG00000156384	8,117645946	6,135107094	4,410455309	0,403684522	0,027766555	SWI5 dependent homologou	SFR1
ENSG00000242259	36,56932157	27,55494218	6,786529752	0,403774085	2,89E-07	chromosome 22 open readi	C22orf39
ENSG00000168014	73,7702197	54,69318373	7,682185416	0,403916132	0,000175753	C2 domain containing 3 cent	C2CD3
ENSG00000249459	3,073886747	2,328974908	3,306001052	0,403954994	0,029180335	zinc finger protein 286B (pse	ZNF286B
ENSG00000169515	61,86619669	46,79808526	7,31431881	0,404270995	1,45E-05	coiled-coil domain containin	CCDC8
ENSG00000182541	108,8610275	82,46472931	8,128834047	0,404643824	0,001564605	LIM domain kinase 2	LIMK2
ENSG00000149480	237,2737049	178,3449559	9,292929503	0,405621891	4,00E-05	metastasis associated 1 fam	MTA2
ENSG00000176401	5,54831739	4,150935419	4,022130001	0,406227699	0,017860221	EP300 interacting inhibitor c	EID2B
ENSG00000135314	2,997750984	2,261124283	3,029090325	0,406326891	0,028267702	KH domain containing 1	KHDC1
ENSG00000176463	53,41081534	40,39725312	6,746669921	0,407520772	0,03204866	solute carrier organic anion	SLCO3A1
ENSG00000177303	79,68924145	60,18466467	7,837991297	0,407761098	8,81E-06	CASK interacting protein 2	CASKIN2
ENSG00000143393	135,1994141	102,0478447	8,602252727	0,407968415	7,60E-06	phosphatidylinositol 4-kinas	PI4KB
ENSG00000185379	37,5494072	28,32358168	6,558216546	0,408116663	0,014854	RAD51 paralog D	RAD51D
ENSG00000171033	44,90196554	33,87279654	6,845644773	0,408635737	0,000931252	cAMP-dependent protein kii	PKIA
ENSG00000204392	39,82519295	29,96545221	6,726799539	0,408663253	0,008700476	LSM2 homolog, U6 small nu	LSM2
ENSG00000164236	57,26709886	42,87354823	7,595409148	0,40924161	0,000676566	ankyrin repeat domain 33B	ANKRD33B
ENSG00000267106	17,35969689	13,08941475	5,570039693	0,409667656	3,58E-05	ZNF561 antisense RNA 1 (he	ZNF561-AS1
ENSG00000163071	55,79509115	40,84160615	7,579206903	0,410788879	0,002527463	spermatogenesis associated	SPATA18
ENSG00000169750	20,95074799	15,5519065	6,075248852	0,411111908	0,012996405	Rac family small GTPase 3	RAC3
ENSG00000149308	57,64190569	42,85134296	7,18445234	0,411169445	4,01E-05	nuclear protein, coactivator	NPAT
ENSG00000134285	17,93339739	13,47921626	5,849795048	0,41159273	0,022940758	FKBP prolyl isomerase 11	FKBP11
ENSG00000067836	45,0935882	33,72472425	7,029206761	0,411979031	0,001847796	rogdi atypical leucine zipper	ROGDI
ENSG00000145241	18,57183572	13,77738526	5,735692246	0,412493174	1,29E-06	centromere protein C	CENPC
ENSG00000151376	23,13586383	17,37615942	5,825705122	0,412659658	0,001146672	malic enzyme 3	ME3
ENSG00000172638	188,5518317	141,8580786	9,01435972	0,412682473	0,011678952	EGF containing fibulin extra	EFEMP2
ENSG00000137656	32,17666333	24,07066887	6,373889915	0,412889432	5,78E-05	BUD13 homolog	BUD13
ENSG00000140386	22,18074513	16,68726646	6,030748001	0,41326002	5,16E-06	S-phase cyclin A associated	SCAPER
ENSG00000259583	4,379096762	3,288413114	3,865088579	0,413523313	0,020413448	ALDH1A3 antisense RNA 1	ALDH1A3-AS1
ENSG00000099901	140,9735065	105,7729913	8,467909827	0,413953228	0,000195943	RAN binding protein 1	RANBP1
ENSG00000196284	16,21507206	12,1621144	5,687600228	0,414261464	9,86E-05	SPT3 homolog, SAGA and ST	SUPT3H
ENSG00000149554	46,13027945	34,66332686	6,842581326	0,414805514	0,001393038	checkpoint kinase 1	CHEK1
ENSG00000100726	107,6876856	79,53017783	8,213259814	0,416406092	8,39E-05	telomere maintenance 2	TELO2

ENSG00000156011	108,5708371	81,31039939	8,331748175	0,416608722	0,000866381	pleckstrin and Sec7 domain	PSD3
ENSG00000135709	39,00307936	28,89231013	6,885839561	0,417107894	1,44E-05	KIAA0513	KIAA0513
ENSG00000173163	16,487735	12,32935284	5,671462741	0,417322212	2,09E-05	copper metabolism domain	COMMD1
ENSG00000120699	26,05870747	19,31894422	6,148546438	0,417331018	0,001957835	exosome component 8	EXOSC8
ENSG00000132383	264,9964697	198,3490558	9,493109428	0,417351097	0,001464383	replication protein A1	RPA1
ENSG00000196230	1505,991077	1118,732304	12,18711036	0,417609639	3,57E-06	tubulin beta class I	TUBB
ENSG00000233757	3,442164467	2,551785971	3,445104995	0,418016469	0,025613518	zinc finger protein 570-like	LOC344065
ENSG00000152454	5,639094356	4,184873025	4,03376116	0,418053711	0,009975857	zinc finger protein 256	ZNF256
ENSG00000173041	15,06298235	11,20114194	5,55287468	0,419485634	0,000142202	zinc finger protein 680	ZNF680
ENSG00000143126	272,5406798	204,6678076	9,462064246	0,419737808	0,023905886	cadherin EGF LAG seven-pas	CELSR2
ENSG00000100106	211,9524551	158,2900798	9,206209685	0,420681283	0,049780592	TRIO and F-actin binding prc	TRIOBP
ENSG00000168785	118,3412637	88,52959352	8,278693807	0,421604305	0,002071169	tetraspanin 5	TSPAN5
ENSG00000177728	104,1393219	77,84147127	8,266794026	0,422888002	3,08E-06	transmembrane protein 94	TMEM94
ENSG00000135074	64,70340563	46,99911278	7,54420867	0,423063251	0,007706027	ADAM metallopeptidase doı	ADAM19
ENSG00000124496	33,6246695	24,9792407	6,38444525	0,423220425	2,28E-05	transcriptional regulating fa	TRERF1
ENSG00000102804	240,954474	177,9620223	9,3471931	0,423519056	0,013916224	TSC22 domain family memb	TSC22D1
ENSG00000150961	87,76265571	65,6163252	8,038424328	0,424113341	0,000226108	SEC24 homolog D, COPII coa	SEC24D
ENSG00000099284	55,59926447	41,5035389	7,2148657	0,424502961	7,91E-06	macroH2A.2 histone	MACROH2A2
ENSG00000180626	9,124897581	6,781081193	4,794732953	0,424617163	0,006908629	zinc finger protein 594	ZNF594
ENSG00000103044	121,0853812	90,37276092	7,838823993	0,424815066	0,048245185	hyaluronan synthase 3	HAS3
ENSG00000162607	113,5995406	84,40374017	8,06034047	0,425602364	0,002062061	ubiquitin specific peptidase	USP1
ENSG00000159685	7,03848754	5,246151957	4,445485321	0,425687523	0,001695503	coiled-coil-helix-coiled-coil-l	CHCHD6
ENSG00000158163	15,90025857	11,80346059	5,310708868	0,425868251	0,000484749	DAZ interacting zinc finger p	DZIP1L
ENSG00000164402	159,2156061	117,9232437	8,684390505	0,425972877	0,000626978	septin 8	SEPTIN8
ENSG00000176890	344,9889683	257,9287166	9,619440298	0,426036655	0,007659648	thymidylate synthetase	TYMS
ENSG00000103187	249,7478614	186,3617432	9,498969515	0,426700478	0,002030986	coactosin like F-actin bindin	COTL1
ENSG00000144645	89,59179603	66,75397462	8,168272687	0,427242945	0,033373691	oxysterol binding protein lik	OSBPL10
ENSG00000253626	10,12332824	7,473570636	5,068659028	0,427502118	0,045618796	eukaryotic translation initiat	EIF5AL1
ENSG00000198889	34,73222712	25,67824658	6,199878221	0,427777906	0,003823063	DDB1 and CUL4 associated f	DCAF12L1
ENSG00000130413	14,29124539	10,57922532	5,418030387	0,428061966	0,028995679	serine/threonine kinase 33	STK33
ENSG00000065621	21,1599921	15,71440442	5,706250497	0,428338399	7,05E-05	glutathione S-transferase or	GSTO2
ENSG00000106077	24,45568178	18,14235159	6,150971433	0,428404758	0,000117369	abhydrolase domain contair	ABHD11
ENSG00000132016	7,551616042	5,595866591	4,562920446	0,429385548	0,018328719	break repair meiotic recomb	BRME1
ENSG00000117676	154,9804492	112,5177192	8,641469384	0,429848479	0,016497035	ribosomal protein S6 kinase	RPS6KA1
ENSG00000204390	3,650762954	2,717326336	3,565591445	0,43105459	0,015295781	heat shock protein family A	HSPA1L
ENSG00000124207	237,2231276	173,6857652	9,351466477	0,431169315	1,12E-05	chromosome segregation 1	CSE1L
ENSG00000143977	49,69523887	36,63607556	6,985177383	0,431624164	0,021308721	small nuclear ribonucleopro	SNRPG
ENSG00000072786	74,50648482	54,82840661	7,695604684	0,431817722	0,035527824	serine/threonine kinase 10	STK10
ENSG00000178814	27,560986	20,08597286	6,432315631	0,432274064	0,001973524	5-oxoprolinase, ATP-hydroly	OPLAH
ENSG00000119403	80,04819276	59,15963476	7,489248703	0,432284407	0,012286673	PHD finger protein 19	PHF19
ENSG00000171150	44,93035619	33,05767597	7,021709236	0,432335203	0,000123894	suppressor of cytokine signa	SOCS5
ENSG00000125347	68,09851574	50,34309993	7,465713719	0,432579181	7,77E-05	interferon regulatory factor	IRF1
ENSG00000183765	21,23748452	15,50368144	5,929667974	0,432592966	4,00E-05	checkpoint kinase 2	CHEK2
ENSG00000229124	13,22067609	9,836520405	5,332456427	0,433535983	0,043235046	VIM antisense RNA 1	VIM-AS1
ENSG00000135211	7,410872751	5,452002576	4,376416254	0,434941968	0,005054437	transmembrane protein 60	TMEM60
ENSG00000006062	51,18703194	36,36682367	7,046094599	0,435526838	0,02263753	mitogen-activated protein k	MAP3K14
ENSG00000139133	9,573098454	7,006777493	4,796984352	0,436095915	0,008925025	ALG10 alpha-1,2-glucosyltra	ALG10
ENSG00000223960	13,23576951	9,688817057	5,293279707	0,436222946	0,003794988	cholesterol induced regulatc	CHROMR
ENSG00000174928	2,786698684	2,003966367	3,234534326	0,436302002	0,02751311	chromosome 3 open reading	C3orf33
ENSG00000128973	23,96041429	17,71732512	5,784834983	0,437219802	0,001055317	CLN6 transmembrane ER prı	CLN6
ENSG00000119632	34,45948706	25,46983172	6,823349114	0,437527457	2,32E-05	interferon alpha inducible p	IFI27L2
ENSG00000145908	13,96015063	10,2016199	5,485910076	0,43859282	0,007024471	zinc finger protein 300	ZNF300
ENSG00000154429	19,7463023	14,56668408	5,789861028	0,438603562	0,018238722	centriole, cilia and spindle a	CCSAP
ENSG00000130844	23,85860663	17,03685709	6,336845997	0,439058197	0,025911144	zinc finger protein 331	ZNF331
ENSG00000127124	60,55270923	44,72379563	7,322212368	0,439649594	0,000600055	HIVEP zinc finger 3	HIVEP3
ENSG00000149929	53,23495604	38,96065554	7,109171175	0,440033022	0,000485322	HIRA interacting protein 3	HIRIP3
ENSG00000145375	23,97417815	17,35917363	6,06524501	0,440148332	0,010940455	spermatogenesis associated	SPATA5
ENSG00000142227	57,79080285	42,70893213	7,294207429	0,440721623	0,004589323	epithelial membrane protei	EMP3
ENSG00000068784	20,8680477	15,35692993	5,904866644	0,441192376	8,65E-07	S1 RNA binding domain 1	SRBD1
ENSG00000277462	4,339801696	3,133625845	3,500210024	0,441466185	0,003641425	zinc finger protein 670	ZNF670
ENSG00000148019	76,87344953	55,900952	7,606422839	0,442715935	0,00056866	centrosomal protein 78	CEP78
ENSG00000163545	45,97117936	33,54559322	7,128265921	0,442778573	0,002062061	NUAK family kinase 2	NUAK2
ENSG00000140682	114,9683349	84,9121237	8,081517398	0,442811701	0,026220665	transforming growth factor	TGFB1I1
ENSG00000170542	41,60988966	30,80565842	6,636928331	0,443641887	0,041419883	serpin family B member 9	SERPINB9
ENSG00000228109	13,29505196	9,766870035	5,055913087	0,444314052	0,023108546	MELTF antisense RNA 1	MELTF-AS1
ENSG00000115363	34,84180261	25,50197137	6,636912677	0,445117909	0,033696989	eva-1 homolog A, regulator	EVA1A
ENSG00000164306	22,89427482	16,72463114	5,878427401	0,445579744	3,77E-05	primase and DNA directed p	PRIMPOL
ENSG00000284024	6,531924595	4,768054977	4,197370281	0,445960851	0,004243538	zinc finger and SCAN domair	LOC100421372
ENSG00000131462	162,267162	116,6831982	8,682965989	0,446455111	0,001015434	tubulin gamma 1	TUBG1
ENSG00000133805	32,64035618	24,08968211	6,396188211	0,44653011	0,019266073	adenosine monophosphate	AMPD3
ENSG00000015475	38,41300337	28,2361524	6,542842511	0,446810009	0,001806671	BH3 interacting domain dea	BID
ENSG00000164032	147,0621833	106,9348022	8,519118873	0,446838621	0,005568261	H2A.Z variant histone 1	H2AZ1
ENSG00000103021	5,988546495	4,360326728	4,563754414	0,447347996	0,024184921	coiled-coil domain containin	CCDC113
ENSG00000169188	72,47575414	52,45677115	7,594479131	0,448012254	6,40E-06	apurinic/apyrimidinic endod	APEX2
ENSG00000213047	17,15572549	12,42127872	5,605694828	0,448245468	5,72E-05	DENN domain containing 1B	DENND1B
ENSG00000164105	16,77290361	12,2475056	5,55621513	0,449284498	0,004740771	Sin3A associated protein 30	SAP30
ENSG00000166788	22,60399213	16,44765623	5,970049222	0,449359392	1,30E-05	serum amyloid A like 1	SAAL1
ENSG00000173193	188,2218462	136,7840553	8,958927825	0,449946113	0,00316802	poly(ADP-ribose) polymeras	PARP14
ENSG00000168778	26,77869835	19,29262272	6,265044785	0,450478503	0,0002757	tectonic family member 2	TCTN2
ENSG00000107798	67,80883889	49,32418542	7,65045062	0,451367648	0,003284616	lipase A, lysosomal acid type	LIPA
ENSG00000132646	279,0806409	204,0609106	9,424757193	0,451384436	0,001025248	proliferating cell nuclear ant	PCNA
ENSG00000075618	426,1031299	312,2724298	10,28313234	0,451787862	0,003148066	fascin actin-bundling proteir	FSCN1
ENSG00000113569	114,3811818	82,4731343	8,135693766	0,451929167	0,000393363	nucleoporin 155	NUP155
ENSG00000155090	122,6498884	89,99426974	8,397851427	0,452313536	1,60E-05	Kruppel like factor 10	KLF10
ENSG00000082497	42,58386054	31,33819998	6,458832644	0,452856486	0,032574897	SERTA domain containing 4	SERTAD4
ENSG00000134574	108,1470775	78,91028606	8,110088378	0,454180782	0,015418764	damage specific DNA bindin	DDB2
ENSG00000197372	11,15379048	8,155619078	4,997331501	0,45432232	5,34E-07	zinc finger protein 675	ZNF675
ENSG00000130005	89,88573238	65,58682722	7,93865315	0,454664253	0,001571003	guanidinoacetate N-methylt	GAMT
ENSG00000214595	6,946714437	4,913682235	3,968435351	0,455593078	0,015590478	EMAP like 6	EML6
ENSG00000244509	223,5729763	163,374195	9,312823653	0,456063757	0,003816326	apolipoprotein B mRNA edit	APOBEC3C
ENSG00000187840	80,15273626	58,11936185	7,839379763	0,456102371	0,000719242	eukaryotic translation initiat	EIF4EBP1
ENSG00000167081	39,38981859	28,51911862	6,625493138	0,456190123	0,003482816	PBX homeobox 3	PBX3
ENSG00000143375	15,09911723	10,84132405	5,114792564	0,456263942	0,007260172	cingulin	CGN
ENSG00000188112	46,06848237	33,42853224	7,061496342	0,456291941	0,001360767	chromosome 6 open reading	C6orf132
ENSG00000138821	46,83036743	34,1645754	7,09358331	0,457639756	3,30E-05	solute carrier family 39 men	SLC39A8
ENSG00000113790	32,06761458	23,10584893	6,764780063	0,457661621	0,00578139	enoyl-CoA hydratase and 3-l	EHHADH
ENSG00000198246	8,978934167	6,286397294	5,100227288	0,458013276	0,008517521	solute carrier family 29 men	SLC29A3
ENSG00000031081	35,33202806	25,23656247	6,839967938	0,458135278	0,014399708	Rho GTPase activating prote	ARHGAP31
ENSG00000157514	82,34335302	59,82150787	8,434052471	0,458208817	0,009824389	TSC22 domain family memb	TSC22D3
ENSG00000112249	278,2364791	202,2570491	9,565686648	0,458501405	7,01E-13	activating signal cointegrato	ASCC3
ENSG00000166483	64,05022952	46,81197306	7,386248752	0,458516795	0,000688927	WEE1 G2 checkpoint kinase	WEE1
ENSG00000173540	58,06751924	42,16396195	7,401028922	0,458768497	0,000404685	GDP-mannose pyrophospho	GMPPB
ENSG00000188921	31,10330439	22,67001279	6,239522316	0,458900694	0,003217735	3-hydroxyacyl-CoA dehydrat	HACD4
ENSG00000165359	9,785758365	7,048556865	4,694156482	0,459364141	0,03645534	integrator complex subunit (	INTS6L
ENSG00000178662	8,103443525	5,887002692	4,7116287	0,459630908	0,036124592	cysteine and serine rich nucl	CSRNP3
ENSG00000101624	15,96228382	11,46199318	5,421750691	0,459727072	0,006409312	centrosomal protein 76	CEP76
ENSG00000133935	49,81881827	35,98537107	7,201279064	0,459748899	0,02364316	ergosterol biosynthesis 28 h	ERG28
ENSG00000167747	126,6244567	91,77060886	8,269692051	0,460003295	0,012860605	multidrug resistance-relatec	C19orf48
ENSG00000147813	65,5803871	44,50887516	7,578422117	0,46024278	0,010061991	nicotinate phosphoribosyltra	NAPRT
ENSG00000089486	181,1108139	131,4898996	8,67298835	0,460397211	1,02E-05	cell death inducing p53 targ	CDIP1
ENSG00000169499	106,2590972	77,37673067	8,427342988	0,460696106	1,28E-08	pleckstrin homology domair	PLEKHA2
ENSG00000123737	33,40202212	24,11853156	6,394061911	0,460841267	0,000196204	exosome component 9	EXOSC9
ENSG00000244486	48,12741762	35,03930649	6,911389401	0,460934683	0,012965427	scavenger receptor class F n	SCARF2
ENSG00000120549	151,5268848	110,9712061	8,623763203	0,461020543	0,008957196	KIAA1217	KIAA1217
ENSG00000111445	54,07715383	39,01824796	7,158787526	0,46110061	0,000834601	replication factor C subunit !	RFC5

ENSG00000133619	29,82586723	21,49048574	6,394666699	0,461269572	0,024508116	KRAB-A domain containing 1	KRBA1
ENSG00000168916	144,704757	105,4710674	8,808889033	0,461644817	0,00689878	zinc finger protein 608	ZNF608
ENSG00000168393	66,06186986	47,62781017	7,418770249	0,461981367	0,002700855	deoxythymidylate kinase	DTYMK
ENSG00000196440	17,88786903	13,00146185	5,965395966	0,462401618	0,002697738	armadillo repeat containing	ARMCX4
ENSG00000156299	32,02120302	22,95829971	6,164305475	0,462427309	0,007219921	TIAM Rac1 associated GEF 1	TIAM1
ENSG00000171792	41,17260884	29,13375105	6,873036809	0,462967365	0,001282799	RAD9-HUS1-RAD1 interactin	RHNO1
ENSG00000062822	130,1126999	93,34351774	8,428799075	0,463119074	0,000200741	DNA polymerase delta 1, cat	POLD1
ENSG00000177432	12,5591487	9,13264703	5,226661718	0,463582669	0,005332561	nucleosome assembly prote	NAP1L5
ENSG00000102743	20,13829	14,05436578	5,545448932	0,463659037	0,011580532	solute carrier family 25 mem	SLC25A15
ENSG00000176014	430,1650228	307,5919562	10,19929745	0,464052995	0,002606905	tubulin beta 6 class V	TUBB6
ENSG00000261801	16,02721559	11,62483339	5,655460246	0,465456603	0,003284616	LOXL1 antisense RNA 1	LOXL1-AS1
ENSG00000196110	7,190116604	5,204491754	4,406885674	0,465568219	0,004383244	zinc finger protein 699	ZNF699
ENSG00000111860	28,24998365	20,34497439	6,356503795	0,466162002	0,000285904	centrosomal protein 85 like	CEP85L
ENSG00000276170	5,761145532	4,168719909	3,853450253	0,46628701	0,01993026	uncharacterized LOC101929	LOC101929494
ENSG00000074527	83,27471517	61,18666619	7,876319695	0,466690733	0,017398103	netrin 4	NTN4
ENSG00000171224	9,648700353	6,973327723	4,915476822	0,467377796	0,000638978	family with sequence similar	FAM241B
ENSG00000147383	36,22929575	25,84361935	6,777487705	0,467735585	0,021184022	NAD(P) dependent steroid d	NSDHL
ENSG00000109971	1340,739308	965,2350007	11,69624157	0,468372272	6,73E-05	heat shock protein family A	HSPA8
ENSG00000154640	56,99584396	41,21015903	7,085692141	0,468697409	0,002770886	BTG anti-proliferation factor	BTG3
ENSG00000179862	81,99678074	55,91127887	8,054716708	0,468867829	0,003129579	Cbp/p300 interacting transa	CITED4
ENSG00000081913	44,55569432	32,21811653	6,817145735	0,469002083	0,018434246	PH domain and leucine rich	PHLPP1
ENSG00000247240	3,062864178	2,182242401	3,186644414	0,46973487	0,01630056	UBL7 divergent transcript	UBL7-DT
ENSG00000204219	54,48031103	38,22048592	7,250723408	0,470139836	0,00801449	transcription elongation fact	TCEA3
ENSG00000206503	519,4363436	368,9420278	10,88540823	0,470202224	0,010804101	major histocompatibility cor	HLA-A
ENSG00000162512	563,9664915	406,8827765	10,55749475	0,470673862	9,00E-07	syndecan 3	SDC3
ENSG00000077514	64,01610705	45,50724125	7,249326007	0,470733035	0,000596833	DNA polymerase delta 3, acc	POLD3
ENSG00000123136	80,57712821	56,53560784	7,656903473	0,472162991	0,004139048	DExD-box helicase 39A	DDX39A
ENSG00000142871	345,596642	248,9159174	10,13041655	0,473638663	0,004087921	cellular communication netw	CCN1
ENSG00000169689	38,23554121	27,2931599	6,632304882	0,473780943	0,022463512	centromere protein X	CENPX
ENSG00000173156	56,84647598	40,94087326	7,058579756	0,474921683	0,001876442	ras homolog family member	RHOD
ENSG00000112655	367,9493942	265,8079831	10,00940482	0,474987653	0,000332205	protein tyrosine kinase 7 (in	PTK7
ENSG00000174099	49,55401665	35,88490968	7,137437655	0,475006595	0,019979742	methionine sulfoxide reduct	MSRB3
ENSG00000146757	21,68159401	15,63059771	6,034817185	0,47519645	2,03E-07	zinc finger protein 92	ZNF92
ENSG00000182253	30,02218409	21,59189518	5,916346342	0,475431162	0,042955766	synemin	SYNM
ENSG00000064666	430,8389057	310,1271728	10,39820051	0,475532285	1,94E-06	calponin 2	CNN2
ENSG00000175643	54,39111865	39,62562356	6,613478975	0,475695338	0,004395698	RecQ mediated genome inst	RMI2
ENSG00000170537	4,140587661	2,942821557	3,376569689	0,475738363	0,032064884	transmembrane channel like	TMC7
ENSG00000099977	39,89105165	28,6105333	6,727362535	0,476310802	0,000978262	D-dopachrome tautomerase	DDT
ENSG00000010219	10,60114353	7,629178657	5,158140573	0,476380031	1,18E-07	dual specificity tyrosine pho	DYRK4
ENSG00000180155	48,66616754	34,92622151	7,516443904	0,476449692	0,013802001	Ly6/neurotoxin 1	LYNX1
ENSG00000158201	22,13551674	15,85972184	5,539148431	0,476558716	0,000150157	abhydrolase domain contair	ABHD3
ENSG00000170689	222,5665809	162,5873665	9,661101439	0,476709571	0,032715415	homeobox B9	HOXB9
ENSG00000159208	6,222293494	4,478082204	4,541950362	0,476947395	0,018224136	circadian associated repress	CIART
ENSG00000114346	95,97008288	67,5658358	8,039886145	0,477288191	4,08E-09	epithelial cell transforming 2	ECT2
ENSG00000173258	3,535807342	2,507167524	3,234715342	0,477924137	0,017387582	zinc finger protein 483	ZNF483
ENSG00000076242	79,42253244	56,7428127	7,767533656	0,478067584	1,57E-05	mutL homolog 1	MLH1
ENSG00000151090	50,9818713	36,29504318	7,064888627	0,478075514	0,002199689	thyroid hormone receptor b	THRB
ENSG00000171960	24,55995762	17,46891646	6,213167041	0,478380385	1,06E-05	peptidylprolyl isomerase H	PPIH
ENSG00000124588	23,62903479	16,51848818	6,069566938	0,478710255	0,015352575	N-ribosyldihydronicotinamic	NQO2
ENSG00000152061	29,91062227	21,41016827	6,266943147	0,479282936	8,65E-07	RAB GTPase activating prote	RABGAP1L
ENSG00000065308	309,4109038	222,030891	9,837682183	0,479817825	0,016205729	translocation associated me	TRAM2
ENSG00000112425	6,588527327	4,708965528	4,211680377	0,480515134	0,010947055	EPM2A glucan phosphatase,	EPM2A
ENSG00000123836	11,4809304	8,014445555	4,981779247	0,480900611	0,048295631	6-phosphofructo-2-kinase/fr	PFKFB2
ENSG00000167523	26,14784013	18,42152282	6,052560313	0,480941002	3,61E-10	spermatogenesis associated	SPATA33
ENSG00000138796	75,04696962	53,97521689	7,543516288	0,48219294	0,00029185	hydroxyacyl-CoA dehydroge	HADH
ENSG00000129295	8,489859696	5,861145188	4,59815727	0,482420365	5,26E-06	dynein axonemal assembly f	DNAAF11
ENSG00000120696	8,992724802	6,446619551	4,586975264	0,483231281	0,016827872	kelch repeat and BTB domai	KBTBD7
ENSG00000158169	37,15254664	26,47666479	6,653635956	0,483462738	0,000110821	FA complementation group	FANCC
ENSG00000214013	17,72402019	12,70743192	5,782506511	0,483687687	2,10E-07	glucosidase alpha, neutral C	GANC
ENSG00000226419	7,360438962	5,235649867	4,325903102	0,484050751	0,009995743	SLC16A1 antisense RNA 1	SLC16A1-AS1
ENSG00000135617	23,949786	17,03132931	6,055067468	0,484213334	0,002703261	protease associated domain	PRADC1
ENSG00000132780	278,8380747	199,0265774	9,379394136	0,484224893	1,53E-05	nuclear autoantigenic sperm	NASP
ENSG00000239672	24,42382574	17,40012592	6,066630653	0,484844003	1,85E-05	NME/NM23 nucleoside diph	NME1
ENSG00000197647	2,591307496	1,824276754	2,81905321	0,484846426	0,031641225	zinc finger protein 433	ZNF433
ENSG00000178202	53,04207401	38,1005608	7,016785155	0,485066012	0,012451049	protein O-glucosyltransferas	POGLUT3
ENSG00000141295	41,26583643	29,34896173	7,051363162	0,485159316	0,001157536	secernin 2	SCRN2
ENSG00000196935	118,1754636	84,52140761	8,099257404	0,485368946	0,001031246	SLIT-ROBO Rho GTPase activ	SRGAP1
ENSG00000196976	26,49095661	18,40503196	6,316558507	0,485404289	0,003641192	L antigen family member 3	LAGE3
ENSG00000152229	53,21210907	38,04843329	7,188699701	0,485574515	0,009168074	proline-serine-threonine ph	PSTPIP2
ENSG00000127824	170,7238151	122,1878461	8,911529127	0,486108591	0,021052883	tubulin alpha 4a	TUBA4A
ENSG00000112118	258,5254232	185,9958259	9,281454711	0,486286135	0,003253291	minichromosome maintenai	MCM3
ENSG00000136147	20,76135604	14,5728818	5,862060211	0,486702645	1,54E-05	PHD finger protein 11	PHF11
ENSG00000135245	20,48309075	14,60000502	6,034454443	0,48726567	0,004183784	hypoxia inducible lipid dropl	HILPDA
ENSG00000221995	5,548391233	3,937326452	4,072949774	0,487342678	0,02751311	myosin XVIIIA	MYO18A
ENSG00000174652	21,75187937	15,39850189	6,237564794	0,487483919	0,008156973	zinc finger protein 266	ZNF266
ENSG00000204519	10,04725616	7,14227691	4,754447225	0,487781274	0,000128182	zinc finger protein 551	ZNF551
ENSG00000178498	47,47309604	33,70966089	7,36780504	0,489021577	0,009122189	deltex E3 ubiquitin ligase 3	DTX3
ENSG00000182054	243,1406251	176,1569849	9,117384126	0,489082196	0,004316853	isocitrate dehydrogenase (N	IDH2
ENSG00000170779	45,44754266	32,28533782	6,674293919	0,489485503	0,007501056	cell division cycle associated	CDCA4
ENSG00000156500	3,835015079	2,686815805	3,457639059	0,489688322	0,017626368	PABIR family member 3	PABIR3
ENSG00000185483	28,37015004	20,23823042	6,340976951	0,489850919	0,003095248	receptor tyrosine kinase like	ROR1
ENSG00000184922	59,24479412	40,21664639	7,436063783	0,4899208	0,019730633	formin like 1	FMNL1
ENSG00000157693	16,26931672	11,59413852	5,449515021	0,490647717	0,014397204	transmembrane protein 268	TMEM268
ENSG00000138772	43,04698948	30,59893612	6,603609845	0,490767756	0,007370541	annexin A3	ANXA3
ENSG00000197008	8,579398306	6,020281953	4,710591558	0,490851969	0,000243336	zinc finger protein 138	ZNF138
ENSG00000156535	242,4209214	172,9300858	9,225747676	0,490908192	0,000499488	CD109 molecule	CD109
ENSG00000128595	413,98918	294,0764059	10,26925144	0,490948236	0,0001942	calumenin	CALU
ENSG00000204991	27,79590071	19,24066784	5,930997469	0,492047445	0,045398437	spire type actin nucleation fa	SPIRE2
ENSG00000106211	418,4580143	298,2813604	10,15544812	0,492108057	1,31E-10	heat shock protein family B	HSPB1
ENSG00000147804	19,08784183	13,38329534	5,692347616	0,492665616	0,021815046	solute carrier family 39 mem	SLC39A4
ENSG00000181827	97,63699	69,40225793	8,086989568	0,493593581	9,91E-06	regulatory factor X7	RFX7
ENSG00000100911	84,59792436	59,05282991	7,85651238	0,49449424	0,000409842	proteasome activator subun	PSME2
ENSG00000162783	118,4390436	82,91404655	8,390222929	0,496085584	0,026400725	immediate early response 5	IER5
ENSG00000175906	63,60078021	45,12618567	7,473492089	0,496519488	8,06E-06	ADP ribosylation factor like	ARL4D
ENSG00000168411	119,8706461	83,84227191	8,076761697	0,49725163	6,06E-05	ring finger and WD repeat d	RFWD3
ENSG00000125657	18,78750074	13,28418011	5,855076276	0,497264273	0,042954433	TNF superfamily member 9	TNFSF9
ENSG00000137267	57,72109734	40,21668139	7,815112424	0,497296315	0,033402502	tubulin beta 2A class IIa	TUBB2A
ENSG00000142910	131,3893713	93,60817378	8,705988341	0,497373619	0,023928322	tubulointerstitial nephritis a	TINAGL1
ENSG00000079819	144,5060112	102,9065819	8,534044196	0,49792999	0,00055934	erythrocyte membrane prot	EPB41L2
ENSG00000158292	30,45531936	21,48200301	6,395558126	0,49815305	0,002960082	G protein-coupled receptor	GPR153
ENSG00000171016	29,13074251	20,64622567	6,449719993	0,498571751	8,38E-07	pygopus family PHD finger 1	PYGO1
ENSG00000103966	165,4777528	116,5420666	8,727577752	0,498695284	0,001140828	EH domain containing 4	EHD4
ENSG00000108448	7,35588177	5,169562485	4,860545022	0,49950432	0,018497942	tripartite motif containing 1	TRIM16L
ENSG00000164904	147,6217393	102,9834031	8,493624279	0,500791594	0,00301821	aldehyde dehydrogenase 7 f	ALDH7A1
ENSG00000242193	9,234467874	6,544809795	4,851978446	0,500960325	1,40E-06	crystallin zeta like 2, pseudo	CRYZL2P
ENSG00000111196	15,55088473	10,99113246	5,281082592	0,501064256	0,000171252	mago homolog B, exon junct	MAGOHB
ENSG00000100908	15,29450891	10,7124777	5,257437567	0,501464271	0,04692073	ER membrane protein comp	EMC9
ENSG00000110200	16,70716298	11,75856097	5,65793486	0,501498775	0,00109819	anaphase promoting comple	ANAPC15
ENSG00000155755	31,56745298	22,33951225	6,538675747	0,501652439	0,009031165	transmembrane protein 237	TMEM237
ENSG00000091073	23,82213678	16,6541846	6,128777959	0,501938399	0,010547472	deltex E3 ubiquitin ligase 2	DTX2
ENSG00000232677	6,69137655	4,685520355	4,42506854	0,502412862	0,016005922	long intergenic non-protein	LINC00665
ENSG00000129116	300,4564171	214,2917473	9,5893761	0,502459407	0,00298938	palladin, cytoskeletal associ	PALLD
ENSG00000198556	9,089876066	6,331115905	4,679317741	0,502581157	0,000361076	zinc finger protein 789	ZNF789
ENSG00000110400	78,43564752	55,12891033	7,514123411	0,502844794	0,002288982	nectin cell adhesion molecul	NECTIN1
ENSG00000160051	10,55157578	7,379296806	4,72298168	0,504265247	0,00192597	IQ motif containing C	IQCC

ENSG00000105327	126,6557777	89,10641731	8,573454596	0,504767337	7,29E-06	BCL2 binding component 3	BBC3
ENSG00000111666	37,31630422	25,59944886	6,741294237	0,505397879	0,042058264	choline phosphotransferase	CHPT1
ENSG00000149679	35,85546756	25,04816415	6,667294739	0,505645592	4,23E-06	Cdk5 and Abl enzyme substr	CABLES2
ENSG00000180881	2,621707617	1,799856664	3,260721607	0,505799413	0,038200757	calcyphosine 2	CAPS2
ENSG00000278535	18,92579713	13,26033051	5,739071035	0,50592901	8,29E-05	dehydrogenase/reductase 1	DHRS11
ENSG00000134291	174,7501828	124,019735	8,710063845	0,50601741	0,00177835	transmembrane protein 106	TMEM106C
ENSG00000101412	63,25925392	44,39520422	7,208340787	0,506636427	0,023384566	E2F transcription factor 1	E2F1
ENSG00000198155	2,219338519	1,567992087	2,909831636	0,506889467	0,029866539	zinc finger protein 876, pseu	ZNF876P
ENSG00000125266	113,1170076	79,74259161	8,450819395	0,507156334	0,01937463	ephrin B2	EFNB2
ENSG00000152422	9,610866539	6,623607507	4,838411676	0,508540262	0,000535274	X-ray repair cross compleme	XRCC4
ENSG00000101347	154,1624903	103,3553087	8,498551416	0,508882289	0,036379049	SAM and HD domain contai	SAMHD1
ENSG00000163918	55,8799039	38,96718612	7,144918358	0,508990356	9,45E-05	replication factor C subunit	RFC4
ENSG00000152240	29,17440754	20,52762708	6,090905723	0,509201657	0,000774608	HAUS augmin like complex s	HAUS1
ENSG00000189308	44,4239211	30,44709769	6,947810133	0,509944045	9,14E-06	lin-54 DREAM MuvB core co	LIN54
ENSG00000145882	55,4515042	38,97254559	7,193523928	0,51017112	0,000302172	prenylcysteine oxidase 1 like	PCYOX1L
ENSG00000118257	196,2358349	137,7547575	9,101166494	0,510968448	0,0104024	neuropilin 2	NRP2
ENSG00000181026	121,277495	84,06047514	8,394007225	0,511434767	0,010349436	apoptosis enhancing nuclea	AEN
ENSG00000183048	16,07369805	11,28407465	5,535514763	0,511562545	0,004092143	solute carrier family 25 men	SLC25A10
ENSG00000177191	5,038406711	3,31054713	3,426832491	0,511626886	0,036759381	UDP-GlcNAc:betaGal beta-1	B3GNT8
ENSG00000122694	60,06700932	41,94551794	7,513609861	0,512026215	0,006528076	GLI pathogenesis related 2	GLIPR2
ENSG00000143416	30,5873968	21,55273376	6,858906238	0,512364077	0,001666675	selenium binding protein 1	SELENBP1
ENSG00000166881	144,2679868	98,36757913	8,527514844	0,512724503	0,001811013	nuclear envelope integral m	NEMP1
ENSG00000170421	756,6144937	534,1343623	10,87080601	0,513004216	0,000213325	keratin 8	KRT8
ENSG00000187676	18,78939209	13,21913764	5,435044507	0,513073702	0,010547472	beta 3-glucosyltransferase	B3GLCT
ENSG00000183840	18,12340811	12,68856523	5,586005524	0,513239923	3,58E-05	G protein-coupled receptor	GPR39
ENSG00000137563	79,84033661	54,03287532	7,796809534	0,513388244	0,000471793	gamma-glutamyl hydrolase	GGH
ENSG00000186153	12,58436016	8,845322664	5,012896908	0,513854413	0,001649592	WW domain containing oxid	WWOX
ENSG00000112715	491,3445136	346,442059	10,80485181	0,515145254	0,042437005	vascular endothelial growth	VEGFA
ENSG00000204516	55,38923527	38,8333245	6,845546419	0,515243943	0,022249542	MHC class I polypeptide-rela	MICB
ENSG00000131470	39,40797256	27,14610156	6,569962444	0,515683613	0,005992071	PSMC3 interacting protein	PSMC3IP
ENSG00000129353	201,0029846	139,7493295	9,013026411	0,516035253	0,000584568	solute carrier family 44 men	SLC44A2
ENSG00000090020	145,1195804	100,888902	8,556664248	0,517169178	0,000849546	solute carrier family 9 meml	SLC9A1
ENSG00000152078	34,76162863	23,81804099	6,276951046	0,517249528	0,00014357	TLC domain containing 4	TLCD4
ENSG00000173083	33,88884376	23,54168347	5,962671231	0,517512342	0,008936345	heparanase	HPSE
ENSG00000186806	5,374195785	3,736090068	4,023631092	0,517752944	0,0049665	V-set and immunoglobulin d	VSIG10L
ENSG00000156802	178,3960285	122,5250709	8,692613273	0,517972629	0,000681101	ATPase family AAA domain c	ATAD2
ENSG00000171723	31,98648499	22,32683616	6,644849424	0,518409067	1,35E-06	gephyrin	GPHN
ENSG00000197696	14,84777517	10,263481	5,616641779	0,519055847	9,15E-06	neuromedin B	NMB
ENSG00000151466	18,64663943	12,74373419	5,760777449	0,519698979	3,60E-08	sodium channel and clathrin	SCLT1
ENSG00000167460	878,837251	613,7748787	11,29328038	0,520406399	1,45E-05	tropomyosin 4	TPM4
ENSG00000169683	76,20255374	52,14773287	7,483980188	0,52110351	0,001719155	leucine rich repeat containir	LRRC45
ENSG00000156711	62,55711032	43,02112052	7,449804334	0,52151997	0,022707638	mitogen-activated protein k	MAPK13
ENSG00000241316	4,415984983	3,055896879	3,787893087	0,521981275	0,002084313	SUCLG2 divergent transcript	SUCLG2-DT
ENSG00000146592	23,14264612	16,28123076	6,128569858	0,522723301	0,049807042	cAMP responsive element b	CREB5
ENSG00000104738	361,4843798	253,0316674	9,707930986	0,523019715	0,002730086	minichromosome maintenai	MCM4
ENSG00000138623	61,74118626	42,91773776	7,652942327	0,523045072	0,022008548	semaphorin 7A (John Milton	SEMA7A
ENSG00000234127	107,6252077	74,0960039	8,045035572	0,523401351	0,003621326	tripartite motif containing 2	TRIM26
ENSG00000198208	8,836091113	5,968951605	4,412554928	0,523514298	0,029773405	ribosomal protein S6 kinase	RPS6KL1
ENSG00000169105	75,91253387	52,8978952	7,751831843	0,523636936	3,09E-07	carbohydrate sulfotransfera	CHST14
ENSG00000125885	99,05634482	66,45517146	8,080339961	0,523916987	5,64E-05	minichromosome maintenai	MCM8
ENSG00000183853	411,0385383	286,5044149	10,28228555	0,524274436	1,64E-10	kirre like nephrin family adh	KIRREL1
ENSG00000063660	619,3558709	430,3789796	10,69291577	0,524403597	0,001282799	glypican 1	GPC1
ENSG00000141569	36,28113724	24,80297819	6,766843649	0,524743582	0,000356741	tripartite motif containing 6	TRIM65
ENSG00000119673	11,68291016	8,091854274	4,948097272	0,52502966	0,000422308	acyl-CoA thioesterase 2	ACOT2
ENSG00000248487	7,46987827	5,182257574	4,433522445	0,525312704	0,000481781	abhydrolase domain contair	ABHD14A
ENSG00000204388	90,82647281	62,78909902	7,714688118	0,52596996	5,01E-05	heat shock protein family A	HSPA1B
ENSG00000149636	48,57567664	33,70999986	6,958680184	0,526325871	0,000293706	DSN1 component of MIS12 I	DSN1
ENSG00000168803	13,47107376	9,255973437	5,314606378	0,52643677	9,93E-06	adenosine deaminase like	ADAL
ENSG00000145014	51,58739707	35,59966069	7,196121792	0,526468346	0,002838481	transmembrane protein 44	TMEM44
ENSG00000006634	17,65580386	11,97348894	5,461386732	0,526532287	0,000852854	DBF4 zinc finger	DBF4
ENSG00000254122	5,108953542	3,533007778	4,640315566	0,527290173	0,046979364	protocadherin gamma subfa	PCDHGB7
ENSG00000180425	3,88405502	2,686465389	3,447988919	0,527337895	0,007591431	chromosome 11 open readi	C11orf71
ENSG00000185133	11,89474411	8,251805486	5,190622256	0,527484191	0,044518902	inositol polyphosphate-5-ph	INPP5J
ENSG00000100714	180,7373498	125,4064158	8,852605096	0,527865664	8,99E-08	methylenetetrahydrofolate	MTHFD1
ENSG00000184785	7,731139715	5,360877689	4,312431724	0,527974198	0,011772054	small integral membrane pr	SMIM10
ENSG00000259768	29,99839395	20,51594821	6,456154422	0,528285927	0,000670144	ZFHX3 antisense RNA 1	ZFHX3-AS1
ENSG00000106366	1649,421885	1152,767907	12,42147288	0,528340745	0,003896916	serpin family E member 1	SERPINE1
ENSG00000184445	76,52557047	52,33315461	7,432920522	0,530047409	0,000158605	kinetochore associated 1	KNTC1
ENSG00000101000	40,72029881	28,38880137	6,988717602	0,530080721	0,008199459	protein C receptor	PROCR
ENSG00000177565	268,5895563	187,7589864	9,344684888	0,530184564	0,01706944	TBL1X receptor 1	TBL1XR1
ENSG00000181104	94,66247733	65,55002071	8,074779273	0,531063894	0,001939296	coagulation factor II thromb	F2R
ENSG00000178921	86,34967808	58,81314919	7,940062097	0,531139279	2,49E-06	phosphoribosylformylglycin	PFAS
ENSG00000272695	16,74658093	11,61071127	5,561929905	0,531636115	0,014006494	GAS6 divergent transcript	GAS6-DT
ENSG00000182158	223,8771041	155,0973509	9,415423092	0,532239047	0,000972041	cAMP responsive element b	CREB3L2
ENSG00000088280	75,31739387	51,68424989	7,641617634	0,532872897	0,000436346	ArfGAP with SH3 domain, ar	ASAP3
ENSG00000026950	26,73796798	18,49465022	6,433229732	0,533178815	0,000401918	butyrophilin subfamily 3 me	BTN3A1
ENSG00000132196	16,33201871	10,90294274	5,457925915	0,533350845	0,021637639	hydroxysteroid 17-beta deh	HSD17B7
ENSG00000108439	63,52675444	43,73033609	7,487883865	0,533762119	8,38E-09	pyridoxamine 5'-phosphate	PNPO
ENSG00000137404	47,98501094	33,23180836	6,824125793	0,533980421	0,001186384	nurim	NRM
ENSG00000280594	2,696192383	1,847812433	3,029742633	0,534087667	0,043691907	BTG3 antisense RNA 1	BTG3-AS1
ENSG00000161642	156,0048814	105,1858266	8,620815997	0,534242315	0,035527824	zinc finger protein 385A	ZNF385A
ENSG00000187790	15,36799843	10,33170029	5,555017906	0,534594392	0,001810682	FA complementation group	FANCM
ENSG00000159335	320,1947941	219,9238695	9,665614567	0,534606346	0,000177413	parathymosin	PTMS
ENSG00000203499	3,680385149	2,529378622	3,381607253	0,53634666	0,002181812	IQ motif and ankyrin repeat	IQANK1
ENSG00000174791	61,80410942	41,46560974	7,471431183	0,536742155	0,004157847	Ras and Rab interactor 1	RIN1
ENSG00000143248	2,471058965	1,689003003	3,233153354	0,53683211	0,01288489	regulator of G protein signal	RGS5
ENSG00000029639	14,79369692	9,901517778	5,297082018	0,537485055	0,029796212	transcription factor B1, mito	TFB1M
ENSG00000128641	176,5335829	121,9730397	9,048380509	0,537490113	2,43E-05	myosin IB	MYO1B
ENSG00000117010	2,564816863	1,74582237	3,084294654	0,537818513	0,006794835	zinc finger protein 684	ZNF684
ENSG00000125871	45,24119751	30,35787271	7,030984302	0,539211877	0,00014298	mitochondrial genome main	MGME1
ENSG00000165105	32,12688776	22,30304948	6,059368672	0,539296103	0,020740421	RAS and EF-hand domain co	RASEF
ENSG00000277639	11,96913221	8,228006855	4,930048176	0,539895023	3,05E-05	uncharacterized LOC105371	LOC105371267
ENSG00000197852	81,07163537	55,07376891	7,689506955	0,540641915	0,004401626	inka box actin regulator 2	INKA2
ENSG00000054967	31,57434579	21,80961126	6,356403759	0,540685787	0,009697064	RELT TNF receptor	RELT
ENSG00000175318	14,72254038	9,533061929	5,1714052	0,541281139	0,041269359	GRAM domain containing 2/	GRAMD2A
ENSG00000120913	14,02574752	9,641118628	5,583890876	0,541465897	0,021138133	PDZ and LIM domain 2	PDLIM2
ENSG00000063241	30,75490832	21,05579728	6,582812392	0,542533413	5,75E-05	isochorismatase domain cor	ISOC2
ENSG00000095383	312,2316132	215,0554361	9,718720936	0,543088897	0,001885261	TBC1 domain family membe	TBC1D2
ENSG00000092470	69,0171693	47,54799646	7,110525082	0,54334199	0,007202979	WD repeat domain 76	WDR76
ENSG00000173517	88,65441146	60,55846952	7,889194045	0,543562586	0,000468991	pseudopodium enriched aty	PEAK1
ENSG00000103257	162,9288331	111,4624641	8,885993467	0,543781881	0,01124704	solute carrier family 7 meml	SLC7A5
ENSG00000166845	43,52353221	29,2339875	7,002523966	0,544109463	1,94E-05	chromosome 18 open readi	C18orf54
ENSG00000100599	35,49502145	24,30790888	6,666910826	0,544453567	6,80E-05	Ras and Rab interactor 3	RIN3
ENSG00000133302	30,88927614	20,59920903	6,180199806	0,544968183	0,001929184	SMC5-SMC6 complex localiz	SLF1
ENSG00000070882	114,0502825	77,83277563	8,407615979	0,545208636	0,000136246	oxysterol binding protein lik	OSBPL3
ENSG00000196083	25,38120509	17,42963136	6,211483512	0,545309821	0,000815592	interleukin 1 receptor acces	IL1RAP
ENSG00000072682	236,3409411	162,5642489	9,579789681	0,545609289	4,36E-07	prolyl 4-hydroxylase subunit	P4HA2
ENSG00000072110	935,042281	640,9417952	11,39504231	0,546494249	1,54E-10	actinin alpha 1	ACTN1
ENSG00000270885	11,58299902	7,903414649	5,212634123	0,546893809	0,035117921	RAS like family 10 member E	RASL10B
ENSG00000172292	131,6999049	89,59281774	8,338652198	0,547372203	6,34E-06	ceramide synthase 6	CERS6
ENSG00000115902	86,12833369	58,43324148	7,530842674	0,547973274	0,035494979	solute carrier family 1 meml	SLC1A4
ENSG00000133247	40,74760042	27,73752309	6,790711384	0,547995333	9,03E-05	lysine methyltransferase 5C	KMT5C
ENSG00000163235	243,2333874	170,3611892	9,344617341	0,549076659	0,040596397	transforming growth factor	TGFA
ENSG00000075223	79,81222301	54,79974935	8,243531873	0,549855345	0,000293632	semaphorin 3C	SEMA3C
ENSG00000158555	48,33680974	33,13797068	6,885586609	0,550439189	0,005720618	glycerophosphodiester phos	GDPD5

ENSG00000152284	26,8940816	18,40338638	6,275021267	0,55054376	0,01565531	transcription factor 7 like 1	TCF7L1
ENSG00000121005	16,69733299	11,13907672	5,480399188	0,550616426	0,005980585	cysteine rich secretory prote	CRISPLD1
ENSG00000137124	99,53714241	67,99966145	8,247179381	0,551185858	0,000232174	aldehyde dehydrogenase 1 f	ALDH1B1
ENSG00000260456	2,648958728	1,77977114	2,685327185	0,551281587	0,03501651	chromosome 16 open readi	C16orf95
ENSG00000070087	174,031904	118,0871735	9,022034217	0,552813607	0,000572902	profilin 2	PFN2
ENSG00000177606	251,6686181	173,3417358	9,549482132	0,553154404	0,001777492	Jun proto-oncogene, AP-1 tr	JUN
ENSG00000181467	106,5149885	72,09333279	8,232526973	0,553286575	3,96E-05	RAP2B, member of RAS onc	RAP2B
ENSG00000204934	7,435962994	4,979893614	4,424394742	0,553728134	0,006726671	ATP6V0E2 antisense RNA 1	ATP6V0E2-AS1
ENSG00000175806	11,67439133	7,963274202	4,957692879	0,554348125	0,000675351	methionine sulfoxide reduct	MSRA
ENSG00000050165	523,3337739	356,26432	10,45750747	0,55494193	0,019036994	dickkopf WNT signaling path	DKK3
ENSG00000213988	5,635102339	3,795072266	4,313441086	0,555520643	0,00194237	zinc finger protein 90	ZNF90
ENSG00000183087	545,2176855	374,3211178	10,76098239	0,555661177	0,009625515	growth arrest specific 6	GAS6
ENSG00000147394	124,1890093	84,67328321	8,341763432	0,556026402	0,010577882	zinc finger protein 185 with	ZNF185
ENSG00000161981	40,70701439	27,46338826	6,710278294	0,556094359	0,00105487	small nuclear ribonucleopro	SNRNP25
ENSG00000073050	79,57721761	53,95206409	7,829711472	0,556238922	2,24E-08	X-ray repair cross compleme	XRCC1
ENSG00000157193	20,48645763	13,86247374	5,4383914	0,556858415	0,028775121	LDL receptor related protein	LRP8
ENSG00000150967	20,37369293	13,86684631	5,896932214	0,556920326	9,06E-05	ATP binding cassette subfan	ABCB9
ENSG00000081052	314,1142459	211,5479246	9,774722042	0,557306942	0,01529117	collagen type IV alpha 4 cha	COL4A4
ENSG00000163293	9,71886993	6,60383182	4,459278282	0,557807565	0,000103909	NIPA like domain containing	NIPAL1
ENSG00000092068	39,98616439	26,4470696	5,873941915	0,558034097	0,010677887	solute carrier family 7 meml	SLC7A8
ENSG00000143401	73,93853052	50,19708353	7,468665779	0,559989939	1,88E-11	acidic nuclear phosphoprote	ANP32E
ENSG00000164002	15,47288188	10,28040011	5,340126824	0,561076934	0,000343125	exonuclease 5	EXO5
ENSG00000112877	11,75875354	7,755375061	5,063583488	0,561133049	0,008275394	centrosomal protein 72	CEP72
ENSG00000041880	48,71784634	32,7272824	7,219087053	0,561716336	2,94E-09	poly(ADP-ribose) polymeras	PARP3
ENSG00000108384	44,42361085	29,72663962	6,909422636	0,563070552	0,001110468	RAD51 paralog C	RAD51C
ENSG00000204264	76,24106371	51,08128444	7,714866209	0,563317787	1,45E-07	proteasome 20S subunit bet	PSMB8
ENSG00000184207	65,01195733	42,91486281	7,511669635	0,563853364	0,001179119	phosphoglycolate phosphat	PGP
ENSG00000121281	84,51898176	57,14003939	7,713417331	0,563899076	8,17E-06	adenylate cyclase 7	ADCY7
ENSG00000167550	6,615152341	4,323976762	4,324840862	0,564038146	0,019508136	RHEB like 1	RHEBL1
ENSG00000131153	36,28593591	24,75371707	6,35542245	0,56459824	0,033569623	GINS complex subunit 2	GINS2
ENSG00000186575	218,2670196	147,53751	9,160603889	0,565912597	0,000347841	NF2, moesin-ezrin-radixin lik	NF2
ENSG00000097021	126,0172926	83,55732572	8,149088548	0,567424385	0,016159894	acyl-CoA thioesterase 7	ACOT7
ENSG00000189403	258,3919624	171,3363965	9,439855622	0,567613479	8,88E-06	high mobility group box 1	HMGB1
ENSG00000198298	4,02505132	2,650716874	3,553192074	0,567905964	0,004514256	zinc finger protein 485	ZNF485
ENSG00000137936	81,99396022	54,87352804	7,917977168	0,568836179	0,023143441	BCAR3 adaptor protein, NSP	BCAR3
ENSG00000166896	6,033340564	4,028908253	4,167109205	0,56896167	0,002269337	ATP23 metallopeptidase anc	ATP23
ENSG00000234882	2,601558794	1,734702113	2,835084328	0,569728904	0,00888699	eukaryotic translation initiat	EIF3EP1
ENSG00000257337	10,22533399	6,892433099	4,788578474	0,569849023	0,001283693	TNS2 antisense RNA 1	TNS2-AS1
ENSG00000112183	6,947694865	4,686422266	4,415262816	0,571612266	0,00076167	RNA binding motif protein 2	RBM24
ENSG00000132967	37,72666977	24,94900428	6,70568176	0,572361201	3,02E-05	high mobility group box 1 ps	HMGB1P5
ENSG00000186280	3,085771836	1,969797514	3,343997955	0,572409516	0,043532415	lysine demethylase 4D	KDM4D
ENSG00000159231	14,44205715	9,607166064	4,87305706	0,572776584	0,038916076	carbonyl reductase 3	CBR3
ENSG00000121297	36,13977862	24,02947828	6,495763315	0,573334788	0,0184984	teashirt zinc finger homeob	TSHZ3
ENSG00000106268	29,89512797	19,70587091	6,522976193	0,573659983	1,72E-06	nudix hydrolase 1	NUDT1
ENSG00000255150	3,00802337	2,025138136	2,890122194	0,574008472	0,014470247	EP300 interacting inhibitor c	EID3
ENSG00000104863	4,833692155	3,244461254	3,781016643	0,574368959	0,005178252	lin-7 homolog B, crumbs cell	LIN7B
ENSG00000106785	63,37336618	41,4581012	7,374013612	0,574865504	0,008623196	tripartite motif containing 1	TRIM14
ENSG00000123374	156,7972295	104,557551	8,583787222	0,575504408	1,66E-06	cyclin dependent kinase 2	CDK2
ENSG00000106991	138,0520696	93,35628611	8,698414141	0,5763358	0,002930276	endoglin	ENG
ENSG00000139410	16,04454384	10,80299712	5,641617814	0,57638779	4,32E-05	serine dehydratase like	SDSL
ENSG00000187391	4,274123641	2,86353601	3,806964351	0,576760335	0,032690993	membrane associated guan	MAGI2
ENSG00000140025	14,54337165	9,489839592	5,433422164	0,576793405	0,012561183	EF-hand calcium binding do	EFCAB11
ENSG00000112186	31,60364073	21,15423477	6,426883146	0,57719476	0,000235316	cyclase associated actin cyto	CAP2
ENSG00000269825	11,25949965	7,438475285	4,635394686	0,577719565	8,82E-05	Zinc finger protein LOC1225	LOC122539214
ENSG00000011422	56,85057838	38,03065516	7,279874755	0,57772176	0,00895804	plasminogen activator, urok	PLAUR
ENSG00000033170	73,09100497	48,93954559	7,690196659	0,578790937	0,002198273	fucosyltransferase 8	FUT8
ENSG00000158062	31,29097525	20,55401196	6,549580598	0,578995131	0,00011676	UBX domain protein 11	UBXN11
ENSG00000076662	5,626264046	3,748101825	4,100134079	0,579391622	0,012701867	intercellular adhesion molec	ICAM3
ENSG00000166153	2,153276946	1,400164895	2,670340559	0,580027571	0,029795965	DEP domain containing 4	DEPDC4
ENSG00000134253	26,23782389	17,48108389	5,857108103	0,581365562	0,004187048	tripartite motif containing 4	TRIM45
ENSG00000188820	1,889461636	1,265960642	2,537850768	0,582474557	0,045047954	calcium homeostasis modul	CALHM6
ENSG00000198521	25,98585655	17,24431832	6,375809731	0,583035747	1,16E-06	zinc finger protein 43	ZNF43
ENSG00000178966	28,41376051	18,81160654	5,993446894	0,583168472	2,52E-06	RecQ mediated genome inst	RMI1
ENSG00000146263	53,34931408	35,32531434	7,078278378	0,583171828	0,000364849	MMS22 like, DNA repair pro	MMS22L
ENSG00000175567	134,0283775	89,885488	8,251974003	0,583326837	0,023789706	uncoupling protein 2	UCP2
ENSG00000065357	147,4667863	97,47438237	8,581437654	0,583651786	0,000177085	diacylglycerol kinase alpha	DGKA
ENSG00000151725	46,76395254	30,82550812	6,996121655	0,58402663	0,00635406	centromere protein U	CENPU
ENSG00000149489	6,197598173	4,163428676	3,901715133	0,584542628	0,00760079	retinal outer segment meml	ROM1
ENSG00000185104	95,6592086	63,02971611	8,021621713	0,584895854	6,76E-13	Fas associated factor 1	FAF1
ENSG00000277443	274,5626275	183,0983577	9,737238232	0,58519805	0,000111514	myristoylated alanine rich p	MARCKS
ENSG00000166813	28,16915872	18,68099848	6,329054746	0,585203733	0,004265413	kinesin family member 7	KIF7
ENSG00000160957	108,2347216	69,38728681	8,32424166	0,587133734	0,001737152	RecQ like helicase 4	RECQL4
ENSG00000101955	84,14752487	55,55504967	7,742912005	0,587480703	0,000596101	sushi repeat containing prot	SRPX
ENSG00000139190	15,11326995	9,940114746	5,471659409	0,589111674	0,036539959	vesicle associated membran	VAMP1
ENSG00000156787	13,60603354	8,682985244	5,10528338	0,589230618	0,005584544	TBC1 domain family membe	TBC1D31
ENSG00000162520	37,86950151	25,29993932	7,032354015	0,58927574	0,000212022	syncoilin, intermediate filam	SYNC
ENSG00000197747	168,0475904	111,8901904	8,777328274	0,589324697	5,11E-06	S100 calcium binding protei	S100A10
ENSG00000233426	17,68536386	11,31565543	6,076210838	0,589466553	0,002460211	eukaryotic translation initiat	EIF3FP3
ENSG00000138346	19,48665251	12,78372344	5,60242537	0,589531867	0,000128182	DNA replication helicase/nu	DNA2
ENSG00000168491	1,590606862	1,051805009	2,366005302	0,589902635	0,01895812	coiled-coil domain containin	CCDC110
ENSG00000167107	91,820807	61,18620143	8,036328097	0,590206187	0,000588554	acyl-CoA synthetase family r	ACSF2
ENSG00000187091	82,95688144	54,09189224	7,750435754	0,592618929	0,000837751	phospholipase C delta 1	PLCD1
ENSG00000099385	65,37043364	43,40186168	7,474073493	0,592680503	1,57E-05	BAF chromatin remodeling c	BCL7C
ENSG00000160949	116,4005275	75,1107832	8,327607161	0,592709752	5,28E-05	tonsoku like, DNA repair pro	TONSL
ENSG00000145833	99,4642332	66,02166313	8,011579687	0,592859015	1,74E-17	DEAD-box helicase 46	DDX46
ENSG00000187800	87,55087553	57,14644068	7,956570264	0,592905605	0,000706538	platelet endothelial aggrega	PEAR1
ENSG00000135740	9,081703435	6,038388979	4,757807397	0,594174203	0,008412727	solute carrier family 9 meml	SLC9A5
ENSG00000141480	59,5950736	38,88858393	6,9569679	0,594342	0,007238323	arrestin beta 2	ARRB2
ENSG00000132170	14,97041674	9,466215667	5,238705641	0,5949281	0,020883973	peroxisome proliferator acti	PPARG
ENSG00000159055	20,21750931	13,1160565	5,946904115	0,597455394	1,16E-05	MIS18 kinetochore protein A	MIS18A
ENSG00000115884	445,0704402	295,0442359	9,957131473	0,597907282	4,15E-07	syndecan 1	SDC1
ENSG00000183814	14,4717975	9,44863072	5,345621036	0,598938798	2,23E-05	lin-9 DREAM MuvB core con	LIN9
ENSG00000139428	26,31290123	17,00927542	6,207257522	0,599510671	0,006480698	metabolism of cobalamin as	MMAB
ENSG00000154678	25,41226082	16,94356301	6,116098615	0,599624695	0,029380824	phosphodiesterase 1C	PDE1C
ENSG00000171649	11,0159818	7,255061401	4,995737228	0,600631842	5,64E-05	zinc finger protein interactir	ZIK1
ENSG00000197935	7,534047185	4,910290911	4,176597166	0,600747142	0,034244675	zinc finger protein 311	ZNF311
ENSG00000075131	12,94839114	8,530608047	4,801948217	0,601585522	4,30E-05	TIMELESS interacting protei	TIPIN
ENSG00000167123	155,6248571	103,0580026	8,913536812	0,601937742	6,61E-06	cerebral endothelial cell adh	CERCAM
ENSG00000156140	14,96304193	9,656904863	5,144398549	0,60210928	0,001318057	ADAM metallopeptidase wit	ADAMTS3
ENSG00000118507	5,228901426	3,391572839	3,733316264	0,602211699	0,004712673	A-kinase anchoring protein 7	AKAP7
ENSG00000135723	107,6202233	70,70917434	8,276920752	0,602300783	8,86E-08	formin homology 2 domain c	FHOD1
ENSG00000105486	133,2790176	87,10259364	8,416131532	0,602382126	0,00011384	DNA ligase 1	LIG1
ENSG00000179222	291,5876385	192,3992323	9,85696114	0,603639011	4,17E-07	MAGE family member D1	MAGED1
ENSG00000204262	369,382455	243,5875988	10,08488907	0,604427316	0,002240017	collagen type V alpha 2 chai	COL5A2
ENSG00000167799	7,962937953	5,180279077	4,179254216	0,604662838	0,000870334	nudix hydrolase 8	NUDT8
ENSG00000118263	95,10123247	62,43389464	8,150461953	0,60494308	8,32E-12	Kruppel like factor 7	KLF7
ENSG00000166965	74,36365584	47,34637645	7,705809208	0,605211755	2,61E-05	RCC1 domain containing 1	RCCD1
ENSG00000117143	53,09008918	34,59205145	7,164797679	0,605297278	0,009394574	UDP-N-acetylglucosamine p	UAP1
ENSG00000184164	65,48548054	42,73714793	7,480226869	0,605440145	1,16E-05	cysteine rich with EGF like d	CRELD2
ENSG00000235863	4,817095328	3,155379277	3,71672288	0,605844328	0,030856092	beta-1,3-galactosyltransfera	B3GALT4
ENSG00000136982	20,10853594	12,80730604	5,652633896	0,606545905	0,000136187	DNA replication and sister cl	DSCC1
ENSG00000263843	1,386077763	0,90591	2,305487558	0,60707141	0,013162042	MIF4GD divergent transcript	MIF4GD-DT
ENSG00000135540	30,37843858	19,94493704	6,267063284	0,607328447	0,001516387	NHS like 1	NHSL1
ENSG00000204764	7,424600916	4,845849862	4,188679219	0,6078035	4,05E-05	RAN binding protein 17	RANBP17
ENSG00000167393	47,17352518	29,86725764	7,165410749	0,608348292	0,00385827	protein phosphatase 2 regul	PPP2R3B

ENSG00000130762	17,30913323	11,04833953	5,497221132	0,608454855	0,03501651	Rho guanine nucleotide excl	ARHGEF16
ENSG00000117519	413,7666632	271,7683299	10,18033446	0,609295406	0,000234373	calponin 3	CNN3
ENSG00000196152	28,9675102	18,53357556	6,208369558	0,609759211	0,011822005	zinc finger protein 79	ZNF79
ENSG00000247796	4,321569593	2,761634947	3,871604633	0,610293131	0,017228063	MOCS2 divergent transcript	MOCS2-DT
ENSG00000167553	240,7035831	154,2012433	9,540190378	0,611083895	0,000481053	tubulin alpha 1c	TUBA1C
ENSG00000114019	193,7464421	127,0915766	9,18985392	0,611277695	4,28E-05	angiomotin like 2	AMOTL2
ENSG00000119514	15,12380023	9,862501906	5,017493152	0,611744485	0,000879148	polypeptide N-acetylgalacto	GALNT12
ENSG00000196172	12,76968585	8,33603185	5,274280529	0,611890253	4,47E-08	zinc finger protein 681	ZNF681
ENSG00000090097	111,3370055	73,32223878	8,153936593	0,61222649	0,001371566	poly(rC) binding protein 4	PCBP4
ENSG00000119397	21,52314365	13,95816745	5,612242349	0,612692171	1,04E-05	centriolin	CNTRL
ENSG00000179588	40,67116868	26,33976854	6,603917463	0,613250115	0,015868224	zinc finger protein, FOG fam	ZFPM1
ENSG00000197020	14,59879333	9,457957566	5,524923153	0,613787438	4,55E-06	zinc finger protein 100	ZNF100
ENSG00000152377	266,9437673	174,8111565	9,713417209	0,614037008	1,26E-06	SPARC (osteonectin), cwcv a	SPOCK1
ENSG00000170684	6,532828588	4,130131565	3,885563729	0,614236881	0,005612954	zinc finger protein 296	ZNF296
ENSG00000121690	12,69240153	7,84522937	5,470286	0,614389934	0,012871814	DEP domain containing 7	DEPDC7
ENSG00000181938	24,11023204	15,63414166	5,971992799	0,614654807	0,000313711	GINS complex subunit 3	GINS3
ENSG00000100075	200,6840445	127,7175162	8,998966915	0,614700527	0,000449467	solute carrier family 25 men	SLC25A1
ENSG00000132481	43,18254745	28,24304158	6,480785207	0,614729789	3,70E-07	tripartite motif containing 4	TRIM47
ENSG00000129355	14,45764238	9,221703668	4,94688062	0,61597059	0,021066465	cyclin dependent kinase inhi	CDKN2D
ENSG00000110171	54,07745813	34,94754415	7,105936443	0,616079936	0,006370216	tripartite motif containing 3	TRIM3
ENSG00000175591	10,21589789	6,633836264	4,588039491	0,616445572	2,76E-06	purinergic receptor P2Y2	P2RY2
ENSG00000260442	6,19407907	3,895196665	3,851020687	0,617410336	0,00879401	ATP2A1 antisense RNA 1	ATP2A1-AS1
ENSG00000142627	327,8388424	213,4839905	9,841500586	0,619013529	2,93E-05	EPH receptor A2	EPHA2
ENSG00000198919	24,96588784	16,09382633	6,104520827	0,619660122	0,003733757	DAZ interacting zinc finger p	DZIP3
ENSG00000111801	20,64961237	13,41820862	6,120832097	0,619726742	0,001954688	butyrophilin subfamily 3 me	BTN3A3
ENSG00000095637	36,14835317	23,78595308	6,665265889	0,620427989	0,014303825	sorbin and SH3 domain cont	SORBS1
ENSG00000006576	33,75221985	21,94572429	6,478635174	0,622073975	0,000344996	putative homeodomain tran	PHTF2
ENSG00000099251	3,899243829	2,415835223	3,019030545	0,622220913	0,043797217	hydroxysteroid 17-beta deh	HSD17B7P2
ENSG00000172831	179,5033633	115,8790419	9,039147812	0,62257543	7,89E-07	carboxylesterase 2	CES2
ENSG00000160447	68,51904007	43,64951849	7,484706302	0,622698384	6,35E-07	protein kinase N3	PKN3
ENSG00000179528	5,215459346	3,322926802	4,056907038	0,623245948	0,019473898	ladybird homeobox 2	LBX2
ENSG00000150281	8,130380956	5,256850298	4,60424446	0,623342665	0,00370775	cardiotrophin 1	CTF1
ENSG00000183137	17,48798865	11,21471511	5,463947165	0,623422077	1,49E-05	centrosomal protein 57 like	CEP57L1
ENSG00000151692	33,55647407	21,71732408	6,21858626	0,62366226	0,001478149	ring finger protein 144A	RNF144A
ENSG00000227811	1,470116861	0,951632944	2,527737085	0,623760048	0,034047017	INKA2 antisense RNA 1	INKA2-AS1
ENSG00000042493	332,8458386	206,3966198	9,668665888	0,624344302	2,33E-05	capping actin protein, gelsol	CAPG
ENSG00000259153	3,387581768	2,19372499	3,403777669	0,624900704	0,001567219	MAP3K9 divergent transcrip	MAP3K9-DT
ENSG00000141524	38,93428345	23,96501873	6,570485435	0,625573976	0,042905034	transmembrane channel like	TMC6
ENSG00000029153	61,53149251	39,3816781	6,987230375	0,625651119	0,001644092	aryl hydrocarbon receptor n	ARNTL2
ENSG00000128596	19,44925592	12,54901092	5,528847277	0,625678822	0,001627054	coiled-coil domain containin	CCDC136
ENSG00000183010	49,76207129	32,35153888	7,355421887	0,625779347	0,000908492	pyrroline-5-carboxylate redu	PYCR1
ENSG00000151364	22,6486936	14,02312424	5,716805386	0,626615232	1,70E-06	potassium channel tetramer	KCTD14
ENSG00000124140	1,753625503	1,096410787	2,155321552	0,626760349	0,039338429	solute carrier family 12 men	SLC12A5
ENSG00000173210	149,9415682	93,00416438	8,485494446	0,626989048	0,007808243	actin binding LIM protein fa	ABLIM3
ENSG00000070950	27,0084641	17,14762508	6,216485346	0,627371452	5,95E-07	RAD18 E3 ubiquitin protein	RAD18
ENSG00000116117	25,66439175	16,69018415	6,178379977	0,627397296	0,000361037	par-3 family cell polarity reg	PARD3B
ENSG00000228486	2,184625276	1,409230324	2,695603552	0,627706109	0,039887747	chromosome 2 open reading	C2orf92
ENSG00000112312	47,05525151	29,83623007	6,634324475	0,627722092	0,00118661	geminin DNA replication inh	GMNN
ENSG00000167601	502,373739	312,6306335	10,44263998	0,627879374	0,004336614	AXL receptor tyrosine kinase	AXL
ENSG00000135045	39,99003436	25,29922046	6,60081416	0,628340029	0,000159811	chromosome 9 open reading	C9orf40
ENSG00000108515	17,01813412	11,11116818	5,199283468	0,628548147	0,022184692	enolase 3	ENO3
ENSG00000137142	3,307032347	2,13896045	3,229270999	0,628822361	0,041428572	insulin like growth factor bir	IGFBPL1
ENSG00000173706	452,9871122	288,7919816	10,23033185	0,629223134	0,017517018	heart development protein	HEG1
ENSG00000147155	55,30815433	35,231216	7,135495898	0,629773484	0,000679947	EBP cholestenol delta-isome	EBP
ENSG00000183779	57,3840271	37,25607782	7,483509078	0,629855923	0,005110018	zinc finger protein 703	ZNF703
ENSG00000053254	189,4283318	123,2925759	9,195855692	0,629863629	0,000293706	forkhead box N3	FOXN3
ENSG00000127329	88,91618654	51,16058921	7,28059418	0,62999163	0,037169754	protein tyrosine phosphatas	PTPRB
ENSG00000114405	16,4926407	10,42783903	5,50217246	0,63060585	0,001523129	chromosome 3 open reading	C3orf14
ENSG00000245648	6,000802281	3,88306574	3,86941525	0,630664393	0,045877837	KLRK1 antisense RNA 1	KLRK1-AS1
ENSG00000176428	8,395018736	5,436879163	4,618748411	0,631217038	0,002910188	VPS37D subunit of ESCRT-I	VPS37D
ENSG00000110074	40,01491126	24,89562692	6,994342808	0,631432917	0,000164325	FAD dependent oxidoreduct	FOXRED1
ENSG00000196839	11,52116341	7,402006904	5,705667928	0,631883723	0,00415769	adenosine deaminase	ADA
ENSG00000090776	216,6134813	138,967233	9,2471665	0,634001639	0,000548	ephrin B1	EFNB1
ENSG00000041353	23,72603137	15,02330325	5,956733613	0,635066825	0,040329571	RAB27B, member RAS oncog	RAB27B
ENSG00000183496	34,04856792	21,94820336	6,326715863	0,637344531	0,008553052	mex-3 RNA binding family m	MEX3B
ENSG00000186470	46,03954112	29,58302031	7,299839152	0,637737457	8,01E-05	butyrophilin subfamily 3 me	BTN3A2
ENSG00000140464	295,8610479	190,379629	9,763791167	0,638464993	7,35E-06	PML nuclear body scaffold	PML
ENSG00000188290	47,82712867	30,45214651	6,48026529	0,63866538	0,016857523	hes family bHLH transcriptio	HES4
ENSG00000145604	50,30546674	32,43488074	6,814059145	0,639428204	5,82E-06	S-phase kinase associated p	SKP2
ENSG00000173894	28,19291278	18,17445546	5,951861293	0,639848854	0,010307784	chromobox 2	CBX2
ENSG00000177990	9,499724019	5,982673355	4,542774938	0,641549026	0,004139048	dpy-19 like 2	DPY19L2
ENSG00000198830	199,6651499	125,6655172	8,853206036	0,641590529	0,001169696	high mobility group nucleos	HMGN2
ENSG00000115232	59,35629577	37,89850006	7,054021998	0,64272469	0,017187362	integrin subunit alpha 4	ITGA4
ENSG00000174125	2,513705226	1,600645692	2,364491058	0,642837829	0,005465565	toll like receptor 1	TLR1
ENSG00000136856	35,12138954	22,33273515	6,491588662	0,644107314	0,017430704	solute carrier family 2 meml	SLC2A8
ENSG00000131351	21,86415695	13,40126417	5,807578751	0,644217386	0,000527247	HAUS augmin like complex s	HAUS8
ENSG00000147010	39,7915508	25,91955083	6,834781087	0,645370063	0,043209997	SH3 domain containing kina	SH3KBP1
ENSG00000136122	8,217331045	5,103090442	4,283227001	0,645692681	0,001475586	BORA aurora kinase A activa	BORA
ENSG00000237523	3,120812564	2,00477445	2,804317175	0,645827566	0,039704966	long intergenic non-protein	LINC00857
ENSG00000143554	21,95847447	13,58018371	6,135619028	0,646545204	2,32E-07	solute carrier family 27 men	SLC27A3
ENSG00000180834	1,425919055	0,90719883	2,091335977	0,64675933	0,025914878	MAP6 domain containing 1	MAP6D1
ENSG00000141682	42,65202256	27,39893862	6,414413231	0,64778734	0,005978314	phorbol-12-myristate-13-ac	PMAIP1
ENSG00000168061	67,31380051	42,42411791	7,222937112	0,648982698	0,000708474	SAC3 domain containing 1	SAC3D1
ENSG00000162639	2,629276562	1,655374481	3,340997063	0,649093043	0,00166802	HEN methyltransferase 1	HENMT1
ENSG00000197472	5,098387184	3,067289716	4,035394884	0,649605596	0,000151616	zinc finger protein 695	ZNF695
ENSG00000196678	23,87642864	14,84186152	6,004552125	0,649989198	3,47E-06	ERI1 exoribonuclease family	ERI2
ENSG00000135835	17,53971022	10,85256981	5,946743794	0,650547211	2,05E-05	KIAA1614	KIAA1614
ENSG00000187994	32,36475185	20,25388523	5,940503442	0,651587948	0,029945949	Ras and Rab interactor like	RINL
ENSG00000178752	14,34488647	8,846054447	5,83463632	0,65259794	0,011956548	erythroferrone	ERFE
ENSG00000079435	8,060424073	5,003019293	4,07507673	0,653259003	0,004764856	lipase E, hormone sensitive	LIPE
ENSG00000159753	2,637414585	1,622922709	2,513481915	0,653351782	0,020624503	capping protein regulator ar	CARMIL2
ENSG00000203995	4,682461412	2,908599455	3,867918402	0,653465454	6,75E-06	zyg-11 family member A, cel	ZYG11A
ENSG00000173848	74,01872704	47,25083496	7,532968544	0,654783259	5,51E-11	neuroepithelial cell transfor	NET1
ENSG00000077312	107,9537084	67,50545595	8,021139118	0,655153121	8,84E-10	small nuclear ribonucleopro	SNRPA
ENSG00000198753	40,79745555	25,46710623	6,835037403	0,655924192	0,020022864	plexin B3	PLXNB3
ENSG00000227825	2,527365659	1,612117117	2,508342266	0,656635253	0,032239086	solute carrier family 9 meml	SLC9A7P1
ENSG00000166669	4,549522043	2,874050017	3,782352587	0,657212822	0,000511791	activating transcription facto	ATF7IP2
ENSG00000166508	289,2484484	182,4529969	9,471331663	0,657387369	3,42E-05	minichromosome maintenai	MCM7
ENSG00000138738	14,0016421	8,671247573	5,170160938	0,657583124	6,62E-08	PR/SET domain 5	PRDM5
ENSG00000167670	128,068153	79,38288242	8,29885346	0,658713878	6,50E-06	chromatin assembly factor 1	CHAF1A
ENSG00000107438	308,8677768	196,3266318	9,624141089	0,658715413	3,29E-07	PDZ and LIM domain 1	PDLIM1
ENSG00000204682	4,587709228	2,928630223	4,16269865	0,659377712	0,007259359	MIR1915 host gene	MIR1915HG
ENSG00000165501	16,13704484	10,07533704	5,250414238	0,659525045	0,000671617	leucine rich repeat protein 1	LRR1
ENSG00000132837	2,597619764	1,566566591	3,095475292	0,661703086	0,02329685	dimethylglycine dehydroger	DMGDH
ENSG00000103995	23,87749535	14,4721217	5,992929366	0,661920259	0,001936991	centrosomal protein 152	CEP152
ENSG00000138336	13,00899141	8,207453254	5,499838292	0,664347331	3,45E-05	tet methylcytosine dioxygen	TET1
ENSG00000101868	92,76218889	58,21450401	7,784850192	0,664494815	0,000134955	DNA polymerase alpha 1, ca	POLA1
ENSG00000166823	6,754086298	4,244815964	3,914719364	0,664795807	0,006921614	mesoderm posterior bHLH t	MESP1
ENSG00000160752	168,3473677	105,1944161	8,768506641	0,664855667	0,023696748	farnesyl diphosphate syntha	FDPS
ENSG00000106868	46,44844857	29,31183494	7,012817723	0,666243243	0,000915855	sushi domain containing 1	SUSD1
ENSG00000181381	33,21444906	20,84584739	6,61326276	0,667382407	6,95E-05	DExD/H-box 60 like	DDX60L
ENSG00000166289	25,06751818	15,38190377	5,99101025	0,667746392	0,000744174	pleckstrin homology and FYV	PLEKHF1
ENSG00000139926	96,07025932	59,89860279	8,277473009	0,667931547	0,018423428	FERM domain containing 6	FRMD6
ENSG00000119640	17,90898097	10,94628352	5,267630591	0,668216035	1,60E-05	acylphosphatase 1	ACYP1
ENSG00000159147	60,21892017	37,47684441	7,016143423	0,669099274	9,91E-06	DNA replication fork stabiliz	DONSON

ENSG00000070404	199,6289469	125,6198825	9,265888298	0,669928453	1,53E-11	follistatin like 3	FSTL3
ENSG00000166451	24,85075955	14,91442021	5,98751386	0,670050723	0,000580883	centromere protein N	CENPN
ENSG00000168394	163,46839	99,94940777	8,729410236	0,671519609	0,007464508	transporter 1, ATP binding c	TAP1
ENSG00000178922	45,11666822	27,77559904	7,010051421	0,671585196	6,82E-05	hydroxypyruvate isomerase	HYI
ENSG00000245571	7,738514863	4,812155421	4,388110932	0,672531545	9,00E-07	FAM111A divergent transcri	FAM111A-DT
ENSG00000185869	14,07308247	8,574585247	5,400607191	0,672736113	0,001113394	zinc finger protein 829	ZNF829
ENSG00000157510	52,64306102	31,6872224	7,005028835	0,673147839	0,003163197	actin filament associated pro	AFAP1L1
ENSG00000008056	4,24094278	2,642441275	3,314506715	0,6733409	0,002288982	synapsin I	SYN1
ENSG00000205181	8,88769021	5,530529213	4,938645793	0,673719572	0,037455463	long intergenic non-protein	LINC00654
ENSG00000118655	44,81372338	27,92342513	6,830832863	0,67682048	2,64E-07	DNA cross-link repair 1B	DCLRE1B
ENSG00000154146	46,36443015	28,85827409	6,683072033	0,677073334	0,019231768	neurogranin	NRGN
ENSG00000119943	17,03168311	10,49896078	6,14897163	0,677658571	0,004860495	pyridine nucleotide-disulphi	PYROXD2
ENSG00000100097	233,2712704	147,0093655	9,302993865	0,678310477	0,001500076	galectin 1	LGALS1
ENSG00000100065	121,267261	75,25358001	8,493585624	0,678696016	0,000764179	caspase recruitment domain	CARD10
ENSG00000120802	233,9128227	143,4818832	8,874373591	0,678704741	2,93E-05	thymopoietin	TMPO
ENSG00000177465	4,176992052	2,531178368	3,446421292	0,679525934	0,013486533	acyl-CoA thioesterase 4	ACOT4
ENSG00000123416	871,6896791	535,0355004	11,23289385	0,679762036	7,58E-13	tubulin alpha 1b	TUBA1B
ENSG00000008517	42,06835206	24,78452971	8,152366545	0,680016178	0,002745701	interleukin 32	IL32
ENSG00000101311	9,70408426	6,093074177	4,429110556	0,680516776	0,001485578	FERM domain containing kir	FERMT1
ENSG00000126705	111,2897399	69,43398429	8,187758052	0,680855326	4,72E-13	AT-hook DNA binding motif	AHDC1
ENSG00000146143	34,51658469	21,52709584	6,493735491	0,681516049	2,30E-05	DNA primase subunit 2	PRIM2
ENSG00000171813	26,05762911	15,88804166	6,173983773	0,681702919	8,47E-05	PWWP domain containing 2	PWWP2B
ENSG00000221890	46,68922015	28,94024363	6,934217694	0,682321626	0,025280724	neuronal pentraxin receptor	NPTXR
ENSG00000103490	31,52678906	18,8801547	6,313630078	0,682834586	0,000488164	PYD and CARD domain conta	PYCARD
ENSG00000123933	296,2631052	178,6787385	9,742073477	0,683254482	0,025911144	MAX dimerization protein 4	MXD4
ENSG00000197044	15,07570137	9,130372119	5,374498483	0,683282117	0,000177413	zinc finger protein 441	ZNF441
ENSG00000176619	377,5281753	232,3408691	9,728827509	0,684446381	7,33E-06	lamin B2	LMNB2
ENSG00000186364	3,942520423	2,449873744	3,573748353	0,685232086	0,03038062	nudix hydrolase 17	NUDT17
ENSG00000100206	9,219636408	5,768950019	4,00452585	0,685645263	0,009525133	DNA meiotic recombinase 1	DMC1
ENSG00000129675	17,51194668	10,75949584	5,369134028	0,686237144	1,43E-07	Rac/Cdc42 guanine nucleoti	ARHGEF6
ENSG00000156869	5,779492902	3,593765135	4,0283993	0,687004993	0,036423096	ferric chelate reductase 1	FRRS1
ENSG00000065534	635,9201091	396,77057	10,9330145	0,687073811	4,98E-07	myosin light chain kinase	MYLK
ENSG00000125898	14,98624635	9,24933859	5,253359603	0,687513567	0,001223542	family with sequence similar	FAM110A
ENSG00000182197	194,6151907	120,0590292	9,135589981	0,688455174	1,78E-14	exostosin glycosyltransferas	EXT1
ENSG00000152455	18,24490607	11,12491555	5,555556769	0,688755703	1,09E-05	SUV39H2 histone lysine met	SUV39H2
ENSG00000087077	206,6143766	126,3085244	9,170523267	0,689401823	9,99E-06	thyroid hormone receptor ir	TRIP6
ENSG00000042088	34,26897031	21,06685159	6,614814417	0,689894815	0,000132683	tyrosyl-DNA phosphodiester	TDP1
ENSG00000151967	4,294052269	2,688972301	3,657612801	0,691107808	0,007498112	schwannomin interacting pr	SCHIP1
ENSG00000161513	221,2992395	133,5381492	9,135045054	0,691930832	0,000635203	ferredoxin reductase	FDXR
ENSG00000183684	67,86253957	41,65734335	7,36114248	0,694724488	4,11E-08	Aly/REF export factor	ALYREF
ENSG00000242715	2,849982279	1,766928973	2,664584857	0,694766515	0,004918054	coiled-coil domain containin	CCDC169
ENSG00000128944	26,58540296	15,8458325	6,143750246	0,695172406	0,000575751	kinetochore localized astrin	KNSTRN
ENSG00000203805	35,84167581	22,33919203	6,842892656	0,695511541	0,001934881	phospholipid phosphatase 4	PLPP4
ENSG00000182179	61,49209848	37,64082768	7,487645321	0,695970938	4,93E-06	ubiquitin like modifier activa	UBA7
ENSG00000278709	7,21407805	4,462834041	4,705924461	0,696540959	0,005480968	NF-kappaB interacting lncRN	NKILA
ENSG00000151470	10,98585699	6,603618071	5,237321575	0,697170085	3,14E-05	chromosome 4 open reading	C4orf33
ENSG00000156876	17,43083981	10,47720355	5,648662054	0,700492803	6,61E-06	SAS-6 centriolar assembly pr	SASS6
ENSG00000269313	3,404526797	2,069815414	3,34640553	0,700649138	1,08E-05	MAGI family member, X-link	MAGIX
ENSG00000266865	2,829561007	1,745312208	3,054859365	0,700845065	0,006857596	SMAD specific E3 ubiquitin p	LOC107984974
ENSG00000095380	145,6116554	85,05628301	8,461996087	0,70440505	0,000814998	N-acetylneuraminate syntha	NANS
ENSG00000188878	21,81227118	13,01377786	5,76954412	0,70461992	2,55E-05	Fas binding factor 1	FBF1
ENSG00000114268	63,53778956	39,2018608	7,633542161	0,704794857	0,001832277	6-phosphofructo-2-kinase/fr	PFKFB4
ENSG00000167216	4,975136948	2,87521485	4,235680705	0,706763964	0,001440448	katanin catalytic subunit A1	KATNAL2
ENSG00000070614	314,1770556	193,5465648	9,79560402	0,706996177	6,73E-06	N-deacetylase and N-sulfotr	NDST1
ENSG00000176225	34,92290053	20,92478732	6,514260153	0,707056198	1,10E-05	rotatin	RTTN
ENSG00000136943	38,22446353	22,54110538	6,755877782	0,707397587	0,008957196	cathepsin V	CTSV
ENSG00000103811	80,4268337	49,39268921	7,750630304	0,70755447	0,000403226	cathepsin H	CTSH
ENSG00000157833	28,51017949	17,33394468	6,030981661	0,708248081	0,0218944	GRB2 associated regulator o	GAREM2
ENSG00000184254	50,78848666	31,99508212	7,229076483	0,709466361	0,033539976	aldehyde dehydrogenase 1 f	ALDH1A3
ENSG00000153029	68,07202027	41,30339021	7,542334904	0,709837764	4,31E-07	major histocompatibility cor	MR1
ENSG00000120915	2,984630207	1,721859193	3,559637441	0,709891697	0,001477364	epoxide hydrolase 2	EPHX2
ENSG00000167325	235,5780124	143,009957	9,102764616	0,710947841	1,65E-05	ribonucleotide reductase ca	RRM1
ENSG00000187688	53,17454817	32,57220273	7,159735618	0,711120501	0,003483428	transient receptor potential	TRPV2
ENSG00000074047	32,06576262	19,28040201	6,250790528	0,712545561	0,04275328	GLI family zinc finger 2	GLI2
ENSG00000132436	35,99003858	21,70577708	6,523969187	0,71284444	2,22E-05	fidgetin like 1	FIGNL1
ENSG00000198885	9,108710018	5,486480513	4,072923263	0,713003128	0,013240824	ITPRIP like 1	ITPRIPL1
ENSG00000163171	108,4202205	66,9912083	8,181991929	0,71353114	0,001636274	CDC42 effector protein 3	CDC42EP3
ENSG00000187624	8,410843887	5,04663899	4,9465698	0,714214302	0,001754543	chromosome 17 open readi	C17orf97
ENSG00000176909	2,780486165	1,655784481	3,498414011	0,714673797	0,000453451	MEF2 activating motif and S	MAMSTR
ENSG00000171631	24,00829082	14,82720061	6,106550947	0,714876889	0,00158994	pyrimidinergic receptor P2Y	P2RY6
ENSG00000229852	5,718922166	3,460873546	4,073857268	0,715274152	0,001522312	RPSAP41-EIF3EP1	LOC122539212
ENSG00000178078	21,10877708	12,72836611	5,969383167	0,71635569	6,79E-08	signal transducing adaptor f	STAP2
ENSG00000172508	7,210836655	4,401428319	4,014906637	0,717051303	0,043895442	carnosine synthase 1	CARNS1
ENSG00000101353	1,501442035	0,906764997	1,856877225	0,717195813	0,036832988	maestro heat like repeat fan	MROH8
ENSG00000171208	113,0712357	68,56666674	7,985985094	0,717197504	1,26E-05	neuropilin and tolloid like 2	NETO2
ENSG00000164100	3,098590679	1,899980309	3,07294453	0,717629735	0,032951307	N-deacetylase and N-sulfotr	NDST3
ENSG00000172345	8,175400104	4,939035911	4,396134175	0,718114073	0,013564817	StAR related lipid transfer do	STARD5
ENSG00000047634	17,74095809	10,65414309	5,474415282	0,718598749	0,000675658	Scm polycomb group protein	SCML1
ENSG00000178295	32,9367356	19,21591558	6,54483647	0,719057666	1,26E-05	GEN1 Holliday junction 5' fla	GEN1
ENSG00000070814	221,7716419	131,7808253	9,186222051	0,71909834	5,01E-05	treacle ribosome biogenesis	TCOF1
ENSG00000204856	11,78516667	7,049441277	5,042250838	0,719850763	2,09E-05	family with sequence similar	FAM216A
ENSG00000109576	8,982958491	5,467982521	4,554172867	0,720043995	0,010098903	aminoadipate aminotransfe	AADAT
ENSG00000205309	5,230538666	3,024756264	3,541302981	0,720083854	0,031472758	5',3'-nucleotidase, mitochor	NT5M
ENSG00000163637	61,93718796	37,67876017	7,6568278	0,72062012	3,18E-06	prickle planar cell polarity pr	PRICKLE2
ENSG00000184635	10,4374622	6,244939676	5,174810745	0,722489084	0,002153083	zinc finger protein 93	ZNF93
ENSG00000104368	498,6579806	301,7894964	10,5086769	0,722631129	0,00107053	plasminogen activator, tissu	PLAT
ENSG00000237624	1,500172362	0,906135666	2,319134534	0,72285548	0,026562729	3-oxoacid CoA-transferase 2	OXCT2P1
ENSG00000184163	2,171087631	1,320838015	2,445727695	0,72320777	0,029841199	C1q and TNF related 12	C1QTNF12
ENSG00000125726	45,44472297	26,28398368	6,60775606	0,723484373	0,004780197	CD70 molecule	CD70
ENSG00000141510	122,7142527	74,22570383	8,337400127	0,724013853	4,75E-06	tumor protein p53	TP53
ENSG00000176845	36,06375008	21,36742257	6,610722556	0,724627262	0,000206271	meteorin like, glial cell differ	METRNL
ENSG00000188868	4,194542087	2,541028047	3,330061826	0,725487168	0,008741926	zinc finger protein 563	ZNF563
ENSG00000170734	131,8318981	78,58654026	8,381448184	0,725532298	1,68E-07	DNA polymerase eta	POLH
ENSG00000186314	8,193203943	4,958537512	4,416515851	0,725876795	2,98E-06	PRELI domain containing 2	PRELID2
ENSG00000125378	28,23615242	17,10874029	6,171895947	0,726678555	0,047609946	bone morphogenetic protein	BMP4
ENSG00000176912	4,818127333	2,934659544	3,740331045	0,726816872	0,000113085	TYMS opposite strand RNA	TYMSOS
ENSG00000140993	5,862057743	3,548380691	4,130863369	0,726890107	1,01E-06	tigger transposable element	TIGD7
ENSG00000214391	1,03439443	0,604478471	1,647452738	0,729284356	0,018840397	tubulin alpha pseudogene 2	TUBAP2
ENSG00000137310	203,0265283	119,2574656	8,774451696	0,730410399	0,00275565	transcription factor 19	TCF19
ENSG00000099822	2,664654784	1,477889032	3,473253704	0,731331816	0,029917458	hyperpolarization activated	HCN2
ENSG00000126803	84,45193443	52,07168401	7,62191671	0,731884912	0,002717809	heat shock protein family A	HSPA2
ENSG00000162063	62,3601137	36,35118884	7,060763752	0,73201635	0,000330799	cyclin F	CCNF
ENSG00000012048	80,376763	46,52592636	7,616549219	0,733007899	5,66E-06	BRCA1 DNA repair associate	BRCA1
ENSG00000166801	111,7337032	64,53443763	8,043132447	0,733344204	1,56E-05	FAM111 trypsin like peptida	FAM111A
ENSG00000151503	124,8826813	72,72749051	8,261855831	0,734607968	1,60E-08	non-SMC condensin II comp	NCAPD3
ENSG00000095397	36,78366697	21,76984692	6,442197408	0,735518967	0,017494617	whirlin	WHRN
ENSG00000182185	8,803468902	5,284437553	4,578451759	0,735754438	1,10E-05	RAD51 paralog B	RAD51B
ENSG00000139174	55,46119159	33,68406436	7,215196755	0,735881693	1,10E-05	prickle planar cell polarity pr	PRICKLE1
ENSG00000013573	105,4742669	61,41680299	8,133909141	0,735984569	7,11E-06	DEAD/H-box helicase 11	DDX11
ENSG00000128591	475,2243493	286,3647436	10,51667568	0,73618661	2,97E-06	filamin C	FLNC
ENSG00000168496	168,9008105	100,5032951	8,581579191	0,737105403	0,000134042	flap structure-specific endor	FEN1
ENSG00000129484	62,62504648	37,06903908	7,326138088	0,73812203	4,11E-08	poly(ADP-ribose) polymeras	PARP2
ENSG00000196247	24,06189992	14,14291859	6,172523013	0,739579034	5,72E-10	zinc finger protein 107	ZNF107
ENSG00000197977	10,58986744	6,419335767	4,566355438	0,740628258	0,030650899	ELOVL fatty acid elongase 2	ELOVL2
ENSG00000100196	61,78345027	37,04495673	7,450114547	0,741325349	0,00080247	KDEL endoplasmic reticulum	KDELR3

ENSG00000213347	20,66466444	12,06225775	5,50314084	0,741386205	0,002327445	MAX dimerization protein 3	MXD3
ENSG00000170153	39,51125494	23,84435917	6,709064846	0,74162236	0,004497459	ring finger protein 150	RNF150
ENSG00000132623	8,179640257	4,840098704	4,622520414	0,74245849	0,008057626	ankyrin repeat and EF-hand	ANKEF1
ENSG00000100139	276,6681136	164,3064653	9,465357652	0,742664204	0,000117369	MICAL like 1	MICALL1
ENSG00000237515	140,9210534	80,20237437	8,506713184	0,743111894	2,03E-06	shisa family member 9	SHISA9
ENSG00000149257	387,2376232	231,7600399	10,09052492	0,74400694	2,23E-26	serpin family H member 1	SERPINH1
ENSG00000101945	22,55550298	13,18435921	5,92467119	0,746566181	3,82E-05	SUV39H1 histone lysine met	SUV39H1
ENSG00000213760	2,500284178	1,488480121	3,151224329	0,747141782	0,001169649	ATPase H+ transporting V1 s	ATP6V1G2
ENSG00000183780	3,230752759	1,879634875	3,173009793	0,747807331	0,030435801	solute carrier family 35 men	SLC35F3
ENSG00000160145	6,69872489	3,97957873	4,698778202	0,748526287	0,001416987	kalirin RhoGEF kinase	KALRN
ENSG00000165244	49,76018102	29,43966414	6,607949249	0,749044853	0,002480688	zinc finger protein 367	ZNF367
ENSG00000111863	0,973051765	0,570606784	1,643314678	0,75069768	0,035005678	androgen dependent TFPI re	ADTRP
ENSG00000183963	203,0891832	117,2748979	9,07130844	0,751537592	7,07E-07	smoothelin	SMTN
ENSG00000146904	3,537760684	2,103206354	3,047520729	0,751758685	0,009413105	EPH receptor A1	EPHA1
ENSG00000117650	26,58751658	14,44641761	5,711895672	0,752536071	0,001120629	NIMA related kinase 2	NEK2
ENSG00000005243	10,41391918	6,166847625	4,852568926	0,754067627	1,56E-05	COPI coat complex subunit z	COPZ2
ENSG00000129534	49,0308312	27,93240943	7,136394086	0,756185614	2,90E-11	MIS18 binding protein 1	MIS18BP1
ENSG00000160229	2,219912827	1,310323255	2,912424921	0,756622139	0,001799607	zinc finger protein 66	ZNF66
ENSG00000180998	12,25006828	7,267368065	4,816356448	0,756996784	0,000455538	G protein-coupled receptor	GPR137C
ENSG00000135821	475,8945288	270,4353078	9,922210618	0,757732534	0,020709963	glutamate-ammonia ligase	GLUL
ENSG00000091592	47,28051513	27,69312168	6,889094474	0,758526276	1,11E-05	NLR family pyrin domain cor	NLRP1
ENSG00000014138	77,82507897	45,35225544	7,437537468	0,758611108	4,07E-06	DNA polymerase alpha 2, ac	POLA2
ENSG00000146038	198,6828155	117,8912645	8,795132619	0,759098959	3,77E-05	doublecortin domain contai	DCDC2
ENSG00000110492	279,8408213	164,9511845	9,283107669	0,759428438	6,98E-05	midkine	MDK
ENSG00000196502	9,636816033	5,451313661	4,64137007	0,76078319	0,03006368	sulfotransferase family 1A m	SULT1A1
ENSG00000149485	384,6519002	222,7603738	9,824231224	0,761668102	0,000105932	fatty acid desaturase 1	FADS1
ENSG00000136153	85,44709894	50,64185138	7,550425256	0,76171574	6,48E-05	LIM domain 7	LMO7
ENSG00000179583	9,86827394	5,881953088	4,751848219	0,76394351	0,02890468	class II major histocompatib	CIITA
ENSG00000134245	7,986229123	4,69719286	4,676939361	0,76515533	0,000261463	Wnt family member 2B	WNT2B
ENSG00000244694	30,14709146	17,75516427	6,810377673	0,766000552	0,000950494	patched domain containing	PTCHD4
ENSG00000108622	6,384208109	3,258960918	4,037007778	0,766642231	0,000320868	intercellular adhesion molec	ICAM2
ENSG00000181007	6,272196945	3,660279263	4,21585678	0,766976435	2,26E-07	ZFP82 zinc finger protein	ZFP82
ENSG00000184205	40,34837605	23,08670078	6,863686874	0,767936563	0,00330938	TSPY like 2	TSPYL2
ENSG00000132749	2,043726649	1,197159687	2,94816703	0,768754211	0,00034456	testis expressed metallothio	TESMIN
ENSG00000143429	5,211338594	3,000341756	3,982843286	0,769112592	2,70E-05	LSP1 pseudogene 5	LSP1P5
ENSG00000273604	23,21060837	13,67642892	5,887642238	0,770498057	0,025137698	elongin BC and polycomb re	EPOP
ENSG00000151849	33,95325562	18,98346047	6,654236785	0,772274587	3,10E-05	centromere protein J	CENPJ
ENSG00000171161	91,0614675	52,19764104	7,679751202	0,773258816	2,61E-08	zinc finger protein 672	ZNF672
ENSG00000132613	341,1856716	189,1790596	9,747976008	0,774315834	0,000356741	MTSS I-BAR domain contain	MTSS2
ENSG00000146530	2,259005168	1,298715916	2,984487148	0,774472346	0,005713857	von Willebrand factor D and	VWDE
ENSG00000093217	6,810715675	3,952906779	4,005016818	0,774483837	0,000914691	xylulokinase	XYLB
ENSG00000129474	196,4143176	113,8896248	9,175685799	0,774557632	2,49E-08	ajuba LIM protein	AJUBA
ENSG00000120334	15,00845699	8,585236673	5,301778981	0,776454806	1,77E-08	centromere protein L	CENPL
ENSG00000091972	16,08683959	9,39488243	5,07096701	0,776533886	0,003970857	CD200 molecule	CD200
ENSG00000138092	57,46054869	32,45282655	7,364346833	0,778552775	1,12E-05	centromere protein O	CENPO
ENSG00000163888	9,032878157	5,246917201	4,171445056	0,779005711	0,031151193	calcium/calmodulin depend	CAMK2N2
ENSG00000187951	14,14237718	7,462123797	5,075899007	0,77902462	0,007383789	OTU deubiquitinase 7A pseu	LOC100288637
ENSG00000110092	234,3687951	143,5498018	9,682698971	0,77994744	0,026674665	cyclin D1	CCND1
ENSG00000237594	2,335137805	1,366708789	2,596284874	0,781291355	0,003406532	TIAM1 antisense RNA 1	TIAM1-AS1
ENSG00000101057	202,1367546	113,9430617	8,886310063	0,782490914	0,001080224	MYB proto-oncogene like 2	MYBL2
ENSG00000135905	24,03192814	14,03699608	5,443522508	0,783539442	0,030777087	dedicator of cytokinesis 10	DOCK10
ENSG00000111344	42,21239881	24,03221535	6,774459785	0,783557718	0,011022769	RAS protein activator like 1	RASAL1
ENSG00000179841	18,8493635	10,96982403	5,523050713	0,784126314	0,004993743	A-kinase anchoring protein !	AKAP5
ENSG00000198554	86,34052976	49,6042473	7,628575084	0,785869083	1,68E-06	WD repeat and HMG-box DI	WDHD1
ENSG00000236914	1,214223111	0,705488034	1,871217808	0,7858998	0,008716824	long intergenic non-protein	LINC01852
ENSG00000238142	4,119370013	2,372987105	3,648566411	0,785979353	6,09E-06	uncharacterized LOC105376	LOC105376805
ENSG00000236155	0,833711849	0,469614719	1,268856506	0,786140944	0,024926458	zona pellucida like domain c	ZPLD2P
ENSG00000115687	52,17570327	29,51648639	6,789024584	0,786871761	4,76E-05	PAS domain containing serir	PASK
ENSG00000142621	1,989921391	1,140494485	1,517218469	0,788205578	0,025460786	forkhead associated phosph	FHAD1
ENSG00000241313	0,853247821	0,492704648	1,430349477	0,789135123	0,020779233	WWTR1 antisense RNA 1	WWTR1-AS1
ENSG00000205208	27,19883125	15,68005377	6,117139053	0,789450635	7,51E-05	chromosome 4 open readin	C4orf46
ENSG00000112394	3,156692936	1,812110832	2,816861426	0,79070989	0,029309008	solute carrier family 16 men	SLC16A10
ENSG00000163935	15,92686715	9,126390508	5,235617893	0,792017729	7,09E-06	Scm like with four mbt dom	SFMBT1
ENSG00000175274	212,1434072	117,161259	9,055439767	0,792436376	0,000600055	tumor protein p53 inducible	TP53I11
ENSG00000081189	6,474517979	3,493159237	3,575338459	0,793459867	0,022064971	myocyte enhancer factor 2C	MEF2C
ENSG00000095203	5,76209732	3,079455639	4,012785349	0,793473653	0,049294983	erythrocyte membrane prot	EPB41L4B
ENSG00000189057	104,0723048	60,67224765	7,445440212	0,794292149	0,003844125	FAM111 trypsin like peptida	FAM111B
ENSG00000100749	22,60872363	12,78483427	5,8816983	0,796156897	4,13E-07	VRK serine/threonine kinase	VRK1
ENSG00000180340	49,26289501	28,27181596	7,326798946	0,79839664	1,20E-09	frizzled class receptor 2	FZD2
ENSG00000168060	1,827460399	1,006753481	3,081819857	0,798654707	0,008925025	N-acetylated alpha-linked ac	NAALADL1
ENSG00000256124	1,728973037	0,984964299	2,342934872	0,800573356	0,00854296	long intergenic non-protein	LINC01152
ENSG00000161921	11,937914	6,828358459	4,729577671	0,800731063	0,000376556	C-X-C motif chemokine ligan	CXCL16
ENSG00000120539	50,4934947	27,13335956	6,988322185	0,802049821	7,75E-09	microtubule associated serir	MASTL
ENSG00000104549	91,12523223	50,44146601	7,967676028	0,802488215	0,017154398	squalene epoxidase	SQLE
ENSG00000123219	25,85381528	14,54349248	5,865331866	0,802552717	0,000175892	centromere protein K	CENPK
ENSG00000031691	16,02864409	8,679277719	5,535047947	0,802765491	0,001777922	centromere protein Q	CENPQ
ENSG00000248019	0,967268004	0,548306686	1,53263605	0,804591067	0,028119662	FAM13A antisense RNA 1	FAM13A-AS1
ENSG00000121753	56,62802863	33,25034771	7,223841252	0,804736634	0,019758518	adhesion G protein-coupled	ADGRB2
ENSG00000249859	16,48125244	9,095674098	5,562538812	0,804873928	0,003284616	Pvt1 oncogene	PVT1
ENSG00000154102	4,119780514	2,238800352	3,159581117	0,804891834	0,022029187	chromosome 16 open readi	C16orf74
ENSG00000228649	1,130387926	0,615818812	1,918767043	0,804962124	0,037934572	small nucleolar RNA host ge	SNHG26
ENSG00000163923	34,07088532	18,756575	6,476641629	0,805069017	1,33E-06	ribosomal protein L39 like	RPL39L
ENSG00000142856	21,57904808	11,84840384	5,651901353	0,806048763	0,000615349	integrin subunit beta 3 bindi	ITGB3BP
ENSG00000183775	2,309494841	1,320160264	2,767423317	0,806603562	0,000655973	potassium channel tetramer	KCTD16
ENSG00000170425	25,54619052	14,60207176	6,031410662	0,806804627	6,33E-06	adenosine A2b receptor	ADORA2B
ENSG00000132423	4,37702429	2,452314737	3,612131204	0,807109861	0,000518581	coenzyme Q3, methyltransf	COQ3
ENSG00000172167	12,58513408	6,976071465	4,951138212	0,807344561	2,27E-05	MDM2 binding protein	MTBP
ENSG00000170835	8,722306261	4,994026117	4,29214827	0,810302179	2,76E-05	carboxyl ester lipase	CEL
ENSG00000259330	40,03760889	22,82902737	6,659665443	0,811514819	6,25E-06	InaF motif containing 2	INAFM2
ENSG00000175305	44,88183647	25,66330289	6,327051239	0,811603913	0,012158	cyclin E2	CCNE2
ENSG00000004399	170,8847151	97,37472608	8,833034495	0,811828567	3,80E-08	plexin D1	PLXND1
ENSG00000140853	39,8493471	21,62135039	6,981439271	0,812053852	1,76E-05	NLR family CARD domain co	NLRC5
ENSG00000136783	17,37386372	9,554428471	5,816614425	0,814193489	5,24E-05	nipsnap homolog 3A	NIPSNAP3A
ENSG00000188486	164,6532025	91,20561925	8,352697365	0,814716481	0,000931772	H2A.X variant histone	H2AX
ENSG00000150687	166,415168	90,72953486	8,633949509	0,816400588	0,002740832	serine protease 23	PRSS23
ENSG00000172123	28,38985654	15,92009532	6,15305207	0,817093784	1,99E-05	schlafen family member 12	SLFN12
ENSG00000137203	63,22577856	33,20825973	6,395510228	0,817406929	0,011741691	transcription factor AP-2 alp	TFAP2A
ENSG00000168209	472,7102731	267,6965856	10,46389628	0,817486039	0,003978613	DNA damage inducible trans	DDIT4
ENSG00000010292	230,1955661	126,4340889	9,009749152	0,81843035	9,10E-06	non-SMC condensin I compl	NCAPD2
ENSG00000183850	5,944696915	3,404101094	3,757865462	0,818630478	0,01984357	zinc finger protein 730	ZNF730
ENSG00000237172	88,85684805	50,17164487	7,925979023	0,819348124	7,44E-08	UDP-GlcNAc:betaGal beta-1	B3GNT9
ENSG00000116962	310,8202284	180,0938579	9,59527651	0,819552912	0,048528515	nidogen 1	NID1
ENSG00000136108	116,4095812	63,62916881	8,110075189	0,820117773	6,42E-06	cytoskeleton associated pro	CKAP2
ENSG00000131944	6,647953903	3,651374333	4,373831197	0,820662266	0,001183116	FA core complex associated	FAAP24
ENSG00000162616	45,35634435	26,15248971	7,407245232	0,822833189	0,028944799	DnaJ heat shock protein fan	DNAJB4
ENSG00000162068	2,004038573	1,120195965	1,938095318	0,823381808	0,0056251	netrin 3	NTN3
ENSG00000099256	19,42589662	10,85731438	5,446853598	0,823906835	2,96E-08	phosphoribosyl transferase	PRTFDC1
ENSG00000175592	112,6232736	61,58660852	7,89729137	0,824129679	0,001728188	FOS like 1, AP-1 transcriptio	FOSL1
ENSG00000136824	135,571733	74,15376093	8,462397695	0,824152729	1,72E-12	structural maintenance of cl	SMC2
ENSG00000250510	10,63824291	5,850138517	4,62315221	0,824519523	0,021945022	G protein-coupled receptor	GPR162
ENSG00000125864	1,216633132	0,68312277	1,937515936	0,82502191	0,009314425	beaded filament structural p	BFSP1
ENSG00000188707	18,66119043	9,95789773	4,949671829	0,825409318	0,006877669	zinc finger BED-type contain	ZBED10P
ENSG00000184226	11,31719172	6,325417965	5,622768331	0,825946565	0,020840867	protocadherin 9	PCDH9
ENSG00000102312	20,17450316	11,40078041	5,82927645	0,826367901	0,000203301	porcupine O-acyltransferase	PORCN
ENSG00000164045	32,86652182	18,06117347	6,134150588	0,826430184	5,87E-05	cell division cycle 25A	CDC25A

ENSG00000069424	26,26634711	13,70990177	5,920686268	0,826571505	0,004686222	potassium voltage-gated cha	KCNAB2
ENSG00000126001	194,5196866	110,0034592	8,945475943	0,826576976	0,000292219	centrosomal protein 250	CEP250
ENSG00000122786	350,7836162	198,7080581	10,03085269	0,829170821	8,08E-07	caldesmon 1	CALD1
ENSG00000114805	5,502264375	3,10950775	3,58218609	0,83322885	0,014226387	phospholipase C eta 1	PLCH1
ENSG00000196584	21,59420003	11,74317461	5,98273025	0,833291765	1,62E-07	X-ray repair cross compleme	XRCC2
ENSG00000175183	64,36811301	35,89259725	7,266773994	0,833413031	2,90E-12	cysteine and glycine rich pro	CSRP2
ENSG00000124813	127,2154429	71,28313369	8,797116898	0,834556767	1,55E-11	RUNX family transcription fa	RUNX2
ENSG00000005379	22,74757982	12,7142612	6,118951709	0,837297314	5,95E-05	TSPO associated protein 1	TSPOAP1
ENSG00000139880	82,32443362	45,45136155	7,504056317	0,8376396	5,07E-06	cadherin 24	CDH24
ENSG00000273274	2,26359951	1,253580971	2,835478842	0,838191653	0,00087133	zinc finger and BTB domain c	ZBTB8B
ENSG00000170955	25,58437404	14,03162068	6,040914778	0,839745173	0,005637365	caveolae associated protein	CAVIN3
ENSG00000127528	5,3319527	2,977185908	3,442731358	0,840599629	0,039887747	Kruppel like factor 2	KLF2
ENSG00000116396	5,647266244	2,996635013	4,137618127	0,840862768	0,011909554	potassium voltage-gated cha	KCNC4
ENSG00000257108	1,04195126	0,570660033	0,985600457	0,84193796	0,016359325	NHL repeat containing 4	NHLRC4
ENSG00000100298	0,988279656	0,548835101	1,392747874	0,842117352	0,034636943	apolipoprotein B mRNA edit	APOBEC3H
ENSG00000172893	85,53044852	46,47058404	8,023202541	0,842229388	0,031097989	7-dehydrocholesterol reduct	DHCR7
ENSG00000106462	52,40425249	28,70434564	6,656931378	0,842612386	9,58E-06	enhancer of zeste 2 polycom	EZH2
ENSG00000125872	100,1107199	57,6845561	8,36102132	0,842946649	0,046893301	leucine rich repeat neuronal	LRRN4
ENSG00000172687	12,25499461	6,736658996	5,121219211	0,844621511	1,39E-07	zinc finger protein 738	ZNF738
ENSG00000164082	2,198974425	1,209758689	1,7421194	0,844798459	0,010873343	glutamate metabotropic rec	GRM2
ENSG00000165304	89,76600964	47,71361062	7,720324094	0,845191139	8,25E-06	maternal embryonic leucine	MELK
ENSG00000091651	42,12870981	22,27721728	6,780306688	0,846133082	1,09E-05	origin recognition complex s	ORC6
ENSG00000109084	114,1577398	62,23996763	8,097078305	0,846582703	0,000676327	transmembrane protein 97	TMEM97
ENSG00000004838	2,291807124	1,198264183	2,296862804	0,846852959	0,01895812	zinc finger MYND-type conta	ZMYND10
ENSG00000176438	12,9281706	7,154662917	4,383343639	0,847103287	0,010029658	spectrin repeat containing n	SYNE3
ENSG00000171877	24,26550891	13,61548017	6,1826676	0,84735277	0,003421863	FERM domain containing 5	FRMD5
ENSG00000187123	20,99882198	11,28383223	5,250819679	0,847667251	8,34E-07	LY6/PLAUR domain containi	LYPD6
ENSG00000100479	12,00630488	6,619953231	4,692620394	0,849414355	4,69E-05	DNA polymerase epsilon 2, a	POLE2
ENSG00000213420	9,592866124	5,354921375	4,385540159	0,84965088	0,001211386	glypican 2	GPC2
ENSG00000100558	19,72052452	10,85310155	5,486403118	0,850778741	1,03E-06	pleckstrin 2	PLEK2
ENSG00000090554	0,634462259	0,346857306	0,904265643	0,85160565	0,043632528	fms related receptor tyrosin	FLT3LG
ENSG00000155367	5,452434525	2,941853728	3,882407079	0,852940756	0,009283728	protein phosphatase, Mg2+/	PPM1J
ENSG00000189212	9,137860761	4,923715495	4,545441523	0,853085651	2,71E-06	DPY19L2 pseudogene 1	DPY19L2P1
ENSG00000131019	4,932856825	2,706632824	3,443745191	0,85320941	8,70E-06	UL16 binding protein 3	ULBP3
ENSG00000072163	8,263687605	4,45641219	5,183578552	0,853874005	7,71E-05	LIM zinc finger domain conta	LIMS2
ENSG00000125845	14,89876916	8,402255146	5,685631531	0,853973119	0,045735704	bone morphogenetic protein	BMP2
ENSG00000197124	5,584208034	3,044965785	4,116525276	0,854872748	1,14E-09	zinc finger protein 682	ZNF682
ENSG00000182481	161,2049933	87,36213828	8,489099218	0,855739572	9,16E-05	karyopherin subunit alpha 2	KPNA2
ENSG00000164611	44,19886429	23,67003869	6,516373475	0,858644066	0,000167024	PTTG1 regulator of sister chr	PTTG1
ENSG00000267041	8,93408294	4,859784608	4,55735212	0,858662964	1,67E-07	zinc finger protein 850	ZNF850
ENSG00000234028	1,352086451	0,73873039	2,094450255	0,859032852	0,006725316	EIF2AK3 divergent transcript	EIF2AK3-DT
ENSG00000052802	81,74296575	43,74699479	7,790038799	0,859659872	0,02565634	methylsterol monooxygenase	MSMO1
ENSG00000151014	13,87362044	7,660733347	5,114454652	0,860713846	1,31E-06	nocturnin	NOCT
ENSG00000100162	29,2891445	15,56981978	6,0720132	0,861281097	0,001371566	centromere protein M	CENPM
ENSG00000197444	15,1716397	8,161887518	6,69864393	0,862288627	0,000566853	oxoglutarate dehydrogenase	OGDHL
ENSG00000103034	38,74757741	21,21506438	7,040283009	0,862712378	3,10E-05	NDRG family member 4	NDRG4
ENSG00000213967	5,79168396	3,078124724	4,097581562	0,86318243	2,58E-06	zinc finger protein 726	ZNF726
ENSG00000140403	43,70807775	23,29077248	6,540492925	0,863851825	0,000334877	DnaJ heat shock protein fam	DNAJA4
ENSG00000085840	22,05524343	11,53703237	5,534881799	0,86406689	0,000595739	origin recognition complex s	ORC1
ENSG00000029993	31,9770457	17,30224551	5,984991703	0,864345289	0,000155465	high mobility group box 3	HMGB3
ENSG00000155761	4,389313575	2,42566665	3,481013183	0,864529763	0,014051947	sperm associated antigen 17	SPAG17
ENSG00000248890	4,903601522	2,687582555	3,751586032	0,864766293	0,000819897	HHIP antisense RNA 1	HHIP-AS1
ENSG00000229267	1,627315906	0,861832637	2,312014245	0,865688102	0,005048161	small nucleolar RNA host ge	SNHG31
ENSG00000230148	10,02327691	5,272247797	4,788955783	0,866107847	9,45E-05	HOXB cluster antisense RNA	HOXB-AS1
ENSG00000167964	3,981654194	2,207073244	3,433917484	0,8669574	0,004486773	RAB26, member RAS oncoge	RAB26
ENSG00000091622	35,07370847	19,27046487	6,493820464	0,867393014	4,75E-07	PITPNM family member 3	PITPNM3
ENSG00000228716	140,8256623	74,07030458	8,338509369	0,868378422	5,63E-08	dihydrofolate reductase	DHFR
ENSG00000183647	7,145218539	3,783441094	4,240380299	0,869183894	0,000106078	zinc finger protein 530	ZNF530
ENSG00000184497	34,59564462	17,60394147	6,301133218	0,869928437	0,011726729	transmembrane protein 255	TMEM255B
ENSG00000186767	27,52361859	14,87452959	5,824548624	0,870317155	1,33E-05	spindlin family member 4	SPIN4
ENSG00000169129	68,07711925	38,28600132	7,579525296	0,870389738	0,019049717	actin filament associated pro	AFAP1L2
ENSG00000000460	18,76957364	9,93345227	5,481798889	0,871832429	4,90E-06	chromosome 1 open reading	C1orf112
ENSG00000106829	48,23003306	26,15432442	7,027971814	0,871895172	6,01E-05	TLE family member 4, transc	TLE4
ENSG00000258457	3,255532652	1,677359914	3,161627227	0,87202011	0,002526995	AJUBA divergent transcript	AJUBA-DT
ENSG00000061337	53,28794533	29,60205545	6,900205545	0,872577548	0,012481418	leucine zipper tumor suppre	LZTS1
ENSG00000172985	31,59786071	16,71525026	6,197891505	0,87406328	0,017154398	SH3 domain containing ring	SH3RF3
ENSG00000109881	21,61781774	11,42014532	5,741669945	0,874116935	5,07E-05	coiled-coil domain containin	CCDC34
ENSG00000140525	140,1192687	72,48175691	8,374075609	0,874522063	1,12E-05	FA complementation group	FANCI
ENSG00000237476	1,430198755	0,782725089	1,679005376	0,8746984	0,010967056	long intergenic non-protein	LINC01637
ENSG00000174370	3,042779228	1,622025627	3,050482757	0,87487885	1,49E-05	KCNJ5 antisense RNA 1	KCNJ5-AS1
ENSG00000196196	2,554540132	1,389341804	2,646062507	0,875842282	0,00724074	histidine rich carboxyl termi	HRCT1
ENSG00000155850	165,2185389	89,0923278	8,79549669	0,876167982	1,75E-05	solute carrier family 26 mem	SLC26A2
ENSG00000183092	1,776941731	0,928774263	2,541680698	0,87663751	0,027845342	brain enriched guanylate kin	BEGAIN
ENSG00000257702	7,99361549	4,119106642	4,471026766	0,876748302	0,000458664	LBX2 antisense RNA 1	LBX2-AS1
ENSG00000134107	157,2394539	85,32673442	8,832682311	0,877633113	5,00E-07	basic helix-loop-helix family	BHLHE40
ENSG00000122966	104,8340061	52,99567943	7,99885066	0,877776798	2,33E-05	citron rho-interacting serine	CIT
ENSG00000006638	7,041617162	3,836529885	4,560591795	0,878250104	0,000998195	thromboxane A2 receptor	TBXA2R
ENSG00000168676	4,207652485	2,137868624	3,498233148	0,879553219	0,005775547	potassium channel tetramer	KCTD19
ENSG00000204389	95,7143865	50,09638988	7,68448219	0,883697464	1,58E-05	heat shock protein family A	HSPA1A
ENSG00000243414	3,39124794	1,824413421	3,23182866	0,884693239	1,04E-06	toll like receptor adaptor mo	TICAM2
ENSG00000101115	6,212139948	3,333162646	4,069703836	0,885253958	0,001985528	spalt like transcription facto	SALL4
ENSG00000138642	19,30231343	10,20009273	5,492048799	0,885254876	0,000747026	HECT and RLD domain conta	HERC6
ENSG00000117226	90,73250538	49,46355104	7,810460537	0,888632558	2,49E-08	guanylate binding protein 3	GBP3
ENSG00000174871	9,752315502	5,299655547	4,028598444	0,888928229	0,006124288	cornichon family AMPA rece	CNIH2
ENSG00000138376	37,72150097	19,88865797	6,265402145	0,888993089	9,31E-06	BRCA1 associated RING dom	BARD1
ENSG00000158315	5,117531686	2,764985692	3,348397364	0,890182328	0,001150025	rhomboid like 2	RHBDL2
ENSG00000117266	98,44341109	53,46981958	8,523013617	0,89066122	0,002688742	cyclin dependent kinase 18	CDK18
ENSG00000153044	24,1239385	12,59740939	5,912310722	0,891104854	3,54E-05	centromere protein H	CENPH
ENSG00000146918	101,3881376	52,83118319	7,876696428	0,891192735	9,59E-08	non-SMC condensin II comp	NCAPG2
ENSG00000161860	0,598055146	0,30234611	1,199502014	0,892660733	0,026928647	synaptonemal complex cent	SYCE2
ENSG00000077152	34,32753662	17,7709512	6,150130675	0,893024381	0,00011256	ubiquitin conjugating enzym	UBE2T
ENSG00000183722	52,45290934	27,95831535	6,992493427	0,893200923	3,65E-05	LHFPL tetraspan subfamily n	LHFPL6
ENSG00000221829	80,94230565	42,08031572	7,645672208	0,893278338	2,44E-06	FA complementation group	FANCG
ENSG00000100077	2,203539081	1,162895499	3,282262155	0,894791299	0,028578731	G protein-coupled receptor	GRK3
ENSG00000196189	31,5716564	14,74911561	6,500769941	0,895521576	0,026469962	semaphorin 4A	SEMA4A
ENSG00000128284	10,90484978	5,812980468	4,787250013	0,897227631	0,023218135	apolipoprotein L3	APOL3
ENSG00000198513	3,910710193	2,090654265	3,710579121	0,898138955	0,002454459	atlastin GTPase 1	ATL1
ENSG00000267757	3,656745365	1,958303008	2,966068782	0,898297329	0,026192094	EML2 antisense RNA 1	EML2-AS1
ENSG00000233532	1,253854366	0,660145675	1,738551615	0,898368992	0,018840397	long intergenic non-protein	LINC00460
ENSG00000160219	1,943537291	1,040500418	2,409305333	0,900694503	0,003551977	GRB2 associated binding pro	GAB3
ENSG00000169116	0,943762669	0,492164317	1,024267605	0,901033077	0,020562036	prostate androgen-regulated	PARM1
ENSG00000197766	8,318262265	4,000471553	4,011362527	0,901449585	0,035778252	complement factor D	CFD
ENSG00000258017	8,676909399	4,613986038	4,540842199	0,903003485	0,000185702	uncharacterized LOC105369	LOC105369760
ENSG00000146376	100,0108687	51,31632991	7,92014481	0,904281432	0,007701679	Rho GTPase activating prote	ARHGAP18
ENSG00000149380	7,372827239	3,749722819	4,461584008	0,904305499	0,004732847	prolyl 4-hydroxylase subunit	P4HA3
ENSG00000108106	111,8628159	57,44823968	7,824631646	0,904731415	0,000858706	ubiquitin conjugating enzym	UBE2S
ENSG00000205362	7,844850294	3,861989306	3,782635048	0,905063053	0,021815046	metallothionein 1A	MT1A
ENSG00000197275	5,595001583	2,932889214	3,780371273	0,905764091	2,29E-09	RAD54 homolog B	RAD54B
ENSG00000164087	41,87690769	20,97052143	6,910637669	0,906092037	9,05E-05	POC1 centriolar protein A	POC1A
ENSG00000104889	121,5413808	62,23153896	8,183891012	0,906203354	1,50E-05	ribonuclease H2 subunit A	RNASEH2A
ENSG00000115267	37,56134673	19,40443787	6,608285276	0,906632588	0,003617411	interferon induced with heli	IFIH1
ENSG00000050405	151,2106987	78,08610275	8,694250029	0,906854095	0,003801341	LIM domain and actin bindir	LIMA1
ENSG00000181773	3,067837991	1,621289045	2,644122205	0,907049314	0,006422301	G protein-coupled receptor	GPR3
ENSG00000134013	671,9157312	354,9346153	10,79686878	0,907459118	1,30E-10	lysyl oxidase like 2	LOXL2
ENSG00000184988	4,484366922	2,393053624	3,773340393	0,90871395	5,71E-08	transmembrane protein 106	TMEM106A

ENSG00000079459	159,5509035	81,21440343	8,705866664	0,909540032	0,026303842	farnesyl-diphosphate farnes	FDFT1
ENSG00000144485	28,25559104	14,323522	5,501532128	0,914935305	0,001183954	hes family bHLH transcriptio	HES6
ENSG00000101400	38,61645665	19,89783623	6,667845693	0,916195187	0,000212337	syntrophin alpha 1	SNTA1
ENSG00000164877	100,5481967	51,41588962	7,765257205	0,917092087	0,001300111	MICAL like 2	MICALL2
ENSG00000188677	40,27512251	19,5842236	6,553827256	0,919584784	0,035100654	parvin beta	PARVB
ENSG00000188322	14,97892829	7,95879585	5,412404997	0,919926276	0,000132785	SH3 domain binding kinase :	SBK1
ENSG00000180332	1,693233327	0,904935836	1,253990368	0,92041809	0,040703732	potassium channel tetramer	KCTD4
ENSG00000101938	98,74001629	52,27510938	7,852702658	0,921542287	5,24E-06	chordin like 1	CHRDL1
ENSG00000174442	59,74251871	29,80847269	7,270680655	0,922120392	1,22E-06	zwilch kinetochore protein	ZWILCH
ENSG00000162062	26,5232801	13,16536777	6,05186761	0,922227015	0,000148342	tubulin epsilon and delta co	TEDC2
ENSG00000144810	396,7680753	209,9596331	10,31113765	0,922495395	5,24E-06	collagen type VIII alpha 1 ch	COL8A1
ENSG00000136040	10,50619103	5,161056391	4,505242486	0,923359822	0,000540985	plexin C1	PLXNC1
ENSG00000185101	23,31495965	12,389955	5,752884447	0,924796944	0,025923396	anoctamin 9	ANO9
ENSG00000198039	7,716055703	4,017502746	4,517564057	0,92493001	4,08E-12	zinc finger protein 273	ZNF273
ENSG00000259479	3,09398895	1,600135529	2,692145423	0,9249412	9,53E-05	sorbitol dehydrogenase 2, p	SORD2P
ENSG00000154822	29,4513435	15,01217512	6,007847444	0,925692808	9,76E-11	phospholipase C like 2	PLCL2
ENSG00000127586	121,1818896	60,39855024	8,478167386	0,926269679	3,83E-05	chromosome transmission fi	CHTF18
ENSG00000149256	278,3218141	146,397541	9,716936741	0,929546296	5,18E-06	teneurin transmembrane pr	TENM4
ENSG00000166839	4,68499499	2,330246239	3,226493374	0,930107444	0,044045159	ankyrin repeat and death do	ANKDD1A
ENSG00000105472	5,718084408	2,896866613	3,02895342	0,930117885	0,032348721	C-type lectin domain contair	CLEC11A
ENSG00000094804	113,8552576	57,43442018	7,943617575	0,930135185	3,17E-05	cell division cycle 6	CDC6
ENSG00000115129	207,0090426	103,0595408	8,818412773	0,93062615	0,000664577	tumor protein p53 inducible	TP53I3
ENSG00000164104	129,2506452	64,24352551	8,044363744	0,931627639	1,51E-06	high mobility group box 2	HMGB2
ENSG00000175322	10,50952003	5,394096211	4,976498388	0,933611871	3,79E-09	zinc finger protein 519	ZNF519
ENSG00000007968	18,75015208	8,942048621	5,141021289	0,933797173	0,001334456	E2F transcription factor 2	E2F2
ENSG00000189184	2,493183332	1,299517664	3,034325385	0,934271054	0,002305103	protocadherin 18	PCDH18
ENSG00000154553	12,57559527	6,556925792	4,439539184	0,934566974	0,026562729	PDZ and LIM domain 3	PDLIM3
ENSG00000171757	1,27910878	0,648675002	1,50763376	0,934576111	0,027235554	leucine rich repeat containir	LRRC34
ENSG00000158623	24,62679526	12,69156406	6,71487	0,936739585	8,36E-08	COPI coat complex subunit g	COPG2
ENSG00000143369	104,2650744	54,44363483	7,992972519	0,9398756	1,16E-10	extracellular matrix protein	ECM1
ENSG00000168386	48,18401639	21,24034429	6,9637496	0,94054952	0,023237089	filamin A interacting protein	FILIP1L
ENSG00000180535	2,062227207	1,064291179	2,323687471	0,940700924	0,007407596	basic helix-loop-helix family	BHLHA15
ENSG00000138111	10,69235681	5,20555567	4,820430883	0,94102203	6,49E-07	major facilitator superfamily	MFSD13A
ENSG00000266094	7,563211794	3,974126214	3,954865556	0,943307965	0,007670738	Ras association domain fam	RASSF5
ENSG00000198814	7,942060031	3,581230546	3,59411352	0,944108175	0,03054764	glycerol kinase	GK
ENSG00000079931	107,1273588	55,76146796	7,86712686	0,949910379	2,93E-08	monooxygenase DBH like 1	MOXD1
ENSG00000146151	1,102281951	0,570172951	1,597243554	0,949983533	0,008754376	3-hydroxymethyl-3-methylg	HMGCLL1
ENSG00000183032	0,683334491	0,358173814	0,680103898	0,948296293	0,033756303	solute carrier family 25 men	SLC25A21
ENSG00000249115	75,9529135	38,16359809	7,563180293	0,94883367	1,22E-07	HAUS augmin like complex s	HAUS5
ENSG00000077616	4,181949866	2,105784013	3,379478793	0,950035322	0,00039321	N-acetylated alpha-linked ac	NAALAD2
ENSG00000105290	67,35864427	34,94440512	7,359353414	0,950947185	0,00303131	amyloid beta precursor like	APLP1
ENSG00000168811	1,742042891	0,905850416	2,55742869	0,951189147	0,038061862	interleukin 12A	IL12A
ENSG00000127423	7,39945991	3,604873475	4,316476564	0,951728797	1,26E-06	aurora kinase A and ninein ir	AUNIP
ENSG00000132561	65,01675143	32,93833218	7,015534207	0,951948912	0,032537946	matrilin 2	MATN2
ENSG00000176927	1,733197243	0,850753712	2,328134981	0,952435488	0,00516711	EF-hand calcium binding dor	EFCAB5
ENSG00000156463	15,55333027	8,052855931	5,514519341	0,95265368	1,48E-05	SH3 domain containing ring	SH3RF2
ENSG00000167767	56,04085805	28,80887915	7,134134876	0,954230778	4,79E-05	keratin 80	KRT80
ENSG00000111424	33,74822993	17,22454674	6,576134398	0,954495059	1,35E-05	vitamin D receptor	VDR
ENSG00000256546	3,488650611	1,790440818	3,019729654	0,955145259	0,001170333	long intergenic non-protein	LINC02985
ENSG00000144712	35,99372102	17,93670682	6,539981897	0,955695181	3,79E-09	cullin associated and neddyl	CAND2
ENSG00000205336	59,28380442	29,22302075	7,511997937	0,957247939	0,004695041	adhesion G protein-coupled	ADGRG1
ENSG00000164342	28,85696757	14,89640369	6,347700915	0,957288622	4,23E-11	toll like receptor 3	TLR3
ENSG00000178226	0,883657842	0,459236626	1,053789229	0,960249958	0,015728908	serine protease 36	PRSS36
ENSG00000257591	0,629759706	0,324468209	0,5437927	0,960766999	0,024802428	zinc finger protein 625	ZNF625
ENSG00000120217	22,63273397	11,14922435	6,05199844	0,961817524	0,008619514	CD274 molecule	CD274
ENSG00000178947	5,881097706	3,025659681	4,995221112	0,962646489	0,032109428	small integral membrane pr	SMIM10L2A
ENSG00000112029	36,00836725	17,53882596	6,232572713	0,963306618	4,32E-05	F-box protein 5	FBXO5
ENSG00000177943	188,2232144	94,46196053	8,991686482	0,963410108	0,027766555	MAM domain containing 4	MAMDC4
ENSG00000162643	15,87069332	8,094413682	5,153965828	0,964661559	0,002330993	dynein axonemal intermedia	DNAI3
ENSG00000164078	14,27987917	6,6931998	4,637904827	0,965039234	1,15E-05	macrophage stimulating 1 re	MST1R
ENSG00000133119	39,64239921	19,13096556	6,729962899	0,966829417	7,18E-08	replication factor C subunit :	RFC3
ENSG00000165323	10,61539612	5,415949806	4,979731709	0,96703116	0,019054642	FAT atypical cadherin 3	FAT3
ENSG00000247708	1,786481311	0,894943908	2,446160407	0,967489804	0,001833617	STX18 antisense RNA 1 (hea	STX18-AS1
ENSG00000146049	2,353670848	1,184857098	2,368875407	0,967762521	0,007728096	kidney associated DCDC2 an	KAAG1
ENSG00000197355	39,66539198	19,94803091	6,991332497	0,968093983	0,010506744	UDP-N-acetylglucosamine p	UAP1L1
ENSG00000197385	7,924932267	4,018108243	4,381409251	0,968302246	3,02E-08	zinc finger protein 860	ZNF860
ENSG00000130940	18,54088525	8,889210895	4,883653467	0,96928128	0,024810041	castor zinc finger 1	CASZ1
ENSG00000049130	108,2482993	55,80702775	8,105125988	0,972638881	1,51E-06	KIT ligand	KITLG
ENSG00000100629	21,45492935	10,52126266	5,918587652	0,973296746	8,40E-10	centrosomal protein 128	CEP128
ENSG00000080823	17,685751	8,593215106	5,921336803	0,973383883	0,041193853	MOK protein kinase	MOK
ENSG00000159708	1,94114814	0,951122028	2,44218951	0,973438455	0,00224493	leucine rich repeat containir	LRRC36
ENSG00000099974	2,915069652	1,443262511	2,744962666	0,973910918	0,000823495	D-dopachrome tautomerase	DDTL
ENSG00000213654	21,51384833	10,50660175	5,798016669	0,974838791	0,001504048	G protein signaling modulat	GPSM3
ENSG00000085999	48,58419617	23,30389908	6,900004304	0,975152784	6,58E-07	RAD54 like	RAD54L
ENSG00000113810	235,018862	111,6950699	9,135853676	0,975655537	1,36E-05	structural maintenance of cl	SMC4
ENSG00000087245	387,1915983	198,0002418	10,28050085	0,977348786	0,003945547	matrix metallopeptidase 2	MMP2
ENSG00000254670	0,624113024	0,313365451	1,407633947	0,977440638	0,024386684	RASSF10 divergent transcrip	RASSF10-DT
ENSG00000204261	5,723196003	2,787142788	3,863277597	0,978654415	3,62E-05	PSMB8 antisense RNA 1 (hea	PSMB8-AS1
ENSG00000164099	43,0099965	22,03945375	6,713549228	0,979705983	0,019705376	serine protease 12	PRSS12
ENSG00000122952	139,0738473	67,98997319	8,388579593	0,980024158	2,43E-06	ZW10 interacting kinetocho	ZWINT
ENSG00000144152	52,64866653	24,90516701	6,803483434	0,980518766	1,50E-06	fibulin 7	FBLN7
ENSG00000171914	67,96720722	33,03772081	7,624935557	0,980563862	5,88E-07	talin 2	TLN2
ENSG00000142552	54,67020466	27,36184395	7,84793052	0,981358378	0,037177819	reticulocalbin 3	RCN3
ENSG00000118513	3,152554113	1,612806032	2,655476988	0,981434184	0,006346721	MYB proto-oncogene, transc	MYB
ENSG00000171246	1,588516681	0,783075505	1,435110325	0,982122748	0,045398437	neuronal pentraxin 1	NPTX1
ENSG00000106948	109,1815797	53,28140745	8,38144624	0,982674876	2,84E-06	AT-hook transcription factor	AKNA
ENSG00000148219	40,67434969	20,12642866	6,768477712	0,984031979	5,76E-09	astrotactin 2	ASTN2
ENSG00000198826	56,31933652	26,71139862	7,095879225	0,985638269	3,96E-07	Rho GTPase activating prote	ARHGAP11A
ENSG00000260428	13,9275207	6,675085715	4,635746399	0,986289411	0,008883229	scleraxis bHLH transcription	SCX
ENSG00000160111	1,303508231	0,649916165	1,928087245	0,987703903	0,043528118	C3 and PZP like alpha-2-mac	CPAMD8
ENSG00000079156	17,95144658	8,485473102	5,491177609	0,987767854	0,009791593	oxysterol binding protein lik	OSBPL6
ENSG00000240065	36,05378567	17,27520006	6,562908074	0,987852561	6,30E-07	proteasome 20S subunit bet	PSMB9
ENSG00000132932	23,69764775	10,44932547	6,574046146	0,98903143	0,026510085	ATPase phospholipid transp	ATP8A2
ENSG00000067064	69,53372334	33,02146907	7,399596229	0,989426063	0,011901142	isopentenyl-diphosphate de	IDI1
ENSG00000004777	33,48308726	16,74783286	6,297150644	0,991570145	2,06E-05	Rho GTPase activating prote	ARHGAP33
ENSG00000051180	35,04909273	16,74013996	6,449517781	0,991681377	7,56E-07	RAD51 recombinase	RAD51
ENSG00000255624	3,006751867	1,365818042	2,81419562	0,993321847	0,005332561	C10orf88B (pseudogene)	C10orf88B
ENSG00000139173	11,30107544	5,67340006	4,950817777	0,993858574	0,000169678	transmembrane protein 117	TMEM117
ENSG00000117245	5,635447136	2,729164923	3,820520935	0,994456955	0,010038698	kinesin family member 17	KIF17
ENSG00000161800	65,64524149	31,07749958	7,179138782	0,994880816	8,25E-06	Rac GTPase activating prote	RACGAP1
ENSG00000257167	9,860324244	4,886973027	4,074295695	0,995010486	0,007769838	TMPO antisense RNA 1	TMPO-AS1
ENSG00000109743	5,954395181	3,009484268	3,701994975	0,995349022	0,016470966	bone marrow stromal cell ar	BST1
ENSG00000240875	1,674444289	0,816513358	2,794193397	0,996103491	0,010913808	long intergenic non-protein	LINC00886
ENSG00000110031	9,610300214	4,821874768	4,582154487	0,996207678	7,53E-10	leupaxin	LPXN
ENSG00000234883	1,945832859	0,974562372	2,364324527	0,996704192	0,015534069	MIR155 host gene	MIR155HG
ENSG00000126778	69,02444469	33,0433036	6,800058109	0,997386502	0,034335986	SIX homeobox 1	SIX1
ENSG00000123975	49,90967788	23,65515919	6,959880854	0,997836371	1,57E-06	CDC28 protein kinase regula	CKS2
ENSG00000177380	8,349734947	4,139690412	5,03214371	0,998182316	3,29E-07	PTPRF interacting protein al	PPFIA3
ENSG00000136826	7,944124552	3,772480584	4,518720683	0,998445033	0,007801762	Kruppel like factor 4	KLF4
ENSG00000170846	30,32868862	14,5424014	5,986488174	0,999006928	0,002546609	Morf4 family associated pro	LOC93622
ENSG00000162881	1,514346994	0,750421147	1,371534511	1,00124683	0,025427839	oxoeicosanoid receptor 1	OXER1
ENSG00000092853	110,5482439	52,09047133	7,807869706	1,003668697	5,04E-05	claspin	CLSPN
ENSG00000214922	3,258797668	1,612378534	3,305377668	1,003968996	0,003221761	HLA-F antisense RNA 1	HLA-F-AS1
ENSG00000105011	183,1703623	88,15836496	8,656075761	1,005310108	6,07E-05	anti-silencing function 1B hi:	ASF1B
ENSG00000150471	30,99447546	14,34974186	6,651139494	1,00609922	1,33E-06	adhesion G protein-coupled	ADGRL3

ENSG00000245522	3,354071031	1,633989717	3,459913021	1,007286239	0,015543223	long intergenic non-protein	LINC02709
ENSG00000110921	26,18875638	12,12757054	6,031459362	1,007889558	9,06E-05	mevalonate kinase	MVK
ENSG00000231574	7,532548312	3,847204392	4,006096627	1,008138213	0,013944081	long intergenic non-protein	LINC02015
ENSG00000185010	7,314177272	3,510482014	4,047410349	1,008249271	5,07E-06	coagulation factor VIII	F8
ENSG00000100302	13,71493287	6,71085957	6,143251069	1,010509023	0,007109294	RASD family member 2	RASD2
ENSG00000204175	17,11303772	8,661666399	5,569267874	1,010594492	0,000676327	G protein regulated inducer	GPRIN2
ENSG00000162882	8,896535072	4,379798131	3,830744689	1,013104145	0,028508469	3-hydroxyanthranilate 3,4-d	HAAO
ENSG00000113368	176,6183087	87,60028197	8,401905871	1,015239642	3,56E-06	lamin B1	LMNB1
ENSG00000100526	21,28564628	9,707885548	5,302452866	1,0155101	0,00158994	cyclin dependent kinase inhi	CDKN3
ENSG00000139618	52,82300399	24,31663196	7,091291892	1,01640884	0,000180274	BRCA2 DNA repair associate	BRCA2
ENSG00000101335	113,5813847	56,27411639	8,657249973	1,017956282	1,26E-05	myosin light chain 9	MYL9
ENSG00000231784	1,007839853	0,481097309	1,025491542	1,018367907	0,022294647	diazepam binding inhibitor-l	DBIL5P
ENSG00000155893	30,25412533	14,48867622	6,3494088	1,019194617	0,002449869	2-phosphoxylose phosphata	PXYLP1
ENSG00000154096	344,5851911	168,1311852	10,45818739	1,019338526	2,93E-05	Thy-1 cell surface antigen	THY1
ENSG00000168918	152,2359109	75,53914888	8,049922247	1,021396438	0,000115223	inositol polyphosphate-5-ph	INPP5D
ENSG00000136378	146,6225821	72,07836589	8,773247486	1,023451534	1,81E-05	ADAM metallopeptidase wit	ADAMTS7
ENSG00000126215	81,83869659	38,27058175	7,607349439	1,023723109	1,65E-05	X-ray repair cross compleme	XRCC3
ENSG00000167641	11,95946411	5,887829026	4,801077566	1,023770899	0,026075617	protein phosphatase 1 regul	PPP1R14A
ENSG00000173567	0,966474725	0,469929385	1,068111827	1,024806729	0,0046076	adhesion G protein-coupled	ADGRF3
ENSG00000178695	160,5758556	78,17836256	8,116955776	1,02518137	0,005893004	potassium channel tetramer	KCTD12
ENSG00000143476	110,9298537	52,22759547	8,062217765	1,025321999	4,94E-07	denticleless E3 ubiquitin pro	DTL
ENSG00000187595	1,510795991	0,716127544	2,290874292	1,026029041	0,009031165	zinc finger protein 385C	ZNF385C
ENSG00000138646	51,53883992	24,37002096	6,897983619	1,026968903	0,008801429	HECT and RLD domain conta	HERC5
ENSG00000164932	58,59663281	28,90769091	7,181666704	1,027222925	4,13E-09	collagen triple helix repeat c	CTHRC1
ENSG00000158887	5,389437859	2,62915894	3,753239966	1,02746205	0,000458664	myelin protein zero	MPZ
ENSG00000277363	2,620473349	1,152708074	2,970673577	1,027751656	0,019730633	SRC kinase signaling inhibito	SRCIN1
ENSG00000231327	0,93561259	0,425264023	1,495307283	1,029144877	0,003238071	long intergenic non-protein	LINC01816
ENSG00000267530	1,737069569	0,851549878	2,475038871	1,02934765	0,000784744	long intergenic non-protein	LINC01836
ENSG00000145861	1,664536065	0,8058921	1,662524111	1,033204879	0,00092097	C1q and TNF related 2	C1QTNF2
ENSG00000267374	6,996148023	3,225255313	4,044407249	1,033662401	1,37E-06	MIR924 host gene	MIR924HG
ENSG00000104147	9,320476032	4,266980149	4,603631712	1,034123129	0,000666985	Opa interacting protein 5	OIP5
ENSG00000197594	19,91154276	9,077584571	5,426718598	1,034422358	0,001425372	ectonucleotide pyrophospha	ENPP1
ENSG00000158246	14,50290665	6,846429735	5,110151265	1,034805531	1,22E-06	terminal nucleotidyltransfer	TENT5B
ENSG00000130720	11,41531779	5,659760974	5,625838763	1,035315101	0,031471354	fibrinogen C domain contair	FIBCD1
ENSG00000152953	27,524079	13,54828274	6,072329916	1,036152415	0,011241632	serine/threonine kinase 32B	STK32B
ENSG00000171848	91,72521507	41,47168319	7,390598934	1,036595255	0,000591345	ribonucleotide reductase re	RRM2
ENSG00000128408	11,94365528	5,579927978	4,336806288	1,036666589	0,000253179	RIB43A domain with coiled-c	RIBC2
ENSG00000114270	997,2310282	451,614905	11,64388051	1,037495947	0,007083724	collagen type VII alpha 1 cha	COL7A1
ENSG00000167900	191,2089135	86,44614884	8,807385433	1,038528695	3,10E-05	thymidine kinase 1	TK1
ENSG00000265148	0,829015579	0,403225342	1,285889203	1,039918125	0,007180041	TSPOAP1, SUPT4H1 and RNF	TSPOAP1-AS1
ENSG00000226887	4,920959119	2,279040641	3,41540598	1,040124745	0,002165325	endogenous retrovirus grou	ERVMER34-1
ENSG00000235217	12,46506015	5,696288156	5,36721526	1,041991231	2,38E-10	testis specific protein Y-linke	TSPY26P
ENSG00000267201	1,244166264	0,593358214	1,758541587	1,042468924	0,000603209	long intergenic non-protein	LINC01775
ENSG00000250305	8,387857081	3,342910656	4,557535151	1,042623377	3,42E-05	tRNA methyltransferase 9B	TRMT9B
ENSG00000168497	49,6527198	24,76412605	6,341106379	1,043818736	0,017430704	caveolae associated protein	CAVIN2
ENSG00000224957	1,961364797	0,927800099	2,162861208	1,045191856	0,036379049	long intergenic non-protein	LINC01266
ENSG00000238266	3,870440892	1,23141754	2,945874531	1,045530349	0,037177819	long intergenic non-protein	LINC00707
ENSG00000276043	168,9621915	81,79311936	8,325840366	1,04632172	0,000432856	ubiquitin like with PHD and	UHRF1
ENSG00000134470	5,546207621	2,496249099	3,230251616	1,046336114	0,007834421	interleukin 15 receptor subu	IL15RA
ENSG00000079616	81,32181826	36,90196825	7,630007891	1,047823068	1,05E-10	kinesin family member 22	KIF22
ENSG00000102384	30,77330017	13,97662894	6,300374406	1,049230896	4,73E-07	centromere protein I	CENPI
ENSG00000105750	8,940726837	4,198226107	4,586960168	1,049290451	4,19E-08	zinc finger protein 85	ZNF85
ENSG00000146411	7,815576802	3,807749104	4,608856499	1,049948956	1,44E-06	solute carrier family 2 meml	SLC2A12
ENSG00000093009	44,0768388	20,28514121	6,600833142	1,050259011	0,000160739	cell division cycle 45	CDC45
ENSG00000100285	9,029279426	4,356244953	4,043550722	1,050480016	0,000339258	neurofilament heavy chain	NEFH
ENSG00000177694	1,879033842	0,895478658	2,749625648	1,051685346	0,003759728	N-acetylated alpha-linked ac	NAALADL2
ENSG00000116830	56,55703303	25,49628914	7,198570316	1,051705112	1,44E-07	transcription termination fa	TTF2
ENSG00000186472	27,38767994	12,19423583	6,153479902	1,051714763	0,00128293	piccolo presynaptic cytomat	PCLO
ENSG00000040608	24,10859689	11,42683266	5,809645871	1,052908672	0,000269781	reticulon 4 receptor	RTN4R
ENSG00000124116	0,474874618	0,22395131	0,409857852	1,053004732	0,031320534	WAP four-disulfide core don	WFDC3
ENSG00000176208	26,75913401	12,37781874	6,045343321	1,05319305	9,93E-08	ATPase family AAA domain c	ATAD5
ENSG00000181544	12,66835559	5,65184926	5,094819131	1,053579312	3,48E-07	FA complementation group	FANCB
ENSG00000136531	22,97131794	10,73043539	6,185207749	1,053639395	0,000256782	sodium voltage-gated chann	SCN2A
ENSG00000178184	28,18008537	13,46317447	6,163596419	1,055427119	0,000335128	par-6 family cell polarity reg	PARD6G
ENSG00000232810	1,812681172	0,871467814	1,806929496	1,056198491	0,007242775	tumor necrosis factor	TNF
ENSG00000215915	2,31327056	1,096666621	2,46567901	1,056782149	1,34E-05	ATPase family AAA domain c	ATAD3C
ENSG00000241634	0,886907513	0,391606086	1,342682297	1,058633585	0,013724441	ribosomal protein L13 pseuc	RPL13P8
ENSG00000179023	75,55415262	41,77699362	7,586734248	1,0587254	0,028099908	kelch domain containing 7A	KLHDC7A
ENSG00000089820	80,64690777	30,08809804	7,390534183	1,059624495	7,10E-06	Rho GTPase activating prote	ARHGAP4
ENSG00000161692	31,01639301	14,21738882	6,404880349	1,059934518	3,07E-08	DBF4 zinc finger B	DBF4B
ENSG00000067798	4,15962886	1,968151934	3,549192056	1,061316593	1,76E-05	neuron navigator 3	NAV3
ENSG00000255737	8,877096656	4,085811037	4,641353816	1,062879225	0,000563408	AGAP2 antisense RNA 1	AGAP2-AS1
ENSG00000102879	21,33441809	10,13013174	5,054736402	1,062968144	0,000133813	coronin 1A	CORO1A
ENSG00000134955	7,8276776	3,604274313	5,194442755	1,06361132	0,000395011	solute carrier family 37 men	SLC37A2
ENSG00000151164	0,59766429	0,280034095	0,60020394	1,064629981	0,026726947	RAD9 checkpoint clamp com	RAD9B
ENSG00000250802	0,914039052	0,435784365	1,493448714	1,064884419	0,004272698	ZBED3 antisense RNA 1	ZBED3-AS1
ENSG00000164109	42,62605982	19,09734415	6,870026796	1,064958464	1,39E-07	mitotic arrest deficient 2 like	MAD2L1
ENSG00000099365	11,41111331	5,44221477	5,05051332	1,066595997	0,002360173	syntaxin 1B	STX1B
ENSG00000057657	3,522059344	1,622880624	3,57031736	1,068107497	0,000147903	PR/SET domain 1	PRDM1
ENSG00000164591	8,024312039	3,783298092	4,371289653	1,069476396	2,60E-07	myozenin 3	MYOZ3
ENSG00000166165	162,7220651	78,76412653	8,305458379	1,070364325	8,37E-08	creatine kinase B	CKB
ENSG00000138769	10,42456893	4,758763881	4,63799589	1,070447105	9,74E-05	cyclin dependent kinase like	CDKL2
ENSG00000183044	10,42037051	4,594946963	5,258935385	1,070944046	0,00183332	4-aminobutyrate aminotran	ABAT
ENSG00000105974	243,6103351	114,9579508	9,174358021	1,071612041	0,000129906	caveolin 1	CAV1
ENSG00000166825	301,6504931	134,3935659	9,794428206	1,072935255	9,34E-11	alanyl aminopeptidase, men	ANPEP
ENSG00000189431	14,11666131	6,683140447	6,052637537	1,073743116	9,76E-10	Ras association domain fami	RASSF10
ENSG00000185347	38,4619974	16,61995691	6,580162316	1,076777613	4,39E-06	tubulin epsilon and delta co	TEDC1
ENSG00000135362	73,2344082	34,42592635	7,42657835	1,077262879	1,54E-16	proline rich 5 like	PRR5L
ENSG00000180611	8,673503207	4,126675075	4,543528723	1,077388443	7,21E-06	Mab-21 domain containing	MB21D2
ENSG00000164211	50,85602596	23,93738961	6,956067123	1,077562275	0,004662013	StAR related lipid transfer do	STARD4
ENSG00000241288	1,968753492	0,849832797	3,047264912	1,07791062	0,002372672	long intergenic non-protein	LINC02614
ENSG00000166341	29,48922921	13,98286012	6,496969144	1,078731941	0,035534987	dachsous cadherin-related 1	DCHS1
ENSG00000113140	837,8804169	430,665294	10,90978308	1,078972921	0,025125327	secreted protein acidic and c	SPARC
ENSG00000182795	14,15563539	7,08587946	4,176513667	1,080251111	0,049659995	chromosome 1 open reading	C1orf116
ENSG00000136492	41,36484706	18,89274508	6,435076251	1,080484638	7,71E-06	BRCA1 interacting helicase 1	BRIP1
ENSG00000186897	1,814448656	0,851442627	2,216579146	1,082060814	0,014590631	complement C1q like 4	C1QL4
ENSG00000227741	0,435550087	0,201663129	0,272447198	1,08537397	0,047609946	uncharacterized LOC729867	LOC729867
ENSG00000240891	17,53805283	8,112698707	5,341259823	1,086924815	3,97E-09	phosphatidylinositol specific	PLCXD2
ENSG00000186638	25,42020836	10,85178327	6,031388594	1,088106347	1,06E-05	kinesin family member 24	KIF24
ENSG00000159958	1,312818525	0,581703207	2,276928373	1,089185978	0,005094177	TNF receptor superfamily m	TNFRSF13C
ENSG00000148926	73,76496147	34,18573117	8,064084643	1,089798442	0,001173882	adrenomedullin	ADM
ENSG00000203760	17,86170092	8,024134734	5,612184351	1,090644448	1,91E-07	centromere protein W	CENPW
ENSG00000130208	5,100258089	2,194693573	3,684148392	1,090963535	9,10E-05	apolipoprotein C1	APOC1
ENSG00000011332	6,890520582	3,122036005	3,748999916	1,091935123	8,65E-05	double PHD fingers 1	DPF1
ENSG00000123473	49,54623157	21,73096241	6,826297358	1,092500184	2,01E-08	STIL centriolar assembly pro	STIL
ENSG00000196951	0,862578891	0,402275011	1,623340193	1,09308876	0,00365817	SCOC antisense RNA 1	SCOC-AS1
ENSG00000100350	104,3837017	46,86618538	8,076819973	1,093107816	9,14E-10	FAD dependent oxidoreduct	FOXRED2
ENSG00000160207	1,534791096	0,715753294	1,88898058	1,093919881	0,001556554	heat shock transcription fact	HSF2BP
ENSG00000155629	8,899447261	4,182621194	4,997813288	1,094937584	5,24E-05	phosphoinositide-3-kinase a	PIK3AP1
ENSG00000122483	18,94840113	8,135225224	5,530533787	1,096643534	1,03E-06	coiled-coil domain containin	CCDC18
ENSG00000101003	67,56521577	30,23552301	7,172922433	1,097449279	1,65E-10	GINS complex subunit 1	GINS1
ENSG00000180720	1,990683701	0,81724094	1,392080854	1,097705388	0,010123028	cholinergic receptor muscar	CHRM4
ENSG00000147536	76,14888501	33,24492054	7,805136912	1,098653378	4,14E-06	GINS complex subunit 4	GINS4
ENSG00000134824	544,3860767	250,654843	10,33989535	1,099829363	0,001531798	fatty acid desaturase 2	FADS2
ENSG00000136514	5,645252807	2,632325351	3,572870692	1,099957281	1,97E-06	receptor transporter protein	RTP4

ENSG00000065328	56,79800664	24,8179507	6,99601737	1,100699423	2,75E-06	minichromosome maintenai	MCM10
ENSG00000187553	0,851010328	0,391320836	1,185488139	1,101192652	0,008456953	cytochrome P450 family 26	CYP26C1
ENSG00000138658	22,72806975	9,97473973	6,016229677	1,10140993	2,58E-07	zinc finger GRF-type contain	ZGRF1
ENSG00000167779	43,52355078	20,12803293	6,720436361	1,102106363	0,000125457	insulin like growth factor bir	IGFBP6
ENSG00000165891	115,4681451	50,47702069	7,919430624	1,102459801	8,61E-05	E2F transcription factor 7	E2F7
ENSG00000198483	1,751560283	0,794136178	2,543786007	1,104217661	0,002435501	ankyrin repeat domain 35	ANKRD35
ENSG00000204642	39,90173343	16,37414568	6,690099131	1,104497359	0,000704582	major histocompatibility cor	HLA-F
ENSG00000112972	51,51607684	22,99462813	6,998489465	1,104700057	0,008625368	3-hydroxy-3-methylglutaryl-	HMGCS1
ENSG00000171435	1,147881997	0,537269094	2,211623702	1,106279012	0,013080921	kinase suppressor of ras 2	KSR2
ENSG00000139985	0,775678558	0,335439883	1,223576129	1,107053216	0,017402728	ADAM metallopeptidase doı	ADAM21
ENSG00000205089	2,309092597	1,063073849	2,101816018	1,10730888	0,001228083	cyclin I family member 2	CCNI2
ENSG00000204248	5,049069356	2,14571039	4,199224248	1,109482551	0,019158846	collagen type XI alpha 2 chai	COL11A2
ENSG00000012171	59,46142823	26,80052014	7,347633571	1,110737109	0,000581017	semaphorin 3B	SEMA3B
ENSG00000149503	75,71504097	32,61117276	7,375937301	1,112378926	8,65E-07	inner centromere protein	INCENP
ENSG00000173207	36,70505804	15,73901925	6,716430954	1,113190843	4,44E-08	CDC28 protein kinase regula	CKS1B
ENSG00000119917	30,81017581	13,3948081	5,953984965	1,113527289	0,000701765	interferon induced protein v	IFIT3
ENSG00000138182	50,60791243	21,5698215	6,993907912	1,114202847	4,20E-08	kinesin family member 20B	KIF20B
ENSG00000121211	9,635796644	3,995802563	4,529309098	1,115531916	3,59E-05	meiotic nuclear divisions 1	MND1
ENSG00000261359	1,112253537	0,503142325	1,202933985	1,116232588	0,020305569	PYCARD antisense RNA 1	PYCARD-AS1
ENSG00000198342	1,74624786	0,728008216	1,74389901	1,116528378	0,004377378	zinc finger protein 442	ZNF442
ENSG00000224687	1,074010014	0,46948997	1,43735182	1,116693649	0,014599551	RASAL2 antisense RNA 1	RASAL2-AS1
ENSG00000035499	16,85513329	7,378708055	5,07758451	1,116890367	7,66E-07	DEP domain containing 1B	DEPDC1B
ENSG00000115339	40,7494855	18,80586955	5,936017881	1,117306599	0,031519688	polypeptide N-acetylgalacto	GALNT3
ENSG00000168280	6,902636532	2,739786181	3,923113523	1,11766165	0,000446519	kinesin family member 5C	KIF5C
ENSG00000197165	1,742019904	0,772044247	1,962311839	1,121922817	0,002095013	sulfotransferase family 1A m	SULT1A2
ENSG00000066248	6,465280291	2,733139417	3,008033217	1,122247485	0,013763531	neuronal guanine nucleotid	NGEF
ENSG00000103145	111,9075554	50,57290896	8,429122182	1,123014132	1,34E-12	host cell factor C1 regulator	HCFC1R1
ENSG00000149926	0,669071569	0,302595609	0,459692494	1,123384224	0,036704761	TLC domain containing 3B	TLCD3B
ENSG00000197705	13,04809132	5,699596816	5,103541693	1,123639636	4,88E-07	kelch like family member 14	KLHL14
ENSG00000280744	2,674375026	1,229730627	2,617012724	1,124498325	0,010536268	long intergenic non-protein	LINC01173
ENSG00000154920	17,02293365	7,207259797	5,637824853	1,125218621	6,98E-05	essential meiotic structure-s	EME1
ENSG00000139734	85,99354795	36,82159495	7,941585938	1,127504813	2,24E-11	diaphanous related formin 3	DIAPH3
ENSG00000164708	0,673387327	0,302120444	0,136621193	1,127726207	0,014346629	phosphoglycerate mutase 2	PGAM2
ENSG00000104892	1,318475291	0,570357285	1,301304831	1,128430175	0,028279281	kinesin light chain 3	KLC3
ENSG00000137501	65,39991269	29,75026587	7,271341129	1,130791896	7,59E-26	synaptotagmin like 2	SYTL2
ENSG00000145569	22,39788764	9,998718147	5,256930418	1,131607484	4,93E-11	OTU deubiquitinase with lin	OTULINL
ENSG00000138160	82,83561671	34,83581822	7,450348883	1,131680077	1,98E-07	kinesin family member 11	KIF11
ENSG00000166963	27,73714395	13,18526748	6,310304079	1,133298669	0,014918231	microtubule associated prot	MAP1A
ENSG00000166897	127,1001155	58,48969046	8,410836233	1,133893477	2,98E-11	extracellular leucine rich rep	ELFN2
ENSG00000144031	2,592444215	1,129724644	2,855201235	1,134116331	0,001321937	ankyrin repeat domain 53	ANKRD53
ENSG00000225614	8,44384354	3,899129856	4,912701132	1,136726276	0,002537611	zinc finger protein 469	ZNF469
ENSG00000068078	7,896538583	3,1804112	3,243151687	1,1379009	0,014125704	fibroblast growth factor rec	FGFR3
ENSG00000214140	6,998411092	3,085996658	4,374562626	1,139611568	0,000453234	photoreceptor disc compon	PRCD
ENSG00000249693	0,568897855	0,257894497	0,206716152	1,139720007	0,034324429	theg spermatid protein like	THEGL
ENSG00000185338	14,68044683	6,67085611	5,374871821	1,140682	6,75E-12	suppressor of cytokine signa	SOCS1
ENSG00000233654	0,784111395	0,347183889	0,656118124	1,140960218	0,004614715	NEMP2 divergent transcript	NEMP2-DT
ENSG00000154040	21,9438968	9,409876294	5,690161339	1,141632365	1,07E-05	calcium binding tyrosine pho	CABYR
ENSG00000182010	12,88845988	5,631217023	5,235146312	1,141803781	6,24E-07	rhotekin 2	RTKN2
ENSG00000175600	33,6057231	15,34966653	6,723588653	1,142443031	0,00034878	succinyl-CoA:glutarate-CoA	SUGCT
ENSG00000271858	0,47418047	0,21259347	0,738909799	1,148444708	0,022133329	cytochrome b561 family me	CYB561D2
ENSG00000133816	535,8151127	244,9636909	10,28148831	1,149379447	0,001392218	microtubule associated mor	MICAL2
ENSG00000183763	17,07505339	6,938975863	5,660431568	1,149559777	2,85E-06	TRAF interacting protein	TRAIP
ENSG00000119630	71,23540656	30,67476054	6,607800527	1,152749206	0,01677031	placental growth factor	PGF
ENSG00000119922	17,01354377	7,56752977	5,232112788	1,153011677	9,93E-06	interferon induced protein v	IFIT2
ENSG00000151388	69,3845001	31,01453176	7,536725121	1,153429127	8,78E-05	ADAM metallopeptidase wit	ADAMTS12
ENSG00000058085	203,2031319	91,59488961	8,749437492	1,154050915	0,000169904	laminin subunit gamma 2	LAMC2
ENSG00000170629	13,77711047	6,110366756	4,825011992	1,155761619	4,15E-12	DPY19L2 pseudogene 2	DPY19L2P2
ENSG00000144554	61,34096847	25,5582653	7,345516398	1,155973816	6,41E-09	FA complementation group	FANCD2
ENSG00000105409	0,983573753	0,424889774	0,843171027	1,15648166	0,009756391	ATPase Na+/K+ transporting	ATP1A3
ENSG00000165300	17,78493126	7,971994768	5,489038437	1,157149147	5,60E-05	SLIT and NTRK like family me	SLITRK5
ENSG00000172965	26,3892451	11,89254028	5,849304117	1,157190516	7,72E-15	MIR4435-2 host gene	MIR4435-2HG
ENSG00000205744	5,320346464	2,393107626	3,344188906	1,157573541	1,91E-05	DENN domain containing 1C	DENND1C
ENSG00000175352	22,13043491	9,611367006	5,530318853	1,157777893	1,04E-05	nuclear receptor interacting	NRIP3
ENSG00000163507	38,2469503	15,88229527	6,513322629	1,157831998	1,73E-07	cellular inhibitor of PP2A	CIP2A
ENSG00000137727	7,043090937	3,308621852	3,461822909	1,15797891	0,014184892	Rho GTPase activating prote	ARHGAP20
ENSG00000160298	52,72753687	22,5486507	6,810909808	1,160490722	4,93E-08	chromosome 21 open readi	C21orf58
ENSG00000183979	1,951152747	0,806503932	1,923288064	1,160736416	0,000390003	neuropeptide B	NPB
ENSG00000154080	27,88652653	14,19507376	6,149115867	1,161726906	0,022827558	carbohydrate sulfotransfera	CHST9
ENSG00000099960	0,598948223	0,246263325	1,806857944	1,162543357	0,033581189	solute carrier family 7 meml	SLC7A4
ENSG00000143194	0,48447113	0,212444887	0,075548976	1,1638373	0,04109478	maelstrom spermatogenic tı	MAEL
ENSG00000198901	118,8647065	49,5542002	7,999757098	1,164235325	1,63E-07	protein regulator of cytokine	PRC1
ENSG00000100344	8,044278777	3,433340294	4,397235045	1,164295356	0,000952173	patatin like phospholipase d	PNPLA3
ENSG00000266714	22,11775908	9,179483847	6,461963424	1,168914072	0,001623963	myosin XVB	MYO15B
ENSG00000111728	4,607461988	1,955736512	3,768928994	1,168980351	0,000200234	ST8 alpha-N-acetyl-neurami	ST8SIA1
ENSG00000137628	41,99597888	17,66057017	6,308781759	1,16927361	6,05E-05	DExD/H-box helicase 60	DDX60
ENSG00000154134	56,70174628	24,00738196	7,504292319	1,170310101	0,000577484	roundabout guidance recept	ROBO3
ENSG00000235109	11,93991359	5,376809184	4,940361984	1,170752729	0,005384794	zinc finger and SCAN domair	ZSCAN31
ENSG00000099194	500,1048319	215,5738343	10,25266763	1,170866793	0,018593223	stearoyl-CoA desaturase	SCD
ENSG00000100626	5,932425084	2,606110343	2,996611694	1,171408484	0,012941272	polypeptide N-acetylgalacto	GALNT16
ENSG00000183773	3,732777918	1,576071436	3,283472438	1,175193952	0,001331609	apoptosis inducing factor mi	AIFM3
ENSG00000162772	33,5159612	13,96461314	5,863231145	1,1753226	0,003823063	activating transcription facto	ATF3
ENSG00000078114	0,573589242	0,246239491	3,15150649	1,175936423	0,025394888	nebulette	NEBL
ENSG00000107738	26,26953299	11,06710449	6,16292205	1,176153872	0,000149474	V-set immunoregulatory rec	VSIR
ENSG00000196081	5,659649918	2,360886349	3,997637501	1,176446141	6,54E-07	zinc finger protein 724	ZNF724
ENSG00000183831	0,693475844	0,301995694	0,844447745	1,177390503	0,027869524	ankyrin repeat domain 45	ANKRD45
ENSG00000005961	2,201373027	0,928175101	2,397368361	1,179945438	0,005945569	integrin subunit alpha 2b	ITGA2B
ENSG00000197299	36,3204051	14,62712194	6,42229184	1,181701954	4,99E-06	BLM RecQ like helicase	BLM
ENSG00000164742	14,44669694	5,829215495	4,644294244	1,18411352	0,006983448	adenylate cyclase 1	ADCY1
ENSG00000127585	1,927809801	0,806765348	2,237702389	1,185659892	0,005766093	F-box and leucine rich repea	FBXL16
ENSG00000204128	1,527311369	0,683996019	2,309898772	1,18635449	0,005058741	chromosome 2 open reading	C2orf72
ENSG00000123080	43,29244186	18,16944311	6,614812219	1,187191472	8,80E-08	cyclin dependent kinase inhi	CDKN2C
ENSG00000228727	0,541364946	0,234721151	0,781610594	1,187326494	0,016221399	suppressor APC domain con	SAPCD1
ENSG00000267419	1,191442356	0,492229483	1,738022614	1,188137559	0,005903127	zinc finger protein 56, pseuc	ZNF56P
ENSG00000247151	1,561398302	0,649464833	2,11552371	1,192774064	0,001523129	CSTF3 divergent transcript	CSTF3-DT
ENSG00000275793	0,574388329	0,245740492	0,000560547	1,19315734	0,033537391	RIMS binding protein 3	RIMBP3
ENSG00000065989	17,86696703	7,417202601	5,623421736	1,193225302	0,000126774	phosphodiesterase 4A	PDE4A
ENSG00000172244	5,878391143	2,463036912	3,861631245	1,195179169	1,67E-06	chromosome 5 open reading	C5orf34
ENSG00000112149	17,50822439	7,596433041	5,301177053	1,195452916	0,002006486	CD83 molecule	CD83
ENSG00000138587	12,60626881	4,690853673	4,950733805	1,195671652	3,16E-05	meiosis specific nuclear stru	MNS1
ENSG00000234409	7,610475494	3,212181898	4,021122288	1,196041025	0,015311942	coiled-coil domain containin	CCDC188
ENSG00000205277	0,546832029	0,234744985	0,584530628	1,196745252	0,032314022	mucin 12, cell surface associ	MUC12
ENSG00000261008	3,136694812	1,365628127	2,904214011	1,197445018	1,15E-05	long intergenic non-protein	LINC01572

Supplementary file S2

GroupID	Category	Term	Description	LogP	Log(q-valu	Genes	Symbols	InTerm_InList
1_Summa	Reactome Gene	R-HSA-164	Cell Cycle	-70,3658	-66,019	207,580,5	AKT1,BARD1,CCND	222/-
1_Membe	Reactome Gene	R-HSA-164	Cell Cycle	-70,3658	-66,019	207,580,5	AKT1,BARD1,CCND	196/691
1_Membe	Reactome Gene	R-HSA-692	Cell Cycle, Mitotic	-59,2753	-55,406	207,595,9	AKT1,CCND1,CDC6	162/560
1_Membe	Reactome Gene	R-HSA-688	M Phase	-29,1397	-25,907	1060,1460	CENPC,CSNK2B,EM	100/417
1_Membe	Reactome Gene	R-HSA-688	Mitotic Prometaphase	-27,4239	-24,282	1060,1460	CENPC,CSNK2B,CE	66/204
1_Membe	Reactome Gene	R-HSA-255	Mitotic Metaphase and Anaphase	-19,6511	-16,896	1060,2010	CENPC,EMD,CENPI	61/237
1_Membe	Reactome Gene	R-HSA-688	Mitotic Anaphase	-19,0535	-16,341	1060,2010	CENPC,EMD,CENPI	60/236
1_Membe	Reactome Gene	R-HSA-566	RHO GTPases Activate Formins	-17,1469	-14,549	60,752,10	ACTB,FMNL1,CENP	43/140
1_Membe	Reactome Gene	R-HSA-964	EML4 and NUDC in mitotic spindle formatio	-14,4998	-12,062	1060,2491	CENPC,CENPI,INCE	36/117
1_Membe	Reactome Gene	R-HSA-195	RHO GTPase Effectors	-14,2901	-11,883	60,752,80	ACTB,FMNL1,CAL	64/326
1_Membe	Reactome Gene	R-HSA-250	Resolution of Sister Chromatid Cohesion	-13,3895	-11,047	1060,2491	CENPC,CENPI,INCE	36/126
1_Membe	Reactome Gene	R-HSA-246	Separation of Sister Chromatids	-13,1671	-10,858	1060,2491	CENPC,CENPI,INCE	45/191
1_Membe	Reactome Gene	R-HSA-141	Amplification of signal from the kinetochore	-11,5943	-9,394	1060,2491	CENPC,CENPI,INCE	29/96
1_Membe	Reactome Gene	R-HSA-141	Amplification of signal from unattached ki	-11,5943	-9,394	1060,2491	CENPC,CENPI,INCE	29/96
1_Membe	Reactome Gene	R-HSA-696	Mitotic Spindle Checkpoint	-11,1193	-8,971	1060,2491	CENPC,CENPI,INCE	31/113
2_Summa	GO Biological Pr	GO:00062	DNA metabolic process	-62,7227	-58,677	580,641,6	BARD1,BLM,BRCA1	233/-
2_Membe	GO Biological Pr	GO:00062	DNA metabolic process	-62,7227	-58,677	580,641,6	BARD1,BLM,BRCA1	196/761
2_Membe	GO Biological Pr	GO:00069	DNA damage response	-57,6225	-53,878	580,581,5	BARD1,BAX,CCND1	188/755
2_Membe	GO Biological Pr	GO:00062	DNA repair	-51,2266	-47,579	580,641,6	BARD1,BLM,BRCA1	143/503
2_Membe	GO Biological Pr	GO:00062	DNA replication	-48,4618	-44,893	580,641,6	BARD1,BLM,BRCA1	87/198
2_Membe	GO Biological Pr	GO:00062	DNA-templated DNA replication	-38,2775	-34,776	580,641,6	BARD1,BLM,BRCA1	64/135
2_Membe	GO Biological Pr	GO:00063	double-strand break repair	-32,588	-29,283	641,672,6	BLM,BRCA1,BRCA2	73/210
2_Membe	GO Biological Pr	GO:00007	recombinational repair	-27,5781	-24,408	641,672,6	BLM,BRCA1,BRCA2	51/121
2_Membe	GO Biological Pr	GO:00007	double-strand break repair via homologous	-27,3918	-24,276	641,672,6	BLM,BRCA1,BRCA2	50/117
2_Membe	GO Biological Pr	GO:00063	DNA recombination	-26,5064	-23,439	580,641,6	BARD1,BLM,BRCA1	71/242
2_Membe	GO Biological Pr	GO:00450	DNA-templated DNA replication maintenan	-17,0247	-14,434	580,641,6	BARD1,BLM,BRCA1	27/55
2_Membe	GO Biological Pr	GO:00312	replication fork processing	-15,4409	-12,958	580,641,6	BARD1,BLM,BRCA1	24/48
3_Summa	GO Biological Pr	GO:00002	mitotic cell cycle	-36,5959	-33,153	580,595,6	BARD1,CCND1,BL	243/-
3_Membe	GO Biological Pr	GO:00002	mitotic cell cycle	-36,5959	-33,153	580,595,6	BARD1,CCND1,BL	137/609
3_Membe	GO Biological Pr	GO:00512	chromosome organization	-33,3343	-29,988	641,675,1	BLM,BRCA2,CENPC	115/481
3_Membe	GO Biological Pr	GO:19030	mitotic cell cycle process	-28,0297	-24,829	580,595,6	BARD1,CCND1,BL	112/522
3_Membe	GO Biological Pr	GO:00070	chromosome segregation	-21,5939	-18,725	292,672,1	SLC25A5,BRCA1,CE	77/332
3_Membe	GO Biological Pr	GO:00513	cell division	-20,1282	-17,338	595,899,9	CCND1,CCNF,CDC6	97/513
3_Membe	GO Biological Pr	GO:00988	nuclear chromosome segregation	-11,4651	-9,283	1060,2177	CENPC,FANCD2,FL	47/228
3_Membe	GO Biological Pr	GO:00008	sister chromatid segregation	-9,85406	-7,821	1060,2316	CENPC,FLNA,CENPI	33/140
3_Membe	GO Biological Pr	GO:00000	mitotic sister chromatid segregation	-9,55584	-7,556	1060,2316	CENPC,FLNA,CENPI	32/136
3_Membe	GO Biological Pr	GO:01400	mitotic nuclear division	-9,22171	-7,239	1060,2316	CENPC,FLNA,CENPI	35/163
4_Summa	GO Biological Pr	GO:00105	regulation of cell cycle process	-35,3216	-31,929	60,207,27	ACTB,AKT1,BIN1,A	173/-
4_Membe	GO Biological Pr	GO:00105	regulation of cell cycle process	-35,3216	-31,929	60,207,27	ACTB,AKT1,BIN1,A	152/745
4_Membe	GO Biological Pr	GO:19019	regulation of cell cycle phase transition	-22,8501	-19,919	60,207,32	ACTB,AKT1,APBB2,	95/456
4_Membe	GO Biological Pr	GO:00073	regulation of mitotic cell cycle	-21,2961	-18,455	60,207,58	ACTB,AKT1,BARD1,	100/520
4_Membe	GO Biological Pr	GO:00457	negative regulation of cell cycle	-16,2834	-13,715	323,580,5	APBB2,BARD1,CCN	74/379
4_Membe	GO Biological Pr	GO:19019	regulation of mitotic cell cycle phase transit	-16,2261	-13,665	60,207,58	ACTB,AKT1,BARD1,	71/355
4_Membe	GO Biological Pr	GO:00109	negative regulation of cell cycle process	-14,4443	-12,022	323,580,5	APBB2,BARD1,CCN	60/291
4_Membe	GO Biological Pr	GO:00315	DNA integrity checkpoint signaling	-13,7023	-11,334	595,641,6	CCND1,BLM,BRCA1	35/117
4_Membe	GO Biological Pr	GO:00000	cell cycle checkpoint signaling	-12,7771	-10,499	580,595,6	BARD1,CCND1,BL	40/159
4_Membe	GO Biological Pr	GO:19019	negative regulation of cell cycle phase trans	-12,5526	-10,289	323,580,5	APBB2,BARD1,CCN	51/245
4_Membe	GO Biological Pr	GO:00427	signal transduction in response to DNA dam	-12,4797	-10,223	595,637,6	CCND1,BID,BLM,B	37/141
4_Membe	GO Biological Pr	GO:00000	DNA damage checkpoint signaling	-10,934	-8,802	595,641,6	CCND1,BLM,BRCA1	30/108
4_Membe	GO Biological Pr	GO:00459	negative regulation of mitotic cell cycle	-10,0762	-8,020	580,595,6	BARD1,CCND1,BL	45/232
4_Membe	GO Biological Pr	GO:19027	regulation of cell cycle G2/M phase transitic	-9,39073	-7,400	580,595,6	BARD1,CCND1,BL	29/116
4_Membe	GO Biological Pr	GO:00447	mitotic DNA integrity checkpoint signaling	-9,0458	-7,085	595,641,6	CCND1,BLM,BRCA1	24/85
4_Membe	GO Biological Pr	GO:00070	mitotic cell cycle checkpoint signaling	-8,57475	-6,643	580,595,6	BARD1,CCND1,BL	29/125
4_Membe	GO Biological Pr	GO:00103	regulation of G2/M transition of mitotic cell	-8,49167	-6,571	580,595,6	BARD1,CCND1,BL	26/104
4_Membe	GO Biological Pr	GO:00447	mitotic DNA damage checkpoint signaling	-8,00458	-6,113	595,641,6	CCND1,BLM,BRCA1	22/81
4_Membe	GO Biological Pr	GO:20000	regulation of G1/S transition of mitotic cell	-7,83205	-5,946	60,207,59	ACTB,AKT1,CCND1,	35/183
4_Membe	GO Biological Pr	GO:19028	regulation of cell cycle G1/S phase transitio	-7,31364	-5,467	60,207,59	ACTB,AKT1,CCND1,	37/209
4_Membe	GO Biological Pr	GO:19019	negative regulation of mitotic cell cycle pha	-6,35041	-4,587	580,595,6	BARD1,CCND1,BL	32/182
4_Membe	GO Biological Pr	GO:00448	mitotic G2/M transition checkpoint	-3,8098	-2,368	580,641,6	BARD1,BLM,BRCA1	12/53
4_Membe	GO Biological Pr	GO:00070	mitotic G2 DNA damage checkpoint signalin	-3,23517	-1,898	641,672,1	BLM,BRCA1,CHEK1	9/37
4_Membe	GO Biological Pr	GO:19027	negative regulation of cell cycle G2/M phase	-3,17875	-1,855	580,641,6	BARD1,BLM,BRCA1	13/70
4_Membe	GO Biological Pr	GO:00109	negative regulation of G2/M transition of m	-2,83415	-1,585	580,641,6	BARD1,BLM,BRCA1	12/67
5_Summa	WikiPathways	WP2446	Retinoblastoma gene in cancer	-29,5383	-26,271	580,595,9	BARD1,CCND1,CDC	139/-
5_Membe	WikiPathways	WP2446	Retinoblastoma gene in cancer	-29,5383	-26,271	580,595,9	BARD1,CCND1,CDC	46/90
5_Membe	Reactome Gene	R-HSA-696	Cell Cycle Checkpoints	-26,7124	-23,621	580,641,6	BARD1,BLM,BRCA1	79/293
5_Membe	Reactome Gene	R-HSA-692	S Phase	-23,9755	-20,991	207,595,9	AKT1,CCND1,CDC6	55/163

5_Membe	Reactome Gene	R-HSA-453	Mitotic G1 phase and G1/S transition	-23,4869	-20,521	207,595,9	AKT1,CCND1,CDC6	52/149
5_Membe	Reactome Gene	R-HSA-692	G1/S Transition	-22,7963	-19,881	207,595,9	AKT1,CCND1,CDC6	48/131
5_Membe	WikiPathways	WP466	DNA replication	-21,2428	-18,415	990,1017,	CDC6,CDK2,MCM3	27/42
5_Membe	Reactome Gene	R-HSA-692	Synthesis of DNA	-19,8938	-17,116	990,1017,	CDC6,CDK2,DNA2,	43/121
5_Membe	Reactome Gene	R-HSA-176	Activation of ATR in response to replication	-17,699	-15,060	990,993,1	CDC6,CDC25A,CDK	23/37
5_Membe	Reactome Gene	R-HSA-689	Activation of the pre-replicative complex	-16,5173	-13,942	990,1017,	CDC6,CDK2,MCM3	21/33
5_Membe	WikiPathways	WP45	G1 to S cell cycle control	-15,9682	-13,428	595,993,1	CCND1,CDC25A,CD	28/64
5_Membe	Reactome Gene	R-HSA-693	DNA Replication	-15,5615	-13,060	990,1017,	CDC6,CDK2,DNA2,	48/187
5_Membe	Reactome Gene	R-HSA-694	G2/M Checkpoints	-15,4933	-13,004	580,641,6	BARD1,BLM,BRCA1	45/167
5_Membe	KEGG Pathway	hsa04110	Cell cycle	-12,2865	-10,047	595,990,9	CCND1,CDC6,CDC2	39/157
5_Membe	GO Biological Pr	GO:00062	DNA replication initiation	-10,442	-8,358	990,4172,	CDC6,MCM3,MCM	15/28
5_Membe	WikiPathways	WP179	Cell cycle	-10,3825	-8,306	595,990,9	CCND1,CDC6,CDC2	31/120
5_Membe	Reactome Gene	R-HSA-690	DNA Replication Pre-Initiation	-7,21609	-5,379	990,1017,	CDC6,CDK2,H2AX,	31/159
6_Summa	Reactome Gene	R-HSA-722	Processing of Capped Intron-Containing Pre	-25,3859	-22,340	988,1478,	CDC5L,CSTF2,CSTF	155/-
6_Membe	Reactome Gene	R-HSA-722	Processing of Capped Intron-Containing Pre	-25,3859	-22,340	988,1478,	CDC5L,CSTF2,CSTF	76/285
6_Membe	Reactome Gene	R-HSA-895	Metabolism of RNA	-19,272	-16,538	207,988,1	AKT1,CDC5L,CSTF2	118/716
6_Membe	GO Biological Pr	GO:00160	mRNA metabolic process	-17,2175	-14,604	333,988,1	APLP1,CDC5L,CSTF	106/646
6_Membe	WikiPathways	WP411	mRNA processing	-15,7463	-13,219	1478,1479	CSTF2,CSTF3,HNRN	39/126
6_Membe	Reactome Gene	R-HSA-721	mRNA Splicing - Major Pathway	-15,6854	-13,165	988,3178,	CDC5L,HNRNPA1,H	51/207
6_Membe	Reactome Gene	R-HSA-721	mRNA Splicing	-15,6144	-13,107	988,3178,	CDC5L,HNRNPA1,H	52/215
6_Membe	GO Biological Pr	GO:00063	mRNA processing	-14,4543	-12,027	333,988,1	APLP1,CDC5L,CSTF	83/489
6_Membe	GO Biological Pr	GO:00003	RNA splicing, via transesterification reaction	-13,5763	-11,212	988,3178,	CDC5L,HNRNPA1,H	57/279
6_Membe	GO Biological Pr	GO:00003	RNA splicing, via transesterification reaction	-13,2877	-10,962	988,3178,	CDC5L,HNRNPA1,H	56/275
6_Membe	GO Biological Pr	GO:00003	mRNA splicing, via spliceosome	-13,2877	-10,962	988,3178,	CDC5L,HNRNPA1,H	56/275
6_Membe	CORUM	CORUM:3	Spliceosome	-12,4797	-10,223	988,6426,	CDC5L,SRSF1,SRSF	37/141
6_Membe	GO Biological Pr	GO:00083	RNA splicing	-11,5325	-9,338	988,3178,	CDC5L,HNRNPA1,H	68/409
6_Membe	KEGG Pathway	hsa03040	Spliceosome	-10,0367	-7,985	988,3178,	CDC5L,HNRNPA1,H	43/216
6_Membe	CORUM	CORUM:1	C complex spliceosome	-9,77249	-7,748	988,3178,	CDC5L,HNRNPA1,H	24/79
7_Summa	Reactome Gene	R-HSA-738	DNA Repair	-25,2943	-22,270	60,580,64	ACTB,BARD1,BLM,	118/-
7_Membe	Reactome Gene	R-HSA-738	DNA Repair	-25,2943	-22,270	60,580,64	ACTB,BARD1,BLM,	83/335
7_Membe	Reactome Gene	R-HSA-568	HDR through Homologous Recombination (	-25,1759	-22,172	580,641,6	BARD1,BLM,BRCA1	37/68
7_Membe	Reactome Gene	R-HSA-569	DNA Double-Strand Break Repair	-21,5751	-18,720	580,641,6	BARD1,BLM,BRCA1	53/168
7_Membe	Reactome Gene	R-HSA-569	Homology Directed Repair	-20,7824	-17,980	580,641,6	BARD1,BLM,BRCA1	47/138
7_Membe	Reactome Gene	R-HSA-569	Homologous DNA Pairing and Strand Exchar	-19,4883	-16,744	580,641,6	BARD1,BLM,BRCA1	26/43
7_Membe	Reactome Gene	R-HSA-569	HDR through Homologous Recombination (	-19,0826	-16,359	580,641,6	BARD1,BLM,BRCA1	44/132
7_Membe	KEGG Pathway	hsa03460	Fanconi anemia pathway	-18,4584	-15,765	641,672,6	BLM,BRCA1,BRCA2	28/54
7_Membe	WikiPathways	WP4946	DNA repair pathways, full network	-18,2777	-15,594	672,675,1	BRCA1,BRCA2,CHE	41/120
7_Membe	Canonical Pathw	M1	PID FANCONI PATHWAY	-18,1279	-15,453	641,672,6	BLM,BRCA1,BRCA2	26/47
7_Membe	Reactome Gene	R-HSA-569	Presynaptic phase of homologous DNA pairi	-17,924	-15,276	580,641,6	BARD1,BLM,BRCA1	24/40
7_Membe	Reactome Gene	R-HSA-967	Diseases of DNA Double-Strand Break Repai	-17,57	-14,948	580,641,6	BARD1,BLM,BRCA1	24/41
7_Membe	Reactome Gene	R-HSA-970	Defective homologous recombination repai	-17,57	-14,948	580,641,6	BARD1,BLM,BRCA1	24/41
7_Membe	Reactome Gene	R-HSA-967	Diseases of DNA repair	-15,7931	-13,260	580,641,6	BARD1,BLM,BRCA1	25/51
7_Membe	KEGG Pathway	hsa03440	Homologous recombination	-15,0572	-12,603	580,641,6	BARD1,BLM,BRCA1	22/41
7_Membe	WikiPathways	WP4016	DNA IR-damage and cellular response via A	-13,745	-11,372	580,672,6	BARD1,BRCA1,BRC	29/81
7_Membe	Reactome Gene	R-HSA-569	Resolution of D-loop Structures through Ho	-13,5229	-11,168	580,641,6	BARD1,BLM,BRCA1	19/34
7_Membe	Reactome Gene	R-HSA-569	Resolution of D-Loop Structures	-13,2111	-10,898	580,641,6	BARD1,BLM,BRCA1	19/35
7_Membe	Reactome Gene	R-HSA-970	Impaired BRCA2 binding to RAD51	-13,2111	-10,898	580,641,6	BARD1,BLM,BRCA1	19/35
7_Membe	Reactome Gene	R-HSA-569	Resolution of D-loop Structures through Syn	-12,0243	-9,795	580,641,6	BARD1,BLM,BRCA1	16/27
7_Membe	Reactome Gene	R-HSA-970	Impaired BRCA2 binding to PALB2	-11,7878	-9,568	580,641,6	BARD1,BLM,BRCA1	15/24
7_Membe	Reactome Gene	R-HSA-568	HDR through Single Strand Annealing (SSA)	-11,4762	-9,291	580,641,6	BARD1,BLM,BRCA1	18/37
7_Membe	Reactome Gene	R-HSA-970	Defective homologous recombination repai	-11,4176	-9,250	580,641,6	BARD1,BLM,BRCA1	15/25
7_Membe	Reactome Gene	R-HSA-970	Defective homologous recombination repai	-11,4176	-9,250	580,641,6	BARD1,BLM,BRCA1	15/25
7_Membe	Reactome Gene	R-HSA-970	Defective HDR through Homologous Recom	-11,4176	-9,250	580,641,6	BARD1,BLM,BRCA1	15/25
7_Membe	Reactome Gene	R-HSA-970	Defective HDR through Homologous Recom	-11,4176	-9,250	580,641,6	BARD1,BLM,BRCA1	15/25
7_Membe	Reactome Gene	R-HSA-680	Regulation of TP53 Activity through Phosph	-11,3067	-9,151	580,641,6	BARD1,BLM,BRCA1	28/92
7_Membe	Reactome Gene	R-HSA-563	Regulation of TP53 Activity	-10,0686	-8,014	207,580,6	AKT1,BARD1,PRDN	36/160
7_Membe	Reactome Gene	R-HSA-569	Processing of DNA double-strand break end	-9,19871	-7,220	580,641,6	BARD1,BLM,BRCA1	26/97
7_Membe	Reactome Gene	R-HSA-694	G2/M DNA damage checkpoint	-8,08638	-6,190	580,641,6	BARD1,BLM,BRCA1	24/94
7_Membe	Reactome Gene	R-HSA-150	Meiosis	-4,53315	-2,996	641,672,6	BLM,BRCA1,BRCA2	21/117
7_Membe	Reactome Gene	R-HSA-147	Reproduction	-4,14332	-2,652	639,641,6	PRDM1,BLM,BMP4	24/152
7_Membe	Reactome Gene	R-HSA-912	Meiotic recombination	-3,84973	-2,401	641,672,6	BLM,BRCA1,BRCA2	16/85
8_Summa	Reactome Gene	R-HSA-691	DNA strand elongation	-23,1201	-20,172	1763,2237	DNA2,FEN1,LIG1,M	96/-
8_Membe	Reactome Gene	R-HSA-691	DNA strand elongation	-23,1201	-20,172	1763,2237	DNA2,FEN1,LIG1,M	25/32
8_Membe	KEGG Pathway	hsa03030	DNA replication	-18,0927	-15,427	1763,2237	DNA2,FEN1,LIG1,M	23/36
8_Membe	Reactome Gene	R-HSA-691	Lagging Strand Synthesis	-15,2587	-12,781	1763,2237	DNA2,FEN1,LIG1,P	16/20
8_Membe	Reactome Gene	R-HSA-174	Telomere C-strand (Lagging Strand) Synthes	-14,7999	-12,351	641,1763,	BLM,DNA2,FEN1,LI	20/34
8_Membe	Reactome Gene	R-HSA-738	Chromosome Maintenance	-14,0437	-11,652	641,1017,	BLM,CDK2,CENPC,	39/140
8_Membe	Reactome Gene	R-HSA-110	Resolution of AP sites via the multiple-nucle	-12,3863	-10,133	670,2237,	BPHL,FEN1,LIG1,P	16/26

8_Membe	Reactome Gene	R-HSA-565	PCNA-Dependent Long Patch Base Excision I	-11,6039	-9,397	2237,3978	FEN1,LIG1,PCNA,P(	14/21
8_Membe	Reactome Gene	R-HSA-691	Processive synthesis on the lagging strand	-11,5436	-9,346	1763,2237	DNA2,FEN1,LIG1,P(	12/15
8_Membe	Reactome Gene	R-HSA-180	Extension of Telomeres	-11,517	-9,329	641,1017,:	BLM,CDK2,DNA2,FI	21/51
8_Membe	Reactome Gene	R-HSA-569	Gap-filling DNA repair synthesis and ligation	-11,4176	-9,250	3978,5111	LIG1,PCNA,POLD1,I	15/25
8_Membe	Reactome Gene	R-HSA-738	DNA Damage Bypass	-11,1144	-8,969	5111,5424	PCNA,POLD1,POLD	20/48
8_Membe	Reactome Gene	R-HSA-110	Recognition of DNA damage by PCNA-conta	-11,0605	-8,918	5111,5424	PCNA,POLD1,POLD	16/30
8_Membe	WikiPathways	WP531	DNA mismatch repair	-10,8125	-8,691	3978,4292	LIG1,MLH1,PCNA,P	14/23
8_Membe	Reactome Gene	R-HSA-565	Termination of translesion DNA synthesis	-10,5	-8,399	5111,5424	PCNA,POLD1,POLD	16/32
8_Membe	Reactome Gene	R-HSA-690	Polymerase switching	-10,4623	-8,371	5111,5422	PCNA,POLA1,POLD	11/14
8_Membe	Reactome Gene	R-HSA-691	Leading Strand Synthesis	-10,4623	-8,371	5111,5422	PCNA,POLA1,POLD	11/14
8_Membe	Reactome Gene	R-HSA-691	Removal of the Flap Intermediate	-10,4623	-8,371	1763,2237	DNA2,FEN1,PCNA,I	11/14
8_Membe	Reactome Gene	R-HSA-110	Translesion synthesis by Y family DNA polym	-9,91909	-7,880	5111,5424	PCNA,POLD1,POLD	17/39
8_Membe	Reactome Gene	R-HSA-739	Resolution of Abasic Sites (AP sites)	-9,91909	-7,880	670,2237,:	BPHL,FEN1,LIG1,PC	17/39
8_Membe	Reactome Gene	R-HSA-174	Polymerase switching on the C-strand of the	-9,82298	-7,792	5111,5422	PCNA,POLA1,POLD	14/26
8_Membe	KEGG Pathway	hsa03410	Base excision repair	-8,92989	-6,978	2237,3146	FEN1,HMGB1,LIG1,	17/44
8_Membe	Reactome Gene	R-HSA-569	Dual Incision in GG-NER	-8,51634	-6,590	1643,5111	DDB2,PCNA,POLD1	16/41
8_Membe	CORUM	CORUM:1:	DNA synthesome complex (15 subunits)	-8,42168	-6,505	3978,5111	LIG1,PCNA,POLA1,I	10/15
8_Membe	KEGG Pathway	hsa03430	Mismatch repair	-8,28983	-6,383	3978,4292	LIG1,MLH1,PCNA,P	12/23
8_Membe	Reactome Gene	R-HSA-174	Processive synthesis on the C-strand of the t	-8,28107	-6,379	641,1763,:	BLM,DNA2,FEN1,LI	11/19
8_Membe	WikiPathways	WP5114	Nucleotide excision repair in xeroderma pig	-7,92841	-6,041	672,1643,:	BRCA1,DDB2,HMGI	21/75
8_Membe	Reactome Gene	R-HSA-569	Global Genome Nucleotide Excision Repair (	-7,68772	-5,817	60,1643,3!	ACTB,DDB2,LIG1,P(	22/84
8_Membe	CORUM	CORUM:1(	DNA synthesome complex (17 subunits)	-7,66432	-5,799	5111,5422	PCNA,POLA1,POLD	10/17
8_Membe	CORUM	CORUM:1:	DNA synthesome complex (17 subunits)	-7,66432	-5,799	5111,5422	PCNA,POLA1,POLD	10/17
8_Membe	GO Biological Pr	GO:00718!	DNA biosynthetic process	-7,49579	-5,639	1032,3320	CDKN2D,HSP90AA:	23/93
8_Membe	Reactome Gene	R-HSA-569	Nucleotide Excision Repair	-7,21589	-5,379	60,1643,3:	ACTB,DDB2,HMGN	25/111
8_Membe	GO Biological Pr	GO:00063(	postreplication repair	-7,08958	-5,260	672,5111,!	BRCA1,PCNA,POLD	13/33
8_Membe	GO Biological Pr	GO:00007:	DNA synthesis involved in DNA repair	-6,9882	-5,168	1032,5111	CDKN2D,PCNA,POL	14/39
8_Membe	Reactome Gene	R-HSA-678	Gap-filling DNA repair synthesis and ligation	-6,80914	-5,004	3150,3978	HMGN1,LIG1,PCNA	18/65
8_Membe	WikiPathways	WP4752	Base excision repair	-6,4897	-4,715	2237,3146	FEN1,HMGB1,LIG1,	12/31
8_Membe	CORUM	CORUM:1(	DNA synthesome complex (13 subunits)	-6,43219	-4,662	5111,5422	PCNA,POLA1,POLD	8/13
8_Membe	Reactome Gene	R-HSA-174	Removal of the Flap Intermediate from the (	-6,41485	-4,648	1763,2237	DNA2,FEN1,PCNA,I	9/17
8_Membe	WikiPathways	WP4753	Nucleotide excision repair	-6,24382	-4,491	1643,3978	DDB2,LIG1,PCNA,P	14/44
8_Membe	CORUM	CORUM:1:	DNA synthesome core complex	-6,23164	-4,486	5111,5422	PCNA,POLA1,POLD	7/10
8_Membe	Reactome Gene	R-HSA-678	Transcription-Coupled Nucleotide Excision F	-6,10622	-4,377	3150,3978	HMGN1,LIG1,PCNA	19/79
8_Membe	Reactome Gene	R-HSA-535	Mismatch repair (MMR) directed by MSH2:I	-6,09058	-4,366	3978,4292	LIG1,MLH1,PCNA,P	8/14
8_Membe	Reactome Gene	R-HSA-535	Mismatch repair (MMR) directed by MSH2:I	-6,09058	-4,366	3978,4292	LIG1,MLH1,PCNA,P	8/14
8_Membe	Reactome Gene	R-HSA-157	Telomere Maintenance	-5,93738	-4,229	641,1017,:	BLM,CDK2,DNA2,FI	23/112
8_Membe	GO Biological Pr	GO:00199!	translesion synthesis	-5,84905	-4,157	5111,5424	PCNA,POLD1,POLD	10/24
8_Membe	Reactome Gene	R-HSA-535	Mismatch Repair	-5,78592	-4,099	3978,4292	LIG1,MLH1,PCNA,P	8/15
8_Membe	Reactome Gene	R-HSA-738	Base Excision Repair	-5,72881	-4,044	670,2237,:	BPHL,FEN1,H2AX,L	20/91
8_Membe	Reactome Gene	R-HSA-678	Dual incision in TC-NER	-5,29028	-3,653	3150,5111	HMGN1,PCNA,POL	16/66
8_Membe	CORUM	CORUM:1(	RC complex during S-phase of cell cycle	-5,15415	-3,537	1017,3978	CDK2,LIG1,POLA1,I	7/13
8_Membe	WikiPathways	WP186	Homologous recombination	-5,15415	-3,537	675,5424,!	BRCA2,POLD1,POLI	7/13
8_Membe	GO Biological Pr	GO:00422'	error-prone translesion synthesis	-4,83568	-3,263	5425,5427	POLD2,POLE2,POLI	6/10
8_Membe	KEGG Pathway	hsa03420	Nucleotide excision repair	-4,25232	-2,748	1643,3978	DDB2,LIG1,PCNA,P	14/63
8_Membe	CORUM	CORUM:2!	PCNA-DNA polymerase delta complex	-4,02804	-2,560	5111,5424	PCNA,POLD1,POLD	4/5
8_Membe	GO Biological Pr	GO:00062	base-excision repair	-3,45455	-2,074	1763,2237	DNA2,FEN1,LIG1,P(	10/42
8_Membe	GO Biological Pr	GO:00062	nucleotide-excision repair	-3,42037	-2,046	675,1643,:	BRCA2,DDB2,FANC	12/58
8_Membe	CORUM	CORUM:1(	RC complex during G2/M-phase of cell cycle	-2,96713	-1,687	5422,5424	POLA1,POLD1,RFC2	5/13
8_Membe	GO Biological Pr	GO:00709	error-free translesion synthesis	-2,57159	-1,382	5424,5429	POLD1,POLH,REV1	3/5
9_Summai	Reactome Gene	R-HSA-194	Signaling by Rho GTPases	-22,6683	-19,769	60,87,393,	ACTB,ACTN1,ARHG	125/-
9_Membe	Reactome Gene	R-HSA-194	Signaling by Rho GTPases	-22,6683	-19,769	60,87,393,	ACTB,ACTN1,ARHG	124/706
9_Membe	Reactome Gene	R-HSA-971	Signaling by Rho GTPases, Miro GTPases anc	-22,2849	-19,401	60,87,393,	ACTB,ACTN1,ARHG	125/722
9_Membe	Reactome Gene	R-HSA-901	RHO GTPase cycle	-10,9572	-8,823	60,87,393,	ACTB,ACTN1,ARHG	71/449
10_Summ	GO Biological Pr	GO:00510!	regulation of DNA metabolic process	-20,8523	-18,037	60,207,30(	ACTB,AKT1,ANXA3,	144/-
10_Memb	GO Biological Pr	GO:00510!	regulation of DNA metabolic process	-20,8523	-18,037	60,207,30(	ACTB,AKT1,ANXA3,	100/527
10_Memb	GO Biological Pr	GO:00510!	positive regulation of DNA metabolic proces	-15,0968	-12,637	60,207,30(	ACTB,AKT1,ANXA3,	62/298
10_Memb	GO Biological Pr	GO:00801	regulation of cellular response to stress	-9,52647	-7,528	60,355,58(	ACTB,FAS,BARD1,B	88/655
10_Memb	GO Biological Pr	GO:00062	regulation of DNA repair	-7,97279	-6,084	60,580,60!	ACTB,BARD1,BCL7/	39/215
10_Memb	GO Biological Pr	GO:20007	regulation of double-strand break repair	-5,64774	-3,976	60,605,11:	ACTB,BCL7A,CHEK1	25/133
10_Memb	GO Biological Pr	GO:00457	positive regulation of DNA repair	-4,79795	-3,230	60,605,67:	ACTB,BCL7A,BRCA1	23/130
10_Memb	GO Biological Pr	GO:20007!	positive regulation of double-strand break r	-3,2742	-1,930	60,605,16(	ACTB,BCL7A,DDX1:	15/86
10_Memb	GO Biological Pr	GO:00000	regulation of DNA recombination	-2,75181	-1,521	60,641,11:	ACTB,BLM,CHEK1,F	19/136
11_Summ	GO Biological Pr	GO:00063:	chromatin organization	-19,7809	-17,014	60,605,67:	ACTB,BCL7A,BRCA1	125/-
11_Memb	GO Biological Pr	GO:00063:	chromatin organization	-19,7809	-17,014	60,605,67:	ACTB,BCL7A,BRCA1	125/771
11_Memb	GO Biological Pr	GO:00063:	chromatin remodeling	-14,305	-11,893	60,605,67:	ACTB,BCL7A,BRCA1	97/623
12_Summ	GO Biological Pr	GO:00002	nuclear division	-18,5939	-15,891	675,1060,:	BRCA2,CENPC,CKS2	86/-
12_Memb	GO Biological Pr	GO:00002	nuclear division	-18,5939	-15,891	675,1060,:	BRCA2,CENPC,CKS2	70/315

12_Memb	GO Biological Pr	GO:00482	organelle fission	-17,2098	-14,604	675,1060,	BRCA2,CENPC,CKS2	71/341
12_Memb	GO Biological Pr	GO:00513	meiotic cell cycle	-14,4778	-12,045	675,1017,	BRCA2,CDK2,CENP	55/251
12_Memb	GO Biological Pr	GO:19030	meiotic cell cycle process	-13,4332	-11,087	675,1060,	BRCA2,CENPC,CKS2	45/188
12_Memb	GO Biological Pr	GO:01400	meiotic nuclear division	-11,9416	-9,719	675,1060,	BRCA2,CENPC,CKS2	40/168
12_Memb	GO Biological Pr	GO:00358	homologous recombination	-11,6643	-9,455	580,672,2	BARD1,BRCA1,FAN	24/66
12_Memb	GO Biological Pr	GO:00071	meiosis I	-10,086	-8,027	675,1060,	BRCA2,CENPC,CKS2	31/123
12_Memb	GO Biological Pr	GO:00619	meiosis I cell cycle process	-9,89417	-7,857	675,1060,	BRCA2,CENPC,CKS2	31/125
12_Memb	GO Biological Pr	GO:00071	reciprocal meiotic recombination	-8,4889	-6,571	2177,4292	FANCD2,MLH1,RAD	19/58
12_Memb	GO Biological Pr	GO:01405	reciprocal homologous recombination	-8,4889	-6,571	2177,4292	FANCD2,MLH1,RAD	19/58
12_Memb	GO Biological Pr	GO:00701	chromosome organization involved in meiot	-6,07964	-4,358	2177,4292	FANCD2,MLH1,RAD	17/65
12_Memb	GO Biological Pr	GO:00451	meiotic chromosome segregation	-4,26558	-2,758	1060,2177	CENPC,FANCD2,ML	17/87
12_Memb	GO Biological Pr	GO:00451	homologous chromosome segregation	-3,57447	-2,168	1060,2177	CENPC,FANCD2,ML	13/64
12_Memb	GO Biological Pr	GO:00071	homologous chromosome pairing at meiosis	-3,19363	-1,868	2177,4292	FANCD2,MLH1,CCN	11/53
13_Summ	GO Biological Pr	GO:00900	positive regulation of cell cycle process	-17,9758	-15,320	207,595,9	AKT1,CCND1,CDC6,	68/-
13_Memb	GO Biological Pr	GO:00900	positive regulation of cell cycle process	-17,9758	-15,320	207,595,9	AKT1,CCND1,CDC6,	62/262
13_Memb	GO Biological Pr	GO:00457	positive regulation of cell cycle	-15,9825	-13,435	207,595,6	AKT1,CCND1,BRCA	68/334
13_Memb	GO Biological Pr	GO:19019	positive regulation of cell cycle phase transi	-7,81062	-5,927	207,595,9	AKT1,CCND1,CDC6,	27/119
13_Memb	GO Biological Pr	GO:19019	positive regulation of mitotic cell cycle phas	-7,70244	-5,829	207,595,9	AKT1,CCND1,CDC6,	24/98
13_Memb	GO Biological Pr	GO:19027	positive regulation of cell cycle G2/M phase	-7,27962	-5,437	595,993,1	CCND1,CDC25A,CD	13/32
13_Memb	GO Biological Pr	GO:00459	positive regulation of mitotic cell cycle	-7,10498	-5,274	207,595,6	AKT1,CCND1,BRCA	27/128
13_Memb	GO Biological Pr	GO:00109	positive regulation of G2/M transition of mi	-6,86961	-5,062	595,993,1	CCND1,CDC25A,CD	12/29
13_Memb	GO Biological Pr	GO:19028	positive regulation of cell cycle G1/S phase t	-2,95347	-1,684	207,990,2	AKT1,CDC6,EZH2,R	12/65
13_Memb	GO Biological Pr	GO:19000	positive regulation of G1/S transition of mit	-2,68754	-1,474	207,990,6	AKT1,CDC6,RRM1,	10/52
14_Summ	Reactome Gene	R-HSA-453	Mitotic G2-G2/M phases	-16,5582	-13,975	993,1017,	CDC25A,CDK2,E2F1	67/-
14_Memb	Reactome Gene	R-HSA-453	Mitotic G2-G2/M phases	-16,5582	-13,975	993,1017,	CDC25A,CDK2,E2F1	51/198
14_Memb	Reactome Gene	R-HSA-692	G2/M Transition	-16,0601	-13,506	993,1017,	CDC25A,CDK2,HSP	50/196
14_Memb	Reactome Gene	R-HSA-562	Anchoring of the basal body to the plasma r	-15,5111	-13,016	3320,4218	HSP90AA1,RAB8A,I	34/98
14_Memb	Reactome Gene	R-HSA-380	Recruitment of NuMA to mitotic centrosom	-14,3702	-11,953	3320,4751	HSP90AA1,NEK2,O	32/94
14_Memb	Reactome Gene	R-HSA-256	Regulation of PLK1 Activity at G2/M Transiti	-13,5352	-11,176	3320,4218	HSP90AA1,RAB8A,I	30/88
14_Memb	Reactome Gene	R-HSA-380	Loss of Nlp from mitotic centrosomes	-12,8324	-10,550	3320,4751	HSP90AA1,NEK2,O	26/70
14_Memb	Reactome Gene	R-HSA-380	Loss of proteins required for interphase mic	-12,8324	-10,550	3320,4751	HSP90AA1,NEK2,O	26/70
14_Memb	Reactome Gene	R-HSA-380	Recruitment of mitotic centrosome proteins	-12,6999	-10,429	3320,4751	HSP90AA1,NEK2,O	28/82
14_Memb	Reactome Gene	R-HSA-380	Centrosome maturation	-12,6999	-10,429	3320,4751	HSP90AA1,NEK2,O	28/82
14_Memb	Reactome Gene	R-HSA-885	AURKA Activation by TPX2	-12,3337	-10,088	3320,4751	HSP90AA1,NEK2,O	26/73
14_Memb	Reactome Gene	R-HSA-561	Cilium Assembly	-9,3819	-7,393	3320,4218	HSP90AA1,RAB8A,I	40/201
14_Memb	Reactome Gene	R-HSA-185	Organelle biogenesis and maintenance	-7,5456	-5,685	808,3320,	CALM3,HSP90AA1,	47/296
15_Summ	KEGG Pathway	hsa05168	Herpes simplex virus 1 infection	-15,6282	-13,114	207,317,3	AKT1,APAF1,FAS,B	88/-
15_Memb	KEGG Pathway	hsa05168	Herpes simplex virus 1 infection	-15,6282	-13,114	207,317,3	AKT1,APAF1,FAS,B	88/512
16_Summ	Reactome Gene	R-HSA-370	Transcriptional Regulation by TP53	-15,2118	-12,740	207,317,3	AKT1,APAF1,FAS,B	92/-
16_Memb	Reactome Gene	R-HSA-370	Transcriptional Regulation by TP53	-15,2118	-12,740	207,317,3	AKT1,APAF1,FAS,B	70/362
16_Memb	Canonical Pathw	M145	PID P53 DOWNSTREAM PATHWAY	-12,9008	-10,611	317,355,4	APAF1,FAS,ATF3,B	37/137
16_Memb	WikiPathways	WP4963	p53 transcriptional gene network	-8,58722	-6,652	136,317,3	ADORA2B,APAF1,F	25/96
17_Summ	GO Biological Pr	GO:00062	regulation of DNA replication	-15,2004	-12,735	641,990,1	BLM,CDC6,CDK2,D	38/-
17_Memb	GO Biological Pr	GO:00062	regulation of DNA replication	-15,2004	-12,735	641,990,1	BLM,CDC6,CDK2,D	38/124
17_Memb	GO Biological Pr	GO:00903	regulation of DNA-templated DNA replicatio	-12,3641	-10,114	641,1017,	BLM,CDK2,MCM3,	21/47
17_Memb	GO Biological Pr	GO:00457	positive regulation of DNA replication	-7,15377	-5,318	1017,1763	CDK2,DNA2,GLI2,P	14/38
17_Memb	GO Biological Pr	GO:20001	positive regulation of DNA-templated DNA r	-2,96713	-1,687	1017,8317	CDK2,CDC7,DBF4,D	5/13
17_Memb	GO Biological Pr	GO:00105	positive regulation of nuclear cell cycle DNA	-2,29267	-1,159	8317,1092	CDC7,DBF4,DBF4B	3/6
17_Memb	GO Biological Pr	GO:00332	regulation of nuclear cell cycle DNA replicat	-2,06093	-0,984	8317,1092	CDC7,DBF4,TIPIN,D	4/13
18_Summ	GO Biological Pr	GO:00323	DNA geometric change	-14,6602	-12,217	641,1663,	BLM,DDX11,DNA2,	47/-
18_Memb	GO Biological Pr	GO:00323	DNA geometric change	-14,6602	-12,217	641,1663,	BLM,DDX11,DNA2,	30/81
18_Memb	GO Biological Pr	GO:00711	DNA conformation change	-14,26	-11,858	641,1663,	BLM,DDX11,DNA2,	31/89
18_Memb	GO Biological Pr	GO:00332	nuclear DNA replication	-13,7684	-11,390	641,675,1	BLM,BRCA2,DNA2,	17/26
18_Memb	GO Biological Pr	GO:00447	cell cycle DNA replication	-13,365	-11,027	641,675,1	BLM,BRCA2,DNA2,	17/27
18_Memb	GO Biological Pr	GO:00062	DNA strand elongation involved in DNA repl	-13,3313	-10,998	1763,3978	DNA2,LIG1,MCM3,	13/15
18_Memb	GO Biological Pr	GO:00226	DNA strand elongation	-12,0533	-9,821	1763,3978	DNA2,LIG1,MCM3,	14/20
18_Memb	GO Biological Pr	GO:00325	DNA duplex unwinding	-12,0177	-9,792	641,1663,	BLM,DDX11,DNA2,	26/75
18_Memb	GO Biological Pr	GO:19029	mitotic DNA replication	-8,87179	-6,923	675,3978,	BRCA2,LIG1,MCM3	10/14
18_Memb	Reactome Gene	R-HSA-176	Unwinding of DNA	-8,32467	-6,416	4172,4173	MCM3,MCM4,MCM	9/12
18_Memb	GO Biological Pr	GO:00062	DNA unwinding involved in DNA replication	-7,66641	-5,799	641,4172,	BLM,MCM3,MCM4	11/21
18_Memb	GO Biological Pr	GO:00007	double-strand break repair via break-induce	-5,46402	-3,811	4172,4173	MCM3,MCM4,MCM	7/12
18_Memb	CORUM	CORUM:67	GINS complex	-4,70322	-3,149	9837,5165	GINS1,GINS2,GINS	4/4
18_Memb	GO Biological Pr	GO:19022	cell cycle DNA replication initiation	-4,02804	-2,560	4172,4173	MCM3,MCM4,POL	4/5
18_Memb	GO Biological Pr	GO:19023	nuclear cell cycle DNA replication initiation	-4,02804	-2,560	4172,4173	MCM3,MCM4,POL	4/5
18_Memb	GO Biological Pr	GO:19029	mitotic DNA replication initiation	-4,02804	-2,560	4172,4173	MCM3,MCM4,POL	4/5
18_Memb	GO Biological Pr	GO:00301	regulation of DNA-templated DNA replicatio	-3,58508	-2,175	1017,4172	CDK2,MCM3,MCM	6/15
18_Memb	CORUM	CORUM:38	MCM complex	-2,29267	-1,159	4172,4173	MCM3,MCM4,MCM	3/6
19_Summ	Canonical Pathw	M40	PID E2F PATHWAY	-14,1849	-11,788	317,672,8	APAF1,BRCA1,CASP	34/-

19_Memb	Canonical Pathw	M40	PID E2F PATHWAY	-14,1849	-11,788	317,672,8	APAF1,BRCA1,CAS	28/73
19_Memb	Reactome Gene	R-HSA-692	G1/S-Specific Transcription	-10,442	-8,358	990,1719,	CDC6,DHFR,E2F1,O	15/28
19_Memb	Reactome Gene	R-HSA-153	G0 and Early G1	-6,26137	-4,507	990,993,1	CDC6,CDC25A,CDK	11/27
19_Memb	Reactome Gene	R-HSA-136	Transcription of E2F targets under negative	-4,82119	-3,251	990,993,1	CDC6,CDC25A,E2F1	8/19
19_Memb	Reactome Gene	R-HSA-136	Transcription of E2F targets under negative	-3,40617	-2,033	1869,4605	E2F1,MYBL2,RBL1,	6/16
19_Memb	CORUM	CORUM:5	LINC complex, S-phase	-3,23035	-1,898	4605,5933	MYBL2,RBL1,LIN54	4/7
19_Memb	Reactome Gene	R-HSA-156	Polo-like kinase mediated events	-2,50963	-1,335	993,4605,	CDC25A,MYBL2,W	5/16
20_Summa	GO Biological Pr	GO:00002	microtubule cytoskeleton organization	-13,8429	-11,455	672,675,1	BRCA1,BRCA2,CDK	114/-
20_Memb	GO Biological Pr	GO:00002	microtubule cytoskeleton organization	-13,8429	-11,455	672,675,1	BRCA1,BRCA2,CDK	89/557
20_Memb	GO Biological Pr	GO:01406	non-membrane-bounded organelle assembl	-11,5263	-9,335	1017,1060	CDK2,CENPC,CFL2,	60/337
20_Memb	GO Biological Pr	GO:00070	spindle organization	-8,99664	-7,038	2316,3619	FLNA,INCENP,KIF1	35/166
20_Memb	GO Biological Pr	GO:00512	spindle assembly	-5,49701	-3,842	2316,3619	FLNA,INCENP,KIF1	21/102
20_Memb	GO Biological Pr	GO:00070	mitotic spindle organization	-3,77133	-2,335	2316,3619	FLNA,INCENP,KIF1	17/95
20_Memb	GO Biological Pr	GO:19028	microtubule cytoskeleton organization invol	-3,40472	-2,033	2316,3619	FLNA,INCENP,KIF1	20/130
20_Memb	GO Biological Pr	GO:00903	mitotic spindle assembly	-2,89287	-1,638	2316,3619	FLNA,INCENP,KIF1	10/49

Supplementary file S3

GroupID	Category	Term	Description	LogP	Log(q-valu	Genes	Symbols	InTerm_InList
2_Summa	GO Biologi	GO:00069	autophagy	-33,5512	-29,506	310,738,2	ANXA7,VPS51	162/-
2_Membe	GO Biologi	GO:00069	autophagy	-33,5512	-29,506	310,738,2	ANXA7,VPS51	102/316
2_Membe	GO Biologi	GO:00619	process utilizing autophagic mechanism	-33,5512	-29,506	310,738,2	ANXA7,VPS51	102/316
3_Summa	GO Biologi	GO:00301	protein catabolic process	-32,9232	-29,054	338,867,1	APOB,CBL,CL	294/-
3_Membe	GO Biologi	GO:00301	protein catabolic process	-32,9232	-29,054	338,867,1	APOB,CBL,CL	163/707
3_Membe	GO Biologi	GO:00516	proteolysis involved in protein catabol	-30,5339	-26,789	867,1499,	CBL,CTNNB1,	142/593
4_Summa	Reactome	R-HSA-199	Membrane Trafficking	-29,9813	-26,334	338,377,3	APOB,ARF3,A	147/-
4_Membe	Reactome	R-HSA-199	Membrane Trafficking	-29,9813	-26,334	338,377,3	APOB,ARF3,A	147/634
3_Membe	GO Biologi	GO:00199	modification-dependent protein catab	-27,8446	-24,276	867,1499,	CBL,CTNNB1,	128/531
3_Membe	GO Biologi	GO:00065	ubiquitin-dependent protein catabolic	-27,5576	-24,056	867,1499,	CBL,CTNNB1,	126/521
3_Membe	GO Biologi	GO:00436	modification-dependent macromolecu	-27,493	-24,050	867,1499,	CBL,CTNNB1,	129/542
4_Membe	Reactome	R-HSA-565	Vesicle-mediated transport	-27,0721	-23,680	338,377,3	APOB,ARF3,A	147/673
3_Membe	GO Biologi	GO:00706	protein modification by small protein c	-25,9732	-22,627	331,867,1	XIAP,CBL,CTN	161/792
5_Summa	GO Biologi	GO:00068	intracellular protein transport	-25,2217	-21,917	377,382,6	ARF3,ARF6,B	220/-
5_Membe	GO Biologi	GO:00068	intracellular protein transport	-25,2217	-21,917	377,382,6	ARF3,ARF6,B	147/700
6_Summa	GO Biologi	GO:00161	endosomal transport	-22,6694	-19,402	382,738,7	ARF6,VPS51,T	83/-
6_Membe	GO Biologi	GO:00161	endosomal transport	-22,6694	-19,402	382,738,7	ARF6,VPS51,T	73/238
7_Summa	GO Biologi	GO:00610	membrane organization	-22,4154	-19,183	21,24,291,	ABCA3,ABCA4	200/-
7_Membe	GO Biologi	GO:00610	membrane organization	-22,4154	-19,183	21,24,291,	ABCA3,ABCA4	149/760
3_Membe	GO Biologi	GO:00165	protein ubiquitination	-22,0405	-18,840	331,867,1	XIAP,CBL,CTN	128/609
3_Membe	GO Biologi	GO:00324	protein modification by small protein c	-21,7961	-18,629	331,867,1	XIAP,CBL,CTN	138/687
7_Membe	GO Biologi	GO:00102	endomembrane system organization	-21,7713	-18,629	523,533,5	ATP6V1A,ATP	120/555
2_Membe	GO Biologi	GO:00162	macroautophagy	-21,6509	-18,535	3162,3964	HMOX1,LGAL	65/201
5_Membe	GO Biologi	GO:00333	protein localization to organelle	-20,742	-17,672	382,583,7	ARF6,BBS2,PT	143/743
8_Summa	GO Biologi	GO:00105	regulation of autophagy	-20,7394	-17,672	291,523,5	SLC25A4,ATP	160/-
8_Membe	GO Biologi	GO:00105	regulation of autophagy	-20,7394	-17,672	291,523,5	SLC25A4,ATP	89/355
7_Membe	GO Biologi	GO:00160	vesicle organization	-20,6049	-17,559	523,528,5	ATP6V1A,ATP	86/337
9_Summa	GO Biologi	GO:00516	localization within membrane	-20,0977	-17,073	382,583,7	ARF6,BBS2,VF	117/-
9_Membe	GO Biologi	GO:00516	localization within membrane	-20,0977	-17,073	382,583,7	ARF6,BBS2,VF	117/558
2_Membe	KEGG Path	hsa04140	Autophagy - animal	-19,8765	-16,872	1508,1514	CTSB,CTSL,ER	56/165
10_Summ	GO Biologi	GO:00346	ncRNA metabolic process	-19,5361	-16,551	283,661,8	ANG,POLR3D,	155/-
10_Memb	GO Biologi	GO:00346	ncRNA metabolic process	-19,5361	-16,551	283,661,8	ANG,POLR3D,	112/530
11_Summ	GO Biologi	GO:00481	Golgi vesicle transport	-19,3732	-16,407	377,583,7	ARF3,BBS2,VF	89/-
11_Memb	GO Biologi	GO:00481	Golgi vesicle transport	-19,3732	-16,407	377,583,7	ARF3,BBS2,VF	75/280
8_Membe	GO Biologi	GO:00162	regulation of macroautophagy	-19,2076	-16,259	291,523,5	SLC25A4,ATP	54/159
12_Summ	GO Biologi	GO:00516	organelle localization	-17,8151	-14,884	583,867,1	BBS2,CBL,CDK	116/-
12_Memb	GO Biologi	GO:00516	organelle localization	-17,8151	-14,884	583,867,1	BBS2,CBL,CDK	110/542
13_Summ	Reactome	R-HSA-556	Metabolism of lipids	-17,7813	-14,866	30,37,211,	ACAA1,ACAD	206/-
13_Memb	Reactome	R-HSA-556	Metabolism of lipids	-17,7813	-14,866	30,37,211,	ACAA1,ACAD	136/743
14_Summ	GO Biologi	GO:00320	regulation of TOR signaling	-17,1124	-14,213	369,1609,	ARAF,DGKQ,H	85/-
14_Memb	GO Biologi	GO:00320	regulation of TOR signaling	-17,1124	-14,213	369,1609,	ARAF,DGKQ,H	49/147
15_Summ	GO Biologi	GO:00070	vacuole organization	-16,56	-13,676	53,523,53	ACP2,ATP6V1	56/-
15_Memb	GO Biologi	GO:00070	vacuole organization	-16,56	-13,676	53,523,53	ACP2,ATP6V1	56/191
16_Summ	GO Biologi	GO:00065	peptide metabolic process	-16,1988	-13,329	833,1203,	CARS1,CLN5,	131/-
16_Memb	GO Biologi	GO:00065	peptide metabolic process	-16,1988	-13,329	833,1203,	CARS1,CLN5,	103/516
9_Membe	GO Biologi	GO:00726	protein localization to membrane	-16,0893	-13,234	583,784,8	BBS2,CACNB3	97/473
17_Summ	GO Biologi	GO:00070	vacuolar transport	-15,8422	-13,001	738,757,9	VPS51,TMEM	57/-
17_Memb	GO Biologi	GO:00070	vacuolar transport	-15,8422	-13,001	738,757,9	VPS51,TMEM	51/168
3_Membe	GO Biologi	GO:00104	proteasomal protein catabolic process	-15,7312	-12,903	1499,3093	CTNNB1,UBE2	84/384
3_Membe	Reactome	R-HSA-983	Class I MHC mediated antigen processi	-15,4412	-12,626	1514,1535	CTSL,CYBA,FG	83/381
5_Membe	GO Biologi	GO:00066	protein targeting	-15,4009	-12,598	738,950,1	VPS51,SCARB	66/265
10_Memb	GO Biologi	GO:00344	ncRNA processing	-15,1772	-12,409	1653,1654	DDX1,DDX3X,	88/421
16_Memb	GO Biologi	GO:00430	peptide biosynthetic process	-15,1772	-12,409	833,1654,	CARS1,DDX3X	88/421
16_Memb	GO Biologi	GO:00064	translation	-15,1758	-12,409	833,1654,	CARS1,DDX3X	84/392
3_Membe	GO Biologi	GO:00431	proteasome-mediated ubiquitin-depen	-15,1276	-12,372	1499,3093	CTNNB1,UBE2	78/350
18_Summ	GO Biologi	GO:00801	regulation of cellular response to stres	-14,9701	-12,226	331,754,9	XIAP,PTTG1IP	184/-
18_Memb	GO Biologi	GO:00801	regulation of cellular response to stres	-14,9701	-12,226	331,754,9	XIAP,PTTG1IP	118/655
19_Summ	Reactome	R-HSA-566	Diseases of signal transduction by grow	-14,8777	-12,144	369,391,6	ARAF,RHOG,B	113/-
19_Memb	Reactome	R-HSA-566	Diseases of signal transduction by grow	-14,8777	-12,144	369,391,6	ARAF,RHOG,B	89/433

8_Membe	GO Biologi	GO:00313	regulation of cellular catabolic process	-14,8552	-12,132	291,405,5	SLC25A4,ARN	126/722
3_Membe	Reactome	R-HSA-983	Antigen processing: Ubiquitination & P	-14,55	-11,837	3093,5693	UBE2K,PSMB	71/309
16_Memb	GO Biologi	GO:00436	amide biosynthetic process	-14,5361	-11,833	384,427,8	ARG2,ASAH1,	103/545
20_Summ	GO Biologi	GO:19025	negative regulation of intracellular sign	-14,4377	-11,744	468,604,7	ATF4,BCL6,PT	108/-
20_Memb	GO Biologi	GO:19025	negative regulation of intracellular sign	-14,4377	-11,744	468,604,7	ATF4,BCL6,PT	108/586
12_Memb	GO Biologi	GO:00516	establishment of vesicle localization	-13,3918	-10,744	583,1453,	BBS2,CSNK1D	48/172
8_Membe	GO Biologi	GO:00105	positive regulation of autophagy	-13,1767	-10,555	291,2308,	SLC25A4,FOX	44/150
2_Membe	Reactome	R-HSA-961	Autophagy	-13,0634	-10,449	1457,1459	CSNK2A1,CSN	44/151
8_Membe	GO Biologi	GO:00098	positive regulation of catabolic process	-12,9842	-10,378	291,405,7	SLC25A4,ARN	105/591
12_Memb	GO Biologi	GO:00516	vesicle localization	-12,7042	-10,118	583,1453,	BBS2,CSNK1D	49/185
10_Memb	GO Biologi	GO:00422	ribosome biogenesis	-12,6468	-10,078	1654,1964	DDX3X,EIF1A	68/314
2_Membe	GO Biologi	GO:19030	organelle disassembly	-12,2936	-9,767	1939,2011	EIF2D,MARK2	35/106
2_Membe	GO Biologi	GO:00224	cellular component disassembly	-12,1034	-9,596	1939,2011	EIF2D,MARK2	68/322
10_Memb	GO Biologi	GO:00226	ribonucleoprotein complex biogenesis	-11,9993	-9,504	1653,1654	DDX1,DDX3X,	90/491
13_Memb	GO Biologi	GO:00086	lipid biosynthetic process	-11,6982	-9,215	221,283,4	ALDH3B1,AN	102/594
3_Membe	Reactome	R-HSA-895	Neddylation	-11,5304	-9,065	3091,4780	HIF1A,NFE2L2	56/245
9_Membe	GO Biologi	GO:00901	establishment of protein localization to	-11,4731	-9,013	583,819,1	BBS2,CAMLG,	54/232
2_Membe	Reactome	R-HSA-163	Macroautophagy	-11,4219	-8,973	1457,1459	CSNK2A1,CSN	39/136
14_Memb	GO Biologi	GO:19034	regulation of TORC1 signaling	-11,0927	-8,683	1609,4287	DGKQ,ATXN3,	31/93
3_Membe	Reactome	R-HSA-128	Adaptive Immune System	-11,0903	-8,683	830,912,1	CAPZA2,CD1D	121/769
8_Membe	GO Biologi	GO:00162	positive regulation of macroautophagy	-10,6359	-8,288	291,2773,	SLC25A4,GNA	27/75
14_Memb	KEGG Path	hsa04150	mTOR signaling pathway	-10,634	-8,288	523,528,1	ATP6V1A,ATP	41/156
5_Membe	GO Biologi	GO:00725	establishment of protein localization to	-10,3178	-8,024	754,867,9	PTTG1IP,CBL,	68/351
13_Memb	GO Biologi	GO:00066	phospholipid metabolic process	-10,2609	-7,975	21,1609,2	ABCA3,DGKQ,	68/352
6_Membe	GO Biologi	GO:00164	cytosolic transport	-10,2213	-7,943	738,1203,	VPS51,CLN5,F	40/154
14_Memb	GO Biologi	GO:00320	negative regulation of TOR signaling	-9,99494	-7,742	3091,4287	HIF1A,ATXN3,	26/74
17_Memb	GO Biologi	GO:00726	protein localization to vacuole	-9,47954	-7,291	950,5289,	SCARB2,PIK3C	27/83
2_Membe	GO Biologi	GO:00004	autophagy of mitochondrion	-9,4694	-7,287	2011,8678	MARK2,BECN	22/57
2_Membe	GO Biologi	GO:00617	mitochondrion disassembly	-9,4694	-7,287	2011,8678	MARK2,BECN	22/57
12_Memb	GO Biologi	GO:00069	vesicle budding from membrane	-9,41659	-7,237	1453,6399	CSNK1D,TRAP	26/78
12_Memb	GO Biologi	GO:00069	vesicle targeting	-9,14407	-6,996	1453,5663	CSNK1D,PSEN	23/64
12_Memb	GO Biologi	GO:00516	establishment of organelle localization	-9,12786	-6,986	583,867,1	BBS2,CBL,CSN	72/405
16_Memb	Reactome	R-HSA-727	Translation	-8,89245	-6,774	833,1936,	CARS1,EEF1D,	57/292
13_Memb	GO Biologi	GO:00086	phospholipid biosynthetic process	-8,87866	-6,763	1609,3612	DGKQ,IMPA1,	50/240
2_Membe	GO Biologi	GO:00000	autophagosome assembly	-8,80867	-6,698	4534,5289	MTM1,PIK3C	25/77
13_Memb	GO Biologi	GO:00464	glycerolipid metabolic process	-8,79404	-6,686	21,283,33	ABCA3,ANG,A	64/348
10_Memb	GO Biologi	GO:00160	rRNA metabolic process	-8,77299	-6,672	283,2976,	ANG,GTF3C2,	51/249
11_Memb	GO Biologi	GO:00068	endoplasmic reticulum to Golgi vesicle-	-8,75398	-6,658	1453,6399	CSNK1D,TRAP	33/125
2_Membe	GO Biologi	GO:19050	autophagosome organization	-8,62965	-6,541	4534,5289	MTM1,PIK3C	26/84
15_Memb	GO Biologi	GO:00070	lysosome organization	-8,58178	-6,505	53,523,53	ACP2,ATP6V1	29/102
15_Memb	GO Biologi	GO:00801	lytic vacuole organization	-8,58178	-6,505	53,523,53	ACP2,ATP6V1	29/102
8_Membe	GO Biologi	GO:00313	positive regulation of cellular catabolic	-8,49167	-6,417	291,405,1	SLC25A4,ARN	68/386
10_Memb	GO Biologi	GO:00063	rRNA processing	-8,41026	-6,345	4839,5303	NOP2,PIN4,R	46/218
13_Memb	GO Biologi	GO:00066	glycerophospholipid metabolic process	-8,2133	-6,181	21,1609,3	ABCA3,DGKQ,	54/281
14_Memb	Reactome	R-HSA-963	Amino acids regulate mTORC1	-8,1401	-6,112	523,528,5	ATP6V1A,ATP	20/55
13_Memb	GO Biologi	GO:00450	glycerolipid biosynthetic process	-7,87046	-5,858	283,1609,	ANG,DGKQ,IM	45/219
14_Memb	GO Biologi	GO:19042	negative regulation of TORC1 signaling	-7,8025	-5,794	4287,5562	ATXN3,PRKAA	18/47
9_Membe	GO Biologi	GO:19907	protein localization to cell periphery	-7,77141	-5,765	583,784,1	BBS2,CACNB3	48/243
6_Membe	GO Biologi	GO:00421	retrograde transport, endosome to Gol	-7,70622	-5,706	738,1203,	VPS51,CLN5,T	26/92
11_Memb	GO Biologi	GO:00070	Golgi organization	-7,6568	-5,664	738,1453,	VPS51,CSNK1	34/144
4_Membe	Reactome	R-HSA-681	Intra-Golgi and retrograde Golgi-to-ER	-7,54748	-5,566	377,738,8	ARF3,VPS51,C	42/202
2_Membe	GO Biologi	GO:00619	selective autophagy	-7,54128	-5,562	3964,5289	LGALS8,PIK3C	22/70
17_Memb	GO Biologi	GO:00726	establishment of protein localization to	-7,46424	-5,494	950,5289,	SCARB2,PIK3C	21/65
17_Memb	GO Biologi	GO:00070	lysosomal transport	-7,45477	-5,487	738,950,4	VPS51,SCARB	32/133
12_Memb	GO Biologi	GO:00990	vesicle tethering	-7,06703	-5,134	6399,7109	TRAPPC2,TRA	14/32
19_Memb	Reactome	R-HSA-568	MAPK family signaling cascades	-6,78584	-4,885	369,1741,	ARAF,DLG3,D	56/325
16_Memb	GO Biologi	GO:01400	mitochondrial gene expression	-6,74645	-4,854	2617,3396	GARS1,MRPL	32/142
13_Memb	GO Biologi	GO:00464	glycerophospholipid biosynthetic proce	-6,74456	-4,854	1609,3612	DGKQ,IMPA1,	40/200
13_Memb	GO Biologi	GO:00904	organophosphate biosynthetic process	-6,30154	-4,464	159,353,5	ADSS2,APRT,	80/544
12_Memb	GO Biologi	GO:00901	COPII-coated vesicle budding	-6,30114	-4,464	1453,6399	CSNK1D,TRAP	15/41
14_Memb	GO Biologi	GO:00320	positive regulation of TOR signaling	-6,27604	-4,442	5290,5292	PIK3CA,PIM1,	20/69

9_Membe	GO Biologi	GO:00726:	protein localization to plasma membra	-6,18263	-4,362	583,784,2:	BBS2,CACNB3	39/202	
17_Memb	GO Biologi	GO:00066:	protein targeting to vacuole	-6,04501	-4,246	950,5289,(	SCARB2,PIK3C	16/48	
19_Memb	Reactome	R-HSA-568	MAPK1/MAPK3 signaling	-5,95147	-4,167	369,1741,:	ARAF,DLG3,D	49/286	
13_Memb	GO Biologi	GO:00066	phosphatidylinositol biosynthetic proce	-5,86	-4,093	3612,4534	IMPA1,MTM1	28/126	
14_Memb	Reactome	R-HSA-971	Cellular response to starvation	-5,71971	-3,970	468,523,5:	ATF4,ATP6V1,	32/157	
18_Memb	GO Biologi	GO:00069	DNA damage response	-5,54834	-3,821	322,331,6(	APBB1,XIAP,B	100/755	
19_Memb	Reactome	R-HSA-567	RAF/MAP kinase cascade	-5,48492	-3,764	369,1741,:	ARAF,DLG3,D	47/280	
12_Memb	GO Biologi	GO:00481	vesicle targeting, to, from or within Go	-5,45707	-3,741	1453,6399	CSNK1D,TRAP	12/31	
12_Memb	GO Biologi	GO:00069	vesicle coating	-5,31211	-3,620	1453,6399	CSNK1D,TRAP	13/37	
2_Membe	KEGG Path	hsa04136	Autophagy - other	-5,28727	-3,599	3476,5289	IGBP1,PIK3C3	12/32	
16_Memb	GO Biologi	GO:00325	mitochondrial translation	-5,0455	-3,393	2617,3396	GARS1,MRPL!	24/109	
13_Memb	GO Biologi	GO:00464	phosphatidylinositol metabolic process	-4,89949	-3,272	3612,4534	IMPA1,MTM1	29/148	
10_Memb	GO Biologi	GO:00304	maturation of SSU-rRNA	-4,72503	-3,121	6234,8602	RPS28,NOP14	15/53	
16_Memb	Reactome	R-HSA-536	Mitochondrial translation	-4,1577	-2,651	3396,4528	MRPL58,MTIF	20/93	
2_Membe	GO Biologi	GO:00448	nucleophagy	-4,11836	-2,622	9140,9821	ATG12,RB1CC	8/19	
12_Memb	GO Biologi	GO:00482	vesicle targeting, rough ER to cis-Golgi	-4,09869	-2,605	1453,6399	CSNK1D,TRAP	9/24	
12_Memb	GO Biologi	GO:00482	COPII vesicle coating	-4,09869	-2,605	1453,6399	CSNK1D,TRAP	9/24	
10_Memb	Reactome	R-HSA-723	rRNA processing	-4,04791	-2,564	1453,4839	CSNK1D,NOP:	34/205	
10_Memb	Reactome	R-HSA-679	rRNA modification in the nucleus and c	-3,94956	-2,491	4839,8602	NOP2,NOP14,	15/61	
2_Membe	GO Biologi	GO:00344	protein localization to phagophore asse	-3,78771	-2,355	5289,1115	PIK3C3,WDR4	7/16	
14_Memb	GO Biologi	GO:19042	positive regulation of TORC1 signaling	-3,57623	-2,187	5292,6198	PIM1,RPS6KB	13/52	
17_Memb	GO Biologi	GO:00614	protein localization to lysosome	-3,31976	-1,972	950,5289,(	SCARB2,PIK3C	13/55	
18_Memb	GO Biologi	GO:00062	regulation of DNA repair	-3,31252	-1,966	1025,2140	CDK9,EYA3,H!	33/215	
10_Memb	GO Biologi	GO:00004	maturation of SSU-rRNA from tricistror	-3,26535	-1,926	8602,9790	NOP14,BMS1,	10/36	
12_Memb	CORUM	CORUM:6	TRAPP complex	-3,25096	-1,917	6399,7109	TRAPPC2,TRA	6/14	
10_Memb	Reactome	R-HSA-679	Major pathway of rRNA processing in t	-3,13402	-1,825	1453,6125	CSNK1D,RPL5	29/185	
16_Memb	Reactome	R-HSA-536	Mitochondrial translation initiation	-3,11444	-1,807	3396,4528	MRPL58,MTIF	17/87	
10_Memb	Reactome	R-HSA-886	rRNA processing in the nucleus and cyt	-3,0845	-1,785	1453,4839	CSNK1D,NOP:	30/195	
10_Memb	GO Biologi	GO:00422	ribosomal small subunit biogenesis	-3,03527	-1,745	6191,6206	RPS4X,RPS12,	19/104	
4_Membe	Reactome	R-HSA-885	Golgi-to-ER retrograde transport	-2,82227	-1,579	377,830,3	ARF3,CAPZA2	22/133	
20_Memb	GO Biologi	GO:00434	negative regulation of MAPK cascade	-2,81822	-1,576	1605,1843	DAG1,DUSP1,	27/176	
16_Memb	Reactome	R-HSA-541	Mitochondrial translation termination	-2,67712	-1,467	3396,5018	MRPL58,OXA:	16/87	
16_Memb	Reactome	R-HSA-538	Mitochondrial translation elongation	-2,26997	-1,165	3396,5018	MRPL58,OXA:	15/87	
1_Summa	CORUM	CORUM:4	VCP-VIMP-DERL1-DERL2-HRD1-SEL1L c	-2,01835	-0,976	55829,791	SELENOS,DER	313/-	
1_Membe	CORUM	CORUM:4	VCP-VIMP-DERL1-DERL2-HRD1-SEL1L c	-2,01835	-0,976	55829,791	SELENOS,DER	3/6	
1_Membe	CORUM	CORUM:6	CORVET complex	-2,01835	-0,976	23355,576	VPS8,VPS18,V	3/6	
1_Membe	CORUM	CORUM:6	HOPS complex	-2,01835	-0,976	27072,576	VPS41,VPS18,	3/6	
1_Membe	GO Biologi	GO:00064	protein sulfation	-2,01835	-0,976	8459,8460	TPST2,TPST1,I	3/6	
1_Membe	GO Biologi	GO:00065	leucine catabolic process	-2,01835	-0,976	549,3155,:	AUH,HMGCL,I	3/6	
1_Membe	GO Biologi	GO:00098	abscission	-2,01835	-0,976	9798,8493	IST1,ZFYVE19,	3/6	
1_Membe	GO Biologi	GO:00109	regulation of phosphate transport	-2,01835	-0,976	468,1051,:	ATF4,CEBPB,C	3/6	
1_Membe	GO Biologi	GO:00171	protein histidyl modification to diphtha	-2,01835	-0,976	1802,5161	DPH2,DPH5,D	3/6	
1_Membe	GO Biologi	GO:00183	protein geranylgeranylation	-2,01835	-0,976	1121,5229	CHM,PGGT1B	3/6	
1_Membe	GO Biologi	GO:00311	tRNA pseudouridine synthesis	-2,01835	-0,976	80324,834	PUS1,PUS3,RI	3/6	
1_Membe	GO Biologi	GO:00341	positive regulation of toll-like receptor	-2,01835	-0,976	1535,9258	CYBA,MFHAS:	3/6	
1_Membe	GO Biologi	GO:00381	interleukin-17A-mediated signaling pat	-2,01835	-0,976	6885,7189	MAP3K7,TRAI	3/6	
1_Membe	GO Biologi	GO:00140	regulation of gliogenesis	-2,01327	-0,971	1030,1499	CDKN2B,CTNI	17/110	
1_Membe	GO Biologi	GO:00163	dendrite development	-2,01327	-0,971	473,1398,:	RERE,CRK,DL(	17/110	
1_Membe	GO Biologi	GO:00217	developmental maturation	-2,01215	-0,971	283,473,5:	ANG,RERE,AT	35/278	
1_Membe	WikiPathw	WP4656	Joubert syndrome	-2,0088	-0,971	468,583,1(	ATF4,BBS2,CE	13/76	
1_Membe	GO Biologi	GO:00105	miRNA metabolic process	-2,00782	-0,971	27161,575	AGO2,XPO5,T	6/23	
1_Membe	GO Biologi	GO:00361	cellular response to platelet-derived gr	-2,00782	-0,971	867,2534,	CBL,FYN,TLR4	6/23	
1_Membe	GO Biologi	GO:00400	positive regulation of embryonic devel	-2,00782	-0,971	3170,5048	FOXA2,PAFAH	6/23	
1_Membe	GO Biologi	GO:00420	T-helper 1 type immune response	-2,00782	-0,971	3142,3566	HLX,IL4R,STA	6/23	
1_Membe	GO Biologi	GO:00704	interleukin-1-mediated signaling pathw	-2,00782	-0,971	1958,3656	EGR1,IRAK2,N	6/23	
1_Membe	GO Biologi	GO:00712	cellular response to misfolded protein	-2,00782	-0,971	4287,7917	ATXN3,BAG6,	6/23	
1_Membe	Reactome	R-HSA-373	Nephrin family interactions	-2,00782	-0,971	2534,4690	FYN,NCK1,PIK	6/23	
1_Membe	Reactome	R-HSA-450	JNK (c-Jun kinases) phosphorylation an	-2,00782	-0,971	3656,5609	IRAK2,MAP2K	6/23	
1_Membe	Reactome	R-HSA-562	BBSome-mediated cargo-targeting to c	-2,00782	-0,971	583,8195,	BBS2,MKKS,B	6/23	
1_Membe	Reactome	R-HSA-894	Mitochondrial calcium ion transport	-2,00782	-0,971	6687,7417	SPG7,VDAC2,I	6/23	
1_Membe	Reactome	R-HSA-901	RHOBTB1 GTPase cycle	-2,00782	-0,971	1627,6093	DBN1,ROCK1,	6/23	

1_Membe	Reactome	R-HSA-975	Regulation of Homotypic Cell-Cell Adhe	-2,00782	-0,971	1499,3728	CTNNB1,JUP,/	6/23
1_Membe	Reactome	R-HSA-976	Regulation of Expression and Function	-2,00782	-0,971	1499,3728	CTNNB1,JUP,/	6/23
1_Membe	WikiPathw	WP3612	Photodynamic therapy-induced NFE2L2	-2,00782	-0,971	1432,1728	MAPK14,NQC	6/23
1_Membe	WikiPathw	WP5046	NAD metabolism in oncogene-induced	-2,00782	-0,971	2539,2806	G6PD,GOT2,H	6/23
1_Membe	WikiPathw	WP4262	Breast cancer pathway	-2,00601	-0,971	369,1457,1	ARAF,CSNK2A	22/155
1_Membe	Reactome	R-HSA-240	Selenoamino acid metabolism	-2,00556	-0,971	875,1491,1	CBS,CTH,EPRS	18/119
1_Membe	CORUM	CORUM:51	Polycystin-1 multiprotein complex (ACT	-2,00381	-0,971	1499,3728	CTNNB1,JUP,I	4/11
1_Membe	CORUM	CORUM:63	anti-BHC110 complex	-2,00381	-0,971	7750,7764	ZMYM2,ZNF2	4/11
1_Membe	GO Biologi	GO:00000	sulfur amino acid catabolic process	-2,00381	-0,971	875,4357,4	CBS,MPST,MT	4/11
1_Membe	GO Biologi	GO:00065	cysteine metabolic process	-2,00381	-0,971	875,1491,1	CBS,CTH,GCLC	4/11
1_Membe	GO Biologi	GO:00068	manganese ion transport	-2,00381	-0,971	4891,2351	SLC11A2,SLC3	4/11
1_Membe	GO Biologi	GO:00093	tRNA transcription	-2,00381	-0,971	661,2976,9	POLR3D,GTF3	4/11
1_Membe	GO Biologi	GO:00183	protein prenylation	-2,00381	-0,971	1121,5229	CHM,PGGT1B	4/11
1_Membe	GO Biologi	GO:00301	proteoglycan catabolic process	-2,00381	-0,971	3423,3425	IDS,IDUA,SGS	4/11
1_Membe	GO Biologi	GO:00340	endosomal vesicle fusion	-2,00381	-0,971	23355,270	VPS8,VPS41,V	4/11
1_Membe	GO Biologi	GO:00349	mitochondrial protein processing	-2,00381	-0,971	4285,6687	MIPEP,SPG7,F	4/11
1_Membe	GO Biologi	GO:00356	TRIF-dependent toll-like receptor signa	-2,00381	-0,971	6885,7099	MAP3K7,TLR4	4/11
1_Membe	GO Biologi	GO:00604	trachea morphogenesis	-2,00381	-0,971	1499,5604	CTNNB1,MAP	4/11
1_Membe	GO Biologi	GO:00755	IRES-dependent viral translational initia	-2,00381	-0,971	1939,6741	EIF2D,SSB,CSD	4/11
1_Membe	GO Biologi	GO:00905	establishment of endothelial intestinal	-2,00381	-0,971	4301,9076	AFDN,CLDN1,	4/11
1_Membe	GO Biologi	GO:00973	prenylation	-2,00381	-0,971	1121,5229	CHM,PGGT1B	4/11
1_Membe	GO Biologi	GO:19000	negative regulation of cellular response	-2,00381	-0,971	2043,4780	EPHA4,NFE2L	4/11
1_Membe	GO Biologi	GO:19038	regulation of PERK-mediated unfolded	-2,00381	-0,971	4690,5771	NCK1,PTPN2,I	4/11
1_Membe	GO Biologi	GO:19044	regulation of GTP binding	-2,00381	-0,971	1203,2043	CLN5,EPHA4,I	4/11
1_Membe	GO Biologi	GO:19909	xenobiotic detoxification by transmem	-2,00381	-0,971	540,10786	ATP7B,SLC17A	4/11
1_Membe	Reactome	R-HSA-196	Biotin transport and metabolism	-2,00381	-0,971	686,3141,5	BTD,HLCS,PC,	4/11
1_Membe	Reactome	R-HSA-963	Constitutive Signaling by Overexpresse	-2,00381	-0,971	2885,3845	GRB2,KRAS,N	4/11
1_Membe	Reactome	R-HSA-966	Pexophagy	-2,00381	-0,971	4077,5830	NBR1,PEX5,SC	4/11
1_Membe	Reactome	R-HSA-975	SARS-CoV-2 modulates autophagy	-2,00381	-0,971	27072,576	VPS41,VPS18,	4/11
1_Membe	WikiPathw	WP3630	NAD metabolism, sirtuins and aging	-2,00381	-0,971	2308,3091	FOXO1,HIF1A	4/11
1_Membe	WikiPathw	WP4788	Autosomal recessive osteopetrosis path	-2,00381	-0,971	1186,7189	CLCN7,TRAF6	4/11
1_Membe	WikiPathw	WP5049	Glycolysis in senescence	-2,00381	-0,971	2539,3098	G6PD,HK1,LD	4/11
1_Membe	WikiPathw	WP5409	PAFAH1B1 copy number variation	-2,00381	-0,971	5048,5049	PAFAH1B1,PA	4/11
1_Membe	GO Biologi	GO:00059	glycogen metabolic process	-2,00247	-0,971	178,2992,2	AGL,GYG1,GY	10/52
1_Membe	GO Biologi	GO:00103	membrane invagination	-2,00247	-0,971	1182,8678	CLCN3,BECN1	10/52
1_Membe	GO Biologi	GO:00309	endoplasmic reticulum unfolded protei	-2,00247	-0,971	468,1491,1	ATF4,CTH,ERN	10/52
1_Membe	GO Biologi	GO:19028	positive regulation of miRNA transcript	-2,00247	-0,971	688,1958,1	KLF5,EGR1,FC	10/52
1_Membe	Canonical	M50	PID PTP1B PATHWAY	-2,00247	-0,971	1398,2534	CRK,FYN,GRB2	10/52
1_Membe	Reactome	R-HSA-242	IRS-related events triggered by IGF1R	-2,00247	-0,971	2260,2549	FGFR1,GAB1,C	10/52
1_Membe	KEGG Path	hsa05323	Rheumatoid arthritis	-2,00144	-0,970	523,528,53	ATP6V1A,ATP	15/93

Supplementary file S4

ID	NPHP1_DMSO_mean	NPHP1_EUP_mean	AveExpr	LogFC	deseq_adj_p	genename	symbol	
ENSG00000115353	3,301561926	9,578599815	1,871027879	-3,974803041	1,93E-07	tachykinin receptor 1	TACR1	RED:UP
ENSG00000039139	4,277073258	16,15024614	3,788060505	-2,291015524	0,000168705	dynein axonemal heavy chai	DNAH5	GREEN:DOWN
ENSG00000173805	5,967718315	21,30743015	5,009091468	-1,896043451	0,001574121	huntingtin associated protei	HAP1	
ENSG00000205795	6,974939415	25,33974176	4,028068685	-1,848306894	0,000563408	cystin 1	CYS1	
ENSG00000127928	0,273696102	0,604336222	-1,088564267	-1,830049616	0,012783611	G protein subunit gamma tra	GNGT1	
ENSG00000168589	0,115042583	0,36925832	-0,544626534	-1,705416156	0,01137632	dynein light chain roadblock	DYNLRB2	
ENSG00000198691	3,293079274	7,084853552	2,897642565	-1,567825876	0,001492475	ATP binding cassette subfam	ABCA4	
ENSG00000118997	0,715786557	1,844669856	1,98870986	-1,35868082	0,000125073	dynein axonemal heavy chai	DNAH7	
ENSG00000138771	146,5712838	351,5608615	8,676850266	-1,28521912	6,17E-10	shroom family member 3	SHROOM3	
ENSG00000180638	0,391823303	0,863157217	0,800048731	-1,13255946	0,036662279	solute carrier family 47 mem	SLC47A2	
ENSG00000204052	0,639726293	1,242478213	1,261638764	-0,960545186	0,033373691	leucine rich repeat containir	LRRC73	
ENSG00000135407	3,334022537	6,207470004	3,744953211	-0,945430707	0,003619906	advillin	AVIL	
ENSG00000185920	8,669905018	15,70238557	5,123759721	-0,927759568	0,000359322	patched 1	PTCH1	
ENSG00000153208	10,34148211	18,14854066	5,173112394	-0,901481536	0,005696942	MER proto-oncogene, tyrosi	MERTK	
ENSG00000167646	1,580931076	2,829544403	2,43822008	-0,859315765	0,005078631	dynein axonemal assembly f	DNAAF3	
ENSG00000088727	1,637474879	2,885942606	2,624400527	-0,814513426	5,27E-06	kinesin family member 9	KIF9	
ENSG00000130363	42,53848609	71,99699749	7,196981294	-0,770080645	4,30E-09	radial spoke head 3	RSPH3	
ENSG00000157796	34,2945428	57,90186348	7,031103167	-0,750732301	7,51E-15	WD repeat domain 19	WDR19	
ENSG00000181004	6,867128782	11,54555267	4,865789872	-0,748088397	1,10E-06	Bardet-Biedl syndrome 12	BBS12	
ENSG00000143801	12,45691976	20,47950752	5,522553648	-0,720899149	7,73E-06	presenilin 2	PSEN2	
ENSG00000048342	17,73536864	27,74420259	5,839628926	-0,720056355	0,000786313	coiled-coil and C2 domain cc	CC2D2A	
ENSG00000186952	0,945075534	1,498525297	1,742328105	-0,717745135	0,020562036	transmembrane protein 232	TMEM232	
ENSG00000127328	25,09280764	41,02568433	6,19857397	-0,708629801	7,27E-11	RAB3A interacting protein	RAB3IP	
ENSG00000084764	6,068108815	9,087327781	4,691615717	-0,703939497	0,002062061	microtubule associated prot	MAPRE3	
ENSG00000046651	22,34930892	33,48282691	5,995671826	-0,669705873	0,00038284	OFD1 centriole and centriola	OFD1	
ENSG00000114841	7,717421443	12,21987369	5,057025889	-0,665730307	0,000149419	dynein axonemal heavy chai	DNAH1	
ENSG00000100644	734,8499123	1109,79753	11,31264585	-0,602254661	1,91E-22	hypoxia inducible factor 1 su	HIF1A	
ENSG00000113966	6,691996167	10,15986285	4,564745947	-0,598699558	1,98E-05	ADP ribosylation factor like (	ARL6	
ENSG00000177042	5,729191015	8,524100238	4,324048134	-0,570608818	0,026164504	transmembrane protein 80	TMEM80	
ENSG00000161011	455,3990549	673,5377698	10,84303762	-0,560688156	1,13E-06	sequestosome 1	SQSTM1	
ENSG00000138190	20,50899748	30,02012853	5,859432476	-0,554960481	8,25E-06	exocyst complex component	EXOC6	
ENSG00000166900	46,95853975	68,55707565	7,275842786	-0,553564737	0,000582812	syntaxin 3	STX3	
ENSG00000095303	49,35037821	70,42888466	7,649316385	-0,548197537	0,018750608	prostaglandin-endoperoxide	PTGS1	
ENSG00000111254	0,885520749	1,263638064	2,052099955	-0,547617687	0,037494409	A-kinase anchoring protein 3	AKAP3	
ENSG00000127948	73,86062102	108,1319693	8,135615912	-0,546577862	1,87E-07	cytochrome p450 oxidoredu	POR	
ENSG00000179941	19,54830601	28,44507968	6,195515786	-0,545738025	2,30E-14	Bardet-Biedl syndrome 10	BBS10	
ENSG00000138036	9,311476978	13,43093798	5,0126612	-0,54389605	0,005473267	dynein cytoplasmic 2 light in	DYNC2LI1	
ENSG00000180758	5,379882667	7,81849153	4,320612167	-0,540312785	0,006317113	G protein-coupled receptor	GPR157	
ENSG00000174007	10,08166589	14,43281986	4,92061192	-0,51667068	0,010718878	centrosomal protein 19	CEP19	
ENSG00000080815	111,3082683	158,75798	8,635881715	-0,514351541	8,11E-10	presenilin 1	PSEN1	
ENSG00000140941	92,33437825	129,6736889	8,37167567	-0,504114798	1,94E-11	microtubule associated prot	MAP1LC3B	
ENSG00000151575	10,33074067	14,44541403	5,162150316	-0,49149683	0,015087786	testis expressed 9	TEX9	
ENSG00000068796	89,00052172	123,8087204	8,021620193	-0,491214619	0,032091649	kinesin family member 2A	KIF2A	
ENSG00000113971	5,416155542	7,628805161	4,209436751	-0,49032299	0,002728632	nephrocystin 3	NPHP3	
ENSG00000174373	29,28498566	40,88174251	6,616335726	-0,482952661	0,000172534	Ral GTPase activating protei	RALGAPA1	
ENSG00000089091	3,618471915	5,010261144	3,780412723	-0,463803488	0,002451829	double zinc ribbon and anky	DZANK1	
ENSG00000175970	73,98312353	101,3554285	8,01014361	-0,455638694	0,000129325	unc-119 lipid binding chaper	UNC119B	
ENSG00000164114	28,34282883	38,70477209	6,77320319	-0,452036575	0,002075237	microtubule associated prot	MAP9	
ENSG00000137942	101,763989	136,1691549	8,289865017	-0,439939619	0,032057444	formin binding protein 1 like	FNBP1L	
ENSG00000105948	10,73950673	14,56070393	5,082380946	-0,43708191	2,13E-05	tetratricopeptide repeat dor	TTC26	
ENSG00000100592	27,80166988	37,42417508	6,227083039	-0,433480461	0,000253451	dishevelled associated activa	DAAM1	
ENSG00000119661	11,46864854	15,47396002	5,330586916	-0,427573049	0,000205424	dynein axonemal light chain	DNAL1	
ENSG00000032742	14,62063387	19,60043477	5,74007497	-0,419156819	5,78E-05	intraflagellar transport 88	IFT88	
ENSG00000187535	47,47192979	62,94191641	7,460265224	-0,417263289	0,004175389	intraflagellar transport 140	IFT140	
ENSG00000141013	30,01729429	39,99082123	6,525114068	-0,412979197	0,001080224	growth arrest specific 8	GAS8	
ENSG00000118965	25,23972296	33,65758791	6,457498517	-0,411247989	4,82E-05	WD repeat domain 35	WDR35	
ENSG00000101350	81,30711563	107,5075483	8,116507408	-0,400136304	1,50E-05	kinesin family member 3B	KIF3B	
ENSG00000084731	74,50856661	97,03811314	7,903914087	-0,397819932	0,001905789	kinesin family member 3C	KIF3C	
ENSG00000176101	39,20370029	51,69261966	7,122378445	-0,397071555	3,64E-06	SS nuclear autoantigen 1	SSNA1	
ENSG00000125124	57,95546878	76,21110115	7,448810434	-0,393751055	5,79E-05	Bardet-Biedl syndrome 2	BBS2	
ENSG00000089289	44,00140252	57,35892207	7,400498232	-0,386611739	0,007068426	immunoglobulin binding pro	IGBP1	
ENSG00000134874	113,8619641	147,9980767	8,457133593	-0,384042623	0,030658854	DAZ interacting zinc finger p	DZIP1	
ENSG00000131437	19,259151	25,17270613	6,103168001	-0,381543894	0,000393279	kinesin family member 3A	KIF3A	
ENSG00000100379	45,75662909	59,32111141	7,368994369	-0,374649651	0,000481781	potassium channel tetramer	KCTD17	
ENSG00000156345	6,706374564	8,700599606	4,805676472	-0,372034022	0,030679438	cyclin dependent kinase 20	CDK20	
ENSG00000155097	64,93394808	83,65601798	7,751304675	-0,362249682	9,47E-08	ATPase H+ transporting V1 s	ATP6V1C1	
ENSG00000163686	14,30670354	18,37900128	5,575684748	-0,360476101	0,032348721	abhydrolase domain contain	ABHD6	
ENSG00000166454	75,96508166	97,4594515	7,991202405	-0,360000523	5,50E-11	ATM interactor	ATMIN	
ENSG00000100271	13,23539933	16,89276669	5,621482584	-0,357515867	0,002629329	TTL family tubulin polyglutar	TTLL1	
ENSG00000114302	92,06929891	116,2057988	8,270644689	-0,337100746	0,006240601	protein kinase cAMP-depen	PRKAR2A	
ENSG00000110367	215,766415	271,4236426	9,532440655	-0,332456411	0,004267759	DEAD-box helicase 6	DDX6	
ENSG00000119048	47,00676387	59,09794535	7,249282657	-0,327755097	1,18E-05	ubiquitin conjugating enzym	UBE2B	
ENSG00000165506	14,80213903	18,55015059	5,601185494	-0,32539207	0,006589376	dynein axonemal assembly f	DNAAF2	
ENSG00000008710	167,7034078	208,0990059	9,186718726	-0,314257971	0,006735141	polycystin 1, transient recep	PKD1	
ENSG00000141551	262,2071288	326,4029678	9,768508669	-0,3128318	7,22E-06	casein kinase 1 delta	CSNK1D	
ENSG00000106012	40,49491938	50,26079182	7,065322607	-0,307715625	0,00107776	IQ motif containing E	IQCE	
ENSG00000172915	22,73893037	28,09381405	6,400231971	-0,304798985	0,046911355	neurobeachin	NBEA	
ENSG00000104450	18,72133165	23,1725895	5,777601921	-0,302152063	0,027909049	sperm associated antigen 1	SPAG1	
ENSG00000107404	125,06815	154,44437	8,752763738	-0,301321358	8,86E-06	dishevelled segment polarity	DVL1	
ENSG00000119977	53,88388319	66,42735478	7,547945244	-0,297555433	0,006294423	tectonic family member 3	TCTN3	
ENSG00000090565	114,2361614	140,0924748	8,496118291	-0,294875321	3,30E-05	RAB11 family interacting pro	RAB11FIP3	
ENSG00000135912	42,02706147	51,23210512	7,114286845	-0,293510349	0,037726208	tubulin tyrosine ligase like 4	TTLL4	
ENSG00000127914	100,6378457	123,2471957	8,371586381	-0,293070665	0,01240676	A-kinase anchoring protein 9	AKAP9	
ENSG00000078142	47,95112723	58,29863002	7,341951948	-0,282701168	0,000157892	phosphatidylinositol 3-kinas	PIK3C3	
ENSG00000166171	14,34218917	17,45129094	5,727747889	-0,27999216	0,014436421	deleted in primary ciliary dy	DPCD	
ENSG00000108946	180,9833736	219,4446542	9,200533449	-0,274945512	0,011177001	protein kinase cAMP-depen	PRKAR1A	
ENSG00000147400	35,76263421	43,17093636	7,021855368	-0,267003652	0,032234189	centrin 2	CETN2	
ENSG00000007168	169,5990066	202,6829601	9,073859228	-0,258516731	1,03E-08	platelet activating factor ace	PAFAH1B1	
ENSG00000069966	54,27420578	64,78297351	7,331236722	-0,256989561	0,02335808	G protein subunit beta 5	GNB5	
ENSG00000103769	140,2324941	167,5412138	8,797280791	-0,252741258	0,013162042	RAB11A, member RAS oncog	RAB11A	
ENSG00000084733	180,4534326	213,7655274	9,187622579	-0,241812971	0,000173892	RAB10, member RAS oncoge	RAB10	
ENSG00000198707	41,90534373	49,60689934	7,115408229	-0,239093859	0,017494379	centrosomal protein 290	CEP290	
ENSG00000007047	82,48756298	97,29101019	8,088478397	-0,23737511	0,007198523	microtubule affinity regulati	MARK4	
ENSG00000179387	29,89396581	35,08880542	6,645286614	-0,228769047	0,042235959	ELMO domain containing 2	ELMOD2	
ENSG00000116198	51,44128096	60,40818751	7,400200553	-0,228268097	0,002595366	centrosomal protein 104	CEP104	
ENSG00000125971	95,33286664	110,3779284	8,290610687	-0,207925357	0,026292754	dynein light chain roadblock	DYNLRB1	
ENSG00000119650	16,62333504	19,16209099	5,924862207	-0,201395993	0,004398977	intraflagellar transport 43	IFT43	
ENSG00000180104	59,08563784	68,06452335	7,73066053	-0,200148352	0,004884762	exocyst complex component	EXOC3	
ENSG00000168036	510,0541533	584,1618208	10,67547217	-0,19615837	0,000449565	catenin beta 1	CTNNB1	
ENSG00000100554	68,26649308	77,89079327	7,697178116	-0,191224398	0,017626368	ATPase H+ transporting V1 s	ATP6V1D	
ENSG00000171703	44,86131308	51,178371	7,360752389	-0,189259378	0,007442995	transcription elongation fact	TCEA2	
ENSG00000160218	43,63450554	49,79357598	7,071640443	-0,187331244	0,00069465	trafficking protein particle cc	TRAPPC10	
ENSG00000171497	52,84347332	60,0940497	7,360167451	-0,182243863	0,001829881	peptidylprolyl isomerase D	PPID	
ENSG00000100815	80,47523325	91,21226812	8,155211946	-0,178176905	0,030953539	thyroid hormone receptor ir	TRIP11	
ENSG00000124659	21,43177143	24,16858352	6,131066167	-0,170659813	0,04956274	tubulin folding cofactor C	TBCC	
ENSG00000025796	97,99961702	110,229354	8,340874307	-0,169606589	0,005685477	SEC63 homolog, protein trar	SEC63	
ENSG00000160007	300,8065935	338,5239879	9,928609977	-0,167325045	0,028404732	Rho GTPase activating prote	ARHGAP35	
ENSG00000125863	23,9942051	26,98327782	6,415313822	-0,167137723	0,011842057	MKKS centrosomal shuttling	MKKS	
ENSG00000119509	27,14956586	30,2287911	6,413915711	-0,154489271	0,040661743	inversin	INVS	

ENSG00000156515	245,2380966	273,5331936	9,710790648	-0,154426099	0,001134803	hexokinase 1	HK1
ENSG00000109756	50,94466275	56,6122166	7,403904056	-0,151600729	0,030074718	Rap guanine nucleotide exch	RAPGEF2
ENSG00000069329	176,1245944	195,1442124	9,156902938	-0,146495524	0,021259033	VPS35 retromer complex co	VPS35
ENSG00000015676	113,3407881	124,3827215	8,511622613	-0,132100617	0,026916433	NudC domain containing 3	NUDCD3
ENSG00000170027	320,3570736	344,7511021	9,963520289	-0,105409729	0,048147098	tyrosine 3-monooxygenase/	YWHAG
ENSG00000142208	263,8570878	243,1793981	9,53471064	0,120392708	0,011707383	AKT serine/threonine kinase	AKT1
ENSG00000086061	257,4121747	232,3980748	9,406059246	0,14987657	0,03924155	DnaJ heat shock protein fam	DNAJA1
ENSG00000204435	21,35378944	19,04034829	6,014305872	0,169775995	0,036671203	casein kinase 2 beta	CSNK2B
ENSG00000167461	109,3255268	96,81570311	8,316072661	0,173199705	0,010291029	RAB8A, member RAS oncoge	RAB8A
ENSG00000067225	2216,64661	1940,904174	12,72004948	0,194935222	0,008595903	pyruvate kinase M1/2	PKM
ENSG00000137822	60,6066683	52,66694411	7,321138082	0,206306575	0,025213517	tubulin gamma complex ass	TUBGCP4
ENSG00000090273	184,6796832	158,9253816	8,983355008	0,211679829	0,013613674	nuclear distribution C, dyne	NUDC
ENSG00000106524	30,97618763	26,63171426	6,560018228	0,219755537	0,028849403	ankyrin repeat and MYND do	ANKMY2
ENSG00000144848	55,10191631	47,17544679	7,368899421	0,220676582	0,005788091	autophagy related 3	ATG3
ENSG00000126883	116,5835395	99,66435985	8,465799478	0,226523674	0,009687026	nucleoporin 214	NUP214
ENSG00000137100	33,85367537	28,92744199	6,742145394	0,227858138	0,010804101	dynactin subunit 3	DCTN3
ENSG00000147044	110,7306848	93,19635559	8,64909192	0,24842217	0,04952883	calcium/calmodulin depend	CASK
ENSG00000124074	26,64264915	22,39749391	6,241954738	0,253506922	0,011824485	enkurin domain containing 1	ENKD1
ENSG00000100578	25,23756146	21,10138914	6,052370752	0,253615699	0,04877785	KIAA0586	KIAA0586
ENSG00000182504	28,05396008	23,19786227	6,192088831	0,271106655	0,035849796	centrosomal protein 97	CEP97
ENSG00000119685	66,0156449	54,06293525	7,481957558	0,281411471	0,003594488	tubulin tyrosine ligase like 5	TTLL5
ENSG00000075188	19,11212527	15,59033284	5,769100393	0,28315434	0,019484295	nucleoporin 37	NUP37
ENSG00000147874	65,40137646	53,3662814	7,516293744	0,28422868	0,029250373	HAUS augmin like complex s	HAUS6
ENSG00000135469	13,60578401	11,10011638	5,178519641	0,285544844	0,009122816	coenzyme Q10A	COQ10A
ENSG00000175416	71,14493442	58,39598383	7,78805487	0,288031853	0,011444849	clathrin light chain B	CLTB
ENSG00000080824	1438,299992	1161,741261	11,89282487	0,295734619	0,003308393	heat shock protein 90 alpha	HSP90AA1
ENSG00000138031	168,3721333	135,4138702	8,719438486	0,305793788	0,027914226	adenylate cyclase 3	ADCY3
ENSG00000141367	527,8256057	419,5755645	10,76599015	0,319145593	0,012224936	clathrin heavy chain	CLTC
ENSG00000143951	4,915897149	3,938965697	3,864424647	0,322484862	0,024360774	WD repeat containing plana	WDPCP
ENSG00000213024	146,4929564	116,6842495	8,619465865	0,325800068	0,001083586	nucleoporin 62	NUP62
ENSG00000196924	2817,011115	2247,399776	13,0485148	0,330308218	0,001602767	filamin A	FLNA
ENSG00000175216	208,4406336	163,9387473	9,079008676	0,332693297	0,013162042	cytoskeleton associated pro	CKAP5
ENSG00000106992	21,55137796	16,99517384	5,844332981	0,337193864	0,001589058	adenylate kinase 1	AK1
ENSG00000136861	112,2565439	88,25747529	8,367138994	0,338352816	0,010171383	CDK5 regulatory subunit ass	CDK5RAP2
ENSG00000164070	157,6383779	123,4710204	8,613721	0,340841009	0,030856092	heat shock protein family A	HSPA4L
ENSG00000203778	9,389770878	7,422311006	5,014894707	0,342219468	0,025268496	family with sequence similar	FAM229B
ENSG00000110047	132,7890737	104,9357804	8,597246872	0,343064761	2,83E-05	EH domain containing 1	EHD1
ENSG00000054282	29,21495524	22,91152223	6,207568615	0,345202413	0,00544089	SHH signaling and ciliogenes	SDCCAG8
ENSG00000136425	23,26253179	18,31824317	6,112805735	0,346543704	0,021066465	calcium and integrin binding	CIB2
ENSG00000126216	46,4467717	36,17671569	6,865710902	0,353273381	0,000510259	tubulin gamma complex ass	TUBGCP3
ENSG00000188229	572,6385346	427,8211519	10,71570839	0,363073967	0,007318651	tubulin beta 4B class IVb	TUBB4B
ENSG00000103540	63,97582485	49,35139281	7,221396034	0,367319678	0,001277811	centriolar coiled-coil protein	CCP110
ENSG00000081870	30,69507894	23,74750291	6,254448124	0,368835347	0,000301755	heat shock protein family B	HSPB11
ENSG00000109062	119,5519871	91,19575593	8,215835685	0,383230325	0,002709468	SLC9A3 regulator 1	SLC9A3R1
ENSG00000158079	17,26662389	13,19727363	5,652398746	0,384792351	2,47E-05	protein tyrosine phosphatas	PTPDC1
ENSG00000139350	56,43706779	42,98719994	7,247075104	0,386778126	0,000491127	NEDD1 gamma-tubulin ring	NEDD1
ENSG00000136811	92,10803503	69,42264595	7,997724436	0,392805733	0,00462862	outer dense fiber of sperm t	ODF2
ENSG00000213186	19,94678919	15,09717127	5,779650779	0,393493363	0,004790234	tripartite motif containing 5	TRIM59
ENSG00000102900	77,10191588	58,26049226	7,797395738	0,399293092	2,38E-06	nucleoporin 93	NUP93
ENSG00000168014	73,7702197	54,69318373	7,682185416	0,403916132	0,000175753	C2 domain containing 3 cent	C2CD3
ENSG00000099901	140,9735065	105,7729913	8,467909827	0,413953228	0,000195943	RAN binding protein 1	RANBP1
ENSG00000196230	1505,991077	1118,732304	12,18711036	0,417609639	3,57E-06	tubulin beta class I	TUBB
ENSG00000158163	15,90025857	11,80346059	5,310708868	0,425868251	0,000484749	DAZ interacting zinc finger p	DZIP1L
ENSG00000130413	14,29124539	10,57922532	5,418030387	0,428061966	0,028995679	serine/threonine kinase 33	STK33
ENSG00000204390	3,650762954	2,717326336	3,565591445	0,43105459	0,015295781	heat shock protein family A	HSPA1L
ENSG00000154429	19,7463023	14,56668408	5,789861028	0,438603562	0,018238722	centriole, cilia and spindle a	CCSAP
ENSG00000148019	76,87344953	55,900952	7,606422839	0,442715935	0,00056866	centrosomal protein 78	CEP78
ENSG00000103021	5,988546495	4,360326728	4,563754414	0,447347996	0,024184921	coiled-coil domain containin	CCDC113
ENSG00000168778	26,77869835	19,29262272	6,265044785	0,450478503	0,0002757	tectonic family member 2	TCTN2
ENSG00000101624	15,96228382	11,46199318	5,421750691	0,459727072	0,006409312	centrosomal protein 76	CEP76
ENSG00000109971	1340,739308	965,2350007	11,69624157	0,468372724	6,73E-05	heat shock protein family A	HSPA8
ENSG00000152061	29,91062227	21,41016827	6,266943147	0,479282936	8,65E-07	RAB GTPase activating prote	RABGAP1L
ENSG00000127824	170,7238151	122,1878461	8,911529127	0,486108591	0,021052883	tubulin alpha 4a	TUBA4A
ENSG00000137267	57,72109734	40,21668139	7,815112424	0,497296315	0,033402502	tubulin beta 2A class IIa	TUBB2A
ENSG00000155755	31,56745298	22,33951225	6,538675747	0,501652439	0,009031165	transmembrane protein 237	TMEM237
ENSG00000152240	29,17440754	20,52762708	6,090905723	0,509201657	0,000774608	HAUS augmin like complex s	HAUS1
ENSG00000151466	18,64663943	12,74373419	5,760777449	0,519698979	3,60E-08	sodium channel and clathrin	SCLT1
ENSG00000070087	174,031904	118,0871735	9,022034217	0,552813607	0,000572902	profilin 2	PFN2
ENSG00000112877	11,75875354	7,755375061	5,063583488	0,561133049	0,008275394	centrosomal protein 72	CEP72
ENSG00000187391	4,274123641	2,86353601	3,806964351	0,576760335	0,032690993	membrane associated guany	MAGI2
ENSG00000149489	6,197598173	4,163428676	3,901715133	0,584542628	0,00760079	retinal outer segment meml	ROM1
ENSG00000166813	28,16915872	18,68099848	6,329054746	0,585203733	0,004265413	kinesin family member 7	KIF7
ENSG00000154678	25,41226082	16,94356301	6,116098615	0,599624695	0,029380824	phosphodiesterase 1C	PDE1C
ENSG00000167553	240,7035831	154,2012433	9,540190378	0,611083895	0,000481053	tubulin alpha 1c	TUBA1C
ENSG00000119397	21,52314365	13,95816745	5,612242349	0,612692171	1,04E-05	centriolin	CNTRL
ENSG00000173210	149,9415682	93,00416438	8,485494446	0,626989048	0,007808243	actin binding LIM protein fa	ABLIM3
ENSG00000131351	21,86415695	13,40126417	5,807578751	0,644217386	0,000527247	HAUS augmin like complex s	HAUS8
ENSG00000103995	23,87749535	14,4721217	5,992929366	0,661920259	0,001936991	centrosomal protein 152	CEP152
ENSG00000123416	871,6896791	535,0355004	11,23289385	0,679762036	7,58E-13	tubulin alpha 1b	TUBA1B
ENSG00000156876	17,43083981	10,47720355	5,648662054	0,700492803	6,61E-06	SAS-6 centriolar assembly pr	SASS6
ENSG00000188878	21,81227118	13,01377786	5,76954412	0,70461992	2,55E-05	Fas binding factor 1	FBF1
ENSG00000167216	4,975136948	2,87521485	4,235680705	0,706763964	0,001440448	katanin catalytic subunit A1	KATNAL2
ENSG00000176225	34,92290053	20,92478732	6,514260153	0,707056198	1,10E-05	rotatin	RTTN
ENSG00000074047	32,06576262	19,28040201	6,250790528	0,712545561	0,04275328	GLI family zinc finger 2	GLI2
ENSG00000117650	26,58751658	14,44641761	5,711895672	0,752536071	0,001120629	NIMA related kinase 2	NEK2
ENSG00000146038	198,6828155	117,8912645	8,795132619	0,759098959	3,77E-05	doublecortin domain contai	DCDC2
ENSG00000151849	33,95325562	18,98346047	6,654236785	0,772274587	3,10E-05	centromere protein J	CENPJ
ENSG00000126001	194,5196866	110,0034592	8,945475943	0,826576976	0,000292219	centrosomal protein 250	CEP250
ENSG00000004838	2,291807124	1,198264183	2,296862804	0,846852959	0,01895812	zinc finger MYND-type conta	ZMYND10
ENSG00000140403	43,70807775	23,29077248	6,540492925	0,863851825	0,000334877	DnaJ heat shock protein fam	DNAJA4
ENSG00000085840	22,05524343	11,53703237	5,534881799	0,86406689	0,000595739	origin recognition complex s	ORC1
ENSG00000155761	4,389313575	2,42566665	3,481013183	0,864529763	0,014051947	sperm associated antigen 17	SPAG17
ENSG00000164087	41,87690769	20,97052143	6,910637669	0,906092037	9,05E-05	POC1 centriolar protein A	POC1A
ENSG00000164104	129,2506452	64,24352551	8,044363744	0,931627639	1,51E-06	high mobility group box 2	HMGB2
ENSG00000171757	1,27910878	0,648675002	1,50763376	0,934576111	0,027235554	leucine rich repeat containir	LRRC34
ENSG00000249115	75,9529135	38,16359809	7,563180293	0,94883367	1,22E-07	HAUS augmin like complex s	HAUS5
ENSG00000080823	17,685751	8,593215106	5,921336803	0,973383883	0,041193853	MOK protein kinase	MOK
ENSG00000118513	3,152554113	1,612806032	2,655476988	0,981434184	0,006346721	MYB proto-oncogene, trans	MYB
ENSG00000079156	17,95144658	8,485473102	5,491177609	0,987767854	0,009791593	oxysterol binding protein lik	OSBPL6
ENSG00000132932	23,69764775	10,44932547	6,574046146	0,98903143	0,026510085	ATPase phospholipid transp	ATP8A2
ENSG00000117245	5,635447136	2,729164923	3,820520935	0,994456955	0,010038698	kinesin family member 17	KIF17
ENSG00000128408	11,94365528	5,579927978	4,336806288	1,036666589	0,000253179	RIB43A domain with coiled-c	RIBC2
ENSG00000105974	243,6103351	114,9579508	9,174358021	1,071612041	0,000129906	caveolin 1	CAV1
ENSG00000186638	25,42020836	10,85178327	6,031388594	1,088106347	1,06E-05	kinesin family member 24	KIF24
ENSG00000123473	49,54623157	21,73096241	6,826297358	1,092500184	2,01E-08	STIL centriolar assembly pro	STIL
ENSG00000168280	6,902636532	2,739786181	3,923113523	1,11766165	0,000446519	kinesin family member 5C	KIF5C
ENSG00000104892	1,318475291	0,570357285	1,301304831	1,128430175	0,028279281	kinesin light chain 3	KLC3
ENSG00000166963	27,73714395	13,18526748	6,310304079	1,133298669	0,014918231	microtubule associated prot	MAP1A
ENSG00000214140	6,998411092	3,085996658	4,374562626	1,139611568	0,000453234	photoreceptor disc compon	PRCD
ENSG00000154040	21,9438968	9,409876294	5,690161339	1,141632365	1,07E-05	calcium binding tyrosine pho	CABYR
ENSG00000160298	52,72753687	22,5486507	6,810909808	1,160490722	4,93E-08	chromosome 21 open readir	C21orf58

ENSG00000138587	12,60626881	4,690853673	4,950733805	1,195671652	3,16E-05	meiosis specific nuclear stru	MNS1
